# Analysis of protein-DNA interactions in chromatin by UV induced cross-linking and mass spectrometry

**DOI:** 10.1038/s41467-020-19047-7

**Published:** 2020-10-16

**Authors:** Alexandra Stützer, Luisa M. Welp, Monika Raabe, Timo Sachsenberg, Christin Kappert, Alexander Wulf, Andy M. Lau, Stefan-Sebastian David, Aleksandar Chernev, Katharina Kramer, Argyris Politis, Oliver Kohlbacher, Wolfgang Fischle, Henning Urlaub

**Affiliations:** 1 Bioanalytical Mass Spectrometry Group, Max Planck Institute for Biophysical Chemistry, 37077 Göttingen, Germany; 2 Institute for Bioinformatics and Medical Informatics, University of Tübingen, 72076 Tübingen, Germany; 3 Applied Bioinformatics, Department for Computer Science, University of Tübingen, 72076 Tübingen, Germany; 4 Somatosensory Signaling and Systems Biology Group, Max Planck Institute of Experimental Medicine, 37075 Göttingen, Germany; 5 Department of Chemistry, King’s College London, London, SE1 1DB UK; 6 Laboratory of Chromatin Biochemistry, Max Planck Institute for Biophysical Chemistry, 37077 Göttingen, Germany; 7 King Abdullah University of Science and Technology (KAUST), Biological and Environmental Science and Engineering Division, Laboratory of Chromatin Biochemistry, 23955 Thuwal, Saudi Arabia; 8 CSL Behring GmbH, 35041 Marburg, Germany; 9 Institute for Translational Bioinformatics, University Hospital Tübingen, 72076 Tübingen, Germany; 10 Biomolecular Interactions, Max Planck Institute for Developmental Biology, 72076 Tübingen, Germany; 11 Bioanalytics Group, Institute for Clinical Chemistry, University Medical Center Göttingen, 37075 Göttingen, Germany

**Keywords:** DNA, DNA-binding proteins, Proteomics, Mass spectrometry, Structural biology

## Abstract

Protein–DNA interactions are key to the functionality and stability of the genome. Identification and mapping of protein–DNA interaction interfaces and sites is crucial for understanding DNA-dependent processes. Here, we present a workflow that allows mass spectrometric (MS) identification of proteins in direct contact with DNA in reconstituted and native chromatin after cross-linking by ultraviolet (UV) light. Our approach enables the determination of contact interfaces at amino-acid level. With the example of chromatin-associated protein SCML2 we show that our technique allows differentiation of nucleosome-binding interfaces in distinct states. By UV cross-linking of isolated nuclei we determined the cross-linking sites of several factors including chromatin-modifying enzymes, demonstrating that our workflow is not restricted to reconstituted materials. As our approach can distinguish between protein–RNA and DNA interactions in one single experiment, we project that it will be possible to obtain insights into chromatin and its regulation in the future.

## Abstract (web summary)

Cross-linking mass spectrometry (XLMS) allows mapping of protein-protein and protein-RNA interactions, but the analysis of protein-DNA complexes remains challenging. Here, the authors develop a UV light-based XLMS workflow to determine protein-DNA interfaces in reconstituted chromatin and isolated nuclei.

## Introduction

Protein–DNA interactions control essential cellular processes, such as replication, transcription, repair and recombination. In consequence, a multitude of proteins is interacting with DNA at any given time. The most prominent DNA–protein complex of a eukaryotic cell is chromatin. In this, basic histone proteins associate with DNA in a repetitive manner to provide structural and functional organization of the genome. The fundamental building block of chromatin is the nucleosome core particle (NCP), which consists of 147 bp of DNA wrapped around a core of 8 histone proteins (2xH2A, 2xH2B, 2xH3, 2xH4). Linker DNA of various length connects NCPs and enables binding of linker histones (H1 type and H5), thereby establishing nucleosome units. Within chromatin, DNA and histones act as platforms for interaction with additional factors that control genome function^1,2^. Of particular importance in this regard are various chemical modifications of DNA and the histone proteins that serve as specific docking sites^3–5^. Technologies for mapping the genome-wide distribution of histone and DNA modifications, as well as chromatin-binding proteins are well-established^6,7^. In contrast, techniques for precise mapping of DNA-binding domains or single residues involved in DNA-binding are limited to simple, homogeneous protein–DNA complexes that are accessible to high-resolution techniques such as crystallography or NMR.

As others and we have shown, RNA-binding sites in proteins can readily be determined by UV-induced cross-linking at 254 nm in combination with mass spectrometry (MS)^8–10^. Similar workflows for DNA–protein cross-linking are not yet available, though it is well established that DNA–protein cross-links (DPC) are induced in vivo after exposure of cells to UV light, ionizing radiation or alkylating agents^11^, which lead to bulky DNA lesions^12^ for review^11,13,14^. UV irradiation of DNA triggers a cellular cascade termed DNA damage response (DDR) that involves a multitude of protein factors^15^ Also, UV irradiation has successfully been used to cross-link single-stranded (ss) and double-stranded (ds) DNA to proteins for the investigation of chromatin dynamics^16–19^. Therefore, UV-induced cross-linking combined with MS could be a valuable technique for investigating functional and structural relations in DNA–protein systems.

Here, we set out to investigate whether UV irradiation at 254 nm is sufficient to cross-link dsDNA efficiently to proteins with the aim of detecting cross-linking sites by MS down to the amino-acid level by using specialized data analysis^8,20,21^. We introduce an experimental and computational protein–DNA cross-linking workflow suitable for simple protein–DNA complexes such as (oligo)nucleosomes, chromatin-binding factors, as well as complex systems such as cell nuclei. In particular, our workflow allows simultaneous detection of protein cross-links to DNA and RNA in chromatin context and, thus, provides a comprehensive picture on interactions of nucleic acids and proteins.

## Results

### MS of linker and core histone UV cross-linked to dsDNA

It is well established that proteins such as histones can readily be cross-linked to dsDNA by chemical reagents^22–26^. However, UV cross-linking of proteins to dsDNA in combination with LC-MS/MS to track the sites of interaction has rarely been investigated.

**Fig. 1:**
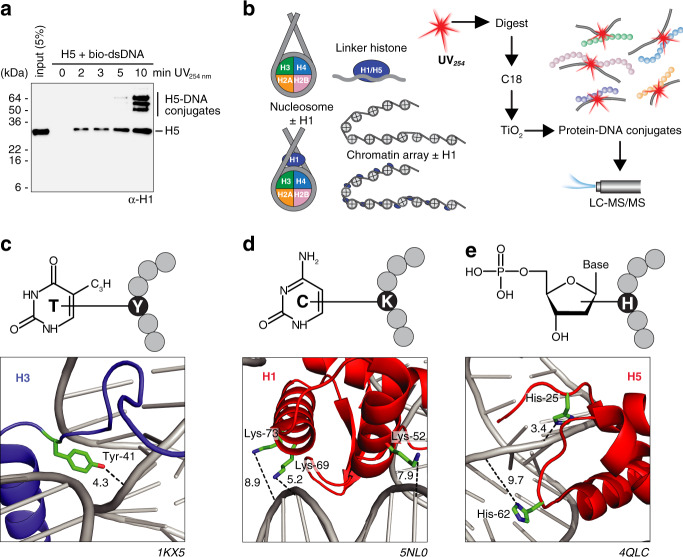
UV-induced protein-DNA cross-links in histones and nucleosomal samples. **a** Pull-down of UV (254 nm)-irradiated histone H5 and biotinylated DNA (187 bp). Samples were cross-linked for the indicated times and subjected to salt washes to remove unbound H5. Immobilized H5 was analyzed by western blotting with anti-H1. **b** Workflow for MS analysis of UV-induced cross-links in histone-DNA complexes, nucleosomes and chromatin arrays. **c**–**e** Protein-DNA cross-linking types identified in (linker) histones. **c** Thymidine-Tyrosine 41 cross-link of histone H3 highlighted in pdb entry 1KX5. **d** Cytosine-Lysine cross-links of histone H1 in pdb 5NL0. **e** Deoxyribose-Histidine cross-links of linker histone H5 in pdb 4QLC. Distances (in Ångstrom) were measured between residue side chains and nearest base or deoxyribose in the structure.

We first examined linker histones bound to dsDNA. We cross-linked native chicken linker histone H5 to a biotinylated DNA fragment (bio-dsDNA, 187 bp) by exposure to UV_254 nm_ for different periods of time. The H5-DNA complexes were captured and the cross-linked protein H5 was monitored by western blotting (Fig. 1a). We observed a time-dependent association of histone H5 with DNA and formation of high-molecular-weight aggregates, demonstrating covalent linkage of protein to dsDNA. We also observed free H5 and assume that DNA is mostly hydrolyzed during sample processing. We obtained similar results in gel-shift assays when irradiating histone H5 or recombinant human linker histone H1.4 with a short, radiolabeled dsDNA oligonucleotide (Supplementary Fig. 1a, b). Overall, these experiments show that UV irradiation at 254 nm cross-links histone proteins efficiently to dsDNA. We therefore adapted our established protein–RNA cross-linking MS workflow^8,27,28^ for detection of protein–DNA cross-links. We prepared linker histone–DNA complexes, as well as single nucleosomes and 12mer oligonucleosomal (‘chromatin’) arrays comprising *Xenopus laevis* core histones^29–31^; Fig. 1b). Following irradiation, DNA and protein complexes were hydrolyzed with DNA nuclease and trypsin to generate peptides, (oligo)nucleotides and cross-linked peptide–DNA oligonucleotide conjugates amenable to MS analysis. Non-cross-linked (oligo)nucleotides were removed by C18 reversed phase chromatography. In a final step, peptide–DNA conjugates were enriched by TiO_2_ affinity chromatography^8,27,28^; Fig. 1b). Purified peptide–DNA oligonucleotide conjugates were analyzed by LC-MS/MS and MS data were analyzed by the RNP^xl^ computational workflow^8,20^; Supplementary Fig. 1). In the analysis workflow, the exact mass of the precursor (i.e., peptide cross-linked to mononucleotides, dinucleotides, trinucleotides, or tetranucleotides) and the masses of fragment ions of the cross-linked peptide sequence with their specific mass adducts derived from the cross-linked (oligo)nucleotide moiety are searched against a sequence database^8,20^.

Early UV irradiation studies have indicated that all nucleobases of ssDNA can be cross-linked to amino acids by UV irradiation^32–35^. On the basis of our earlier results with RNA^8,33^, we expected mainly the pyrimidine bases thymine and cytosine to cross-link to proteins. The RNP^xl^ database search settings were changed to deoxyribose-nucleotide adducts and expected derivatives of attached DNA nucleotides (Supplementary Fig. 1c). The required exact masses of all theoretical thymine, cytosine, adenine and guanine adducts were calculated (Supplementary Fig. 1d, e), incorporated into the algorithm and searched against a database containing histone sequences. The MS analyses showed that peptides of core histones H3, H2A and linker histone H1.4 cross-linked to thymine (Fig. 1c; Supplementary Fig. 1f, g; Supplementary Data 1, 2, 3). The intact masses of cross-links show that cross-linked peptides contain different nucleotide combinations, without an apparent trend towards a particular nucleotide sequence (Supplementary Data 1, 2, 3). MS/MS spectra of cross-links usually permit the localization of the cross-linked amino acid by shifted y-type and b-type fragment ions series with an adduct mass corresponding to thymine nucleotide, nucleoside or base only (Supplementary Data 1, 2, 3). We mainly found lysine residues cross-linked to thymine, with the exception of one tyrosine and one proline residue in H3 and H1.4, respectively. Cross-links to cytosine also occurred with lysine residues and under the loss of ammonia. Cytosine cross-links were identified in all histones except for linker histone H5. Also, guanine (in H2A, H2B, H3, H4) and adenine (in H2B, H3, H4) cross-links were identified (Supplementary Fig. 1; Supplementary Data 1, 2, 3).

### UV cross-linking of the DNA deoxyribose moiety to histones

In some MS/MS spectra we observed a reoccurring mass shift of peptide fragment ions of 196 amu; this was attributed unambiguously to the deoxyribose phosphate moiety. The cross-linking potential of deoxyribose was unexpected, however, a possible photo-induced radical-based mechanism for deoxyribose has been described^36^. Cross-links of deoxyribose occurred specifically with histidine residues in linker histone H5, and core histones H2A, H2B, and H4 (Fig. 1d,e; Supplementary Fig. 1; Supplementary Data 1, 2, 3). In particular, H5 residues His-25 and His-62 represent two of the few cross-linkable amino acids that have been described in early studies of photo-induced cross-linking of DNA to histones^37^. We confirmed that all types of cross-links and identified adducts were of DNA origin by cross-linking histone H1.4 and H5 to isotopically labeled DNA (Supplementary Fig. 2a–c). For example, for cross-links to the deoxyribose phosphate moiety, we detected a mass shift of 201.0308 Da instead of 196.014 Da when using a fully ^13^C/^15^N-labeled DNA. This 5-Da increment originates from the five ^13^C-atoms in the labeled deoxyribose-PO_4_ adduct (^13^C5H9O8P) and cannot be explained by any other nucleobase adduct. Similarly, experiments with labeled DNA confirmed that thymine and cytosine bases are cross-linkable moieties of nucleotides (Supplementary Fig. 2a, b).

### Location of UV cross-linking sites in a nucleosome model

**Fig. 2:**
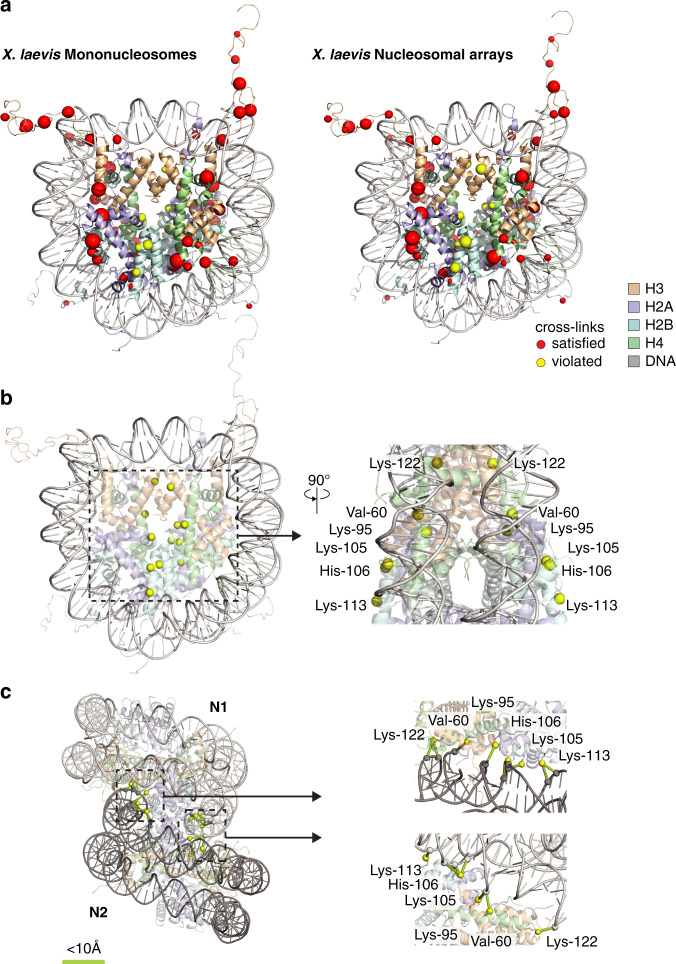
Correlation of DNA and cross-liking sites. **a** Nucleosome core particle structure (pdb 1KX5) with cross-linked amino acids (Cɑ) in *X. laevis* mononucleosomes or nucleosomal arrays highlighted as red or yellow spheres, representing cross-linking site that satisfy or violate distance restraints, respectively. The sphere scale is determined by the CSM count-rank of the cross-linked amino acid. **b** NCP structure as in **a**. Cross-links violating distance restraints are highlighted in yellow. Right panel, zoomed side view of the NCP and indicated cross-linked residues. **c** Model of the tetranucleosome (46) full-filling distance restraints for H2A Lys-95, H2B Lys-105, His-106 and Lys-113, H3 Lys-122, and H4 Val-60. Right panel, zoomed view of violated cross-links in the tetranucleosome. N1-nucleosome 1; N2-nucleosome 2.

UV_254 nm_ light induces zero-length cross-links between proteins and nucleic acids, i.e., only sites in immediate proximity are cross-linkable. This should be reflected by the relative positions of cross-linked amino acids and DNA in available 3D structures of linker histones in complexes with DNA and of nucleosomes^38^. For linker histones H5 and H1.4, we detected cross-links in the globular domain whose positions correlate well with distance restraints in the range of 10 Å, (Fig. 1d, e; Supplementary Fig. 1h). In case of H1.4, we also identified a number of cross-links in the unstructured C-terminal domain (CTD) that could not be assessed further owing to the lack of high-resolution structures of full-length linker histones bound to nucleosomes (Supplementary Fig. 1h). These cross-linking sites are in agreement with other studies^39–43^ that have demonstrated that the CTD of H1 binds to linker DNA and is important for binding of linker histones to chromatin^44^. Superimposition of all core histone cross-link sites with available nucleosome structures (e.g.,^45^ show that most cross-links cluster at the periphery of the nucleosomal core in close proximity to DNA (Fig. 2a). However, six sites (H2A Lys-95, H2B Lys-105, His-106, Lys-113, H3 Lys-122, and H4 Val-60) were positioned at distances greater than 25 Å from the nucleosomal DNA but reproducibly gave rise to well-annotated MS/MS spectra and cross-link identification (Supplementary Data 3). Since these cross-linking sites are placed toward the center of the nucleosomal core, facing away from the DNA (Fig. 2b), they cannot contact DNA of the same nucleosome. It is therefore conceivable that these sites represent cross-links between two individual nucleosomes. We therefore compared cross-links of histones in mononucleosomes with those in nucleosomal arrays in a semiquantitative manner (Fig. 2a; Supplementary Fig. 3). We calculated the ratio between cross-link spectrum matches (CSM) of individual cross-links and the total number of CSMs in the sample. In this calculation we did not consider differences in DNA adducts but only the individual cross-link sites within the respective histone sequences. The analysis revealed that those sites that do not meet the distance restraints are relatively more abundant in nucleosomal arrays compared with mononucleosomes (Fig. 2a; Supplementary Fig. 3). The fact that we still observe these cross-links in mononucleosomes (despite low spectral counts) might be due to interactions of two separate mononucleosomes in solution. Indeed, we were able to model such an interaction and generate a model in which each of the six cross-links could be explained by the protein sidechains interacting with DNA of an adjacent nucleosome. In this model, H2A Lys-95, H2B Lys-105, His-106, Lys-113, H3 Lys-122, and H4 Val-60 were each located within 10 Å to DNA (Fig. 2c). This conformation was generated using a current chromatin fiber model^46^, which required modest repositioning of two neighboring nucleosomes to bring protein cross-link sites and DNA closer together. However, the overall structure of nucleosomes in a chromatin model, including the way DNA is bent around the core histones, depends on several factors such as the DNA sequence itself, the modification state of DNA, nucleosome repeat length, the presence of linker histones, ionic strength etc.^31^. Hence, a generic structure that describes the arrangement of several nucleosome cores assembled in a chromatin-like structure might display different conformations.

### UV cross-linking of SCML2 to DNA

**Fig. 3:**
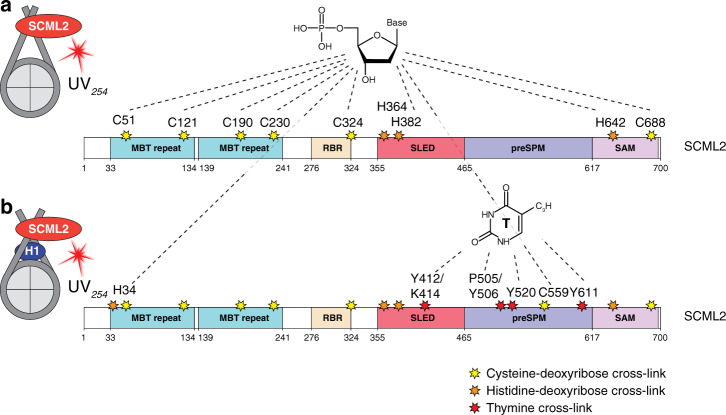
SCML2 cross-linking to nucleosomes. **a** Cysteine and histidine cross-links to deoxyribose-phosphates identified in UV-irradiated samples of the SCML2-nucleosome complex are highlighted with yellow or orange stars, respectively. SCML2 structure is shown schematically and protein domains are indicated. **b** Cross-links to deoxyribose-phosphate identified in SCML2-nucleosome+H1 irradiated samples are shown as in **a**). Cross-links to thymine are highlighted with red stars and marked by residue number. Non-marked stars represent cross-links to deoxyribose as described under **a**. Domains: MBT malignant brain tumor, RBR RING-BetweenRING-RING, SLED Scm-Like Embedded Domain, SAM sterile alpha motif, preSAM-aa466-616.

Having established reproducible cross-linking of histone proteins to dsDNA, we next tested whether contact sites of other proteins bound to nucleosomes can be determined by UV cross-linking. We investigated the interaction of human SCML2, an MBT-repeat containing Polycomb protein that is part of the PRC2 complex^47,48^. SCML2 binds methylated histones^49,50^, and interacts with RNA and DNA through its RBR and SLED domains^47,50,51^. We found that SCML2 binds to unmodified nucleosomes with no preference for the length of the DNA templates used during reconstitution, suggesting that it could bind both nucleosomal and linker DNA (Supplementary Fig. 4a). When irradiated together with nucleosomes and subjected to our MS-based workflow, we detected nine SCML2–DNA cross-linking sites. All of these sites represented histidine or cysteine residues cross-linking to the deoxyribose moiety of DNA (Fig. 3a; Supplementary Fig. 4b; Supplementary Data 4). This observation suggests that SCML2 interacts mainly with the sugar–phosphate backbone of dsDNA in unmodified nucleosomes. Next, we investigated whether the cross-linking pattern of SCML2 to nucleosomes changes in the presence of linker histone H1.4, since linker DNA was recently shown to be a major determinant of PRC2 recruitment to nucleosomes^52,53^. In the presence of linker histone, we detected the same cross-liking sites of SCML2 to the deoxyribose backbone of nucleosomal DNA as before plus His-34 and Cys-559. In addition, we identified cross-linking sites in the SLED domain (Tyr-412/Lys-414) and in a putatively unstructured region (preSAM: aa 466-616, Pro-505/Tyr-506, Tyr-520, Tyr-611) of SCML2 (Fig. 3b). The preSAM region did not display any DNA cross-links in the absence of linker histones (Fig. 3a, b; Supplementary Fig. 4b). In comparison with cross-links observed in the SCML2-nucleosome complex, most of the additional cross-links were to thymines through lysine or tyrosine residues and not to deoxyribose (Fig. 3b). Quantification of MS signal intensities (XIC) and CSM ratio (see above) of cross-linked peptides confirmed that these SCML2 sites interacted with bases of DNA only in the presence of linker histone (Fig. 3b; Supplementary Fig. 4d, e, f). Surprisingly, evaluation of a non-cross-linked control, with mononucleosomes ±H1.4 and SCML2 showed that cysteine cross-links are also present with similar spectral counts and MS2 spectrum quality in the non-UV irradiated controls (Supplementary Fig. 4f; Supplementary Data 4). This unexpected observation might be explained by cysteine residues being highly susceptible to cross-linking with the phosphate ribose backbone of nucleosomal DNA that laboratory light is sufficient to induce such cross-links (see Discussion). We performed gel shift assays of SCML2 with mononucleosomes in the presence and absence of H1.4 to investigate the importance of H1.4 for binding of SCML2. We found no effect of H1.4 on the binding affinity of SCML2 to mononucleosomes (Supplementary Fig. 4g), but observed that the interaction of SCML2 with mononucleosomes was reduced upon deletion of the preSAM region. While gel shift experiments can be used to roughly test binding strength, they cannot discriminate between different binding behaviors. The conformational changes introduced to the linker DNA by binding of linker histones e.g.,^54^ influence how SCML2 contacts the DNA. In the presence of H1.4, SCML2 switches from ‘sugar–phosphate only’ to ‘sugar–phosphate and nucleobase’ interactions with dsDNA. This difference cannot be observed by gel shift assays. Our results establish that interaction of common DNA-binding factors with nucleosomes can be reliably analyzed by UV cross-linking in vitro. The findings further indicate that UV cross-linking can be applied to monitor differences in interactions between DNA-binding proteins and dsDNA.

### UV cross-linking of DNA-binding proteins in native chromatin

We next wanted to apply our UV cross-linking MS approach to a sample of higher complexity and cross-linked a sample that contained purified native human mononucleosomes, as well as a significant number of other (DNA-)binding proteins (Supplementary Fig. 5; Supplementary Data 5). In this sample, we found most of the core histone cross-links that have been identified in the *X. laevis* nucleosomes described above (Supplementary Data 2, 6). In addition to the histone cross-links, we unambiguously identified 33 other proteins cross-linked to DNA. We identified DNA/chromatin-binding proteins such as TOP2A, BHE40, CAF1A, SUV92, ANM5, NSD2, PHF2, MBB1A, and also RNA-binding proteins that have been reported to interact with chromatin, like HNRDL, HNRPC and YBOX3^55,56^. We also identified RNA-binding proteins not yet reported to interact directly with DNA, such as SF3B4 and PTBP1. For both these cross-links, we identified thymine-base adducts on MS2 fragment ions enabling for an unambiguous DNA-cross-link assignment.

**Fig. 4:**
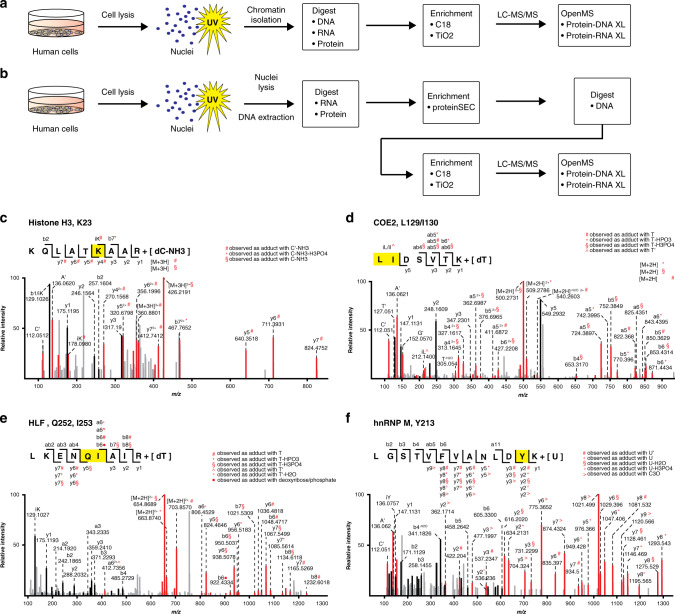
DNA-protein and RNA-protein cross-links in human cells. **a** Cross-linking workflow for human nuclei. Nuclei are isolated by hypotonic lysis, UV-irradiated and isolated chromatin is subjected to MS-sample preparation and cross-link analysis in OpenMS. **b** Cross-linking workflow for human nuclei with advanced cross-link enrichment strategy. Nuclei are isolated by NP-40 based lysis, UV-irradiated and genomic DNA is isolated using the DNAzol® Reagent. RNA and proteins are sequentially digested and the DNA-peptide mixture is separated by protein size-exclusion chromatography (proteinSEC). DNA-associated peptides are subjected to DNA digest, C18 and TiO2 enrichment, LC-MS/MS and cross-link analysis in OpenMS. **c** MS/MS spectrum of an H3 peptide cross-linked to deoxycytidine monophosphate (dC). **d** MS/MS spectrum of an COE2 peptide cross-linked to deoxythymidine monophosphate (dT). **e** MS/MS spectrum of a HLF peptide cross-linked to dT. **f** MS/MS spectrum of a HNRPM peptide cross-linked to uridine monophosphate (U). In each spectrum, the identified peptide sequence is shown and the cross-linked amino acid is highlighted in yellow. Black: fragment ions; Red: shifted ion fragments and marker ions.

Encouraged by these results showing that our workflow can be applied for the analysis of protein–DNA interactions in complex samples, we then isolated intact nuclei from HeLa cells and subjected these to UV irradiation. Chromatin was isolated from the cross linked nuclei using a method optimized for formaldehyde-induced cross-linking based on chromatin-precipitation (Fig. 4a)^57^. The isolated UV-irradiated chromatin fraction was digested with RNase, DNase and trypsin. The obtained mixture—peptides, cross-linked species and oligonucleotides—was further processed as described for recombinant chromatin (Fig. 4a). Overall, we sequenced peptides cross-linked to thymidine and deoxycytidine derived from 36 cross-linked proteins (Fig. 4c-e; Supplementary Data 7). Importantly, we detected exactly the same cross-link of a histone H3 peptide encompassing Lys-23 (Fig. 4c) that we had earlier observed with recombinant chromatin (Supplementary Data 3). Further analysis of proteins/peptides cross-linking to thymidine or cytosine identified several known DNA-binding proteins, including EBF2/COE2 (Fig. 4d), which is a helix-loop-helix transcription factor belonging to the COE2-family. The cross-linking site we identified is located directly in front of the zinc-finger motif of this protein. Indeed, a large subset of identified cross-linked proteins belongs to the Zn-Finger C2H2 family (ten out of 36) and most of the cross-linked peptides in these factors are located within or close to a zinc-finger domain (Supplementary Data 7). We further identified DNA-binding proteins containing leucine-zipper motifs (CEBPE, HLF (Fig. 4e)), coiled-coil regions (CEP85, P66A/B, and PDCD7) and ankyrin repeats (UACA). Strikingly, several of the cross-linked proteins we identified are associated with histones and nucleosome modification, such as BPTF, PHF19, and EP400. When we added adenine and guanosine adducts to the database search, we identified 15 additional proteins cross-linked to DNA, e.g., the zinc-finger motif containing proteins ZBT38, ZFHX2, ZNF98, and RPC4 (Supplementary Data 7, 8). Between the two biological replicates of our initial in nucleo experiment the overlap of identified cross-linked peptides, as well as of cross-linked proteins (ignoring differences in DNA adducts) was approximately 60%.

Surprisingly, in these initial in nucleo cross-linking experiments, we barely observed histone cross-links, with the exception of H3 (Supplementary Data 7, 8). We attribute this to the particular purification strategy used, employing TiO_2_-based enrichment of cross-linked peptides from isolated chromatin without any further steps to remove e.g., the multitude of phosphorylated peptides or peptide–RNA cross-links that are present in UV cross-linked and digested nuclei. These co-enriched peptides hamper MS-detection and subsequent sequencing of DNA cross-linked peptides e.g., from histones. To overcome this issue, we applied a more sophisticated enrichment strategy adapted specifically for purification of DNA-linked peptides (Fig. 4b). In addition to chromatin-isolation and final TiO_2_ affinity enrichment, we added a size-exclusion chromatography (SEC) step. By removing most RNAs by RNase digestion and trypsinizing proteins while keeping the DNA intact, we could successfully separate the larger-sized DNA–peptide cross-links from linear (phosphorylated) peptides and also from the vast majority of peptide–RNA oligonucleotide cross-links. Using this strategy, we identified 45 cross-linked proteins. Specifically, we found all core histones cross-linked to DNA (Supplementary Data 7, 8). Remarkably, we identified the same cross-linking sites in native human mononucleosomes (Supplementary Data 2, 6). We further found DNA-binding proteins such as SSBP and KMT2C cross-linked to DNA nucleotides and identified a multitude of proteins cross-linked to deoxyribose phosphate including several DNA-binding proteins.

### UV cross-linking of RNA-binding proteins in native chromatin

Beside DNA, RNA is another major component of cellular nuclei that is also susceptible to UV-induced protein–nucleic acid cross-linking. As mentioned above, we were able to identify RNA-binding proteins cross-linked to DNA. Conversely, we detected protein–RNA cross-links in addition to protein–DNA cross-links within the same sample using the appropriate RNP^xl^ search parameters for RNA^8,20^. We identified cross-links to well-described RNA-binding proteins such as hnRNP M (heterogeneous nuclear ribonucleoprotein M), a pre-mRNA-binding protein, with its RNA Recognition Motif (RRM) cross-linked to uracil (Fig. 4f).

Overall, our studies demonstrate that UV_254 nm_-induced cross-linking, purification and subsequent MS/MS analyses of peptides carrying deoxynucleotides is a straightforward approach to identify and map DNA-binding proteins in direct contact with DNA in complex cellular environments.

## Discussion

Cross-linking mass spectrometry of RNA–protein complexes has received much attention in recent years (references ^[e.g.,,58^, but a similar, straightforward approach for DNA–protein complexes is lacking. Here, we report a workflow for UV-induced DNA–protein cross-linking in combination with MS analysis that allows identification of protein regions that are cross-linked to dsDNA including the mapping of the cross-linked amino acid. Our approach makes use of the RNP^xl^ software tool, described previously^8,20^. It relies on identification of cross-linked species at the MS1 and MS2 level and leads to identification and detailed annotation of cross-linked spectra including fragment ions. The annotation of mass shifts in MS2 spectra is the basis for automated localization of the cross-linking site within a peptide. With our reported DNA mass adducts it is possible to analyze even complex datasets.

UV-based irradiation of DNA has been described for high-energy, laser-based applications^59–62^. However, we find that standard germicidal lamps with an emission maximum at 254 nm are sufficient to induce protein–DNA cross-links. During revision of our manuscript, Reim et al.^62^ published a study very similar to ours in which they used femtosecond laser irradiation at 258 nm to connect proteins covalently to DNA. They also applied a workflow similar to ours and cross-linking data annotation. Intriguingly, they found exactly the same cross-links of histones as we did in our study. We identified five types of cross-links in our chromatin samples: adenosine, cytosine, guanosine, thymine and deoxyribose cross-links. Distinct cross-linked histones peptides were repetitively found in all samples, and cross-linked peptides derived from homologous proteins were identified in different species (*X. laevis* und human) demonstrating the specificity and reliability of our cross-linking workflow. We observed various types of DNA adducts ranging from four to single nucleotides and their respective derivates in MS1 and MS2, such as loss of ammonia from adenosines, cytosines and guanosines. Surprisingly, in in vitro reconstituted *X. laevis* nucleosomes, we identified mainly lysines cross-linked to cytosines whereas in native HeLa mononucleosomes the minority of cross-links involve cytosines. A possible explanation could be that most lysines are post-translationally modified under native conditions preventing cross-linking reactions at these sites.

Relative quantitation of cross-linked peptides is possible when considering specific cross-linke spectrum matches (CSMs) versus all CSMs in the sample. Especially, spectral-counting-based semi-quantitative analyses have demonstrated that certain histone peptides are more frequently cross-linked to DNA in arrays than in mononucleosomes. These results support the interactions of two nucleosomes in an array consistent with current models of chromatin structure^46^. Moreover, relative quantification also revealed conformational changes in protein–DNA interaction of SCML2 in complex with nucleosomes.

A hitherto infrequently described cross-link type in all chromatin samples is the cross-link between deoxyribose phosphate and histidine or cysteine residues in proteins. Although radical formation of sugars in nucleic acids by photoactivation has been observed^63^. Cross-linked peptides of this type occurred in MS1 scans with oligonucleotides attached but were also observed with only the mass of deoxyribose phosphate added to the peptide-precursor. The UV cross-links of peptides only to the deoxyribose phosphate moiety, i.e., without the presence of additional nucleotides, might entail a certain ambiguity. However, the semiquantitative evaluation of our nucleosome samples reveals unambiguously that these are induced by UV cross-linking, i.e., histones harbor this particular modification only in cross-linked samples, while in non-cross-linked controls the modification is not detectable on histidines. Also, in the 3D structures of nucleosomes the histidines found to carry this modification are in close proximity to DNA (Fig. 2a). We therefore deduce, that most of these adducts indeed represent a true UV-induced cross-link and cannot be explained away as artefacts.

Cross-links are also found between deoxyribose phosphate and cysteine residues, as in SCML2 bound to nucleosomes. Surprisingly, such cysteine-deoxyribose phosphate conjugates are also present in non-UV cross-linked samples, i.e., in SCML2 bound to nucleosomes. A semiquantitative analysis based on CSM-counting shows that these conjugates are indeed equally present in non-UV and UV cross-linked samples. At present we cannot explain this finding. Cysteine residues are highly reactive towards nucleic acids in UV cross-linking^64^, and they might already form covalent bonds to nucleotides/nucleosides under ambient light. Indeed, cross-linking can be induced under laboratory light conditions, as it has been demonstrated for methylene blue, which cross-links proteins to dsRNA under a 60 W fluorescent lamp^65^. As for histidines, the location of cysteine cross-links in nucleic-acid-binding proteins, for which 3D structures are available, shows that these cysteine residues are in close proximity to the oligonucleotides.

Our SCML2-results show that cross-linking studies of recombinant complexes can support the characterization of known and unknown DNA-binding domains and extend sequence-alignment tools, mutation studies or gel-shift assays. The SCML2-nucleosome cross-linking experiment revealed that DNA-binding is not limited to the established SLED domain interaction^47^, but involves various sites in SCML2. Moreover, using H1-containing nucleosome substrates, we could quantitatively follow changes in interactions of SCML2 with DNA, which are not detected by band-shift assays. The fact that SCML2 binds differentially to nucleosomes carrying linker histones is supported by studies showing that the PRC2 complex can accommodate different lengths of linker DNA^53^ and that PRC2 binding to nucleosomes requires protein-free linker DNA^54^. Since no 3D structures of PRC2 with SCML2 are available, follow-up studies with SCML2 bound in PRC2 complexes are needed, to allow assessment of the biological implications of our observations. Still, it is not given that the strength of a given protein/peptide–DNA interaction can be reliably determined by quantifying cross-linked species in MS experiments. Indeed, the presence of H1.4 in our band-shift experiments had no effect on binding of SCML2 to nucleosomes. Instead, the deletion of the entire preSAM region, in which we observed the specific cross-links to the bases of nucleotides, resulted in less binding of SCML2 to nucleosomes irrespective of H1.4. Such discrepancies between band-shift assays monitoring binding affinities and cross-link experiments monitoring binding sites may provide insight into very specific interactions between proteins and nucleic acids, as already shown for protein–RNA complexes^8,66,67^. Similar seeming discrepancies have been observed in the analysis of protein–RNA complexes, where cross-linking unambiguously revealed a very specific interaction of an amino acid with its cognate RNA (also reflected in high-resolution 3D structures of protein–RNA complexes) while deletion or mutation of the cross-linked residues had no effect in band-shift experiments^66,67^.

We further demonstrated that our UV cross-linking approach is applicable to complex samples. We were able to identify cross-linked peptides derived from almost 80 different proteins containing known DNA-binding motifs when using cell nuclei. However, cross-linked peptides derived from histones (except H3) were rarely identified in our initial in nucleo experiments. An alternative enrichment strategy, including an additional purification step for large molecules of cross-linked DNA, enabled us to detect and sequence several cross-linked histone peptides. This highlights the need for a more sophisticated strategy for DNA purification and digestion to remove the multitude of phosphorylated peptides and peptide–RNA cross-links that are otherwise co-enriched by TiO_2_.

Obviously, proteins identified from UV_254 nm_-cross-linked cellular samples represent only a small fraction of the recently published chromatin proteomes^7,57,68,69^. Most of the proteome studies are based on formaldehyde cross-linking, which is reversed before MS analysis. Formaldehyde is a well-established protein–protein and protein–nucleic acid cross-linker. Therefore, formaldehyde cross-linking leaves a certain ambiguity as to whether proteins identified were in direct contact with DNA or were associated indirectly by protein–protein interactions with chromatin. In contrast, our UV cross-linking MS workflow identifies unambiguously proteins that interact directly with DNA. Moreover, our analysis can distinguish between protein–RNA and protein–DNA cross-links by database search in a single experiment. The DNA or RNA origin of a cross-linked nucleotide can be identified unambiguously in case a whole nucleotide including a deoxyribose or ribose is attached to the cross-linked peptide. However, if only a base is attached to the precursor, unambiguous assignment is not possible except for uracil and thymine.

For data analyses, we applied the RNP^xl^ tool from the OpenMS framework for mass spectrometry analyses. We note that RNP^xl^ in its current version does not support automated calculation of false discovery rates (FDR), which leaves a certain ambiguity in database search. Consequently, spectra have to be manually evaluated (Supplementary Note I). In addition, RNP^xl^ results needed to be carefully examined for removal of wrongly annotated phosphopeptides (e.g., neutral loss of H_3_PO_4_ although the precursor is assigned to a DNA cross-link nucleotide that already lost a 3’ or 5’ H_3_PO_4_/HPO_3_) and RNA-cross-links (nucleotide adducts that could correspond to DNA or RNA). We also noticed the presence of a relatively large number of non-annotated signals in MS2 spectra. These might have resulted from (i) co-isolation and fragmentation of co-eluting species, (ii) cross-linked DNA fragments cleaved off the peptide upon collision in the gas phase, (iii) internal fragments which are not yet considered in our RNP^xl^ settings, or (iv) cross-linked peptide fragment ions with DNA adducts of types yet unknown. The above-mentioned points, in combination with the lack of a proper FDR calculation, might explain why the majority of cross-linked peptides identified from complex samples were relatively short, as in these cases most of the fragment ions could be annotated.

Although RNP^xl^ in its current version still has some shortcomings, it is the only software tool available for automated annotation of cross-linked fragment ions. We envision that an improved version of RNP^xl^—including improved scoring, FDR calculation, and implementation of internal cross-linked fragment ions, cleaved oligonucleotide fragments and additional nucleotide adduct masses—will lead to a comprehensive, automated annotation of protein–nucleotide cross-links, satisfying the standards in database search for proteomics including PTM analyses.

## Methods

### Recombinant proteins and DNA templates

Recombinant core histones from *Xenopus laevis* and human linker histone H1.4 were expressed and purified as described before^7,29^; see Supplementary Methods for details). Chicken linker histone H5 was purchased from Abcam (ab81966).

Human full-length SCML2 (Q9UQR0) and SCML2 delta preSAM were overexpressed in BL21 CodonPlus (DE3)-RIL (Stratagene, La Jolla, USA) with a C-terminal His_6_-tag and purified by Ni^2+^-NTA chromatography (See Supplementary Methods for details).

The dsDNA-oligonucleotide (21 bp) was generated by annealing of two complementary oligonucleotides (Supplementary Fig. 1i; 21bp-fwd, 21bp-rev)^29^ and 5’-labeled with T4 polynucleotide kinase and γ-[^32^P]-ATP (6000 Ci/mmol) following standard procedures.

DNA fragments for mononucleosome reconstitution were generated by digest of the following plasmids: pUC18_52 × 187^70^ for the 187 bp (AvaI) and the 171 bp (digest with AvaI and NotI) fragments and pUC18_16 × 145 (gift from Song Tan^71^) (digest with EcoRV) for the 145 bp fragment. Reconstitution templates were isolated as described^72^. ^13^C/^15^N-labeled 187 bp DNA was generated by PCR under standard conditions using non-labeled 187 bp DNA as a template and ^13^C/^15^N-dNTPs (Sigma Aldrich; Silantes GmbH). Primer sequences are listed in Supplementary Fig. 1i (187-fwd, 187-rev). Biotinylated 187 bp DNA was generated by PCR using the same reverse primer but containing a 5’ biotin tag at the 187-fwd primer. Oligonucleosomes were reconstituted on a 12 × 200 × 601 DNA template that was prepared as described before^29,30,73^.

### Chromatin reconstitution

Oligonucleosomal arrays and nucleosome core particles were reconstituted by salt-gradient dialysis^74^ using recombinant histone protein octamers and the 12 × 200 × 601 or the 187 × 601 DNA templates. Linker histones were added to the reconstitution mixture in stoichiometric amounts at the start of dialysis^29^. Concentration of reconstituted oligonucleosomes and nucleosomal core particles was determined by measuring A_260_. All mass and molar concentrations thus reflect the DNA content.

### UV cross-linking

Cross-linking of in vitro reconstituted complexes was performed by spotting 50 μl droplets of protein–DNA complexes on a parafilm-coated metal block on ice. Irradiation at 254 nm was performed using an in-house built cross-linking apparatus as described^8^.

All DNA–linker histone complexes were incubated in XL buffer (20 mM Tris-HCl pH 7.5, 50 mM NaCl) for 30 min on ice before irradiation at 254 nm. For cross-linking with biotinylated 187 bp DNA fragments 0.25 nmol biotinylated-DNA and 0.225 nmol linker histones were incubated in 40 μl XL buffer and irradiated for 0, 2, 3, 5, or 10 min at 254 nm. The reaction mixture was added to 40 μl paramagnetic streptavidin-coated beads (MagneSphere, Promega) in 1 ml PD300 (20 mM HEPES-KOH pH 7.8, 0.3 M KCl, 0.2 mM EDTA, 10% [v/v] glycerol, 0.2% [v/v] Triton X-100) and incubated for 2 h at 4 °C under rotation. Beads were washed 4 times for 5 min with 1 ml PD800 (PD300 but 0.8 M KCl) for linker histone H5 or PD1000 (1 M KCl) when using H1.4. Bound proteins were eluted by incubation of magnetic beads in SDS sample buffer for 5 min at 95 °C. Samples were separated by SDS-PAGE and analyzed by western blotting against histone H1 (anti-H1, 1:1000, Active Motif 61201; for details see Supplementary Methods).

Experiments using the dsDNA oligonucleotide contained 100 pmol H5 or H1.4 and 1 pmol [^32^P]-labeled dsDNA oligonucleotide, which were cross-linked for 2 min (H5) or 2, 5, or 10 min (H1.4). The cross-linked samples were separated by SDS-PAGE, Coomassie-stained and autoradiographed. Gel scans and autoradiographs are displayed unprocessed in Supplementary Fig. 18.

Cross-linking experiments with 187 bp DNA and linker histones for MS analysis contained 50 μg DNA (0.43 nmol) and 33.5 μg H5 (1.6 nmol) or 30 μg H1.4 (1.4 nmol) in 250 μl XL buffer. Samples were irradiated for 5 or 10 min at 254 nm. Oligonucleosomes, as well as *X. laevis* mononucleosomes (each 60 μg) were cross-linked for 10 min at 254 nm in either reconstitution buffer (10 mM Tris-HCl pH 7.5, 25 mM NaCl, 1 mM EDTA, 2 mM DTT) or NaCl and MgCl_2_ were adjusted to a final concentration of 150 mM and 1 mM, respectively. The reaction volume was between 150 and 250 μl depending on nucleosome concentration.

For SCML2-nucleosome cross-linking, 50 μg of nucleosomes or 50 μg nucleosomes+H1.4 were incubated with 23.5 μg SCML2 (equals a molar SCML2: nucleosomal DNA ratio of 0.75) in low salt reconstitution buffer for 10 min at room temperature in a final volume of 400 μl.

Each cross-linking experiment involving recombinant histone samples included a non-cross-linked sample as control that was not irradiated but otherwise treated identically during sample preparation.

Nuclei from HeLa S3 cells were isolated by hypotonic lysis and mechanical disruption^76^ or by NP-40 based cell-lysis^77^. For cross-linking, 5 × 10^7^ nuclei were resuspended in 1 ml PBS with protease inhibitors (Roche, cOmplete EDTA-free).

HeLa nuclei and native mononucleosomes purified from human HeLa cells (52039; BPS Bioscience) were irradiated (254 nm) in a glass Petri dish for 10 min on ice^8^).

### Enrichment of UV-induced cross-links for MS analysis

Enrichment of UV-induced cross-links was performed according to established protocols for RNA-protein cross-linking^8,27,75^ with modifications. UV-irradiated and control samples were supplemented with 1 mM MgCl_2_ and 250 U Pierce^™^ universal nuclease (88700, Thermo Fisher Scientific) and incubated for 2 h at 37 °C. Proteins and protein–DNA conjugates were acetone-precipitated and pellets were first dissolved in 4 M urea, 50 mM Tris-HCl pH 7.9 and then diluted to 1 M urea by adding three volumes of 50 mM Tris-HCl pH 7.9. DNA was further hydrolyzed by addition of 250 U benzonase and incubated for 1–2 h at 37 °C. Protein hydrolysis was performed by addition of trypsin (sequencing grade, Promega) at a 1:20 mass ratio. After overnight incubation at 37 °C an additional 12.5 U of benzonase HC were added for 1 h at 37 °C followed by addition of trypsin at a 1:20 ratio for 1 h at 37 °C. Samples were desalted using C18 columns (ReproSil-Pur 120 Å, 5 μm, C18-AQ, Dr. Maisch, Germany) packed in-house as described for RNA-protein cross-links^8^. Desalted samples were enriched for cross-links by using in-house packed TiO_2_ columns (Titansphere 5 μm; GL Sciences, Japan). Samples were dissolved in buffer A (5% [v/v] glycerol, 80% [v/v] acetonitrile, 5% [v/v] trifluoroacetic acid (TFA)) and applied to TiO_2_ columns pre-washed with buffer B (80% [v/v] acetonitrile, 5% [v/v] TFA) followed by equilibration with buffer A. After sample application, the columns were washed three times with buffer A, four times with buffer B and once with buffer B2 (60% [v/v] acetonitrile, 0.1% [v/v] TFA). Samples were eluted with buffer C (0.3 M NH4OH, pH 10.5). The elution step was repeated twice. The eluate was dried in a speed vac.

### Enrichment of cross-links from native mononucleosomes

Nucleosomes were ethanol-precipitated and the pellet was dissolved in 4 M urea, 50 mM Tris-HCl pH 7.9. Then, the sample was diluted to 1 M urea by adding three volumes of 50 mM Tris-HCl pH 7.9. 500 U Quick CIP (M0525S, NEB) were added and the sample was incubated for 2 h at 37 °C. 1 mM MgCl_2_, 1 kU of Pierce^™^ universal nuclease, 20 U of DNase I (M0303S, NEB) and 1 kU of nuclease P1 (M0660S, NEB) were added for 2 h at 37 °C. Lys-C (mass-spec grade, Promega) was then added in a 1: 50 enzyme-to-protein ratio and the sample was incubated at 37 °C for 2 h. Trypsin digestion (1: 20 enzyme-to-protein ratio) was performed overnight at 37 °C. Finally, 1 kU of Pierce^™^ universal nuclease were added to the sample following incubation at 37 °C for 2 h. The sample was desalted and enriched as described for recombinant histone samples. The resulting eluate was dried in a speed vac and dissolved in 10 mM NH_4_OH pH10, 5% [v/v] acetonitrile (ACN). One-twentieth of the material was directly subjected to LC-MS/MS measurement as input sample. Residual peptides were loaded onto an Xbridge C18 column (186003128, Waters) using an Agilent 1100 series chromatography system. The column was operated at a flow rate of 60 μl/min with a buffer system consisting of 10 mM NH_4_OH pH10 (buffer A) and 10 mM NH_4_OH pH10, 80% [v/v] ACN (buffer B). The column was equilibrated with 5% buffer B and developed over 64 min using the following gradient: 5% buffer B (0–7 min), 8–30% buffer B (8–42 min), 30–50% buffer B (43–50 min), 90–95% buffer B (51–56 min), 5% buffer B (57–64 min). The first 6 min were collected as one flow-through fraction, followed by 48 ×1 min fractions, which were reduced to 12 fractions by concatenated pooling. The fractions were dried in a speed vac.

### Chromatin precipitation-based enrichment of UV-induced cross-links from HeLa nuclei for MS analysis

Nuclei were collected and pelleted at 1200×*g* for 5 min at 4 °C. Chromatin isolation from cross-linked nuclei was performed as described^57^. 2.5–5 × 10^7^ nuclei were digested with RNaseA (Thermo Fisher Scientific), followed by SDS and urea wash steps. After the final SDS wash step, the pellet was covered with 0.5 ml storage buffer (10 mM Tris-HCl pH 7.5, 1 mM EDTA, 25 mM NaCl, 10% glycerol, 1× protease inhibitors) and sonicated in a cooled water bath for 15 min, in alternating 30 s ‘on’ and 30 s ‘off’ cycles at the highest intensity setting (Bioruptor, Diagenode). Protein concentration was determined by a Bradford assay. The sonicated chromatin was adjusted to 0.05% SDS/1 mM MgCl_2_ and 250 U of Pierce universal nuclease were added. DNA digest was performed for 1 h at 37 °C and the reaction was stopped by adding five volumes of 100% acetone. After acetone precipitation the pellet was covered with 50 μl urea buffer (4 M urea, 50 mM Tris-HCl pH 7.5, 1 mM MgCl_2_) and sonicated in a cooled water bath for 10 min, in alternating 30 s ‘on’ and 30 s ‘off’ cycles at the highest intensity setting. The urea concentration was adjusted to 1 M with 50 mM Tris-HCl pH 7.5/1 mM MgCl_2_ and 250 U universal nuclease (Pierce) were added for 2 h at 37 °C. Trypsin digest was performed overnight at 37 °C with a 1:20 ratio of trypsin to protein. Peptides were desalted using a Sep-Pak tC18 1 cc Vac cartridge (Waters), followed by TiO_2_ affinity chromatography as described for histone proteins.

### SEC-based Enrichment of UV-induced cross-links from HeLa nuclei for MS analysis

2.5–5 × 10^7^ HeLa nuclei were collected and pelleted at 600×*g* for 5 min at 4 °C. Genomic DNA was isolated using DNAzol^™^ reagent (Invitrogen^™^) according to manufacturer’s instructions. Isolated DNA was dissolved in 8 mM NaOH and sonicated at 30% for 30 s (SFX150, tip diameter 3/32” (2.4 mm)). DNA was then ethanol-precipitated and dissolved in 1% SDS. The SDS concentration was diluted 1:10 and trypsin was added in a 1:60 enzyme-to-protein ratio. After overnight incubation at 37 °C, DNA was ethanol-precipitated and then dissolved in 4 M urea. Urea concentration was adjusted to 1 M and 40 μg of RNaseA and 4000 U of RNase T1 (Thermo Fisher Scientific) were added following incubation for 2 h at 37 °C. After ethanol-precipitation, the pellet was dissolved in 4 M urea following centrifugation at 17,000×*g* for 2 min. The supernatant was loaded onto a Superdex 200 Increase 10/300 GL (GE Healthcare) operated at a flow rate of 500 μl/min in 50 mM Tris/HCL pH 7.5, 2 mM EDTA. Fractions of 500 μl were collected and those containing both DNA and peptides were pooled and ethanol-precipitated. The pellet was dissolved in 4 M urea and then diluted 1:4. 1 mM MgCl_2_, 1000 kU of Pierce^™^ universal nuclease and 1 kU of nuclease P1 were added following incubation for 3 h at 37 °C. Another trypsin digest was performed overnight at 37 °C. The sample was desalted using a Sep-Pak tC18 1 cc Vac cartridge, following TiO_2_ affinity chromatography as described before.

### SDS-PAGE and in-gel digest of proteins from native mononucleosomes for proteomics

Ten microgram of native mononucleosomes were loaded onto a 4–12% NuPAGE Bis-Tris Gel (Invitrogen) and separated by SDS-PAGE. The entire lane was cut from the gel and into 23 slices that were treated according to^78^ (See Supplementary Methods for details). Peptides were dried in a speed vac followed by ESI-MS/MS analysis. A scan of the coomassie stained gel is displayed unprocessed in Supplementary Fig. 18.

### LC-MS/MS analysis

Sample pellets from TiO_2_ enrichment, high-pH reversed-phase chromatography or in-gel digestion were dissolved in 2% [v/v] acetonitrile, 0.05% [v/v] TFA. LC-MS/MS analyses were performed on Q Exactive mass spectrometers Plus, HF, HF-X or Exploris (Thermo Fisher Scientific) coupled to a nanoflow liquid chromatography system (1100 series, Agilent Technologies). Analytes were loaded on either an in-house-packed trapping column (2 cm; ReproSil-Pur 120 Å, 5 μm, C18-AQ; inner diameter, 150 μm) or a Pepmap 300 C18 column (Thermo Fisher Scientific) at a flow rate of 10–15 μl/min in buffer A (0.1% [v/v] formic acid) and washed for 3 min with buffer A. The sample was separated on an in-house-packed C18 column (30 cm; ReproSil-Pur 120 Å, 3 μm, C18-AQ; inner diameter, 75 μm) at a flow rate of 300 nl/min. Sample separation was performed using a linear gradient and a buffer system consisting of 0.1% [v/v] formic acid (buffer A) and 80% [v/v] acetonitrile, 0.08% [v/v] formic acid (buffer B). Linker histone-DNA cross-links were analyzed with a 38-min LC run, cross-linked *X. laevis* mononucleosomes, oligonucleosomal arrays, SCML2-nucleosome samples and native HeLa mononucleosomes were separated over 58 min and HeLa nuclei over 90 min. For histone-DNA and (oligo)nucleosome samples the column was equilibrated with 1% buffer B for 5 min and elution was performed with a linear gradient (2–48% B) over 19 or 44 min, followed by a wash step at 90% B and 95% B for 5 min each. For HeLa nuclei samples the column was equilibrated with 3% buffer B for 5 min and elution was performed with a linear gradient from 8 to 48% B over 75 min, followed by a wash step at 90% B and 95% B for 5 min each. Eluting peptides and heteroconjugates were analyzed online in positive mode using a data-dependent top 10, 15, 20, or 30 acquisition methods. MS1 and MS2 resolution were set to 120,000 and 30,000 FWHM, respectively for cross-link samples and to 60,000 and 15,000 FWHM for proteomics samples from in-gel digest. AGC targets were set to 10^6^ and 10^5^. Precursors selected during MS1 scans (scan range *m/z* 350-1600) were fragmented using higher-energy collision-induced dissociation (HCD) fragmentation. All MS/MS experiments with chromatin, SCML2, native mononucleosomes and HeLa nuclei employed 28% or 30% normalized collision energy (NCE). Linker histone-DNA samples were initially analyzed at 20%, 25%, and 30% NCE to test fragmentation behavior of heteroconjugates. Other MS/MS parameters were set as follows: isolation width, 1.4–1.6 *m/z*; dynamic exclusion, 9 s for cross-link samples, 30 s for proteomics samples; max. injection time (MS1/MS2), 50 ms/120 ms or 50 ms/60 ms. The lock mass option (*m/z* 445.120025) was used for internal calibration.

### Data analysis with MaxQuant

Proteomic analysis of MS-data from in-gel-digested native mononucleosomes and from cross-linked and non-cross-linked SCML2 samples was performed using MaxQuant software (version 1.6.0.1,^79^. For database search, a reviewed human database from Uniprot (downloaded 25.01.2019, 20,413 proteins) or a database containing all core histone sequences plus the SCML2 sequence was used for native mononucleosomes and SCML2 samples, respectively. Carbamidomethylation of cysteines was set as fixed and oxidation of methionines, as well as N-terminal acetylation were set as variable modifications. The maximum number of missed cleavages was set to two. The proteins from the output data were filtered for >2 unique peptides. For native mononucleosomes, the resulting list of proteins was used to create a sequence database for RNP^xl^ cross-link search.

### Data analysis with RNP^xl^

Data analysis and spectra validation were performed as described in^8^ with the RNP^xl^ tool^20^ in the OpenMS software network (https://www.openms.de/, Version 2.5.0). Briefly: Raw files were converted to the.mzML format, centroided and spectra matching to linear peptides and phosphopeptides were filtered out. If a control sample was present, it was aligned with the cross-link sample based on retention time and spectra corresponding to features also present in the control sample were removed. The filtered.mzML files were subjected to RNP^xl^ analysis (for DNA settings see Supplementary Fig. 1c, d, e). For linker-histone experiments, as well as for the initial analysis of chromatin-precipitation based in nucleo dataset (Supplementary Data 7, Chrtnprec_RNPxl_settings#1), we searched for C and T adducts only. The data obtained from reconstituted nucleosomes, native mononucleosomes and SEC-enriched in nucleo sample were searched including A, C, G and T adducts. In a second analysis of the chromatin-precipitation based in nucleo sample (Supplementary Data 7, Chrtnprec_RNPxl_settings#2) we also set up a search including A, C, G, and T. Other RNP^xl^ search parameters were set as follows: max. DNA adduct length, 4; *m/z* mass tolerance (MS1/MS2), 6 ppm/20 ppm; max. missed cleavages (HeLa nuclei/histones, chromatin, SCML2), 2/3. For a detailed documentation of OpenMS based cross-link data analysis, please refer to s.

### Quantification of cross-links

Skyline software (https://skyline.ms) was used to measure quantitative differences in identified SCML2-DNA cross-link precursors using the MS1 scans from nucleosome and nucleosome+H1 samples. A library was generated containing all transitions (*n* = 74) derived from cross-link data with SCML2 to extract precursor ion chromatograms (XIC). Picked precursors were examined manually for the presence of at least two isotope peaks and, when present, in a ± 2 min time window of measured retention times and an *m/z* match tolerance of ±5 ppm. For each transition the ratio of XIC[nucleosome]:XIC[nucleosome+H1] was calculated and XIC ratios were compared for cross-links to deoxyribose and thymine.

For spectral counting based cross-link quantification, we counted manually validated cross-link spectra from RNP^xl^ searches (CSMs, see Supplementary Note 1 for quality criteria) and divided this number by the total number of manually validated CSMs identified in the respective sample. In case of SCML2 data, the CSM count of individual cross-link sites was divided by the total number of SCML2 peptide spectrum matches (PSMs) that were identified by MaxQuant database search of the same sample.

### Modeling of nucleosome structures

In order to generate a tetranucleosome model in which protein-DNA cross-links were satisfied, the cryo-EM structure of the 12 × 177bp chromatin fiber (EMD-2600;^46^ was adjusted. The fitted cryo-EM models were kindly provided by Prof. Ping Zhu (Institute of Biophysics, Chinese Academy of Sciences). The relative position of each nucleosome within an adjacent pair, were adjusted by sliding each nucleosome past one another by approximately 10 Å which brought H2A, H2B, and H4 of each nucleosome unit together with the DNA of the adjacent unit. Cross-links between H2A Lys-95, H2B Lys-105, H2B Lys-113, and H4 Val-60 to any DNA backbone were monitored during translation to identify a position which brought each cross-link distance to within 10 Å distance. The overall model of the cross-link-satisfied tetranucleosome, differs through only relative positioning of the nucleosome stacks.

### Gel-shift assays

Five picomole mononucleosomes were incubated with 0.5, 1.0, or 2.0 molar equivalents of recombinant SCML2. In the gel shift assays testing SCML2’s binding to linker DNA, unmodified nucleosomes reconstituted on 145, 171, or 187 bp template DNA were incubated with FL SCML2. In the assays testing the effect of the H1.4 linker histone, unmodied and H1.4-containing nucleosomes were incubated with FL SCML2. In the assays testing the effect of the preSAM region, unmodified and H1.4-containing nucleosomes were incubated with recombinant FL and delta preSAM SCML2. The reactions were performed in 10 mM Tris.HCl pH 7.9, 150 mM NaCl, 1 mM EDTA and 1 mM DTT for 1 h, at 300 rpm and 16 °C. The protein-nucleosome complexes were resolved on 1% agarose in 18 mM Tris and 18 mM boric acid at 100 V for 1 h at 4 °C and stained after the run with EtBr. All gel-shift assays are displayed unprocessed in Supplementary Fig. 18.

### Reporting summary

Further information on research design is available in the Nature Research Reporting Summary linked to this article.

## Supplementary information

Supplementary Information

Description of Additional Supplementary Files

Supplementary Data 1

Supplementary Data 2

Supplementary Data 3

Supplementary Data 4

Supplementary Data 5

Supplementary Data 6

Supplementary Data 7

Supplementary Data 8

Reporting Summary

## Data Availability

The datasets (.raw,.mzML and.idxML) generated and analyzed during this study are available in the PRIDE repository under the accession number PXD020290.
