# Supplementary material for: Analysis of protein-DNA interactions in chromatin by UV induced cross-linking and mass spectrometry
Source: Nat Commun. 2020 Oct 16;11:5250. doi: 10.1038/s41467-020-19047-7 (PMC7567871; doi:10.1038/s41467-020-19047-7)
Supplement: Supplementary file 1 — Supplementary Information [file 41467_2020_19047_MOESM1_ESM.pdf]

Supplementary Information

Analysis of protein-DNA interactions in chromatin by UV induced cross-linking and mass spectrometry

Stützer *et al.*

List of contents

Supplementary Figure 1-5	p. 1-7
Supplementary Note 1	p. 8-16
Supplementary Figures 6-18	p. 10-17
Supplementary Methods	p. 18-19
Supplementary References	p. 20

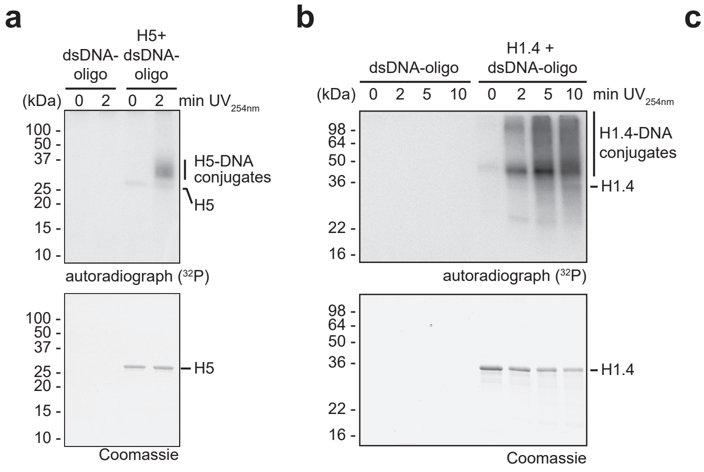

c

parameter	value	
database		
threads	8	
no_progress	true	
precursor		
mass_tolerance	6	
mass_tolerance_unit	ppm	
min_charge	2	
max_charge	5	
isotopes	[0, 1]	
fragment		
mass_tolerance	20	
mass_tolerance_unit	ppm	
modifications		
fixed	[]	
variable	[Oxidation (M)]	
variable_max_per_peptide	2	
peptide		
missed_cleavages	2	
enzyme	Trypsin	
RNPxl		
length	3	
sequence		
target_nucleotides	[A=C10H14N5O6P, C=C9H14N3O7P, G=C10H14N5O7P, T=C10H15N2O8P]	DNA nucleotides
mapping	[A->A, C->C, G->G, T->T]	
can_cross_link	T	
fragment_adducts	[T:C10H15N2O8P;T, T:C10H13N2O7P;T-H2O, T:C10H14N2O5;T-HPO3, T:C10H12N2O4;T-H3PO4, T:C5H6N2O2;T', T:C5H4N2O;T'-H2O, T:C5H9O6P;dribose, T:C5H7O5P;dribose-H2O, T:C5H8O3;dribose-HPO3]	expected DNA adducts
modifications	[T:, T:-H2O, T:-H2O-HPO3, T:-HPO3, T:+C5H7O5P, T:-C4H5N3O, T:+HPO3]	nucleotide modifications

d

Cross-link to	Nucleotide modification
T	-H2O<br>-H2O-HPO3<br>-HPO3<br>-C4H5N3O (base loss)
C	-H2O<br>-NH3<br>-H2O-HPO3<br>-HPO3<br>-NH3-HPO3
G	-H2O<br>-NH3<br>-H2O-HPO3<br>-HPO3<br>-NH3-HPO3
A	-H2O<br>-NH3<br>-H2O-HPO3<br>-HPO3<br>-NH3-HPO3<br>-C5H5N5 (base loss)
deoxyribose	-H2O<br>-H2O-HPO3<br>-HPO3

e

DNA adduct	Molecular formula
T	C10H15N2O8P1
T-H2O	C10H13N2O7P1
T-HPO3	C10H14N2O5
T-H3PO4	C10H12N2O4
T'	C5H6N2O2
T'-H2O	C5H4N2O1
C	C9H14N3O7P1
C-H2O	C9H12N3O6P1
C-NH3	C9H11N2O7P1
C-HPO3	C9H13N3O4
C-H3PO4	C9H11N3O3
C-NH3-HPO3	C9H10N2O4
C'	C4H5N3O1
C'-H2O	C4H3N3
C'-NH3	C4H2N2O1
G	C10H14N5O7P1
G-H2O	C10H12N5O6P1
G-NH3	C10H11N4O7P1
G-HPO3	C10H13N5O4
G-H3PO4	C10H11N5O3
G-NH3-HPO3	C10H10N4O4
G'	C5H5N5O1
G'-H2O	C5H3N5
G'-NH3	C5H2N4O1
A	C10H14N5O5P1
A-NH3	C10H11N4O6P1
A-H2O	C10H12N5O5P1
A-HPO3	C10H13N5O3
A-H3PO4	C10H11N5O2
A-NH3-HPO3	C10H10N5O3
A'	C5H5N5
A'-NH3	C5H2N4
deoxyribose/phosphate	C5H9O6P1
deoxyribose/phosphate-H2O	C5H7O5P1
deoxyribose	C5H8O3
13C/15N labeled DNA (examples)	
C'-NH3	(13)C4H2(15)N2O1
deoxyribose/phosphate	(13)C5H9O6P1

f

linker histone	peptide	(aa)	cross-linked aa	cross-linked DNA moiety
**H1.4**	**K**ASGPPVSELITK	(33-45)	Lys33	cytosine
	[K]ASG**PP**VSELITK	(33/34-45)	Pro37/38	thymine
	ASGPPVSELIT**K**AVAASK	(34-51)	Lys45	cytosine
	SGVSLAAL**K**K	(54-63)	Lys62	cytosine
	**K**ALAAAGYDVEK	(63-74)	Lys63*	cytosine
	ALAAAGYDVE**K**NNSR	(64-78)	Lys74	cytosine
	I**K**LGLK	(79-84)	Lys80*	cytosine/thymine
	LGL**K**SLVSK	(81-89)	Lys84*	cytosine
	SLVS**K**GTLVQTK	(85-96)	Lys89	cytosine
	GTGASGSF**K**LNK	(97-108)	Lys105	thymine
	GTGASGSF**K**LNKK	(97-109)	Lys105	cytosine
	**K**AASGEAKPK	(109-118)	Lys109	cytosine
	A**K**KPAGAAK	(127-135)	Lys128	cytosine
	KPAGAA**K**KPK	(129-138)	Lys135	cytosine
	**K**ATGAATPK	(139-147)	Lys147	cytosine
	KATGAATP**K**K	(139-148)	Lys156	cytosine
	**K**AKKPAAAAGAK	(156-167)	Lys167	cytosine
	KPAAAAGA**K**K	(159-168)	Lys167	cytosine
	**K**A**K**AAKPK	(174-181)	Lys174/176	cytosine
	A**K**AVKPK	(190-196)	Lys191	cytosine
	AVKP**K**AAKPK	(192-201)	Lys196	cytosine
	AAKP**K**TAKPK	(197-206)	Lys201	cytosine
	TAKP**K**AAKPK	(202-211)	Lys206	cytosine
**H5**	SAS**H**PTYSEMIAAAIR	(22-37)	His25*	deoxyribose
	VG**H**NADLQIK	(60-69)	His62*	deoxyribose

g

core histone	peptide	(aa)	cross-linked aa	cross-linked DNA moiety
**H3**	KSTGG**K**APR	(9-17)	Lys14	cytosine
	**K**QLATK	(18-23)	Lys18	cytosine
				thymine
	KQLAT**K**AAR	(18-26)	Lys23	cytosine
				adenine
	QLAT**K**AAR	(19-26)	Lys23	cytosine
	**K**SAPATGGVK	(27-36)	Lys27	cytosine
	SAPATGGV**K**KPHR	(28-40)	Lys36	cytosine
				adenine
				guanine
	**Y**RPGTVALR	(41-49)	Tyr41*	thymine
				guanine
	YQ**K**STELLIR	(54-63)	Lys56	cytosine
	**K**LPFQR	(64-69)	Lys64	cytosine
	EIAQDF**K**TDLR	(74-83)	Lys79	cytosine
	VTIMP**K**DIQLAR	(117-128)	Lys122	cytosine
				guanine
**H2A**	AKTRSSRAGLQFPVGR	(14-29)	n/d	thymine
	AGLQFP**V**GR	(21-29)	Val27	cytosine
				guanine
	IIPR**H**LQLAVR	(78-88)	His82	deoxyribose
	**H**LQLAVR	(82-88)	His82	deoxyribose
	**H**LQLAVRNDEELNK	(82-95)	His82	deoxyribose
	**H**LQLAVRNDEELNKLLGR	(82-99)	His82	deoxyribose
	NDEELN**K**LLGR	(89-99)	Lys95	cytosine
	VTIAQGGVLPNIQSVLLP**K**K	(100-119)	Lys118	cytosine
**H2B**	KAVT**K**TQK	(13-20)	Lys17	cytosine
	KESYAIYV**Y**K	(31-40)	Tyr39	cytosine
	ESYAIYV**Y**K	(32-40)	Tyr39	cytosine
	ESYAIYVY**K**VLK	(32-43)	Lys40	cytosine
	VL**K**QVHPDTGISSK	(41-54)	Lys43	cytosine
				guanine
				deoxyribose
	QV**H**PDTGISSK	(44-54)	His46	deoxyribose
	LA**H**YNK	(77-84)	His79	deoxyribose
	LA**H**YNK**R**	(77-84)	His79	deoxyribose
	EIQTA**V**R	(90-96)	Val95	cytosine
	LLLPGELA**K**HAVSEGTK	(97-113)	Lys105	cytosine
				adenine
				guanine
	**H**AVSEGTK	(106-113)	His106	deoxyribose
	HAVSEGT**K**AVTK	(106-117)	Lys113	cytosine
	AVT**K**YTSAK	(114-122)	Lys117	cytosine
**H4**	GG**K**GLGK	(6-12)	Lys8	cytosine
	GLG**K**GGAK	(9-16)	Lys12	cytosine
	GLG**K**GGAKR	(9-17)	Lys12	cytosine
	DNIQGIT**K**PAIR	(24-35)	Lys31	cytosine
				adenine
				guanine
	**V**FLENVIR	(60-67)	Val60	cytosine
	DAVTYTE**H**AK	(68-77)	His75	deoxyribose
	DAVTYTEHA**K**R	(68-78)	Lys77	cytosine
	**K**TVTAMDVVYALK	(79-91)	Lys79	cytosine
	T**V**TAMDVVYALKR	(80-92)	Val81	cytosine

h

```
H1.4   SETAPAAPAAPAPAEKTPVKKKARKSAGAAKRKASGPPVSELITKAVA   48
H5     TESLVLSPAPAKP-----------KRVKASRRSASHPTYSEMIAAAIR   37
                                          *
                     *                *   *
H1.4   ASKERSGVSLAALKKALAAAGYDVEKNNSRIKLGLKSLVSKGTLVQTK   96
H5     AEKSRGGSSRQSIQKYIKSHYKVGHNADLQIKLSIRRLLAAGVLKQTK   85
                               *

H1.4   GTGASGSFKLNKKAASGEAKPKAKKAGA--------AKAKKPAGAAKK   136
H5     GVGASGSFRLAKSDKAKRSPGKKKKAVRRSTSPKKAARPRKARSPAKK   133

H1.4   PKKATGAATPKKSAKKTPKKAKKPAAAAGA-KKAKSPKKAKAAKPKKA   183
H5     PKATARK--ARKKSRASPKKAKKPKTVKAKSRKASKAKKVKR------   173

H1.4   PKSPAKAKAVKPKAAKPKTAKPKAAKPKKAAAKKK                218
H5     --------------SKPRAKSGARKSPKKK-----                189
```

cross-linked peptide

cross-linked residue

* residues higlightes in Fig.1d & 1e

i

oligonucleotide	sequence 5'-3'	product
21bp-fwd	CCGAGGCTGTTCAATACATGC	21bp dsDNA
21bp-rev	CGTACATAACTTGTCGGAGCC	
187-fwd	CCGAGGCTGTTCAATACATG	187bp DNA
187-rev	GACCCTATACGCGGCCG	

Suppl. Fig. 1: UV-cross-linking of 32P-labeled oligonucleotide (21 bp) to a) linker histone H5 and b) linker histone H1.4 for indicated times. Reactions were separated by SDS-PAGE. Top, autoradiograph; Bottom, Coomassie-stained gel. c) RNPxl search configuration window showing the modifications introduced to search for DNA-protein cross-links. d) Table summarizing the most common nucleotide modifications detected in DNA-protein cross-links. e) Table summarizing the most common DNA adducts and their molecular formulas. f) Linker histone peptides identified to cross-link to DNA under UV-irradiation. Sequence, length, cross-linked amino acid and cross-linked DNA moiety are indicated. g) Summary of core histone cross-links as described under f). h) Sequence alignment (Clustal Omega) of human linker histones H1.4 (P10412) and chicken linker histone H5 (P02259) comparing sites of cross-linking. i) Oligonucleotide sequences used for generation of 21bp dsDNA and for PCR-based generation of 187bp DNA fragment.

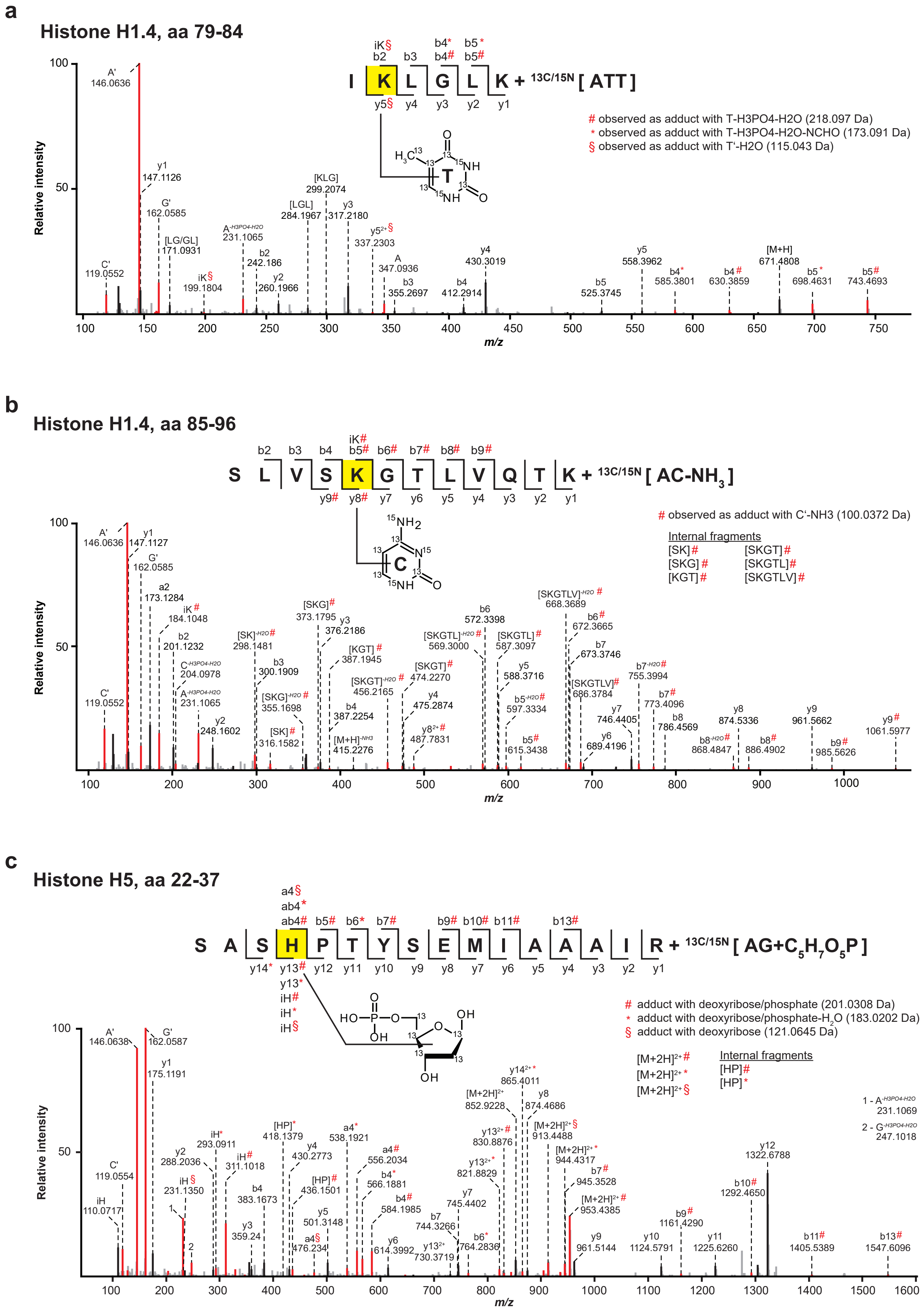

Suppl. Fig. 2: MS/MS spectra of linker histones H1.4 and H5 cross-linked to fully labeled 13C/15N-DNA (187 bp). In each spectrum, the identified peptide sequence is shown and the cross-linked amino acid is highlighted in yellow. Black: fragment ions; Red: shifted ion fragments and marker ions. a) Cross-link of H1.4 Lys-80 to ATT trinucleotide and shifted ion series revealing thymine as the cross- linked DNA moiety. b) Cross-link of H1.4 Lys-89 to AC-NH3 dinucleotide and shifted ion series revealing cytosine as the cross- linked DNA moiety. c) Cross-link of H5 His-25 to CCCC-H2O tetranucleotide and shifted ion series revealing deoxyribose as the cross- linked DNA moiety.

X. laevis mononucleosome and nucleosomal array CSM count

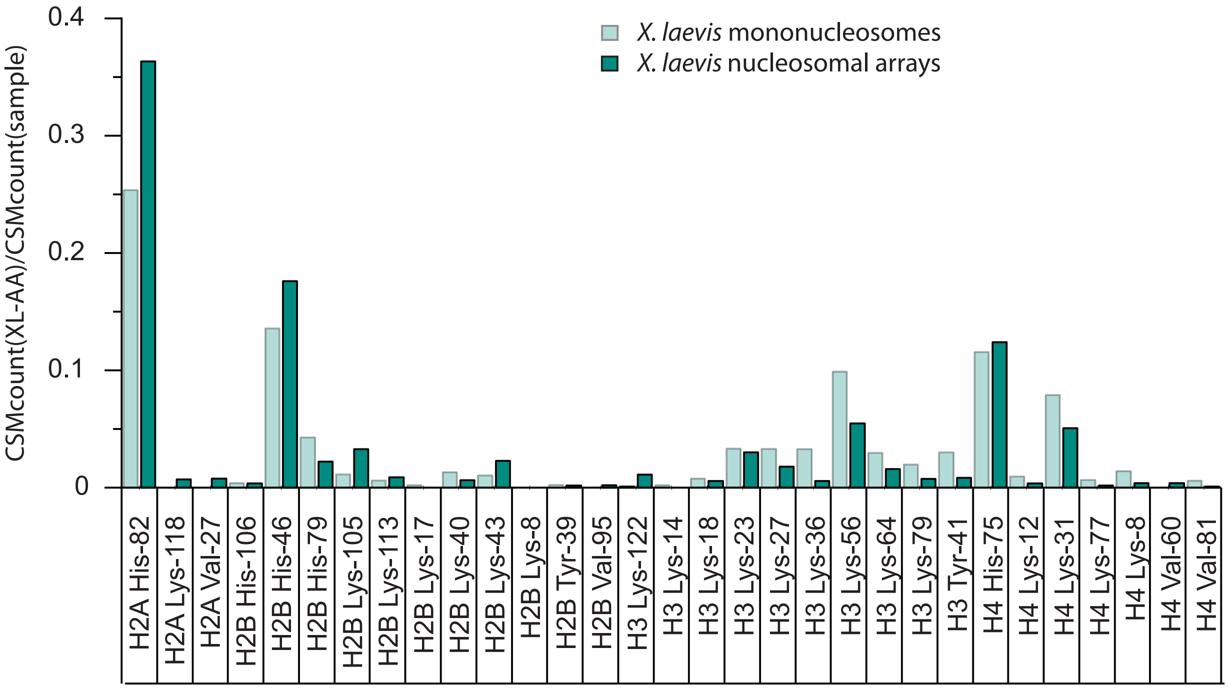

Suppl. Fig. 3**:** Bar plot of cross-link spectrum match (CSM) count for individual cross-link sites normalized on total CSM count in *in vitro* reconstituted Xenopus laevis mononucleosomes and nucleosomal arrays.

a

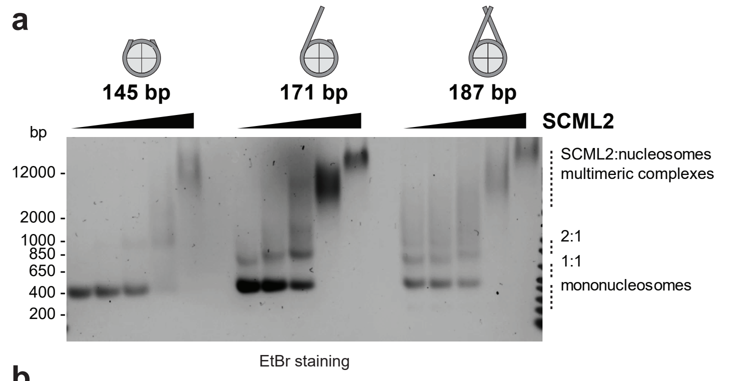

b

SCML2 peptide	(aa)	cross-link site	cross-link DNA
DDFHWEEYLK	(31-40)	His-34	deoxyribose
ETGSISAPSECFR	(41-53)	Cys-51	deoxyribose
LVDSPDIQPVGTCEK	(109-123)	Cys-121	deoxyribose
NPYLICPATIGDVK	(185-198)	Cys-190	deoxyribose
DIFPAGWCR	(223-231)	Cys-230	deoxyribose
EKPLPVICSTSAASLK	(317-322)	Cys-324	deoxyribose
HGNFGPHLDPK	(364-374)	His-364/His-369	deoxyribose
IQQLPDHFGPGPVNVVLR	(376-393)	His-382	deoxyribose
TVFGYLKPDNR	(408-418)	Lys-414	thymine
TKEYASEGEPLFAGGSAIPK	(517-536)	Tyr-520	thymine
(R)SPQQTVPYVVPLSPK	(498/499-513)	Pro-505/Tyr-506	thymine
SSSLNSGNYLNPACR	(546-560)	Cys-559	deoxyribose
(K)SEAPSYIAVPDPSVLK	(605/606-621)	Tyr-611	thymine
HTDPQISGPLADLFR	(642-656)	His-642	deoxyribose
LCYYIEK	(687-693)	Cys-688	deoxyribose

c

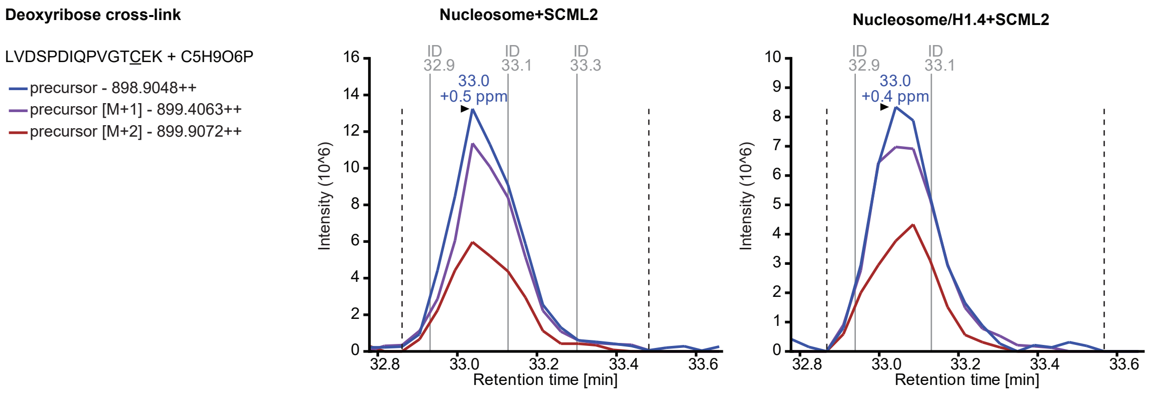

d

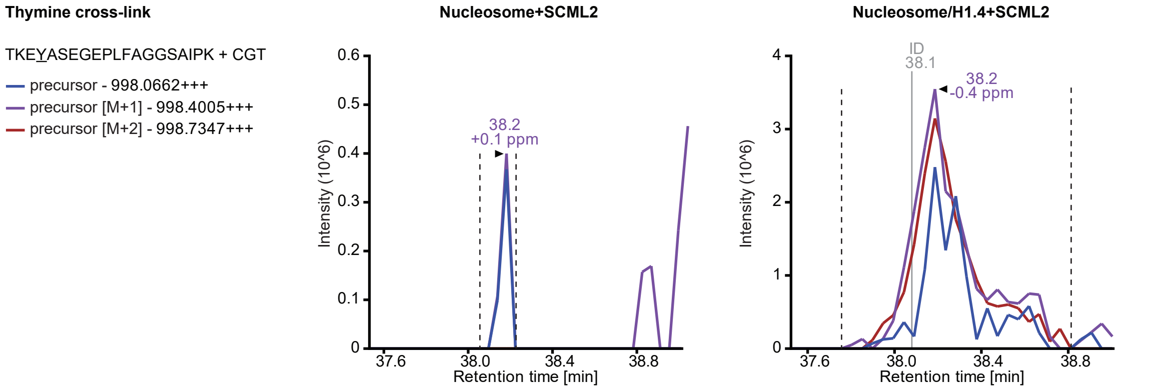

e

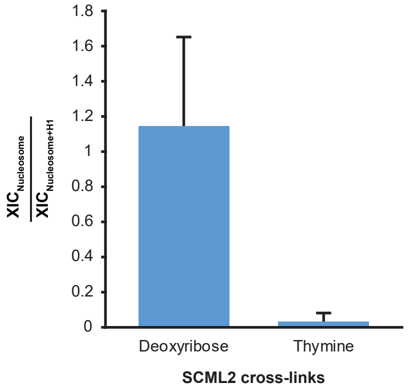

f

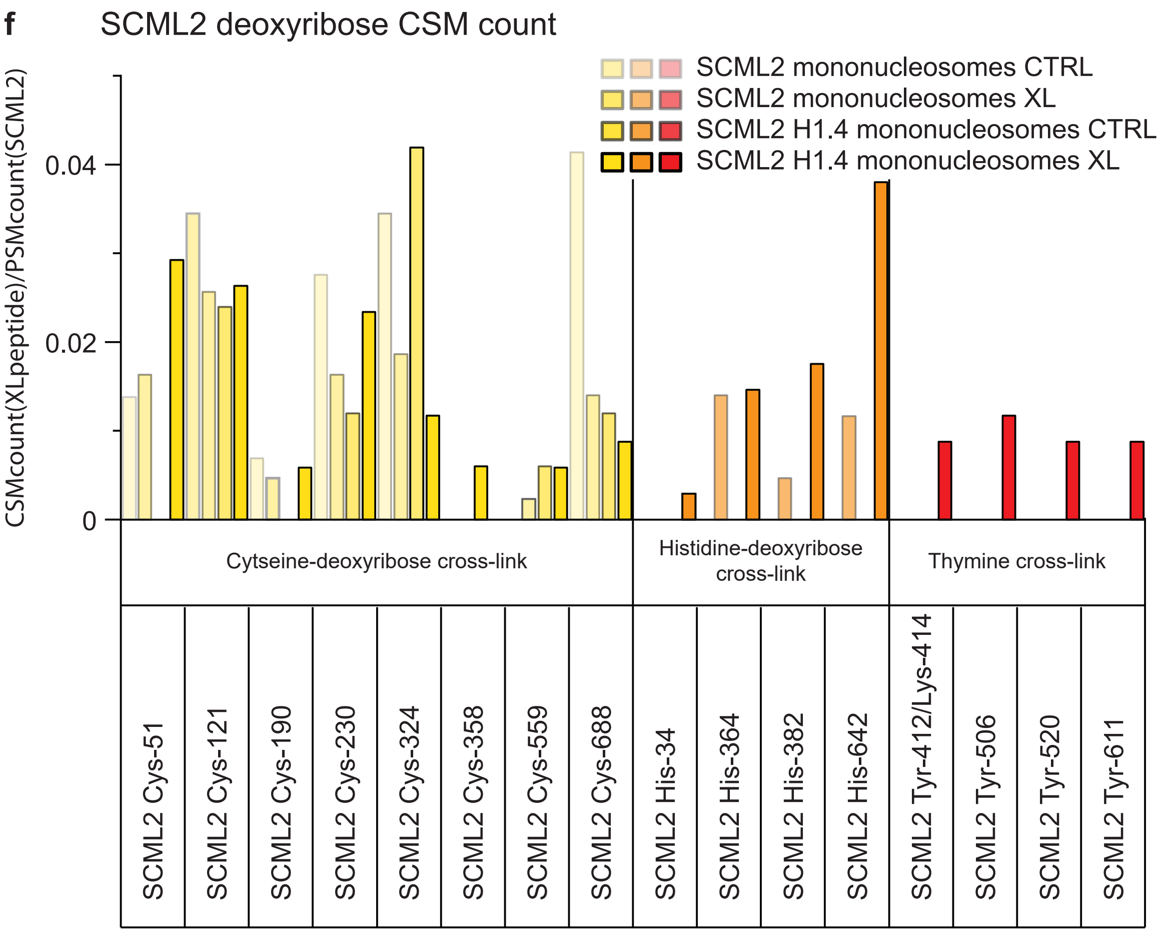

g

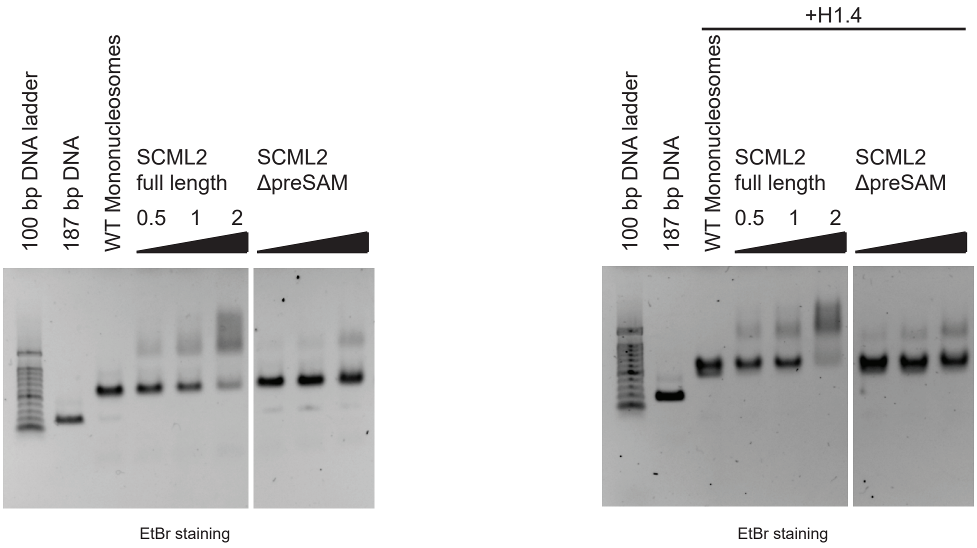

Suppl. Fig. 4: a) Gel-shift assay of SCML2 and nucleosomes of different DNA length (145 bp, 171 bp, 187 bp). SCML2 was incubated with nucleosomes and reactions were separated by agarose gel electrophoresis and visualized by staining with ethidium bromide. b) Table summarizing all cross-linked SCML2 peptides, their sequence, length, cross-linked amino acid and the cross-linked DNA moiety. c) Example of a SCML2 peptide cross-link to deoxyribose and its quantification of signal intensities (XIC) in both samples, nucleosome+SCML2 and nucleosome/H1.4+SCML2, by Skyline software. Monoisotopic, [M+1] and [M+2] isotope peaks of identified precursor are indicated by a color code. Retention time (min) and precursor m/z error in ppm are shown. Dashed lines represent peak boundaries used for signal integration. In grey retention times of identified cross-link spectral matches are marked. d) Example of a SCML2 peptide cross-link to thymine and its quanti-fication of signal intensities (XIC) as explained in c). e) Bar diagram of average XIC ratios of XICnucleosome:XICnucleosome+H1.4 of SCML2 cross-links to deoxyribose and thymine. Error bars represent standard error of the mean. f) Bar plot of cross-link spectrum match (CSM) count for individual cross-link sites normalized on total SCML2 peptide spectrum match (PSM) count in cross-link and non-cross-linked samples. Cross-link sites are categorized by cysteine- or histidine-deoxyribose phosphate and thymine-base cross-links. g) Gel-shift assay of WT mononucleosomes in complex with increasing amounts of full-length SCML2 and ΔpreSAM SCML2 mutant in the absence (left panel) and presence (right panel) of H1.4. 187 bp DNA and WT mononucleosomes were loaded as controls. The agarose-gel was stained with ethidium bromide.

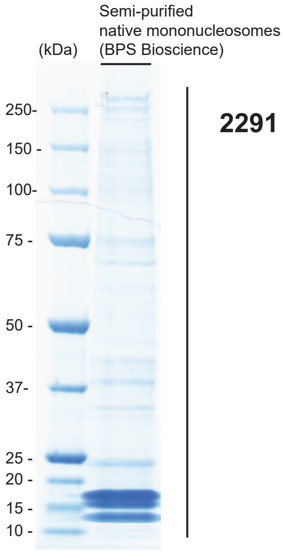

2291 idenified proteins:

1512 nuclear proteins (Uniprot; Subcellular location)

core histones H2A, H2B, H3, H4
5 linker histones

400 DNA binding proteins (Uniprot; GO molecular function)
659 RNA binding proteins (Uniprot; GO molecular function)
160 DNA/RNA binding proteins (Uniprot; GO molecular function)

Suppl. Fig. 5: SDS-PAGE of native mononucleosomes purified from human HeLa cells. On the right: Proteins identified by ESI-MS/MS and MaxQuant analysis of the depicted gel-lane.

Supplementary Note 1

Evaluation of MS/MS spectra of peptides cross-linked to DNA in TOPPView

Data analysis and spectra validation was performed as described in ([1]) with the RNPxl tool ([2]) in the OpenMS software network (https://www.openms.de/). Briefly, .raw files were converted to the .mzml format, centroided and spectra matching to linear peptides were removed. RNPxl setting for the various searches can be retrieved if .idxml files are opened as text files.

.raw, .mzML and idXML files have been uploaded to the ProteomeXchange Consortium via the PRI-DE partner repository with the dataset identifier PXD020290.

The following files are deposited in PRIDE database:

1. mzML files:
    a. *in vitro*
        i. C_Kappert_231015_2_H5_187_20HCD.mzML
        ii. C_Kappert_231015_3_H5_187_25HCD.mzML
        iii. C_Kappert_231015_4_H5_187_30HCD.mzML
        iv. C_Kappert_131015_H5_187labeled.mzML
        v. A_Stuetzer_251115_187_H14_20HCD.mzML
        vi. A_Stuetzer_251115_187_H14_25HCD.mzML
        vii. A_Stuetzer_251115_187_H14_30HCD.mzML
        viii. A_Stuetzer_080316_H14_lableledDNA.mzML
        ix. A_Stuetzer_010218_020218_187nuc_SCML2.mzML
        x. A_Stuetzer_010218_020218_187nuc_SCML2_Ctr.mzML
        xi. A_Stuetzer_010218_020218_187-H14_SCML2.mzML
        xii. A_Stuetzer_010218_020218_187-H14_SCML2_Ctr.mzML
        xiii. A_Stuetzer_200318_210318_monos_UV1.mzML
        xiv. A_Stuetzer_200318_210318_monos_UV2.mzML
        xv. A_Stuetzer_180816_12mer_25mM.mzML
        xvi. A_Stuetzer_180816_12mer_150mM.mzML
        xvii. A_Stuetzer_180816_12mer_H14_25mM.mzML
        xviii. A_Stuetzer_180816_12mer_H14_150mM.mzML
    b. *ex vivo*
        i. LWelp_MRaabe_130320_150320_PurchasedMononuc_brpfraction1.mzML
        ii. LWelp_MRaabe_130320_150320_PurchasedMononuc_brpfraction2.mzML
        iii. LWelp_MRaabe_130320_150320_PurchasedMononuc_brpfraction3.mzML
        iv. LWelp_MRaabe_130320_150320_PurchasedMononuc_brpfraction4.mzML
        v. LWelp_MRaabe_130320_150320_PurchasedMononuc_brpfraction5.mzML
        vi. LWelp_MRaabe_130320_150320_PurchasedMononuc_brpfraction6.mzML
        vii. LWelp_MRaabe_130320_150320_PurchasedMononuc_brpfraction7.mzML
        viii. LWelp_MRaabe_130320_150320_PurchasedMononuc_brpfraction8.mzML
        ix. LWelp_MRaabe_130320_150320_PurchasedMononuc_brpfraction9.mzML
        x. LWelp_MRaabe_130320_150320_PurchasedMononuc_brpfraction10.mzML
        xi. LWelp_MRaabe_130320_150320_PurchasedMononuc_brpfraction11.mzML
        xii. LWelp_MRaabe_130320_150320_PurchasedMononuc_brpfraction12.mzML
        xiii. LWelp_MRaabe_130320_150320_PurchasedMononuc_brpFT.mzML
        xiv. LWelp_MRaabe_130320_150320_PurchasedMononuc_input.mzML
    c. *in nucleo*
        i. AStuetzer_100118_120118_HeLaUV_rep1_sample1.mzML
        ii. AStuetzer_100118_120118_HeLaUV_rep1_sample2.mzML
        iii. AStuetzer_100118_120118_HeLaUV_rep2_sample1.mzML
        iv. AStuetzer_100118_120118_HeLaUV_rep2_sample2.mzML
        v. LWelp_MRaabe_190420_270420_HeLanuclei_UVXL_pSEC.mzML

2. idXML files:
    a. *in vitro*
        i. C_Kappert_231015_2_H5_187_20HCD_DNAsearch.idXML
        ii. C_Kappert_231015_3_H5_187_25HCD_DNAsearch.idXML
        iii. C_Kappert_231015_4_H5_187_30HCD_DNAsearch.idXML
        iv. C_Kappert_131015_H5_187labeled_DNAsearch.idXML
        v. A_Stuetzer_251115_187_H14_20HCD_DNAsearch.idXML
        vi. A_Stuetzer_251115_187_H14_25HCD_DNAsearch.idXML
        vii. A_Stuetzer_251115_187_H14_30HCD_DNAsearch.idXML

viii. A_Stuetzer_080316_H14_lableledDNA_DNAsearch.idXML
ix. A_Stuetzer_010218_020218_187nuc_SCML2_DNAsearch.idXML
x. A_Stuetzer_010218_020218_187nuc_SCML2_Ctr_DNAsearch.idxml
xi. A_Stuetzer_010218_020218_187-H14_SCML2_DNAsearch.idXML
xii. A_Stuetzer_010218_020218_187-H14_SCML2_Ctr_DNAsearch.idxml
xiii. A_Stuetzer_200318_210318_monos_UV1_DNAsearch.idXML
xiv. A_Stuetzer_200318_210318_monos_UV2_DNAsearch.idXML
xv. A_Stuetzer_180816_12mer_25mM_DNAsearch.idXML
xvi. A_Stuetzer_180816_12mer_150mM_DNAsearch.idXML
xvii. A_Stuetzer_180816_12mer_H14_25mM_DNAsearch.idXML
xviii. A_Stuetzer_180816_12mer_H14_150mM_DNAsearch.idXML

b. *ex vivo*

i. LWelp_MRaabe_130320_150320_PurchasedMononuc_brpfraction1_DNAsearch.idXML
ii. LWelp_MRaabe_130320_150320_PurchasedMononuc_brpfraction2_DNAsearch.idXML
iii. LWelp_MRaabe_130320_150320_PurchasedMononuc_brpfraction3_DNAsearch.idXML
iv. LWelp_MRaabe_130320_150320_PurchasedMononuc_brpfraction4_DNAsearch.idXML
v. LWelp_MRaabe_130320_150320_PurchasedMononuc_brpfraction5_DNAsearch.idXML
vi. LWelp_MRaabe_130320_150320_PurchasedMononuc_brpfraction6_DNAsearch.idXML
vii. LWelp_MRaabe_130320_150320_PurchasedMononuc_brpfraction7_DNAsearch.idXML
viii. LWelp_MRaabe_130320_150320_PurchasedMononuc_brpfraction8_DNAsearch.idXML
ix. LWelp_MRaabe_130320_150320_PurchasedMononuc_brpfraction9_DNAsearch.idXML
x. LWelp_MRaabe_130320_150320_PurchasedMononuc_brpfraction10_DNAsearch.idXML
xi. LWelp_MRaabe_130320_150320_PurchasedMononuc_brpfraction11_DNAsearch.idXML
xii. LWelp_MRaabe_130320_150320_PurchasedMononuc_brpfraction12_DNAsearch.idXML
xiii. LWelp_MRaabe_130320_150320_PurchasedMononuc_brpFT_DNAsearch.idXML
xiv. LWelp_MRaabe_130320_150320_PurchasedMononuc_input_DNAsearch.idXML

c. *in nucleo*

i. AStuetzer_100118_120118_HeLaUV_rep1_sample1_DNAsearch.idXML
ii. AStuetzer_100118_120118_HeLaUV_rep1_sample2_DNAsearch.idXML
iii. AStuetzer_100118_120118_HeLaUV_rep2_sample1_DNAsearch.idXML
iv. AStuetzer_100118_120118_HeLaUV_rep2_sample2_DNAsearch.idXML
v. AStuetzer_100118_120118_HeLaUV_rep1_sample1_DNAsearch_RNPxlsettings2.idxml
vi. AStuetzer_100118_120118_HeLaUV_rep1_sample2_DNAsearch_RNPxlsettings2.idXML
vii. AStuetzer_100118_120118_HeLaUV_rep2_sample1_DNAsearch_RNPxlsettings2.idXML
viii. AStuetzer_100118_120118_HeLaUV_rep2_sample2_DNAsearch_RNPxlsettings2.idXML
ix. AStuetzer_100118_120118_HeLaUV_rep1_sample1_RNAsearch.idXML
x. AStuetzer_100118_120118_HeLaUV_rep1_sample2_RNAsearch.idXML
xi. AStuetzer_100118_120118_HeLaUV_rep2_sample1_RNAsearch.idXML
xii. AStuetzer_100118_120118_HeLaUV_rep2_sample2_RNAsearch.idXML
xiii. LWelp_MRaabe_190420_270420_HeLanuclei_UVXL_pSEC_DNAsearch.idXML

Evaluation of cross-linked peptide-DNA oligonucleotides MS2 spectra in ToppView (Version 2.5.0, [3])

1. Install OpenMS (Version 2.5.0) on your computer (http://www.openms.de) start TOPPView by double-clicking on the chosen .mzML. Of note, loading of the .mzML file into ToppView is accelerated if the corresponding .mzML is available on your local computer/hard drive.

2. ToppView will open the .mzML file (Suppl. Fig. 1).

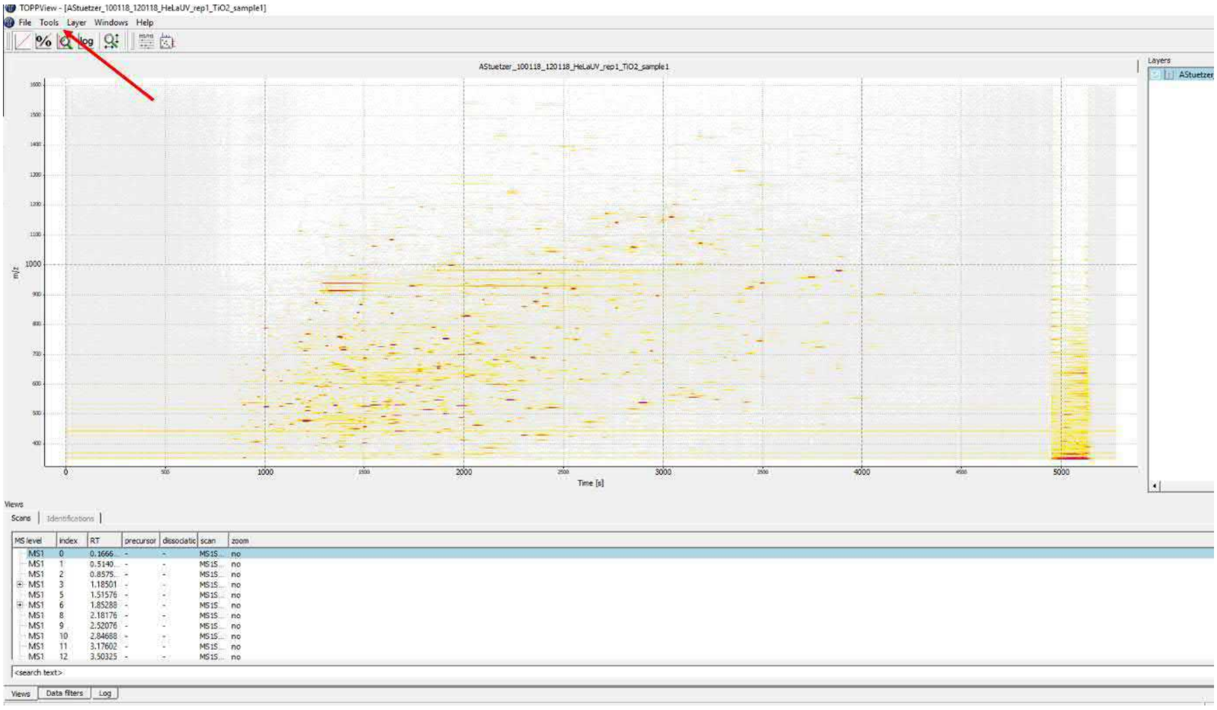

Suppl. Fig. 6: Screenshot of TOPPView showing m/z values over LC-MS retention time of the .mzML file AStuetzer_100118_120118_HeLaUV_rep1_TiO2_sample1.mzML (Pride PXD020290).

3. Chose "Tools" and click on "Annotate with identifications" (Suppl. Fig. 6).
4. Chose the corresponding .idXML file (Suppl. Fig. 7). Ensure that you chose the .idXML output from RNPxl corresponds to the loaded .mzML file.

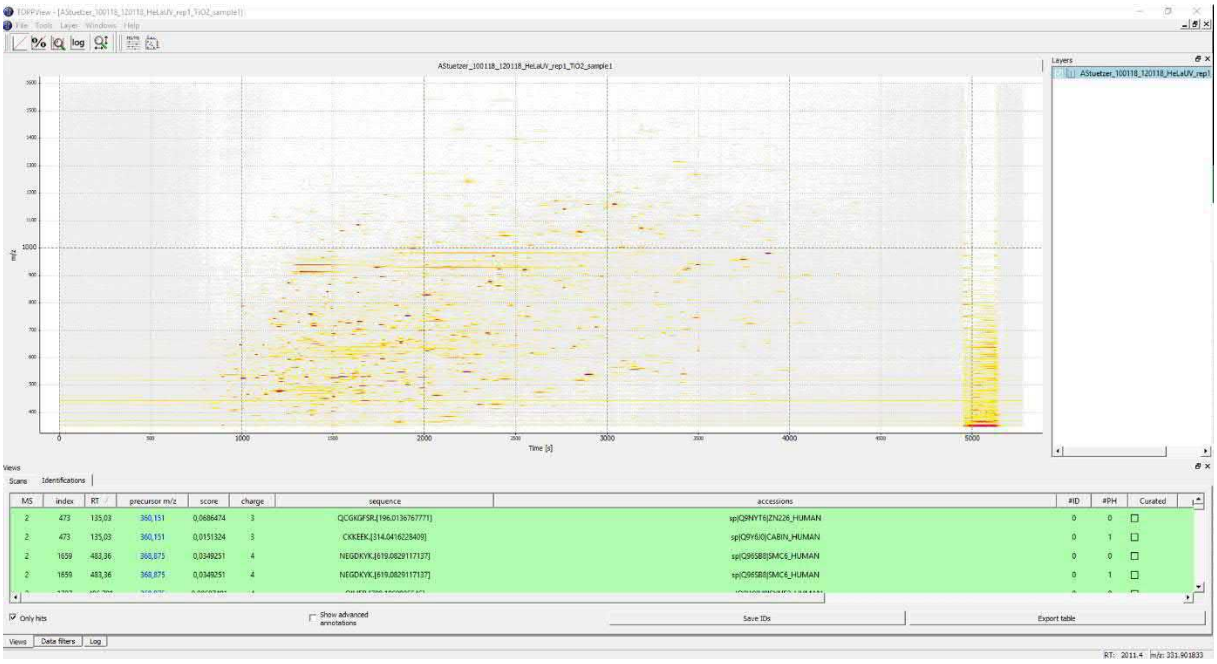

Suppl. Fig. 7: Screenshot of TOPPView showing m/z values over LC-MS retention time. AStuetzer_100118_120118_HeLaUV_rep1_TiO2_sample1.mzML with RNPxl annotated file loaded (AStuetzer_100118_120118_HeLaUV_rep1_TiO2_sample1_DNAsearch_RNPxlsettings2.idxml (Pride PXD020290).

5. Click on "Show advanced annotation" (Suppl. Fig. 8, red arrow).

6. Click on "Scans" (Suppl. Fig. 8, red arrow 2).

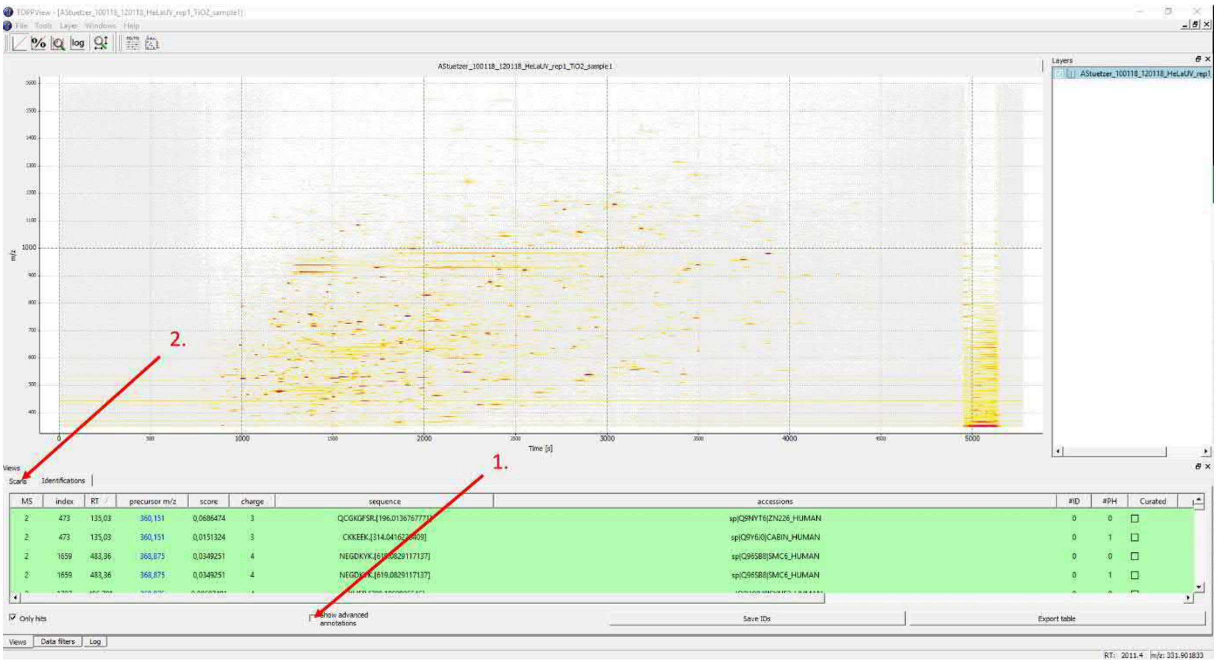

Suppl. Fig. 8: Screenshot of TOPPView showing m/z values over LC-MS retention time. AStuetzer_100118_120118_HeLaUV_rep1_TiO2_sample1.mzML with RNPxl annotated file loaded (AStuetzer_100118_120118_HeLaUV_rep1_TiO2_sample1_DNAsearch_RNPxlsettings2.idxml (Pride PXD020290). Red arrows indicate points 5 and 6 mentioned above.

The screen will change as shown in Supplementary Figure 9.

7. Click on "Identifications" (red arrow Suppl. Fig. 9)

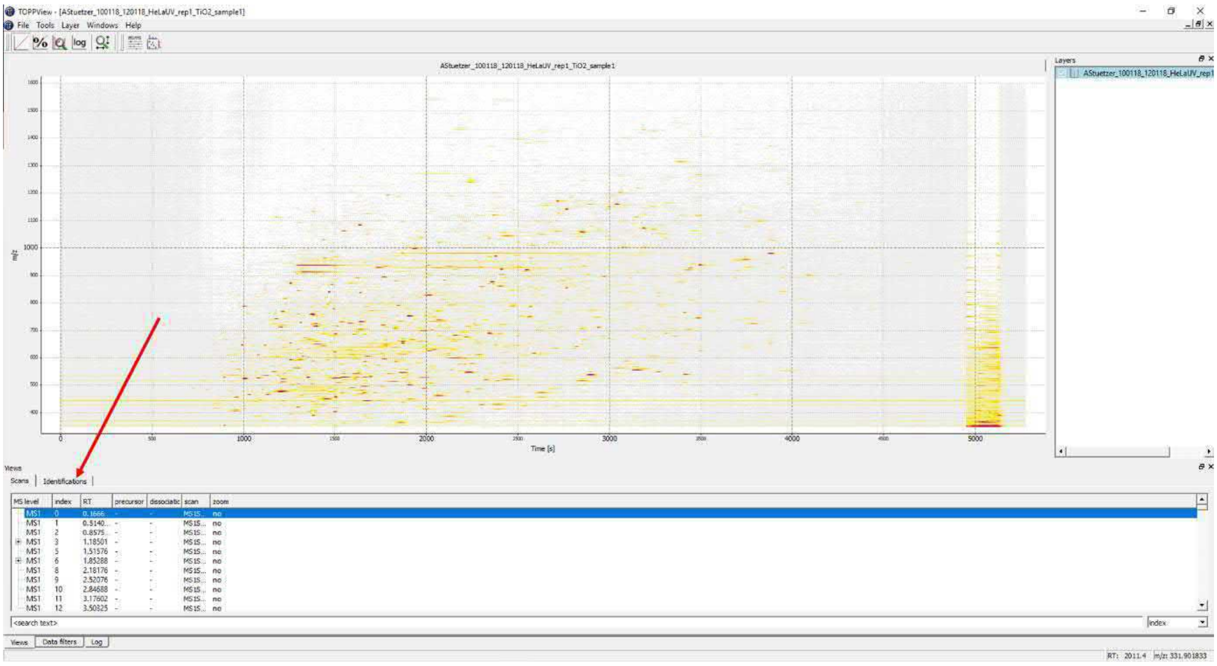

Suppl. Fig. 9: Screenshot of TOPPView after having clicked on "Scans" showing m/z values over LC-MS retention time. AStuetzer_100118_120118_HeLaUV_rep1_TiO2_sample1.mzML with AStuetzer_100118_120118_HeLaUV_rep1_TiO2_sample1_DNA search_RNPxlsettings2.idxml loaded (Pride PXD020290).

The screen will change as shown in Supplementary Figure 10.

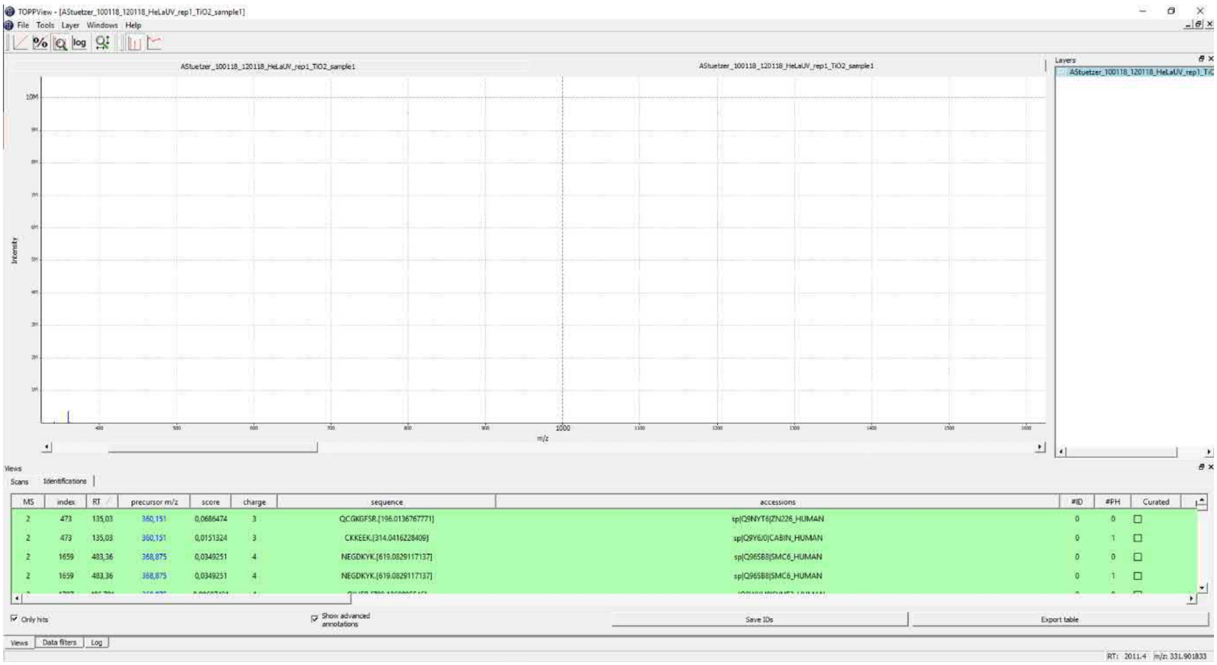

Suppl. Fig. 10: Screenshot of TOPPView showing intensities over m/z. No MS2 spectrum of a cross-linked peptide yet shown of RNPxl annotated file loaded (AStuetzer_100118_120118_HeLaUV_rep1_TiO2_sample1_DNA search_RNPxlsettings2.idxml (Pride PXD020290)) after having clicked on "Identifications" (Suppl. Fig. 9). In the green area, sequenced peptide-DNA cross-links are listed.

In the green report grid, sequenced peptide-DNA cross-links are listed. The columns in the can be moved and adjusted to the user’s needs (see below).

8. For evaluation, several settings can be adjusted by right-clicking into the spectrum field and selecting the respective options like "show/hide grid lines", etc. (Suppl. Fig. 11). For optimal visualization of cross-link spectra, "Style: Stick <--> Area" should be changed to stick-representation of signals. For manual evaluation we set options to "Hide grid lines", "Stick", and "Percent".

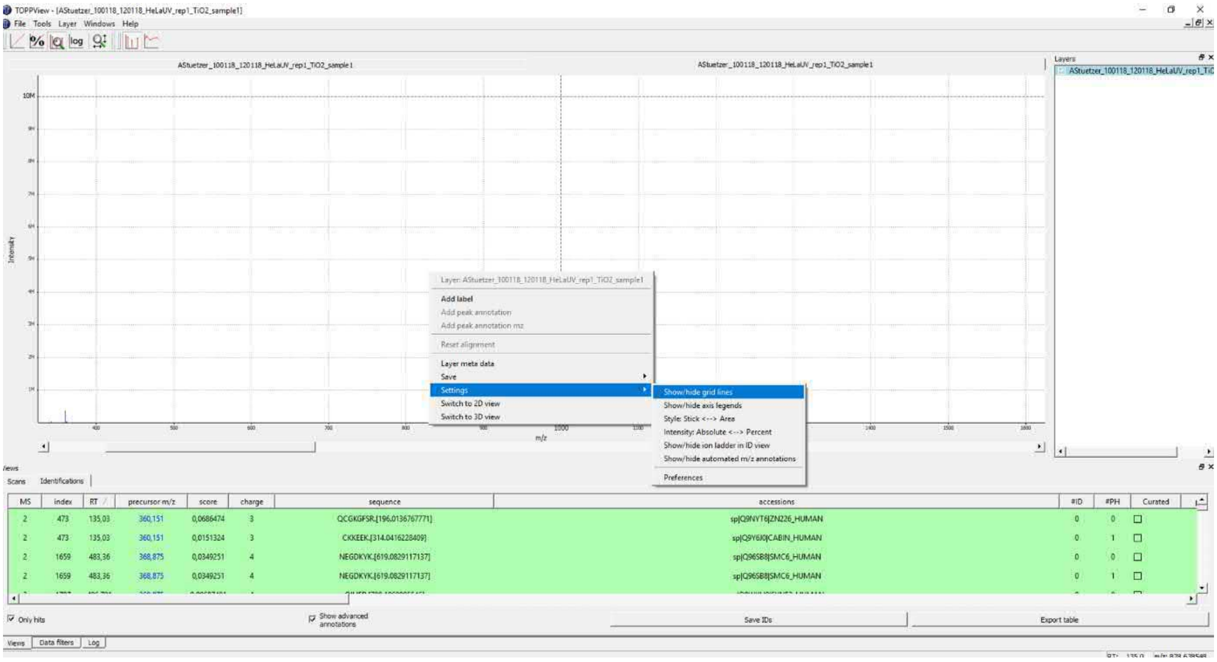

Suppl. Fig. 11: Screenshot of TOPPView showing intensities over m/z. No MS2 spectrum of a cross-linked peptide shown, yet. RNPxl annotated file loaded (AStuetzer_100118_120118_HeLaUV_rep1_TiO2_sample1_DNA search_RNPxlsettings2.idxml (Pride PXD020290) with "Settings" clicked showing the available options.

9. Sort the list of sequenced cross-links according to their index numbers starting with the smallest number (Suppl. Fig. 12; index 473).
The TOPPView spectrum will appear as in Supplementary Figure 12.

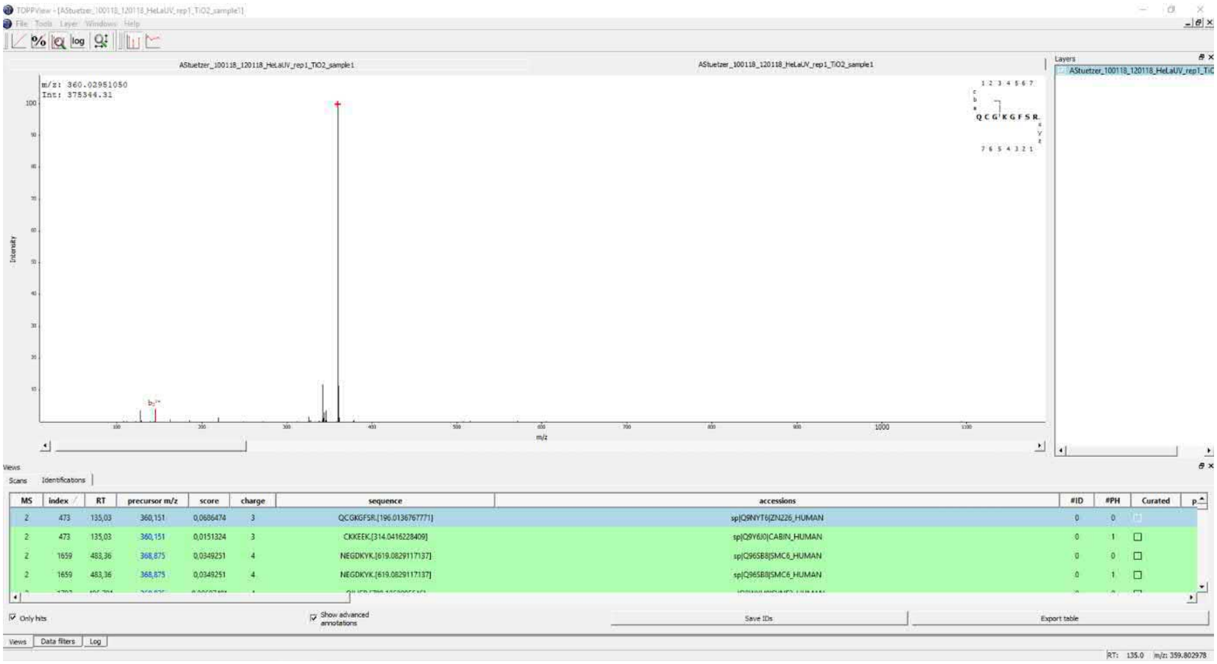

Suppl. Fig. 12: Screenshot of TOPPView showing fragment ion intensities with m/z values of the putatively cross-linked peptide QCGKGFSR after having chosen the above mentioned settings, sorting the list according to index numbers (smallest to largest) and choosing the sequence (QCGKGFSR) of index 473. Annotated b-type ions are in red, y-type ions are in green (here not present). The sequence with the corresponding b- and y-type ions indicated as lines is shown in the upper right corner of the annotated MS2 spectrum.
We note that sometimes the precursor (here m/z 360, 151 [Th]) is not annotated automatically. The cursor (red cross) depicts the apex of the precursor peak and its corresponding m/z, though not automatically annotated, is shown in the upper left corner of the MS2 spectrum.

10. For manual evaluation of all annotated spectra adjust the values in the list according to the your needs like exemplary shown in Supplementary Figure 13.

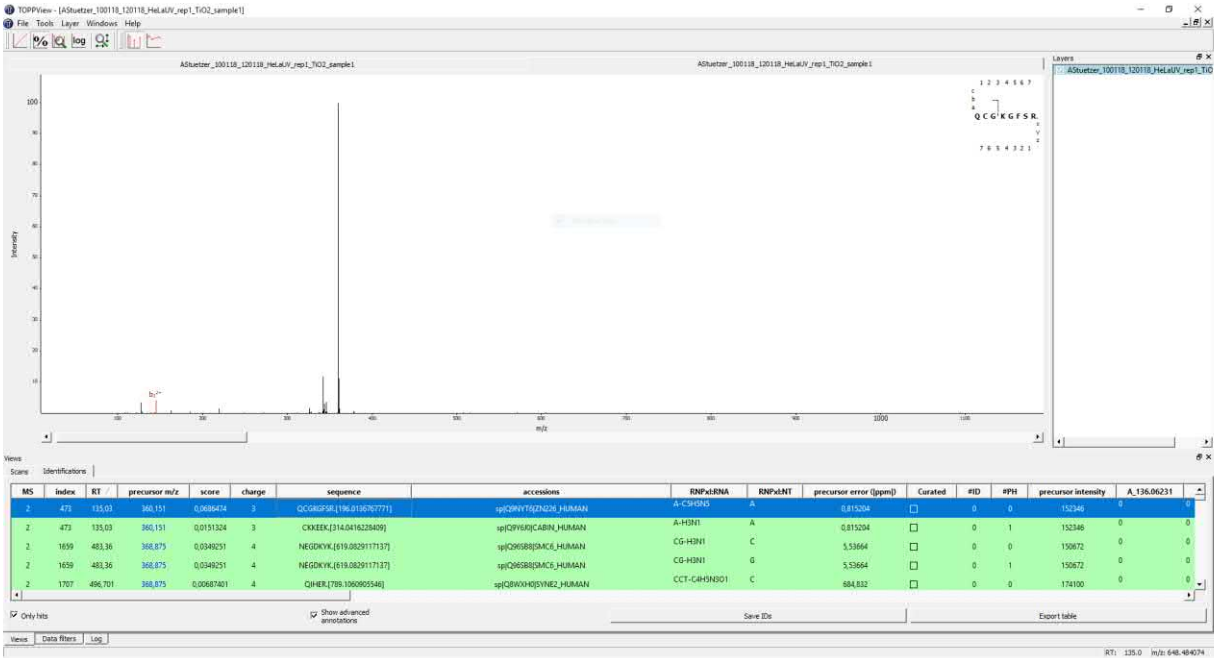

Suppl. Fig. 13: Screenshot of TOPPView showing fragment ion intensities with m/z values of the putatively cross-linked peptide QCGKGFSR after having chosen the above listed settings, sorting the list according to index numbers (smallest to largest) and choosing the sequence (QCGKGFSR) of index 473. The following values are listed, which are recommended for manual evaluation: MS, index, retention time (RT), precursor *m/z*, score, charge, sequence (with the adduct mass of putatively cross-linked DNA moiety in brackets), accessions (here Uniprot database), RNPxl:RNA (representing the cross-linked DNA adduct; note that despite DNA search was performed, still :RNA is stated),

RNPxk:NT (cross-linked nucleotide), precursor error and curated. The boxes in the "curated" column can be checked, facilitating manual validation. Resuls can be exported as .tsv file by clicking "Export table" in the lower right corner of the result grid. Of note, the information from "curated" column not automatically saved, i.e. when closing and opening TOPPView with the respective .mzML and .idXML files, the "curated" boxes are no longer checked.

11. The retention time at which (cross-linked) peptides eluted can be seen in the TOPPView window shown in Suppl. Fig. 11 (m/z over Retention time of sample). We suggest to sort results by index, retention time, peptide sequence or protein accession following successive spectra validation for comprehensive manual evaluation.

Supplementary Figure 14 shows the first mannually approved hit, a peptide derived from protein PHF 19 that meet manual evaluation criteria with sufficient b- (red) and y (green)-type ions assigned with DNA mass adducts.

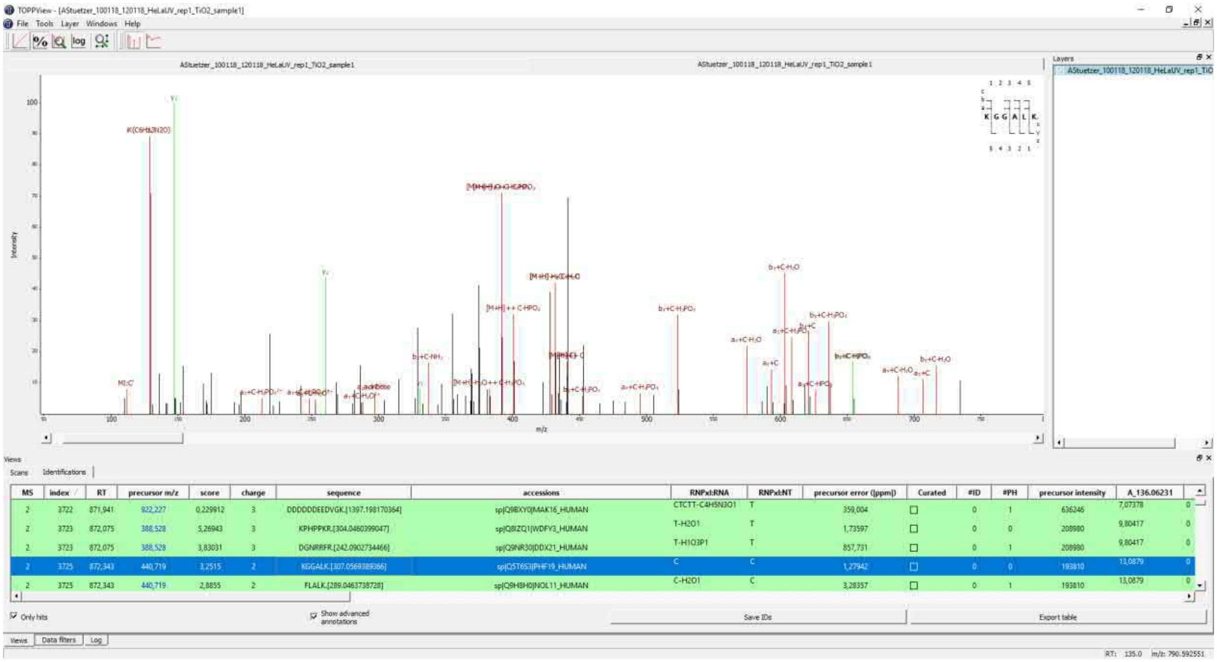

Suppl. Fig. 14: Screenshot of TOPPView with RNPxl annotated MS2 spectrum of the peptide KGGALK cross-linked to cytidine-monophosphate. Of note, the cytosine base, which is cleaved off during MS/MS fragmentation is detected in the lower m/z range.

The same precursor mass matches to the proceeding MS2 spectra in the list (Suppl. Fig. 15) but with less fragment ions annotated.

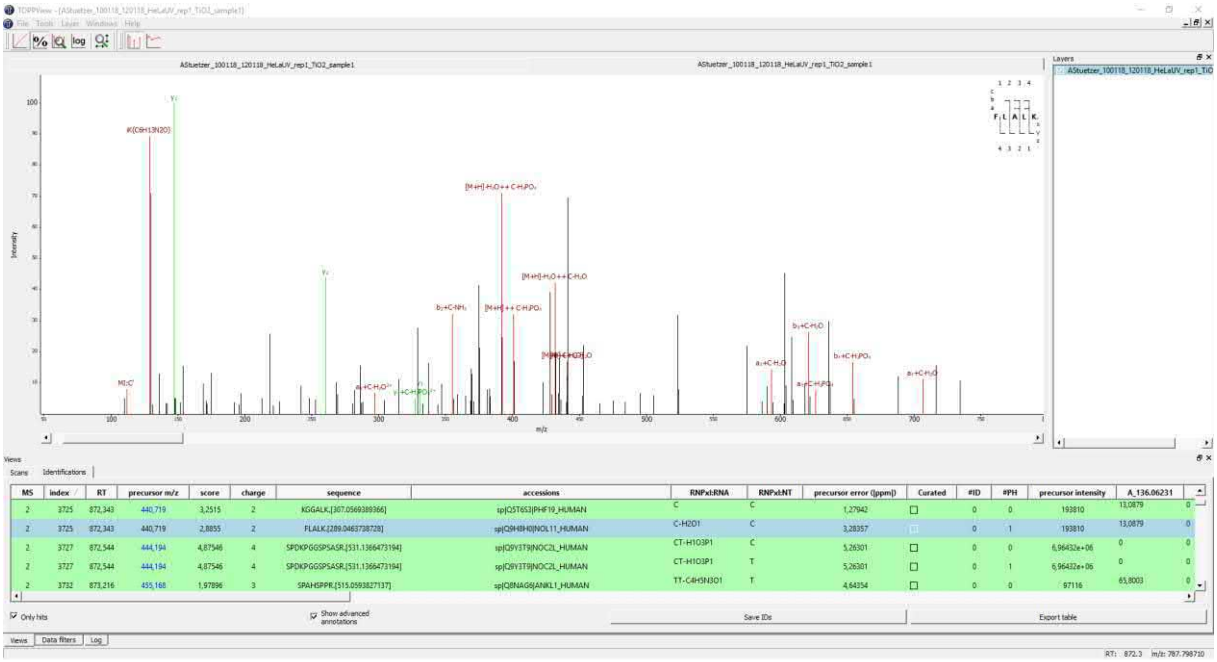

Suppl. Figure 15: Screenshot of TOPPView with RNPxl annotated MS2 spectrum of the peptide FLALK cross-linked to cytidine-monophosphate-$H_2$0. Of note, the cytosine base, which is cleaved off during MS/MS is detected in the lower m/z range.

Due to a larger number of annotated fragment ions, the peptide derived from PHF19 is considered as a true cross-link spectrum and is included in Suppl. Data 2, 4, 5, 6, 7 and 8. (In Suppl. Data 8 the TOPPView spectrum of the same cross-link is shown but by another MS2 spectrum with index no. 3827.) For presentation of the annotated MS spectra in Suppl. Data 5, 6, 7 and 8, the TOPPView presentation is rearranged as shown in Suppl. Fig. 16.

In this manner, all MS2 spectra annotated by RNPxl were manually validated for our study and, if approved listed/shown in Supplementary Data 2, 4, 5, 6, 7 and 8.

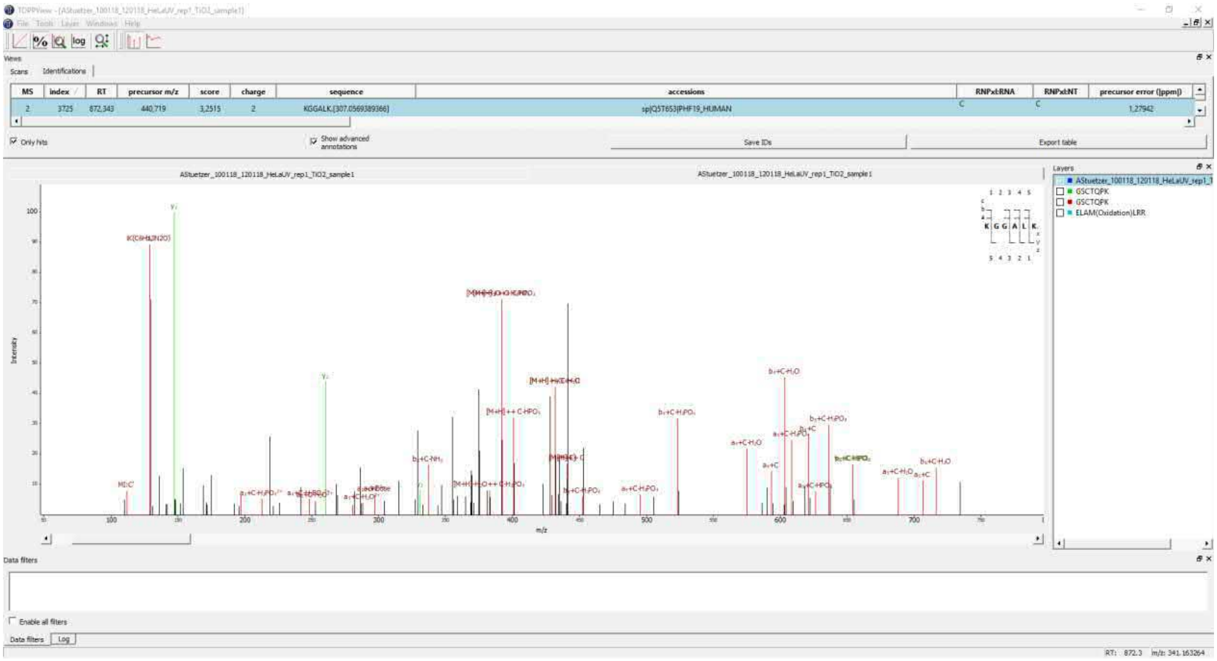

Suppl. Fig. 16: Screenshot of annotated MS2 spectrum of the peptide KGGALK cross-linked to cytidine-monophosphate with rearranged TOPPView presentation as in Supplementary Data 5, 6, 7 and 8.

Suppl. Fig. 17 shows the screenshot of an RNPxl annotated MS2 spectrum of a peptide derived from protein NOC2L_HUMAN cross-linked to a CT – HPO3. The triply charged precursor is assigned and cytosine base marker ion as well as y-type fragment ions harboring C-HPO3 adducts are present indicating that this spectrum might represent a cross-link. Notably, the MS2 spectrum includes a peak which is the precursor minus phosphate (assigned manually as ~ 33 [Th]). The occurrence of such a neutral loss on a precursor that theoretically (as deduced from RNP^{xl} annotation) cannot lose phosphate is indicative for this spectrum to be a false positive hit. Currently, it can be assumed that it is very unlikely, that the peptide cross-linked to CT-HPO3 loses another phosphate when the dinucleotide CT is bridged by a phosphate bond. It might be that the two nucleotides are cross-linked independently as single nucleotides, i.e. one including a phosphate residue and the other one lost the phosphate residue. More likely, this precursor represents a phosphorylated peptide.

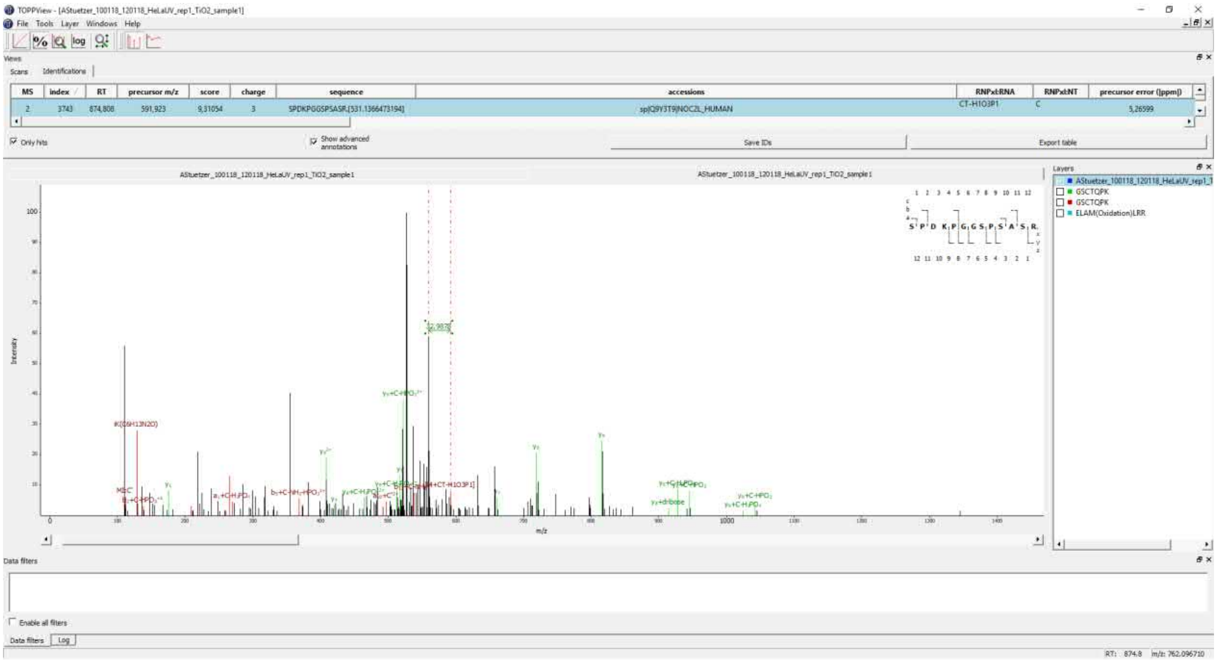

Suppl. Fig. 17: Screenshot of TOPPView with RNPxl annotated MS2 spectrum of a peptide derived from protein NOC2L_HUMAN cross-linked to a CT – HPO3, which is considered being a false positive identification (see text for details).

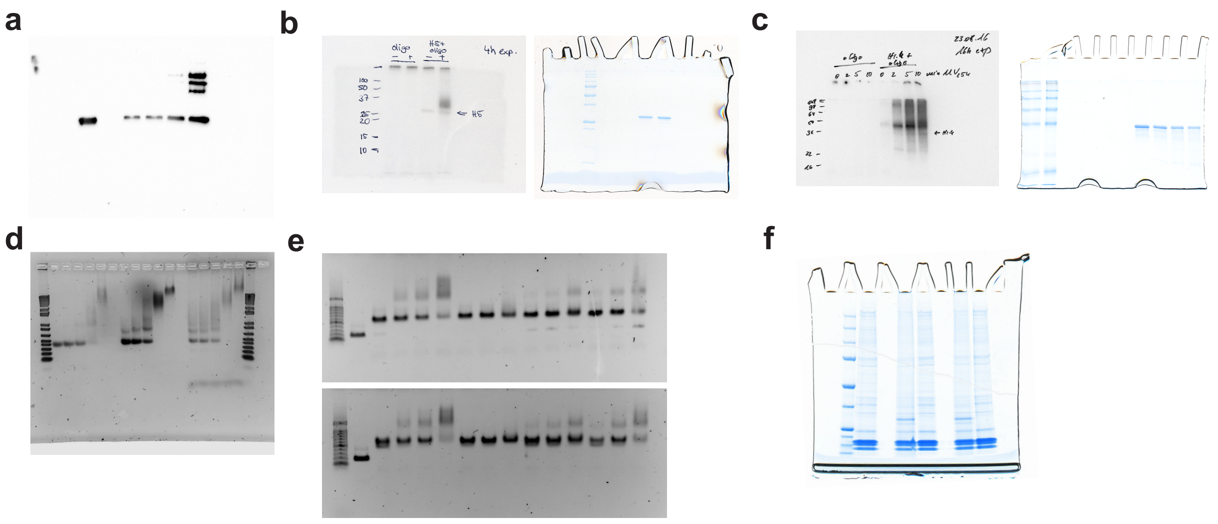

Suppl. Fig. 18: Unprocessed gel scans and autoradiographs. a) Western-blot displayed in Figure 1. b) Left panel: Autoradiograph displayed in Suppl. Fig. 1a. Right panel: Scan of coomassie-stained gel from Suppl. Fig. 1a. c) Left panel: Autoradiograph displayed in Suppl. Fig. 1b. Right panel: Scan of coomassie-stained gel from Suppl. Fig. 1b. d) Scan of ethidiumbromide-stained gel displayed in Suppl. Fig. 4a. e) Upper panel: Scan of ethidiumbromide-stained gel displayed in Suppl. Fig. 4g (left panel). Lower panel: Scan of ethidiumbromide-stained gel displayed in Suppl. Fig. 4g (right panel). f) Scan of coomassie-stained gel from Suppl. Fig. 5.

Supplementary Methods

Protein expression constructs

pET3a expression vectors containing *Xenopus laevis* core histone sequences (H3 (GenBank: CAD89679), H2A (GenBank: CAD89676), H2B (GenBank: CAD89678), H4 (GenBank: CAD89677) were a kind gift of Karolin Luger (Colorado State University, Fort Collins, USA)). Human linker histone H1.4 was cloned into pET3a using NdeI/BamHI sites including a C-terminal TEV-site and a His_6-tag. Full-length and mutant human SCML2 (Q9UQR0) were cloned into pETM-40 including a C-terminal TEV-site and a His_6-tag.

Protein expression and histone octamer assembly

X. laevis core histones were expressed and octamers were assembled as described before ([4],[5]). BL21 CodonPlus (DE3)-RIL (Stratagene, La Jolla, USA) cells were transfected with the histone expression constructs and proteins were overexpressed by IPTG induction. BL21 cells were disrupted with an EmulsiFlex-C5 homogenizer (>10,000 psi, AVESTIN) and inclusion bodies containing the histone proteins were collected by centrifugation. Histones were purified in a first step by gel filtration (Sephacryl S200 XK50/100 column, 90100175, Cytiva). Peak fractions were analyzed by SDS-PAGE and fractions containing histone proteins were further purified by ion-exchange chromatography. A XK26/20 Q-Sepharose column (28987027, Cytiva) was used connected in series to a XK26/20 SP-Sepharose (28987033, Cytiva) column for loading. For histone elution, the Q-Sepharose column was detached and histones were eluted from the SP-Sepharose column with a linear gradient from 200-600 mM NaCl. Purified core histones were lyophilized.

For octamer assembly, core histones (H2A, H2B, H3 and H4) were unfolded and mixed in equimolar ratios. To assemble the octamer, core histones were refolded by dialysis against high salt buffer. Octamers were purififed using a HiLoad 16/60 Superdex 200 (28989335, Cytiva) prep grade gelfiltration column.

Linker histone H1.4 was overexpressed in BL21 and purified via standard Ni^{2+}-NTA chromatography following His_6-tag removal by TEV protease digest followed by another Ni-NTA purification.

Human SCML2 (Q9UQR0) was overexpressed in BL21 and purified by Ni^{2+}-NTA chromatography. His-SCML2 and His-SCML2 delta preSAM were further purified on a HiTrap SP HP column. Purified proteins were dialyzed against a buffer containing 50 mM Tris-HCl pH 7.5, 300 mM NaCl and 10% glycerol.

Western blotting

Immunoblotting was performed using MiniTrans Blot system (1703930, Bio-Rad Laboratories) according to manufacturers instructions. Proteins were blotted to a nitrocellulose membrane for 1 h at 100 V, 4 °C. After washing and blocking (PBS and PBS/5% milkpowder) of the membrane, the primary

antibody (anti-H1, Active Motif 61201) was added in a 1:1000 dilution in PBS/5% milkpowder follwing incubation for 1 h at RT. The membrane was washed with PBS and incubated in the secondary antibody solution (anti-rabbit HRP, Dako P0399 in PBS/2.5 % milkpowder) for 1 hr at RT. After washing (three times PBS, 10 min each) the blot was developed with the Pierce™ ECL system (32209, Thermo Fisher Scientific). All Western blots performed for this study are displayed unprocessed in Supplementary Figure 18.

In-gel digestion for MS analysis

In-gel digestion of proteins for MS anaylsis was basically performed according to [6]. The entire SDS-PAGE lane was cut from the gel and into 23 slices. Acetonitrile was added to the gel pieces for 10 min until these started shrinking. The acetonitrile was removed and 10 mM dithiothreitol were added following incubation at 56 °C for 30 min. The supernatant was removed and proteins were alkylated by incubation with 55 mM iodoacetamide at RT for 20 min in the dark. All liquid was removed and replaced by acetonitrile. After shrinking, again the supernatant was removed and trypsin was added (1:20 enzyme-to-protein ratio) follwoing overnight digestion at 37 °C. Extraction buffer (1:2 (vol/vol) 5% formic acid/acetonitrile) was added following incubation for 15 min at 37 °C. The liquid was collected and dried in a speed-vac prior to MS analysis.

Supplementary References

1 Kramer, K. *et al.* Photo-cross-linking and high-resolution mass spectrometry for assignment of RNA-binding sites in RNA-binding proteins. *Nat. Methods* (2014). doi:10.1038/nmeth.3092

2 Angelov, D., Stefanovsky, VYu., Dimitrov, S. I., Russanova, V. R., Keskinova, E. & Pashev, I. G. Protein-DNA cross-linking in reconstituted nucleohistone, nuclei and whole cell by picosecond UV laser irradiation. *Nucleic Acids Res.* **16**, 4525–4538 (1988).

3 Sturm, M. & Kohlbacher, O., TOPPView: An open-source viewer for mass spectrometry data. *J. Proteome Res.* **8**, 3760–3763 (2009).

4 Luger, K., Rechsteiner, T. J. & Richmond, T. J. Preparation of nucleosome core particle from recombinant histones. *Methods Enzymol.* **304**, 3–19 (1999).

5 Stützer, A. *et al.* Modulations of DNA Contacts by Linker Histones and Post-translational Modifications Determine the Mobility and Modifiability of Nucleosomal H3 Tails. *Mol. Cell* **61**, 247–259 (2016)

6 Shevchenko, A., Thomas, H., Havlis, J., Olsen, J. V. & Mann, M. In-gel digestion for mass spectrometric characterization of proteins and proteomes. *Nat. Protocols*. **1**, 2856–2860 (2006).
