# Supplementary material for: Analysis of protein-DNA interactions in chromatin by UV induced cross-linking and mass spectrometry
Source: Nat Commun. 2020 Oct 16;11:5250. doi: 10.1038/s41467-020-19047-7 (PMC7567871; doi:10.1038/s41467-020-19047-7)
Supplement: Supplementary file 2 — Description of Additional Supplementary Files [file 41467_2020_19047_MOESM2_ESM.docx]

**Description of Additional Supplementary Files**

File Name: **Supplementary Data 1**

Description: Table containing cross-linked peptides of linker histones H1.4 and H5 including cross-linking sites (cross-linked amino acid and nucleotide), DNA adduct (MS1 level), experimental *m/z* values, molecular weight (MW) values, mass errors in parts per million (ppm), and file names.

File Name: **Supplementary Data 2**

Description: Table containing cross-linked peptides identified in *X. Laevis* mononucleosomes (sheet 1), nucleosomal arrays (sheet 2), SCML2-samples (sheet 3) and native HeLa mononucleosomes (sheet 3). Information is provided about: Original .raw file names, spectrum index number, retention time (RT), precursor *m/z*, RNPxl score, precursor charge state, peptide sequence, protein accession numbers, mass error (in ppm), cross-linked nucleotide (RNPxl:NT), DNA adduct on precursor level (RNPxl:RNA), and spectrum numbers refering to Supplementary Data files 5, 6 and 7 displaying TOPPView spectra.

File Name: **Supplementary Data 3**

Description: Modified MaxQuant output file proteingroups.txt summarizing information on proteins identified from purified native HeLa mononucleosomes including log2-transformed iBAQ values, number of identified peptides, summed number of razor and unique peptides, number of unique peptides, sequence coverage, sequence coverage based on unique and razor peptides, sequence coverage based on unique peptides, molecular weight of proteins, MaxQuant q-value, MaxQuant score, intensity, count of MS2 scans, protein IDs, majority protein IDs, protein names and gene names.

File Name: **Supplementary Data 4**

Description: Table containing cross-linked peptides identified from UV cross-linked Hela nuclei (chromatin precipitation, RNPxl setting #1: sheet 1; chromatin precipitation, RNPxl setting #2: sheet 2; SEC: sheet 3). Information is provided about: Original .raw file names, spectrum index number, retention time (RT), precursor *m/z*, RNPxl score, precursor charge state, peptide sequence, protein accession numbers, protein features according to Uniprot, mass error (in ppm), cross-linked nucleotide (RNPxl:NT), DNA adduct on precursor level (RNPxl:RNA), and spectrum numbers refring to Supplementary Data files displaying TOPPView spectra.

File Name: **Supplementary Data 5**

Description: MS/MS (manually annotated and TOPPView) spectra of linker histones (H1.4, H5) and core histones (H2a, H2B, H3, H4). For manually annotated spectra, the identified peptide sequence is shown and the cross-linked amino acid is highlighted in yellow. Black: fragment ions; Red: shifted ion fragments and marker ions.

File Name: **Supplementary Data 6**

Description: MS/MS (manually annotated and TOPPView) spectra of SCML2 cross-link peptides. For the manually validated spectra, the cross-linked amino acid is highlighted in yellow within the peptide sequence. Black: fragment ions; Red: shifted ion fragments and marker ions. The TOPPView spectra also include MS/MS spectra from SCML2 non-irradiated controls ﬁtting to cysteine-deoxyribose phosphate modiﬁed spectra.

File Name: **Supplementary Data 7**

Description: TOPPView MS/MS spectra of cross-linked peptides identiﬁed in high-pH reversed-phase chromatography fractions and input sample of UV cross-linked native human mononucleosomes.

File Name: **Supplementary Data 8**

Description: TOPPView MS/MS spectra of DNA and RNA cross-link peptides identiﬁed in UV cross-linked HeLa nuclei samples: Initial analysis of chromatin-precipitation based enriched sample, second analysis of chromatin-precipitation based enriched sample and size-exclusion chromatography enriched sample.
