# Supplementary material for: Analysis of protein-DNA interactions in chromatin by UV induced cross-linking and mass spectrometry
Source: Nat Commun. 2020 Oct 16;11:5250. doi: 10.1038/s41467-020-19047-7 (PMC7567871; doi:10.1038/s41467-020-19047-7)
Supplement: Supplementary file 5 — Supplementary Data 3 [file 41467_2020_19047_MOESM5_ESM.pdf]

Supplementary Data 3

Analysis of protein-DNA interactions in chromatin by UV induced cross-linking and mass spectrometry

Stützer *et al.*

List of contents

Linker histones + 187bp DNA

Annotated MS/MS spectra of linker histone H1.4 p. 2-9

TOPPView MS/MS spectra of linker histone H1.4 p. 10-16

Annotated MS/MS spectra of linker histone H5 p. 17

X. laevis mononucleosomes and nucleosomal arrays

Annotated MS/MS spectra of core histone H3 p. 18-20

Annotated MS/MS spectra of core histone H2A p. 20-22

Annotated MS/MS spectra of core histone H2B p. 23-24

Annotated MS/MS spectra of core histone H4 p. 25-27

TOPPView MS/MS spectra of core histones from *X. laevis* mononucleosomes p. 28-44

TOPPView MS/MS spectra of core histones from *X. laevis* nucleosomal arrays p. 45-66

Linker histone H1.4

protein	peptide (aa)	cross-link site (aa - base/sugar)	DNA	*m/z* exp	z	cross-link MW exp	cross-link MW calc	peptide MW calc	DNA MW calc	Δ ppm
**H1.4**	**K**ASGPPVSELITK (33-45)	Lys33 - C	ACGT-NH3	854.9878	3	2561.9399	2561.9414	1325.7549	1236.1866	-0.59
		Lys33 - C	CGTT-NH3	851.9836	3	2552.9273	2552.9298	1325.7549	1227.1750	-0.99
		Lys33 - C	AACT-NH3	849.6545	3	2545.9400	2545.9465	1325.7549	1220.1917	-2.56
		Lys33 - C	AC-NH3	965.4271	2	1928.8386	1928.8429	1325.7549	603.0880	-2.24
**H1.4**	KASG**PP**VSELITK (33-45)	Pro37/38 - T	AATT	860.3301	3	2577.9668	2577.9728	1325.7549	1252.2179	-2.32
		Pro37/38 - T	GGTT-NH3	865.3224	3	2592.9437	2592.9360	1325.7549	1267.1812	2.96
		Pro37/38 - T	AAT	758.9818	3	2273.9219	2273.9268	1325.7549	948.1719	-1.86
**H1.4**	ASG**P**PVSELITK (34-45)	Pro37 - T	T-H3PO4	711.8806	2	1421.7456	1421.7397	1197.6600	224.0797	4.13
**H1.4**	ASGPPVSELIT**K**AVAASK (34-51)	Lys45 - C	CC-NH3	769.0228	3	2304.0449	2304.0434	1724.9667	579.0767	0.68
		Lys45 - C	CCC-NH3	865.3712	3	2593.0901	2593.0897	1724.9667	868.1230	0.16
		Lys45 - C	AC-NH3	777.0282	3	2328.0611	2328.0547	1724.9667	603.0880	2.77
		Lys45 - C	ACCC-NH3	727.5435	4	2906.1427	2906.1473	1724.9667	1181.1807	-1.60
**H1.4**	SGVSLAAL**K**K (54-63)	Lys62 - C	CT-NH3	784.3438	2	1566.6720	1566.6727	972.5963	594.0764	-0.46
		Lys62 - C	ACT-NH3	940.8710	2	1879.7264	1879.7303	972.5963	907.1340	-2.10
		Lys62 - C	CGT-NH3	632.9144	3	1895.7197	1895.7252	972.5963	923.1289	-2.89
		Lys62 - C	CG-NH3	796.8442	2	1591.6728	1591.6792	972.5963	619.0829	-4.03
**H1.4**	**K**ALAAAGYDVEK (63-74)	Lys63 - C	CG-NH3	618.9192	3	1853.7341	1853.7381	1234.6552	619.0829	-2.13
		Lys63 - C	CCG-NH3	715.2669	3	2142.7772	2142.7844	1234.6552	908.1292	-3.35
		Lys63 - C	CCT-NH3	706.9329	3	2117.7752	2117.7779	1234.6552	883.1227	-1.27
		Lys63 - C	ACT-NH3	714.9366	3	2141.7863	2141.7892	1234.6552	907.1340	-1.34
**H1.4**	ALAAAGYDVE**K**NNSR (64-78)	Lys74 - C	CT-NH3	1086.9341	2	2171.8526	2171.8557	1577.7793	594.0764	-1.43
		Lys74 - C	CG-NH3	733.2930	3	2196.8555	2196.8622	1577.7793	619.0829	-3.02
		Lys74 - C	ACC-NH3	824.3106	3	2469.9083	2469.9136	1577.7793	892.1343	-2.14
		Lys74 - C	ACT-NH3	829.3095	3	2484.9050	2484.9133	1577.7793	907.1340	-3.33
**H1.4**	I**K**LGLK (79-84)	Lys80 - C	ACG-NH3	802.3120	2	1602.6084	1602.6141	670.4736	932.1405	-3.59
		Lys80 - C	CT-NH3	633.2813	2	1264.5470	1264.5500	670.4736	594.0764	-2.38
		Lys80 - C	CGT-NH3	797.8080	2	1593.6004	1593.6025	670.4736	923.1289	-1.35
		Lys80 - C	CC-NH3	625.7816	2	1249.5476	1249.5503	670.4736	579.0767	-2.17
**H1.4**	LGL**K**SLVSK (81-89)	Lys84 - C	ACGT-NH3	1090.9069	2	2179.7982	2179.7926	943.6061	1236.1866	2.53
		Lys84 - C	CGGT-NH3	732.9365	3	2195.7860	2195.7875	943.6061	1252.1815	-0.69
		Lys84 - C	CGT-NH3	934.3757	2	1866.7358	1866.7350	943.6061	923.1289	0.40
		Lys84 - C	CTT-NH3	921.8704	2	1841.7252	1841.7285	943.6061	898.1224	-1.82
**H1.4**	SLVS**K**GTLVQTK (85-96)	Lys89 - C	CG-NH3	940.4194	2	1878.8232	1878.8273	1259.7444	619.0829	-2.19
		Lys89 - C	AAC-NH3	726.3054	3	2175.8927	2175.8900	1259.7444	916.1456	1.25
		Lys89 - C	ACCG-NH3	827.9844	3	2480.9297	2480.9312	1259.7444	1221.1869	-0.61
		Lys89 - C	AC-NH3	932.4214	2	1862.8272	1862.8324	1259.7444	603.0880	-2.80
**H1.4**	GTGASGSF**K**LNK (97-108)	Lys105 - T	T-H3PO4	464.2390	3	1389.6935	1389.6883	1165.6086	224.0797	3.78
**H1.4**	GTGASGSF**K**LNKK (97-109)	Lys105 - C	ACCG-NH3	629.7283	4	2514.8819	2514.8904	1293.7036	1221.1869	-3.40
		Lys105 - C	CCG-NH3	734.9493	3	2201.8244	2201.8328	1293.7036	908.1292	-3.81
		Lys105 - C	ACT-NH3	734.6190	3	2200.8335	2200.8376	1293.7036	907.1340	-1.85
		Lys105 - C	CC-NH3	625.2660	3	1872.7745	1872.7803	1293.7036	579.0767	-3.06
**H1.4**	**K**AASGEAKPK (109-118)	Lys109 - C	ACCG-NH3	736.5859	3	2206.7342	2206.7419	985.5551	1221.1869	-3.50
		Lys109 - C	AACC-NH3	731.2549	3	2190.7412	2190.7470	985.5551	1205.1920	-2.66
**H1.4**	A**K**KPAGAAK (127-135)	Lys128 - C	ACCG-NH3	688.2408	3	2061.6989	2061.7044	840.5176	1221.1869	-2.68
		Lys128 - C	AACC-NH3	682.9091	3	2045.7038	2045.7095	840.5176	1205.1920	-2.80
		Lys128 - C	ACC-NH3	578.5570	3	1732.6475	1732.6519	840.5176	892.1343	-2.53
		Lys128 - C	AAC-NH3	586.5613	3	1756.6604	1756.6632	840.5176	916.1456	-1.58
**H1.4**	KPAGAA**K**KPK (129-138)	Lys135 - C	ACC-NH3	629.9267	3	1886.7566	1886.7625	994.6282	892.1343	-3.12
		Lys135 - C	AAC-NH3	637.9318	3	1910.7719	1910.7738	994.6282	916.1456	-0.98
		Lys135 - C	ACCG-NH3	739.6117	3	2215.8116	2215.8150	994.6282	1221.1869	-1.54
		Lys135 - C	CCG-NH3	635.2580	3	1902.7505	1902.7574	994.6282	908.1292	-3.62
**H1.4**	**K**ATGAATPK (139-147)	Lys139 - C	ACCG-NH3	689.2292	3	2064.6641	2064.6677	843.4809	1221.1869	-1.75
		Lys139 - C	CCGG-NH3	694.5621	3	2080.6628	2080.6626	843.4809	1237.1818	0.09
		Lys139 - C	ACGT-NH3	694.2296	3	2079.6653	2079.6674	843.4809	1236.1866	-1.02
		Lys139 - C	CCGT-NH3	686.2266	3	2055.6563	2055.6561	843.4809	1212.1753	0.09
**H1.4**	KATGAATP**K**K (139-148)	Lys147 - C	ACCT-NH3	723.5920	3	2167.7525	2167.7562	971.5759	1196.1804	-1.72
		Lys147 - C	ACCG-NH3	731.9276	3	2192.7593	2192.7627	971.5759	1221.1869	-1.56
		Lys147 - C	ACCC-NH3	718.5917	3	2152.7516	2152.7565	971.5759	1181.1807	-2.29
**H1.4**	**K**AKKPAAAAGAK (156-167)	Lys156 - C	ACT-NH3	673.6133	3	2017.8164	2017.8208	1110.6868	907.1340	-2.17
		Lys156 - C	CGT-NH3	678.9455	3	2033.8130	2033.8157	1110.6868	923.1289	-1.32
		Lys156 - C	ACCG-NH3	778.2985	3	2331.8720	2331.8736	1110.6868	1221.1869	-0.69
		Lys156 - C	CT-NH3	569.2610	3	1704.7595	1704.7632	1110.6868	594.0764	-2.14
**H1.4**	KPAAAAGA**K**K (159-168)	Lys167 - C	ACCG-NH3	711.9207	3	2132.7386	2132.7415	911.5547	1221.1869	-1.37
		Lys167 - C	ACCC-NH3	698.5844	3	2092.7297	2092.7353	911.5547	1181.1807	-2.69
		Lys167 - C	ACC-NH3	602.2365	3	1803.6860	1803.6890	911.5547	892.1343	-1.65
		Lys167 - C	AAC-NH3	610.2402	3	1827.6971	1827.7003	911.5547	916.1456	-1.74
**H1.4**	**K**A**K**AAKPK (174-181)	Lys174/Lys176 - C	ACCG-NH3	516.4416	4	2061.7351	2061.7408	840.5540	1221.1869	-2.79
	(likely co-elution of 2 cross-links)	Lys174/Lys176 - C	ACCC-NH3	674.9178	3	2021.7299	2021.7346	840.5540	1181.1807	-2.33
		Lys174/Lys176 - C	ACT-NH3	583.5688	3	1747.6829	1747.6880	840.5540	907.1340	-2.91
**H1.4**	A**K**AVKPK (190-196)	Lys191 - C	ACT-NH3	550.2144	3	1647.6197	1647.6243	740.4903	907.1340	-2.78
		Lys191 - C	ACGT-NH3	659.8982	3	1976.6711	1976.6768	740.4903	1236.1866	-2.89
		Lys191 - C	CCG-NH3	550.5467	3	1648.6166	1648.6195	740.4903	908.1292	-1.75
		Lys191 - C	CGT-NH3	555.5461	3	1663.6148	1663.6192	740.4903	923.1289	-2.63
**H1.4**	AVKP**K**AAKPK (192-201)	Lys196 - C	CGT-NH3	654.2748	3	1959.8009	1959.8041	1036.6752	923.1289	-1.62
		Lys196 - C	AACT-NH3	565.2228	4	2256.8599	2256.8668	1036.6752	1220.1917	-3.08
		Lys196 - C	ACGT-NH3	569.2213	4	2272.8539	2272.8617	1036.6752	1236.1866	-3.45
		Lys196 - C	ACG-NH3	657.2778	3	1968.8099	1968.8157	1036.6752	932.1405	-2.94
**H1.4**	AAKP**K**TAKPK (197-206)	Lys201 - C	ACCG-NH3	565.9667	4	2259.8355	2259.8412	1038.6544	1221.1869	-2.54
		Lys201 - C	CCT-NH3	961.8959	2	1921.7762	1921.7771	1038.6544	883.1227	-0.50
		Lys201 - C	AAC-NH3	652.6061	3	1954.7948	1954.8000	1038.6544	916.1456	-2.65
		Lys201 - C	ACC-NH3	644.6018	3	1930.7819	1930.7887	1038.6544	892.1343	-3.51
**H1.4**	TAKP**K**AAKPK (202-211)	Lys206 - C	ACT-H2O	649.2745	3	1944.8000	1944.8044	1038.6544	906.1500	-2.25
		Lys206 - C	ACCT-H2O	745.6240	3	2233.8485	2233.8507	1038.6544	1195.1964	-0.99
		Lys206 - C	CCCG-NH3	746.2853	3	2235.8324	2235.8299	1038.6544	1197.1756	1.11
		Lys206 - C	AACC-NH3	561.9680	4	2243.8407	2243.8463	1038.6544	1205.1920	-2.52

Annotated MS/MS spectra of linker histone H1.4

1) **Histone H1.4, aa 33-45**

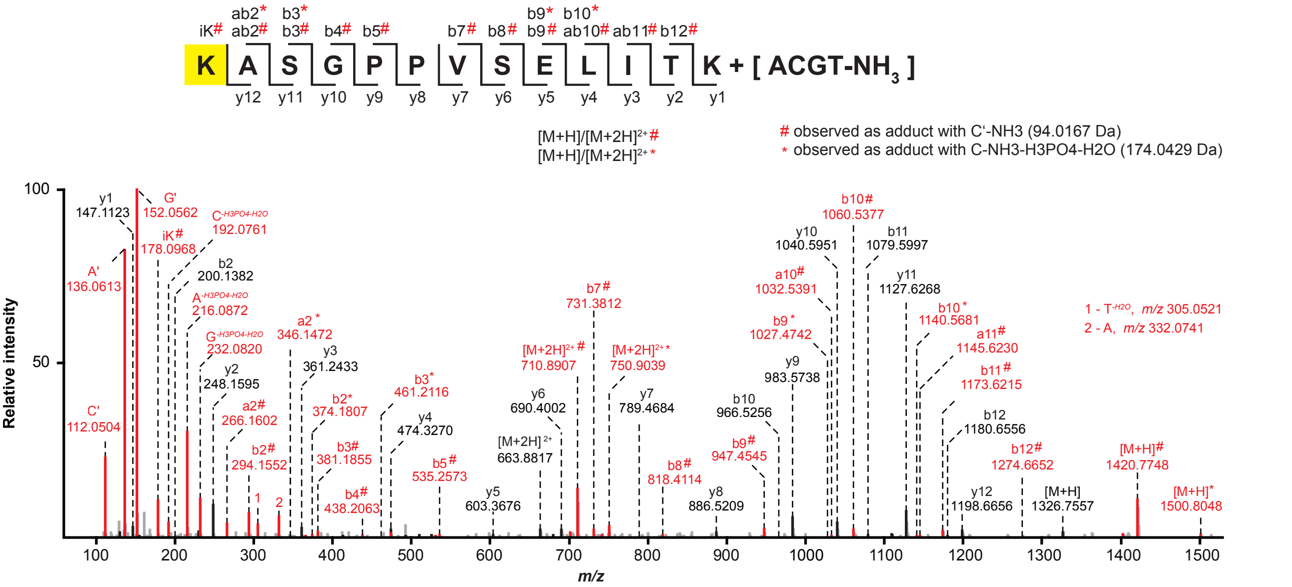

File: A_Stuetzer_251115_H14_187_25HCD; Index No. 6657; RT: 1515.67 s; *m/z:* 854.988; z: 3

2) **Histone H1.4, aa 33-45**

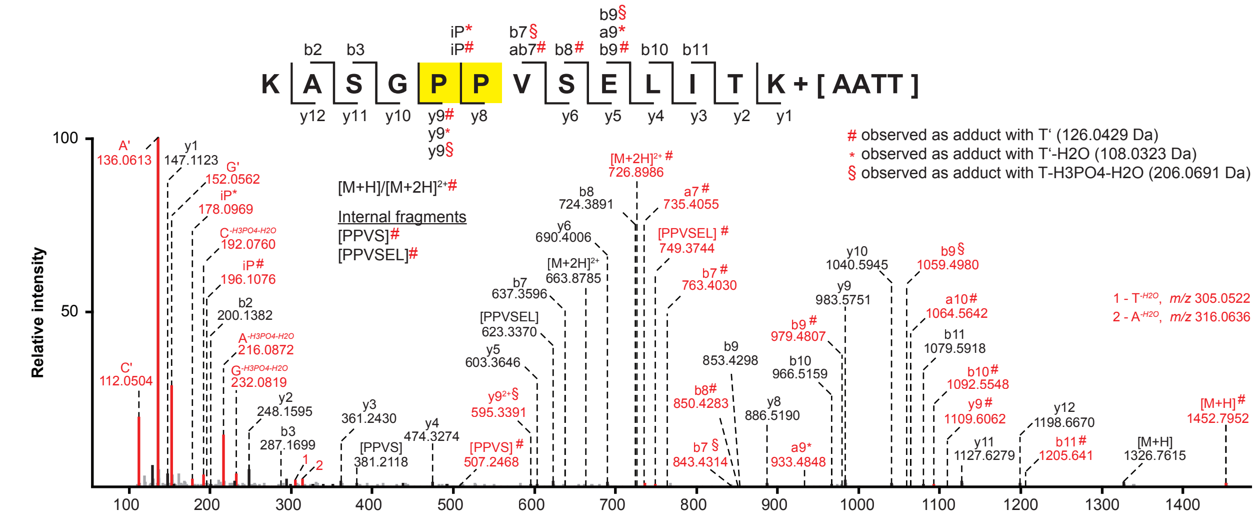

File: ; Index No. ; RT: s; *m/z:* 860.330; z: 3

3) **Histone H1.4, aa 34-45**

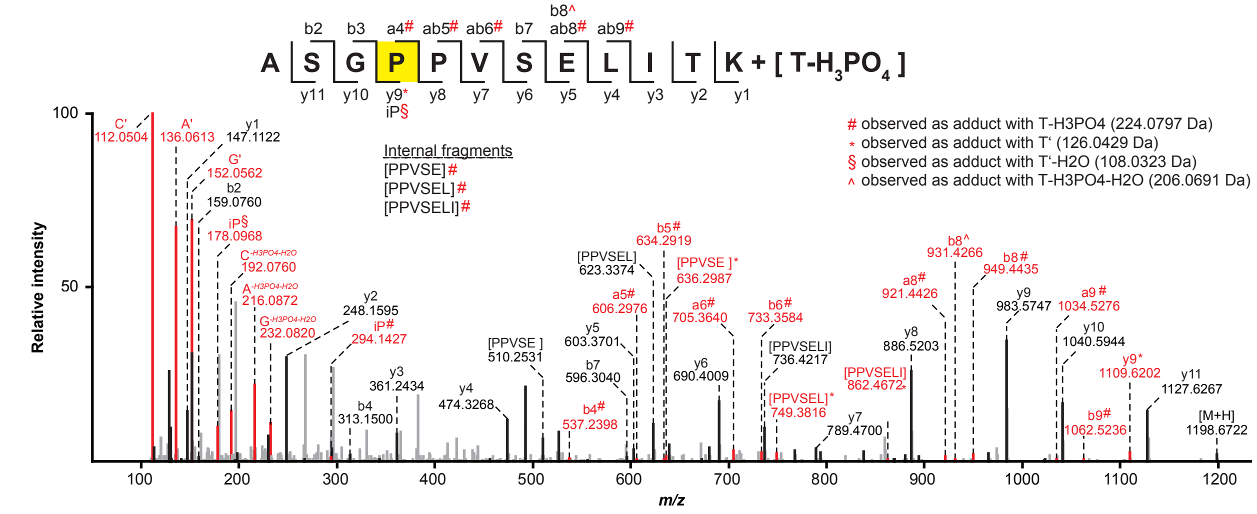

File: A_Stuetzer_251115_H14_187_25HCD; Index No. 6047; RT: 1404.55 s; *m/z:* 711.881; z: 2

4) Histone H1.4, aa 34-51

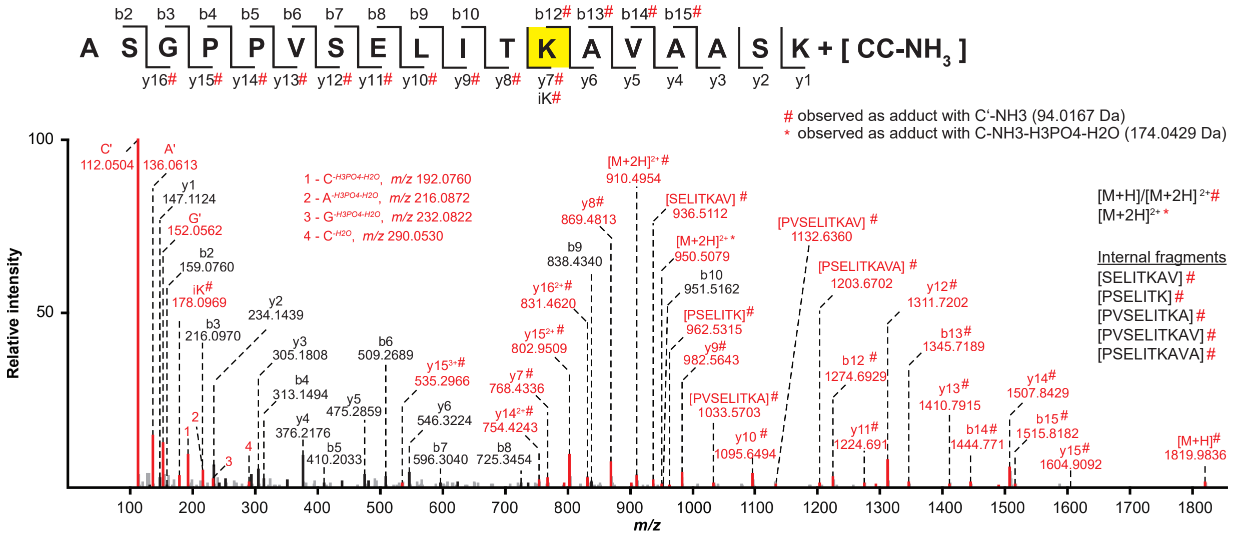

File: A_Stuetzer_251115_H14_187_25HCD; Index No. 7564; RT: 1681.3 s; *m/z:* 769.023; z: 3

5) Histone H1.4, aa 54-63

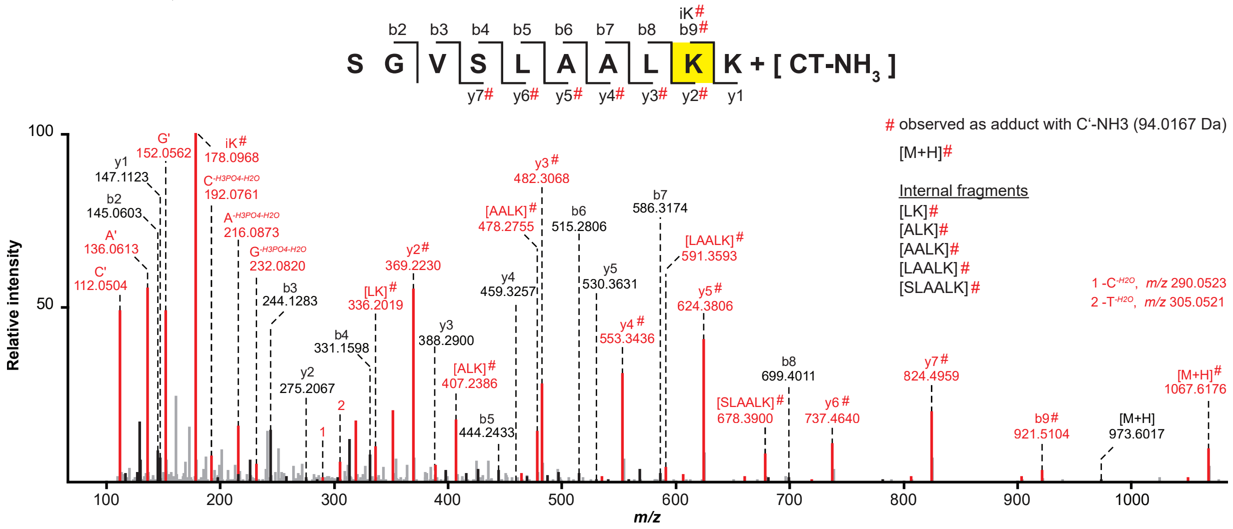

File: A_Stuetzer_251115_H14_187_30HCD; Index No. 6039; RT: 1413.53 s; *m/z:* 784.343; z: 2

6) Histone H1.4, aa 63-74

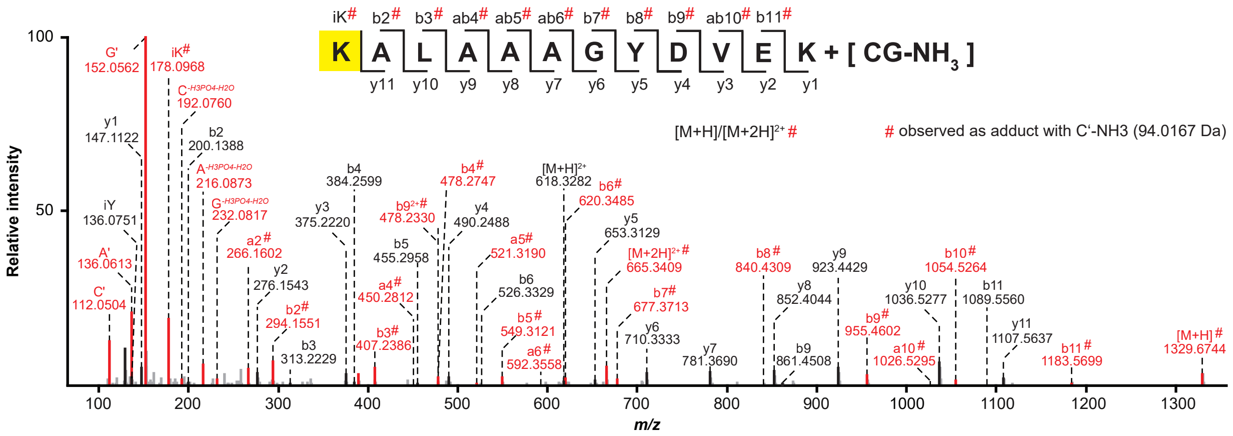

File: A_Stuetzer_251115_H14_187_25HCD; Index No. 5153; RT: 1245.88 s; *m/z:* 618.919; z: 3

7) Histone H1.4, aa 64-78

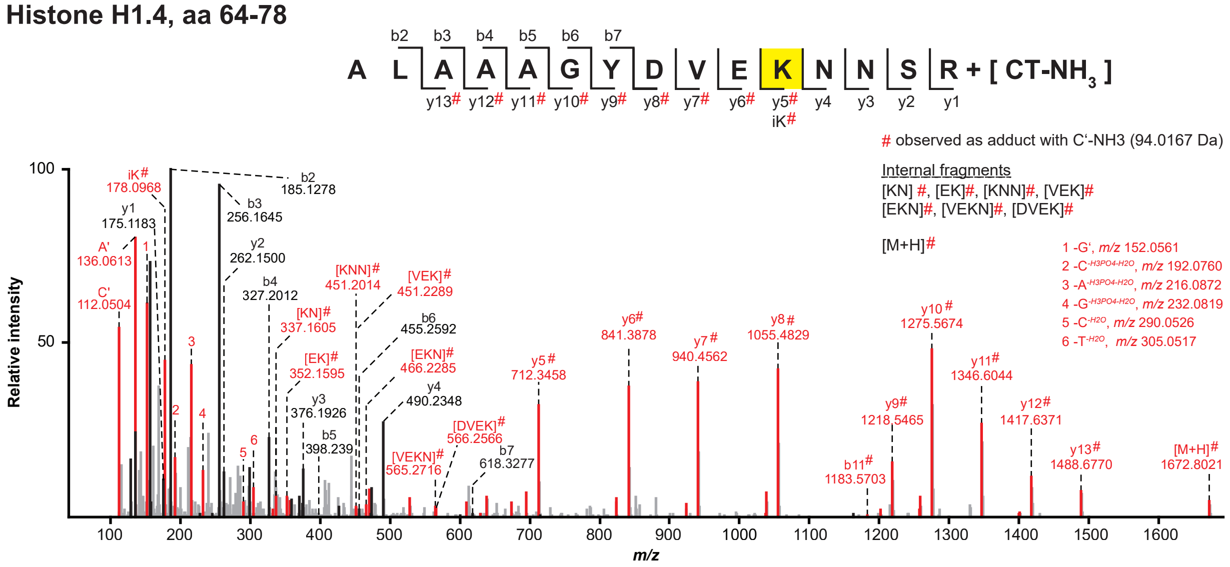

File: A_Stuetzer_251115_H14_187_30HCD; Index No. 5109; RT: 1248.97 s; *m/z:* 1086.934; z: 2

8) Histone H1.4, aa 79-84

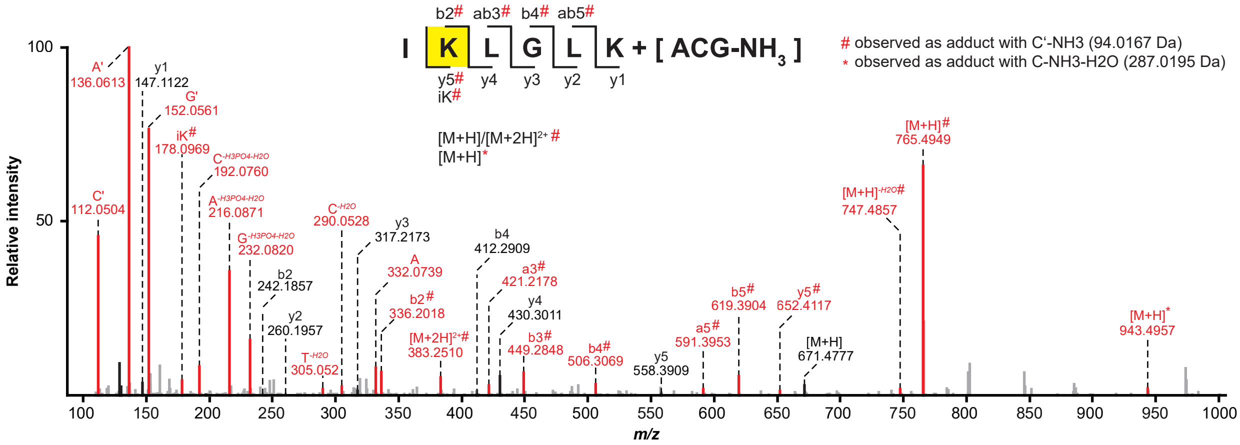

File: A_Stuetzer_251115_H14_187_25HCD; Index No. 6046; RT: 1404.4 s; *m/z:* 802.312; z: 2

9) Histone H1.4, aa 81-89

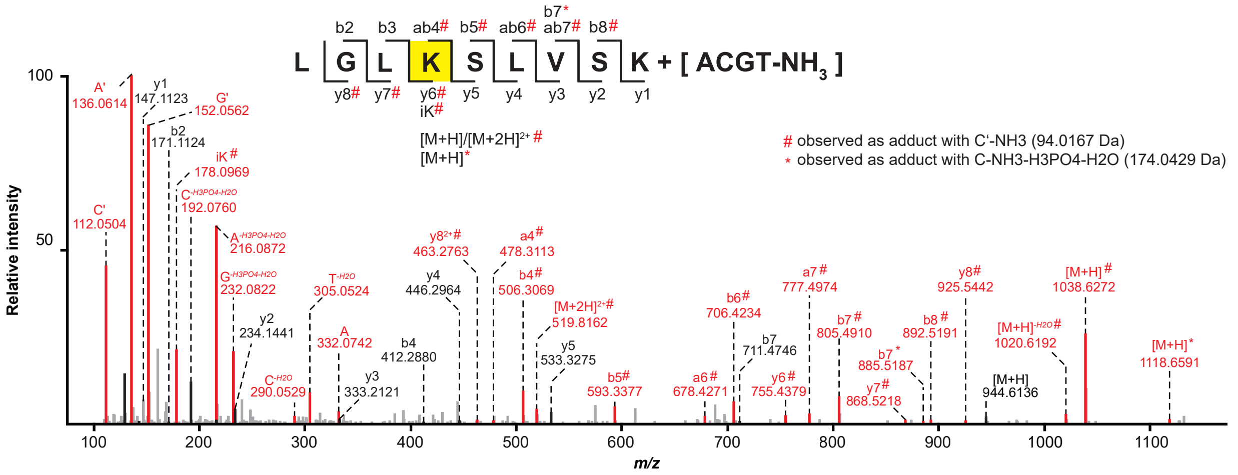

File: A_Stuetzer_251115_H14_187_30HCD; Index No. 6896; RT: 1571.62 s; *m/z:* 1090.907; z: 2

10) Histone H1.4, aa 85-96

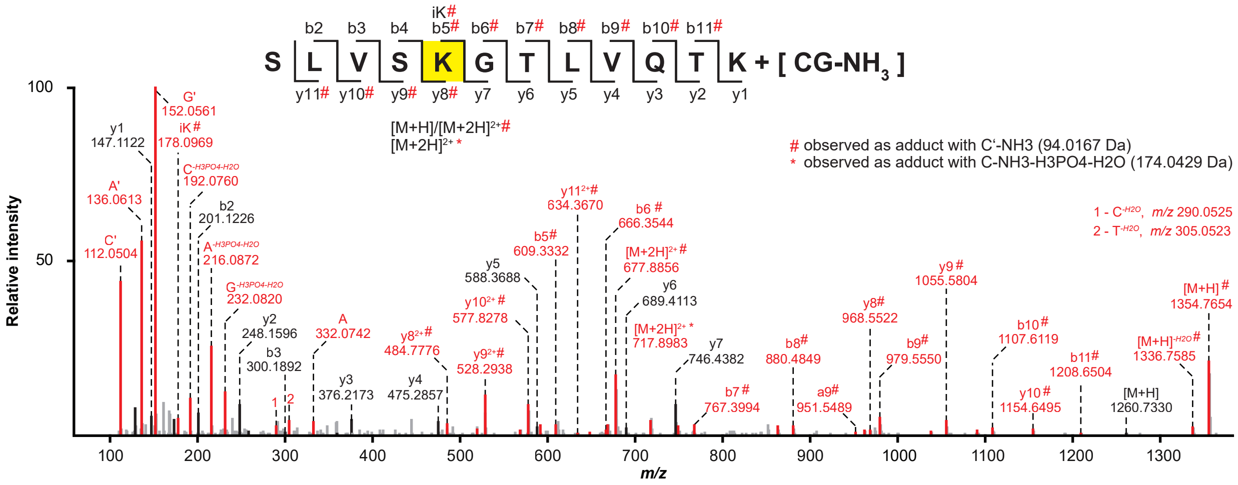

File: A_Stuetzer_251115_H14_187_25HCD; Index No. 5645; RT: 1331.96 s; *m/z:* 940.4194; z: 2

11) Histone H1.4, aa 97-108

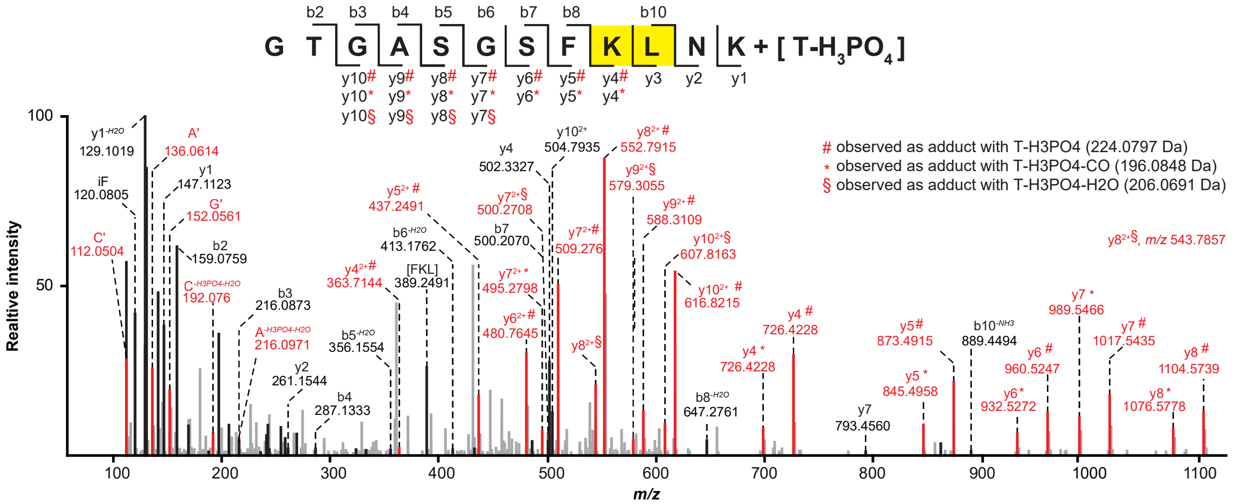

File: A_Stuetzer_251115_H14_187_20HCD; Index No. 4436; RT: 1096.92 s; *m/z:* 464.239; z: 3

12) Histone H1.4, aa 97-109

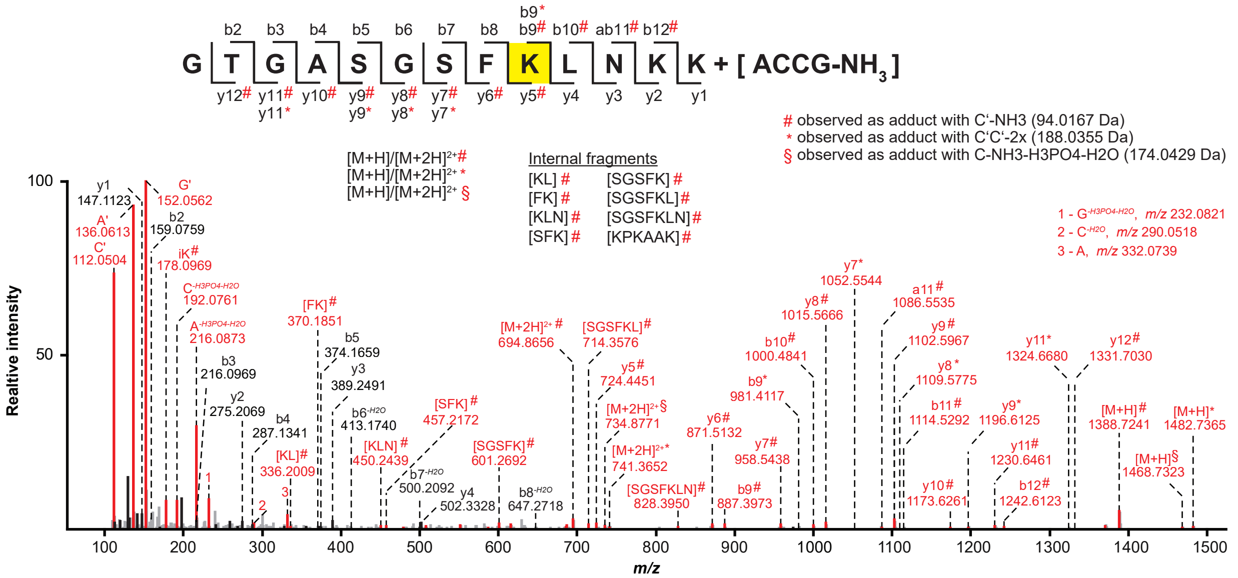

File: A_Stuetzer_251115_H14_187_30HCD; Index No. 4470; RT: 1137.05 s; *m/z:* 629.728; z: 4

13) Histone H1.4, aa 109-118

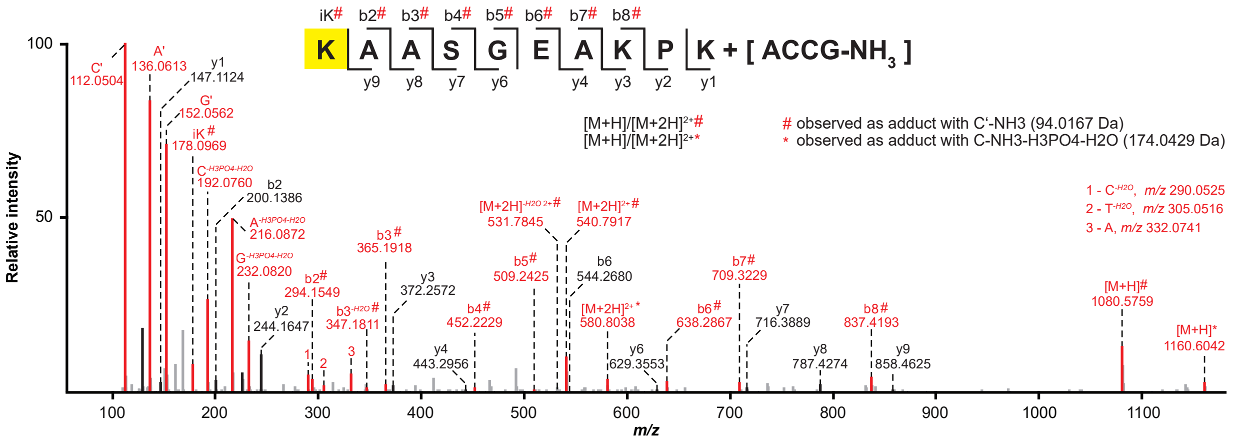

File: A_Stuetzer_251115_H14_187_25HCD; Index No. 3061; RT: 875.201 s; *m/z:* 736.586; z: 3

14) Histone H1.4, aa 127-135

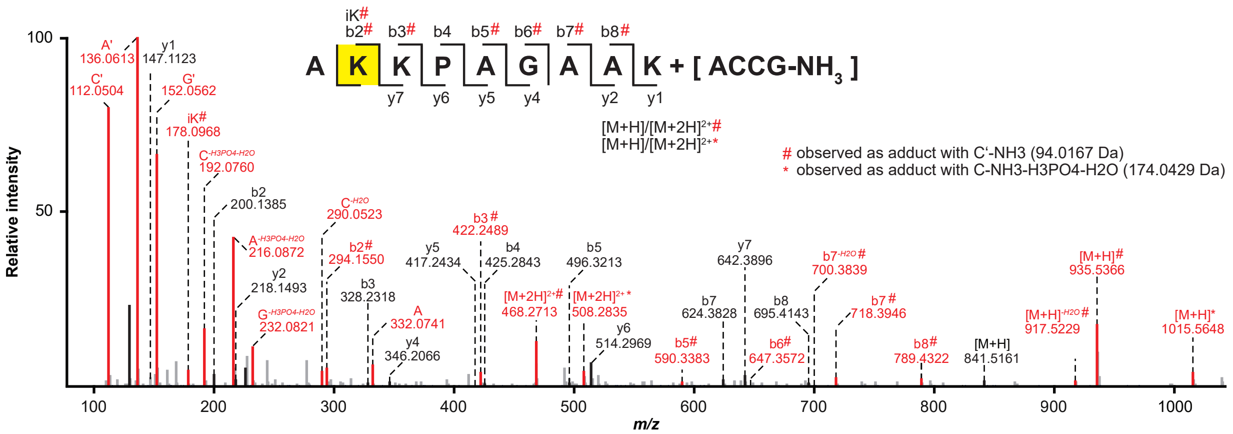

File: A_Stuetzer_251115_H14_187_25HCD; Index No. 3076; RT: 877.92 s; *m/z:* 688.2408; z: 3

15) Histone H1.4, aa 129-138

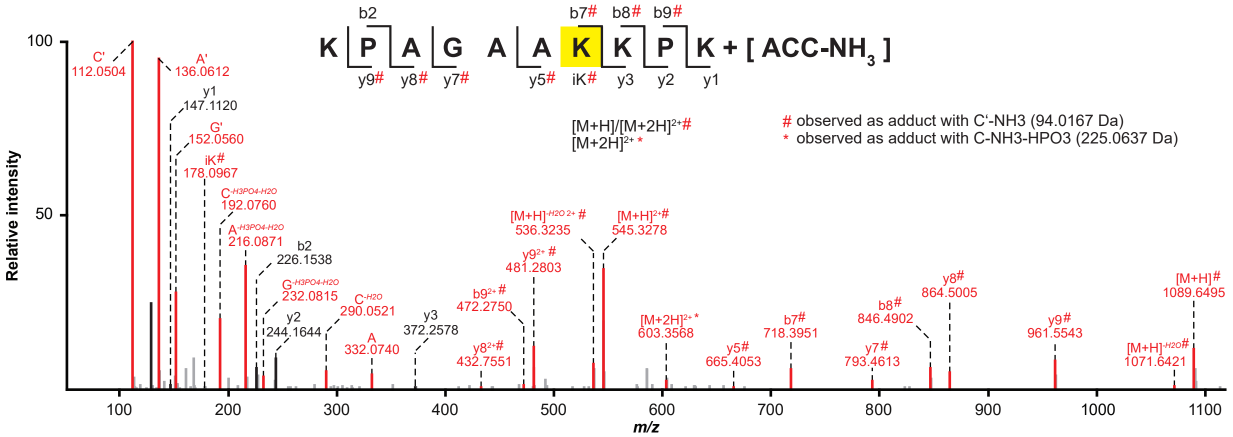

File: A_Stuetzer_251115_H14_187_25HCD; Index No. 2944; RT: 893.932 s; *m/z:* 629.927; z: 3

16) Histone H1.4, aa 139-147

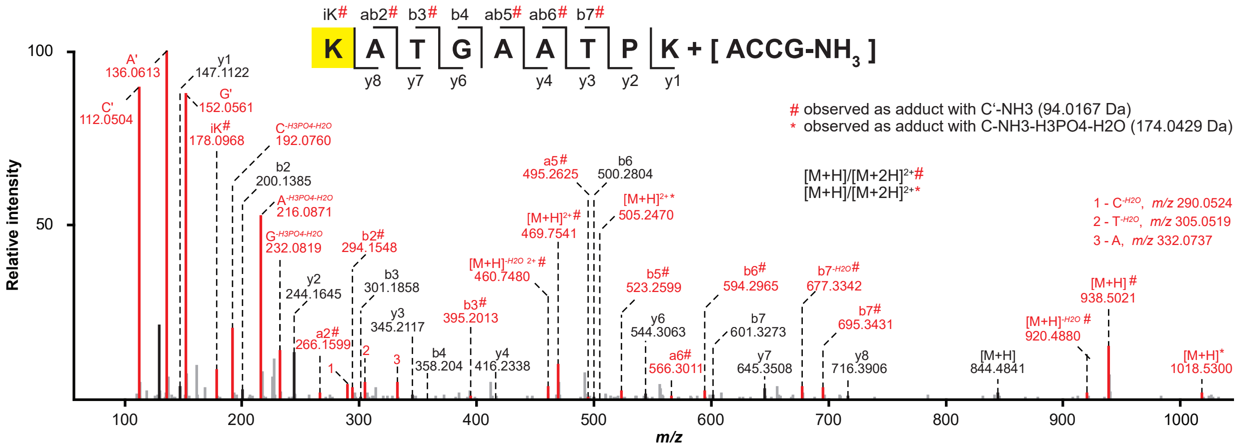

File: A_Stuetzer_251115_H14_187_25HCD; Index No. 3451; RT: 944.58 s; *m/z:* 689.229; z: 3

17) Histone H1.4, aa 139-148

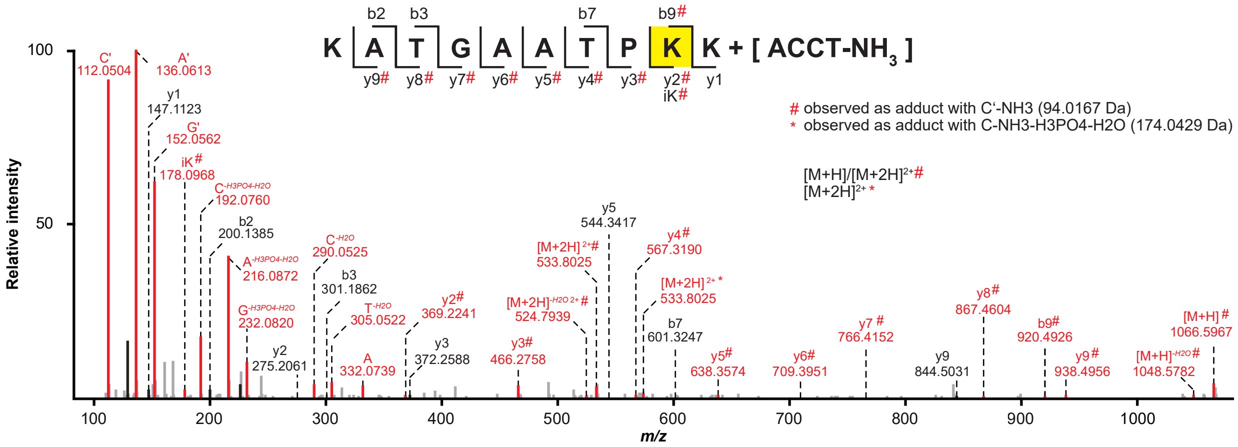

File: ; Index No. ; RT: s; *m/z:* 723.592; z: 3

18) Histone H1.4, aa 156-167

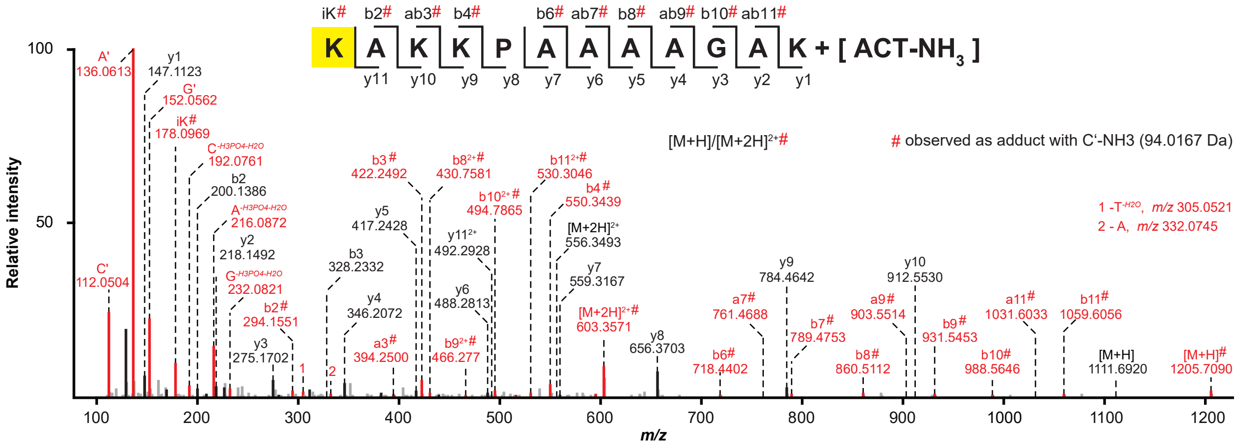

File: A_Stuetzer_251115_H14_187_30HCD; Index No. 3288; RT: 918.355 s; *m/z:* 673.613; z: 3

19) Histone H1.4, aa 159-168

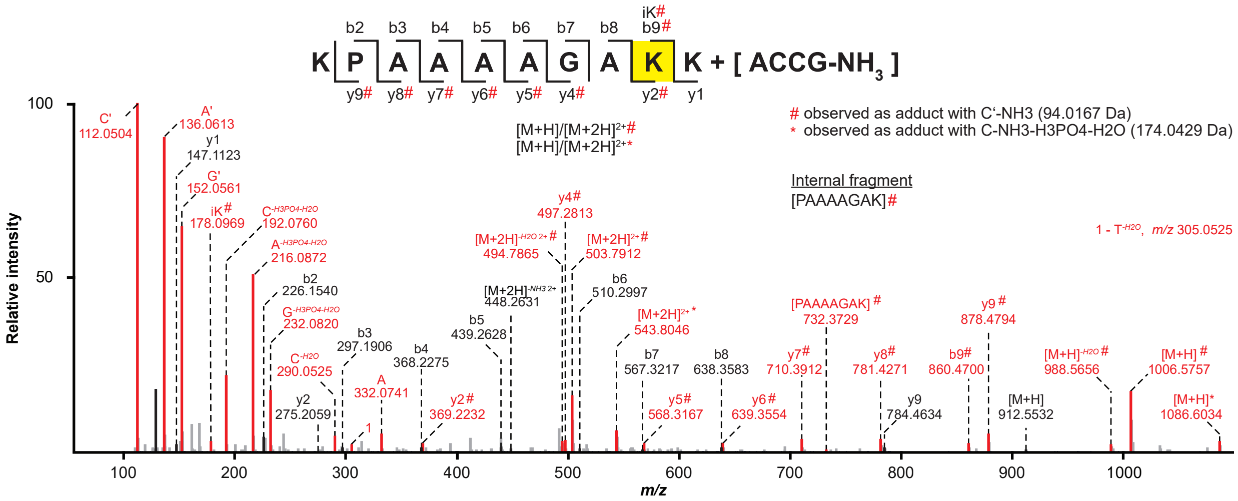

File: A_Stuetzer_251115_H14_187_25HCD; Index No. 3167; RT: 893.932 s; *m/z:* 711.921; z: 3

20) Histone H1.4, aa 174-181

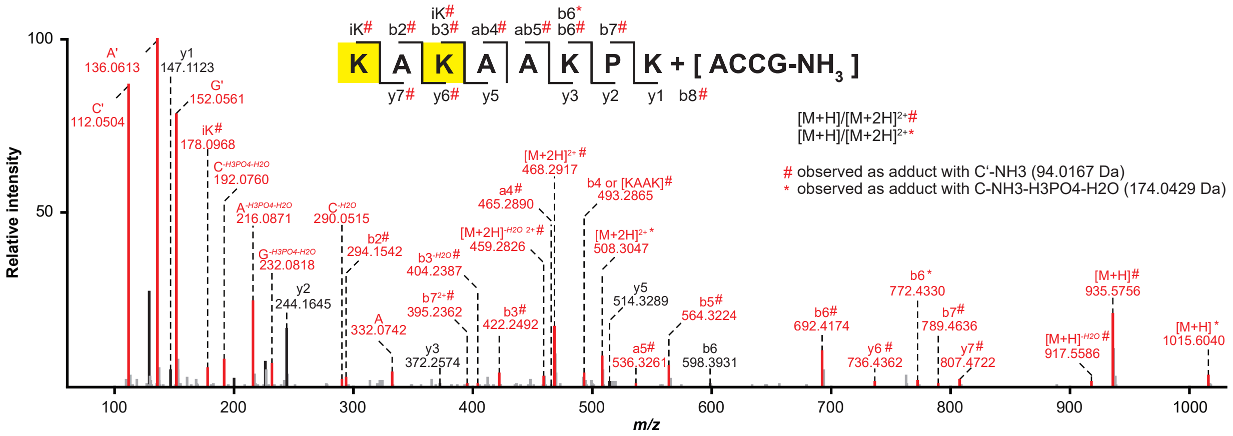

File: A_Stuetzer_251115_H14_187_25HCD; Index No. 2977; RT: 860.255 s; *m/z:* 516.442; z: 4

21) Histone H1.4, aa 190-196

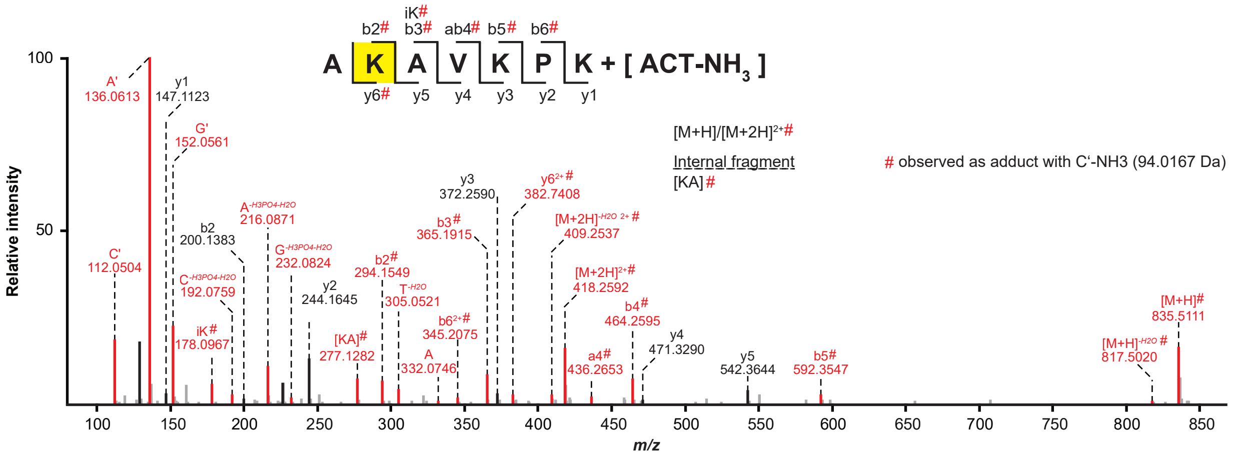

File: A_Stuetzer_251115_H14_187_25HCD; Index No. 3383; RT: 932.517 s; *m/z:* 550.214; z: 3

22) Histone H1.4, aa 192-201

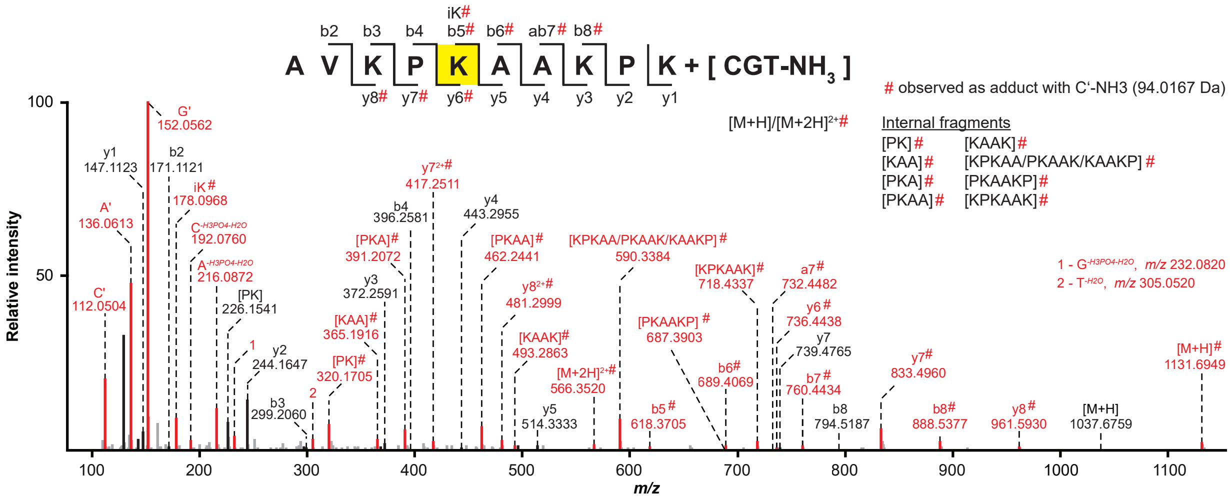

File: ; Index No. ; RT: s; *m/z:* 550.214; z: 3

23) Histone H1.4, aa 197-206

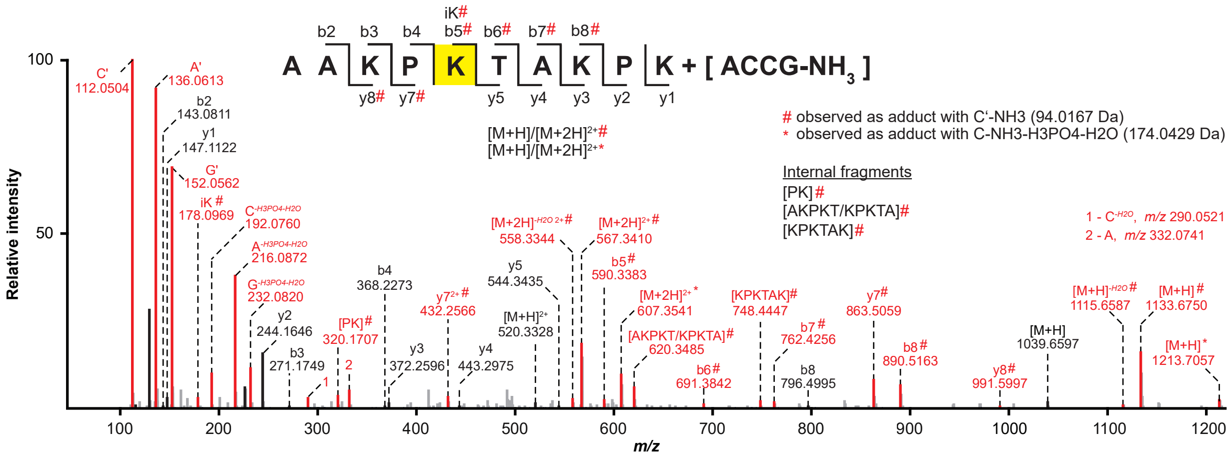

File: A_Stuetzer_251115_H14_187_25HCD; Index No. 3077; RT: 878.068 s; *m/z:* 565.967; z: 4

24) Histone H1.4, aa 202-211

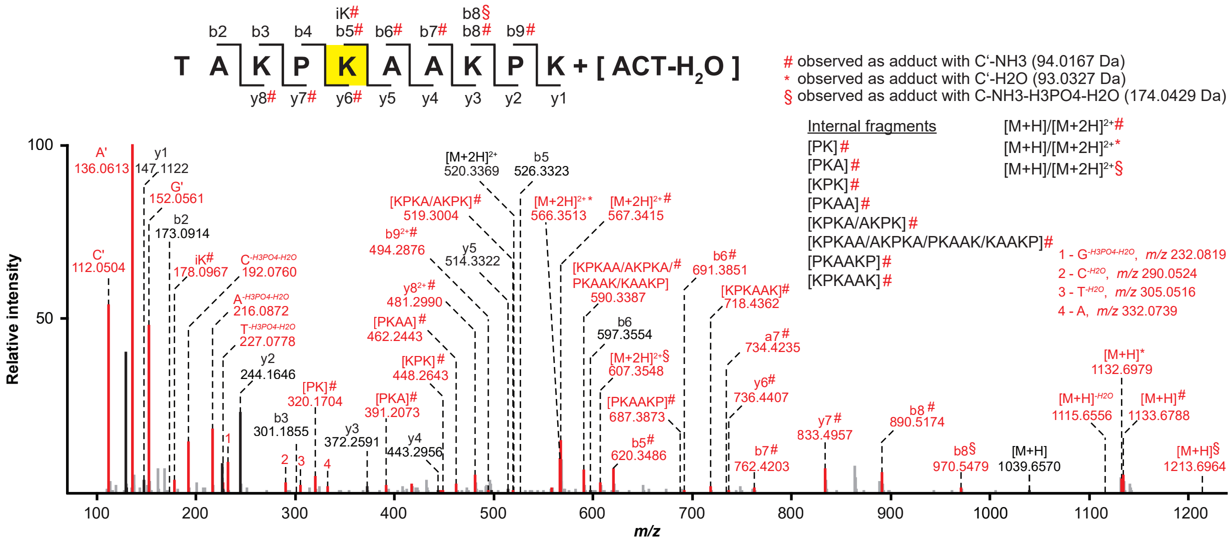

File: ; Index No. ; RT: s; *m/z:* 649.275; z: 3

TOPPView spectra of Linker histone H1.4

1)

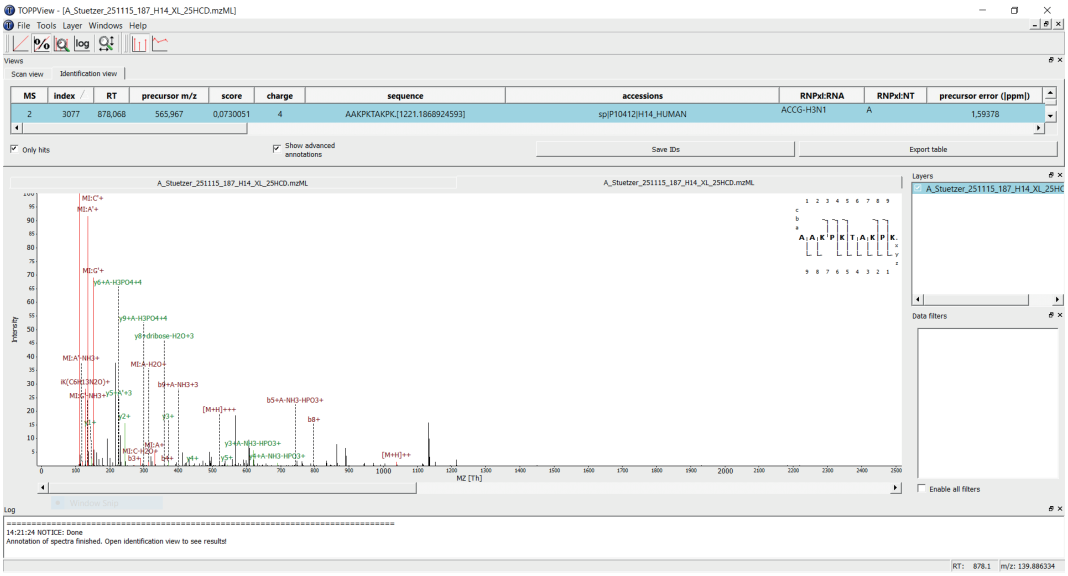

2)

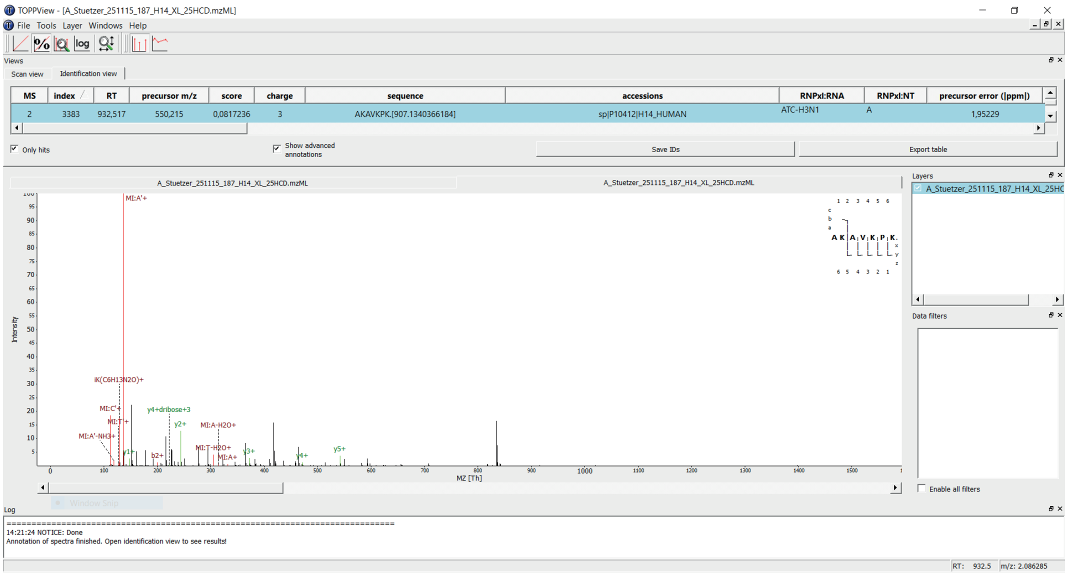

3)

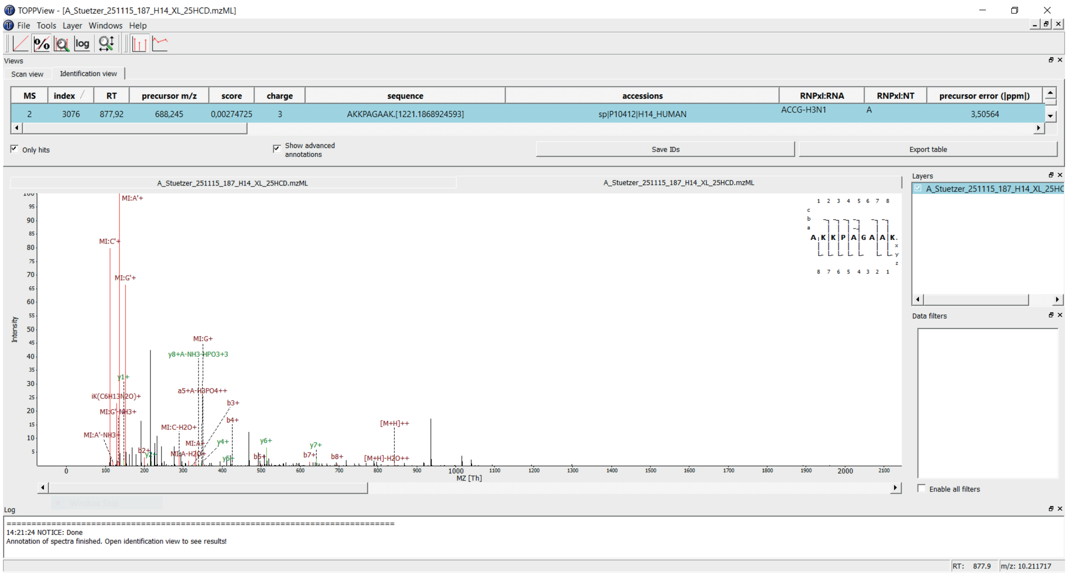

4)

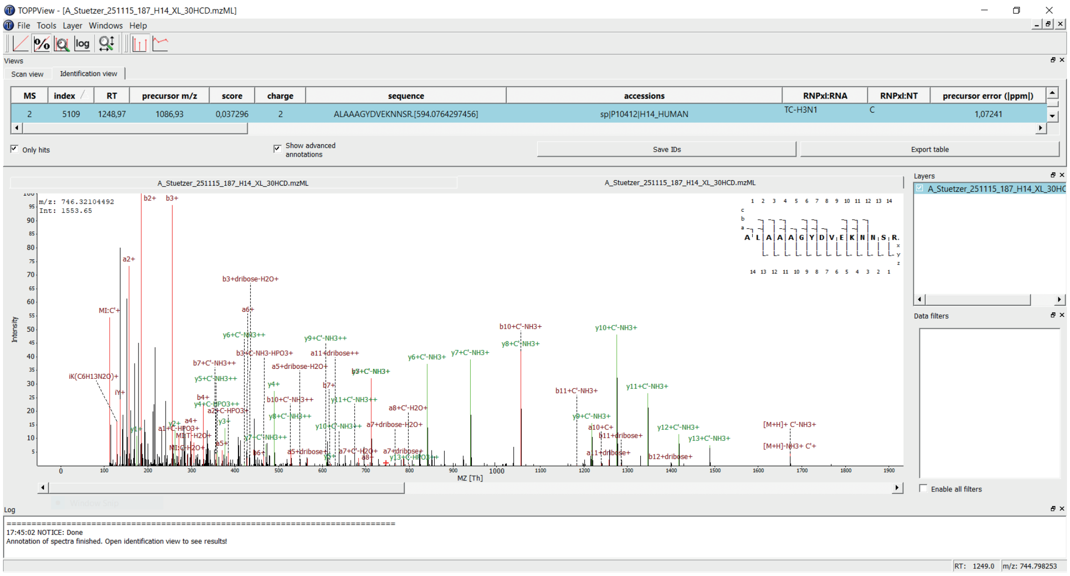
TOPPView - [A_Stuetzer_251115_187_H14_XL_30HCD.mzML]
MS | index | RT | precursor m/z | score | charge | sequence | accessions | RNPxl:RNA | RNPxl:NT | precursor error (|ppm|)
2 | 5109 | 1248,97 | 1086,93 | 0,037296 | 2 | ALAAAGYDVEKNNSR.[594.0764297456] | sp|P10412|H14_HUMAN | TC-H3N1 | C | 1,07241
Annotation of spectra finished. Open identification view to see results!

5)

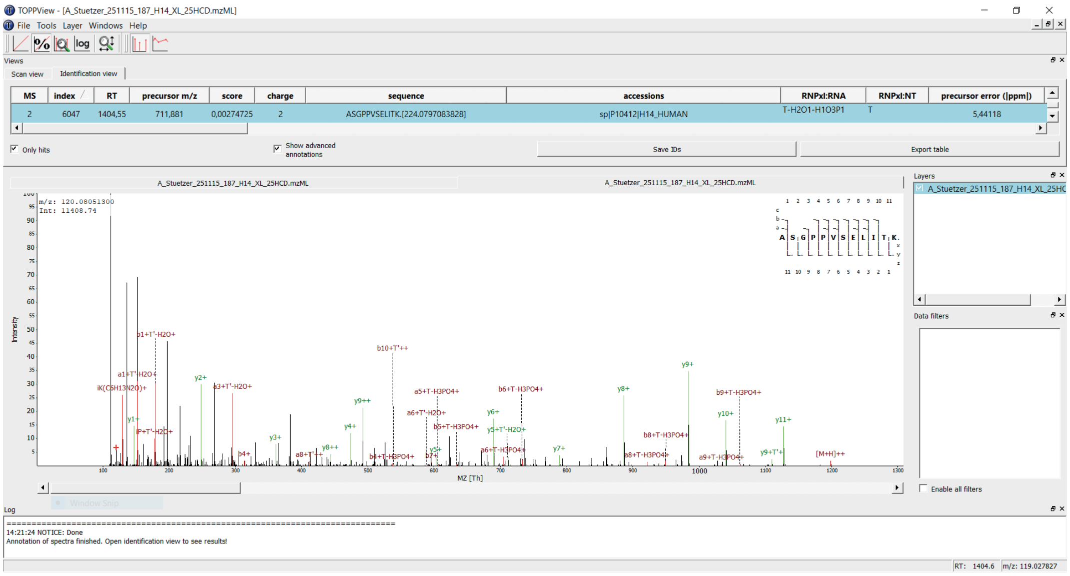
TOPPView - [A_Stuetzer_251115_187_H14_XL_25HCD.mzML]
MS | index | RT | precursor m/z | score | charge | sequence | accessions | RNPxl:RNA | RNPxl:NT | precursor error (|ppm|)
2 | 6047 | 1404,55 | 711,881 | 0,00274725 | 2 | ASGPPVSELITK.[224.0797083828] | sp|P10412|H14_HUMAN | T-H2O1-H1O3P1 | T | 5,44118
Annotation of spectra finished. Open identification view to see results!

6)

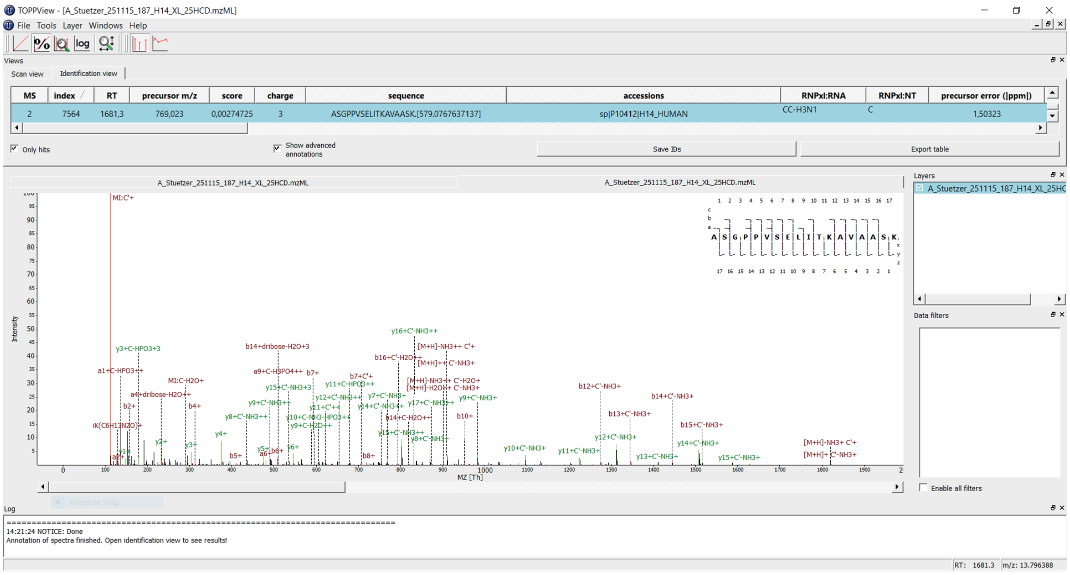
TOPPView - [A_Stuetzer_251115_187_H14_XL_25HCD.mzML]
MS | index | RT | precursor m/z | score | charge | sequence | accessions | RNPxl:RNA | RNPxl:NT | precursor error (|ppm|)
2 | 7564 | 1681,3 | 769,023 | 0,00274725 | 3 | ASGPPVSELITKAVAASK.[579.0767637137] | sp|P10412|H14_HUMAN | CC-H3N1 | C | 1,50323
Annotation of spectra finished. Open identification view to see results!

7)

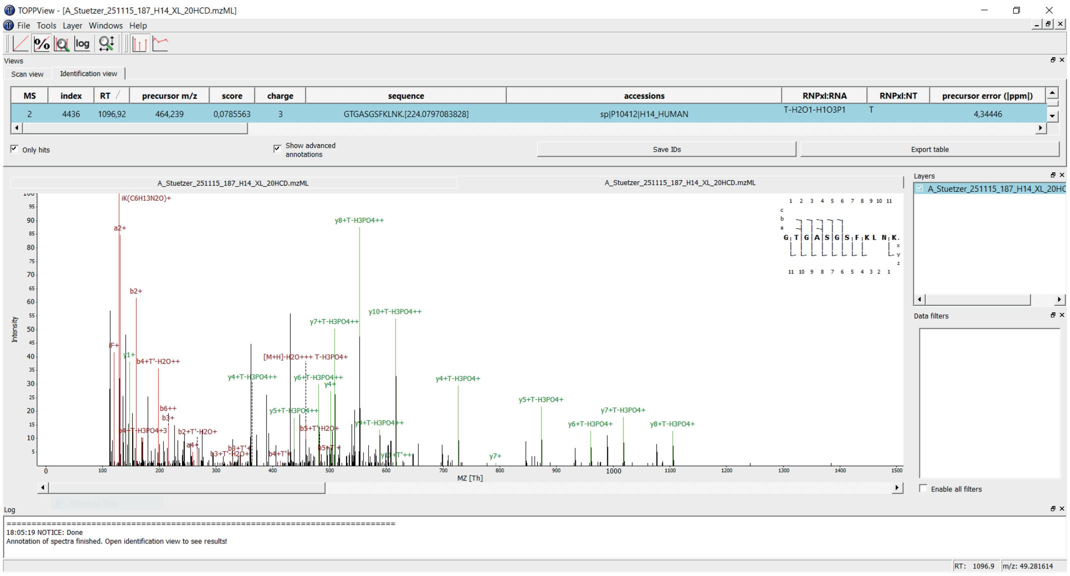
TOPPView - [A_Stuetzer_251115_187_H14_XL_20HCD.mzML]
File Tools Layer Windows Help
Scan view Identification view
MS | index | RT | precursor m/z | score | charge | sequence | accessions | RNPxl:RNA | RNPxl:NT | precursor error (|ppm|)
2 | 4436 | 1096,92 | 464,239 | 0,0785563 | 3 | GTGASGSFKLNK.[224.0797083828] | sp|P10412|H14_HUMAN | T-H2O1-H1O3P1 | T | 4,34446
Only hits
Show advanced annotations
Save IDs
Export table
A_Stuetzer_251115_187_H14_XL_20HCD.mzML
MZ [Th]
Intensity
Layers
Data filters
Enable all filters
Log
18:05:19 NOTICE: Done
Annotation of spectra finished. Open identification view to see results!
RT: 1096.9 m/z: 49.281614

8)

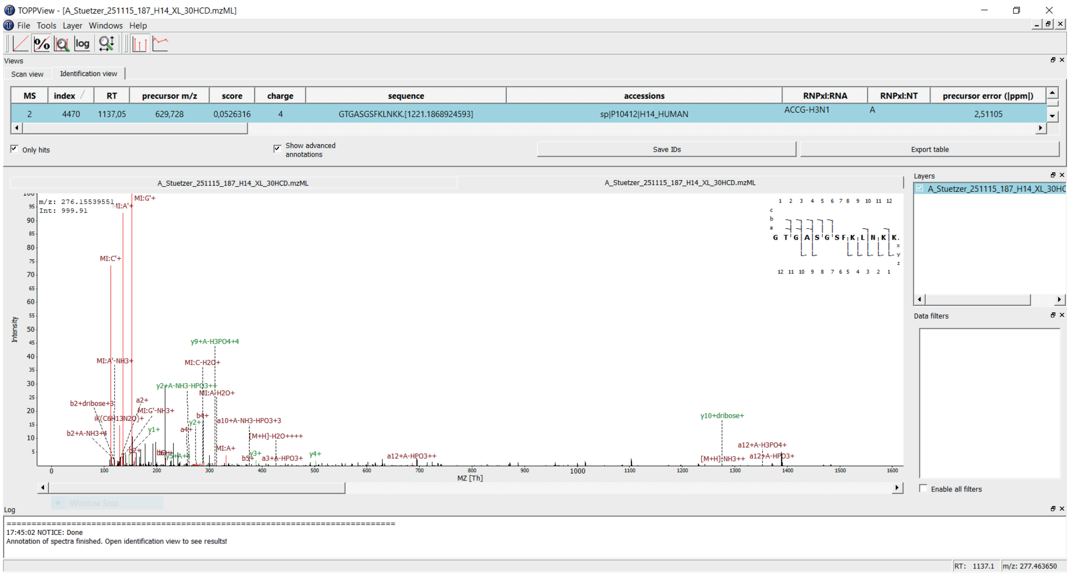
TOPPView - [A_Stuetzer_251115_187_H14_XL_30HCD.mzML]
File Tools Layer Windows Help
Scan view Identification view
MS | index | RT | precursor m/z | score | charge | sequence | accessions | RNPxl:RNA | RNPxl:NT | precursor error (|ppm|)
2 | 4470 | 1137,05 | 629,728 | 0,0526316 | 4 | GTGASGSFKLNKK.[1221.1868924593] | sp|P10412|H14_HUMAN | ACCG-H3N1 | A | 2,51105
Only hits
Show advanced annotations
Save IDs
Export table
A_Stuetzer_251115_187_H14_XL_30HCD.mzML
m/z: 276.15539551
Int: 999.91
MZ [Th]
Intensity
Layers
Data filters
Enable all filters
Log
17:45:02 NOTICE: Done
Annotation of spectra finished. Open identification view to see results!
RT: 1137.1 m/z: 277.463650

9)

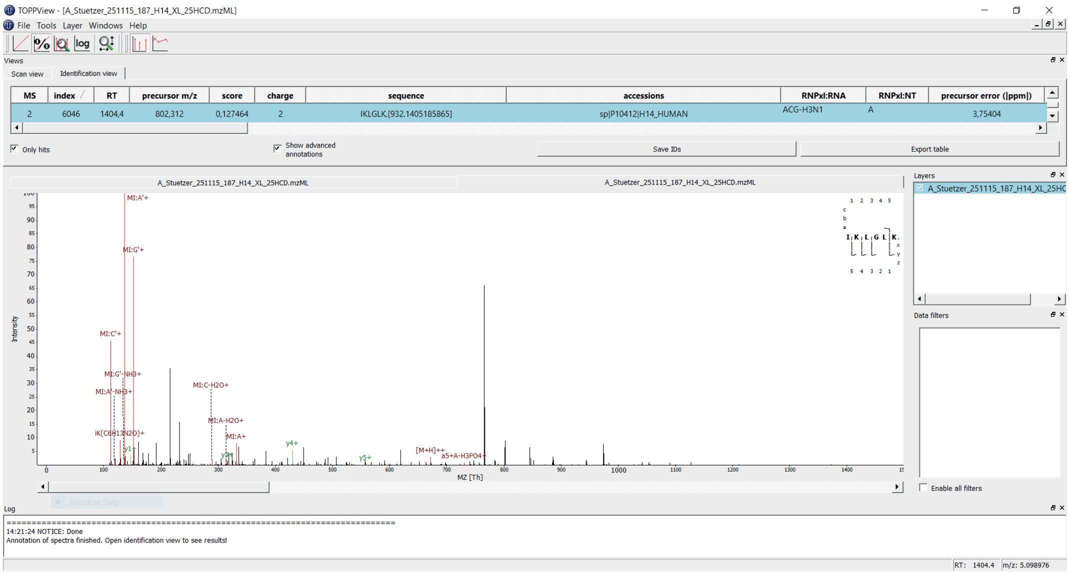
TOPPView - [A_Stuetzer_251115_187_H14_XL_25HCD.mzML]
File Tools Layer Windows Help
Scan view Identification view
MS | index | RT | precursor m/z | score | charge | sequence | accessions | RNPxl:RNA | RNPxl:NT | precursor error (|ppm|)
2 | 6046 | 1404,4 | 802,312 | 0,127464 | 2 | IKLGLK.[932.1405185865] | sp|P10412|H14_HUMAN | ACG-H3N1 | A | 3,75404
Only hits
Show advanced annotations
Save IDs
Export table
A_Stuetzer_251115_187_H14_XL_25HCD.mzML
MZ [Th]
Intensity
Layers
Data filters
Enable all filters
Log
14:21:24 NOTICE: Done
Annotation of spectra finished. Open identification view to see results!
RT: 1404.4 m/z: 5.098976

10)

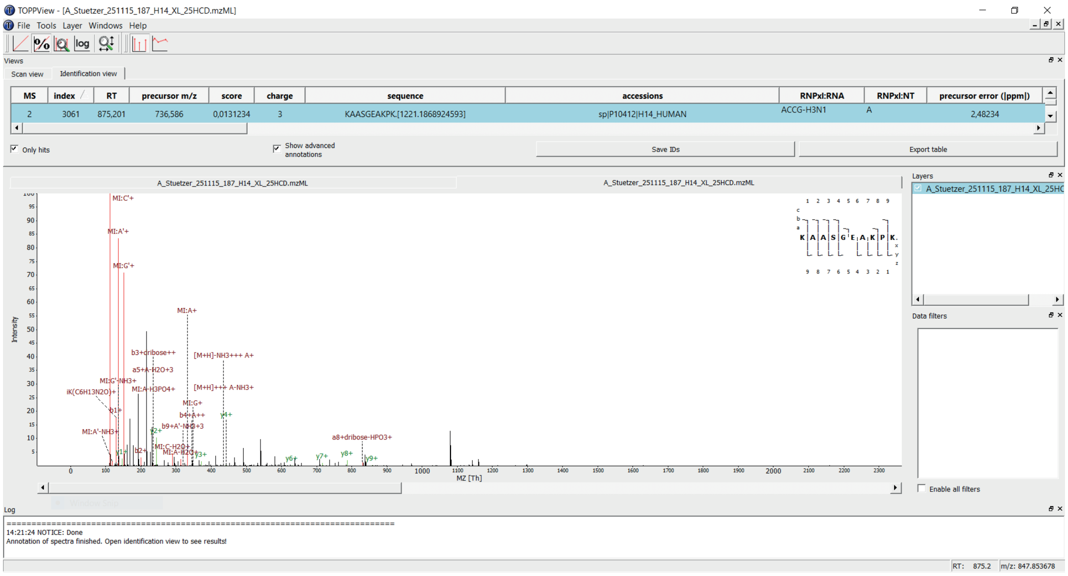
TOPPView - [A_Stuetzer_251115_187_H14_XL_25HCD.mzML]
MS | index | RT | precursor m/z | score | charge | sequence | accessions | RNPxl:RNA | RNPxl:NT | precursor error (|ppm|)
2 | 3061 | 875,201 | 736,586 | 0,0131234 | 3 | KAASGEAKPK.[1221.1868924593] | sp|P10412|H14_HUMAN | ACCG-H3N1 | A | 2,48234
A_Stuetzer_251115_187_H14_XL_25HCD.mzML
Intensity
MZ [Th]
14:21:24 NOTICE: Done
Annotation of spectra finished. Open identification view to see results!

11)

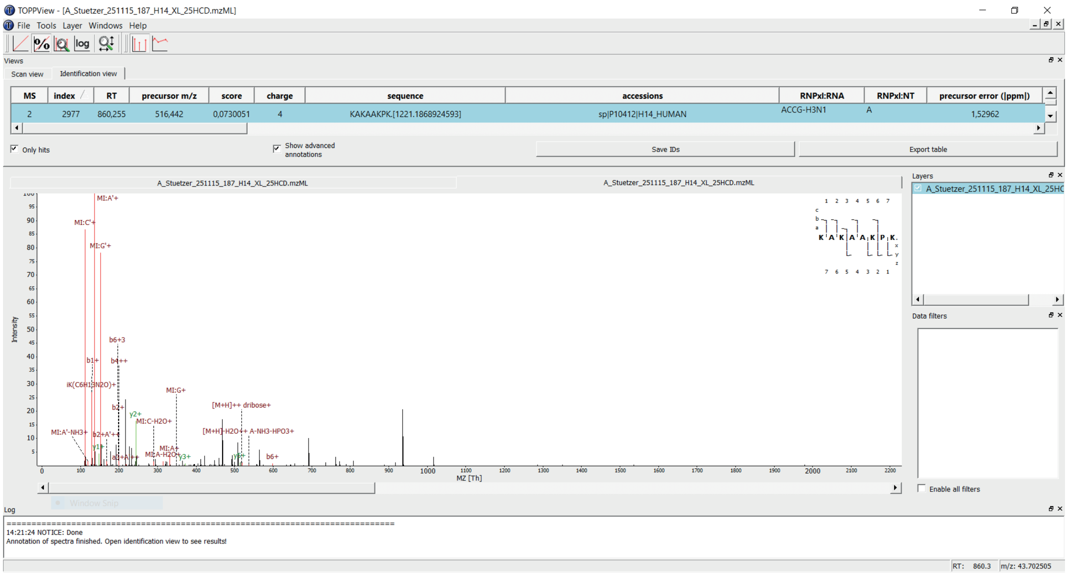
TOPPView - [A_Stuetzer_251115_187_H14_XL_25HCD.mzML]
MS | index | RT | precursor m/z | score | charge | sequence | accessions | RNPxl:RNA | RNPxl:NT | precursor error (|ppm|)
2 | 2977 | 860,255 | 516,442 | 0,0730051 | 4 | KAKAAKPK.[1221.1868924593] | sp|P10412|H14_HUMAN | ACCG-H3N1 | A | 1,52962
A_Stuetzer_251115_187_H14_XL_25HCD.mzML
Intensity
MZ [Th]
14:21:24 NOTICE: Done
Annotation of spectra finished. Open identification view to see results!

12)

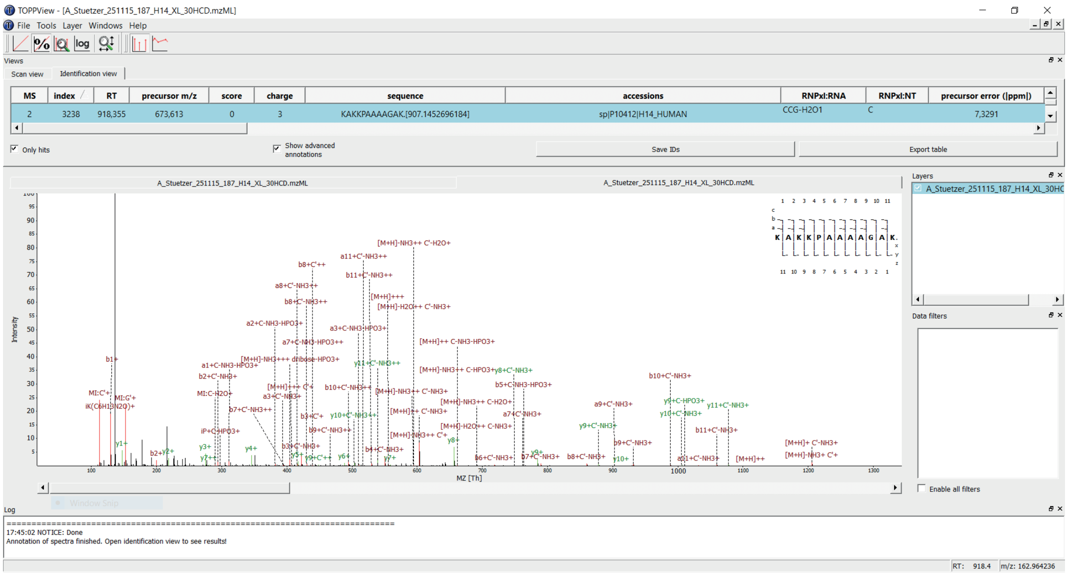
TOPPView - [A_Stuetzer_251115_187_H14_XL_30HCD.mzML]
MS | index | RT | precursor m/z | score | charge | sequence | accessions | RNPxl:RNA | RNPxl:NT | precursor error (|ppm|)
2 | 3238 | 918,355 | 673,613 | 0 | 3 | KAKKPAAAAGAK.[907.1452696184] | sp|P10412|H14_HUMAN | CCG-H2O1 | C | 7,3291
A_Stuetzer_251115_187_H14_XL_30HCD.mzML
Intensity
MZ [Th]
17:45:02 NOTICE: Done
Annotation of spectra finished. Open identification view to see results!

13)

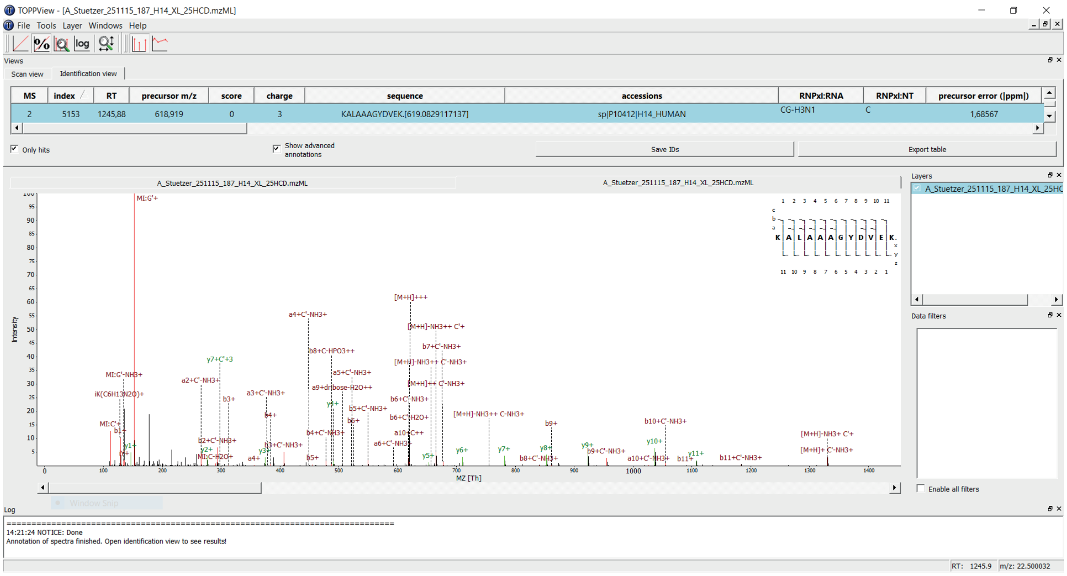
TOPPView - [A_Stuetzer_251115_187_H14_XL_25HCD.mzML]
MS | index | RT | precursor m/z | score | charge | sequence | accessions | RNPxl:RNA | RNPxl:NT | precursor error (|ppm|)
2 | 5153 | 1245,88 | 618,919 | 0 | 3 | KALAAAGYDVEK.[619.0829117137] | sp|P10412|H14_HUMAN | CG-H3N1 | C | 1,68567
14:21:24 NOTICE: Done
Annotation of spectra finished. Open identification view to see results!

14)

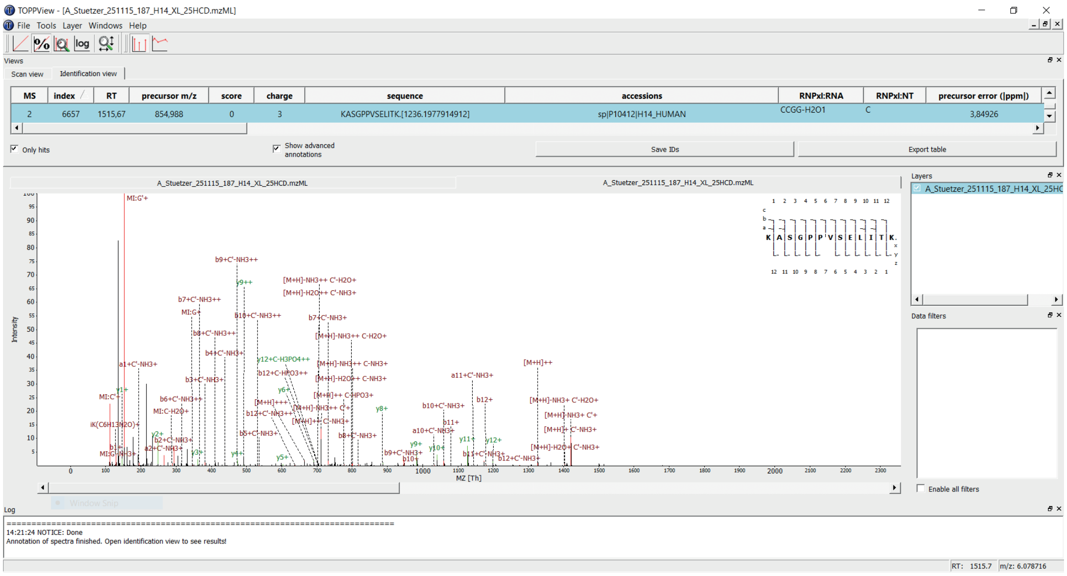
TOPPView - [A_Stuetzer_251115_187_H14_XL_25HCD.mzML]
MS | index | RT | precursor m/z | score | charge | sequence | accessions | RNPxl:RNA | RNPxl:NT | precursor error (|ppm|)
2 | 6657 | 1515,67 | 854,988 | 0 | 3 | KASGPPVSELITK.[1236.1977914912] | sp|P10412|H14_HUMAN | CCGG-H2O1 | C | 3,84926
14:21:24 NOTICE: Done
Annotation of spectra finished. Open identification view to see results!

15)

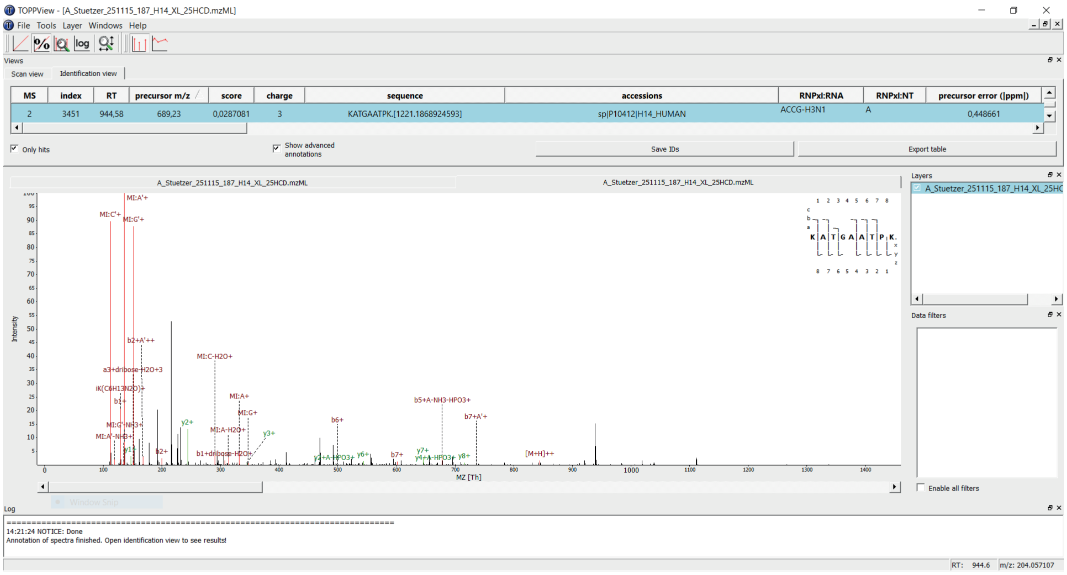
TOPPView - [A_Stuetzer_251115_187_H14_XL_25HCD.mzML]
MS | index | RT | precursor m/z | score | charge | sequence | accessions | RNPxl:RNA | RNPxl:NT | precursor error (|ppm|)
2 | 3451 | 944,58 | 689,23 | 0,0287081 | 3 | KATGAATPK.[1221.1868924593] | sp|P10412|H14_HUMAN | ACCG-H3N1 | A | 0,448661
14:21:24 NOTICE: Done
Annotation of spectra finished. Open identification view to see results!

16)

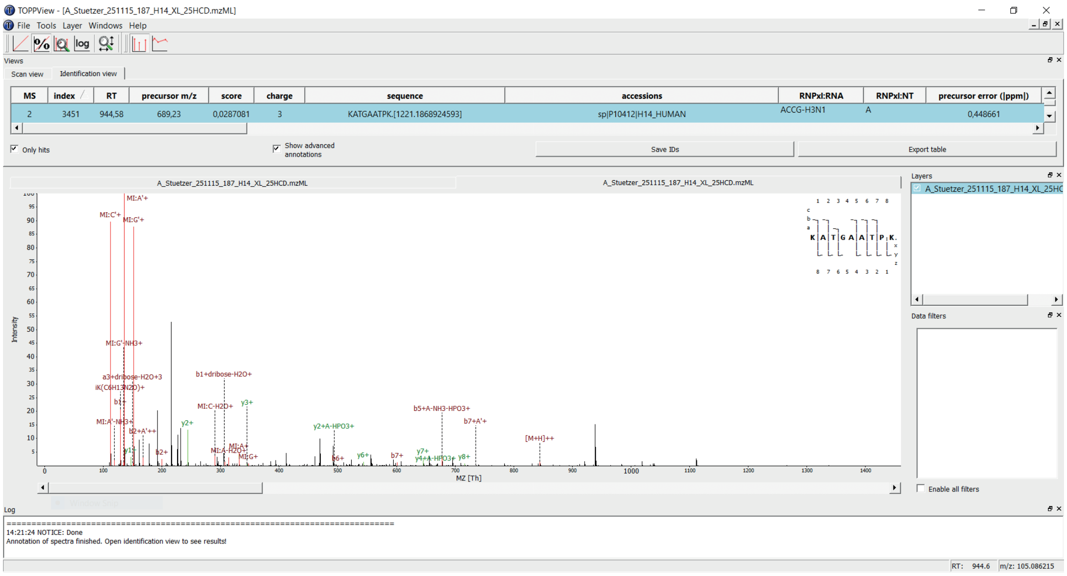
TOPPView - [A_Stuetzer_251115_187_H14_XL_25HCD.mzML]
MS | index | RT | precursor m/z | score | charge | sequence | accessions | RNPxl:RNA | RNPxl:NT | precursor error (|ppm|)
2 | 3451 | 944,58 | 689,23 | 0,0287081 | 3 | KATGAATPK.[1221.1868924593] | sp|P10412|H14_HUMAN | ACCG-H3N1 | A | 0,448661
A_Stuetzer_251115_187_H14_XL_25HCD.mzML
MZ [Th]
Intensity

17)

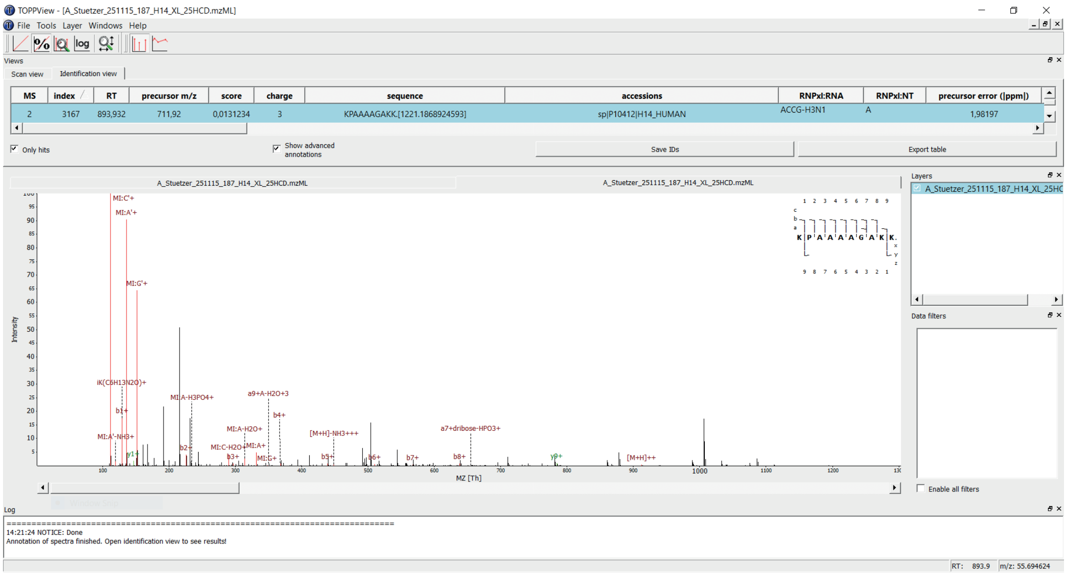
TOPPView - [A_Stuetzer_251115_187_H14_XL_25HCD.mzML]
MS | index | RT | precursor m/z | score | charge | sequence | accessions | RNPxl:RNA | RNPxl:NT | precursor error (|ppm|)
2 | 3167 | 893,932 | 711,92 | 0,0131234 | 3 | KPAAAAGAKK.[1221.1868924593] | sp|P10412|H14_HUMAN | ACCG-H3N1 | A | 1,98197
A_Stuetzer_251115_187_H14_XL_25HCD.mzML
MZ [Th]
Intensity

18)

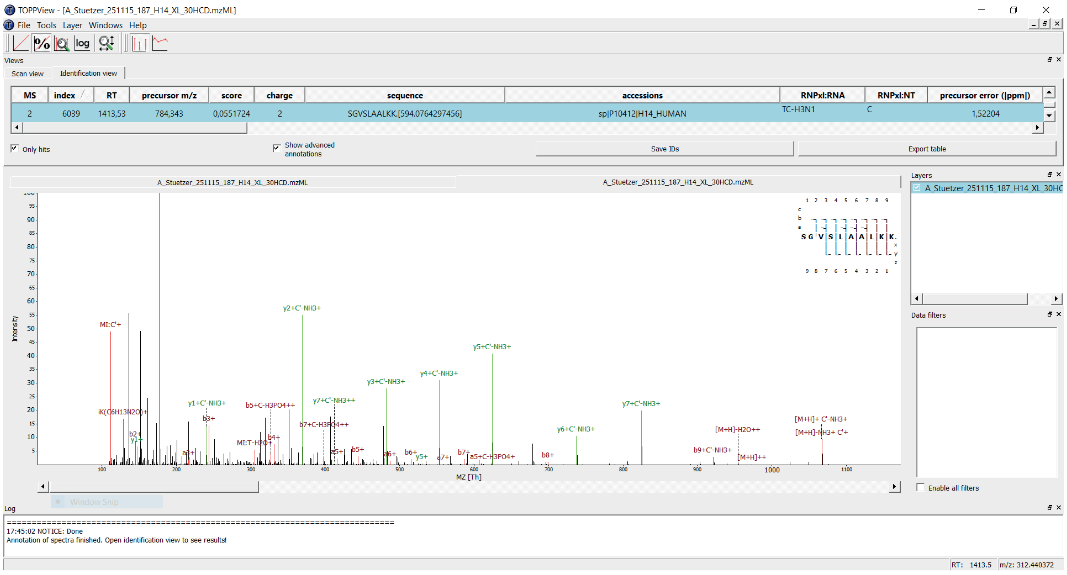
TOPPView - [A_Stuetzer_251115_187_H14_XL_30HCD.mzML]
MS | index | RT | precursor m/z | score | charge | sequence | accessions | RNPxl:RNA | RNPxl:NT | precursor error (|ppm|)
2 | 6039 | 1413,53 | 784,343 | 0,0551724 | 2 | SGVSLAALKK.[594.0764297456] | sp|P10412|H14_HUMAN | TC-H3N1 | C | 1,52204
A_Stuetzer_251115_187_H14_XL_30HCD.mzML
MZ [Th]
Intensity

19)

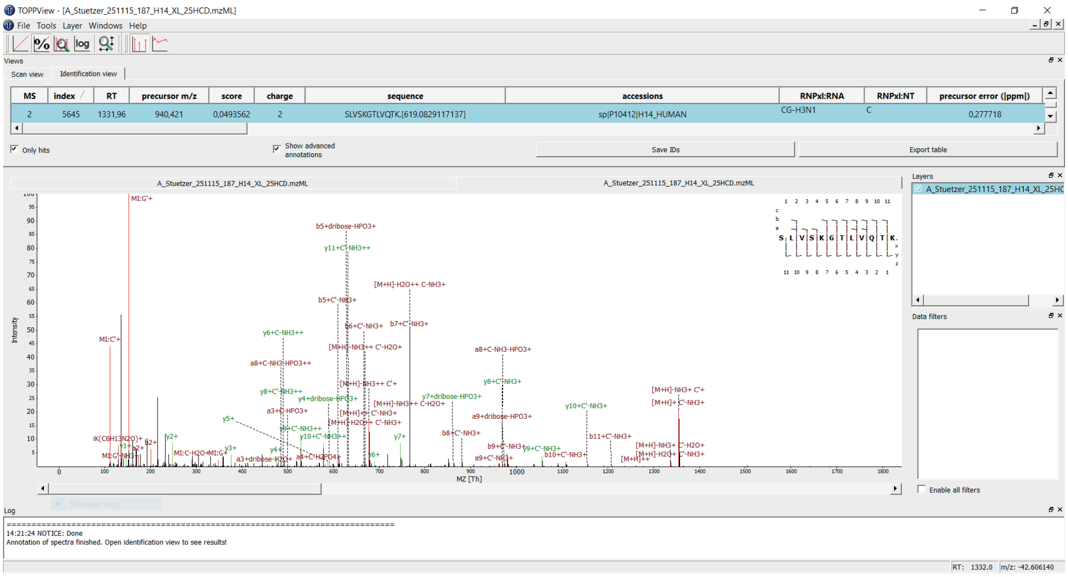
TOPPView - [A_Stuetzer_251115_187_H14_XL_25HCD.mzML]
File Tools Layer Windows Help
Views
Scan view
Identification view
MS | index | RT | precursor m/z | score | charge | sequence | accessions | RNPxl:RNA | RNPxl:NT | precursor error (|ppm|)
2 | 5645 | 1331,96 | 940,421 | 0,0493562 | 2 | SLVSKGTLVQTK.[619.0829117137] | sp|P10412|H14_HUMAN | CG-H3N1 | C | 0,277718
Only hits
Show advanced annotations
Save IDs
Export table
A_Stuetzer_251115_187_H14_XL_25HCD.mzML
Layers
Data filters
Enable all filters
Intensity
MZ [Th]
Log
14:21:24 NOTICE: Done
Annotation of spectra finished. Open identification view to see results!
RT: 1332.0
m/z: -42.606140

Linker histone H5

protein	peptide (aa)	cross-link site (aa - base/sugar)	DNA	m/z $_{exp}$	z	cross-link MW $_{exp}$	cross-link MW $_{calc}$	peptide MW $_{calc}$	DNA MW $_{calc}$	Δ ppm
**H5**	SAS**H**PTYSEMIAAAIR (22-37)	His25 - deoxyrib.	CGG+C5H7O5P	950.0063	3	2846.9954	2846.9947	1703.8296	1143.1651	0.27
		His25 - deoxyrib.	ACT+C5H7O5P	936.3387	3	2805.9926	2805.9933	1703.8296	1102.1637	-0.22
		His25 - deoxyrib.	C+C5H7O5P	1095.4534	2	2188.8912	2188.8896	1703.8296	485.0600	0.72
		His25 - deoxyrib.	A-C5H5N5 (=C5H9O6P)	950.9295	2	1899.8434	1899.8433	1703.8296	196.0137	0.03
**H5**	VG**H**NADLQIK (60-69)	His62 - deoxyrib.	AT	577.2418	3	1728.7019	1728.7017	1703.8296	635.1142	0.12
		His62 - deoxyrib.	CC+C5H7O5P	623.5733	3	1867.6964	1867.6938	1703.8296	774.1063	1.40
		His62 - deoxyrib.	ACC+C5H7O5P	727.9250	3	2180.7515	2180.7515	1703.8296	1087.1640	0.03
		His62 - deoxyrib.	GT+C5H7O5P	962.3581	2	1922.7006	1922.6997	1703.8296	829.1122	0.43

Annotated MS/MS spectra of linker histone H5

1) Histone H5, aa 60-69

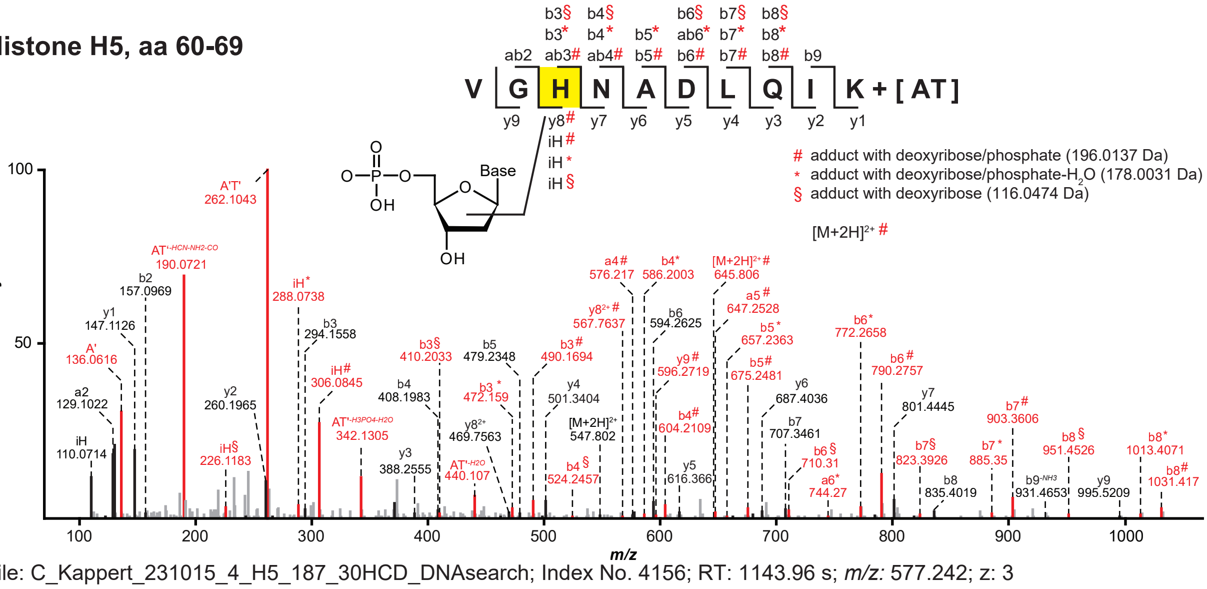

File: C_Kappert_231015_4_H5_187_30HCD_DNAsearch; Index No. 4156; RT: 1143.96 s; *m/z:* 577.242; z: 3

2) Histone H5, aa 22-37

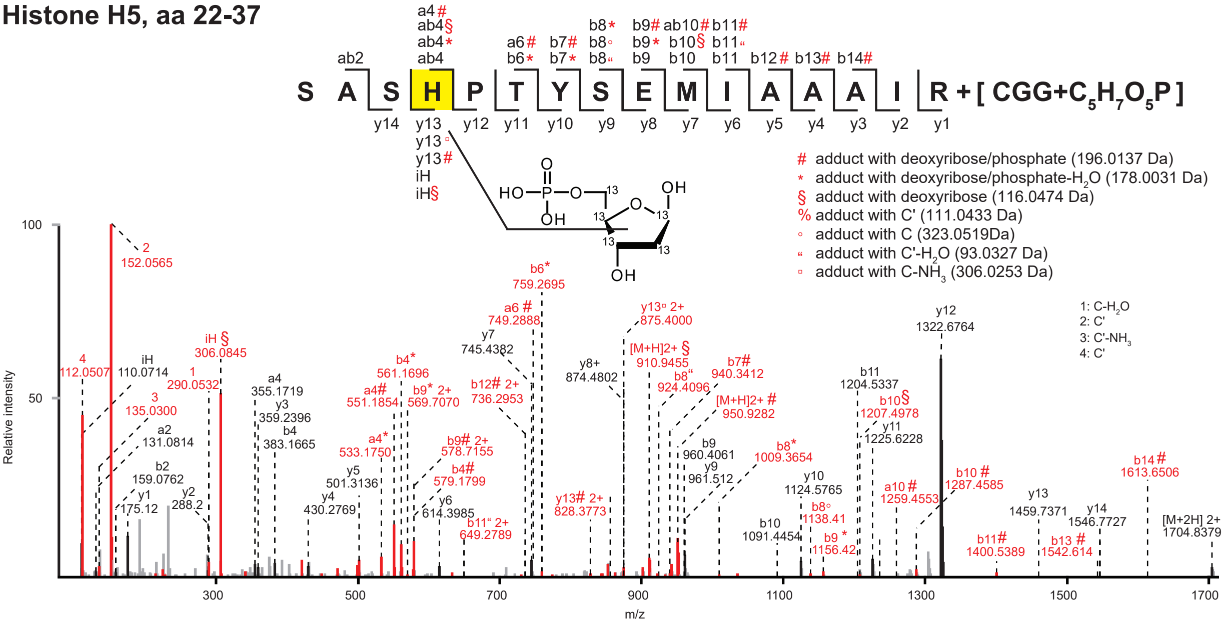

File: C_Kappert_231015_4_H5_187_30HCD_DNAsearch; Index No. 6710; RT: 1609.52 s; *m/z:* 950.006; z: 3

Histone H3

protein	peptide (aa)	cross-link site (aa - base/sugar)	DNA	m/z_{exp}	z	cross-link MW_{exp}	cross-link MW_{calc}	peptide MW_{calc}	DNA MW_{calc}	Δ ppm
**H3**	KQLAT**K**AAR (18-26)	Lys23 - C	ACG-NH3	640.2553	3	1917.7424	1917.7432	985.6027	932.14053	-0.41
		Lys23 - C	CT-NH3	790.8479	2	1579.6802	1579.6791	985.6027	594.07639	0.69
		Lys23 - C	CCGT-NH3	733.6006	3	2197.7783	2197.7779	985.6027	1212.1753	0.17
		Lys23 - C	CCCG-NH3	728.6011	3	2182.7798	2182.7782	985.6027	1197.1756	0.72
**H3**	**K**SAPATGGVK (27-36)	Lys27 - C	ACG-NH3	616.5604	3	1846.6577	1846.6585	914.5180	932.14053	-0.42
		Lys27 - C	AC-NH3	506.8767	3	1517.6066	1517.6060	914.5180	603.08799	0.43
		Lys27 - C	CT-NH3	755.3050	2	1508.5944	1508.5944	914.5180	594.07639	-0.01
		Lys27 - C	CCG-NH3	608.5570	3	1822.6475	1822.6472	914.5180	908.12923	0.18
**H3**	**Y**RPGTVALR (41-49)	Tyr41 - T	AT	556.5752	3	1666.7021	1666.7013	1031.5871	635.1142	0.49
		Tyr41 - T	TT	829.8544	2	1657.6932	1657.6897	1031.5871	626.1026	2.07
		Tyr41 - T	AATT-HPO3	551.9673	4	2203.8379	2203.8387	1031.5871	1172.2516	-0.35
		Tyr41 - T	ACGT	762.6077	3	2284.7996	2284.8002	1031.5871	1253.2131	-0.25
**H3**	YQ**K**STELLIR (54-63)	Lys56 - C	ACT-NH3	719.9533	3	2156.8364	2156.8365	1249.7025	907.13403	-0.04
		Lys56 - C	CT-NH3	922.8987	2	1843.7818	1843.7789	1249.7025	594.07639	1.56
		Lys56 - C	ACCG-NH3	824.6379	3	2470.8902	2470.8893	1249.7025	1221.1869	0.36
		Lys56 - C	CG-NH3	935.4019	2	1868.7882	1868.7854	1249.7025	619.08289	1.49
**H3**	**K**LPFQR (64-69)	Lys64 -C	AC-NH3	696.2867	2	1390.5578	1390.5579	787.4699	603.0880	-0.08
		Lys64 -C	CCT	563.5478	3	1687.6199	1687.6192	787.4699	900.14928	0.46
		Lys64 -C	CT-NH3	691.7808	2	1381.5460	1381.5463	787.4699	594.07639	-0.23
		Lys64 -C	ACCG-NH3	670.5587	3	2008.6526	2008.6567	787.4699	1221.1869	-2.05
**H3**	EIAQDF**K**TDLR (74-83)	Lys79 - C	CT-NH3	965.3860	2	1928.7564	1928.7589	1334.6825	594.07639	-1.30
		Lys79 - C	CCCG-NH3	844.9614	3	2531.8607	2531.8580	1334.6825	1197.1756	1.06
		Lys79 - C	ACGT-NH3	857.9668	3	2570.8769	2570.8690	1334.6825	1236.1866	3.07
		Lys79 - C	CCG-H2O	748.2809	3	2241.8192	2241.8277	1334.6825	907.1452	-3.78
**H3**	VTIMP**K**DIQLAR (117-128)	Lys122 - C	CCG-NH3	764.9806	3	2291.9183	2291.9195	1383.7903	908.1292	-0.51
		Lys122 - C	CCTT	863.6687	3	2587.9826	2587.9856	1383.7903	1204.1953	-1.15
		Lys122 - C	ACCC-NH3	856.0003	3	2564.9774	2564.9709	1383.7903	1181.1807	2.53
		Lys122 - C	CCCG-NH3	861.3287	3	2580.9626	2580.9658	1383.7903	1197.1756	-1.25

Annotated MS/MS spectra of core histone H3

1)

Histone H3, aa 18-26

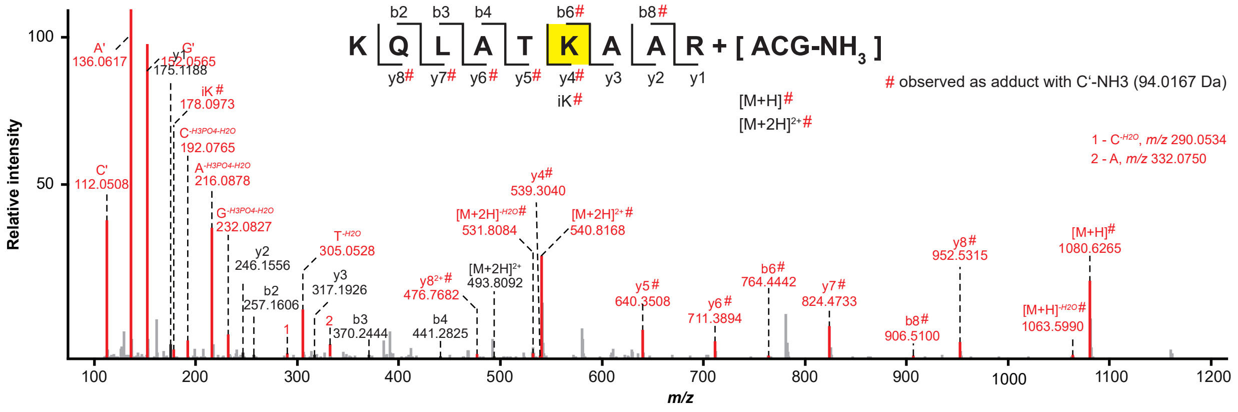

File: A_Stuetzer_180816_12mer_H14_UV_25mM; Index No. 6077; RT: 1162.38 s; *m/z:* 640.256; z: 3

2)

m/z: 934.844; z: 2; score: 27.5404

Histone H3, aa 27-36

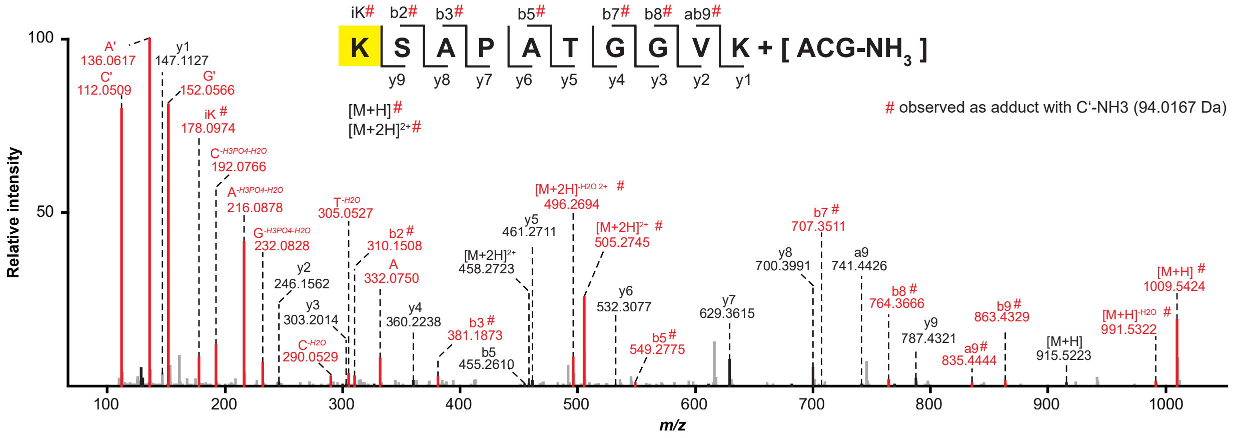

File: A_Stuetzer_180816_12mer_H14_UV_25mM; Index No. 6069; RT: 1161.26 s; *m/z:* 616.56; z: 3

3) Histone H3, aa 41-49

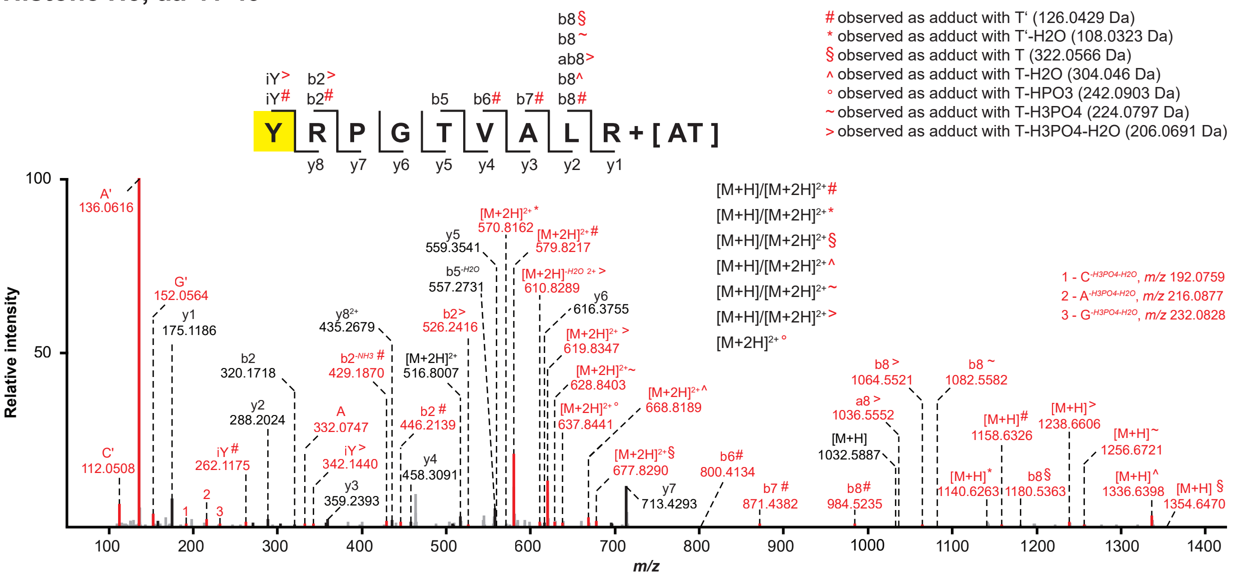

File: A_Stuetzer_180816_12mer_UV_150mM; Index No. 8469; RT: 1551.76 s; m/z: 556.575; z: 3

4) Histone H3, aa 54-63

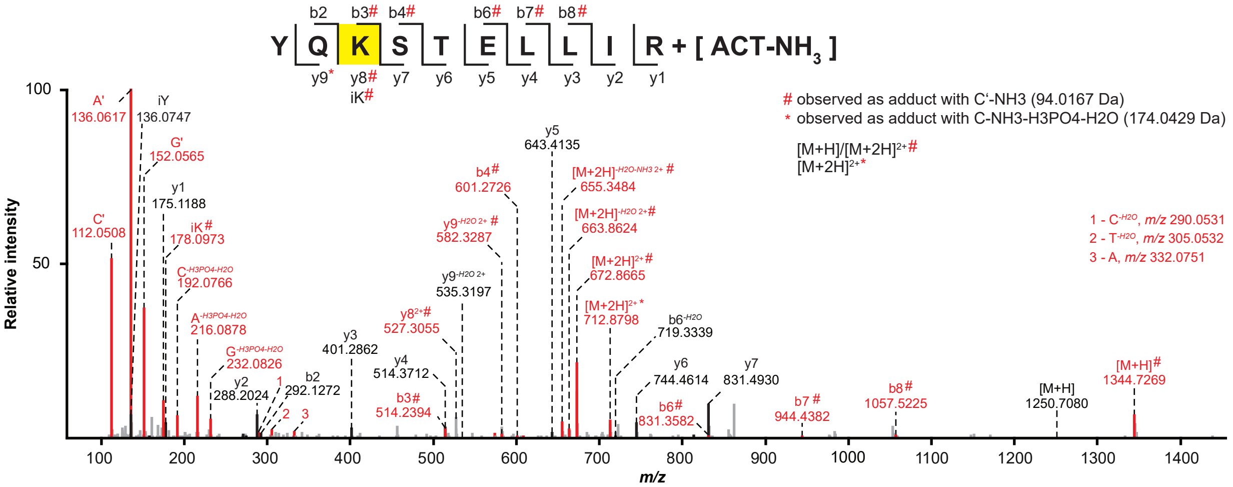

File: A_Stuetzer_180816_12mer_UV_25mM; Index No. 12150; RT: 2092.87 s; m/z: 719.953; z: 3

5) Histone H3, aa 64-69

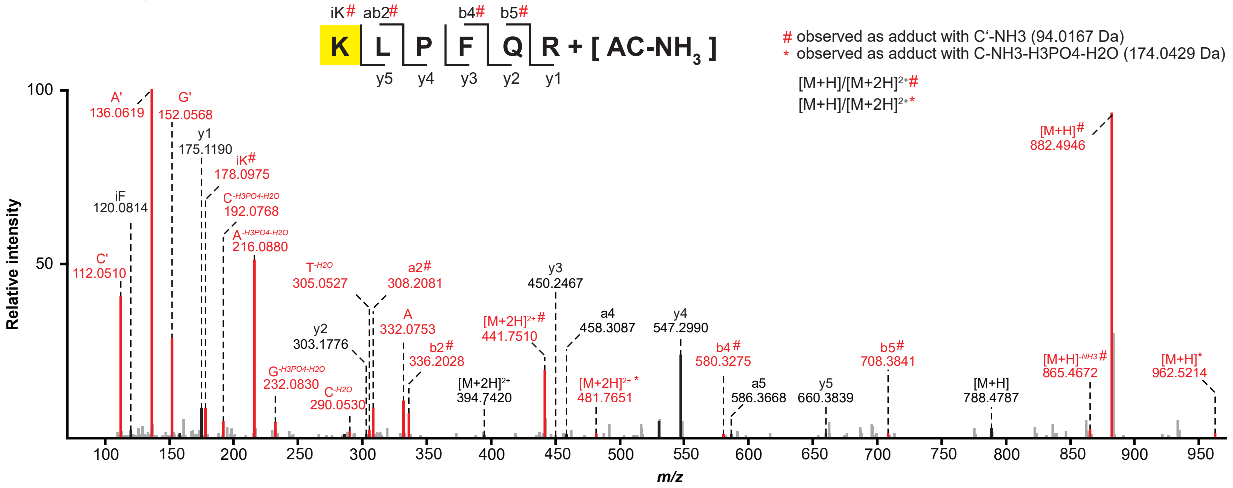

File: A_Stuetzer_180816_12mer_UV_150mM; Index No. 10471; RT: 1849.7 s; m/z: 696.287; z: 2

6)

Histone H3, aa 73-83

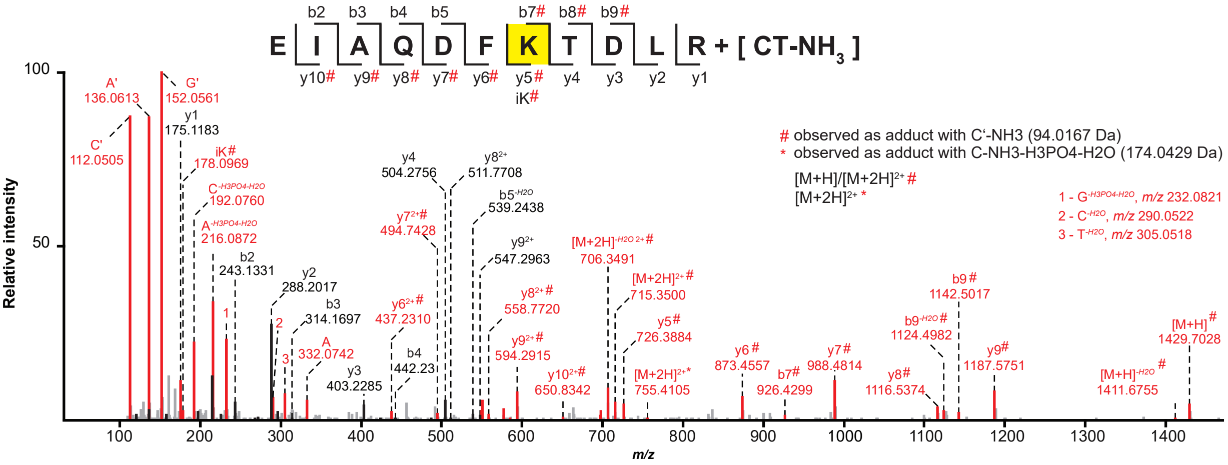

File: A_Stuetzer_180816_12mer_H14_UV_150mM; Index No. 12808; RT: s; m/z: ; z:

7)

Histone H3, aa 117-128

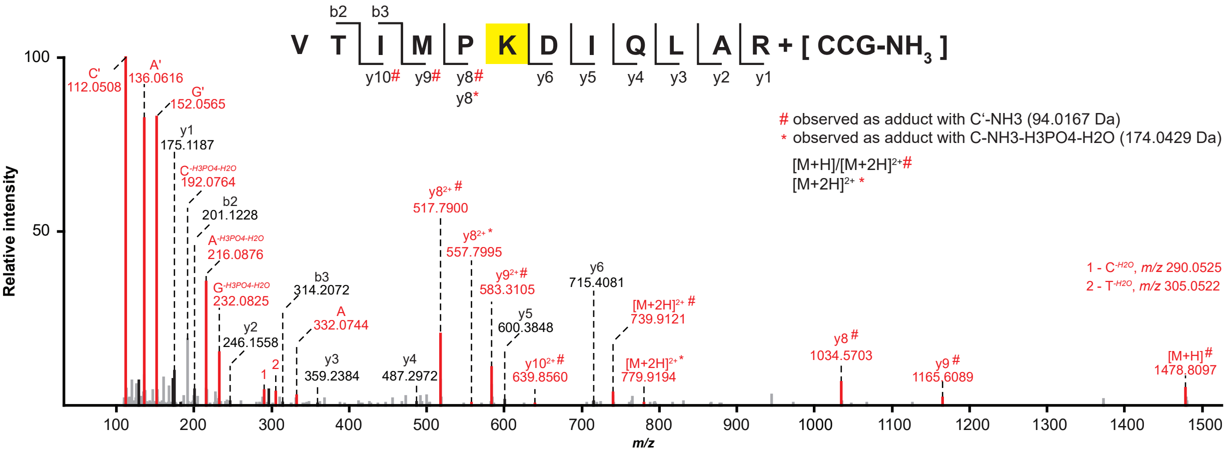

File: A_Stuetzer_180816_12mer_H14_UV_150mM; Index No. 13760; RT: 2335.68 s; m/z: 764.961; z: 3

Histone H2A

protein	peptide (aa)	cross-link site (aa - base/sugar)	DNA	*m/z* exp	z	cross-link MW exp	cross-link MW calc	peptide MW calc	DNA MW calc	Δ ppm
**H2A**	AKTRSSRAGLQFPVGR (14-29)	(n/d) - T	AACT	742.8054	4	2967.1903	2967.1877	1729.9695	1237.2182	0.88
		(n/d) - T	CCGT	987.4012	3	2959.1801	2959.1713	1729.9695	1229.2018	2.98
		(n/d) - T	CGT	891.0502	3	2670.1271	2670.1250	1729.9695	940.1555	0.81
**H2A**	**H**LQLAVR (82-88)	His82 - deoxyrib.	CCT	579.5580	3	1735.6505	1735.6516	835.5023	900.1493	-0.59
		His82 - deoxyrib.	AAA	598.5699	3	1792.6862	1792.6858	835.5023	957.1835	0.26
		His82 - deoxyrib.	AT	491.2130	3	1470.6155	1470.6165	835.5023	635.1142	-0.67
		His82 - deoxyrib.	ACT	587.5614	3	1759.6607	1759.6629	835.5023	924.1606	-1.21
**H2A**	NDEELN**K**LLGR (89-99)	Lys95 - C	CCCG-NH3	833.2941	3	2496.8588	2496.8533	1299.6778	1197.1756	2.19
		Lys95 - C	CCC-NH3	723.6083	3	2167.8014	2167.8008	1299.6778	868.1230	0.29
		Lys95 - C	ACCG-NH3	841.2941	3	2520.8588	2520.8646	1299.6778	1221.1869	-2.31
		Lys95 - C	ACCC-NH3	827.9618	3	2480.8619	2480.8584	1299.6778	1181.1807	1.40
**H2A**	VTIAQGGVLPNIQSVLLP**K**K (100-119)	Lys118 - C	AC-NH3	893.4549	3	2677.3412	2677.3389	2074.2509	603.0880	0.88
		Lys118 - C	ACC-NH3	989.8029	3	2966.3852	2966.3852	2074.2509	892.1343	0.01
		Lys118 - C	CCG-NH3	995.1349	3	2982.3812	2982.3801	2074.2509	908.1292	0.38
		Lys118 - C	CGT-NH3	1000.1380	3	2997.3905	2997.3798	2074.2509	923.1289	3.58

Annotated MS/MS spectra of core histone H2A

1) Histone H2A, aa 14-29

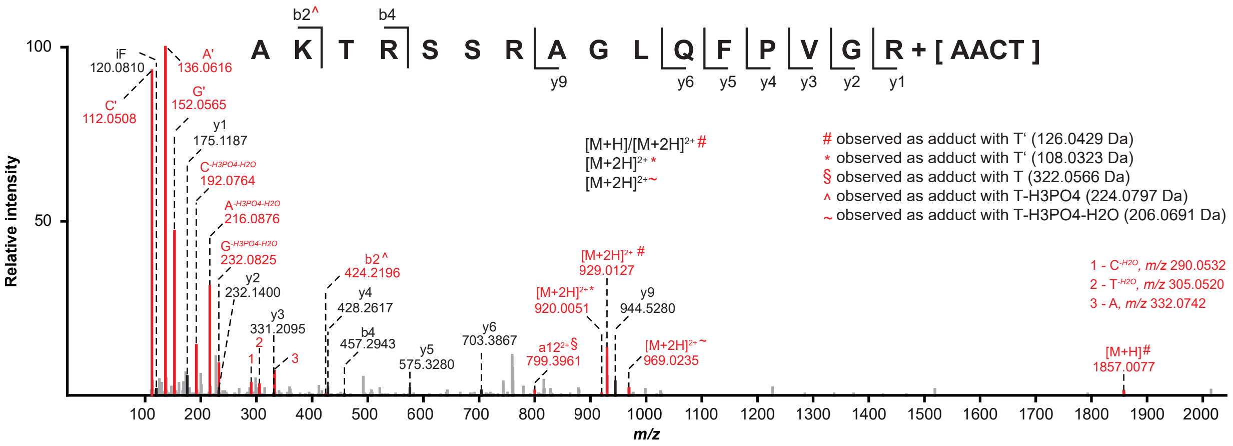

File: A_Stuetzer_180816_12mer_UV_25mM; Index No. 14124; RT: 2390.01 s; m/z: 743.307; z: 4

2) Histone H2A, aa 82-88

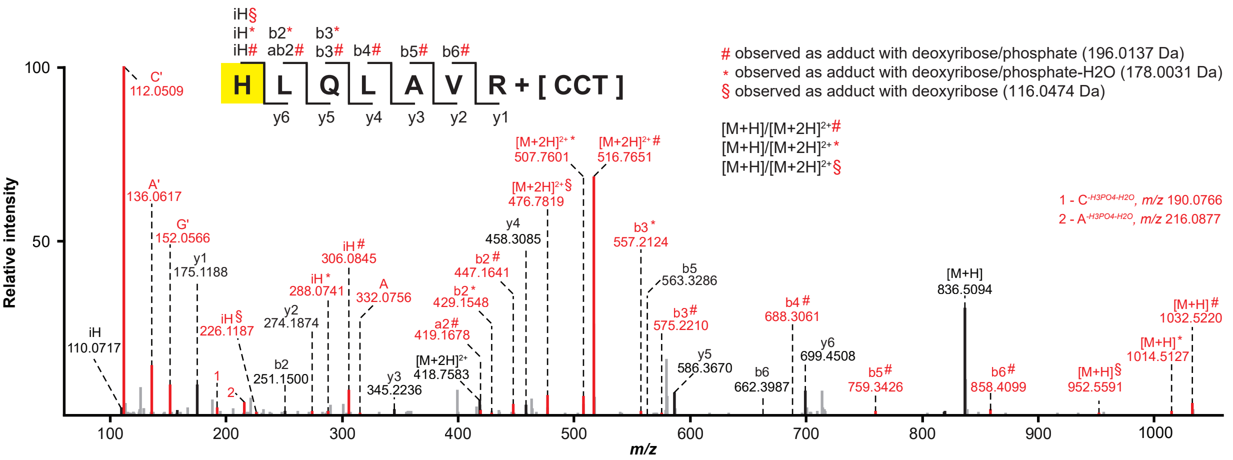

File: A_Stuetzer_180816_12mer_UV_25mM; Index No. 8662; RT: 1579.42 s; m/z: 579.559; z: 3

3) Histone H2A, aa 89-99

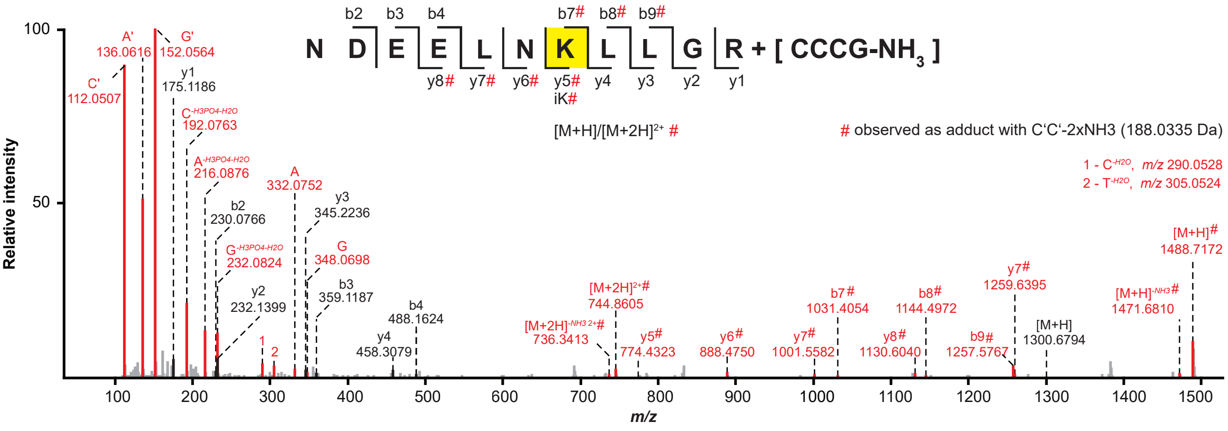

File: A_Stuetzer_180816_12mer_H14_UV_150mM; Index No. ; RT: s; m/z: 833.294; z: 3

4) Histone H2A, aa 100-119

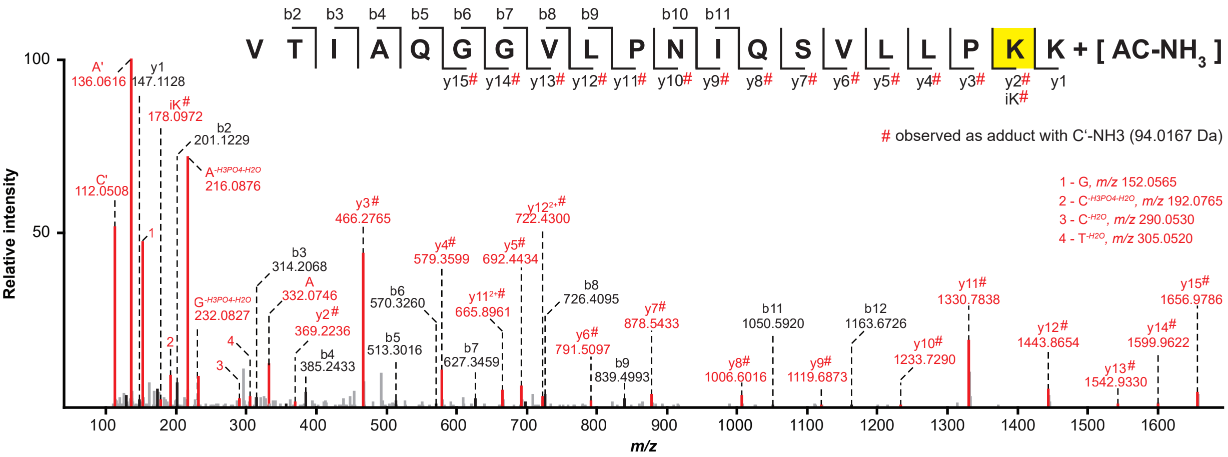

File: A_Stuetzer_180816_12mer_H14_UV_150mM; Index No. 16954; RT: 2821.18 s; m/z: 893.455; z: 3

Histone H2B

protein	peptide (aa)	cross-link site (aa - base/sugar)	DNA	m/z_{exp}	z	cross-link MW_{exp}	cross-link MW_{calc}	peptide MW_{calc}	DNA MW_{calc}	Δ ppm
**H2B**	ESYAIYVY**K**VLK (32-43)	Lys40 - C	CC-NH3	1027.9506	2	2053.8856	2053.8833	1474.8066	579.0767	1.11
		Lys40 - C	AC-NH3	693.6397	3	2077.8956	2077.8946	1474.8066	603.0880	0.51
		Lys40 - C	CCC-NH3	781.9844	3	2342.9297	2342.9296	1474.8066	868.1230	0.05
		Lys40 - C	CT-NH3	1035.4493	2	2068.8830	2068.8830	1474.8066	594.0764	-0.01
**H2B**	VL**K**QVHPDTGISSK (41-54)	Lys43 - C	AC-NH3	704.6492	3	2110.9241	2110.9233	1507.8353	603.0880	0.41
		Lys43 - C	CCGT-NH3	907.6777	3	2720.0096	2720.0105	1507.8353	1212.1753	-0.34
		Lys43 - C	ACC-NH3	800.9990	3	2399.9735	2399.9696	1507.8353	892.1343	1.63
		Lys43 - C	CCGG-NH3	916.0153	3	2745.0224	2745.0170	1507.8353	1237.1818	1.96
**H2B**	QV**H**PDTGISSK (44-54)	His46 - deoxyrib.	GGGG	834.9465	3	2501.8160	2501.8086	1167.5879	1334.2207	2.97
**H2B**	LLLPGELA**K**HAVSEGTK (97-113)	Lys105 - C	ACT-NH3	890.7191	3	2669.1338	2669.1324	1761.9984	907.1340	0.53
		Lys105 - C	CC-NH3	781.3661	3	2341.0748	2341.0751	1761.9984	579.0767	-0.10
		Lys105 - C	CCCC-NH3	974.0627	3	2919.1646	2919.1677	1761.9984	1157.1694	-1.07
		Lys105 - C	CG-NH3	794.7014	3	2381.0807	2381.0813	1761.9984	619.0829	-0.23
**H2B**	HAVSEGT**K**AVTK (106-117)	Lys113 - C	CCGG-NH3	822.2889	3	2463.8432	2463.8431	1226.6614	1237.1818	0.03
		Lys113 - C	CCCG-NH3	808.9543	3	2423.8394	2423.8369	1226.6614	1197.1756	1.02
		Lys113 - C	ACCG-NH3	816.9567	3	2447.8466	2447.8482	1226.6614	1221.1869	-0.66
		Lys113 - C	CCG-NH3	712.6044	3	2134.7897	2134.7906	1226.6614	908.1292	-0.41

Annotated MS/MS spectra of core histone H2B

1) Histone H2B, aa 32-43

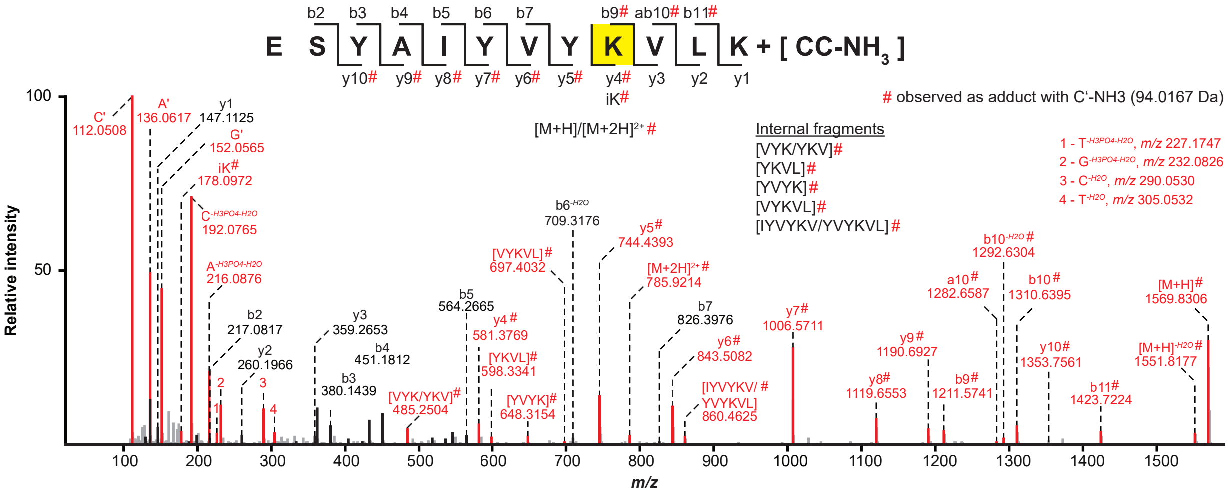

File: A_Stuetzer_180816_12mer_H14_UV_150mM; Index No. 16207; RT: 2705.19 s; m/z: 693.64; z: 3

2) Histone H2B, aa 41-54

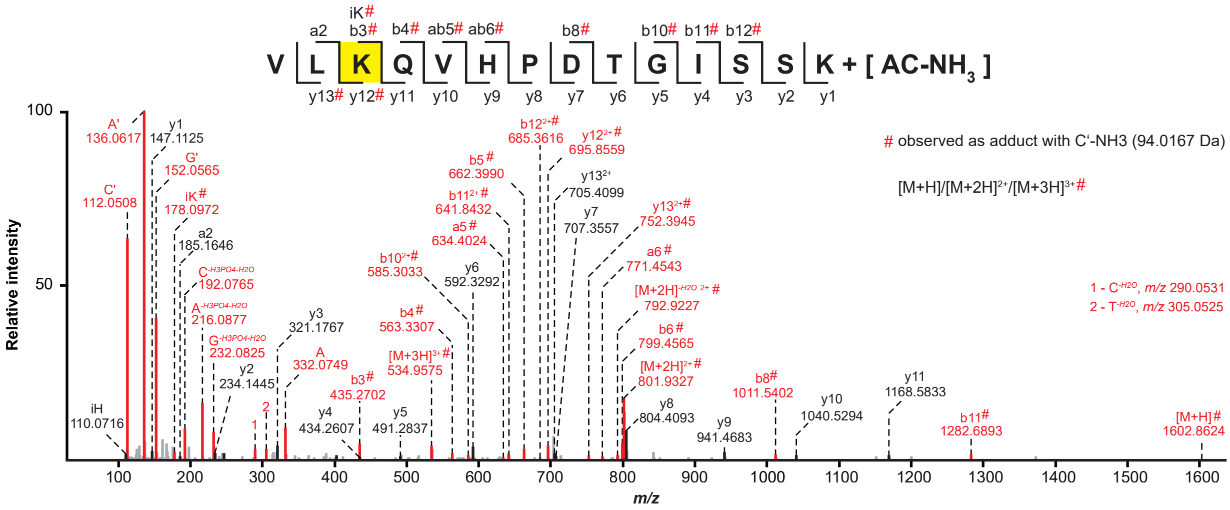

File: A_Stuetzer_180816_12mer_H14_UV_150mM; Index No. 7902; RT: 1465.69 s; m/z: 704.649; z: 3

3) Histone H2B, aa 44-54

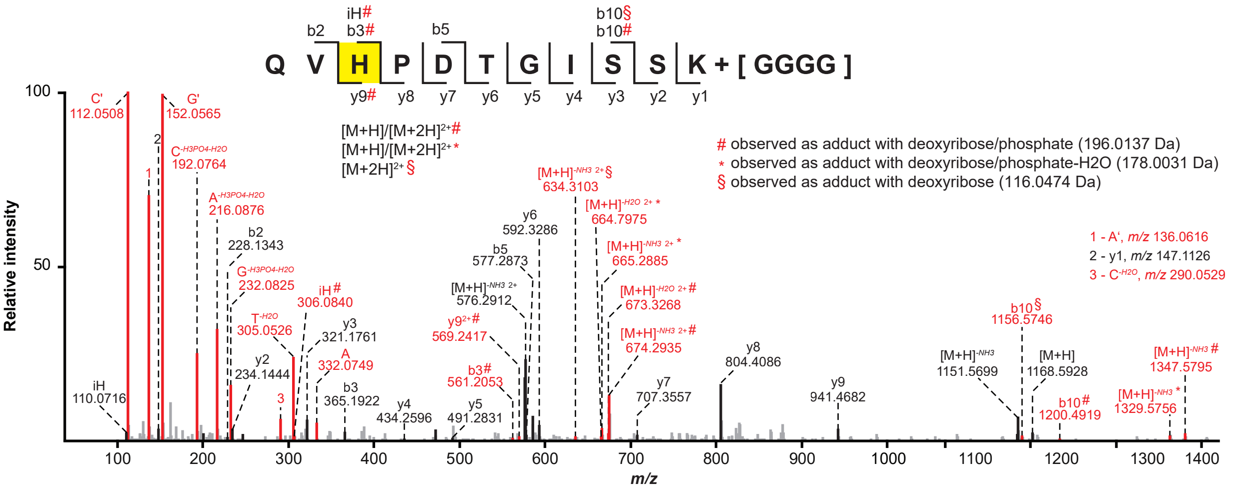

File: ; Index No. ; RT: s; m/z: 834.947; z: 3

4) Histone H2B, aa 97-113

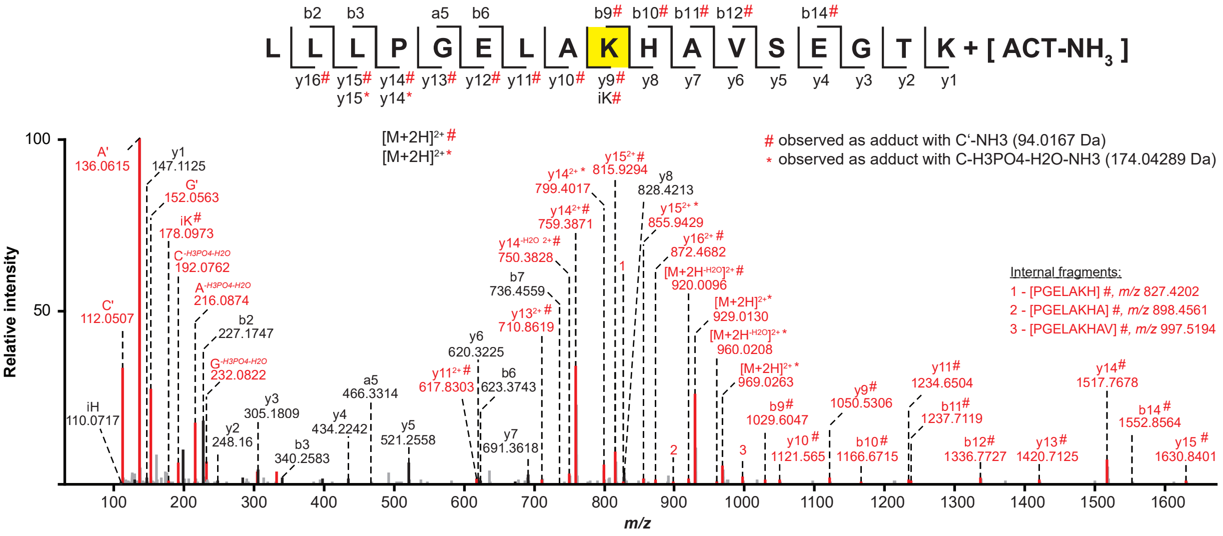

File: A_Stuetzer_180816_12mer_UV_25mM; Index No. 15016; RT: 2524.11 s; m/z: 890.719; z: 3

5) Histone H2B, aa 106-117

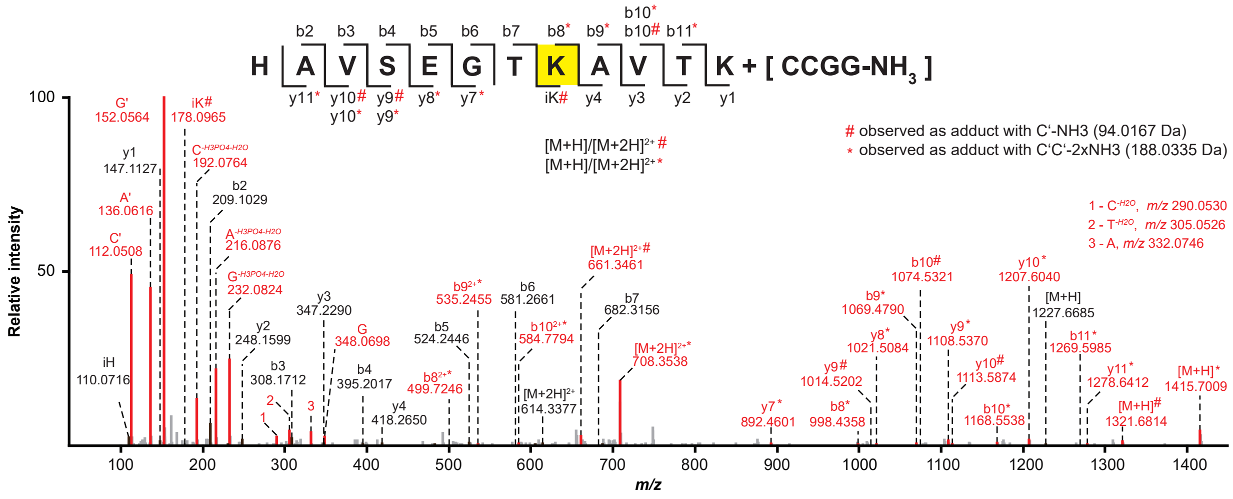

File: A_Stuetzer_180816_12mer_UV_25mM; Index No. ; RT: s; m/z: 822.289; z: 3

Histone H4

protein	peptide (aa)	cross-link site (aa - base/sugar)	DNA	m/z_{exp}	z	cross-link MW $_{exp}$	cross-link MW $_{calc}$	peptide MW $_{calc}$	DNA MW $_{calc}$	Δ ppm
**H4**	GG**K**GLGK (6-12)	Lys8 - C	ACC-NH3	503.5088	3	1507.5029	1507.5042	615.3699	892.1343	-0.85
		Lys8 - C	AC-NH3	407.1602	3	1218.4571	1218.4579	615.3699	603.0880	-0.61
		Lys8 - C	CCG-NH3	508.8409	3	1523.4992	1523.4991	615.3699	908.1292	0.08
		Lys8 - C	CT-NH3	605.7315	2	1209.4474	1209.4463	615.3699	594.0764	0.90
**H4**	GLG**K**GGAK (9-16)	Lys12 - C	ACG-NH3	540.5239	3	1618.5482	1618.5475	686.4070	932.1405	0.44
		Lys12 - C	ACC-NH3	527.1881	3	1578.5408	1578.5413	686.4070	892.1343	-0.30
		Lys12 - C	CCG-NH3	532.5201	3	1594.5368	1594.5362	686.4070	908.1292	0.39
		Lys12 - C	AC-NH3	430.8393	3	1289.4944	1289.4950	686.4070	603.0880	-0.42
**H4**	DNIQGIT**K**PAIR (24-35)	Lys31 - C	ACT-NH3	744.9653	3	2231.8724	2231.8798	1324.7458	907.1340	-3.31
		Lys31 - C	ACC-NH3	739.9674	3	2216.8787	2216.8801	1324.7458	892.1343	-0.62
		Lys31 - C	CCC-NH3	731.9644	3	2192.8697	2192.8688	1324.7458	868.1230	0.42
		Lys31 - C	CC-NH3	635.6160	3	1903.8245	1903.8225	1324.7458	579.0767	1.08
**H4**	DAVTYTE**H**AK (68-77)	His75 - deoxyrib.	CCTT-H2O	774.2497	3	2319.7256	2319.7195	1133.5348	1186.1848	2.63
		His75 - deoxyrib.	AAC-NH3	684.2335	3	2049.6770	2049.6804	1133.5348	916.1456	-1.65
**H4**	DAVTYTEHA**K**R (68-78)	Lys77 - C	ACC-NH3	728.2651	3	2181.7718	2181.7702	1289.6359	892.1343	0.74
		Lys77 - C	CCGT-NH3	834.9448	3	2501.8109	2501.8111	1289.6359	1212.1753	-0.09
		Lys77 - C	ACCT-NH3	829.6128	3	2485.8149	2485.8162	1289.6359	1196.1804	-0.53
		Lys77 - C	ACCG-NH3	837.9500	3	2510.8265	2510.8227	1289.6359	1221.1869	1.51
**H4**	**K**TVTAMDVVYALK (79-91)	Lys79 - C	AC-NH3	681.3000	3	2040.8765	2040.8776	1437.7896	603.0880	-0.51
		Lys79 - C	CCCC-NH3	865.9927	3	2594.9546	2594.9589	1437.7896	1157.1694	-1.66
		Lys79 - C	CCG-NH3	782.9805	3	2345.9180	2345.9188	1437.7896	908.1292	-0.33
		Lys79 - C	ACC-NH3	777.6490	3	2329.9235	2329.9239	1437.7896	892.1343	-0.16

Annotated MS/MS spectra of core histone H4

1)

Histone H4 aa 6-12

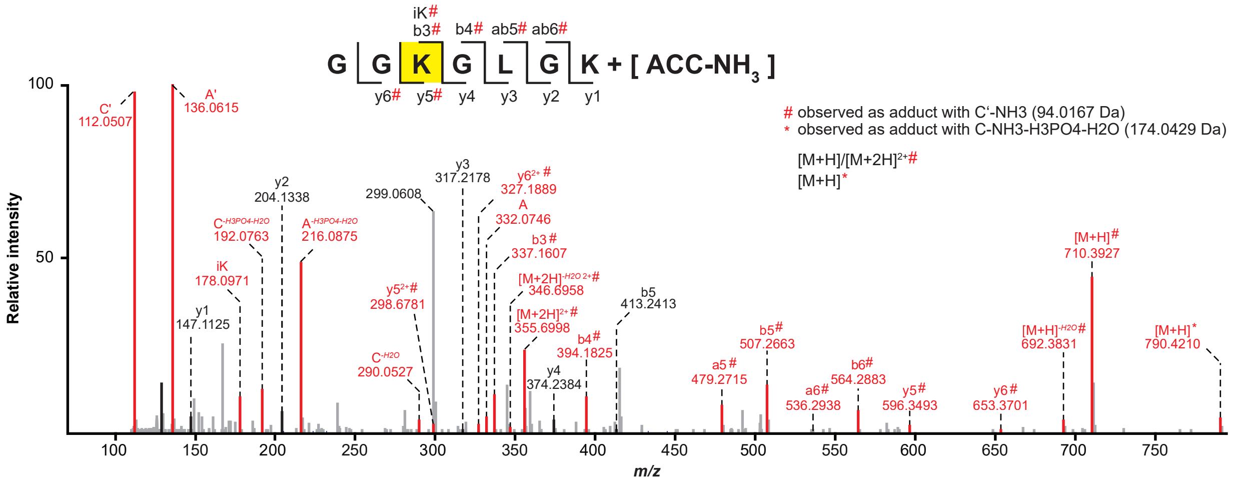

File: A_Stuetzer_180816_12mer_H14_UV_25mM; Index No. 5515; RT: 1080.55 s; m/z: 503.509; z: 3

2)

Histone H4, aa 9-16

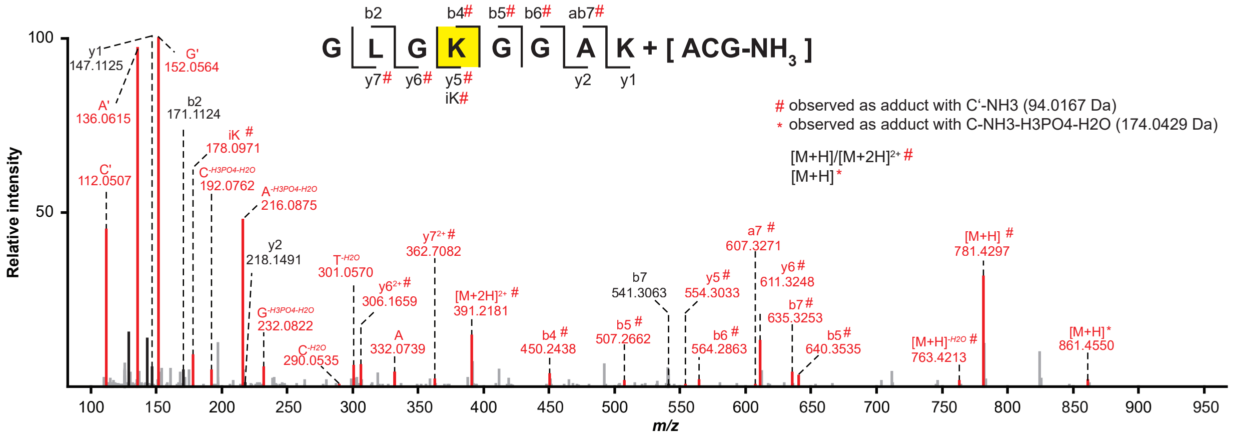

File: A_Stuetzer_180816_12mer_UV_150mM; Index No. 5610; RT: 1120.26 s; m/z: 540.524; z: 3

3) Histone H4, aa 24-35

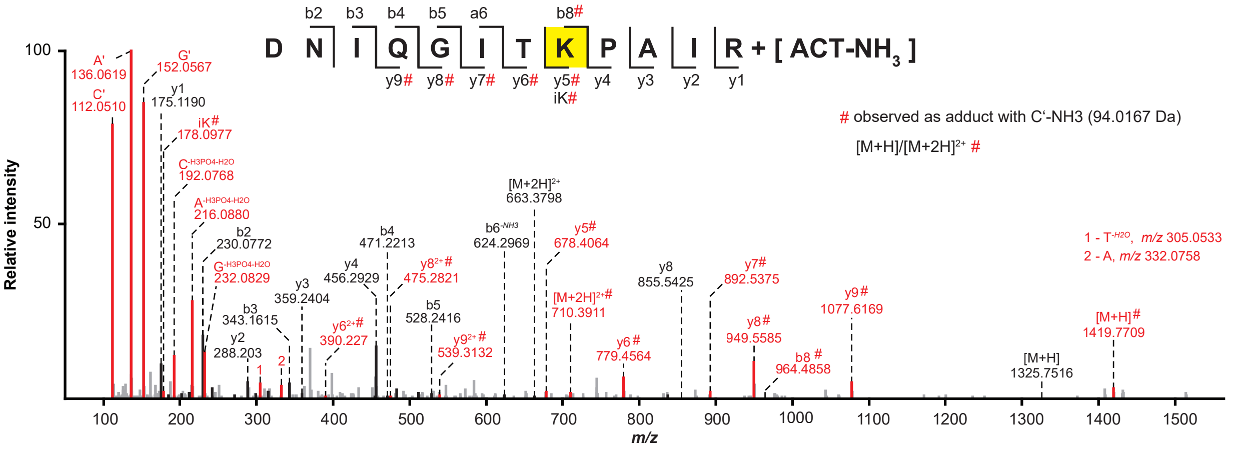

File: A_Stuetzer_180816_12mer_UV_25mM; Index No. 11342; RT: 1973.72 s; m/z: 744.967; z: 3

4) Histone H4, aa 68-77

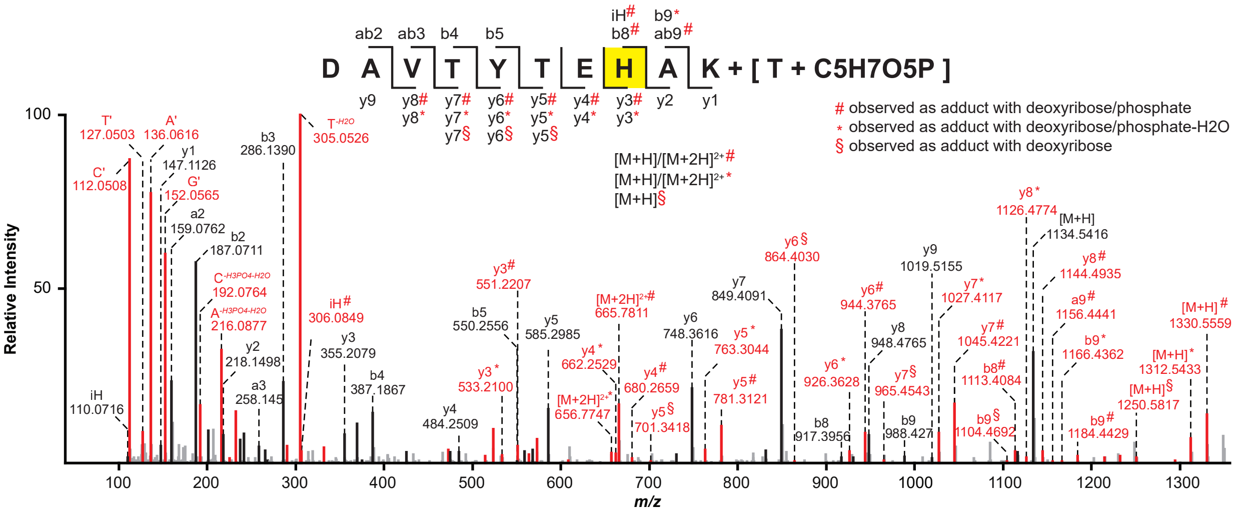

File: A_Stuetzer_180816_12mer_UV_25mM; Index No. 7171; RT: 1353.21 s; m/z: 817.805; z: 2

5) Histone H4, aa 68-78

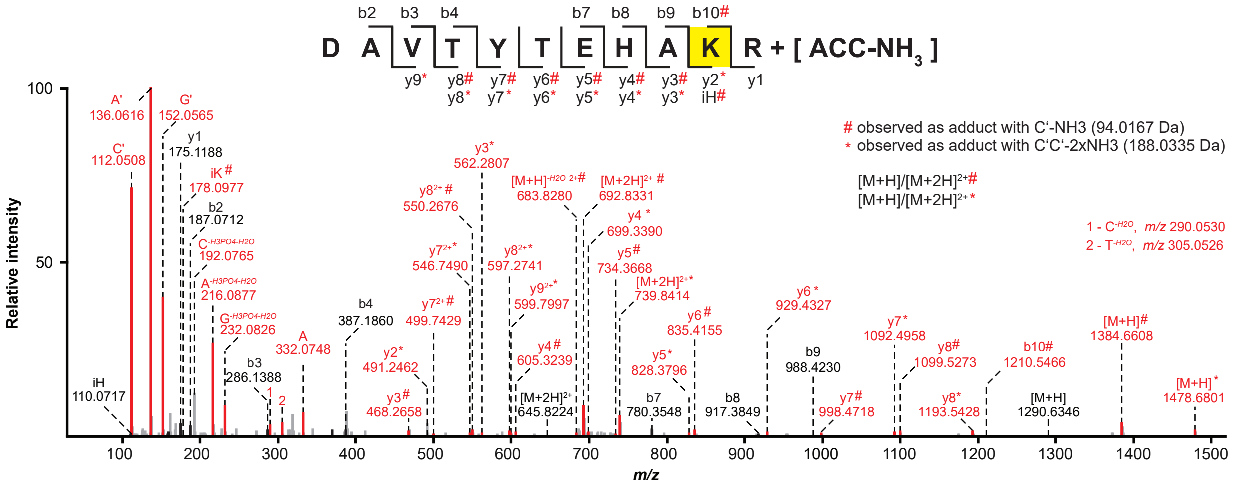

File: A_Stuetzer_180816_12mer_UV_150mM; Index No. 6619; RT: 1120.26 s; m/z: 540.524; z: 3

6) Histone H4, aa 79-91

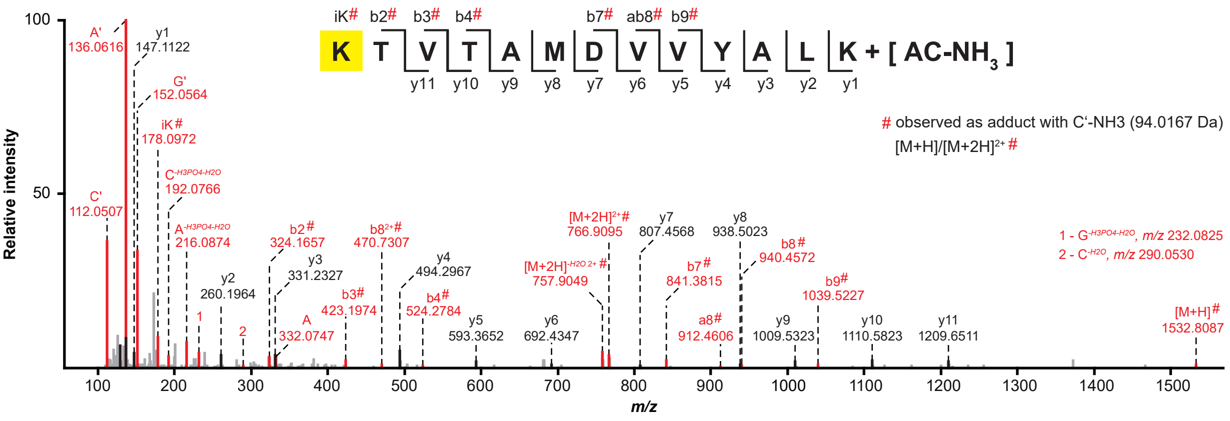

File: ; Index No. ; RT: s; m/z: 681.3; z: 3

TOPPView MS/MS spectra of core histones from *X. laevis* mononucleosomes

1)

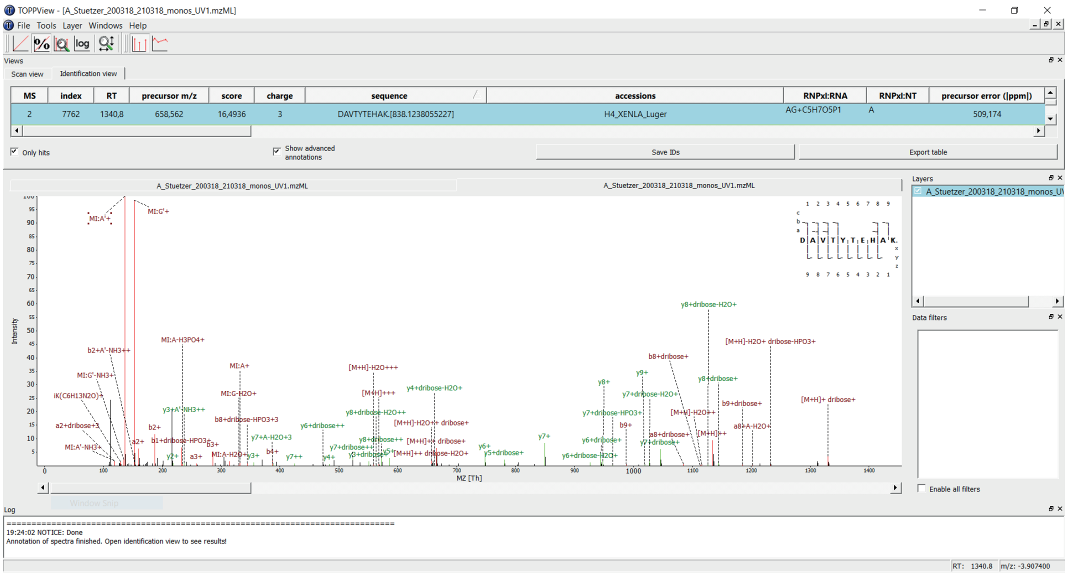

2)

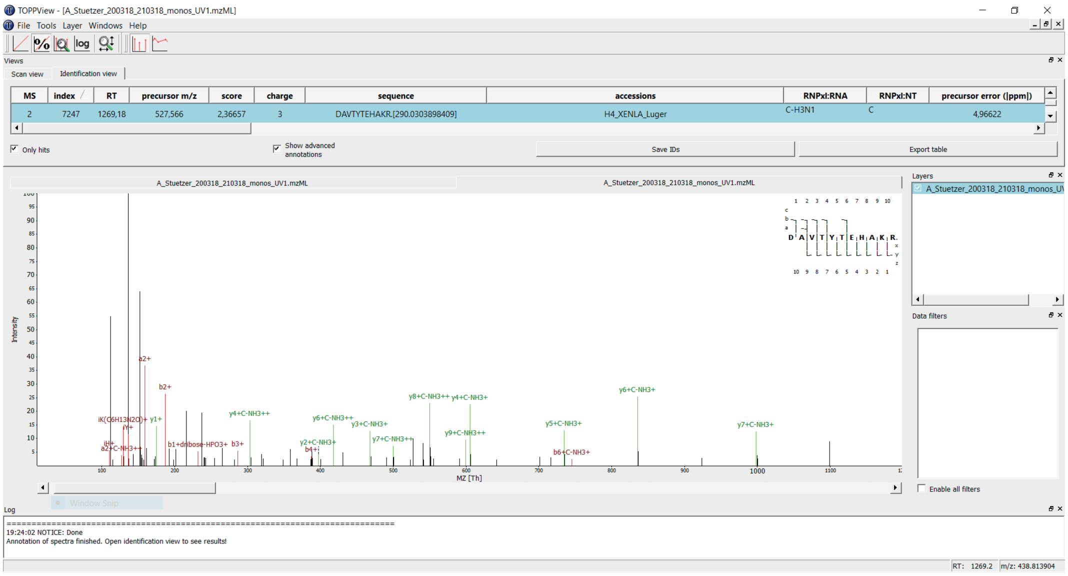

3)

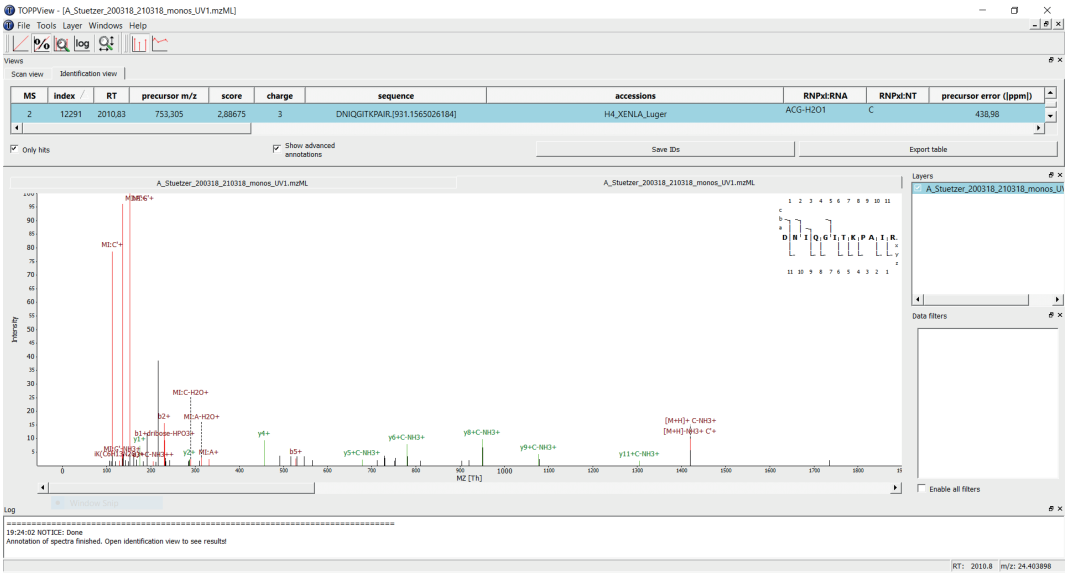

4)

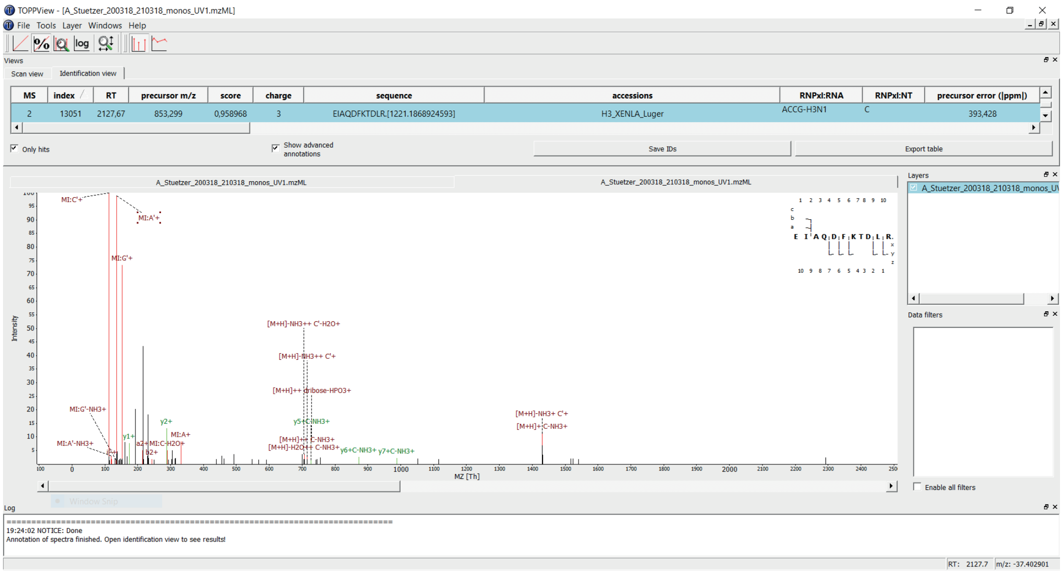
TOPPView - [A_Stuetzer_200318_210318_monos_UV1.mzML]
File Tools Layer Windows Help
Scan view
Identification view
MS | index | RT | precursor m/z | score | charge | sequence | accessions | RNPxl:RNA | RNPxl:NT | precursor error (|ppm|)
2 | 13051 | 2127,67 | 853,299 | 0,958968 | 3 | EIAQDFKTDLR.[1221.1868924593] | H3_XENLA_Luger | ACCG-H3N1 | C | 393,428
Only hits
Show advanced annotations
Save IDs
Export table
A_Stuetzer_200318_210318_monos_UV1.mzML
Layers
Data filters
Enable all filters
Intensity
MZ [Th]
Log
19:24:02 NOTICE: Done
Annotation of spectra finished. Open identification view to see results!
RT: 2127.7
m/z: -37.402901

5)

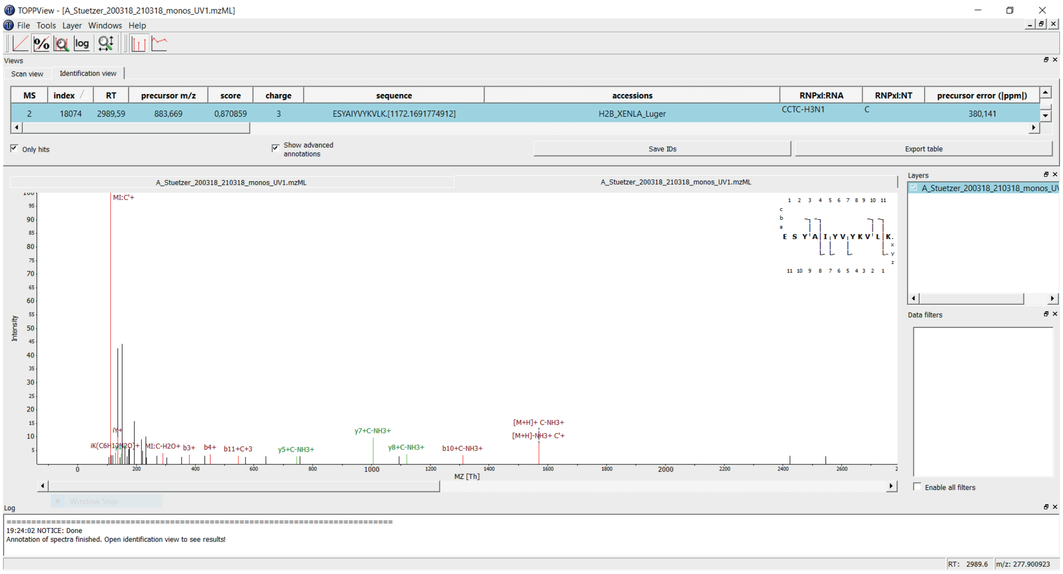
TOPPView - [A_Stuetzer_200318_210318_monos_UV1.mzML]
File Tools Layer Windows Help
Scan view
Identification view
MS | index | RT | precursor m/z | score | charge | sequence | accessions | RNPxl:RNA | RNPxl:NT | precursor error (|ppm|)
2 | 18074 | 2989,59 | 883,669 | 0,870859 | 3 | ESYAIYVYKVLK.[1172.1691774912] | H2B_XENLA_Luger | CCTC-H3N1 | C | 380,141
Only hits
Show advanced annotations
Save IDs
Export table
A_Stuetzer_200318_210318_monos_UV1.mzML
Layers
Data filters
Enable all filters
Intensity
MZ [Th]
Log
19:24:02 NOTICE: Done
Annotation of spectra finished. Open identification view to see results!
RT: 2989.6
m/z: 277.900923

6)

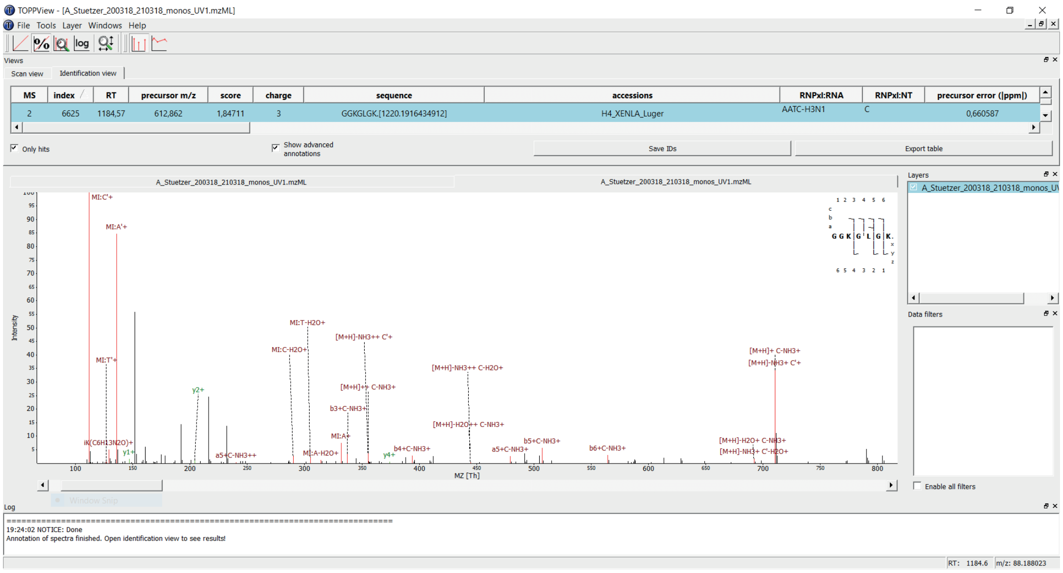
TOPPView - [A_Stuetzer_200318_210318_monos_UV1.mzML]
File Tools Layer Windows Help
Scan view
Identification view
MS | index | RT | precursor m/z | score | charge | sequence | accessions | RNPxl:RNA | RNPxl:NT | precursor error (|ppm|)
2 | 6625 | 1184,57 | 612,862 | 1,84711 | 3 | GGKGLGK.[1220.1916434912] | H4_XENLA_Luger | AATC-H3N1 | C | 0,660587
Only hits
Show advanced annotations
Save IDs
Export table
A_Stuetzer_200318_210318_monos_UV1.mzML
Layers
Data filters
Enable all filters
Intensity
MZ [Th]
Log
19:24:02 NOTICE: Done
Annotation of spectra finished. Open identification view to see results!
RT: 1184.6
m/z: 88.188023

7)

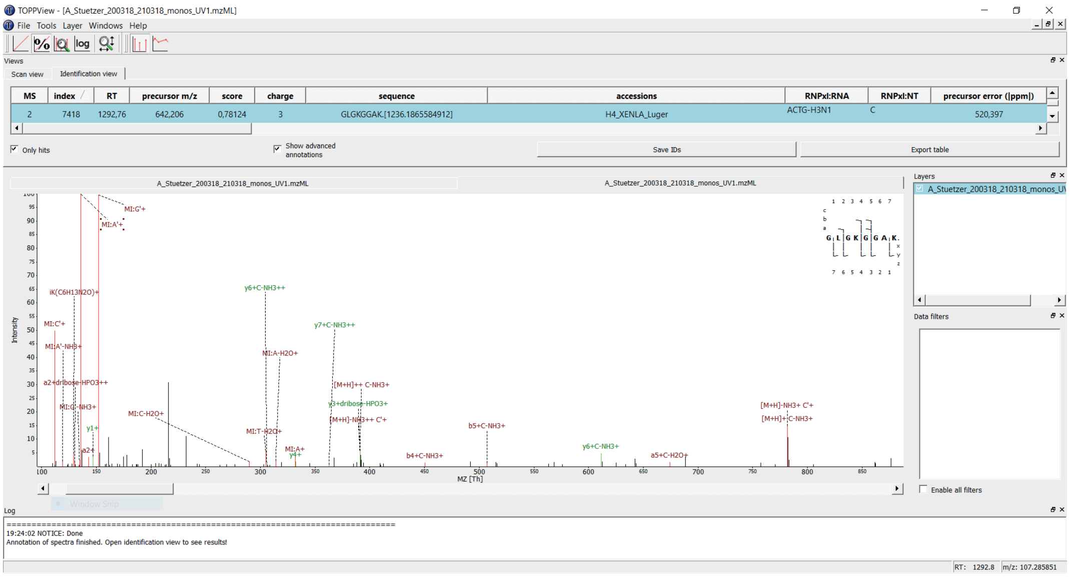
TOPPView - [A_Stuetzer_200318_210318_monos_UV1.mzML]
File Tools Layer Windows Help
Scan view Identification view
MS index RT precursor m/z score charge sequence accessions RNPxl:RNA RNPxl:NT precursor error (|ppm|)
2 7418 1292,76 642,206 0,78124 3 GLGKGGAK.[1236.1865584912] H4_XENLA_Luger ACTG-H3N1 C 520,397
Only hits
Show advanced annotations
Save IDs
Export table
A_Stuetzer_200318_210318_monos_UV1.mzML
Layers
Data filters
Enable all filters
MZ [Th]
Intensity
19:24:02 NOTICE: Done
Annotation of spectra finished. Open identification view to see results!
RT: 1292.8 m/z: 107.285851

8)

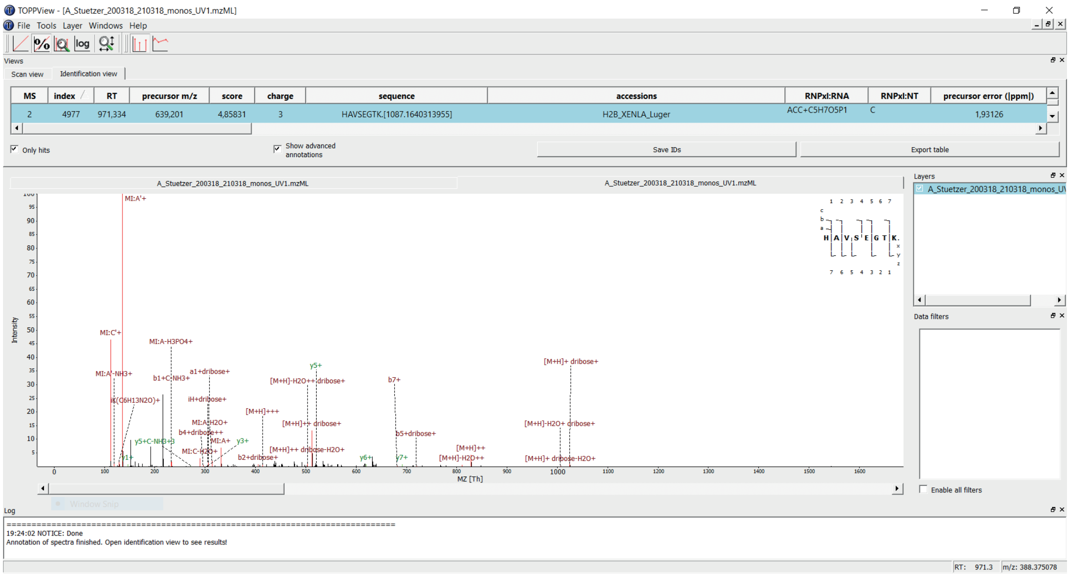
TOPPView - [A_Stuetzer_200318_210318_monos_UV1.mzML]
File Tools Layer Windows Help
Scan view Identification view
MS index RT precursor m/z score charge sequence accessions RNPxl:RNA RNPxl:NT precursor error (|ppm|)
2 4977 971,334 639,201 4,85831 3 HAVSEGTK.[1087.1640313955] H2B_XENLA_Luger ACC+C5H7O5P1 C 1,93126
Only hits
Show advanced annotations
Save IDs
Export table
A_Stuetzer_200318_210318_monos_UV1.mzML
Layers
Data filters
Enable all filters
MZ [Th]
Intensity
19:24:02 NOTICE: Done
Annotation of spectra finished. Open identification view to see results!
RT: 971.3 m/z: 388.375078

9)

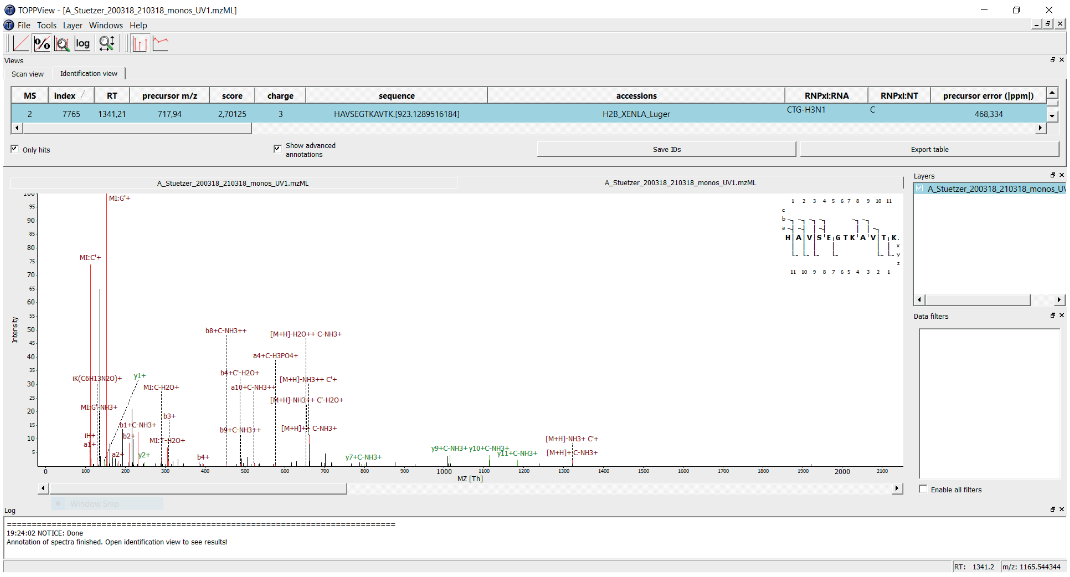
TOPPView - [A_Stuetzer_200318_210318_monos_UV1.mzML]
File Tools Layer Windows Help
Scan view Identification view
MS index RT precursor m/z score charge sequence accessions RNPxl:RNA RNPxl:NT precursor error (|ppm|)
2 7765 1341,21 717,94 2,70125 3 HAVSEGTKAVTK.[923.1289516184] H2B_XENLA_Luger CTG-H3N1 C 468,334
Only hits
Show advanced annotations
Save IDs
Export table
A_Stuetzer_200318_210318_monos_UV1.mzML
Layers
Data filters
Enable all filters
MZ [Th]
Intensity
19:24:02 NOTICE: Done
Annotation of spectra finished. Open identification view to see results!
RT: 1341.2 m/z: 1165.544344

10)

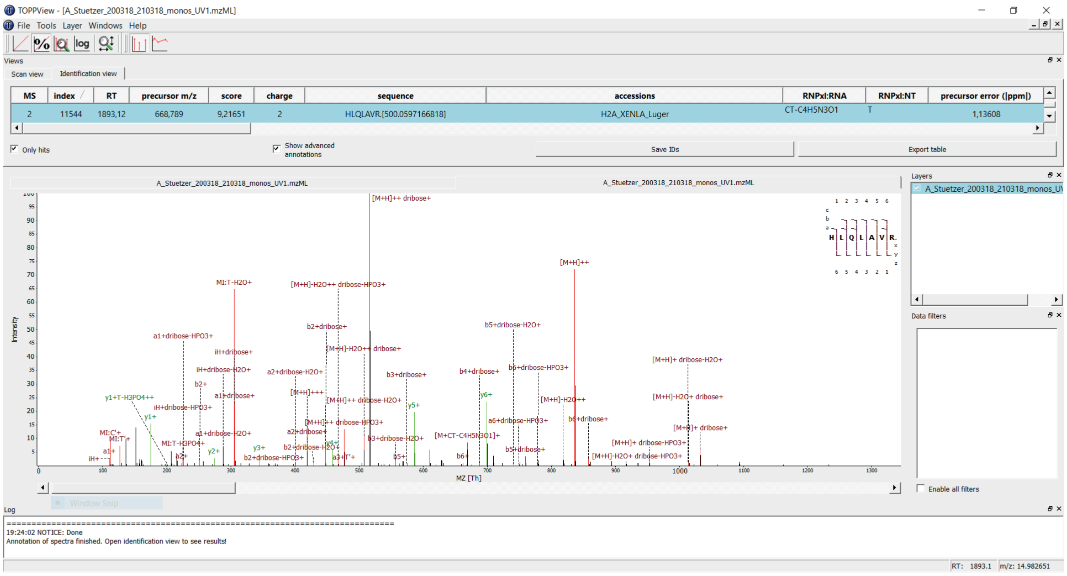
TOPPView - [A_Stuetzer_200318_210318_monos_UV1.mzML]
File Tools Layer Windows Help
Scan view
Identification view
MS | index | RT | precursor m/z | score | charge | sequence | accessions | RNPxl:RNA | RNPxl:NT | precursor error (|ppm|)
2 | 11544 | 1893,12 | 668,789 | 9,21651 | 2 | HLQLAVR.[500.0597166818] | H2A_XENLA_Luger | CT-C4H5N3O1 | T | 1,13608
Only hits
Show advanced annotations
Save IDs
Export table
A_Stuetzer_200318_210318_monos_UV1.mzML
[M+H]++ dribose+
[M+H]++
MI:T-H2O+
[M+H]-H2O++ dribose-HPO3+
MZ [Th]
Intensity
Layers
Data filters
Enable all filters
19:24:02 NOTICE: Done
Annotation of spectra finished. Open identification view to see results!
RT: 1893.1 m/z: 14.982651

11)

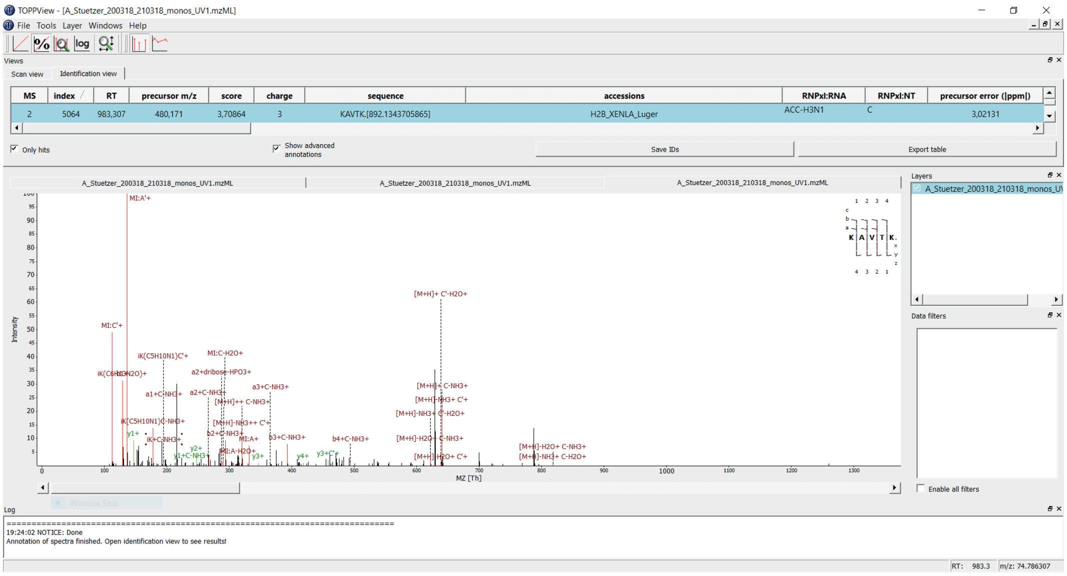
TOPPView - [A_Stuetzer_200318_210318_monos_UV1.mzML]
File Tools Layer Windows Help
Scan view
Identification view
MS | index | RT | precursor m/z | score | charge | sequence | accessions | RNPxl:RNA | RNPxl:NT | precursor error (|ppm|)
2 | 5064 | 983,307 | 480,171 | 3,70864 | 3 | KAVTK.[892.1343705865] | H2B_XENLA_Luger | ACC-H3N1 | C | 3,02131
Only hits
Show advanced annotations
Save IDs
Export table
A_Stuetzer_200318_210318_monos_UV1.mzML
MI:A'+
MI:C'+
[M+H]+ C'-H2O+
MZ [Th]
Intensity
Layers
Data filters
Enable all filters
19:24:02 NOTICE: Done
Annotation of spectra finished. Open identification view to see results!
RT: 983.3 m/z: 74.786307

12)

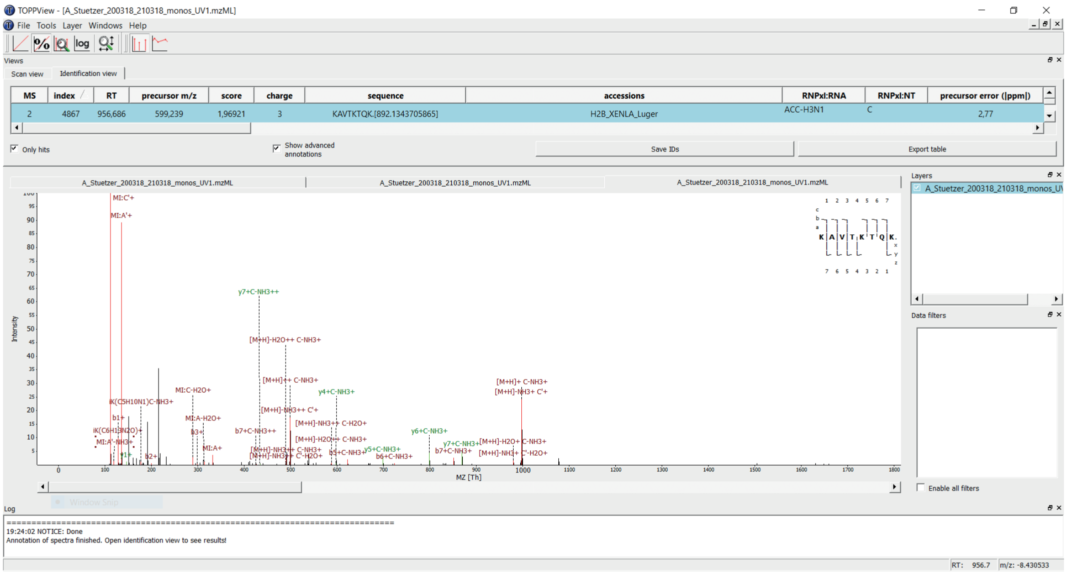
TOPPView - [A_Stuetzer_200318_210318_monos_UV1.mzML]
File Tools Layer Windows Help
Scan view
Identification view
MS | index | RT | precursor m/z | score | charge | sequence | accessions | RNPxl:RNA | RNPxl:NT | precursor error (|ppm|)
2 | 4867 | 956,686 | 599,239 | 1,96921 | 3 | KAVTKTQK.[892.1343705865] | H2B_XENLA_Luger | ACC-H3N1 | C | 2,77
Only hits
Show advanced annotations
Save IDs
Export table
A_Stuetzer_200318_210318_monos_UV1.mzML
MI:C'+
MI:A'+
y7+C-NH3++
[M+H]-H2O++ C-NH3+
MZ [Th]
Intensity
Layers
Data filters
Enable all filters
19:24:02 NOTICE: Done
Annotation of spectra finished. Open identification view to see results!
RT: 956.7 m/z: -8.430533

13)

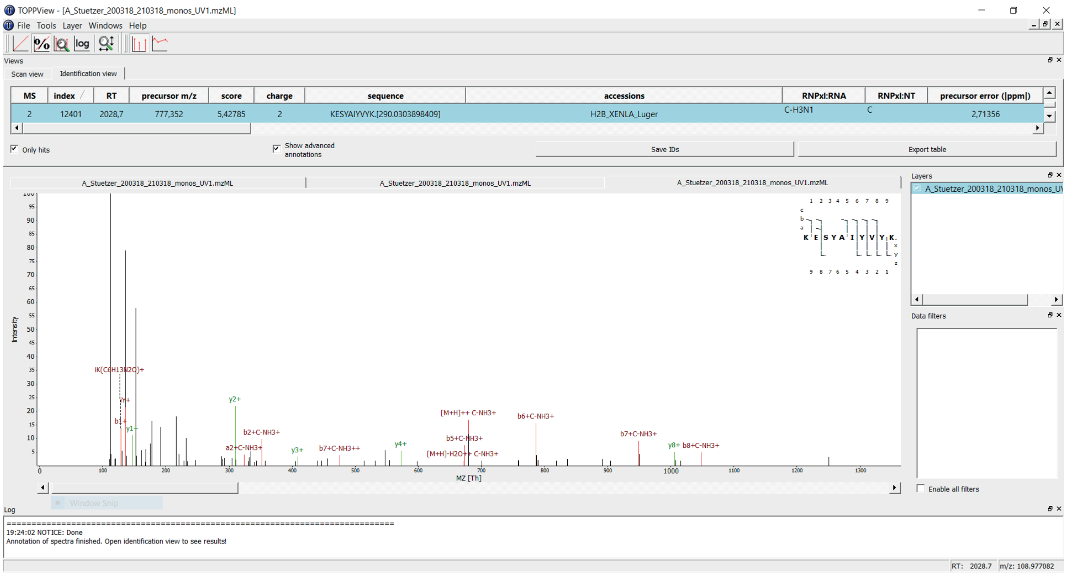
TOPPView - [A_Stuetzer_200318_210318_monos_UV1.mzML]
MS | index | RT | precursor m/z | score | charge | sequence | accessions | RNPxl:RNA | RNPxl:NT | precursor error (|ppm|)
2 | 12401 | 2028,7 | 777,352 | 5,42785 | 2 | KESYAIYVYK.[290.0303898409] | H2B_XENLA_Luger | C-H3N1 | C | 2,71356
19:24:02 NOTICE: Done
Annotation of spectra finished. Open identification view to see results!
RT: 2028.7 m/z: 108.977082

14)

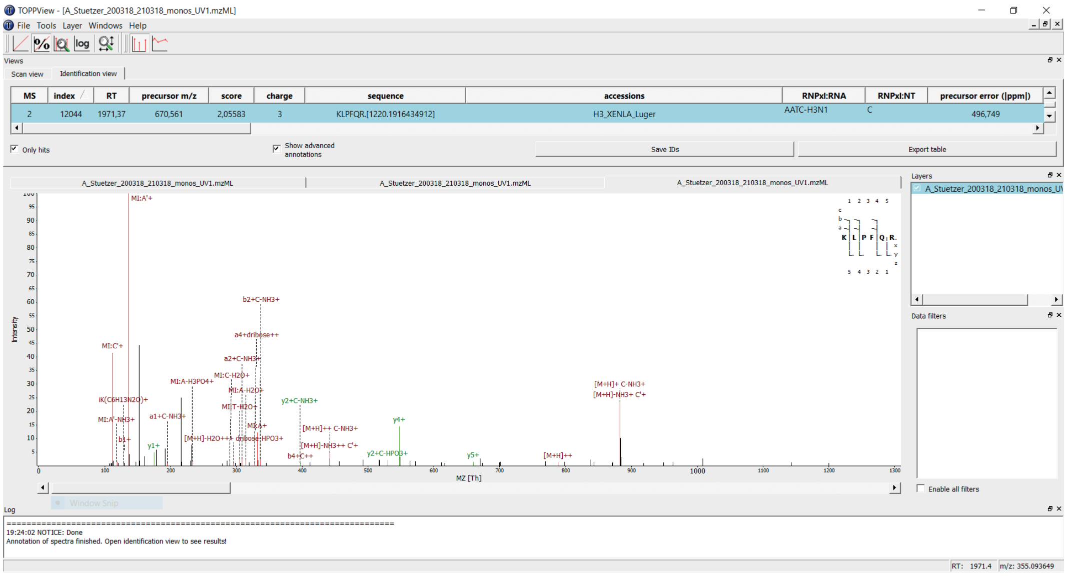
TOPPView - [A_Stuetzer_200318_210318_monos_UV1.mzML]
MS | index | RT | precursor m/z | score | charge | sequence | accessions | RNPxl:RNA | RNPxl:NT | precursor error (|ppm|)
2 | 12044 | 1971,37 | 670,561 | 2,05583 | 3 | KLPFQR.[1220.1916434912] | H3_XENLA_Luger | AATC-H3N1 | C | 496,749
19:24:02 NOTICE: Done
Annotation of spectra finished. Open identification view to see results!
RT: 1971.4 m/z: 355.093649

15)

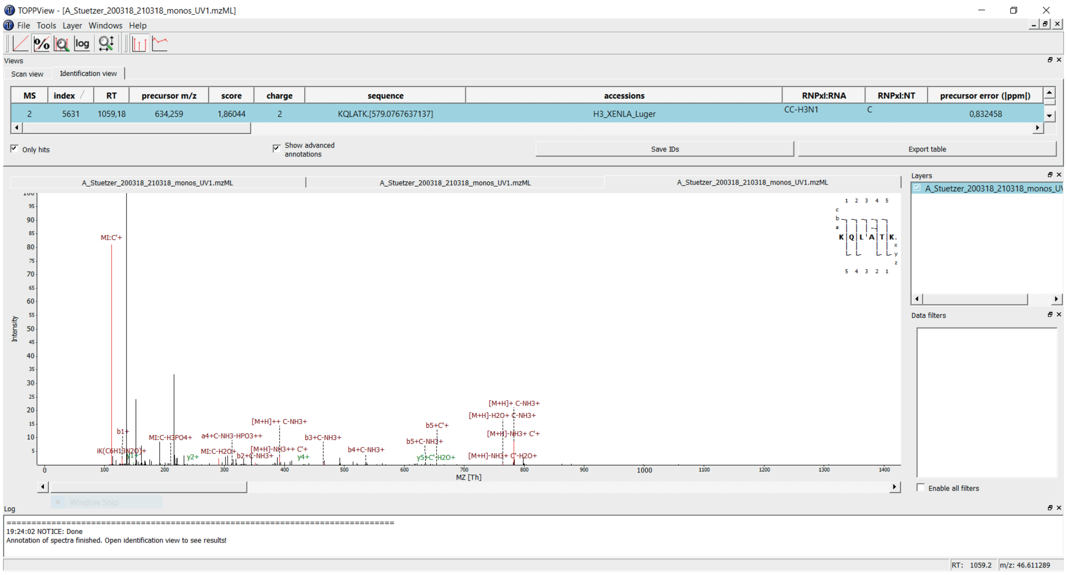
TOPPView - [A_Stuetzer_200318_210318_monos_UV1.mzML]
MS | index | RT | precursor m/z | score | charge | sequence | accessions | RNPxl:RNA | RNPxl:NT | precursor error (|ppm|)
2 | 5631 | 1059,18 | 634,259 | 1,86044 | 2 | KQLATK.[579.0767637137] | H3_XENLA_Luger | CC-H3N1 | C | 0,832458
19:24:02 NOTICE: Done
Annotation of spectra finished. Open identification view to see results!
RT: 1059.2 m/z: 46.611289

16)

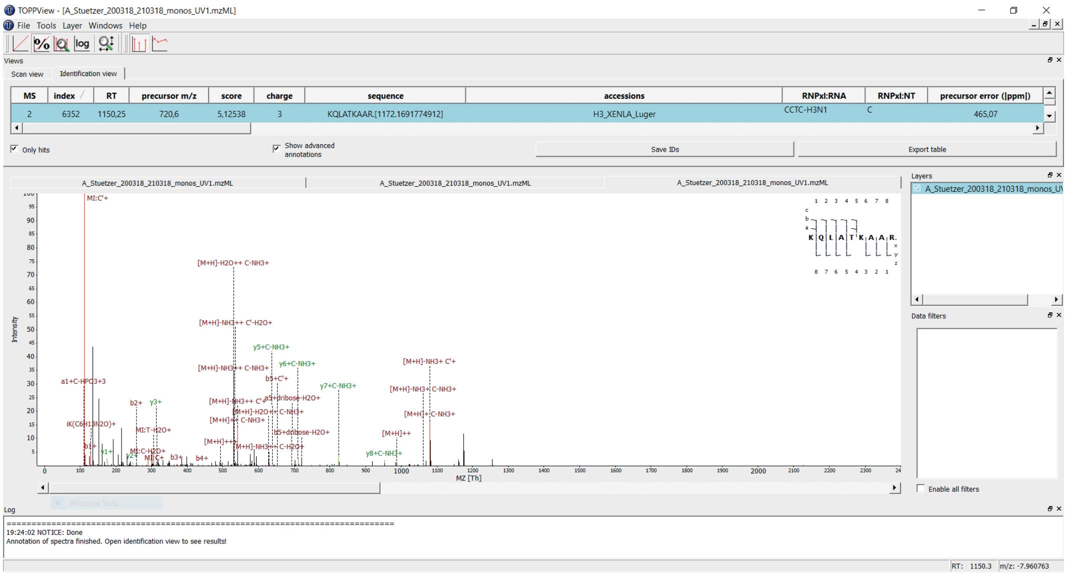
TOPPView - [A_Stuetzer_200318_210318_monos_UV1.mzML]
File Tools Layer Windows Help
Scan view Identification view
MS index RT precursor m/z score charge sequence accessions RNPxl:RNA RNPxl:NT precursor error (|ppm|)
2 6352 1150,25 720,6 5,12538 3 KQLATKAAR.[1172.1691774912] H3_XENLA_Luger CCTC-H3N1 C 465,07
Only hits
Show advanced annotations
Save IDs
Export table
Layers
Data filters
Enable all filters
MZ [Th]
Intensity
19:24:02 NOTICE: Done
Annotation of spectra finished. Open identification view to see results!

17)

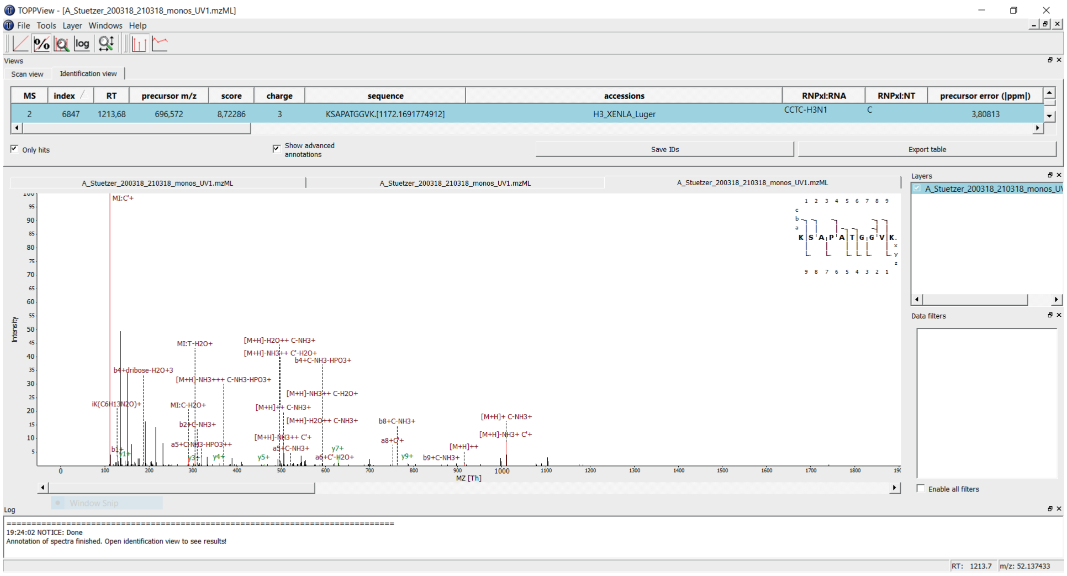
TOPPView - [A_Stuetzer_200318_210318_monos_UV1.mzML]
File Tools Layer Windows Help
Scan view Identification view
MS index RT precursor m/z score charge sequence accessions RNPxl:RNA RNPxl:NT precursor error (|ppm|)
2 6847 1213,68 696,572 8,72286 3 KSAPATGGVK.[1172.1691774912] H3_XENLA_Luger CCTC-H3N1 C 3,80813
Only hits
Show advanced annotations
Save IDs
Export table
Layers
Data filters
Enable all filters
MZ [Th]
Intensity
19:24:02 NOTICE: Done
Annotation of spectra finished. Open identification view to see results!

18)

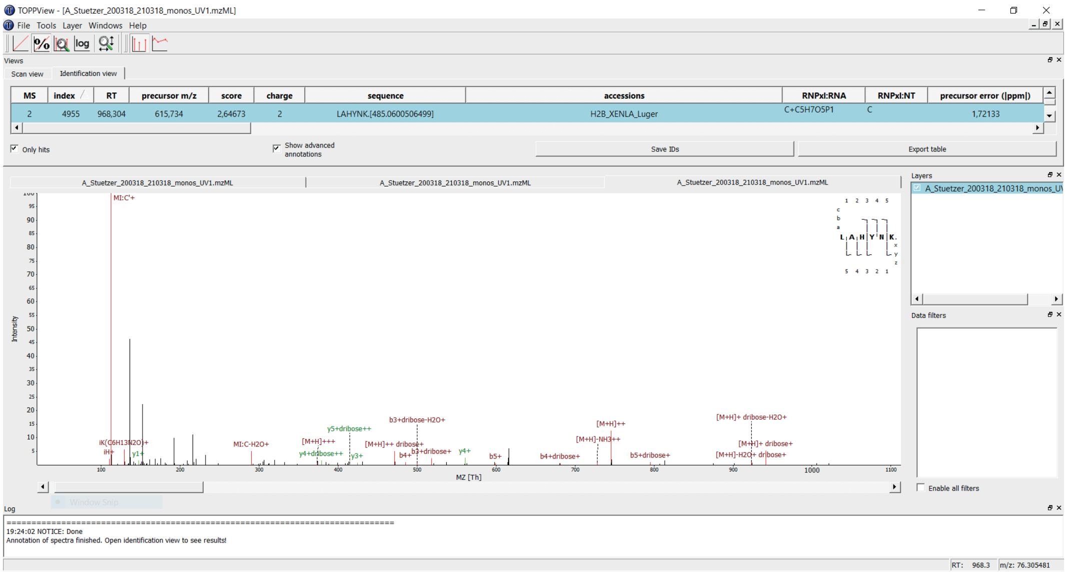
TOPPView - [A_Stuetzer_200318_210318_monos_UV1.mzML]
File Tools Layer Windows Help
Scan view Identification view
MS index RT precursor m/z score charge sequence accessions RNPxl:RNA RNPxl:NT precursor error (|ppm|)
2 4955 968,304 615,734 2,64673 2 LAHYNK.[485.0600506499] H2B_XENLA_Luger C+C5H7O5P1 C 1,72133
Only hits
Show advanced annotations
Save IDs
Export table
Layers
Data filters
Enable all filters
MZ [Th]
Intensity
19:24:02 NOTICE: Done
Annotation of spectra finished. Open identification view to see results!

19)

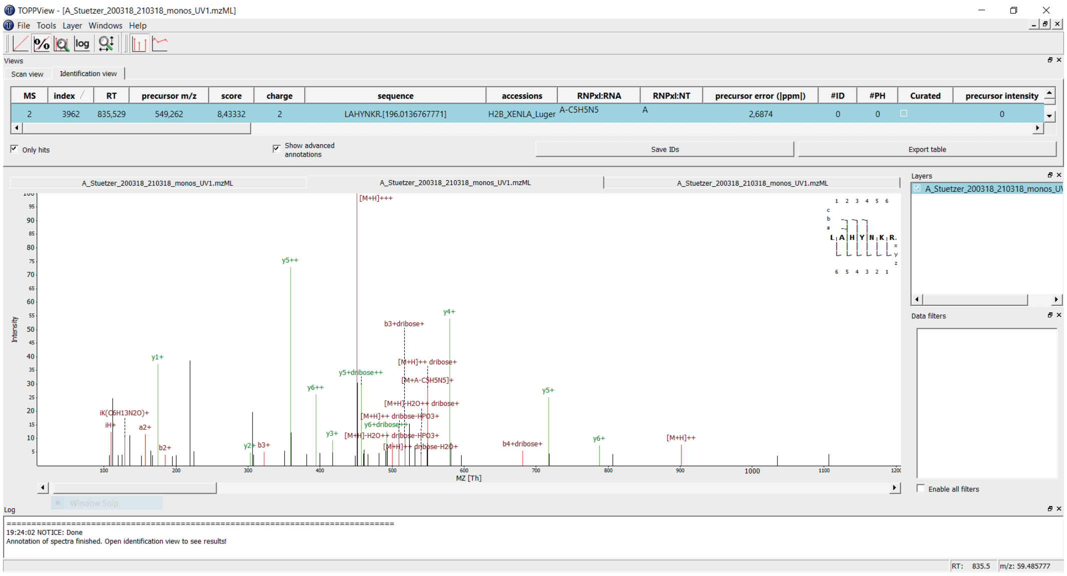
TOPPView - [A_Stuetzer_200318_210318_monos_UV1.mzML]
MS | index | RT | precursor m/z | score | charge | sequence | accessions | RNPxl:RNA | RNPxl:NT | precursor error (|ppm|) | #ID | #PH | Curated | precursor intensity
2 | 3962 | 835,529 | 549,262 | 8,43332 | 2 | LAHYNKR.[196.0136767771] | H2B_XENLA_Luger | A-C5H5N5 | A | 2,6874 | 0 | 0 | | 0
19:24:02 NOTICE: Done
Annotation of spectra finished. Open identification view to see results!

20)

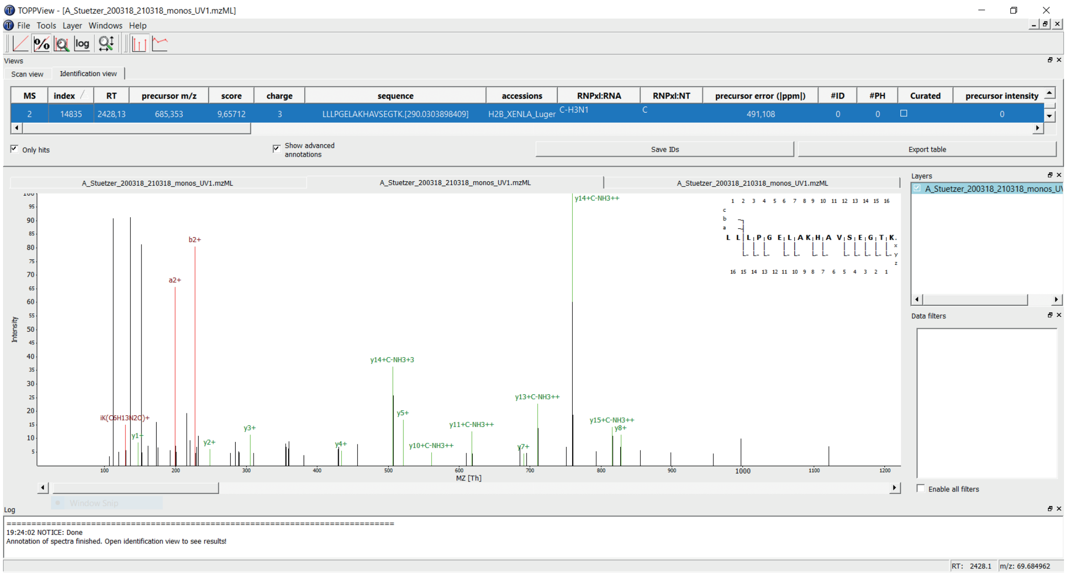
TOPPView - [A_Stuetzer_200318_210318_monos_UV1.mzML]
MS | index | RT | precursor m/z | score | charge | sequence | accessions | RNPxl:RNA | RNPxl:NT | precursor error (|ppm|) | #ID | #PH | Curated | precursor intensity
2 | 14835 | 2428,13 | 685,353 | 9,65712 | 3 | LLLPGELAKHAVSEGTK.[290.0303898409] | H2B_XENLA_Luger | C-H3N1 | C | 491,108 | 0 | 0 | | 0
19:24:02 NOTICE: Done
Annotation of spectra finished. Open identification view to see results!

21)

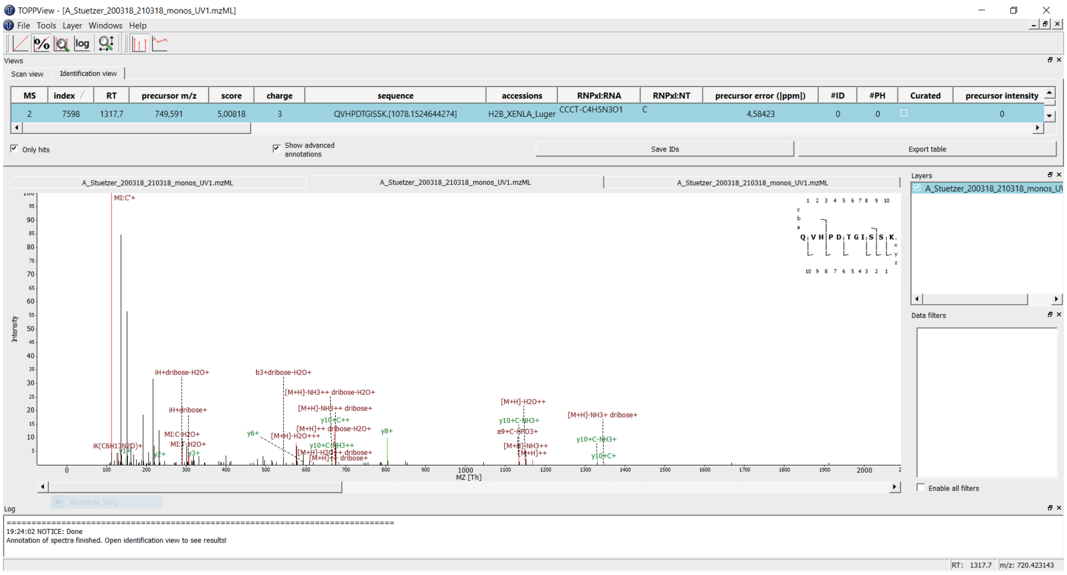
TOPPView - [A_Stuetzer_200318_210318_monos_UV1.mzML]
MS | index | RT | precursor m/z | score | charge | sequence | accessions | RNPxl:RNA | RNPxl:NT | precursor error (|ppm|) | #ID | #PH | Curated | precursor intensity
2 | 7598 | 1317,7 | 749,591 | 5,00818 | 3 | QVHPDTGISSK.[1078.1524644274] | H2B_XENLA_Luger | CCCT-C4H5N3O1 | C | 4,58423 | 0 | 0 | | 0
19:24:02 NOTICE: Done
Annotation of spectra finished. Open identification view to see results!

22)

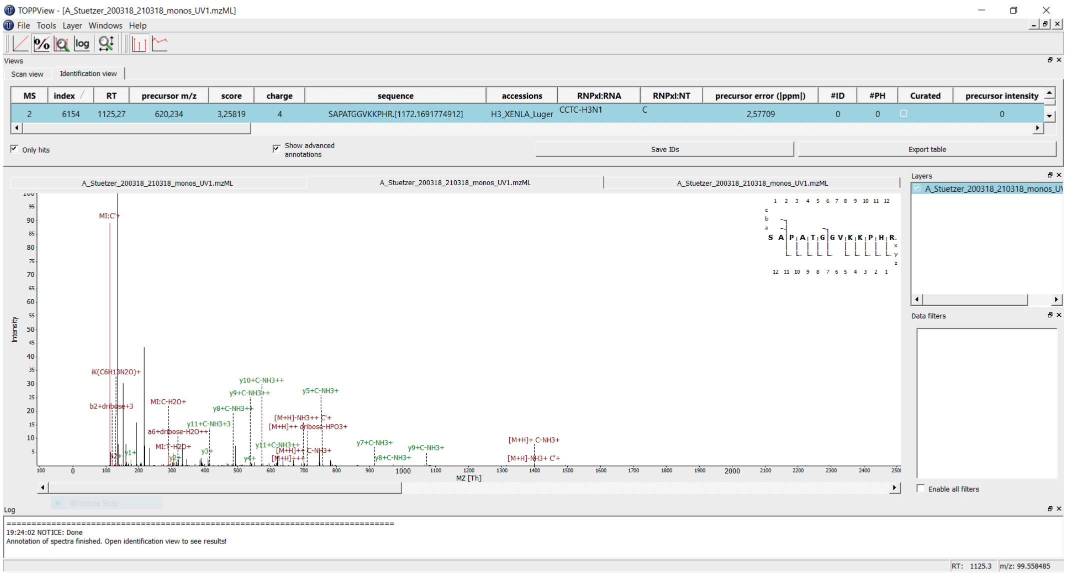
TOPPView - [A_Stuetzer_200318_210318_monos_UV1.mzML]
MS | index | RT | precursor m/z | score | charge | sequence | accessions | RNPxl:RNA | RNPxl:NT | precursor error (|ppm|) | #ID | #PH | Curated | precursor intensity
2 | 6154 | 1125,27 | 620,234 | 3,25819 | 4 | SAPATGGVKKPHR.[1172.1691774912] | H3_XENLA_Luger | CCTC-H3N1 | C | 2,57709 | 0 | 0 | | 0
19:24:02 NOTICE: Done
Annotation of spectra finished. Open identification view to see results!

23)

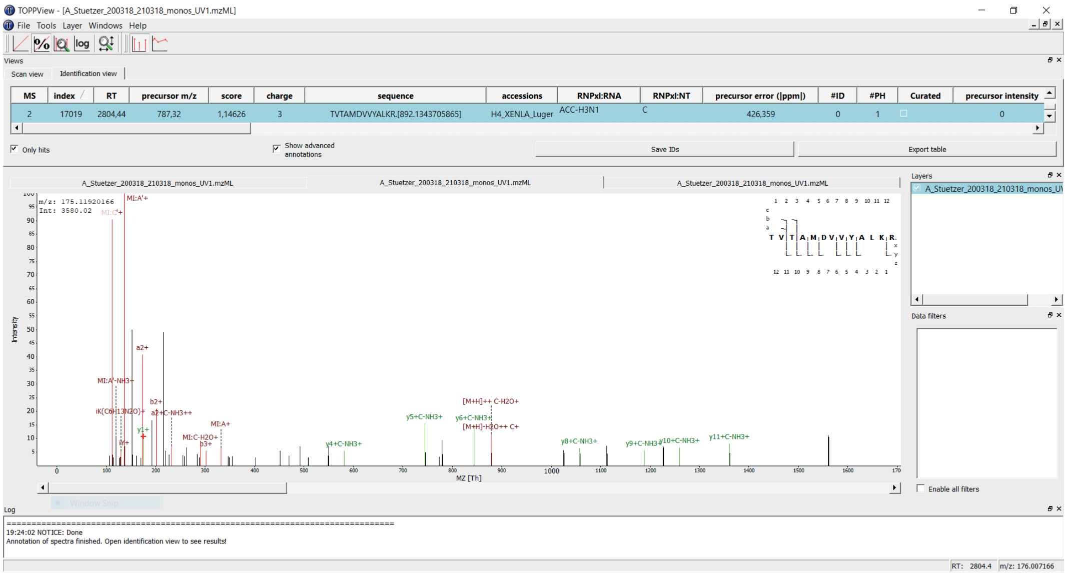
TOPPView - [A_Stuetzer_200318_210318_monos_UV1.mzML]
MS | index | RT | precursor m/z | score | charge | sequence | accessions | RNPxl:RNA | RNPxl:NT | precursor error (|ppm|) | #ID | #PH | Curated | precursor intensity
2 | 17019 | 2804,44 | 787,32 | 1,14626 | 3 | TVTAMDVVYALKR.[892.1343705865] | H4_XENLA_Luger | ACC-H3N1 | C | 426,359 | 0 | 1 | | 0
19:24:02 NOTICE: Done
Annotation of spectra finished. Open identification view to see results!

24)

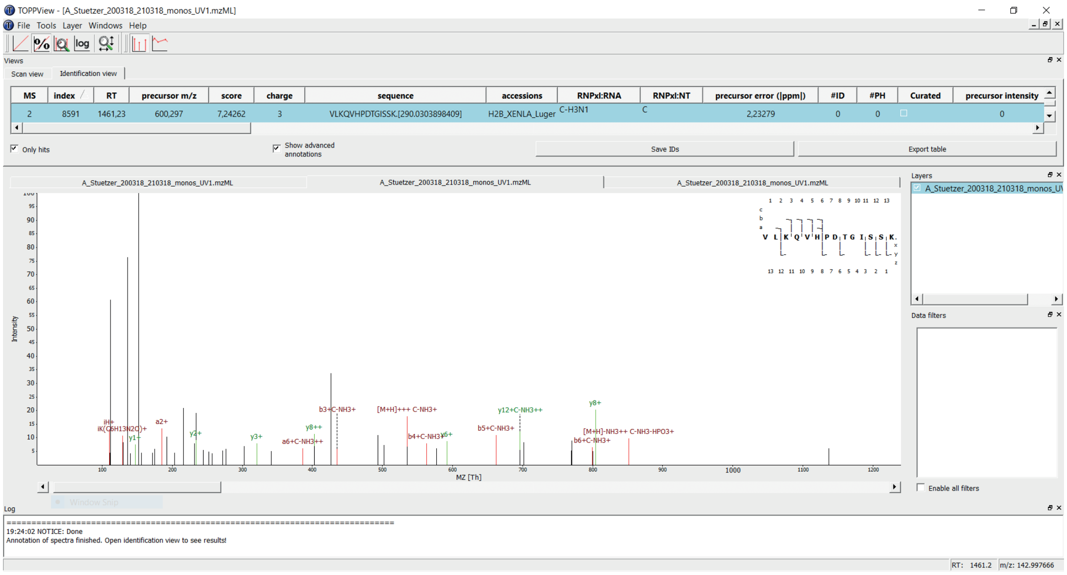
TOPPView - [A_Stuetzer_200318_210318_monos_UV1.mzML]
MS | index | RT | precursor m/z | score | charge | sequence | accessions | RNPxl:RNA | RNPxl:NT | precursor error (|ppm|) | #ID | #PH | Curated | precursor intensity
2 | 8591 | 1461,23 | 600,297 | 7,24262 | 3 | VLKQVHPDTGISSK.[290.0303898409] | H2B_XENLA_Luger | C-H3N1 | C | 2,23279 | 0 | 0 | | 0
19:24:02 NOTICE: Done
Annotation of spectra finished. Open identification view to see results!

25)

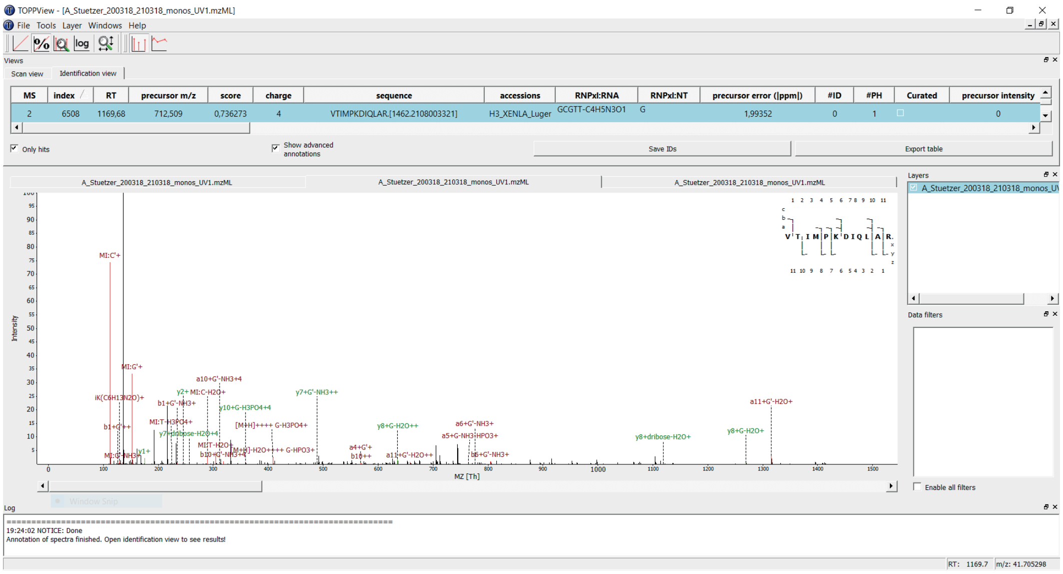
TOPPView - [A_Stuetzer_200318_210318_monos_UV1.mzML]
MS | index | RT | precursor m/z | score | charge | sequence | accessions | RNPxl:RNA | RNPxl:NT | precursor error (|ppm|) | #ID | #PH | Curated | precursor intensity
2 | 6508 | 1169,68 | 712,509 | 0,736273 | 4 | VTIMPKDIQLAR.[1462.2108003321] | H3_XENLA_Luger | GCGTT-C4H5N3O1 | G | 1,99352 | 0 | 1 | | 0
19:24:02 NOTICE: Done
Annotation of spectra finished. Open identification view to see results!

26)

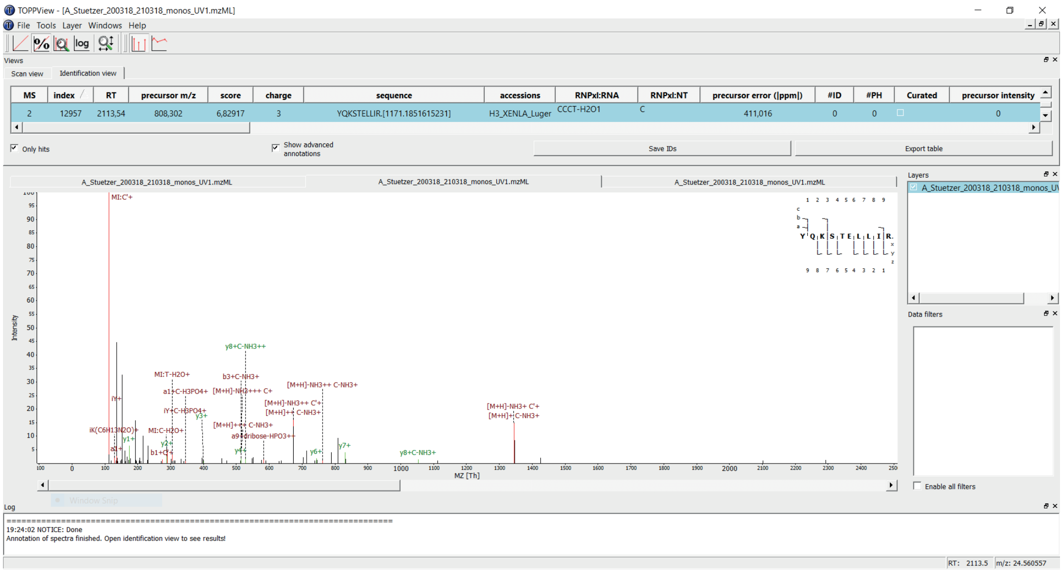
TOPPView - [A_Stuetzer_200318_210318_monos_UV1.mzML]
MS | index | RT | precursor m/z | score | charge | sequence | accessions | RNPxl:RNA | RNPxl:NT | precursor error (|ppm|) | #ID | #PH | Curated | precursor intensity
2 | 12957 | 2113,54 | 808,302 | 6,82917 | 3 | YQKSTELLIR.[1171.1851615231] | H3_XENLA_Luger | CCCT-H2O1 | C | 411,016 | 0 | 0 | | 0
19:24:02 NOTICE: Done
Annotation of spectra finished. Open identification view to see results!

27)

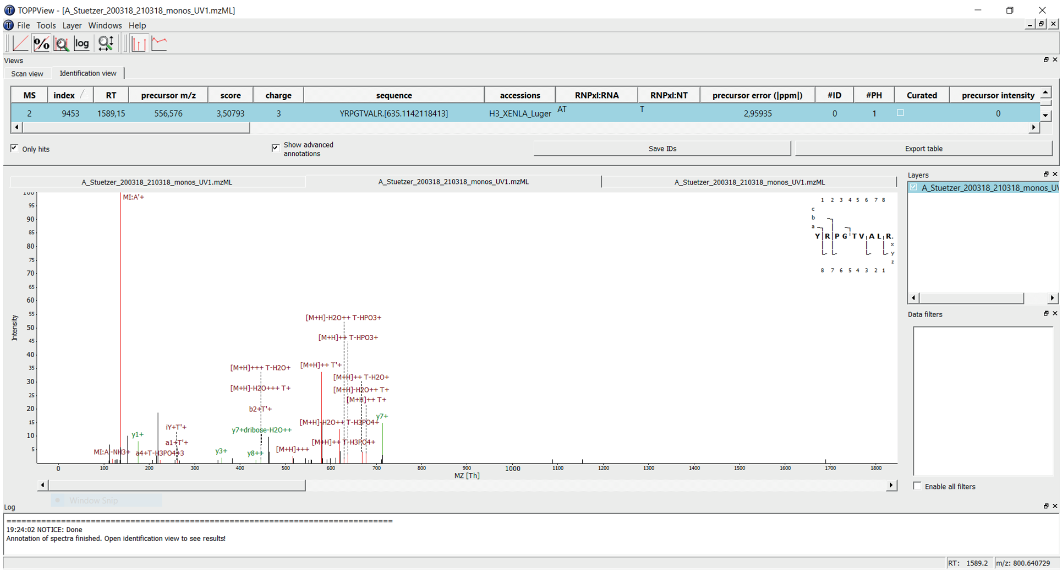
TOPPView - [A_Stuetzer_200318_210318_monos_UV1.mzML]
MS | index | RT | precursor m/z | score | charge | sequence | accessions | RNPxl:RNA | RNPxl:NT | precursor error (|ppm|) | #ID | #PH | Curated | precursor intensity
2 | 9453 | 1589,15 | 556,576 | 3,50793 | 3 | YRPGTVALR.[635.1142118413] | H3_XENLA_Luger | AT | T | 2,95935 | 0 | 1 | | 0
19:24:02 NOTICE: Done
Annotation of spectra finished. Open identification view to see results!

28)

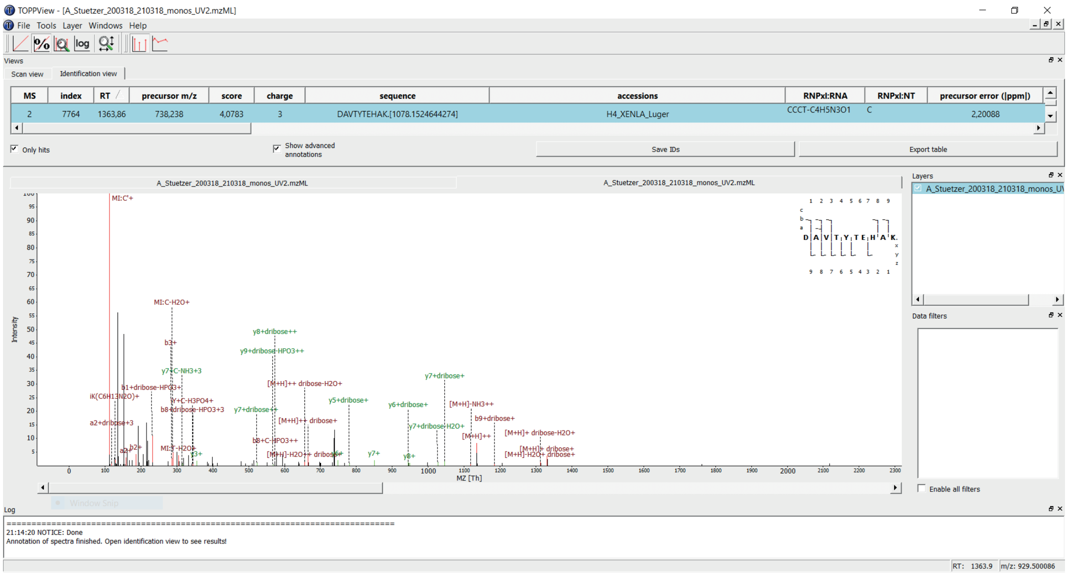

TOPPView - [A_Stuetzer_200318_210318_monos_UV2.mzML]
File Tools Layer Windows Help
Scan view
Identification view
MS | index | RT | precursor m/z | score | charge | sequence | accessions | RNPxl:RNA | RNPxl:NT | precursor error (|ppm|)
2 | 7764 | 1363,86 | 738,238 | 4,0783 | 3 | DAVTYTEHAK.[1078.1524644274] | H4_XENLA_Luger | CCCT-C4H5N3O1 | C | 2,22088
Only hits
Show advanced annotations
Save IDs
Export table
A_Stuetzer_200318_210318_monos_UV2.mzML
MI:C'+
MI:C-H2O+
y8+dribose++
y9+dribose-HPO3++
y7+dribose+
MZ [Th]
Intensity
Layers
Data filters
Enable all filters
Log
21:14:20 NOTICE: Done
Annotation of spectra finished. Open identification view to see results!
RT: 1363.9
m/z: 929.500086

29)

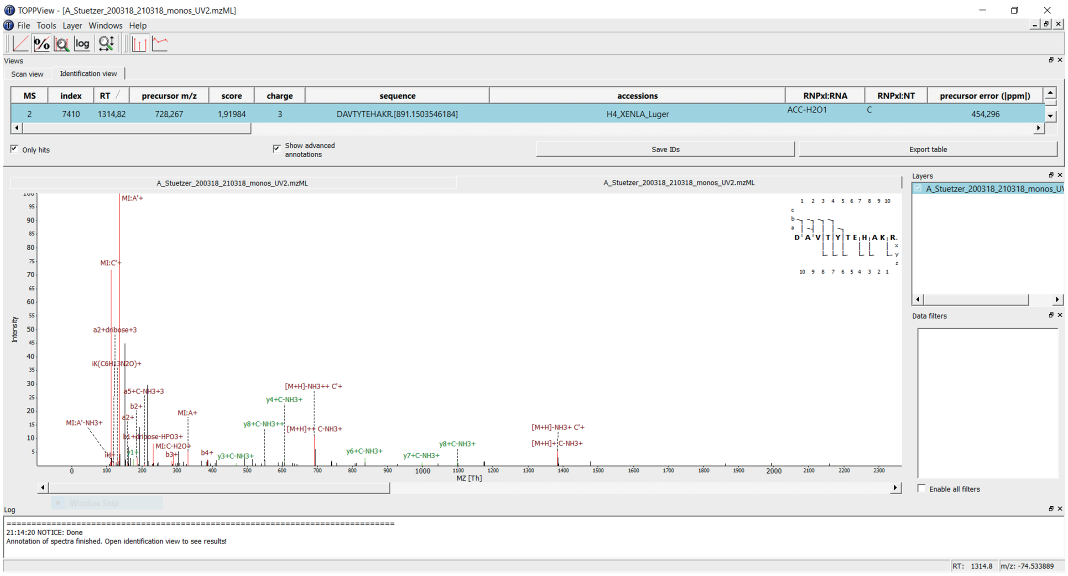

TOPPView - [A_Stuetzer_200318_210318_monos_UV2.mzML]
File Tools Layer Windows Help
Scan view
Identification view
MS | index | RT | precursor m/z | score | charge | sequence | accessions | RNPxl:RNA | RNPxl:NT | precursor error (|ppm|)
2 | 7410 | 1314,82 | 728,267 | 1,91984 | 3 | DAVTYTEHAKR.[891.1503546184] | H4_XENLA_Luger | ACC-H2O1 | C | 454,296
Only hits
Show advanced annotations
Save IDs
Export table
A_Stuetzer_200318_210318_monos_UV2.mzML
MI:A'+
MI:C'+
a2+dribose+3
MI:A+
MZ [Th]
Intensity
Layers
Data filters
Enable all filters
Log
21:14:20 NOTICE: Done
Annotation of spectra finished. Open identification view to see results!
RT: 1314.8
m/z: -74.533889

30)

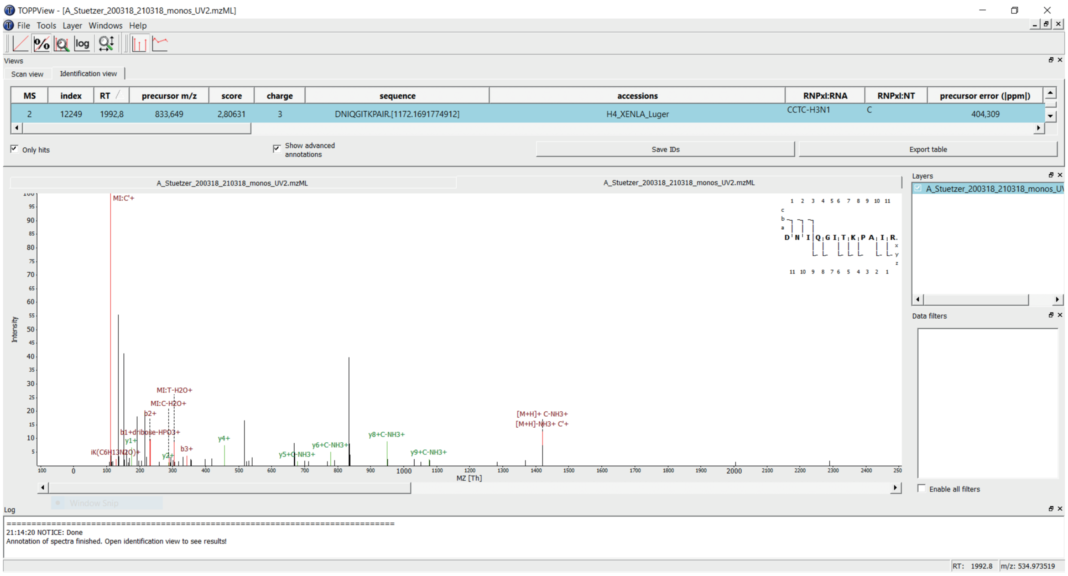

TOPPView - [A_Stuetzer_200318_210318_monos_UV2.mzML]
File Tools Layer Windows Help
Scan view
Identification view
MS | index | RT | precursor m/z | score | charge | sequence | accessions | RNPxl:RNA | RNPxl:NT | precursor error (|ppm|)
2 | 12249 | 1992,8 | 833,649 | 2,80631 | 3 | DNIQGITKPAIR.[1172.1691774912] | H4_XENLA_Luger | CCTC-H3N1 | C | 404,309
Only hits
Show advanced annotations
Save IDs
Export table
A_Stuetzer_200318_210318_monos_UV2.mzML
MI:C'+
MI:T-H2O+
MI:C-H2O+
MZ [Th]
Intensity
Layers
Data filters
Enable all filters
Log
21:14:20 NOTICE: Done
Annotation of spectra finished. Open identification view to see results!
RT: 1992.8
m/z: 534.973519

31)

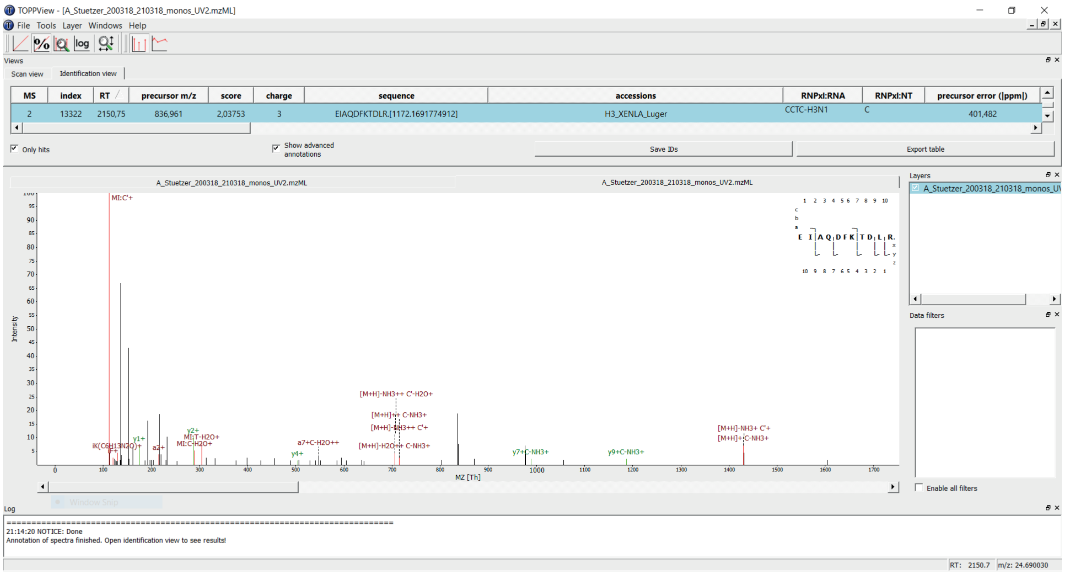
TOPPView - [A_Stuetzer_200318_210318_monos_UV2.mzML]
MS | index | RT | precursor m/z | score | charge | sequence | accessions | RNPxl:RNA | RNPxl:NT | precursor error (|ppm|)
2 | 13322 | 2150,75 | 836,961 | 2,03753 | 3 | EIAQDFKTDLR.[1172.1697774912] | H3_XENLA_Luger | CCTC-H3N1 | C | 401,482
Only hits
Show advanced annotations
Save IDs
Export table
A_Stuetzer_200318_210318_monos_UV2.mzML
MI:C'+
Intensity
MZ [Th]
Log
21:14:20 NOTICE: Done
Annotation of spectra finished. Open identification view to see results!
RT: 2150.7
m/z: 24.690030

32)

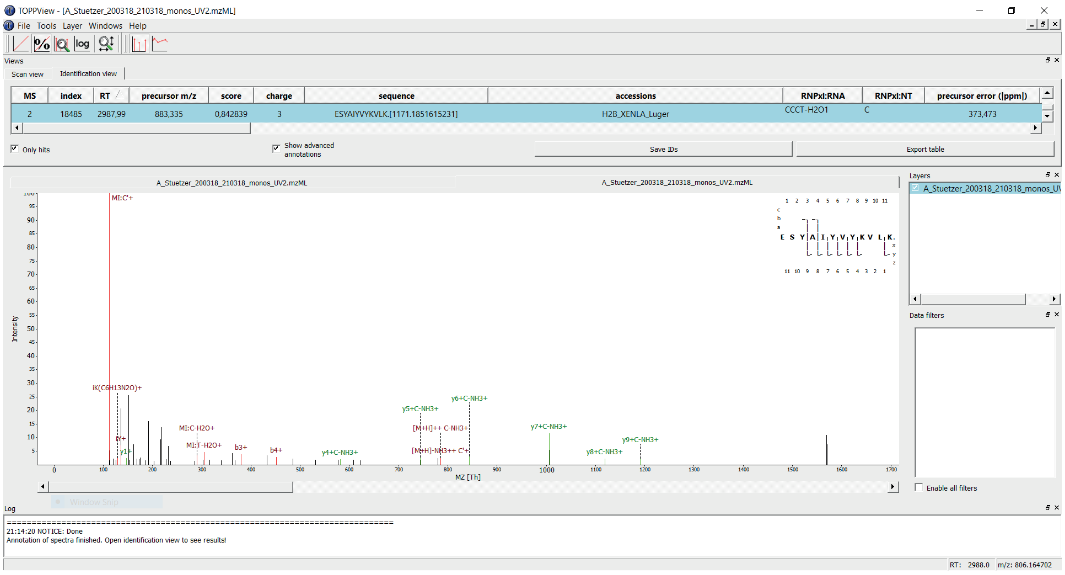
TOPPView - [A_Stuetzer_200318_210318_monos_UV2.mzML]
MS | index | RT | precursor m/z | score | charge | sequence | accessions | RNPxl:RNA | RNPxl:NT | precursor error (|ppm|)
2 | 18485 | 2987,99 | 883,335 | 0,842839 | 3 | ESYAIYVYKVLK.[1171.1851615231] | H2B_XENLA_Luger | CCCT-H2O1 | C | 373,473
Only hits
Show advanced annotations
Save IDs
Export table
A_Stuetzer_200318_210318_monos_UV2.mzML
MI:C'+
Intensity
MZ [Th]
Log
21:14:20 NOTICE: Done
Annotation of spectra finished. Open identification view to see results!
RT: 2988.0
m/z: 806.164702

33)

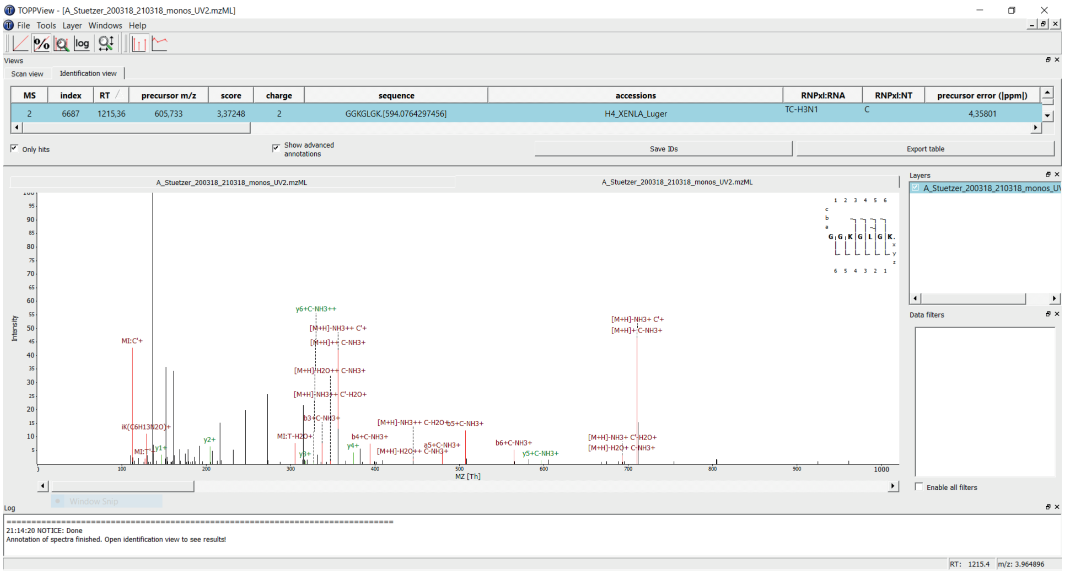
TOPPView - [A_Stuetzer_200318_210318_monos_UV2.mzML]
MS | index | RT | precursor m/z | score | charge | sequence | accessions | RNPxl:RNA | RNPxl:NT | precursor error (|ppm|)
2 | 6687 | 1215,36 | 605,733 | 3,37248 | 2 | GGKGLGK.[594.0764297456] | H4_XENLA_Luger | TC-H3N1 | C | 4,35801
Only hits
Show advanced annotations
Save IDs
Export table
A_Stuetzer_200318_210318_monos_UV2.mzML
MI:C'+
Intensity
MZ [Th]
Log
21:14:20 NOTICE: Done
Annotation of spectra finished. Open identification view to see results!
RT: 1215.4
m/z: 3.964896

34)

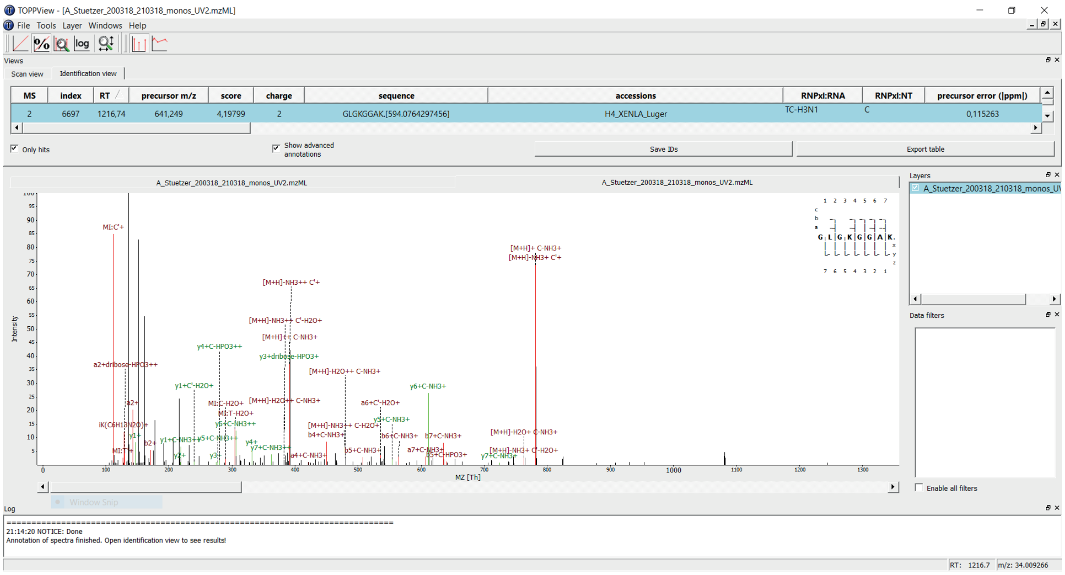
TOPPView - [A_Stuetzer_200318_210318_monos_UV2.mzML]
File Tools Layer Windows Help
Scan view
Identification view
MS index RT precursor m/z score charge sequence accessions RNPxl:RNA RNPxl:NT precursor error (|ppm|)
2 6697 1216,74 641,249 4,19799 2 GLGKGGAK.[594.0764297456] H4_XENLA_Luger TC-H3N1 C 0,115263
Only hits
Show advanced annotations
Save IDs
Export table
Layers
Data filters
Enable all filters
Intensity
MZ [Th]
Log
21:14:20 NOTICE: Done
Annotation of spectra finished. Open identification view to see results!

35)

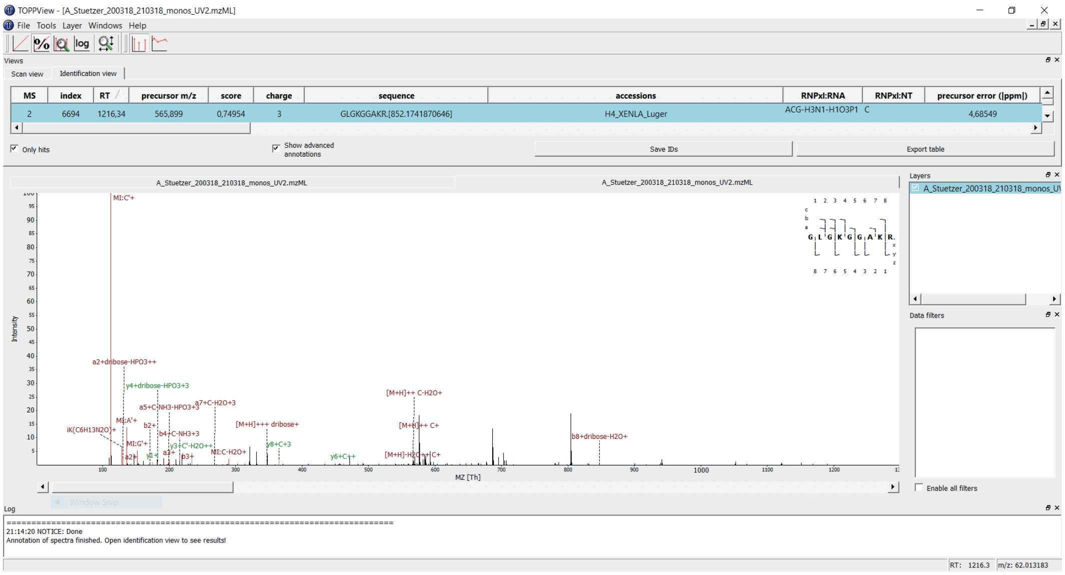
TOPPView - [A_Stuetzer_200318_210318_monos_UV2.mzML]
File Tools Layer Windows Help
Scan view
Identification view
MS index RT precursor m/z score charge sequence accessions RNPxl:RNA RNPxl:NT precursor error (|ppm|)
2 6694 1216,34 565,899 0,74954 3 GLGKGGAKR.[852.1741870646] H4_XENLA_Luger ACG-H3N1-H1O3P1 C 4,68549
Only hits
Show advanced annotations
Save IDs
Export table
Layers
Data filters
Enable all filters
Intensity
MZ [Th]
Log
21:14:20 NOTICE: Done
Annotation of spectra finished. Open identification view to see results!

36)

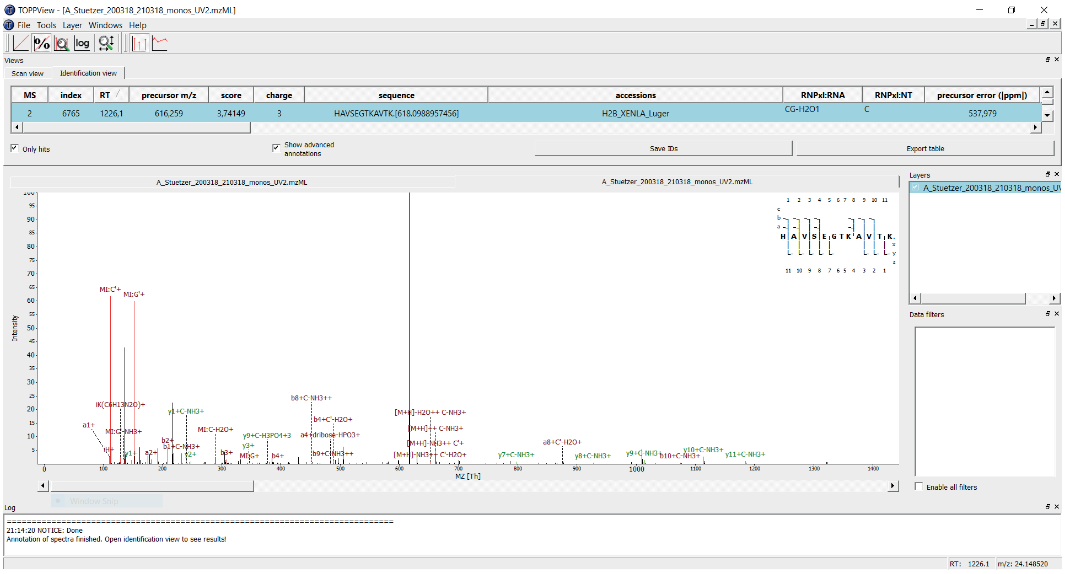
TOPPView - [A_Stuetzer_200318_210318_monos_UV2.mzML]
File Tools Layer Windows Help
Scan view
Identification view
MS index RT precursor m/z score charge sequence accessions RNPxl:RNA RNPxl:NT precursor error (|ppm|)
2 6765 1226,1 616,259 3,74149 3 HAVSEGTKAVTK.[618.0988957456] H2B_XENLA_Luger CG-H2O1 C 537,979
Only hits
Show advanced annotations
Save IDs
Export table
Layers
Data filters
Enable all filters
Intensity
MZ [Th]
Log
21:14:20 NOTICE: Done
Annotation of spectra finished. Open identification view to see results!

37)

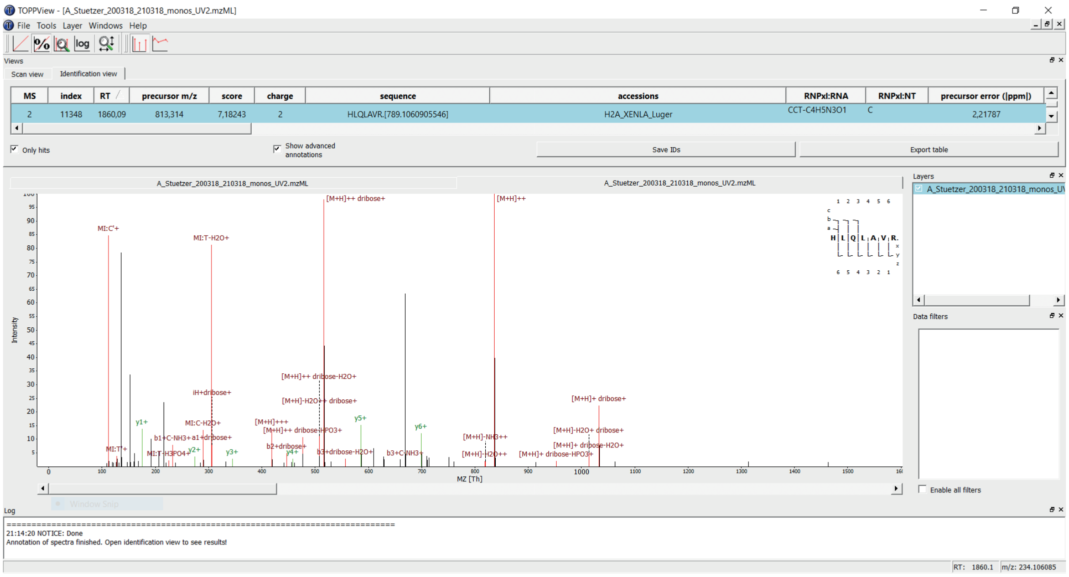
TOPPView - [A_Stuetzer_200318_210318_monos_UV2.mzML]
File Tools Layer Windows Help
Scan view Identification view
MS index RT precursor m/z score charge sequence accessions RNPxl:RNA RNPxl:NT precursor error (|ppm|)
2 11348 1860,09 813,314 7,18243 2 HLQLAVR.[789.1060905546] H2A_XENLA_Luger CCT-C4H5N3O1 C 2,21787
Only hits
Show advanced annotations
Save IDs
Export table
A_Stuetzer_200318_210318_monos_UV2.mzML
Layers
Data filters
Enable all filters
MZ [Th]
Intensity
Log
21:14:20 NOTICE: Done
Annotation of spectra finished. Open identification view to see results!
RT: 1860.1 m/z: 234.106085

38)

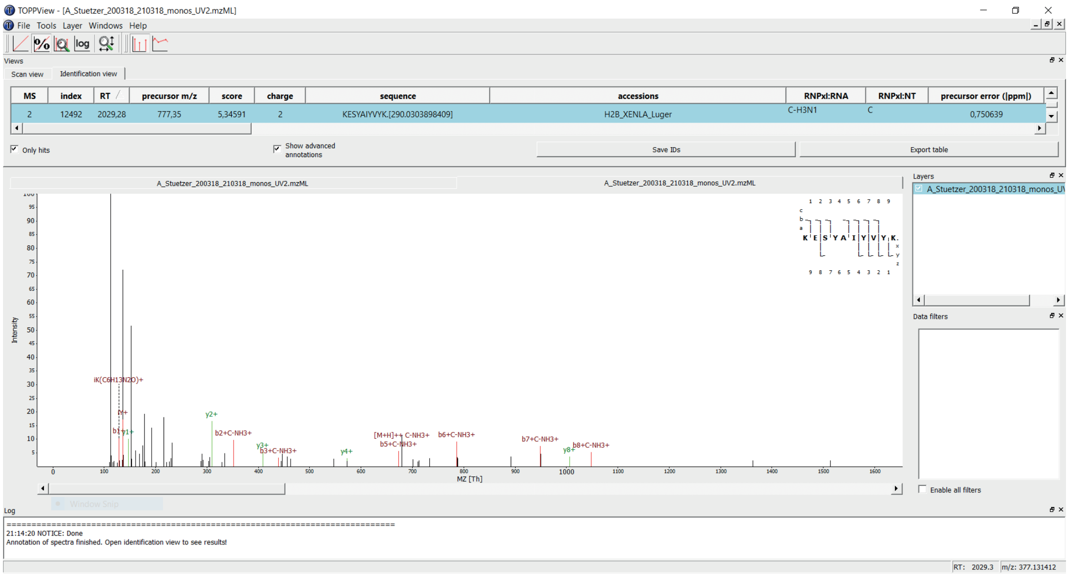
TOPPView - [A_Stuetzer_200318_210318_monos_UV2.mzML]
File Tools Layer Windows Help
Scan view Identification view
MS index RT precursor m/z score charge sequence accessions RNPxl:RNA RNPxl:NT precursor error (|ppm|)
2 12492 2029,28 777,35 5,34591 2 KESYAIYVYK.[290.0303898409] H2B_XENLA_Luger C-H3N1 C 0,750639
Only hits
Show advanced annotations
Save IDs
Export table
A_Stuetzer_200318_210318_monos_UV2.mzML
Layers
Data filters
Enable all filters
MZ [Th]
Intensity
Log
21:14:20 NOTICE: Done
Annotation of spectra finished. Open identification view to see results!
RT: 2029.3 m/z: 377.131412

39)

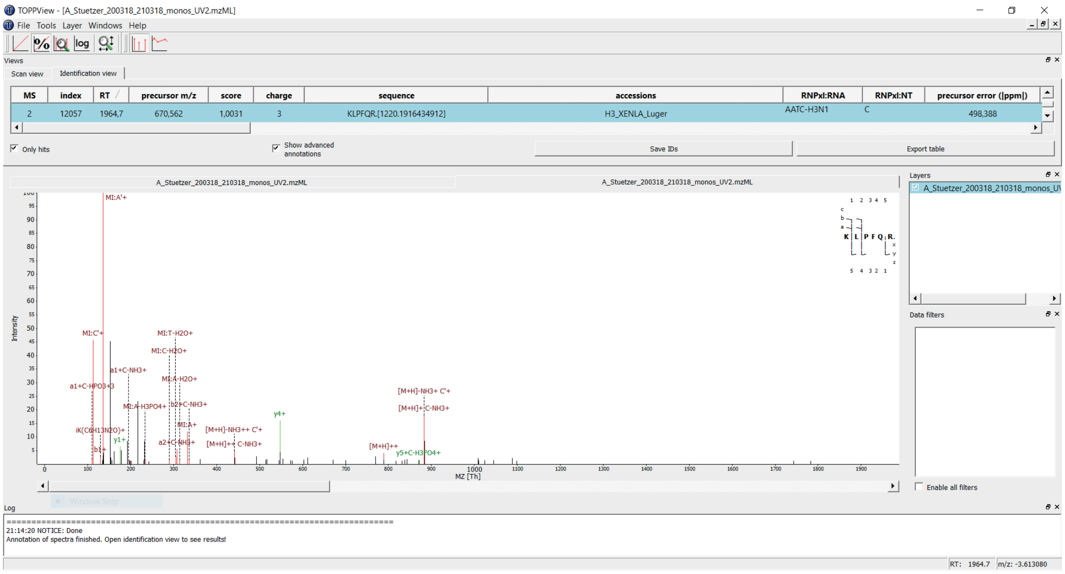
TOPPView - [A_Stuetzer_200318_210318_monos_UV2.mzML]
File Tools Layer Windows Help
Scan view Identification view
MS index RT precursor m/z score charge sequence accessions RNPxl:RNA RNPxl:NT precursor error (|ppm|)
2 12057 1964,7 670,562 1,0031 3 KLPFQR.[1220.1916434912] H3_XENLA_Luger AATC-H3N1 C 498,388
Only hits
Show advanced annotations
Save IDs
Export table
A_Stuetzer_200318_210318_monos_UV2.mzML
Layers
Data filters
Enable all filters
MZ [Th]
Intensity
Log
21:14:20 NOTICE: Done
Annotation of spectra finished. Open identification view to see results!
RT: 1964.7 m/z: -3.613080

40)

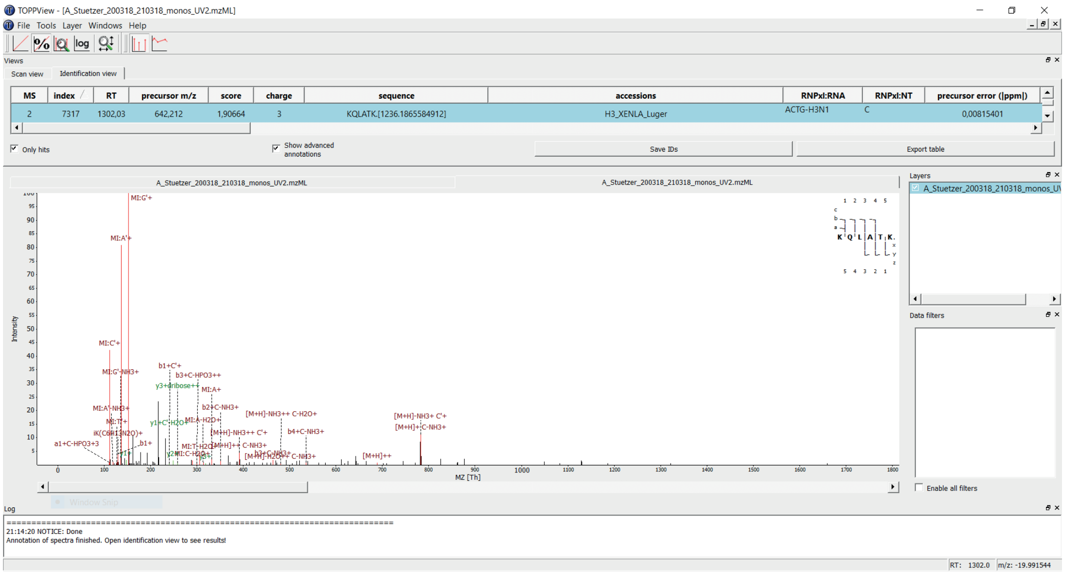
TOPPView - [A_Stuetzer_200318_210318_monos_UV2.mzML]
File Tools Layer Windows Help
Scan view Identification view
MS index RT precursor m/z score charge sequence accessions RNPxl:RNA RNPxl:NT precursor error (|ppm|)
2 7317 1302,03 642,212 1,90664 3 KQLATK.[1236.1865584912] H3_XENLA_Luger ACTG-H3N1 C 0,00815401
Only hits
Show advanced annotations
Save IDs
Export table
A_Stuetzer_200318_210318_monos_UV2.mzML
Intensity
MZ [Th]
Layers
Data filters
Enable all filters
Log
21:14:20 NOTICE: Done
Annotation of spectra finished. Open identification view to see results!
RT: 1302.0 m/z: -19.991544

41)

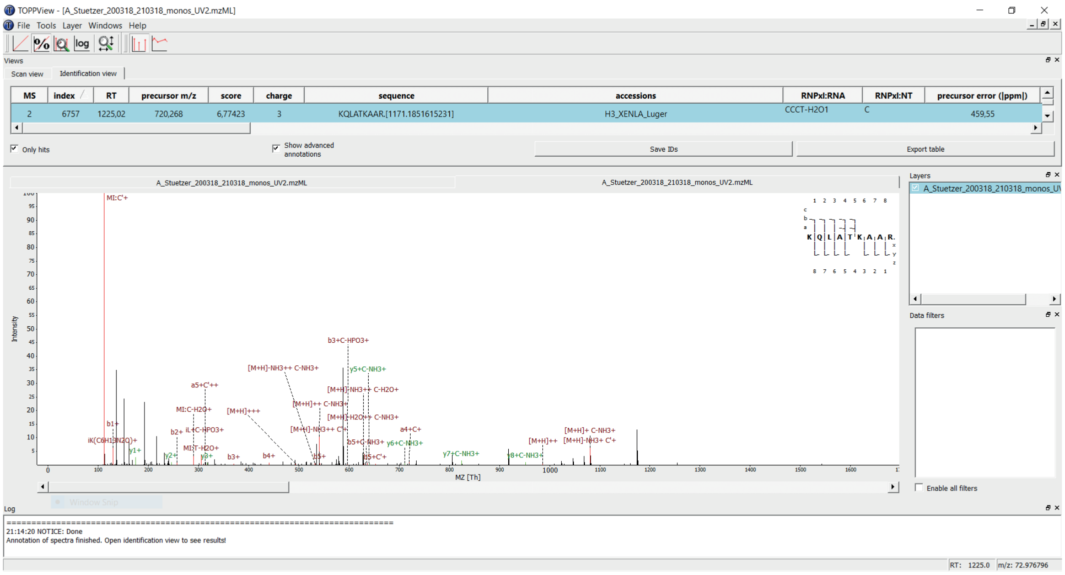
TOPPView - [A_Stuetzer_200318_210318_monos_UV2.mzML]
File Tools Layer Windows Help
Scan view Identification view
MS index RT precursor m/z score charge sequence accessions RNPxl:RNA RNPxl:NT precursor error (|ppm|)
2 6757 1225,02 720,268 6,77423 3 KQLATKAAR.[1171.1851615231] H3_XENLA_Luger CCCT-H2O1 C 459,55
Only hits
Show advanced annotations
Save IDs
Export table
A_Stuetzer_200318_210318_monos_UV2.mzML
Intensity
MZ [Th]
Layers
Data filters
Enable all filters
Log
21:14:20 NOTICE: Done
Annotation of spectra finished. Open identification view to see results!
RT: 1225.0 m/z: 72.976796

42)

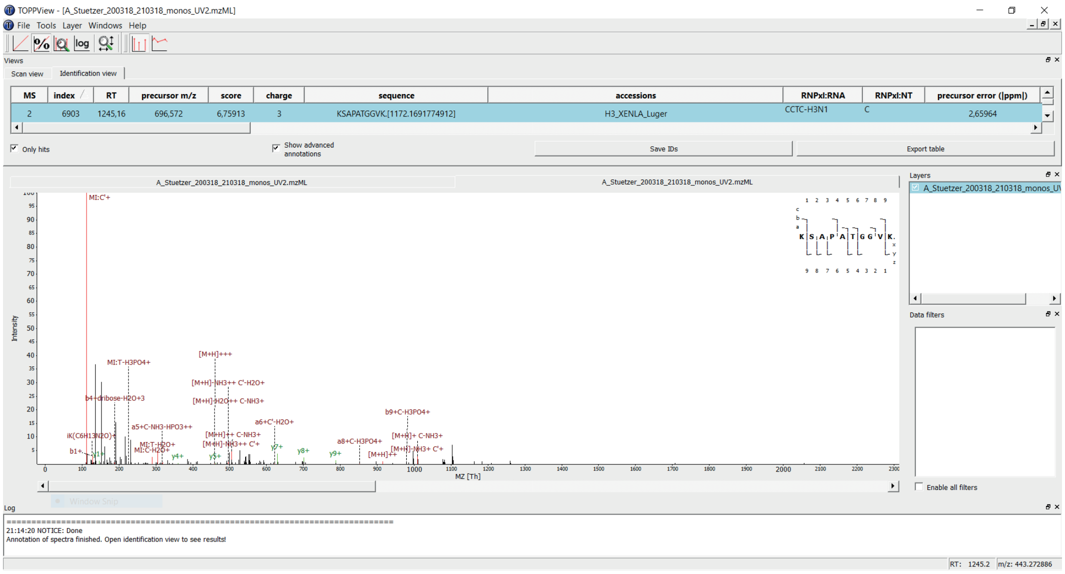
TOPPView - [A_Stuetzer_200318_210318_monos_UV2.mzML]
File Tools Layer Windows Help
Scan view Identification view
MS index RT precursor m/z score charge sequence accessions RNPxl:RNA RNPxl:NT precursor error (|ppm|)
2 6903 1245,16 696,572 6,75913 3 KSAPATGGVK.[1172.1691774912] H3_XENLA_Luger CCTC-H3N1 C 2,65964
Only hits
Show advanced annotations
Save IDs
Export table
A_Stuetzer_200318_210318_monos_UV2.mzML
Intensity
MZ [Th]
Layers
Data filters
Enable all filters
Log
21:14:20 NOTICE: Done
Annotation of spectra finished. Open identification view to see results!
RT: 1245.2 m/z: 443.272886

43)

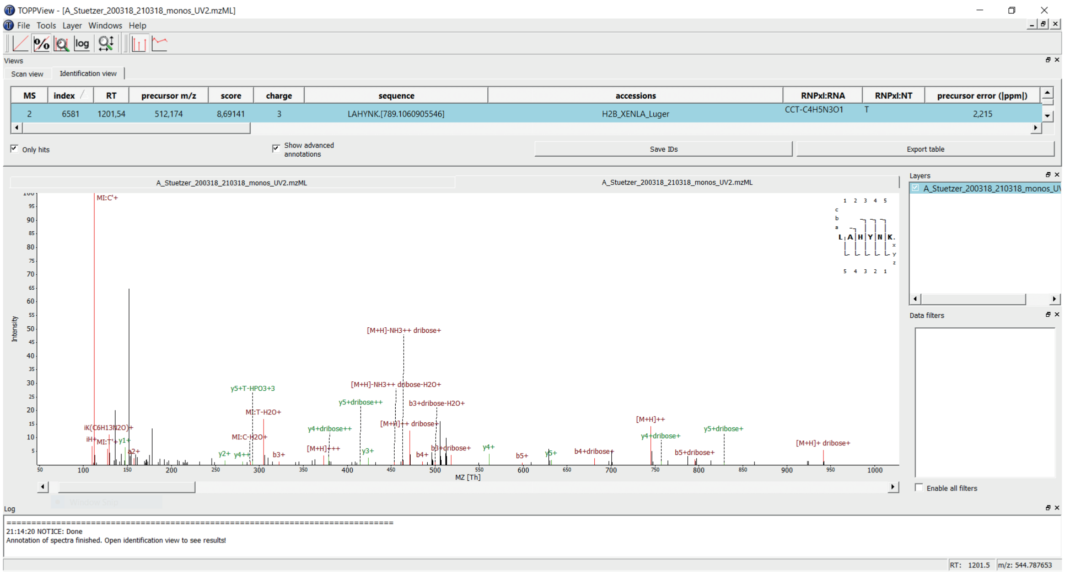
TOPPView - [A_Stuetzer_200318_210318_monos_UV2.mzML]
MS | index | RT | precursor m/z | score | charge | sequence | accessions | RNPxl:RNA | RNPxl:NT | precursor error (|ppm|)
2 | 6581 | 1201,54 | 512,174 | 8,69141 | 3 | LAHYNK.[789.1060905546] | H2B_XENLA_Luger | CCT-C4H5N3O1 | T | 2,215
21:14:20 NOTICE: Done
Annotation of spectra finished. Open identification view to see results!
RT: 1201.5 m/z: 544.787653

44)

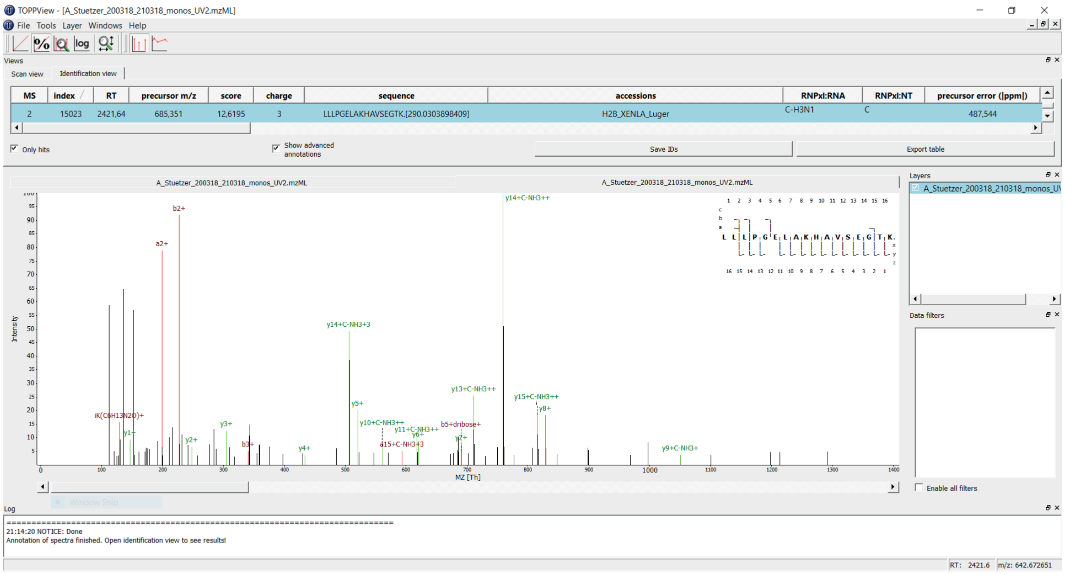
TOPPView - [A_Stuetzer_200318_210318_monos_UV2.mzML]
MS | index | RT | precursor m/z | score | charge | sequence | accessions | RNPxl:RNA | RNPxl:NT | precursor error (|ppm|)
2 | 15023 | 2421,64 | 685,351 | 12,6195 | 3 | LLLPGELAKHAVSEGTK.[290.0303898409] | H2B_XENLA_Luger | C-H3N1 | C | 487,544
21:14:20 NOTICE: Done
Annotation of spectra finished. Open identification view to see results!
RT: 2421.6 m/z: 642.672651

45)

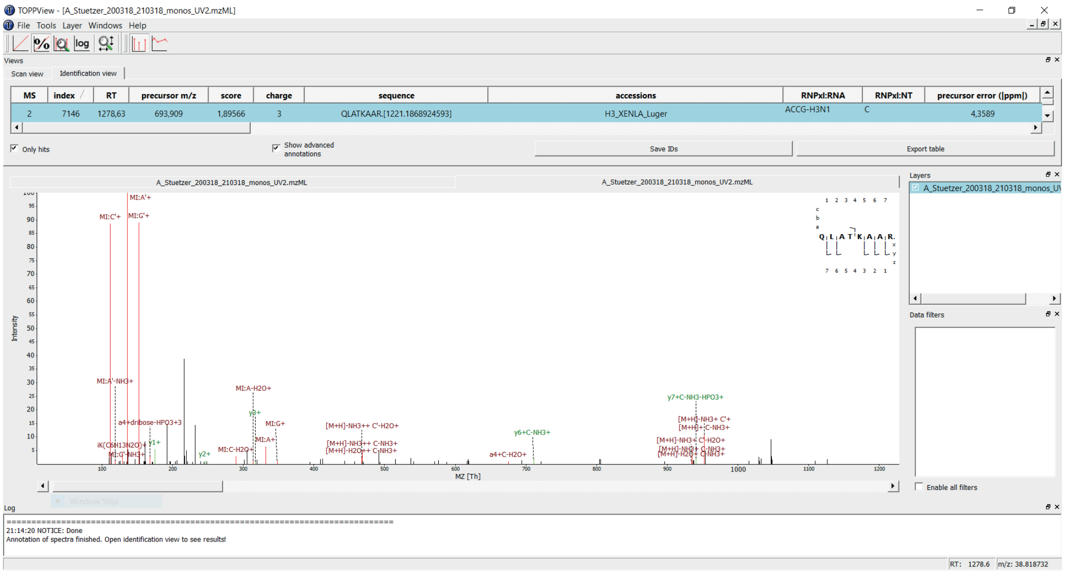
TOPPView - [A_Stuetzer_200318_210318_monos_UV2.mzML]
MS | index | RT | precursor m/z | score | charge | sequence | accessions | RNPxl:RNA | RNPxl:NT | precursor error (|ppm|)
2 | 7146 | 1278,63 | 693,909 | 1,89566 | 3 | QLATKAAR.[1221.1868924593] | H3_XENLA_Luger | ACCG-H3N1 | C | 4,3589
21:14:20 NOTICE: Done
Annotation of spectra finished. Open identification view to see results!
RT: 1278.6 m/z: 38.818732

46)

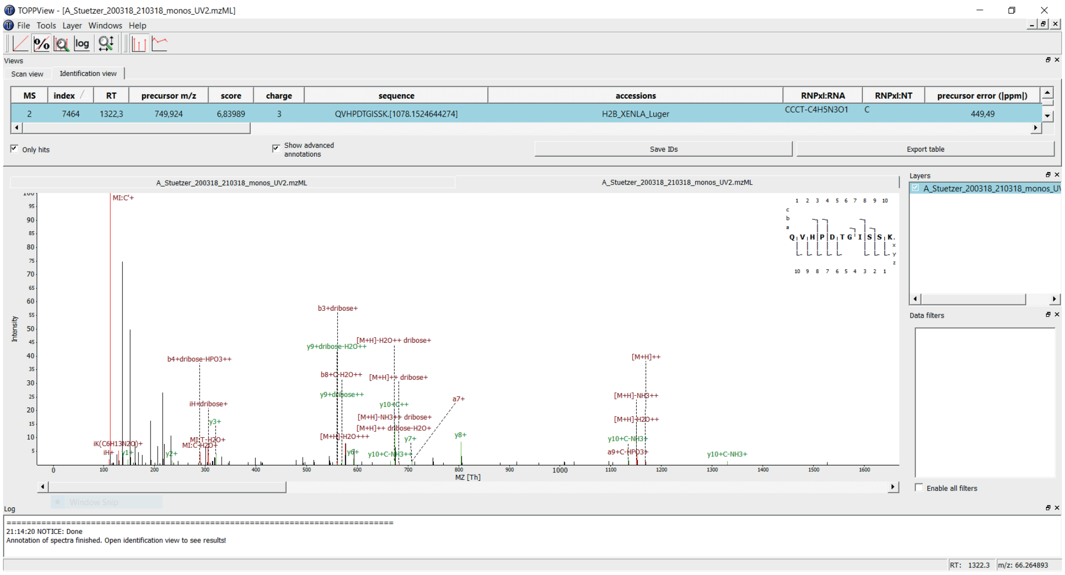
TOPPView - [A_Stuetzer_200318_210318_monos_UV2.mzML]
File Tools Layer Windows Help
Scan view
Identification view
MS
index
RT
precursor m/z
score
charge
sequence
accessions
RNPxl:RNA
RNPxl:NT
precursor error (|ppm|)
2
7464
1322,3
749,924
6,83989
3
QVHPDTGISSK.[1078.1524644274]
H2B_XENLA_Luger
CCCT-C4H5N3O1
C
449,49
Only hits
Show advanced annotations
Save IDs
Export table
A_Stuetzer_200318_210318_monos_UV2.mzML
Layers
Data filters
Enable all filters
MZ [Th]
Intensity
21:14:20 NOTICE: Done
Annotation of spectra finished. Open identification view to see results!
RT: 1322.3
m/z: 66.264893

47)

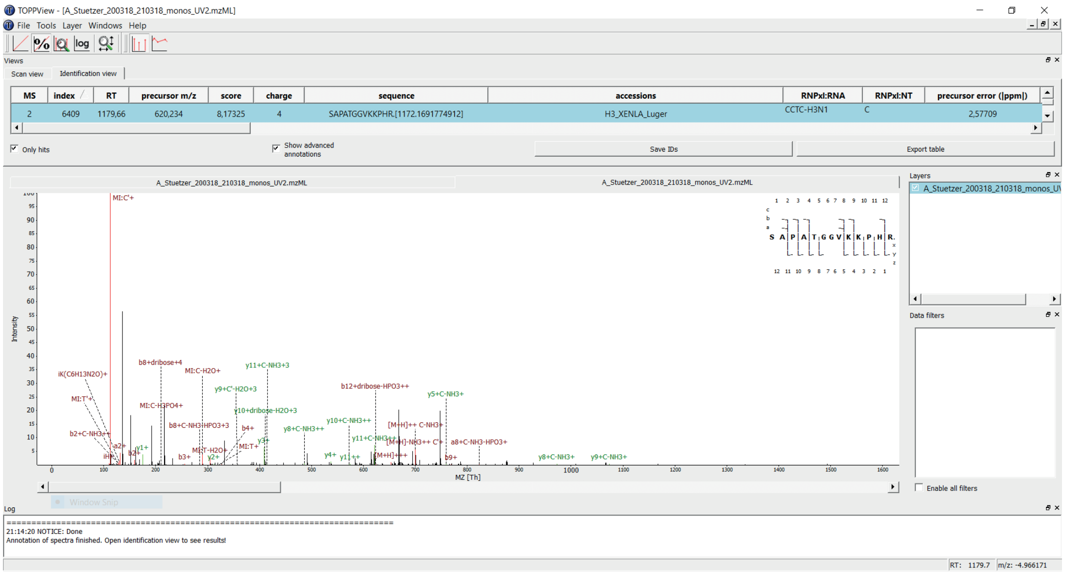
TOPPView - [A_Stuetzer_200318_210318_monos_UV2.mzML]
File Tools Layer Windows Help
Scan view
Identification view
MS
index
RT
precursor m/z
score
charge
sequence
accessions
RNPxl:RNA
RNPxl:NT
precursor error (|ppm|)
2
6409
1179,66
620,234
8,17325
4
SAPATGGVKKPHR.[1172.1691774912]
H3_XENLA_Luger
CCTC-H3N1
C
2,57709
Only hits
Show advanced annotations
Save IDs
Export table
A_Stuetzer_200318_210318_monos_UV2.mzML
Layers
Data filters
Enable all filters
MZ [Th]
Intensity
21:14:20 NOTICE: Done
Annotation of spectra finished. Open identification view to see results!
RT: 1179.7
m/z: -4.966171

48)

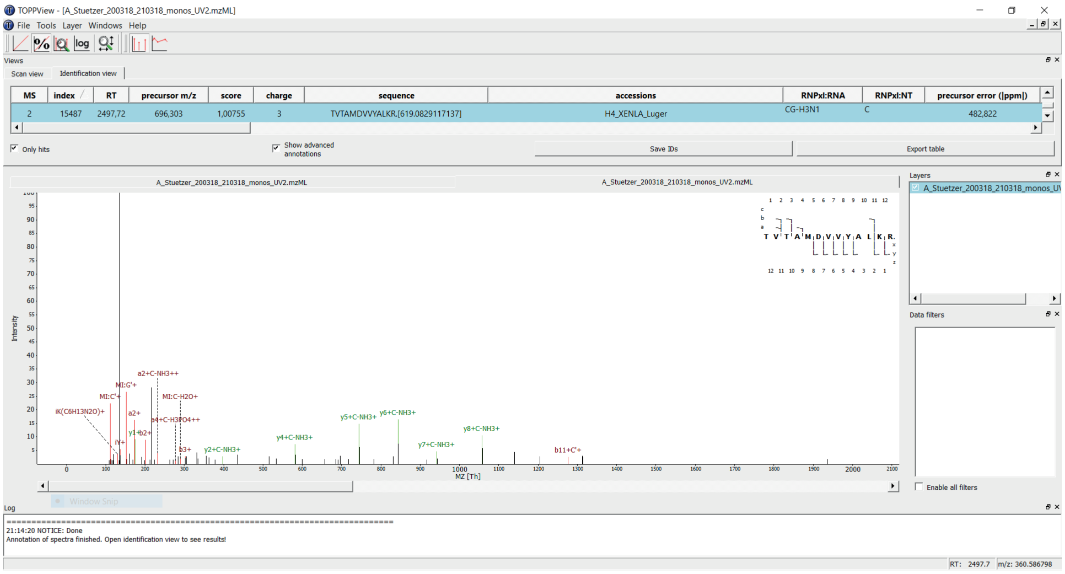
TOPPView - [A_Stuetzer_200318_210318_monos_UV2.mzML]
File Tools Layer Windows Help
Scan view
Identification view
MS
index
RT
precursor m/z
score
charge
sequence
accessions
RNPxl:RNA
RNPxl:NT
precursor error (|ppm|)
2
15487
2497,72
696,303
1,00755
3
TVTAMDVVYALKR.[619.0829117137]
H4_XENLA_Luger
CG-H3N1
C
482,822
Only hits
Show advanced annotations
Save IDs
Export table
A_Stuetzer_200318_210318_monos_UV2.mzML
Layers
Data filters
Enable all filters
MZ [Th]
Intensity
21:14:20 NOTICE: Done
Annotation of spectra finished. Open identification view to see results!
RT: 2497.7
m/z: 360.586798

49)

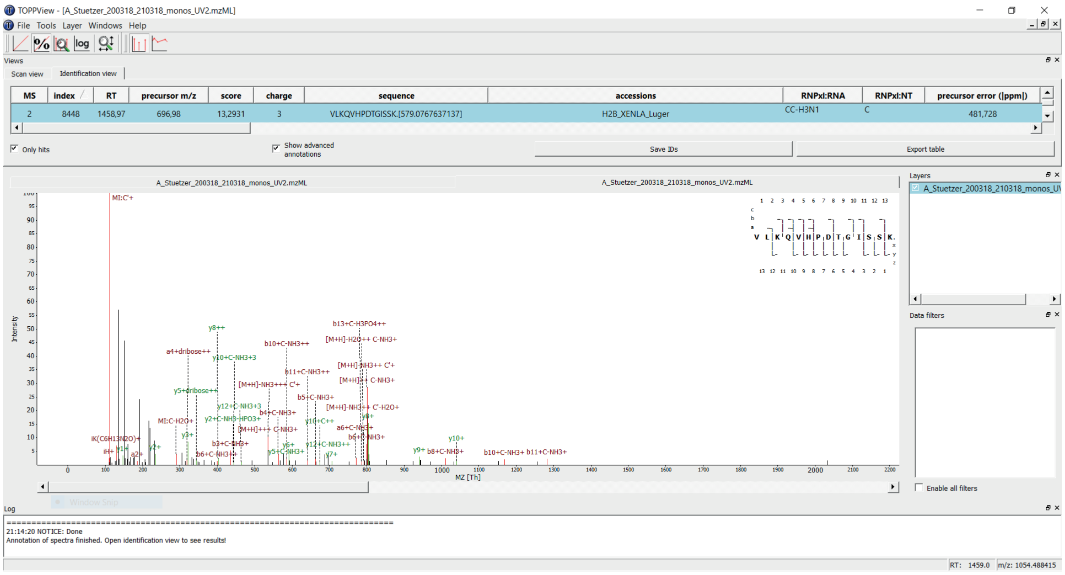
TOPPView - [A_Stuetzer_200318_210318_monos_UV2.mzML]
MS index RT precursor m/z score charge sequence accessions RNPxl:RNA RNPxl:NT precursor error (|ppm|)
2 8448 1458,97 696,98 13,2931 3 VLKQVHPDTGISSK.[579.0767637137] H2B_XENLA_Luger CC-H3N1 C 481,728
21:14:20 NOTICE: Done
Annotation of spectra finished. Open identification view to see results!

50)

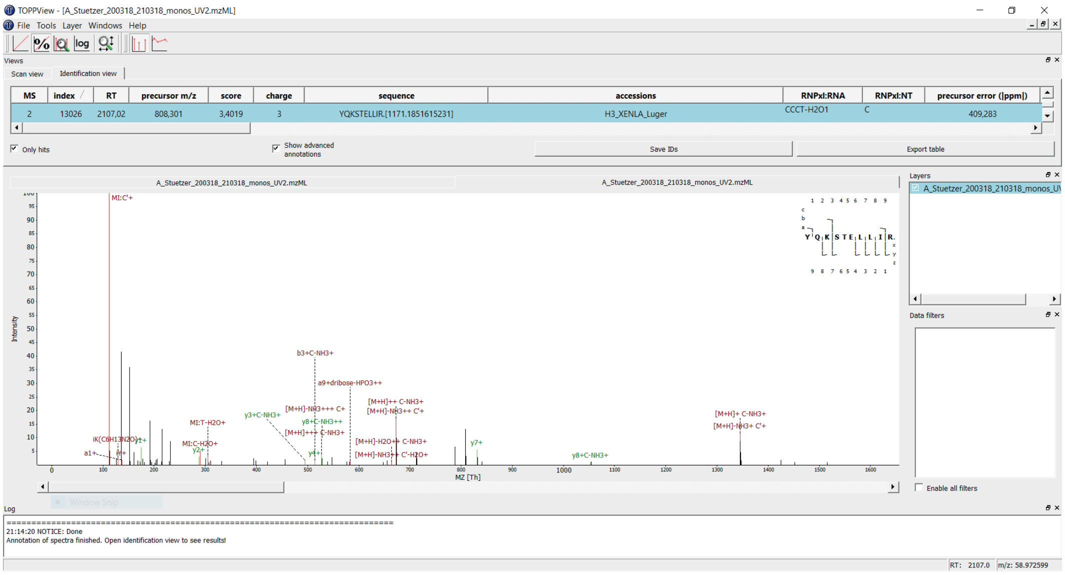
TOPPView - [A_Stuetzer_200318_210318_monos_UV2.mzML]
MS index RT precursor m/z score charge sequence accessions RNPxl:RNA RNPxl:NT precursor error (|ppm|)
2 13026 2107,02 808,301 3,4019 3 YQKSTELLIR.[1171.1851615231] H3_XENLA_Luger CCCT-H2O1 C 409,283
21:14:20 NOTICE: Done
Annotation of spectra finished. Open identification view to see results!

51)

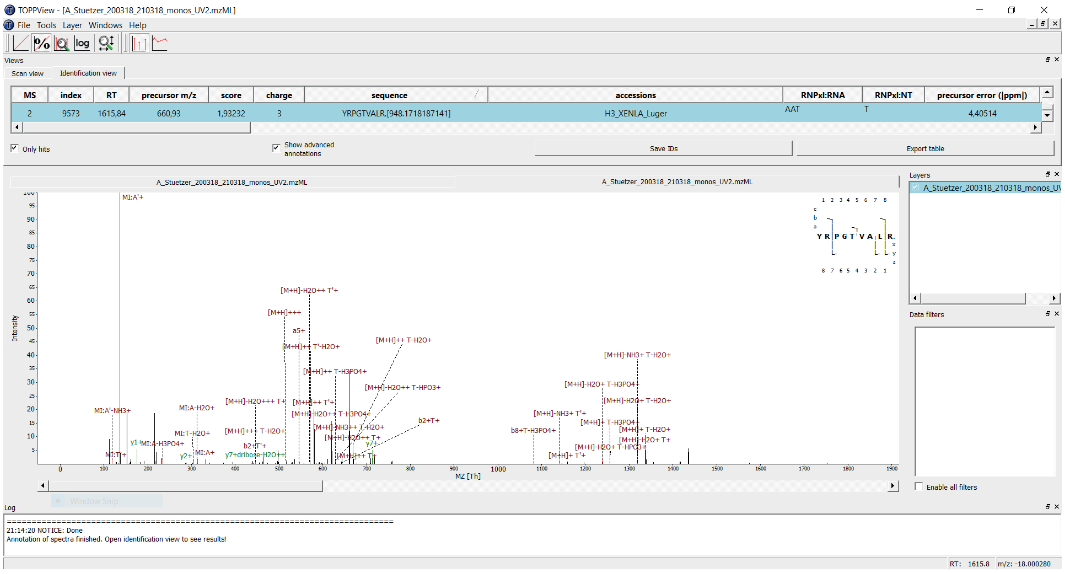
TOPPView - [A_Stuetzer_200318_210318_monos_UV2.mzML]
MS index RT precursor m/z score charge sequence accessions RNPxl:RNA RNPxl:NT precursor error (|ppm|)
2 9573 1615,84 660,93 1,93232 3 YRPGTVALR.[948.1718187141] H3_XENLA_Luger AAT T 4,40514
21:14:20 NOTICE: Done
Annotation of spectra finished. Open identification view to see results!

TOPPView MS/MS spectra of core histones from *X. laevis* nucleosomal arrays

1)

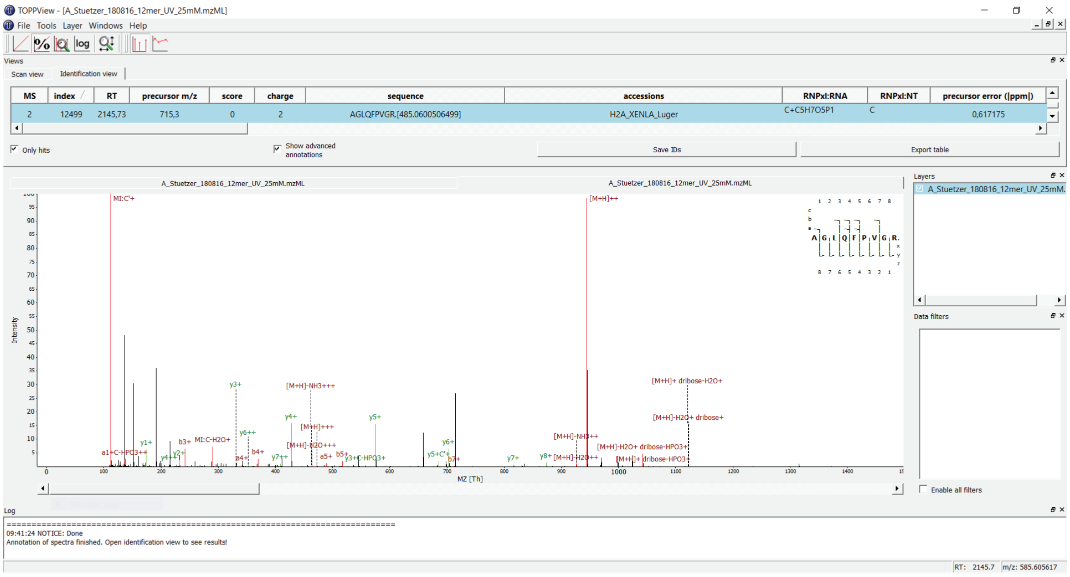

2)

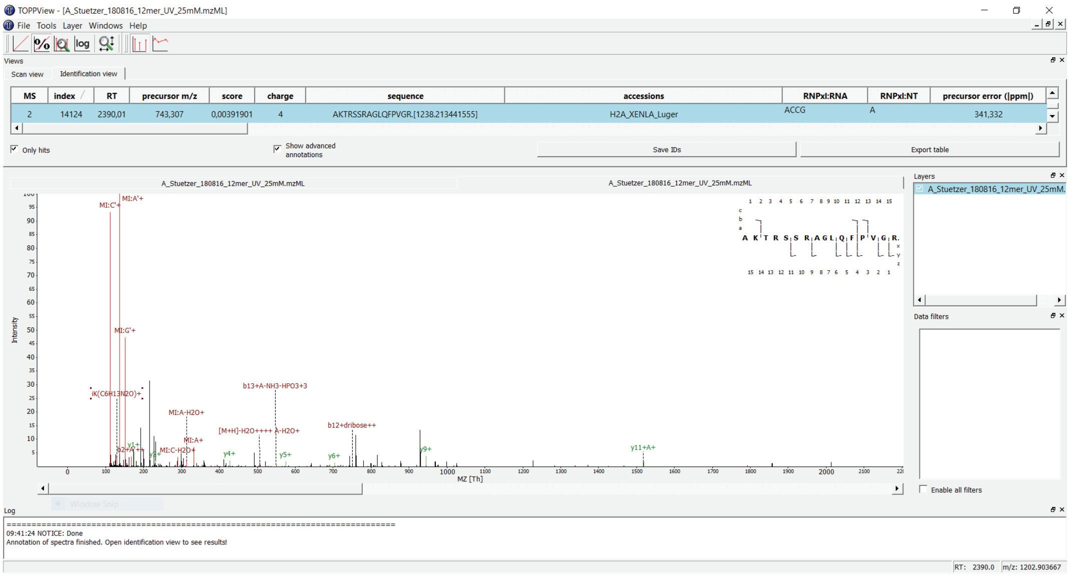

3)

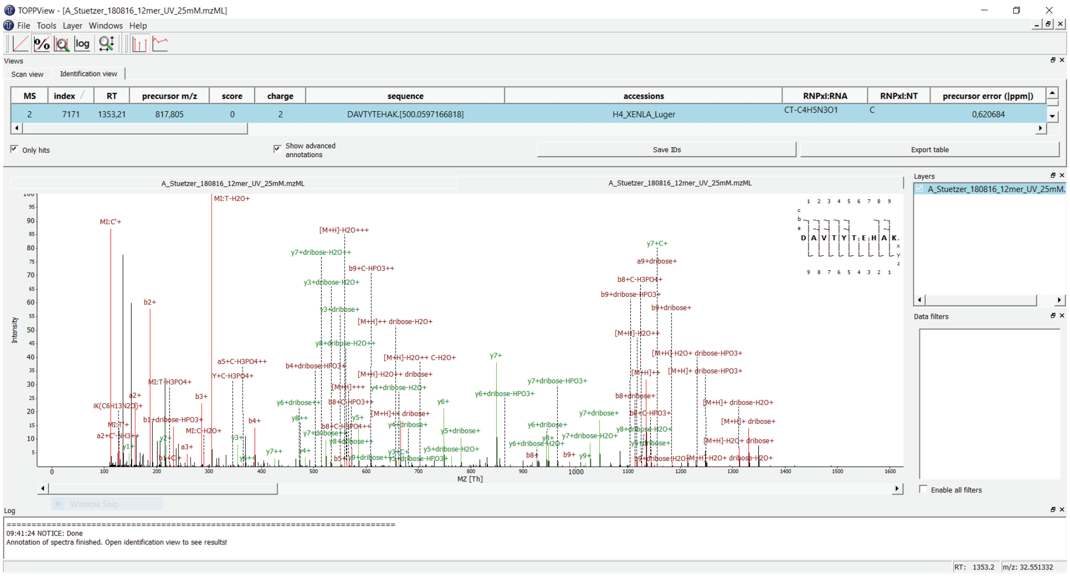

4)

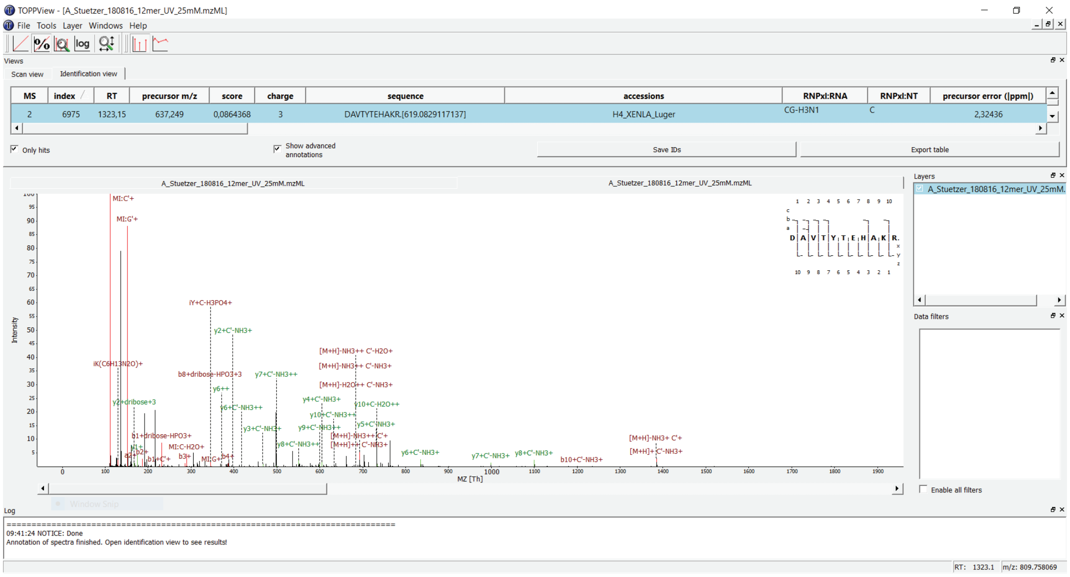
TOPPView - [A_Stuetzer_180816_12mer_UV_25mM.mzML]
File Tools Layer Windows Help
Scan view Identification view
MS | index | RT | precursor m/z | score | charge | sequence | accessions | RNPxl:RNA | RNPxl:NT | precursor error (|ppm|)
2 | 6975 | 1323,15 | 637,249 | 0,0864368 | 3 | DAVTYTEHAKR.[619.0829117137] | H4_XENLA_Luger | CG-H3N1 | C | 2,32436
Only hits
Show advanced annotations
Save IDs
Export table
A_Stuetzer_180816_12mer_UV_25mM.mzML
Layers
Data filters
Enable all filters
MZ [Th]
Intensity
09:41:24 NOTICE: Done
Annotation of spectra finished. Open identification view to see results!
RT: 1323.1 m/z: 809.758069

5)

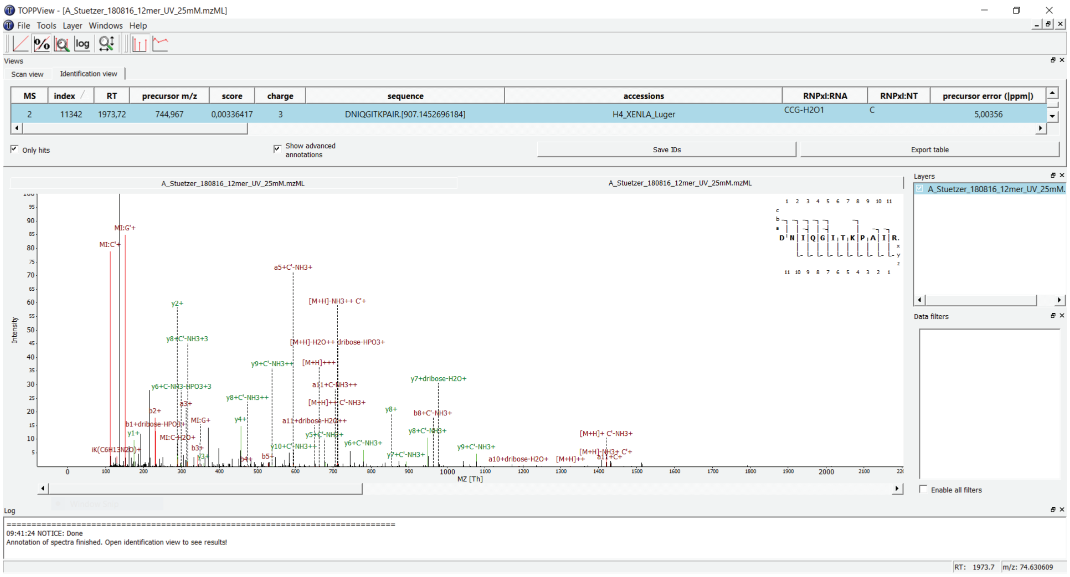
TOPPView - [A_Stuetzer_180816_12mer_UV_25mM.mzML]
File Tools Layer Windows Help
Scan view Identification view
MS | index | RT | precursor m/z | score | charge | sequence | accessions | RNPxl:RNA | RNPxl:NT | precursor error (|ppm|)
2 | 11342 | 1973,72 | 744,967 | 0,00336417 | 3 | DNIQGITKPAIR.[907.1452696184] | H4_XENLA_Luger | CCG-H2O1 | C | 5,00356
Only hits
Show advanced annotations
Save IDs
Export table
A_Stuetzer_180816_12mer_UV_25mM.mzML
Layers
Data filters
Enable all filters
MZ [Th]
Intensity
09:41:24 NOTICE: Done
Annotation of spectra finished. Open identification view to see results!
RT: 1973.7 m/z: 74.630609

6)

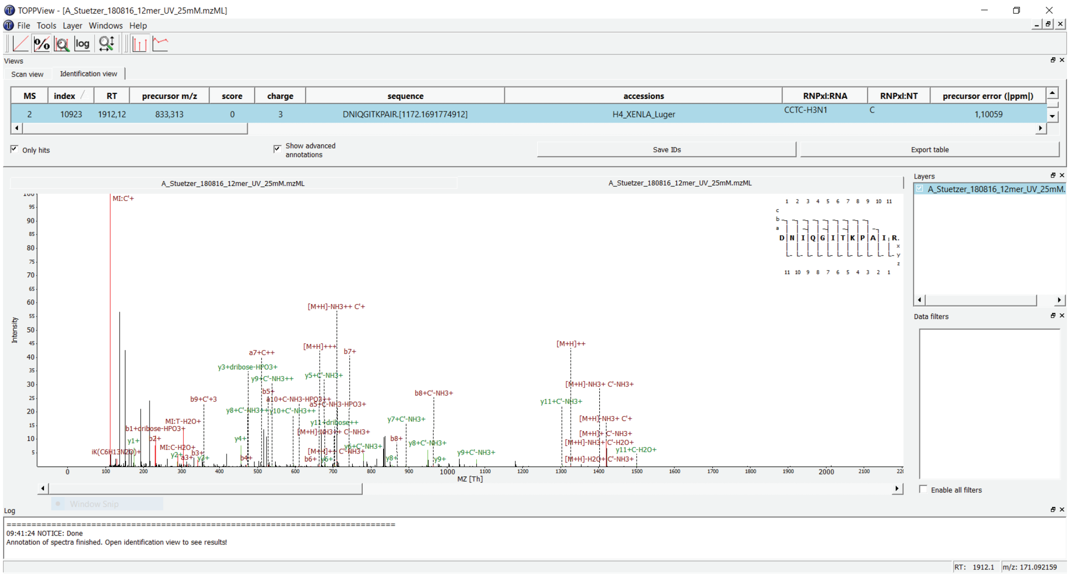
TOPPView - [A_Stuetzer_180816_12mer_UV_25mM.mzML]
File Tools Layer Windows Help
Scan view Identification view
MS | index | RT | precursor m/z | score | charge | sequence | accessions | RNPxl:RNA | RNPxl:NT | precursor error (|ppm|)
2 | 10923 | 1912,12 | 833,313 | 0 | 3 | DNIQGITKPAIR.[1172.1691774912] | H4_XENLA_Luger | CCTC-H3N1 | C | 1,10059
Only hits
Show advanced annotations
Save IDs
Export table
A_Stuetzer_180816_12mer_UV_25mM.mzML
Layers
Data filters
Enable all filters
MZ [Th]
Intensity
09:41:24 NOTICE: Done
Annotation of spectra finished. Open identification view to see results!
RT: 1912.1 m/z: 171.092159

7)

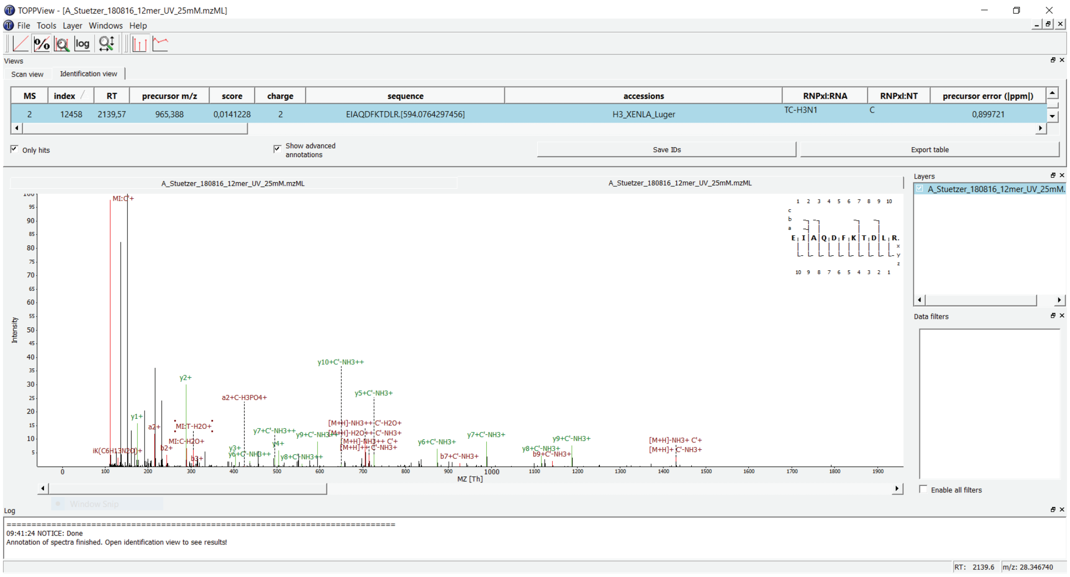
TOPPView - [A_Stuetzer_180816_12mer_UV_25mM.mzML]
File Tools Layer Windows Help
Scan view
Identification view
MS | index | RT | precursor m/z | score | charge | sequence | accessions | RNPxl:RNA | RNPxl:NT | precursor error (|ppm|)
2 | 12458 | 2139,57 | 965,388 | 0,0141228 | 2 | EIAQDFKTDLR.[594.0764297456] | H3_XENLA_Luger | TC-H3N1 | C | 0,899721
Only hits
Show advanced annotations
Save IDs
Export table
A_Stuetzer_180816_12mer_UV_25mM.mzML
Layers
Data filters
Enable all filters
Intensity
MZ [Th]
09:41:24 NOTICE: Done
Annotation of spectra finished. Open identification view to see results!
RT: 2139.6 m/z: 28.346740

8)

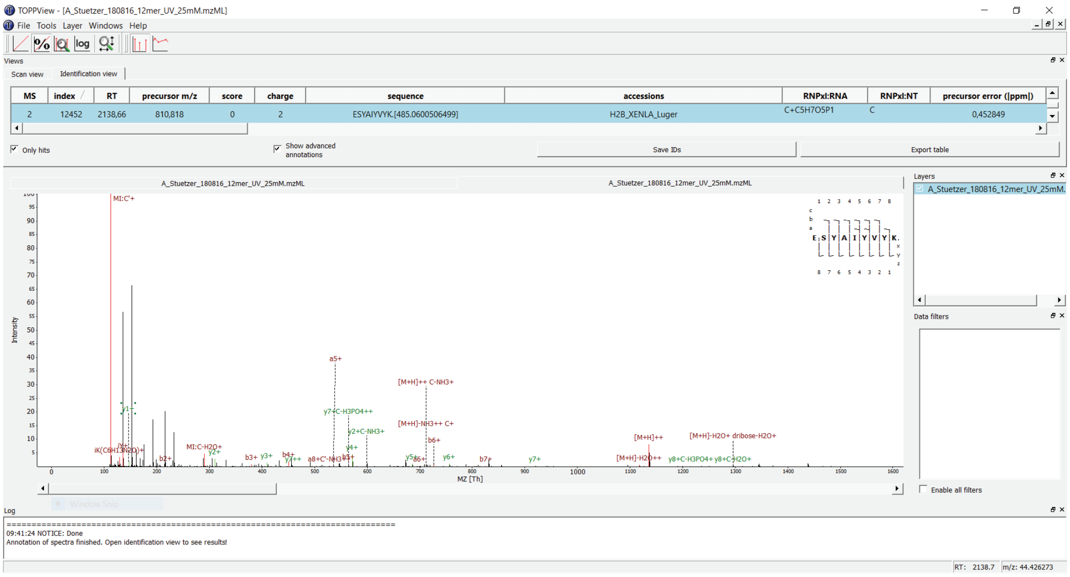
TOPPView - [A_Stuetzer_180816_12mer_UV_25mM.mzML]
File Tools Layer Windows Help
Scan view
Identification view
MS | index | RT | precursor m/z | score | charge | sequence | accessions | RNPxl:RNA | RNPxl:NT | precursor error (|ppm|)
2 | 12452 | 2138,66 | 810,818 | 0 | 2 | ESYAIYVYK.[485.0600506499] | H2B_XENLA_Luger | C+C5H7O5P1 | C | 0,452849
Only hits
Show advanced annotations
Save IDs
Export table
A_Stuetzer_180816_12mer_UV_25mM.mzML
Layers
Data filters
Enable all filters
Intensity
MZ [Th]
09:41:24 NOTICE: Done
Annotation of spectra finished. Open identification view to see results!
RT: 2138.7 m/z: 44.426273

9)

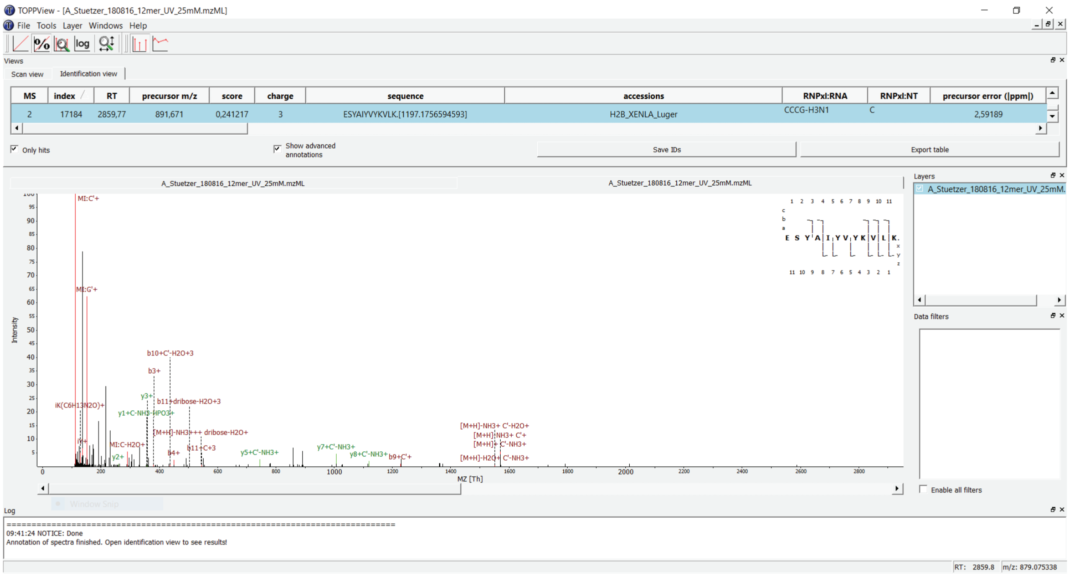
TOPPView - [A_Stuetzer_180816_12mer_UV_25mM.mzML]
File Tools Layer Windows Help
Scan view
Identification view
MS | index | RT | precursor m/z | score | charge | sequence | accessions | RNPxl:RNA | RNPxl:NT | precursor error (|ppm|)
2 | 17184 | 2859,77 | 891,671 | 0,241217 | 3 | ESYAIYVYKVLK.[1197.1756594593] | H2B_XENLA_Luger | CCCG-H3N1 | C | 2,59189
Only hits
Show advanced annotations
Save IDs
Export table
A_Stuetzer_180816_12mer_UV_25mM.mzML
Layers
Data filters
Enable all filters
Intensity
MZ [Th]
09:41:24 NOTICE: Done
Annotation of spectra finished. Open identification view to see results!
RT: 2859.8 m/z: 879.075338

10)

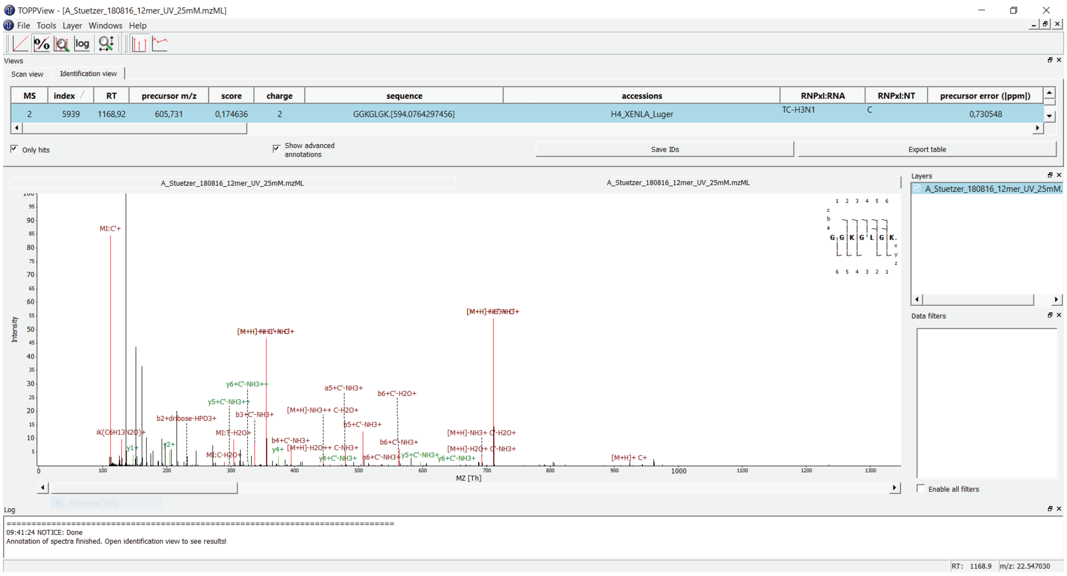

TOPPView - [A_Stuetzer_180816_12mer_UV_25mM.mzML]
File Tools Layer Windows Help
Scan view
Identification view
MS | index | RT | precursor m/z | score | charge | sequence | accessions | RNPxl:RNA | RNPxl:NT | precursor error (|ppm|)
2 | 5939 | 1168,92 | 605,731 | 0,174636 | 2 | GGKGLGK.[594.0764297456] | H4_XENLA_Luger | TC-H3N1 | C | 0,730548
Only hits
Show advanced annotations
Save IDs
Export table
A_Stuetzer_180816_12mer_UV_25mM.mzML
Layers
Data filters
Enable all filters
MZ [Th]
Intensity
09:41:24 NOTICE: Done
Annotation of spectra finished. Open identification view to see results!
RT: 1168.9
m/z: 22.547030

11)

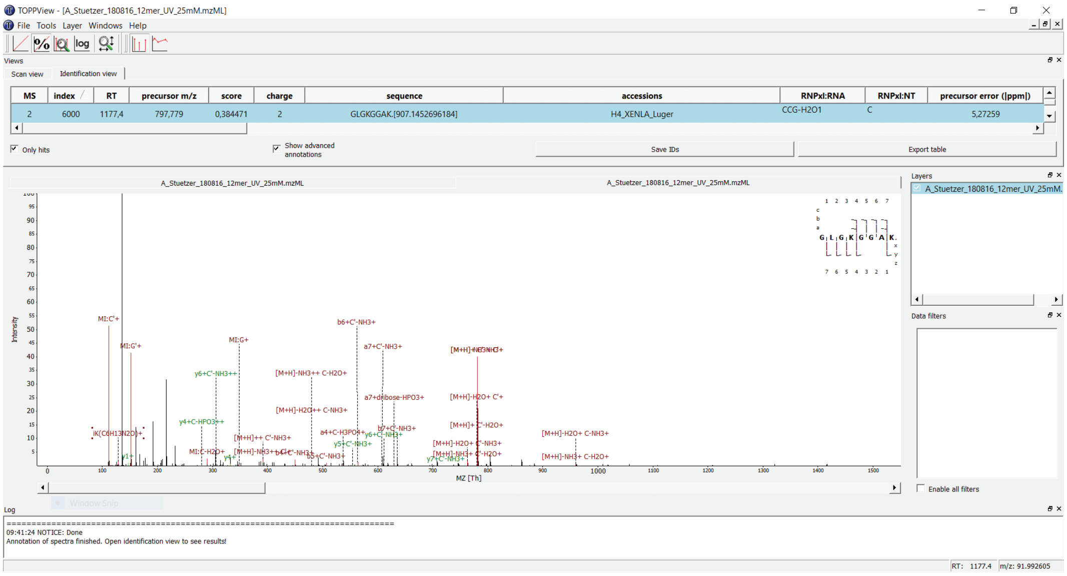

TOPPView - [A_Stuetzer_180816_12mer_UV_25mM.mzML]
File Tools Layer Windows Help
Scan view
Identification view
MS | index | RT | precursor m/z | score | charge | sequence | accessions | RNPxl:RNA | RNPxl:NT | precursor error (|ppm|)
2 | 6000 | 1177,4 | 797,779 | 0,384471 | 2 | GLGKGGAK.[907.1452696184] | H4_XENLA_Luger | CCG-H2O1 | C | 5,27259
Only hits
Show advanced annotations
Save IDs
Export table
A_Stuetzer_180816_12mer_UV_25mM.mzML
Layers
Data filters
Enable all filters
MZ [Th]
Intensity
09:41:24 NOTICE: Done
Annotation of spectra finished. Open identification view to see results!
RT: 1177.4
m/z: 91.992605

12)

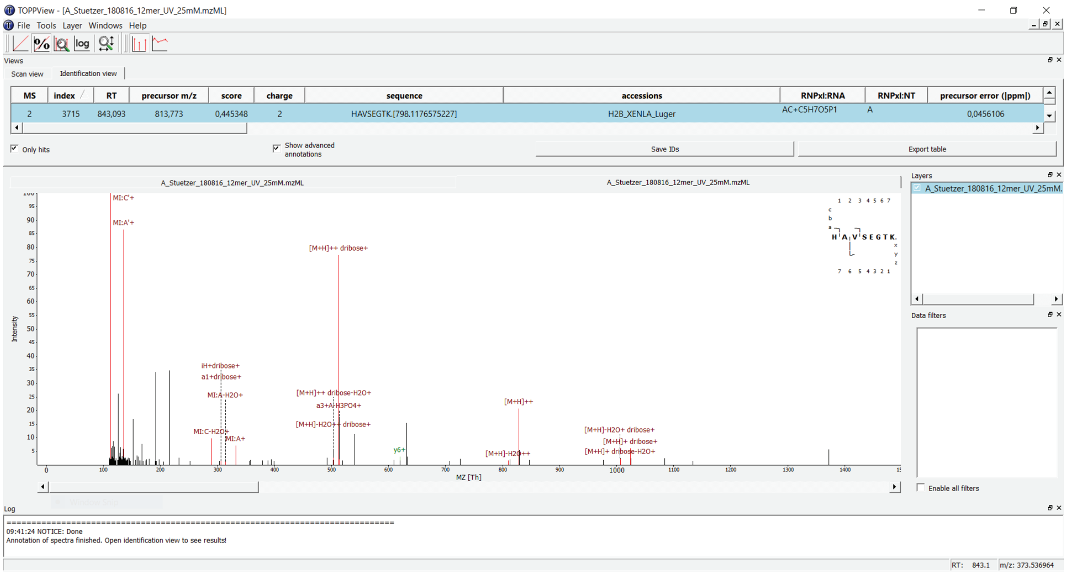

TOPPView - [A_Stuetzer_180816_12mer_UV_25mM.mzML]
File Tools Layer Windows Help
Scan view
Identification view
MS | index | RT | precursor m/z | score | charge | sequence | accessions | RNPxl:RNA | RNPxl:NT | precursor error (|ppm|)
2 | 3715 | 843,093 | 813,773 | 0,445348 | 2 | HAVSEGTK.[798.1176575227] | H2B_XENLA_Luger | AC+C5H7O5P1 | A | 0,0456106
Only hits
Show advanced annotations
Save IDs
Export table
A_Stuetzer_180816_12mer_UV_25mM.mzML
Layers
Data filters
Enable all filters
MZ [Th]
Intensity
09:41:24 NOTICE: Done
Annotation of spectra finished. Open identification view to see results!
RT: 843.1
m/z: 373.536964

13)

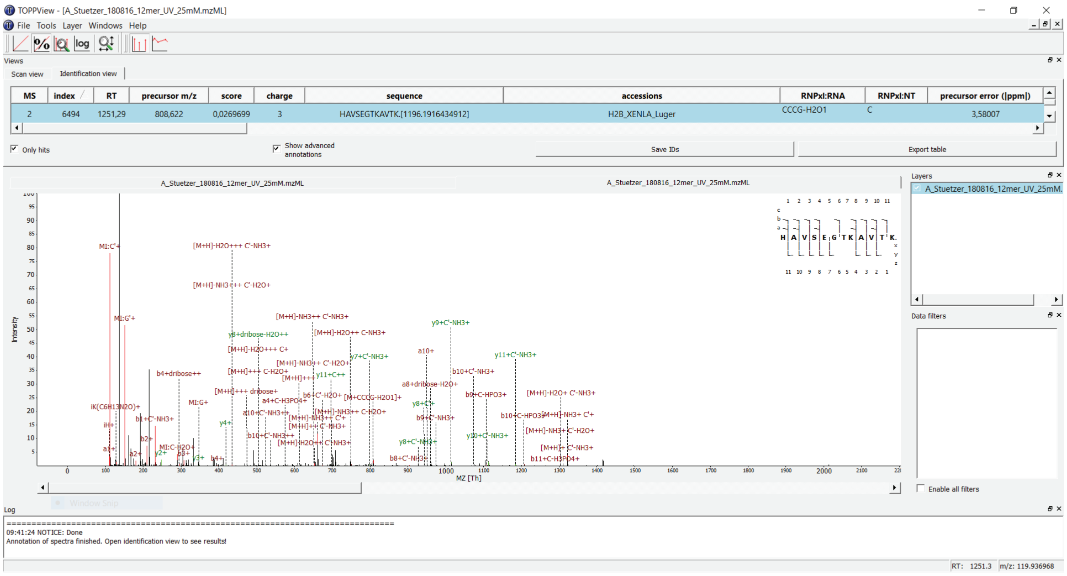
TOPPView - [A_Stuetzer_180816_12mer_UV_25mM.mzML]
File Tools Layer Windows Help
Scan view
Identification view
MS | index | RT | precursor m/z | score | charge | sequence | accessions | RNPxl:RNA | RNPxl:NT | precursor error (|ppm|)
2 | 6494 | 1251,29 | 808,622 | 0,0269699 | 3 | HAVSEGTKAVTK.[1196.1916434912] | H2B_XENLA_Luger | CCCG-H2O1 | C | 3,58007
Only hits
Show advanced annotations
Save IDs
Export table
A_Stuetzer_180816_12mer_UV_25mM.mzML
Layers
Data filters
Enable all filters
Intensity
MZ [Th]
09:41:24 NOTICE: Done
Annotation of spectra finished. Open identification view to see results!
RT: 1251.3 m/z: 119.936968

14)

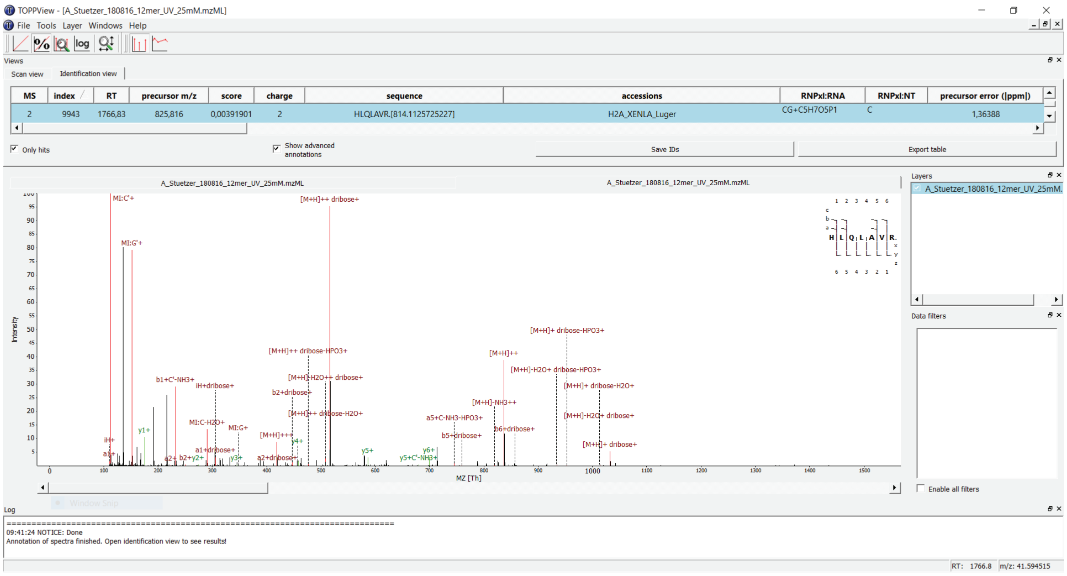
TOPPView - [A_Stuetzer_180816_12mer_UV_25mM.mzML]
File Tools Layer Windows Help
Scan view
Identification view
MS | index | RT | precursor m/z | score | charge | sequence | accessions | RNPxl:RNA | RNPxl:NT | precursor error (|ppm|)
2 | 9943 | 1766,83 | 825,816 | 0,00391901 | 2 | HLQLAVR.[814.1125725227] | H2A_XENLA_Luger | CG+C5H7O5P1 | C | 1,36388
Only hits
Show advanced annotations
Save IDs
Export table
A_Stuetzer_180816_12mer_UV_25mM.mzML
Layers
Data filters
Enable all filters
Intensity
MZ [Th]
09:41:24 NOTICE: Done
Annotation of spectra finished. Open identification view to see results!
RT: 1766.8 m/z: 41.594515

15)

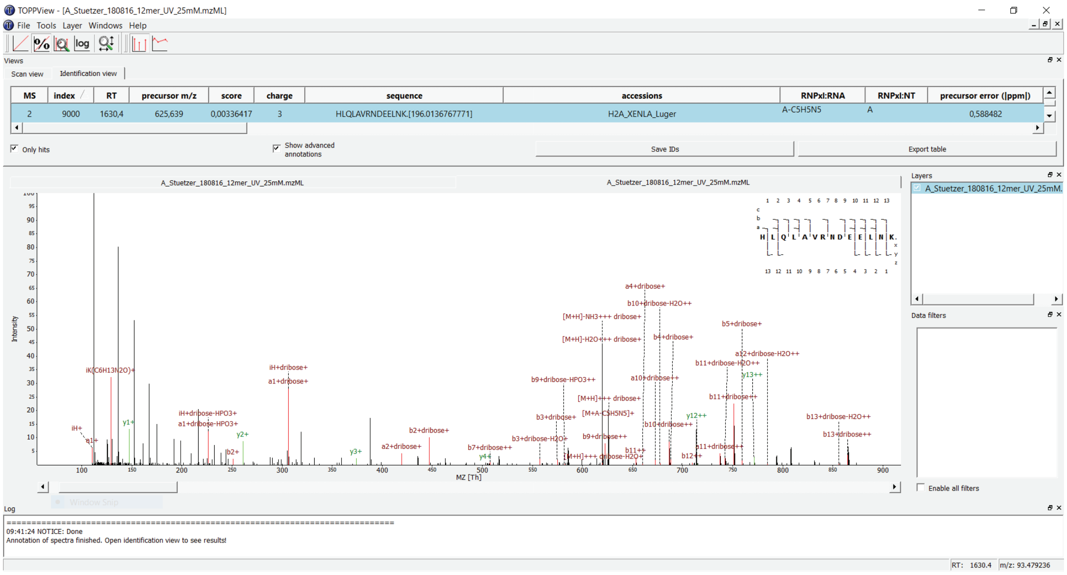
TOPPView - [A_Stuetzer_180816_12mer_UV_25mM.mzML]
File Tools Layer Windows Help
Scan view
Identification view
MS | index | RT | precursor m/z | score | charge | sequence | accessions | RNPxl:RNA | RNPxl:NT | precursor error (|ppm|)
2 | 9000 | 1630,4 | 625,639 | 0,00336417 | 3 | HLQLAVRNDEELNK.[196.0136767771] | H2A_XENLA_Luger | A-C5H5N5 | A | 0,588482
Only hits
Show advanced annotations
Save IDs
Export table
A_Stuetzer_180816_12mer_UV_25mM.mzML
Layers
Data filters
Enable all filters
Intensity
MZ [Th]
09:41:24 NOTICE: Done
Annotation of spectra finished. Open identification view to see results!
RT: 1630.4 m/z: 93.479236

16)

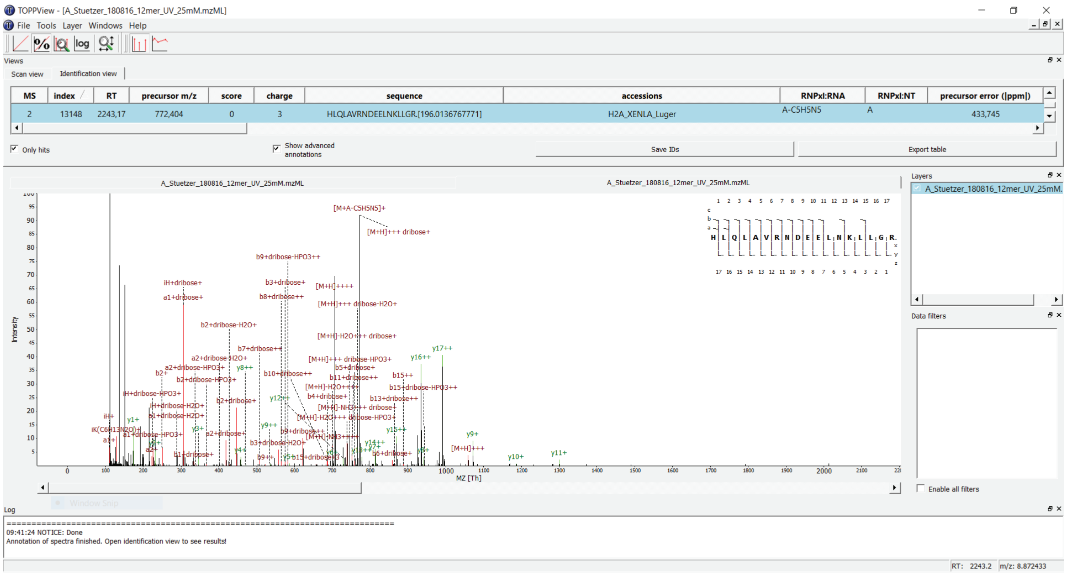
TOPPView - [A_Stuetzer_180816_12mer_UV_25mM.mzML]
File Tools Layer Windows Help
MS | index | RT | precursor m/z | score | charge | sequence | accessions | RNPxl:RNA | RNPxl:NT | precursor error (|ppm|)
2 | 13148 | 2243,17 | 772,404 | 0 | 3 | HLQLAVRNDEELNKLLGR.[196.0136767771] | H2A_XENLA_Luger | A-C5H5N5 | A | 433,745
A_Stuetzer_180816_12mer_UV_25mM.mzML
MZ [Th]
Intensity
09:41:24 NOTICE: Done
Annotation of spectra finished. Open identification view to see results!

17)

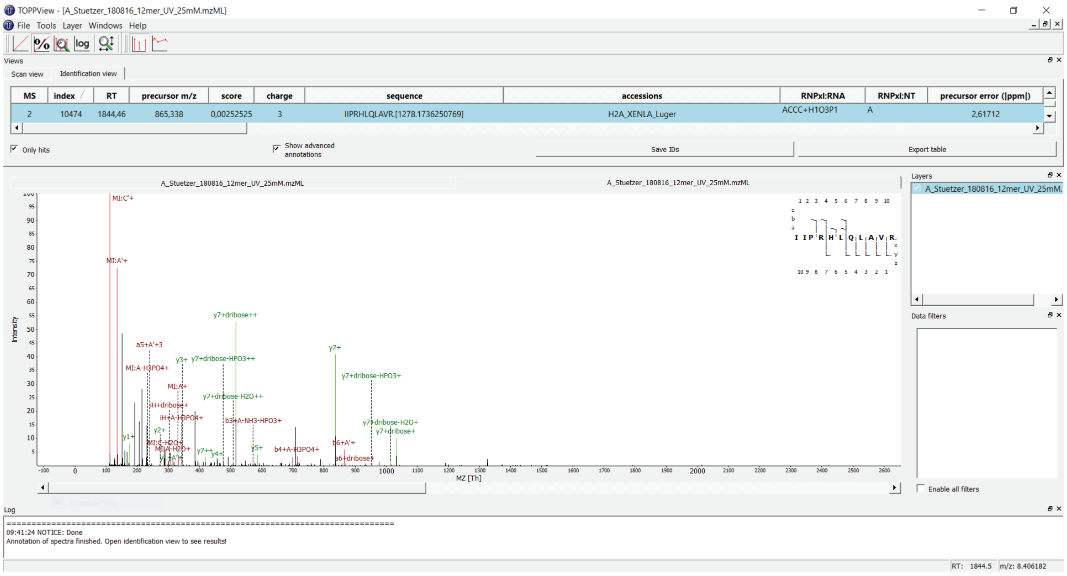
TOPPView - [A_Stuetzer_180816_12mer_UV_25mM.mzML]
File Tools Layer Windows Help
MS | index | RT | precursor m/z | score | charge | sequence | accessions | RNPxl:RNA | RNPxl:NT | precursor error (|ppm|)
2 | 10474 | 1844,46 | 865,338 | 0,00252525 | 3 | IIPRHLQLAVR.[1278.1736250769] | H2A_XENLA_Luger | ACCC+H1O3P1 | A | 2,61712
A_Stuetzer_180816_12mer_UV_25mM.mzML
MZ [Th]
Intensity
09:41:24 NOTICE: Done
Annotation of spectra finished. Open identification view to see results!

18)

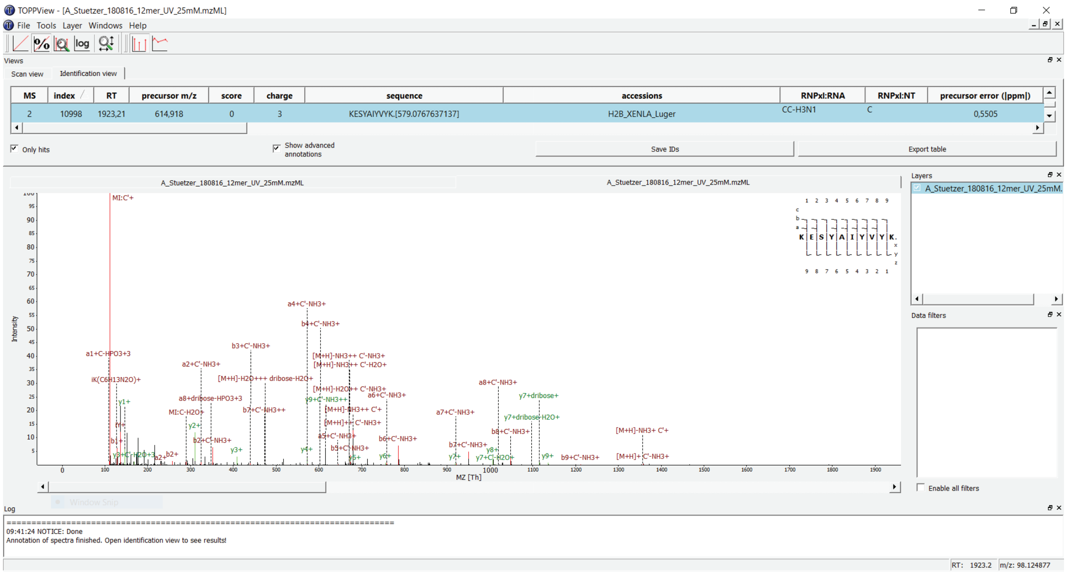
TOPPView - [A_Stuetzer_180816_12mer_UV_25mM.mzML]
File Tools Layer Windows Help
MS | index | RT | precursor m/z | score | charge | sequence | accessions | RNPxl:RNA | RNPxl:NT | precursor error (|ppm|)
2 | 10998 | 1923,21 | 614,918 | 0 | 3 | KESYAIYVYK.[579.0767637137] | H2B_XENLA_Luger | CC-H3N1 | C | 0,5505
A_Stuetzer_180816_12mer_UV_25mM.mzML
MZ [Th]
Intensity
09:41:24 NOTICE: Done
Annotation of spectra finished. Open identification view to see results!

19)

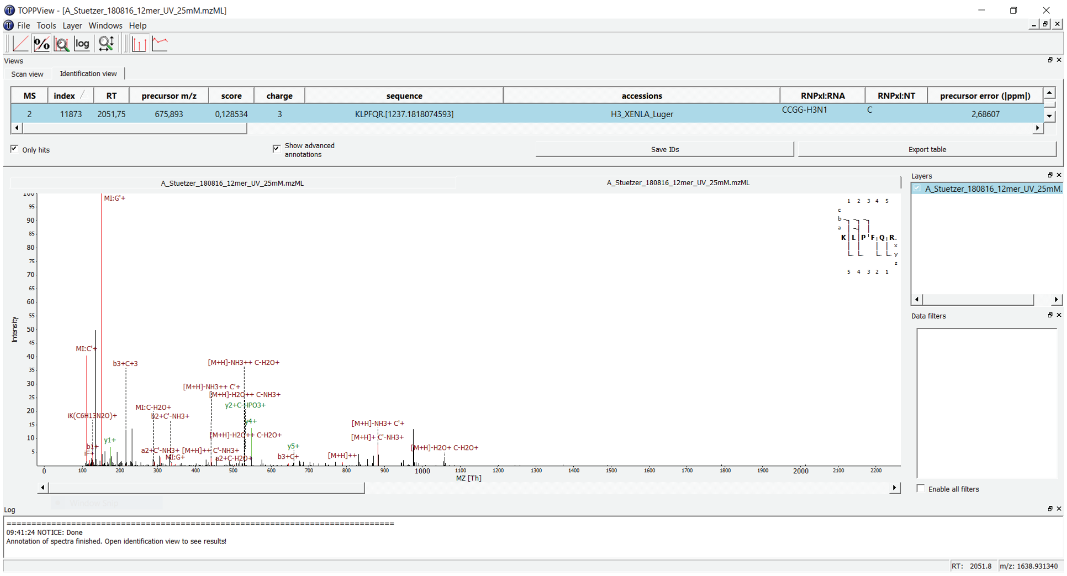
TOPPView - [A_Stuetzer_180816_12mer_UV_25mM.mzML]
MS | index | RT | precursor m/z | score | charge | sequence | accessions | RNPxl:RNA | RNPxl:NT | precursor error (|ppm|)
2 | 11873 | 2051,75 | 675,893 | 0,128534 | 3 | KLPFQR.[1237.1818074593] | H3_XENLA_Luger | CCGG-H3N1 | C | 2,68607
A_Stuetzer_180816_12mer_UV_25mM.mzML
MZ [Th]
Intensity

20)

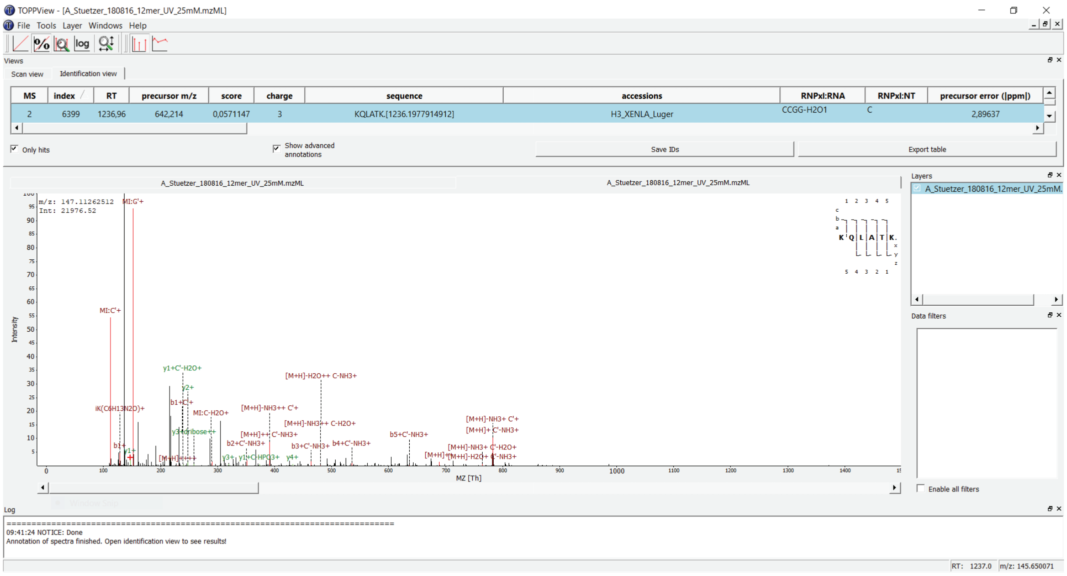
TOPPView - [A_Stuetzer_180816_12mer_UV_25mM.mzML]
MS | index | RT | precursor m/z | score | charge | sequence | accessions | RNPxl:RNA | RNPxl:NT | precursor error (|ppm|)
2 | 6399 | 1236,96 | 642,214 | 0,0571147 | 3 | KQLATK.[1236.1977914912] | H3_XENLA_Luger | CCGG-H2O1 | C | 2,89637
A_Stuetzer_180816_12mer_UV_25mM.mzML
MZ [Th]
Intensity

21)

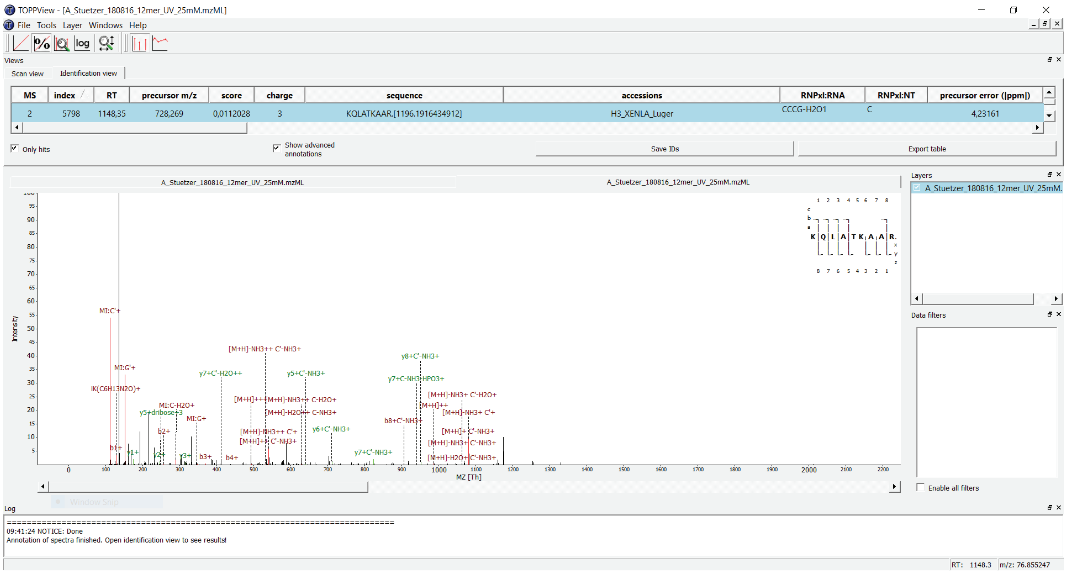
TOPPView - [A_Stuetzer_180816_12mer_UV_25mM.mzML]
MS | index | RT | precursor m/z | score | charge | sequence | accessions | RNPxl:RNA | RNPxl:NT | precursor error (|ppm|)
2 | 5798 | 1148,35 | 728,269 | 0,0112028 | 3 | KQLATKAAR.[1196.1916434912] | H3_XENLA_Luger | CCGG-H2O1 | C | 4,23161
A_Stuetzer_180816_12mer_UV_25mM.mzML
MZ [Th]
Intensity

22)

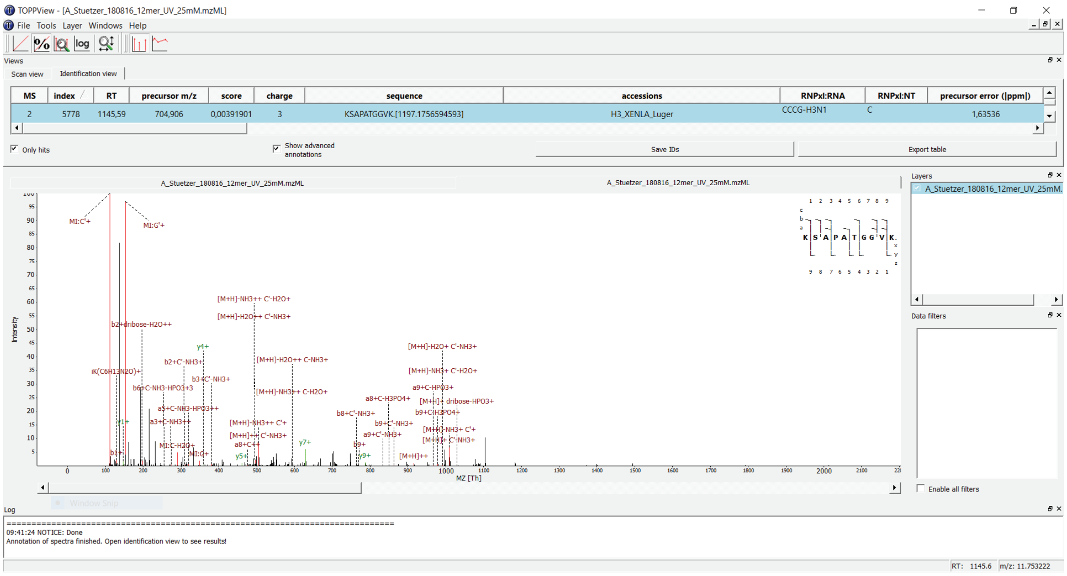
TOPPView - [A_Stuetzer_180816_12mer_UV_25mM.mzML]
File Tools Layer Windows Help
Scan view Identification view
MS | index | RT | precursor m/z | score | charge | sequence | accessions | RNPxl:RNA | RNPxl:NT | precursor error (|ppm|)
2 | 5778 | 1145,59 | 704,906 | 0,00391901 | 3 | KSAPATGGVK.[1197.1756594593] | H3_XENLA_Luger | CCCG-H3N1 | C | 1,63536
Only hits
Show advanced annotations
Save IDs
Export table
A_Stuetzer_180816_12mer_UV_25mM.mzML
Layers
Data filters
Enable all filters
MZ [Th]
Intensity
Log
09:41:24 NOTICE: Done
Annotation of spectra finished. Open identification view to see results!
RT: 1145.6 m/z: 11.753222

23)

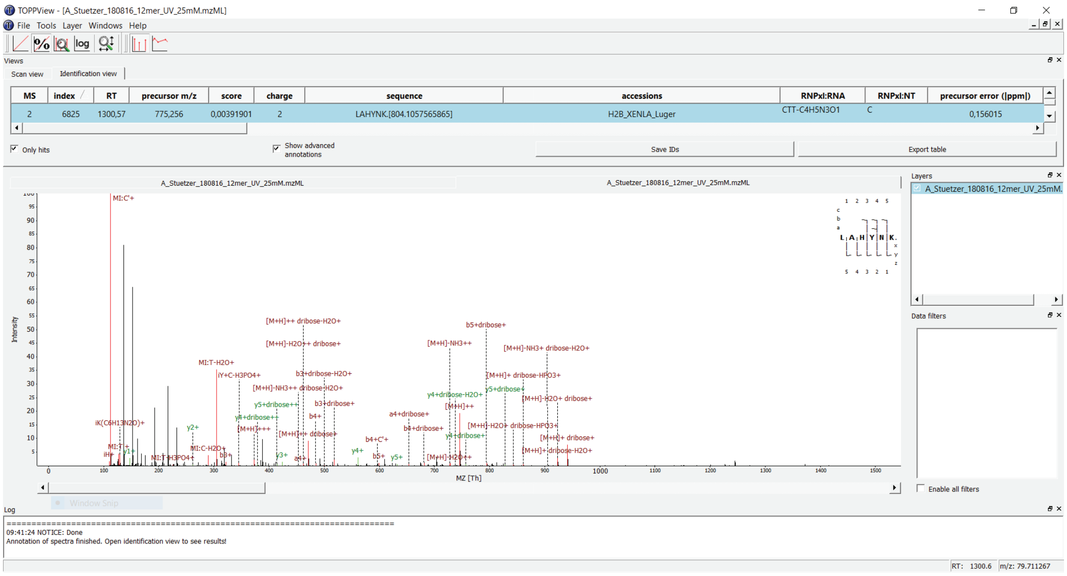
TOPPView - [A_Stuetzer_180816_12mer_UV_25mM.mzML]
File Tools Layer Windows Help
Scan view Identification view
MS | index | RT | precursor m/z | score | charge | sequence | accessions | RNPxl:RNA | RNPxl:NT | precursor error (|ppm|)
2 | 6825 | 1300,57 | 775,256 | 0,00391901 | 2 | LAHYNK.[804.1057565865] | H2B_XENLA_Luger | CTT-C4H5N3O1 | C | 0,156015
Only hits
Show advanced annotations
Save IDs
Export table
A_Stuetzer_180816_12mer_UV_25mM.mzML
Layers
Data filters
Enable all filters
MZ [Th]
Intensity
Log
09:41:24 NOTICE: Done
Annotation of spectra finished. Open identification view to see results!
RT: 1300.6 m/z: 79.711267

24)

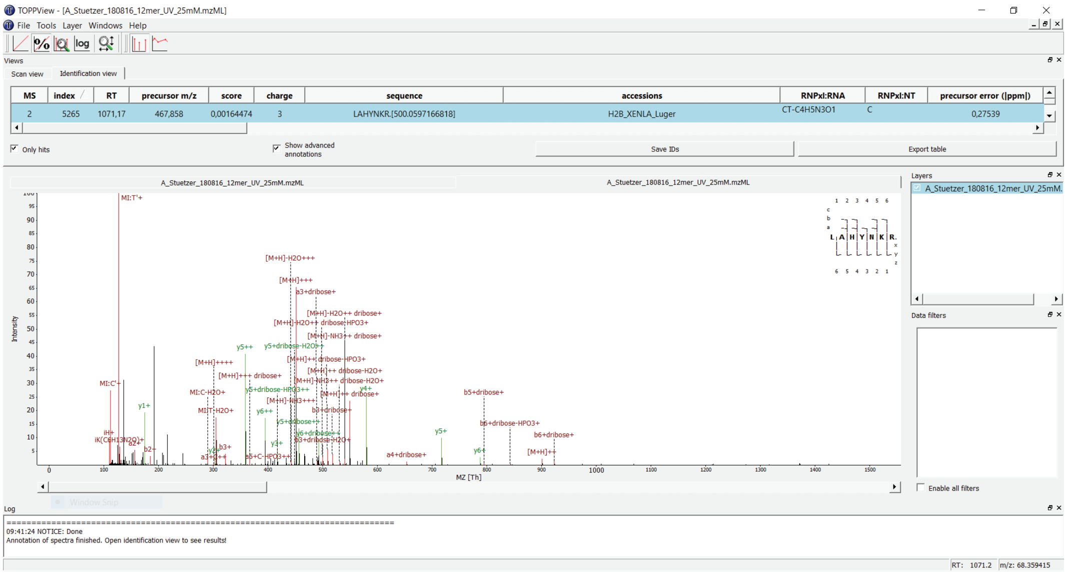
TOPPView - [A_Stuetzer_180816_12mer_UV_25mM.mzML]
File Tools Layer Windows Help
Scan view Identification view
MS | index | RT | precursor m/z | score | charge | sequence | accessions | RNPxl:RNA | RNPxl:NT | precursor error (|ppm|)
2 | 5265 | 1071,17 | 467,858 | 0,00164474 | 3 | LAHYNKR.[500.0597166818] | H2B_XENLA_Luger | CT-C4H5N3O1 | C | 0,27539
Only hits
Show advanced annotations
Save IDs
Export table
A_Stuetzer_180816_12mer_UV_25mM.mzML
Layers
Data filters
Enable all filters
MZ [Th]
Intensity
Log
09:41:24 NOTICE: Done
Annotation of spectra finished. Open identification view to see results!
RT: 1071.2 m/z: 68.359415

25)

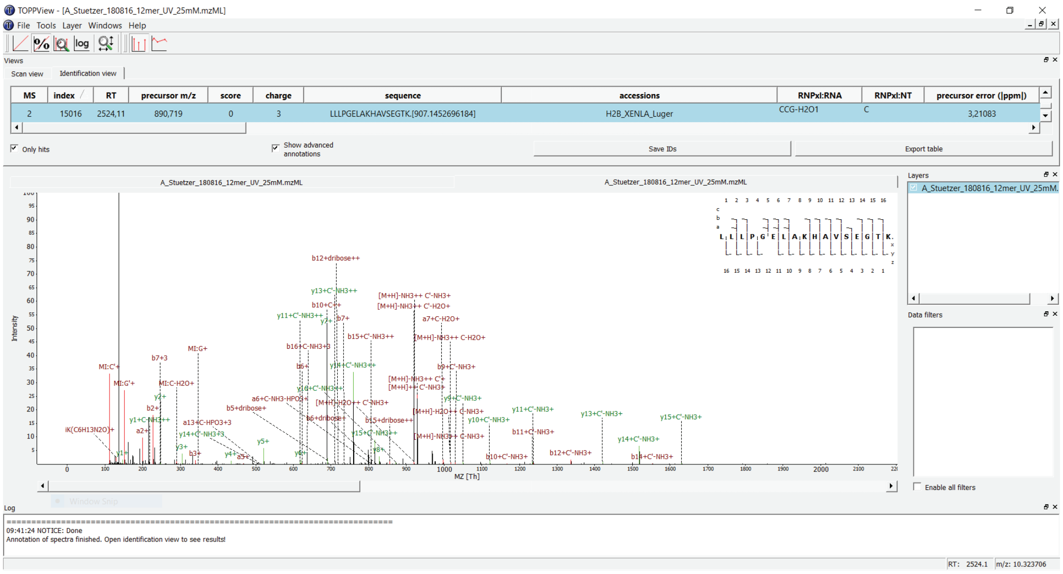
TOPPView - [A_Stuetzer_180816_12mer_UV_25mM.mzML]
MS | index | RT | precursor m/z | score | charge | sequence | accessions | RNPxl:RNA | RNPxl:NT | precursor error (|ppm|)
2 | 15016 | 2524,11 | 890,719 | 0 | 3 | LLLPGELAKHAVSEGTK.[907.1452696184] | H2B_XENLA_Luger | CCG-H2O1 | C | 3,21083
09:41:24 NOTICE: Done
Annotation of spectra finished. Open identification view to see results!

26)

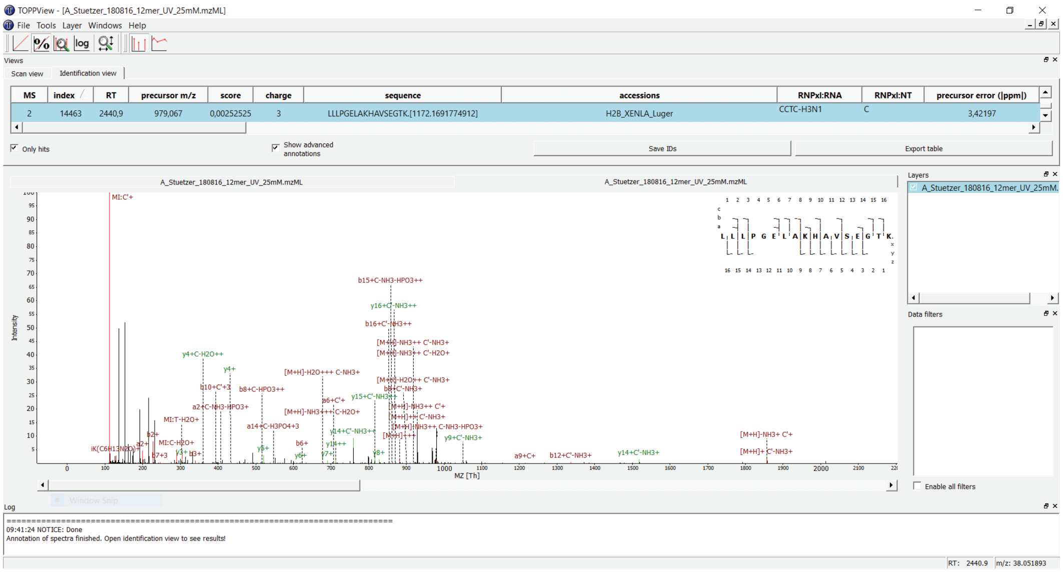
TOPPView - [A_Stuetzer_180816_12mer_UV_25mM.mzML]
MS | index | RT | precursor m/z | score | charge | sequence | accessions | RNPxl:RNA | RNPxl:NT | precursor error (|ppm|)
2 | 14463 | 2440,9 | 979,067 | 0,00252525 | 3 | LLLPGELAKHAVSEGTK.[1172.1691774912] | H2B_XENLA_Luger | CCTC-H3N1 | C | 3,42197
09:41:24 NOTICE: Done
Annotation of spectra finished. Open identification view to see results!

27)

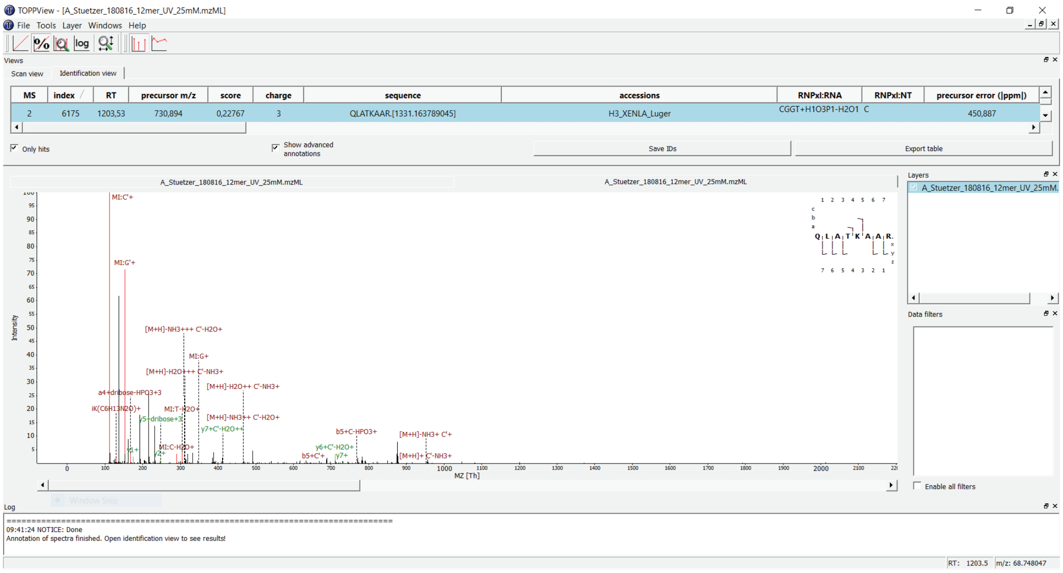
TOPPView - [A_Stuetzer_180816_12mer_UV_25mM.mzML]
MS | index | RT | precursor m/z | score | charge | sequence | accessions | RNPxl:RNA | RNPxl:NT | precursor error (|ppm|)
2 | 6175 | 1203,53 | 730,894 | 0,22767 | 3 | QLATKAAR.[1331.163789045] | H3_XENLA_Luger | CGGT+H1O3P1-H2O1 | C | 450,887
09:41:24 NOTICE: Done
Annotation of spectra finished. Open identification view to see results!

28)

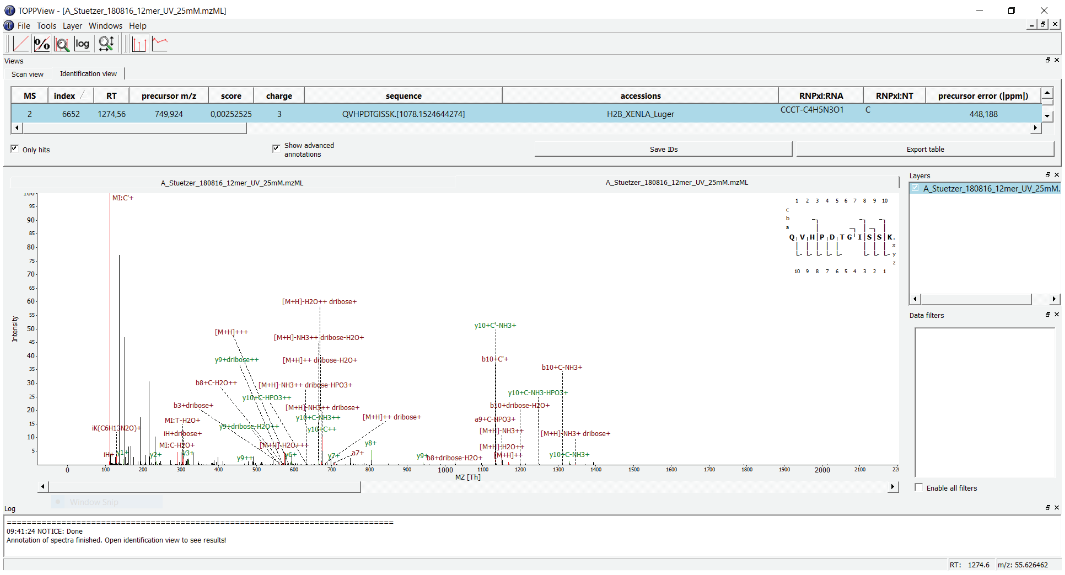
TOPPView - [A_Stuetzer_180816_12mer_UV_25mM.mzML]
File Tools Layer Windows Help
Scan view Identification view
MS index RT precursor m/z score charge sequence accessions RNPxl:RNA RNPxl:NT precursor error (|ppm|)
2 6652 1274,56 749,924 0,00252525 3 QVHPDTGISSK.[1078.1524644274] H2B_XENLA_Luger CCCT-C4H5N3O1 C 448,188
Only hits
Show advanced annotations
Save IDs
Export table
A_Stuetzer_180816_12mer_UV_25mM.mzML
Layers
Data filters
Enable all filters
MZ [Th]
Intensity
09:41:24 NOTICE: Done
Annotation of spectra finished. Open identification view to see results!
RT: 1274.6 m/z: 55.626462

29)

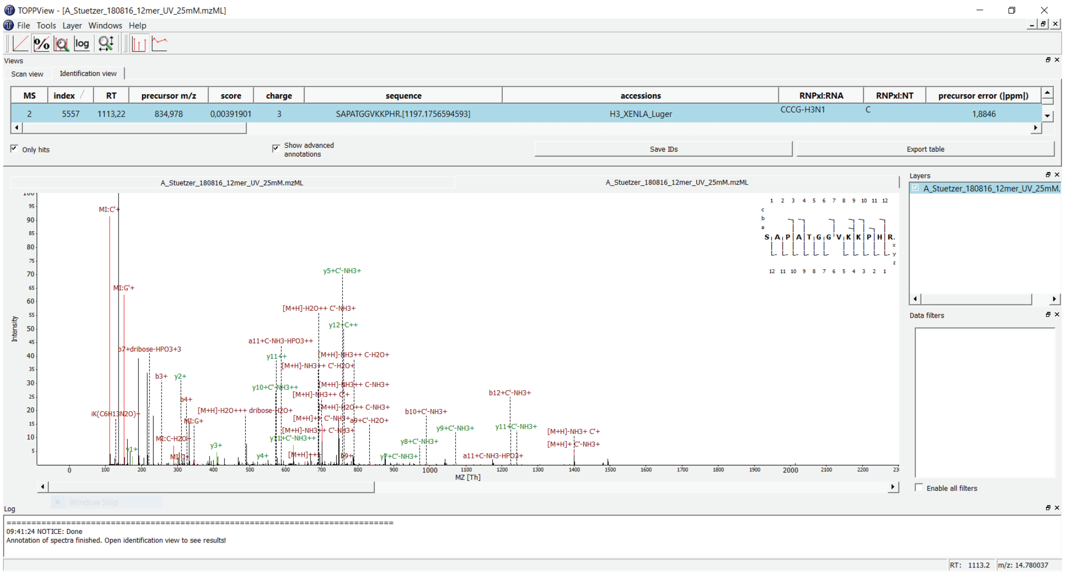
TOPPView - [A_Stuetzer_180816_12mer_UV_25mM.mzML]
File Tools Layer Windows Help
Scan view Identification view
MS index RT precursor m/z score charge sequence accessions RNPxl:RNA RNPxl:NT precursor error (|ppm|)
2 5557 1113,22 834,978 0,00391901 3 SAPATGGVKKPHR.[1197.1756594593] H3_XENLA_Luger CCCG-H3N1 C 1,8846
Only hits
Show advanced annotations
Save IDs
Export table
A_Stuetzer_180816_12mer_UV_25mM.mzML
Layers
Data filters
Enable all filters
MZ [Th]
Intensity
09:41:24 NOTICE: Done
Annotation of spectra finished. Open identification view to see results!
RT: 1113.2 m/z: 14.780037

30)

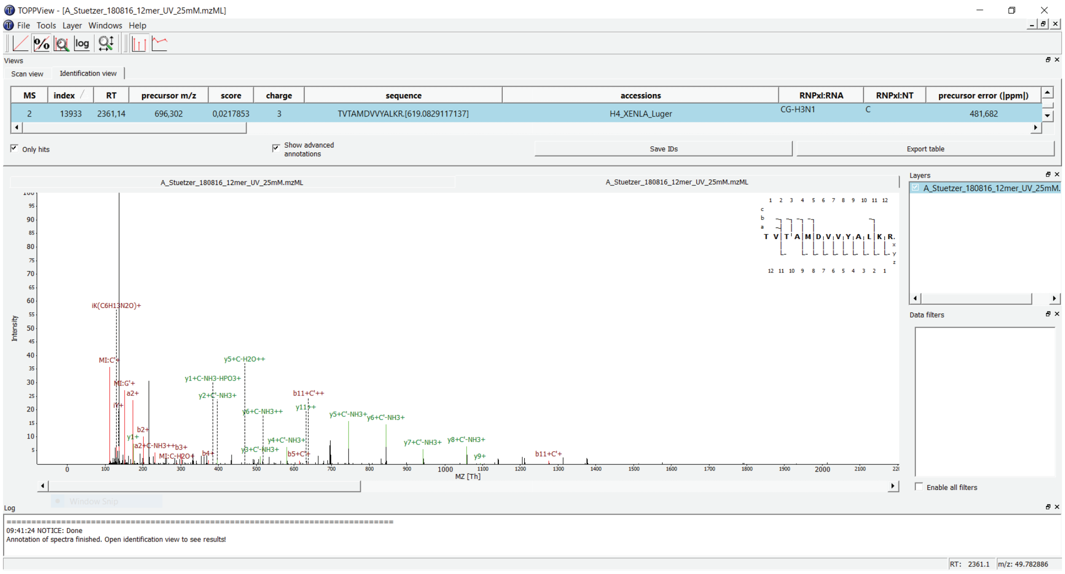
TOPPView - [A_Stuetzer_180816_12mer_UV_25mM.mzML]
File Tools Layer Windows Help
Scan view Identification view
MS index RT precursor m/z score charge sequence accessions RNPxl:RNA RNPxl:NT precursor error (|ppm|)
2 13933 2361,14 696,302 0,0217853 3 TVTAMDVVYALKR.[619.0829117137] H4_XENLA_Luger CG-H3N1 C 481,682
Only hits
Show advanced annotations
Save IDs
Export table
A_Stuetzer_180816_12mer_UV_25mM.mzML
Layers
Data filters
Enable all filters
MZ [Th]
Intensity
09:41:24 NOTICE: Done
Annotation of spectra finished. Open identification view to see results!
RT: 2361.1 m/z: 49.782886

31)

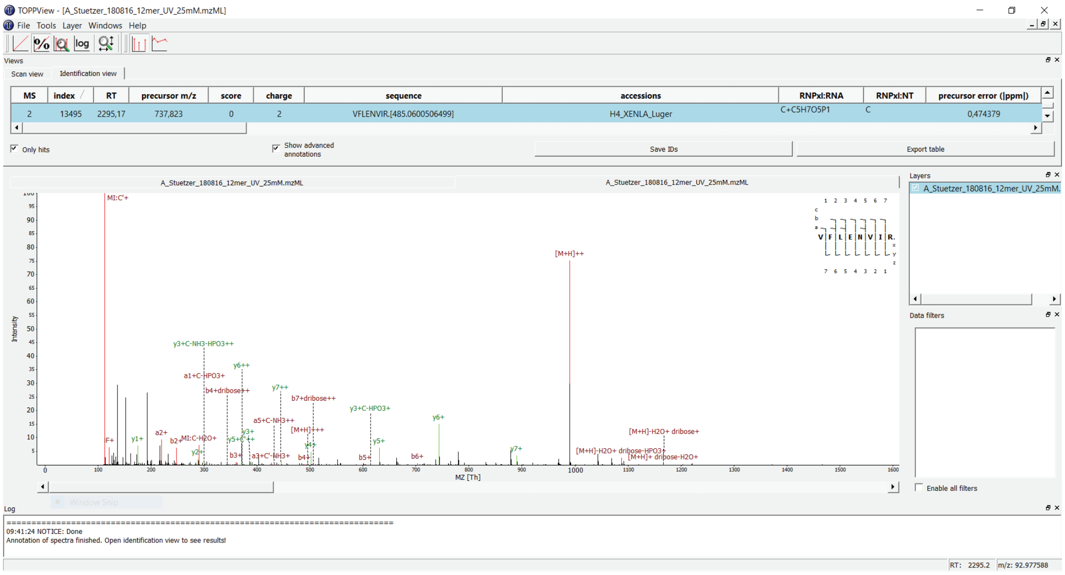
TOPPView - [A_Stuetzer_180816_12mer_UV_25mM.mzML]
File Tools Layer Windows Help
Scan view
Identification view
MS index RT precursor m/z score charge sequence accessions RNPxl:RNA RNPxl:NT precursor error (|ppm|)
2 13495 2295,17 737,823 0 2 VFLENVIR.[485.0600506499] H4_XENLA_Luger C+C5H7O5P1 C 0,474379
Only hits
Show advanced annotations
Save IDs
Export table
A_Stuetzer_180816_12mer_UV_25mM.mzML
MZ [Th]
Intensity
Enable all filters
Data filters
Layers
09:41:24 NOTICE: Done
Annotation of spectra finished. Open identification view to see results!
RT: 2295.2 m/z: 92.977588

32)

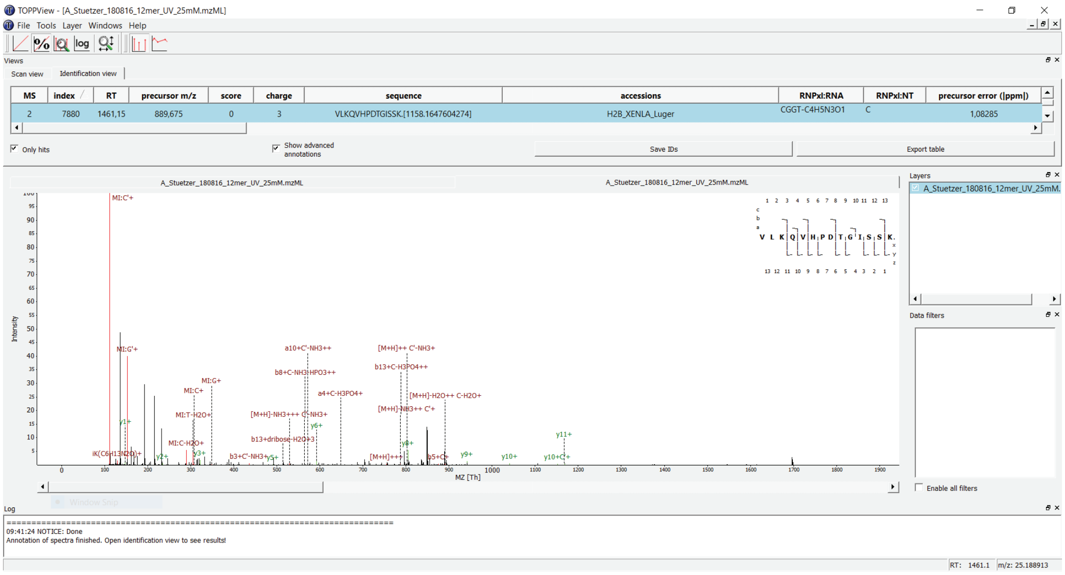
TOPPView - [A_Stuetzer_180816_12mer_UV_25mM.mzML]
File Tools Layer Windows Help
Scan view
Identification view
MS index RT precursor m/z score charge sequence accessions RNPxl:RNA RNPxl:NT precursor error (|ppm|)
2 7880 1461,15 889,675 0 3 VLKQVHPDTGISSK.[1158.1647604274] H2B_XENLA_Luger CGGT-C4H5N3O1 C 1,08285
Only hits
Show advanced annotations
Save IDs
Export table
A_Stuetzer_180816_12mer_UV_25mM.mzML
MZ [Th]
Intensity
Enable all filters
Data filters
Layers
09:41:24 NOTICE: Done
Annotation of spectra finished. Open identification view to see results!
RT: 1461.1 m/z: 25.188913

33)

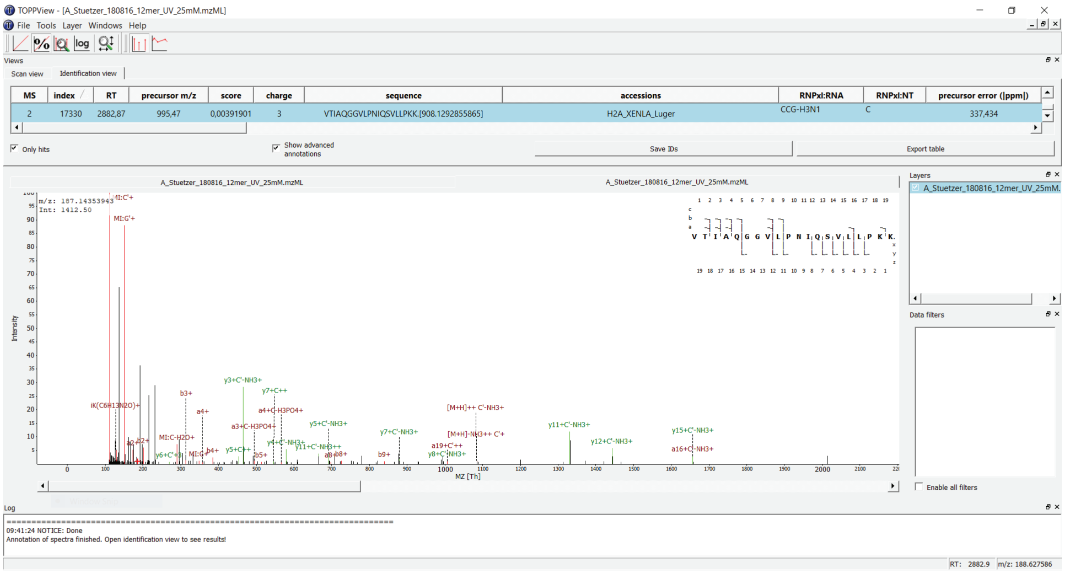
TOPPView - [A_Stuetzer_180816_12mer_UV_25mM.mzML]
File Tools Layer Windows Help
Scan view
Identification view
MS index RT precursor m/z score charge sequence accessions RNPxl:RNA RNPxl:NT precursor error (|ppm|)
2 17330 2882,87 995,47 0,00391901 3 VTIAQGGVLPNIQSVLLPKK.[908.1292855865] H2A_XENLA_Luger CCG-H3N1 C 337,434
Only hits
Show advanced annotations
Save IDs
Export table
A_Stuetzer_180816_12mer_UV_25mM.mzML
MZ [Th]
Intensity
Enable all filters
Data filters
Layers
09:41:24 NOTICE: Done
Annotation of spectra finished. Open identification view to see results!
RT: 2882.9 m/z: 188.627586

34)

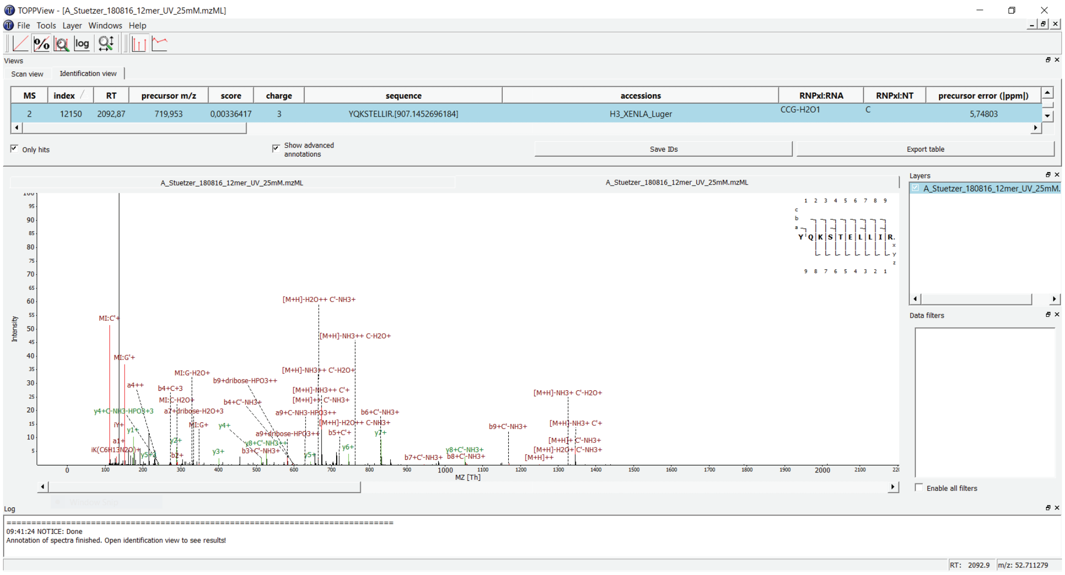
TOPPView - [A_Stuetzer_180816_12mer_UV_25mM.mzML]
File Tools Layer Windows Help
Scan view Identification view
MS index RT precursor m/z score charge sequence accessions RNPxl:RNA RNPxl:NT precursor error (|ppm|)
2 12150 2092,87 719,953 0,00336417 3 YQKSTELLIR.[907.1452696184] H3_XENLA_Luger CCG-H2O1 C 5,74803
Only hits
Show advanced annotations
Save IDs
Export table
A_Stuetzer_180816_12mer_UV_25mM.mzML
Layers
Data filters
Enable all filters
MZ [Th]
Intensity
Log
09:41:24 NOTICE: Done
Annotation of spectra finished. Open identification view to see results!
RT: 2092.9 m/z: 52.711279

35)

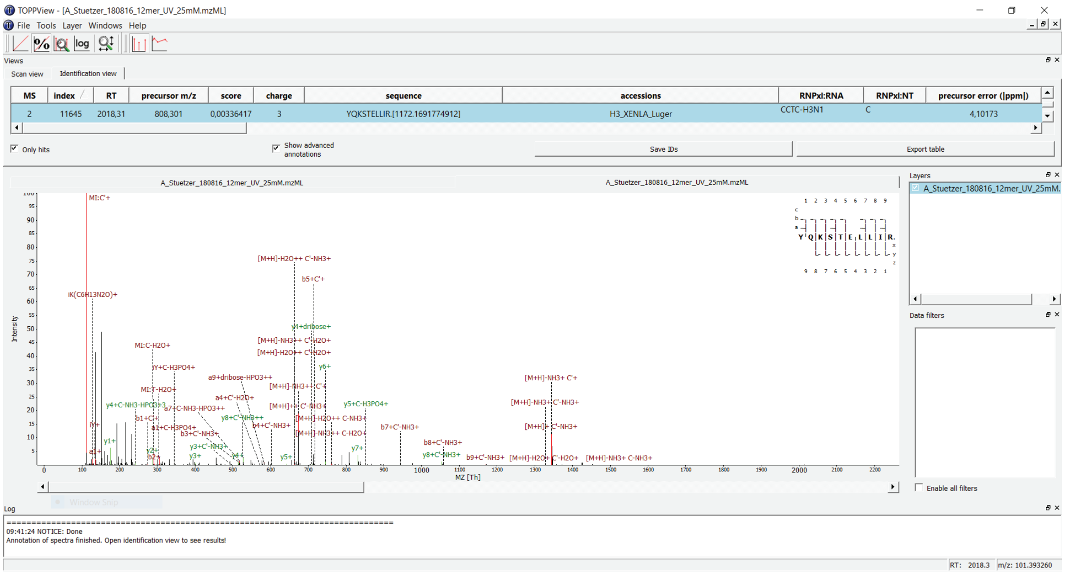
TOPPView - [A_Stuetzer_180816_12mer_UV_25mM.mzML]
File Tools Layer Windows Help
Scan view Identification view
MS index RT precursor m/z score charge sequence accessions RNPxl:RNA RNPxl:NT precursor error (|ppm|)
2 11645 2018,31 808,301 0,00336417 3 YQKSTELLIR.[1172.1691774912] H3_XENLA_Luger CCTC-H3N1 C 4,10173
Only hits
Show advanced annotations
Save IDs
Export table
A_Stuetzer_180816_12mer_UV_25mM.mzML
Layers
Data filters
Enable all filters
MZ [Th]
Intensity
Log
09:41:24 NOTICE: Done
Annotation of spectra finished. Open identification view to see results!
RT: 2018.3 m/z: 101.393260

36)

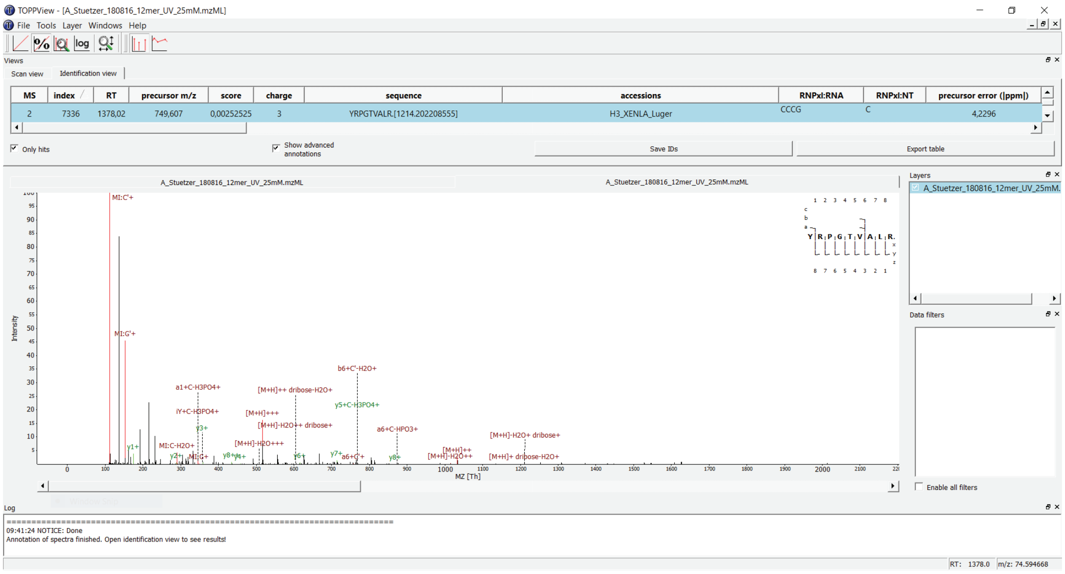
TOPPView - [A_Stuetzer_180816_12mer_UV_25mM.mzML]
File Tools Layer Windows Help
Scan view Identification view
MS index RT precursor m/z score charge sequence accessions RNPxl:RNA RNPxl:NT precursor error (|ppm|)
2 7336 1378,02 749,607 0,00252525 3 YRPGTVALR.[1214.202208555] H3_XENLA_Luger CCCG C 4,2296
Only hits
Show advanced annotations
Save IDs
Export table
A_Stuetzer_180816_12mer_UV_25mM.mzML
Layers
Data filters
Enable all filters
MZ [Th]
Intensity
Log
09:41:24 NOTICE: Done
Annotation of spectra finished. Open identification view to see results!
RT: 1378.0 m/z: 74.594668

37)

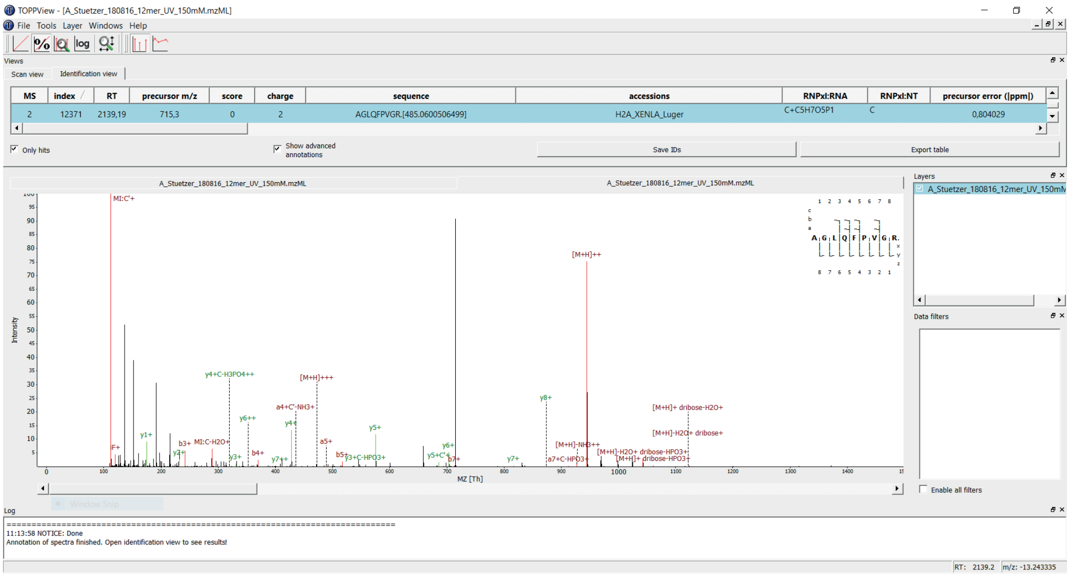
TOPPView - [A_Stuetzer_180816_12mer_UV_150mM.mzML]
File Tools Layer Windows Help
Scan view
Identification view
MS | index | RT | precursor m/z | score | charge | sequence | accessions | RNPxl:RNA | RNPxl:NT | precursor error (|ppm|)
2 | 12371 | 2139,19 | 715,3 | 0 | 2 | AGLQFPVGR.[485.0600506499] | H2A_XENLA_Luger | C+C5H7O5P1 | C | 0,804029
Only hits
Show advanced annotations
Save IDs
Export table
A_Stuetzer_180816_12mer_UV_150mM.mzML
Layers
Data filters
Enable all filters
MZ [Th]
Intensity
Log
11:13:58 NOTICE: Done
Annotation of spectra finished. Open identification view to see results!
RT: 2139.2 m/z: -13.243335

38)

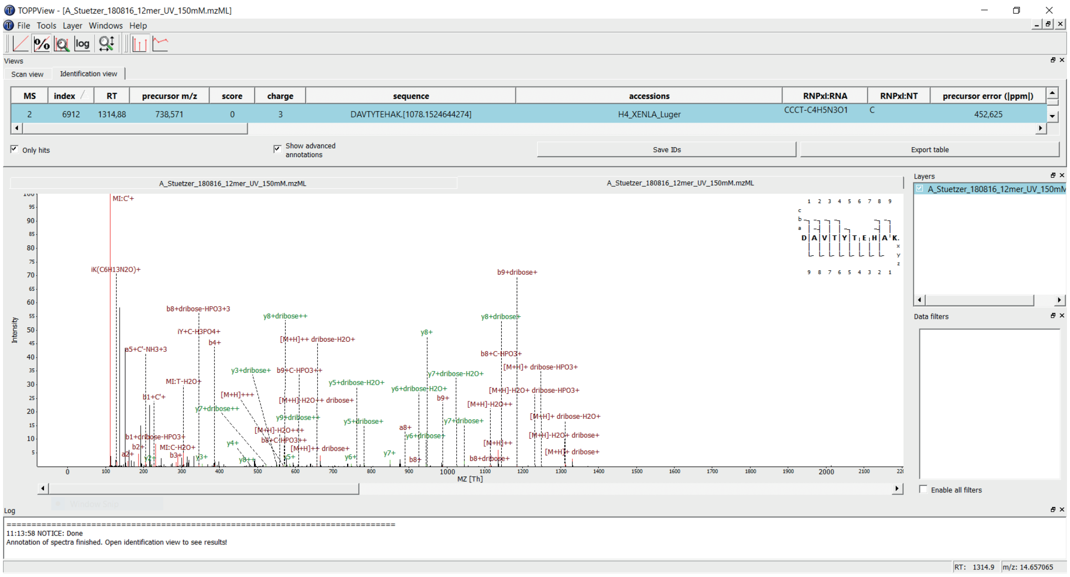
TOPPView - [A_Stuetzer_180816_12mer_UV_150mM.mzML]
File Tools Layer Windows Help
Scan view
Identification view
MS | index | RT | precursor m/z | score | charge | sequence | accessions | RNPxl:RNA | RNPxl:NT | precursor error (|ppm|)
2 | 6912 | 1314,88 | 738,571 | 0 | 3 | DAVTYTEHAK.[1078.1524644274] | H4_XENLA_Luger | CCCT-C4H5N3O1 | C | 452,625
Only hits
Show advanced annotations
Save IDs
Export table
A_Stuetzer_180816_12mer_UV_150mM.mzML
Layers
Data filters
Enable all filters
MZ [Th]
Intensity
Log
11:13:58 NOTICE: Done
Annotation of spectra finished. Open identification view to see results!
RT: 1314.9 m/z: 14.657065

39)

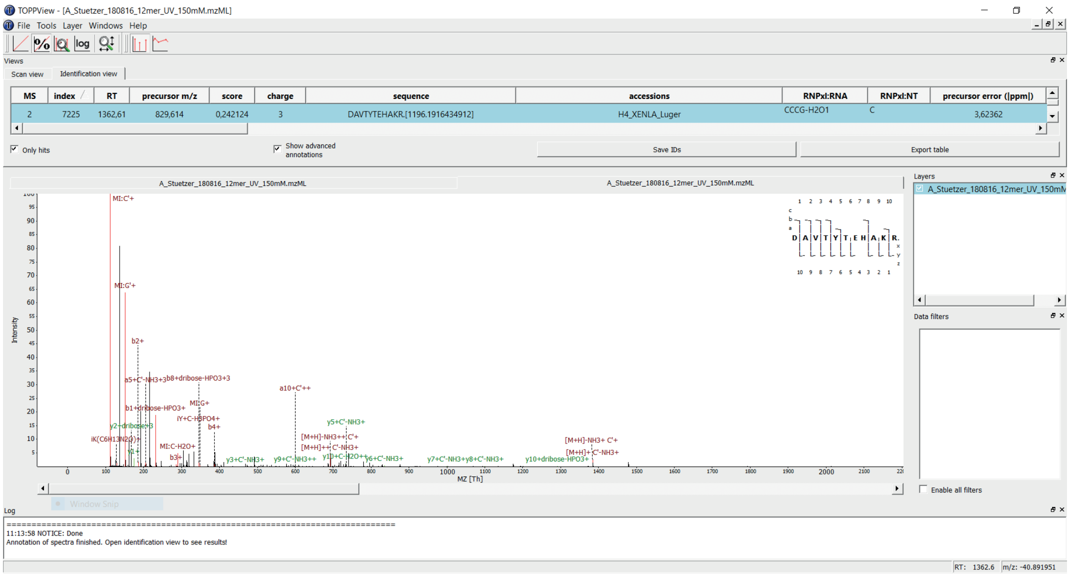
TOPPView - [A_Stuetzer_180816_12mer_UV_150mM.mzML]
File Tools Layer Windows Help
Scan view
Identification view
MS | index | RT | precursor m/z | score | charge | sequence | accessions | RNPxl:RNA | RNPxl:NT | precursor error (|ppm|)
2 | 7225 | 1362,61 | 829,614 | 0,242124 | 3 | DAVTYTEHAKR.[1196.1916434912] | H4_XENLA_Luger | CCCG-H2O1 | C | 3,62362
Only hits
Show advanced annotations
Save IDs
Export table
A_Stuetzer_180816_12mer_UV_150mM.mzML
Layers
Data filters
Enable all filters
MZ [Th]
Intensity
Log
11:13:58 NOTICE: Done
Annotation of spectra finished. Open identification view to see results!
RT: 1362.6 m/z: -40.891951

40)

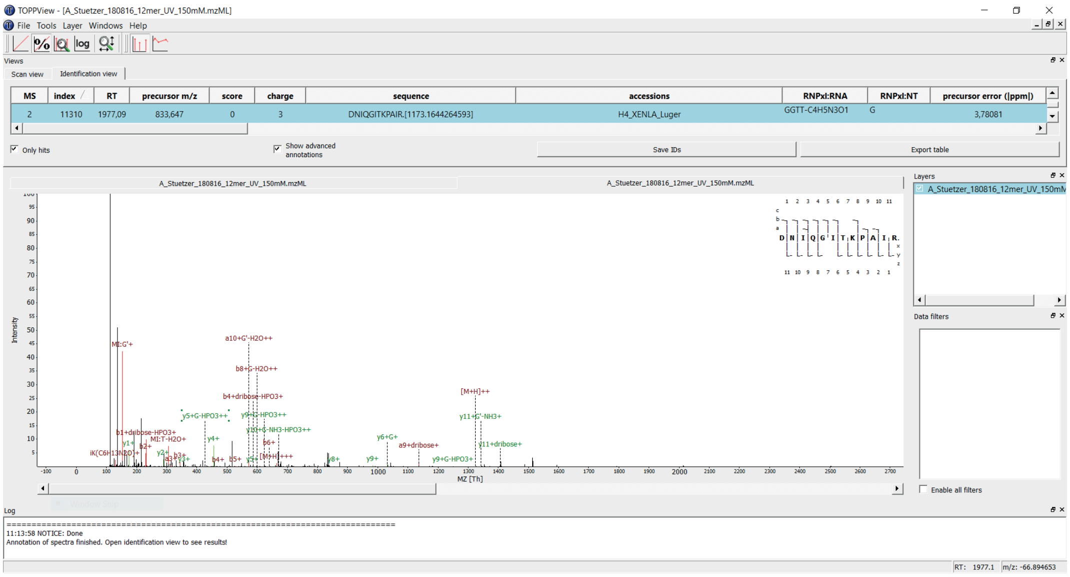
TOPPView - [A_Stuetzer_180816_12mer_UV_150mM.mzML]
File Tools Layer Windows Help
Scan view Identification view
MS | index | RT | precursor m/z | score | charge | sequence | accessions | RNPxl:RNA | RNPxl:NT | precursor error (|ppm|)
2 | 11310 | 1977,09 | 833,647 | 0 | 3 | DNIQGITKPAIR.[1173.1644264593] | H4_XENLA_Luger | GGTT-C4H5N3O1 | G | 3,78081
Only hits
Show advanced annotations
Save IDs
Export table
A_Stuetzer_180816_12mer_UV_150mM.mzML
Layers
Data filters
Enable all filters
Intensity
MZ [Th]
Log
11:13:58 NOTICE: Done
Annotation of spectra finished. Open identification view to see results!
RT: 1977.1 m/z: -66.894653

41)

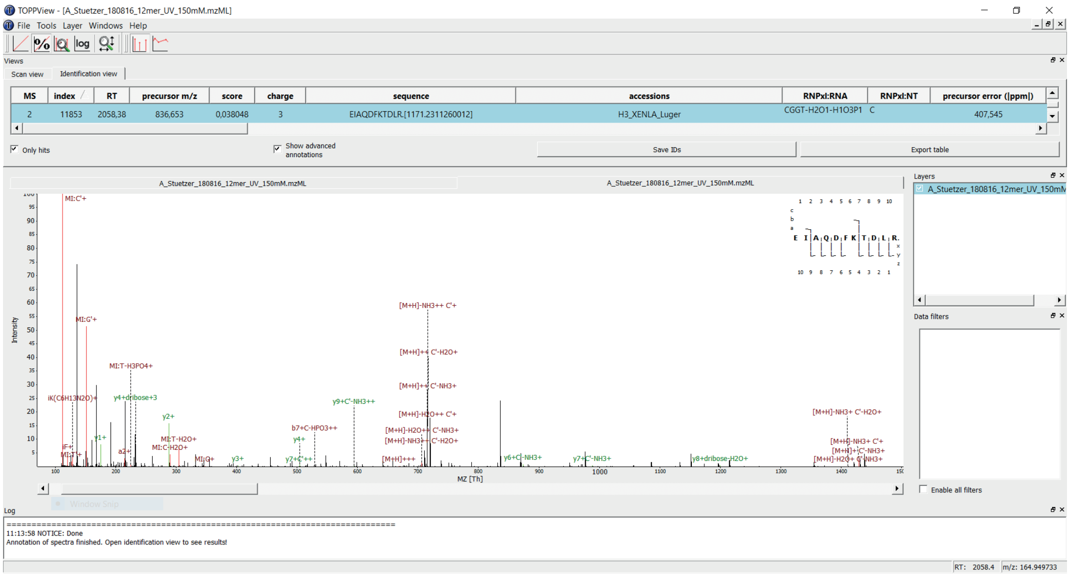
TOPPView - [A_Stuetzer_180816_12mer_UV_150mM.mzML]
File Tools Layer Windows Help
Scan view Identification view
MS | index | RT | precursor m/z | score | charge | sequence | accessions | RNPxl:RNA | RNPxl:NT | precursor error (|ppm|)
2 | 11853 | 2058,38 | 836,653 | 0,038048 | 3 | EIAQDFKTDLR.[1171.2311260012] | H3_XENLA_Luger | CGGT-H2O1-H1O3P1 | C | 407,545
Only hits
Show advanced annotations
Save IDs
Export table
A_Stuetzer_180816_12mer_UV_150mM.mzML
Layers
Data filters
Enable all filters
Intensity
MZ [Th]
Log
11:13:58 NOTICE: Done
Annotation of spectra finished. Open identification view to see results!
RT: 2058.4 m/z: 164.949733

42)

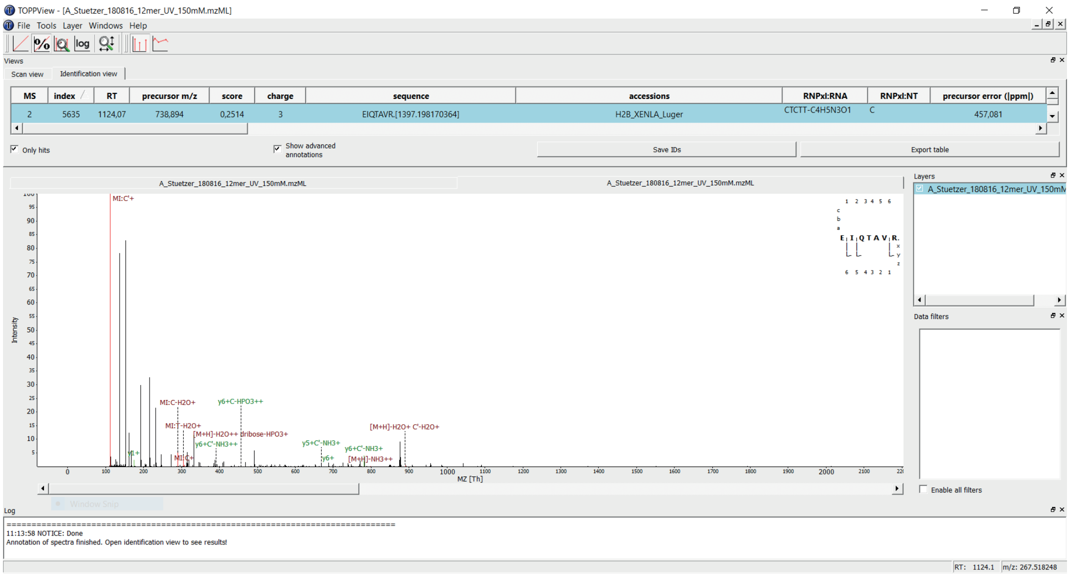
TOPPView - [A_Stuetzer_180816_12mer_UV_150mM.mzML]
File Tools Layer Windows Help
Scan view Identification view
MS | index | RT | precursor m/z | score | charge | sequence | accessions | RNPxl:RNA | RNPxl:NT | precursor error (|ppm|)
2 | 5635 | 1124,07 | 738,894 | 0,2514 | 3 | EIQTAVR.[1397.198170364] | H2B_XENLA_Luger | CTCTT-C4H5N3O1 | C | 457,081
Only hits
Show advanced annotations
Save IDs
Export table
A_Stuetzer_180816_12mer_UV_150mM.mzML
Layers
Data filters
Enable all filters
Intensity
MZ [Th]
Log
11:13:58 NOTICE: Done
Annotation of spectra finished. Open identification view to see results!
RT: 1124.1 m/z: 267.518248

43)

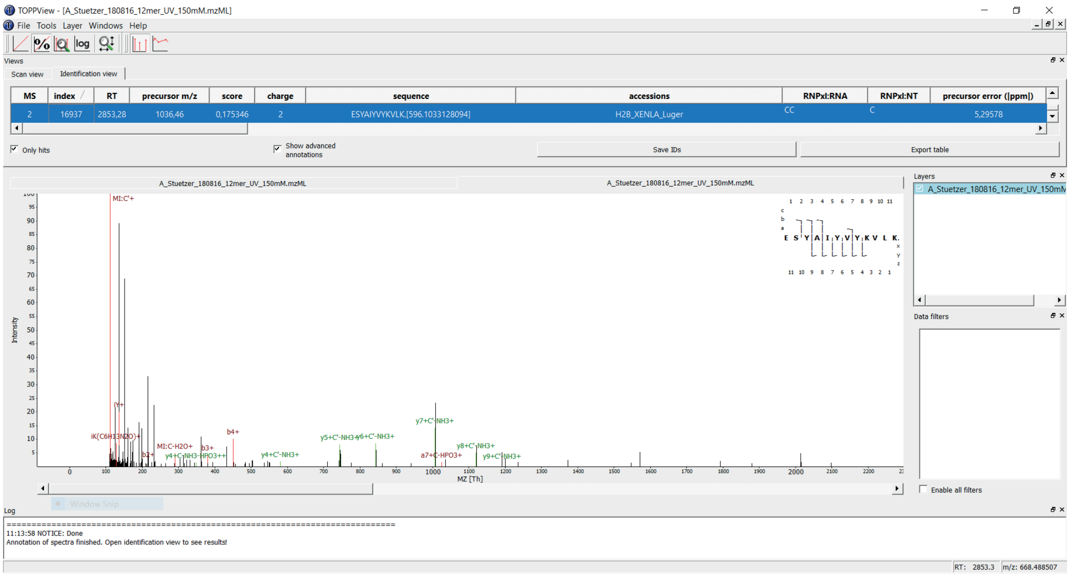
TOPPView - [A_Stuetzer_180816_12mer_UV_150mM.mzML]
MS | index | RT | precursor m/z | score | charge | sequence | accessions | RNPxl:RNA | RNPxl:NT | precursor error (|ppm|)
2 | 16937 | 2853,28 | 1036,46 | 0,175346 | 2 | ESYAIYVYKVLK.[596.1033128094] | H2B_XENLA_Luger | CC | C | 5,29578
11:13:58 NOTICE: Done
Annotation of spectra finished. Open identification view to see results!

44)

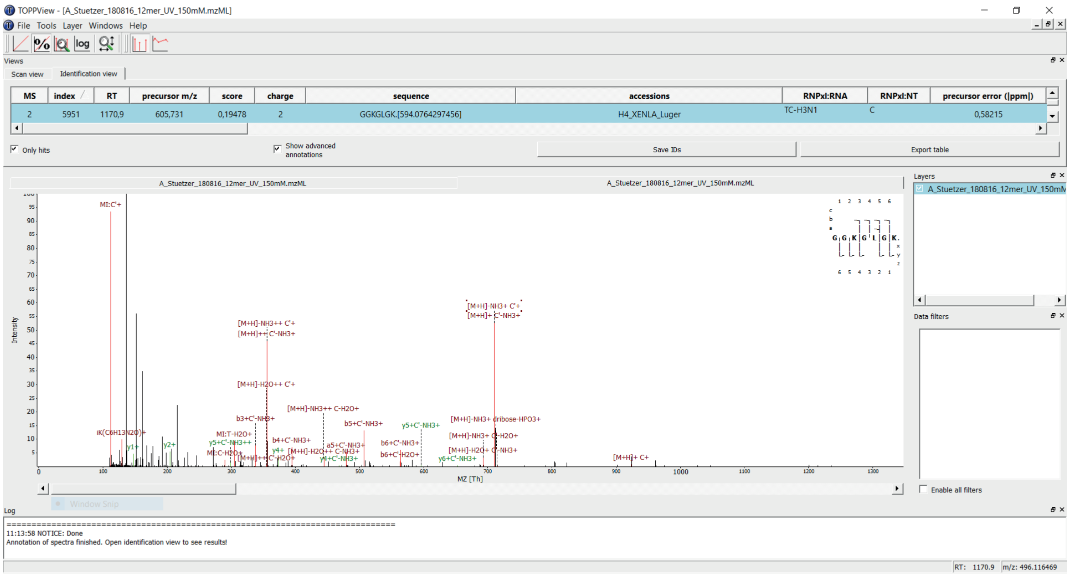
TOPPView - [A_Stuetzer_180816_12mer_UV_150mM.mzML]
MS | index | RT | precursor m/z | score | charge | sequence | accessions | RNPxl:RNA | RNPxl:NT | precursor error (|ppm|)
2 | 5951 | 1170,9 | 605,731 | 0,19478 | 2 | GGKGLGK.[594.0764297456] | H4_XENLA_Luger | TC-H3N1 | C | 0,58215
11:13:58 NOTICE: Done
Annotation of spectra finished. Open identification view to see results!

45)

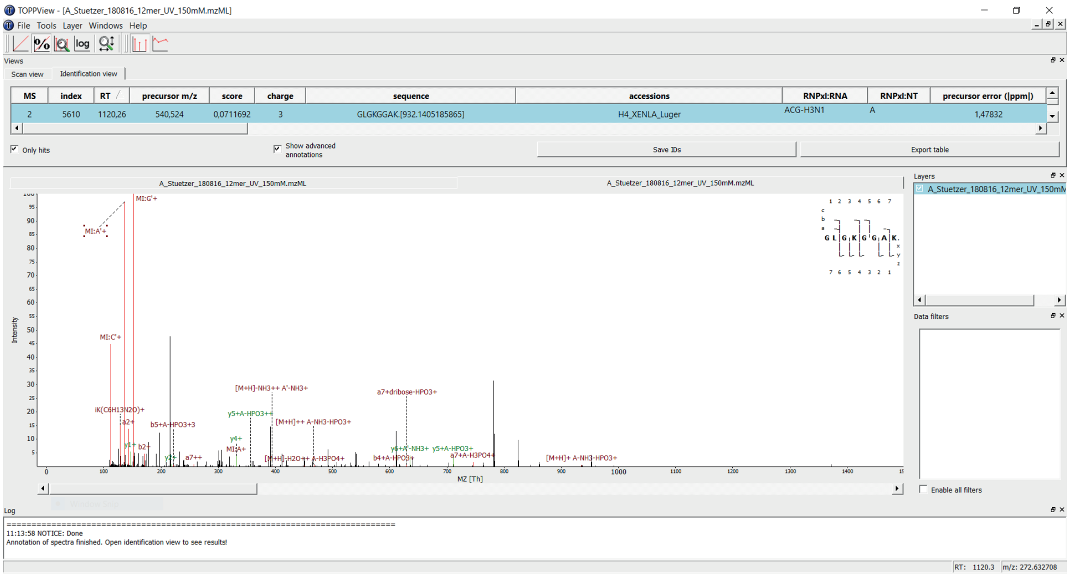
TOPPView - [A_Stuetzer_180816_12mer_UV_150mM.mzML]
MS | index | RT | precursor m/z | score | charge | sequence | accessions | RNPxl:RNA | RNPxl:NT | precursor error (|ppm|)
2 | 5610 | 1120,26 | 540,524 | 0,0711692 | 3 | GLGKGGAK.[932.1405185865] | H4_XENLA_Luger | ACG-H3N1 | A | 1,47832
11:13:58 NOTICE: Done
Annotation of spectra finished. Open identification view to see results!

46)

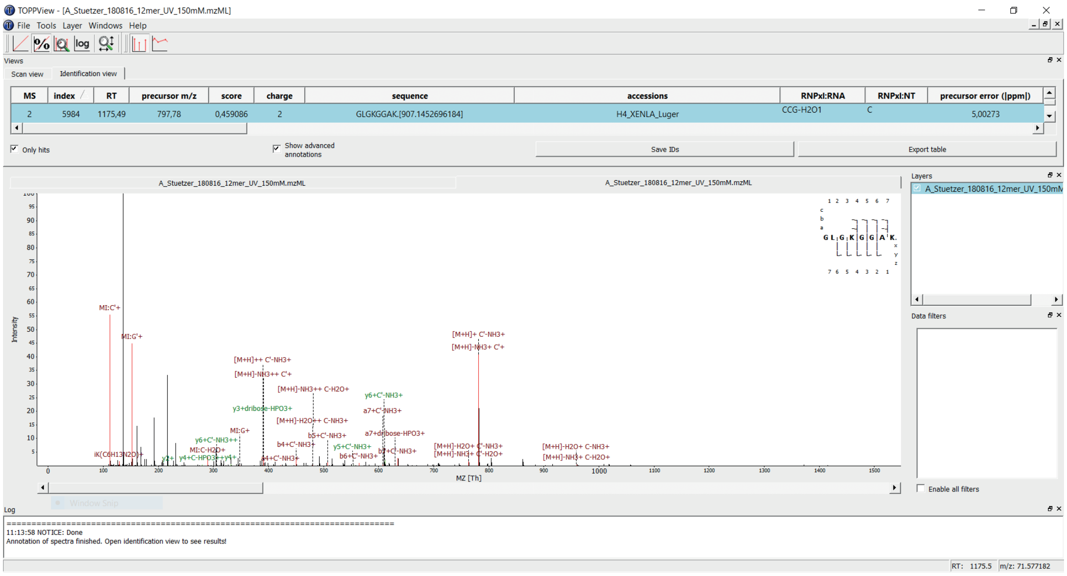
TOPPView - [A_Stuetzer_180816_12mer_UV_150mM.mzML]
MS | index | RT | precursor m/z | score | charge | sequence | accessions | RNPxl:RNA | RNPxl:NT | precursor error (|ppm|)
2 | 5984 | 1175,49 | 797,78 | 0,459086 | 2 | GLGKGGAK.[907.1452696184] | H4_XENLA_Luger | CCG-H2O1 | C | 5,00273
11:13:58 NOTICE: Done
Annotation of spectra finished. Open identification view to see results!

47)

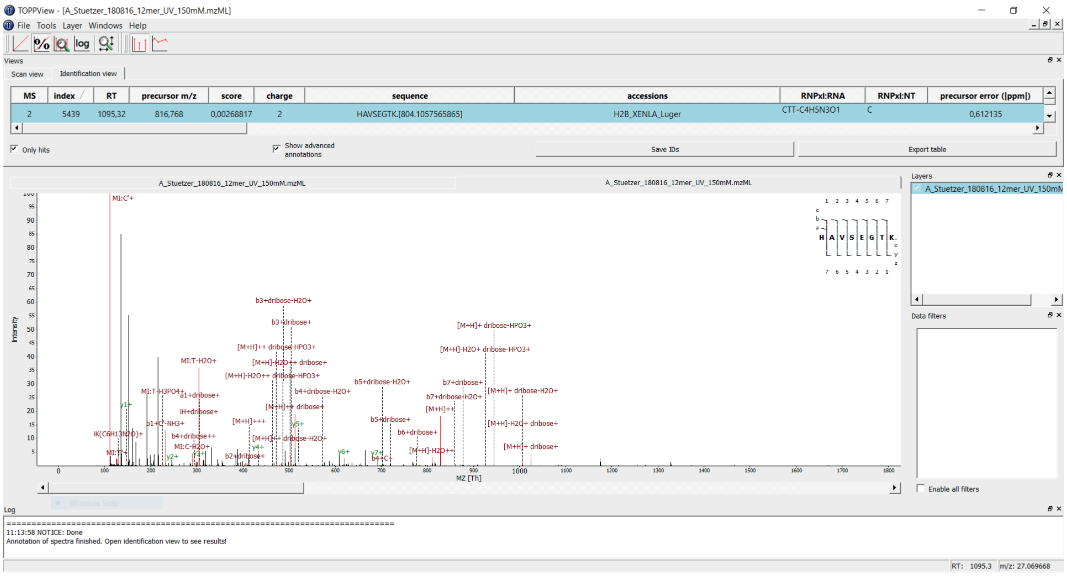
TOPPView - [A_Stuetzer_180816_12mer_UV_150mM.mzML]
MS | index | RT | precursor m/z | score | charge | sequence | accessions | RNPxl:RNA | RNPxl:NT | precursor error (|ppm|)
2 | 5439 | 1095,32 | 816,768 | 0,00268817 | 2 | HAVSEGTK.[804.1057565865] | H2B_XENLA_Luger | CTT-C4H5N3O1 | C | 0,612135
11:13:58 NOTICE: Done
Annotation of spectra finished. Open identification view to see results!

48)

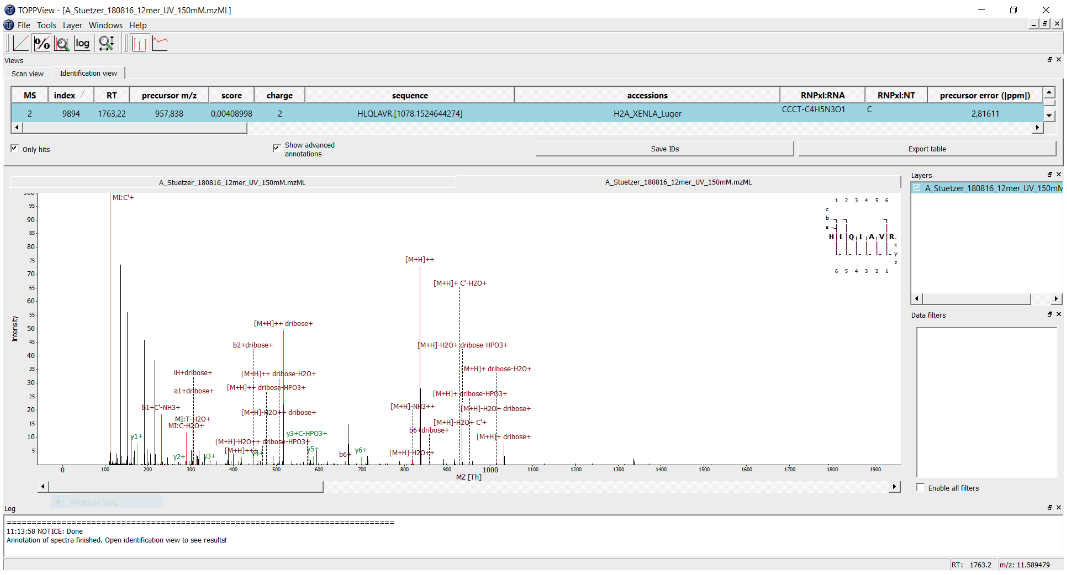
TOPPView - [A_Stuetzer_180816_12mer_UV_150mM.mzML]
MS | index | RT | precursor m/z | score | charge | sequence | accessions | RNPxl:RNA | RNPxl:NT | precursor error (|ppm|)
2 | 9894 | 1763,22 | 957,838 | 0,00408998 | 2 | HLQLAVR.[1078.1524644274] | H2A_XENLA_Luger | CCCT-C4H5N3O1 | C | 2,81611
11:13:58 NOTICE: Done
Annotation of spectra finished. Open identification view to see results!

49)

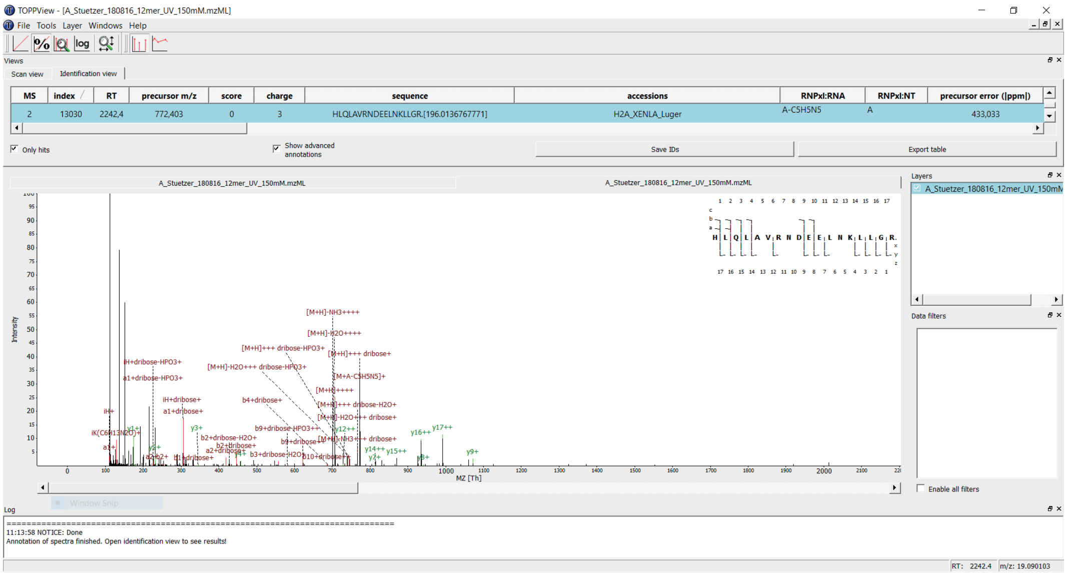
TOPPView - [A_Stuetzer_180816_12mer_UV_150mM.mzML]
File Tools Layer Windows Help
Scan view
Identification view
MS | index | RT | precursor m/z | score | charge | sequence | accessions | RNPxl:RNA | RNPxl:NT | precursor error (|ppm|)
2 | 13030 | 2242,4 | 772,403 | 0 | 3 | HLQLAVRNDEELNKLLGR.[196.0136767771] | H2A_XENLA_Luger | A-C5H5N5 | A | 433,033
Only hits
Show advanced annotations
Save IDs
Export table
A_Stuetzer_180816_12mer_UV_150mM.mzML
Layers
Data filters
Enable all filters
Intensity
MZ [Th]
Log
11:13:58 NOTICE: Done
Annotation of spectra finished. Open identification view to see results!
RT: 2242.4
m/z: 19.090103

50)

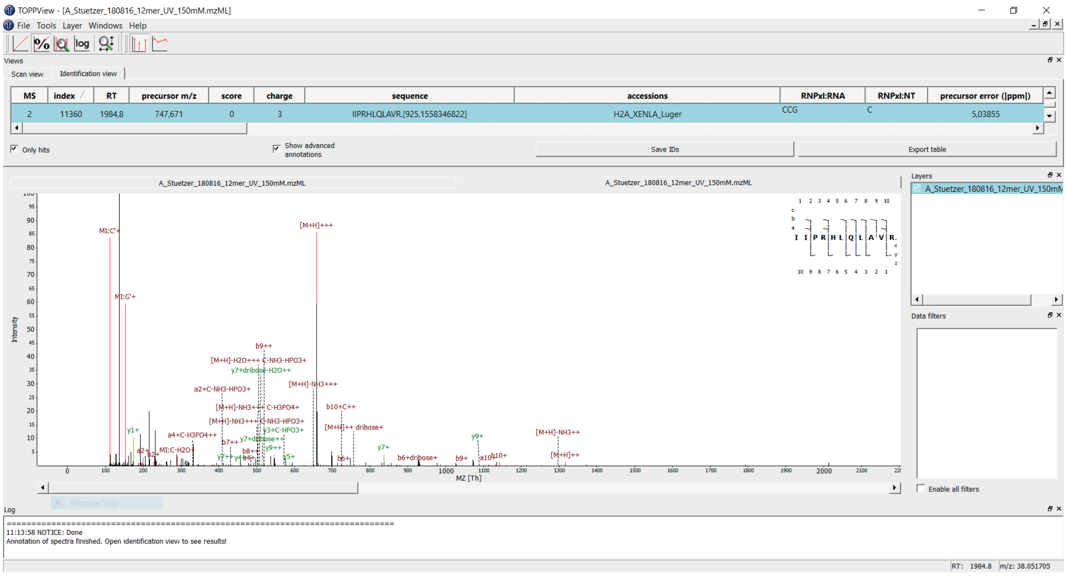
TOPPView - [A_Stuetzer_180816_12mer_UV_150mM.mzML]
File Tools Layer Windows Help
Scan view
Identification view
MS | index | RT | precursor m/z | score | charge | sequence | accessions | RNPxl:RNA | RNPxl:NT | precursor error (|ppm|)
2 | 11360 | 1984,8 | 747,671 | 0 | 3 | IIPRHLQLAVR.[925.1558346822] | H2A_XENLA_Luger | CCG | C | 5,03855
Only hits
Show advanced annotations
Save IDs
Export table
A_Stuetzer_180816_12mer_UV_150mM.mzML
Layers
Data filters
Enable all filters
Intensity
MZ [Th]
Log
11:13:58 NOTICE: Done
Annotation of spectra finished. Open identification view to see results!
RT: 1984.8
m/z: 38.051705

51)

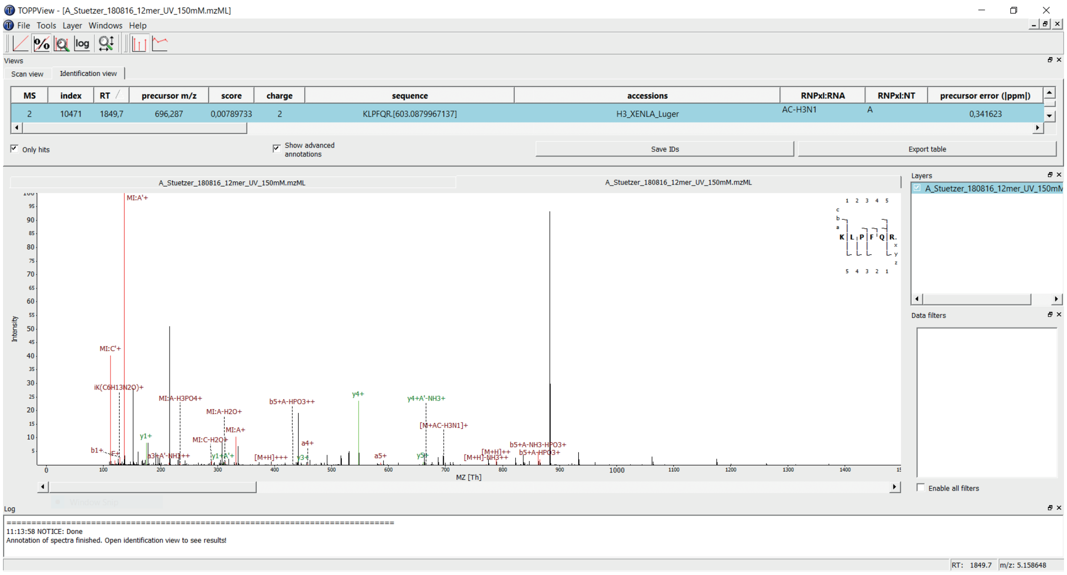
TOPPView - [A_Stuetzer_180816_12mer_UV_150mM.mzML]
File Tools Layer Windows Help
Scan view
Identification view
MS | index | RT | precursor m/z | score | charge | sequence | accessions | RNPxl:RNA | RNPxl:NT | precursor error (|ppm|)
2 | 10471 | 1849,7 | 696,287 | 0,00789733 | 2 | KLPFQR.[603.0879967137] | H3_XENLA_Luger | AC-H3N1 | A | 0,341623
Only hits
Show advanced annotations
Save IDs
Export table
A_Stuetzer_180816_12mer_UV_150mM.mzML
Layers
Data filters
Enable all filters
Intensity
MZ [Th]
Log
11:13:58 NOTICE: Done
Annotation of spectra finished. Open identification view to see results!
RT: 1849.7
m/z: 5.158648

52)

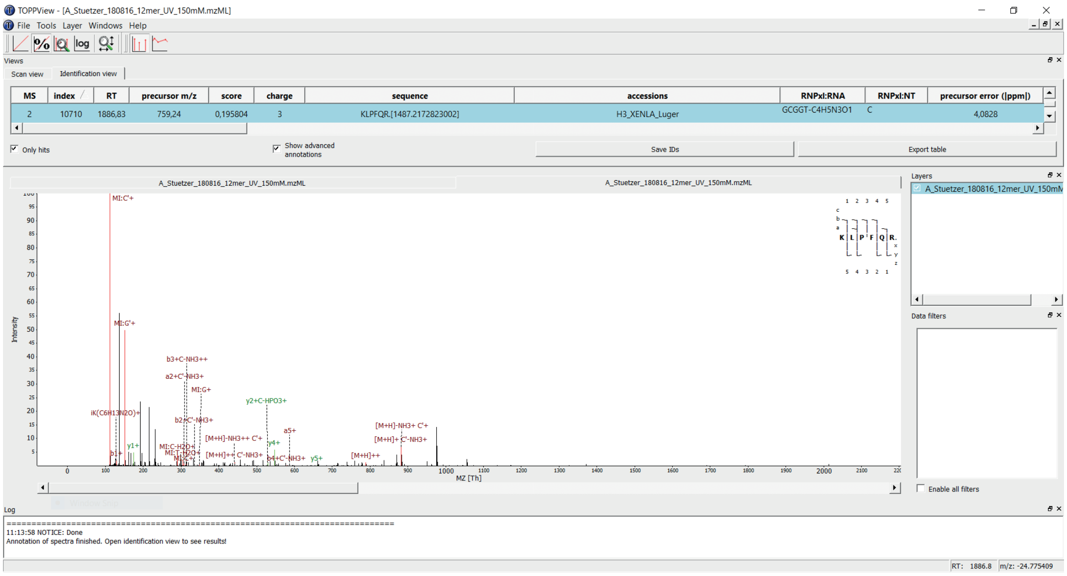
TOPPView - [A_Stuetzer_180816_12mer_UV_150mM.mzML]
MS | index | RT | precursor m/z | score | charge | sequence | accessions | RNPxl:RNA | RNPxl:NT | precursor error (|ppm|)
2 | 10710 | 1886,83 | 759,24 | 0,195804 | 3 | KLPFQR.[1487.2172823002] | H3_XENLA_Luger | GCGGT-C4H5N3O1 | C | 4,0828
11:13:58 NOTICE: Done
Annotation of spectra finished. Open identification view to see results!

53)

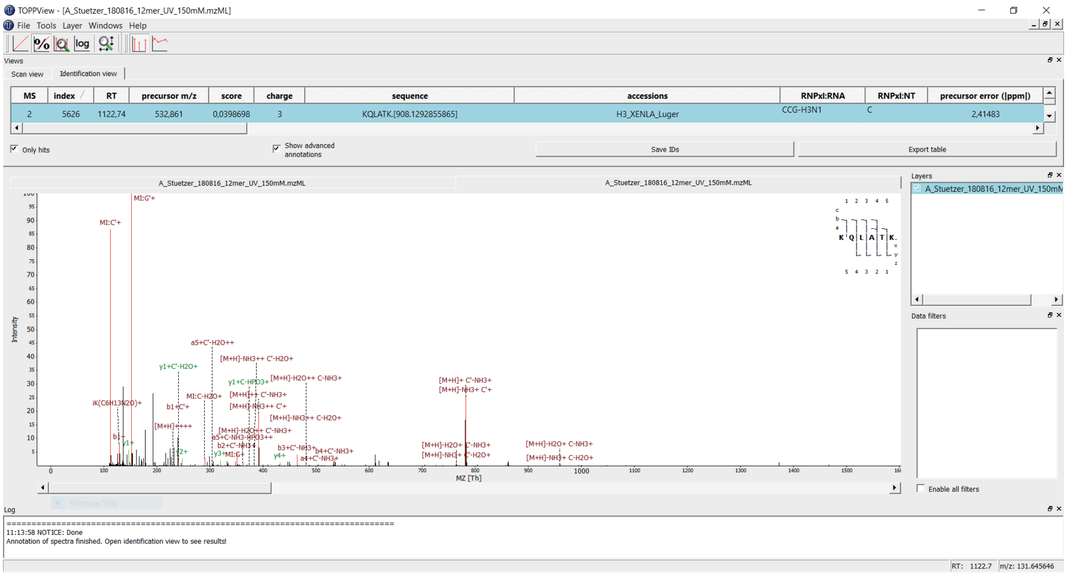
TOPPView - [A_Stuetzer_180816_12mer_UV_150mM.mzML]
MS | index | RT | precursor m/z | score | charge | sequence | accessions | RNPxl:RNA | RNPxl:NT | precursor error (|ppm|)
2 | 5626 | 1122,74 | 532,861 | 0,0398698 | 3 | KQLATK.[908.1292855865] | H3_XENLA_Luger | CCG-H3N1 | C | 2,41483
11:13:58 NOTICE: Done
Annotation of spectra finished. Open identification view to see results!

54)

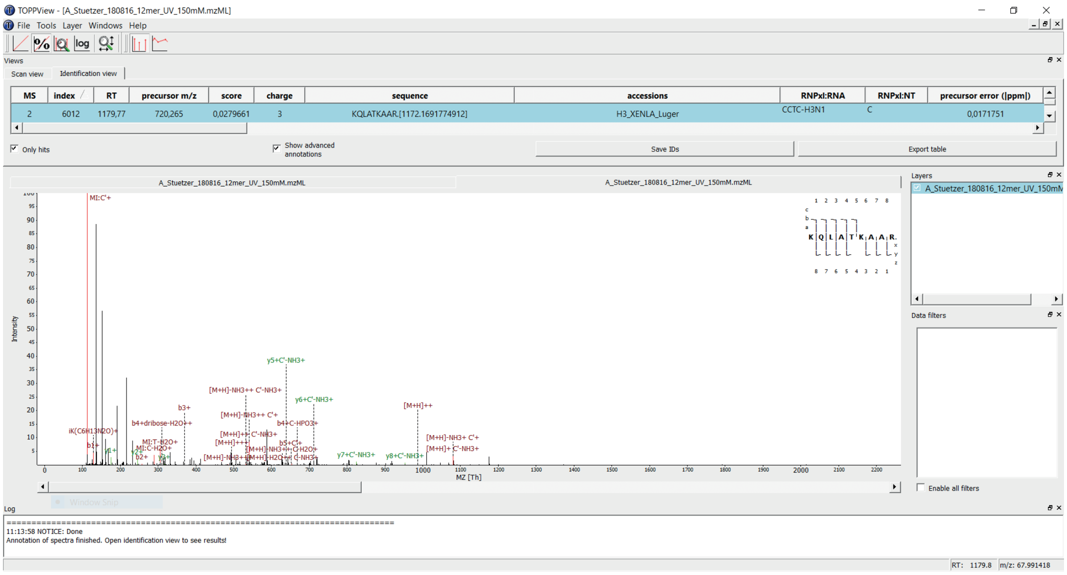
TOPPView - [A_Stuetzer_180816_12mer_UV_150mM.mzML]
MS | index | RT | precursor m/z | score | charge | sequence | accessions | RNPxl:RNA | RNPxl:NT | precursor error (|ppm|)
2 | 6012 | 1179,77 | 720,265 | 0,0279661 | 3 | KQLATKAAR.[1172.1691774912] | H3_XENLA_Luger | CCTC-H3N1 | C | 0,0171751
11:13:58 NOTICE: Done
Annotation of spectra finished. Open identification view to see results!

55)

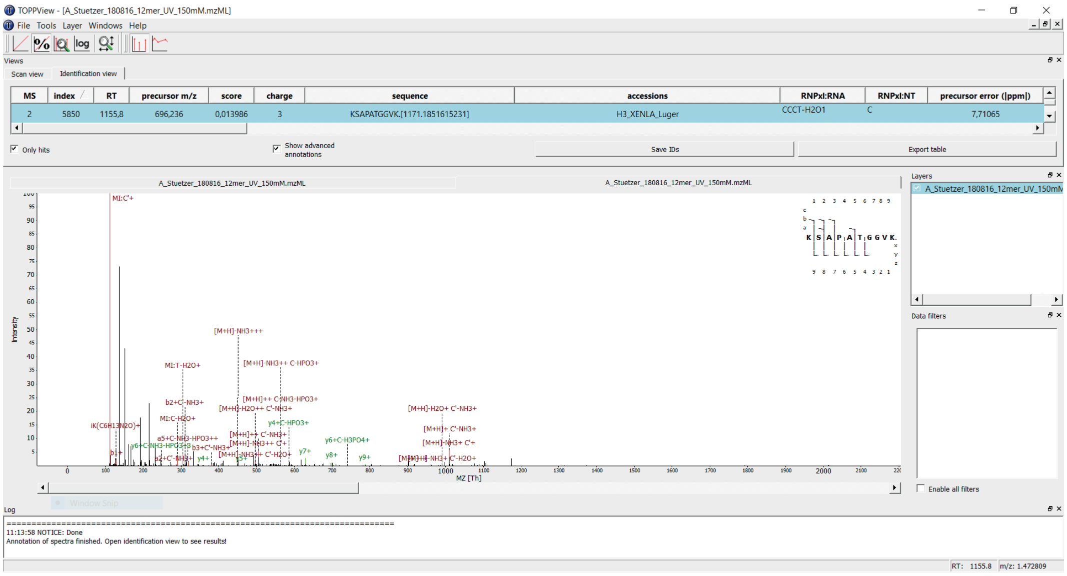
TOPPView - [A_Stuetzer_180816_12mer_UV_150mM.mzML]
File Tools Layer Windows Help
Scan view
Identification view
MS | index | RT | precursor m/z | score | charge | sequence | accessions | RNPxl:RNA | RNPxl:NT | precursor error (|ppm|)
2 | 5850 | 1155,8 | 696,236 | 0,013986 | 3 | KSAPATGGVK.[1171.1851615231] | H3_XENLA_Luger | CCCT-H2O1 | C | 7,71065
Only hits
Show advanced annotations
Save IDs
Export table
A_Stuetzer_180816_12mer_UV_150mM.mzML
MI:C'+
[M+H]-NH3+++
MI:T-H2O+
[M+H]-NH3++ C-HPO3+
MZ [Th]
Intensity
Layers
Data filters
Enable all filters
Log
11:13:58 NOTICE: Done
Annotation of spectra finished. Open identification view to see results!
RT: 1155.8
m/z: 1.472809

56)

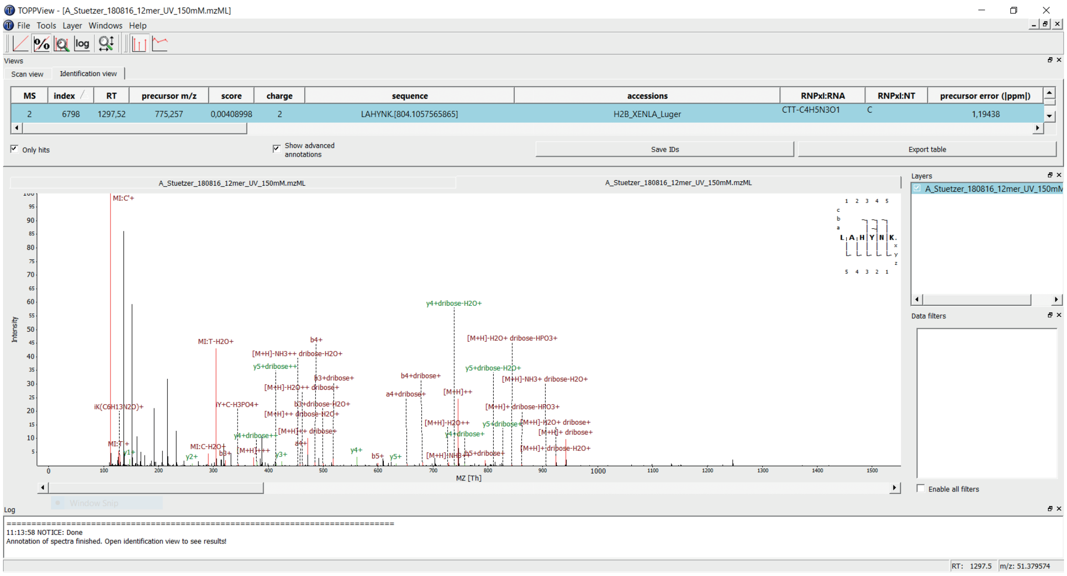
TOPPView - [A_Stuetzer_180816_12mer_UV_150mM.mzML]
File Tools Layer Windows Help
Scan view
Identification view
MS | index | RT | precursor m/z | score | charge | sequence | accessions | RNPxl:RNA | RNPxl:NT | precursor error (|ppm|)
2 | 6798 | 1297,52 | 775,257 | 0,00408998 | 2 | LAHYNK.[804.1057565865] | H2B_XENLA_Luger | CTT-C4H5N3O1 | C | 1,19438
Only hits
Show advanced annotations
Save IDs
Export table
A_Stuetzer_180816_12mer_UV_150mM.mzML
MI:C'+
y4+dribose-H2O+
[M+H]-H2O+ dribose-HPO3+
MI:T-H2O+
MZ [Th]
Intensity
Layers
Data filters
Enable all filters
Log
11:13:58 NOTICE: Done
Annotation of spectra finished. Open identification view to see results!
RT: 1297.5
m/z: 51.379574

57)

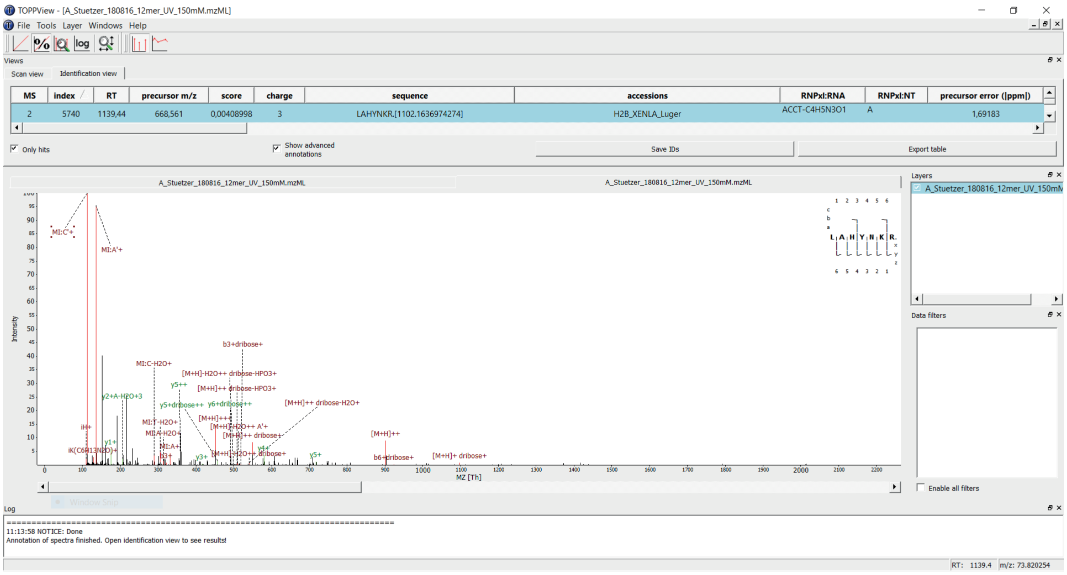
TOPPView - [A_Stuetzer_180816_12mer_UV_150mM.mzML]
File Tools Layer Windows Help
Scan view
Identification view
MS | index | RT | precursor m/z | score | charge | sequence | accessions | RNPxl:RNA | RNPxl:NT | precursor error (|ppm|)
2 | 5740 | 1139,44 | 668,561 | 0,00408998 | 3 | LAHYNKR.[1102.1636974274] | H2B_XENLA_Luger | ACCT-C4H5N3O1 | A | 1,69183
Only hits
Show advanced annotations
Save IDs
Export table
A_Stuetzer_180816_12mer_UV_150mM.mzML
MI:C'+
MI:A'+
b3+dribose+
MI:C-H2O+
MZ [Th]
Intensity
Layers
Data filters
Enable all filters
Log
11:13:58 NOTICE: Done
Annotation of spectra finished. Open identification view to see results!
RT: 1139.4
m/z: 73.820254

58)

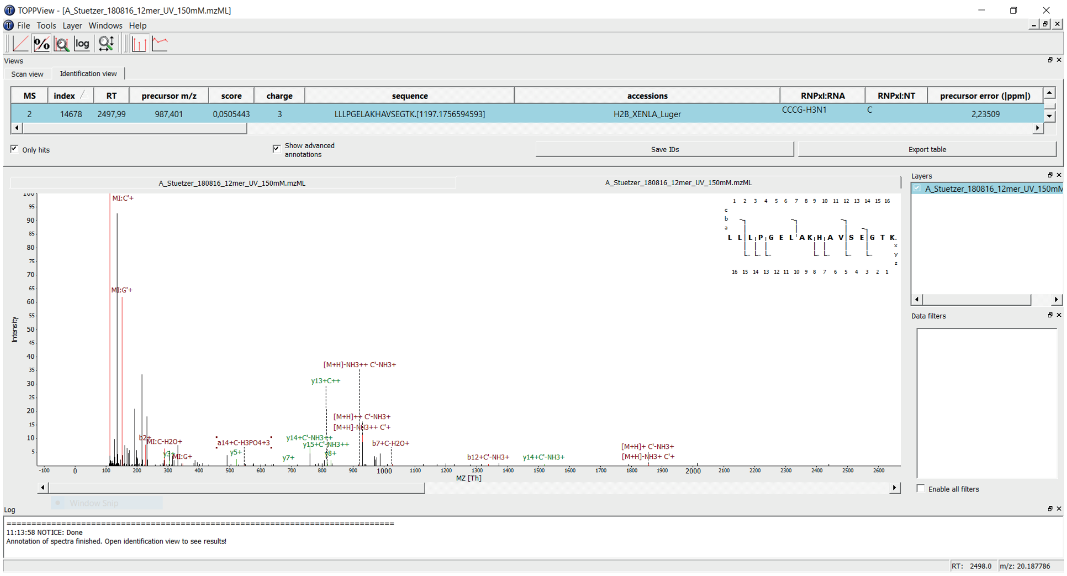
TOPPView - [A_Stuetzer_180816_12mer_UV_150mM.mzML]
MS | index | RT | precursor m/z | score | charge | sequence | accessions | RNPxl:RNA | RNPxl:NT | precursor error (|ppm|)
2 | 14678 | 2497,99 | 987,401 | 0,0505443 | 3 | LLLPGELAKHAVSEGTK.[1197.1756594593] | H2B_XENLA_Luger | CCCG-H3N1 | C | 2,23509
A_Stuetzer_180816_12mer_UV_150mM.mzML
11:13:58 NOTICE: Done
Annotation of spectra finished. Open identification view to see results!

59)

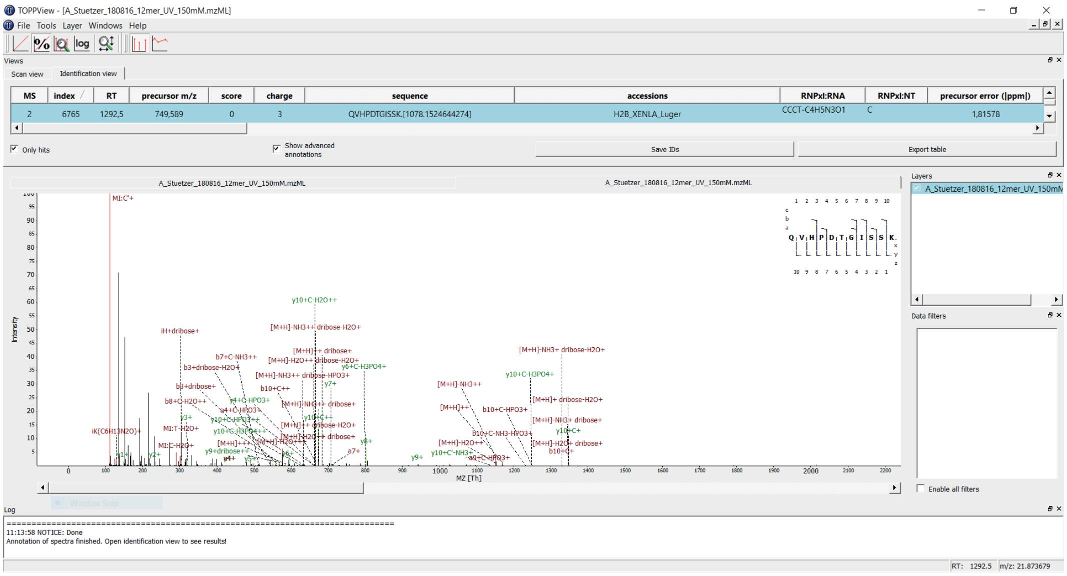
TOPPView - [A_Stuetzer_180816_12mer_UV_150mM.mzML]
MS | index | RT | precursor m/z | score | charge | sequence | accessions | RNPxl:RNA | RNPxl:NT | precursor error (|ppm|)
2 | 6765 | 1292,5 | 749,589 | 0 | 3 | QVHPDTGISSK.[1078.1524644274] | H2B_XENLA_Luger | CCCT-C4H5N3O1 | C | 1,81578
A_Stuetzer_180816_12mer_UV_150mM.mzML
11:13:58 NOTICE: Done
Annotation of spectra finished. Open identification view to see results!

60)

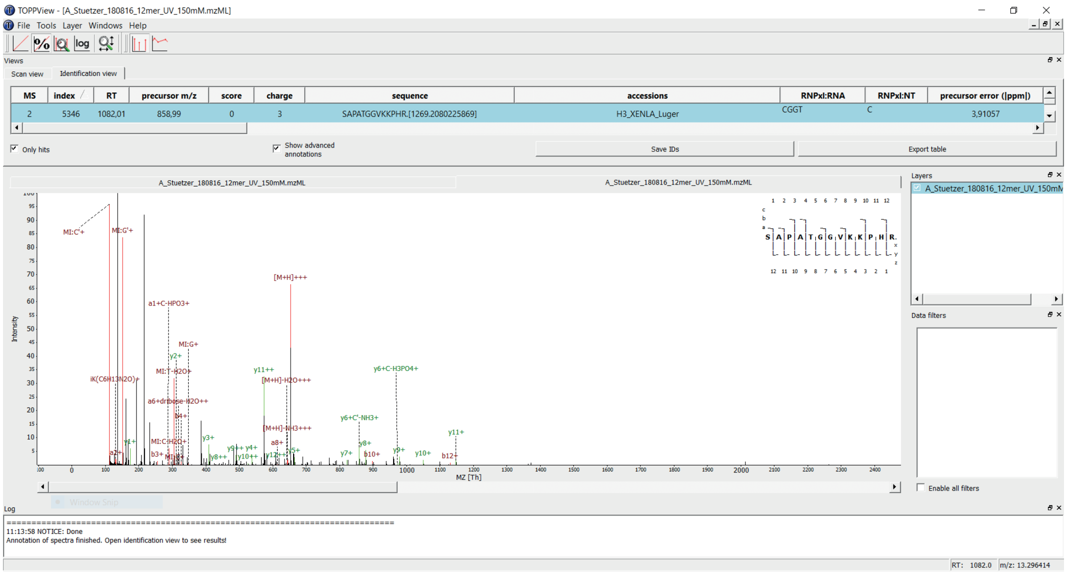
TOPPView - [A_Stuetzer_180816_12mer_UV_150mM.mzML]
MS | index | RT | precursor m/z | score | charge | sequence | accessions | RNPxl:RNA | RNPxl:NT | precursor error (|ppm|)
2 | 5346 | 1082,01 | 858,99 | 0 | 3 | SAPATGGVKKPHR.[1269.2080225869] | H3_XENLA_Luger | CGGT | C | 3,91057
A_Stuetzer_180816_12mer_UV_150mM.mzML
11:13:58 NOTICE: Done
Annotation of spectra finished. Open identification view to see results!

61)

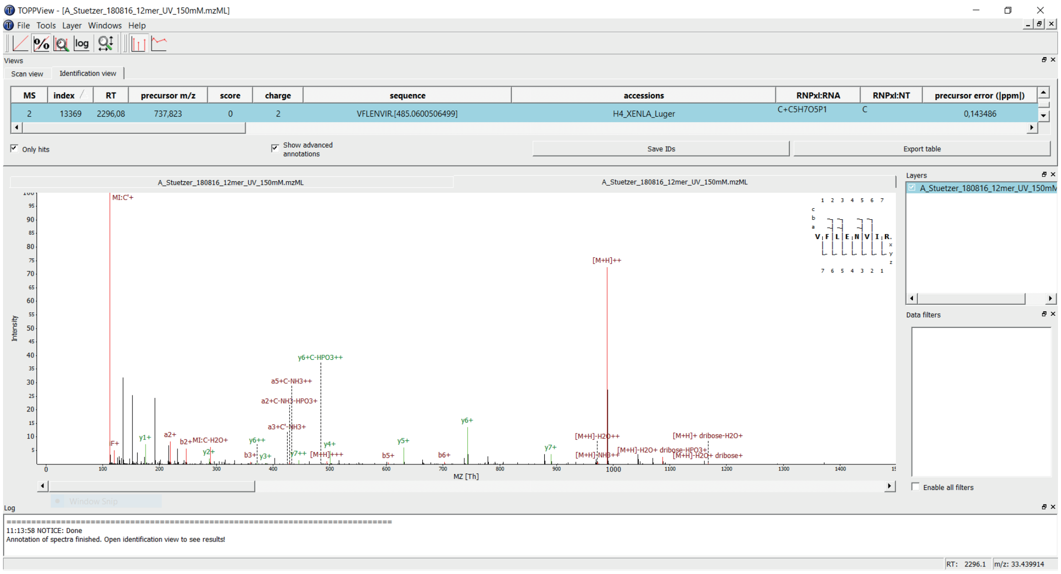
TOPPView - [A_Stuetzer_180816_12mer_UV_150mM.mzML]
MS | index | RT | precursor m/z | score | charge | sequence | accessions | RNPxl:RNA | RNPxl:NT | precursor error (|ppm|)
2 | 13369 | 2296,08 | 737,823 | 0 | 2 | VFLENVIR.[485.0600506499] | H4_XENLA_Luger | C+C5H7O5P1 | C | 0,143486
11:13:58 NOTICE: Done
Annotation of spectra finished. Open identification view to see results!

62)

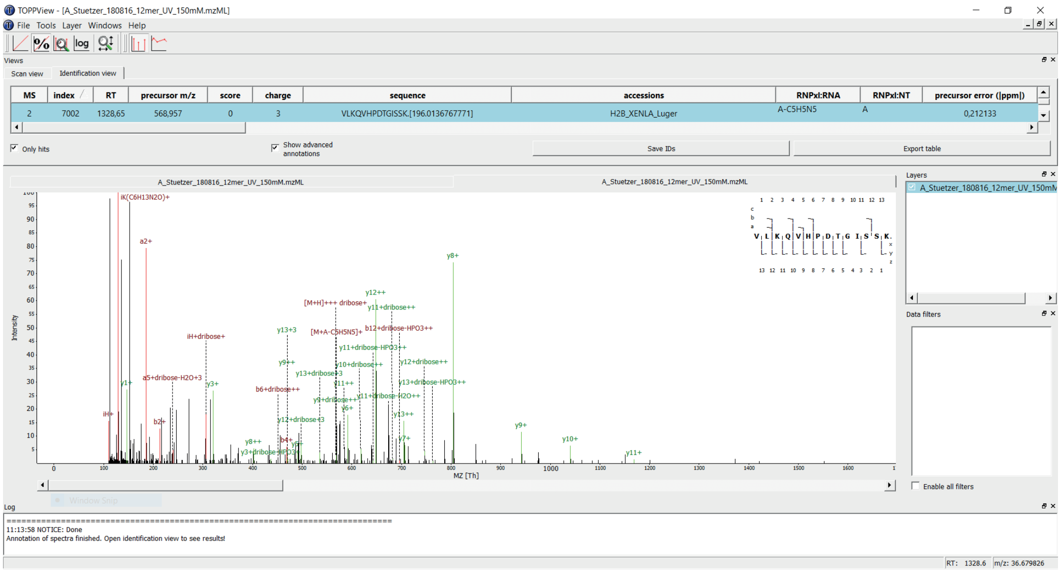
TOPPView - [A_Stuetzer_180816_12mer_UV_150mM.mzML]
MS | index | RT | precursor m/z | score | charge | sequence | accessions | RNPxl:RNA | RNPxl:NT | precursor error (|ppm|)
2 | 7002 | 1328,65 | 568,957 | 0 | 3 | VLKQVHPDTGISSK.[196.0136767771] | H2B_XENLA_Luger | A-C5H5N5 | A | 0,212133
11:13:58 NOTICE: Done
Annotation of spectra finished. Open identification view to see results!

63)

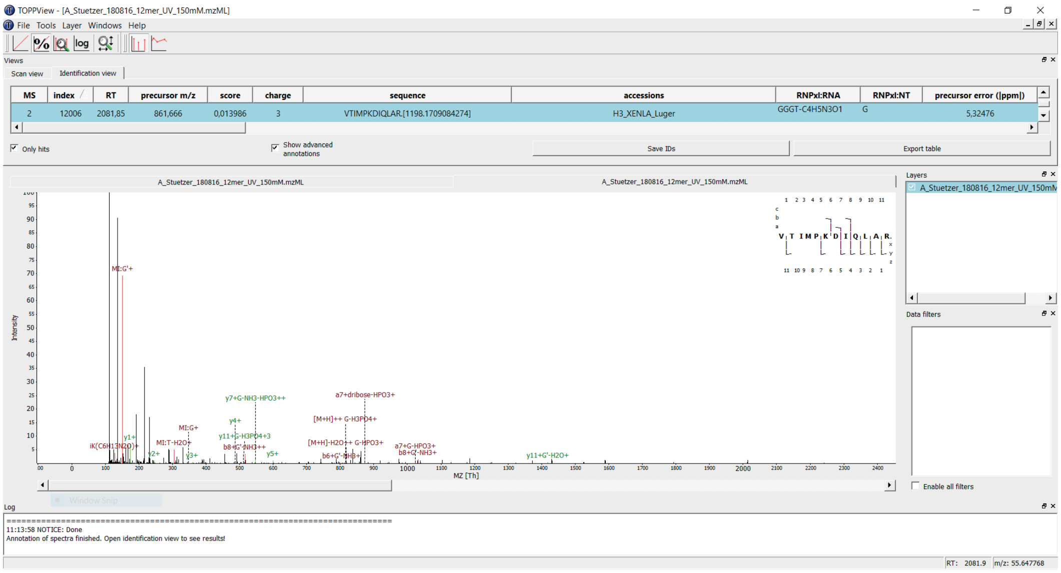
TOPPView - [A_Stuetzer_180816_12mer_UV_150mM.mzML]
MS | index | RT | precursor m/z | score | charge | sequence | accessions | RNPxl:RNA | RNPxl:NT | precursor error (|ppm|)
2 | 12006 | 2081,85 | 861,666 | 0,013986 | 3 | VTIMPKDIQLAR.[1198.1709084274] | H3_XENLA_Luger | GGGT-C4H5N3O1 | G | 5,32476
11:13:58 NOTICE: Done
Annotation of spectra finished. Open identification view to see results!

64)

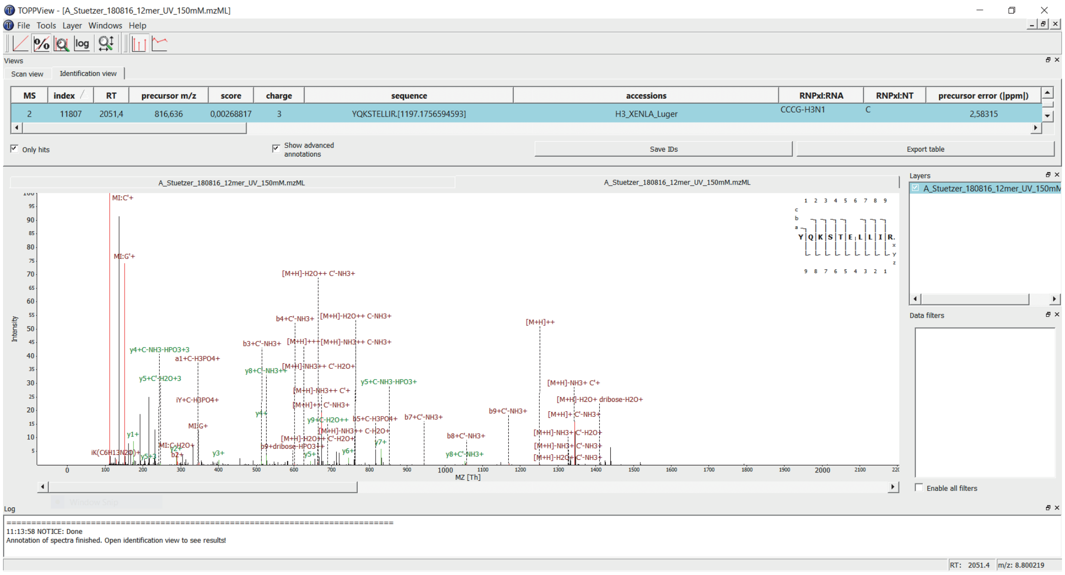
TOPPView - [A_Stuetzer_180816_12mer_UV_150mM.mzML]
File Tools Layer Windows Help
Scan view Identification view
MS | index | RT | precursor m/z | score | charge | sequence | accessions | RNPxl:RNA | RNPxl:NT | precursor error (|ppm|)
2 | 11807 | 2051,4 | 816,636 | 0,00268817 | 3 | YQKSTELLIR.[1197.1756594593] | H3_XENLA_Luger | CCCG-H3N1 | C | 2,58315
Only hits
Show advanced annotations
Save IDs
Export table
A_Stuetzer_180816_12mer_UV_150mM.mzML
Layers
Data filters
Enable all filters
MZ [Th]
Intensity
11:13:58 NOTICE: Done
Annotation of spectra finished. Open identification view to see results!
RT: 2051.4 m/z: 8.800219

65)

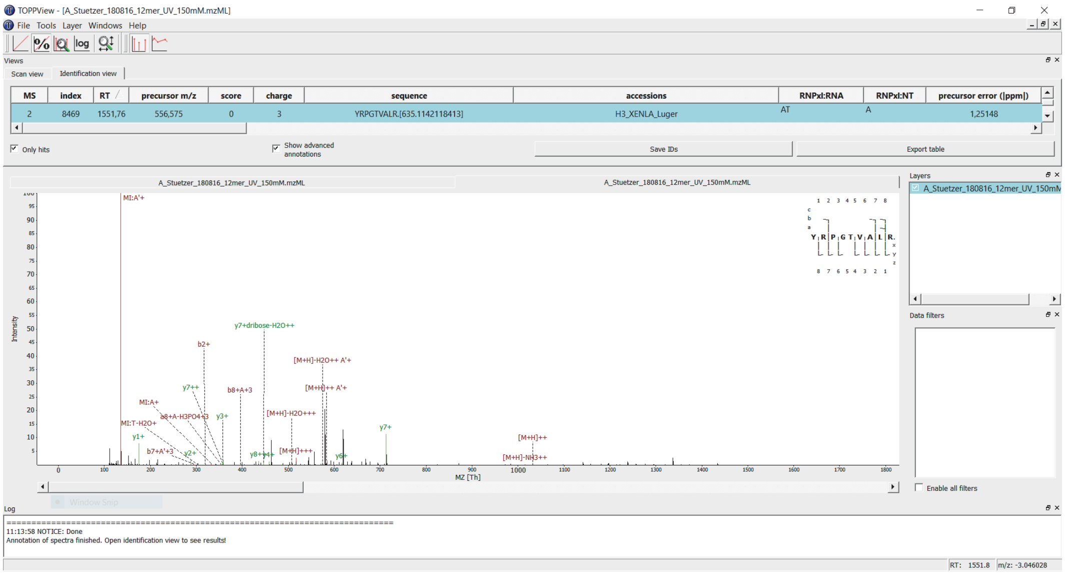
TOPPView - [A_Stuetzer_180816_12mer_UV_150mM.mzML]
File Tools Layer Windows Help
Scan view Identification view
MS | index | RT | precursor m/z | score | charge | sequence | accessions | RNPxl:RNA | RNPxl:NT | precursor error (|ppm|)
2 | 8469 | 1551,76 | 556,575 | 0 | 3 | YRPGTVALR.[635.1142118413] | H3_XENLA_Luger | AT | A | 1,25148
Only hits
Show advanced annotations
Save IDs
Export table
A_Stuetzer_180816_12mer_UV_150mM.mzML
Layers
Data filters
Enable all filters
MZ [Th]
Intensity
11:13:58 NOTICE: Done
Annotation of spectra finished. Open identification view to see results!
RT: 1551.8 m/z: -3.046028

66)

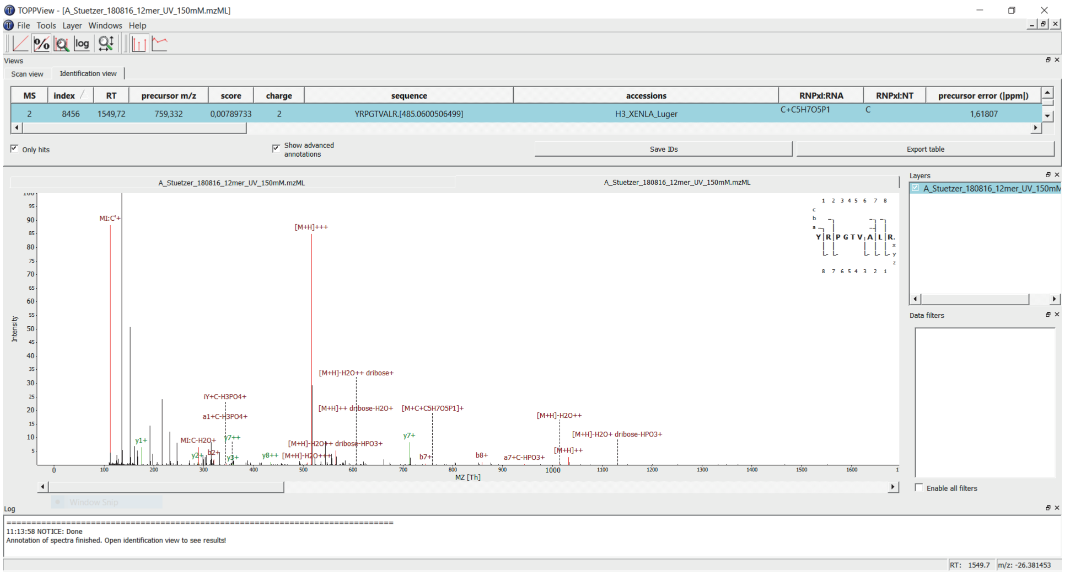
TOPPView - [A_Stuetzer_180816_12mer_UV_150mM.mzML]
File Tools Layer Windows Help
Scan view Identification view
MS | index | RT | precursor m/z | score | charge | sequence | accessions | RNPxl:RNA | RNPxl:NT | precursor error (|ppm|)
2 | 8456 | 1549,72 | 759,332 | 0,00789733 | 2 | YRPGTVALR.[485.0600506499] | H3_XENLA_Luger | C+C5H7O5P1 | C | 1,61807
Only hits
Show advanced annotations
Save IDs
Export table
A_Stuetzer_180816_12mer_UV_150mM.mzML
Layers
Data filters
Enable all filters
MZ [Th]
Intensity
11:13:58 NOTICE: Done
Annotation of spectra finished. Open identification view to see results!
RT: 1549.7 m/z: -26.381453
