# Supplementary material for: Analysis of protein-DNA interactions in chromatin by UV induced cross-linking and mass spectrometry
Source: Nat Commun. 2020 Oct 16;11:5250. doi: 10.1038/s41467-020-19047-7 (PMC7567871; doi:10.1038/s41467-020-19047-7)
Supplement: Supplementary file 6 — Supplementary Data 4 [file 41467_2020_19047_MOESM6_ESM.pdf]

Supplementary Data 4

Analysis of protein-DNA interactions in chromatin by UV induced cross-linking and mass spectrometry

Stützer *et al.*

List of contents

Annotated MS/MS spectra of SCML2 p. 1-5

TOPPView MS/MS spectra of SCML2 UV XL p. 6-9

TOPPView MS/MS spectra of SCML2 CTRL p. 9-11

TOPPView MS/MS spectra of SCML2 UV XL + H1.4 p. 11-16

TOPPView MS/MS spectra of SCML2 CTRL + H1.4 p. 16-18

Annotated MS/MS spectra of SCML2

1) SCML2, aa 41-53

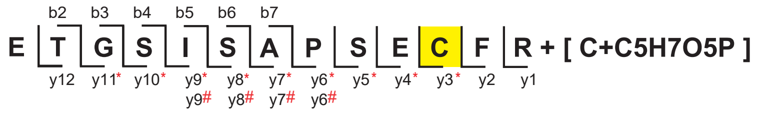

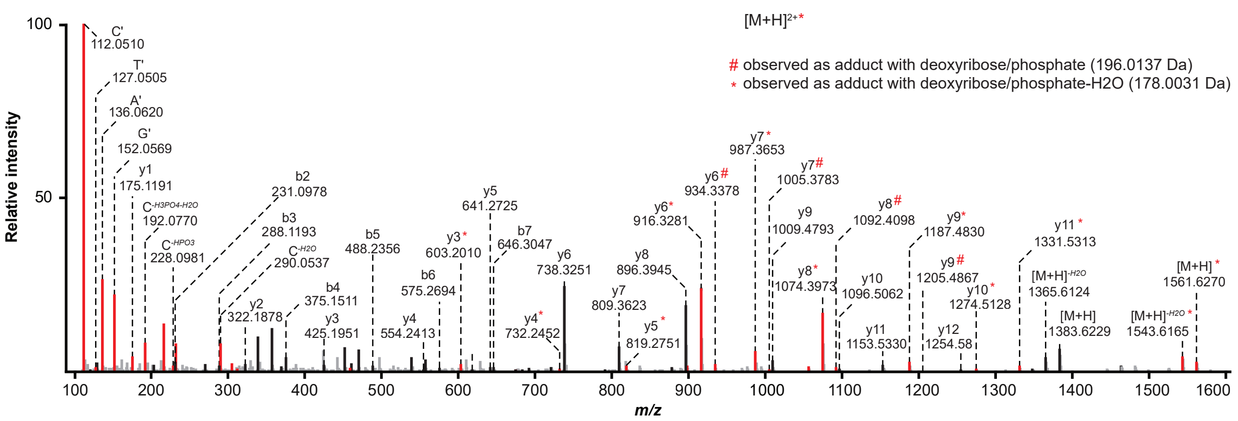

Index No. 12109; RT: 1814.69 s; *m/z*: 934.844; z: 2; score: 27.5404

2) SCML2, aa 109-123

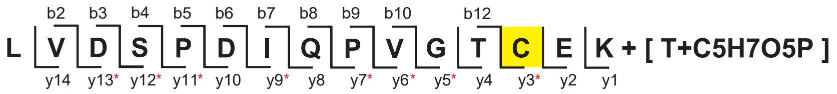

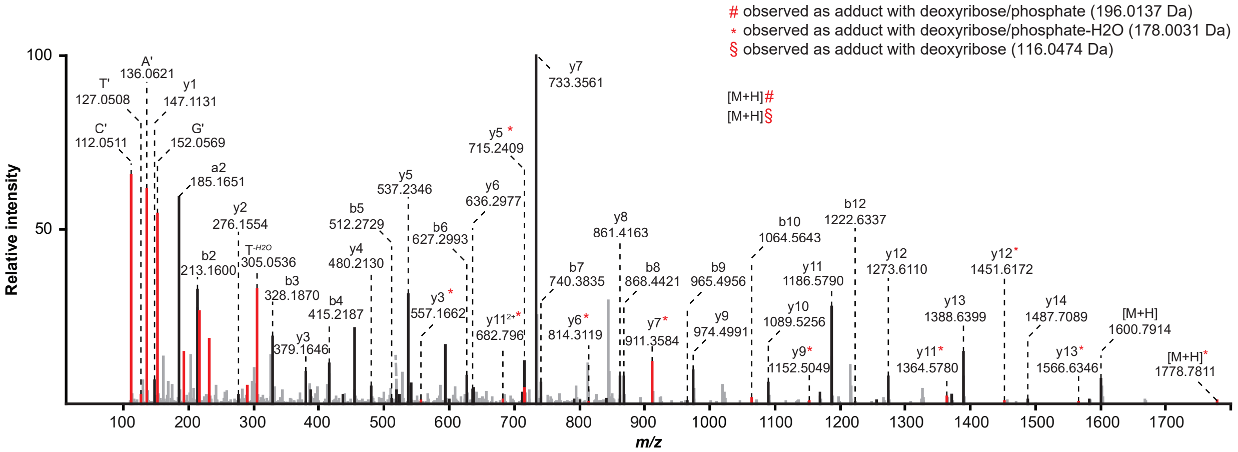

Index No. 14883; RT: 2149.14 s; *m/z*: 1050.93; z: 2; score: 42.1462

3) SCML2, aa 185-198

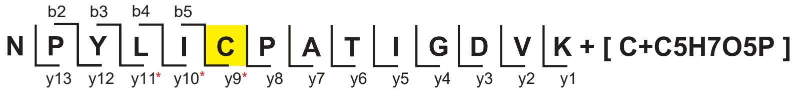

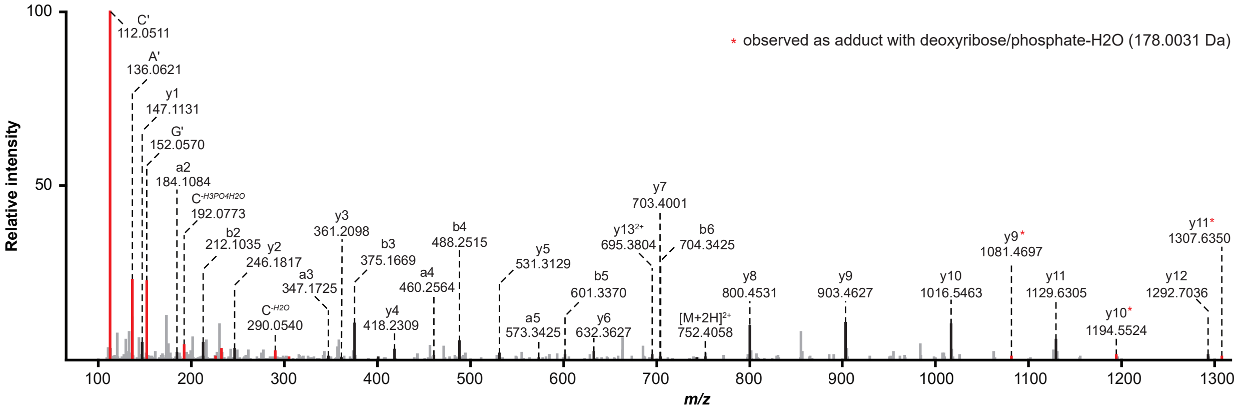

Index No. 18190; RT: 2580.36 s; *m/z*: 663.62; z: 3; score: 30.7801

4) SCML2, aa 223-231

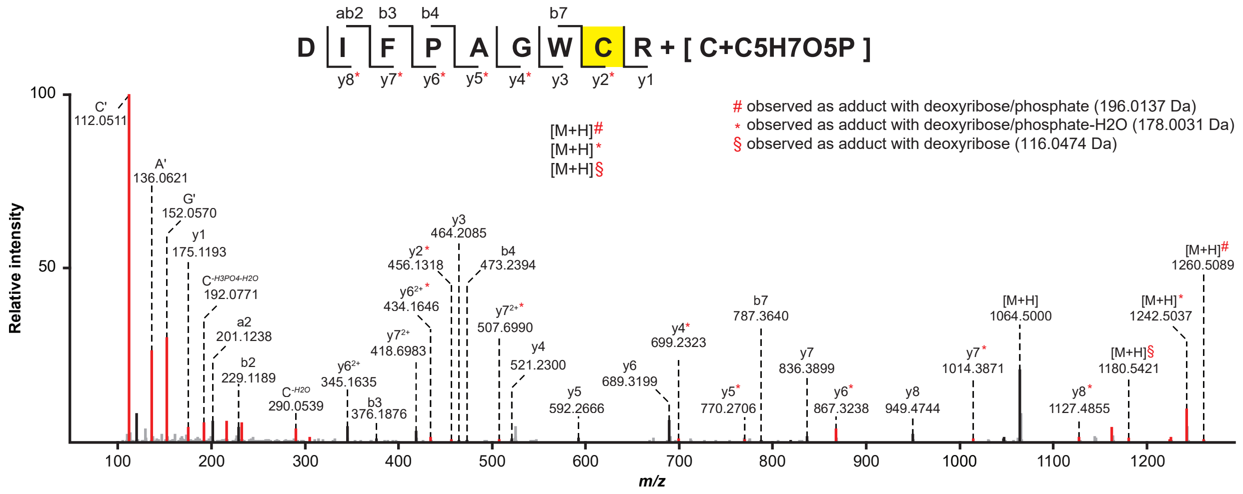

Index No. 18050; RT: 2561.58 s; *m/z*: 775.283; z: 2; score: 12.1732

5) SCML2, aa 317-322

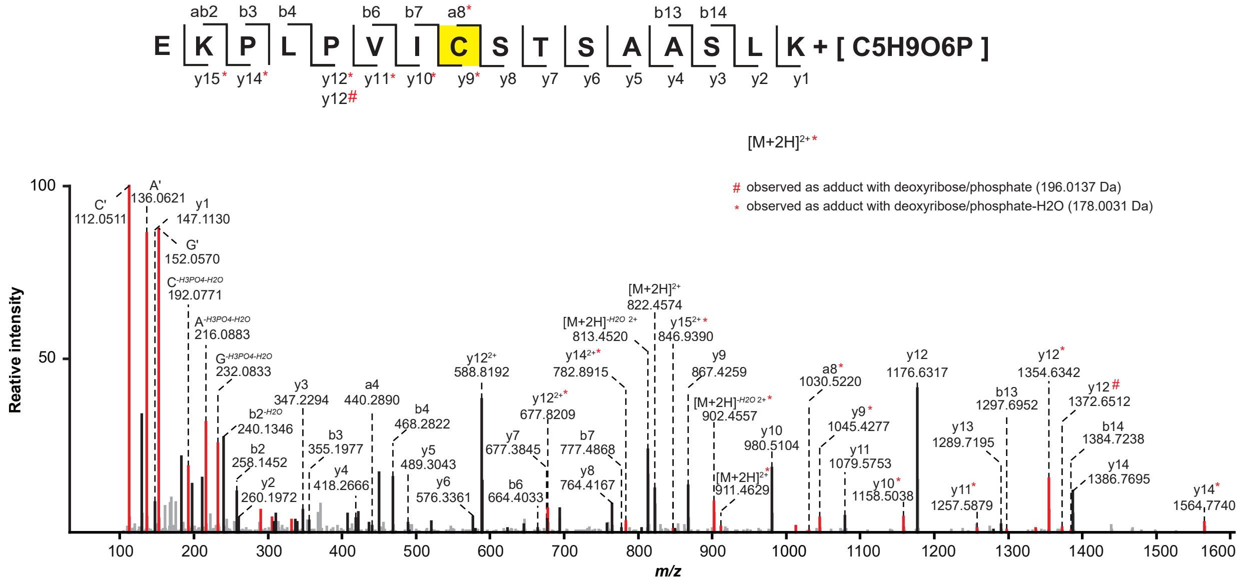

Index No. 14913; RT: 2152.83 s; *m/z*: 920.464; z: 2; score: 34.903

6) SCML2, aa 364-374

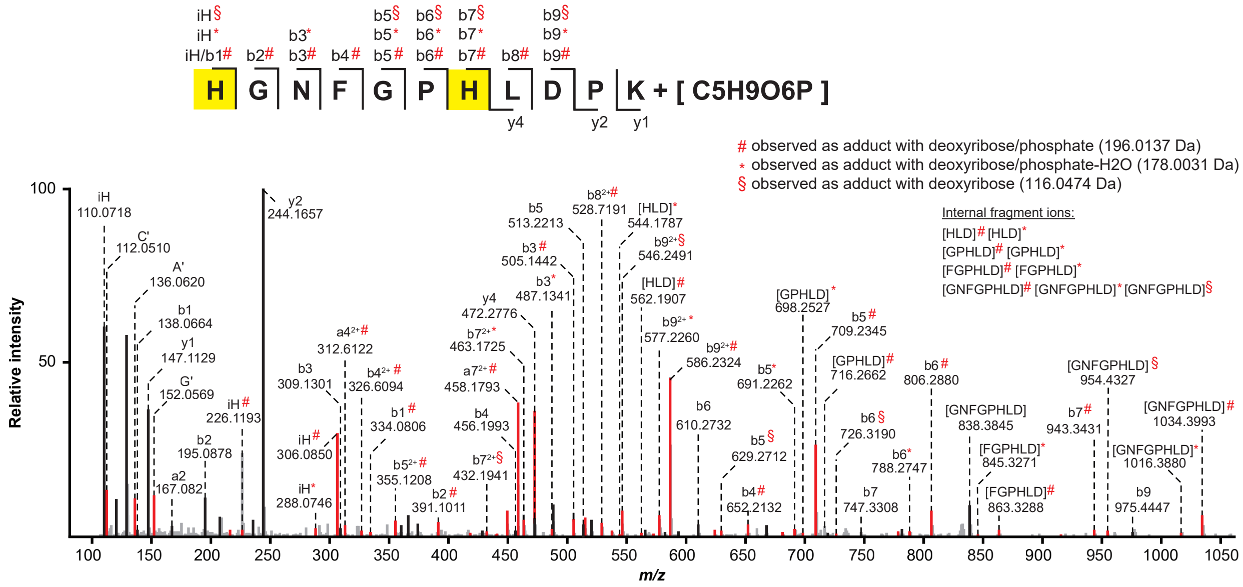

Index No. 9212; RT: 1493.54 s; *m/z*: 472.21; z: 3; score: 18.6023

7) SCML2, aa 376-393

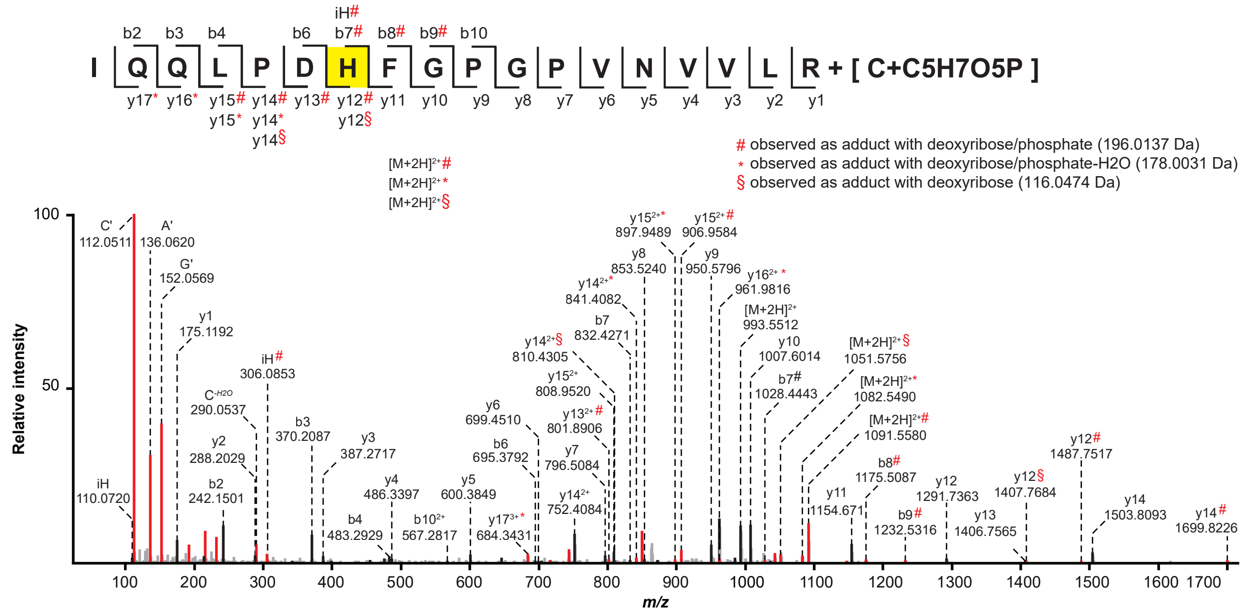

Index No. 18376; RT: 2604.64 s; *m/z*: 824.39; z: 3; score: 56.9496

8) SCML2, aa 408-418

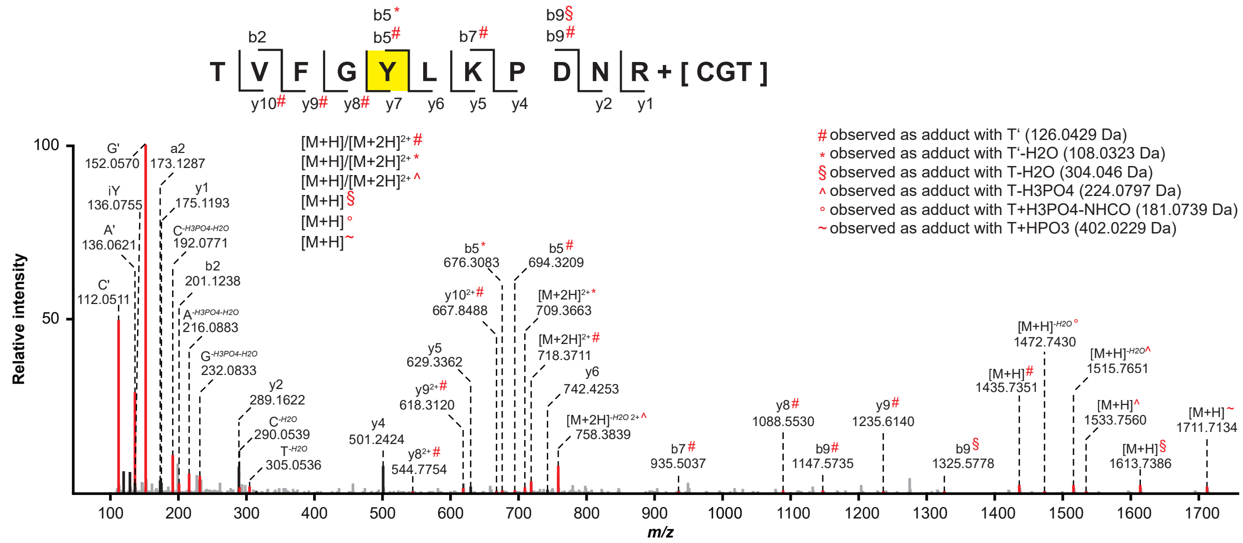

Index No. 14909; RT: 2193.9 s; *m/z*: 750.619; z: 3; score: 4.31377

9) SCML2, aa 499-513

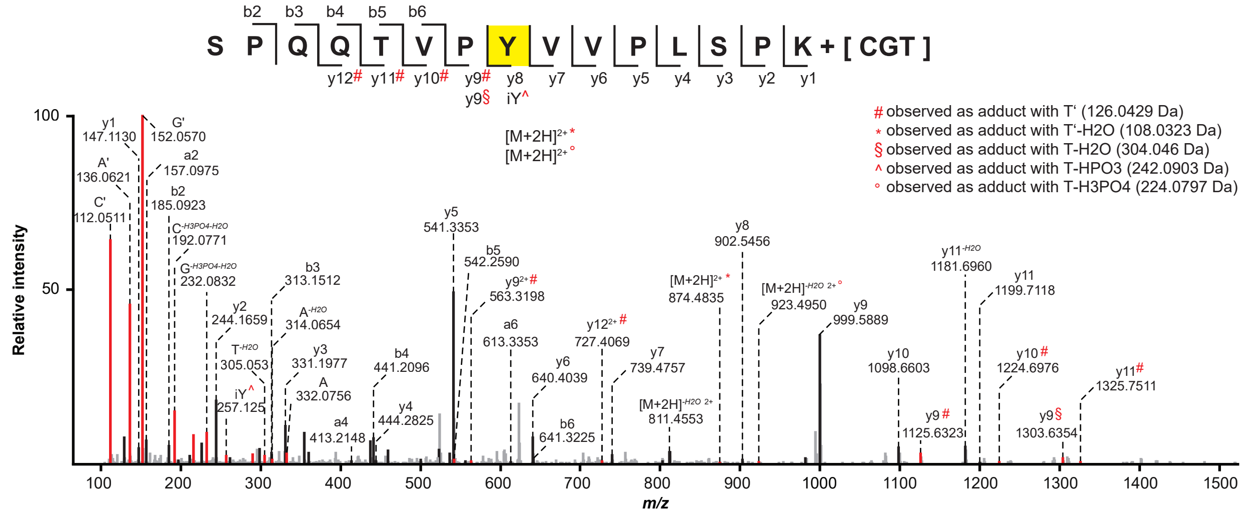

Index No. 16791; RT: 2433.97 s; *m/z*: 860.692; z: 3; score: 22.7313

10) SCML2, aa 517-536

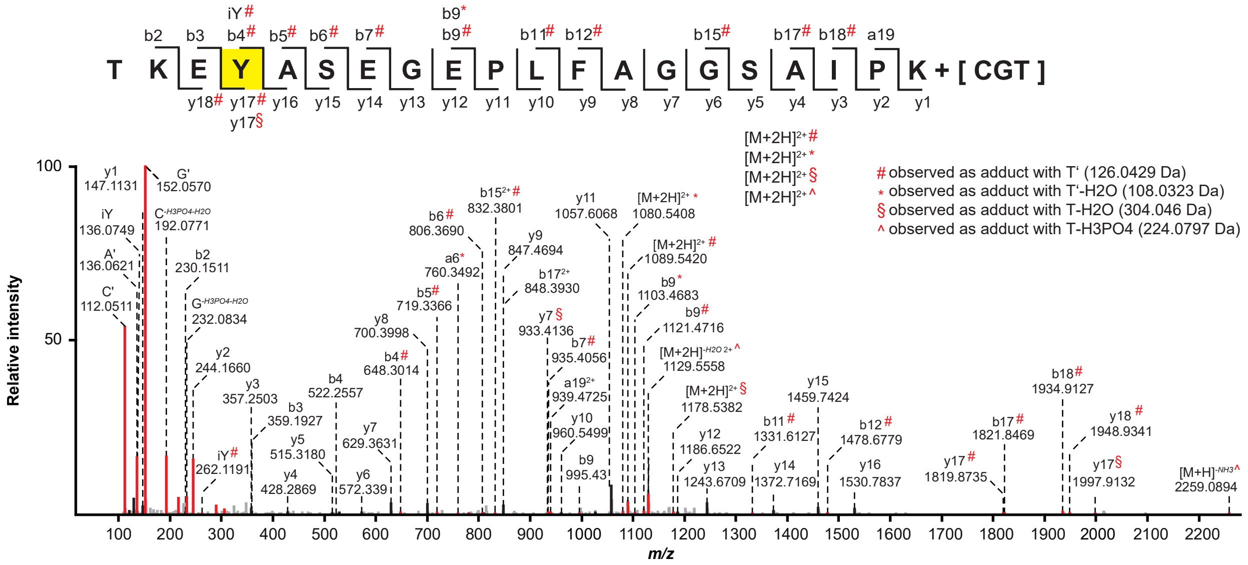

Index No. 15636; RT: 2284.79 s; *m/z*: 998.4; z: 3; score: 36.5399

11) SCML2, aa 605-621

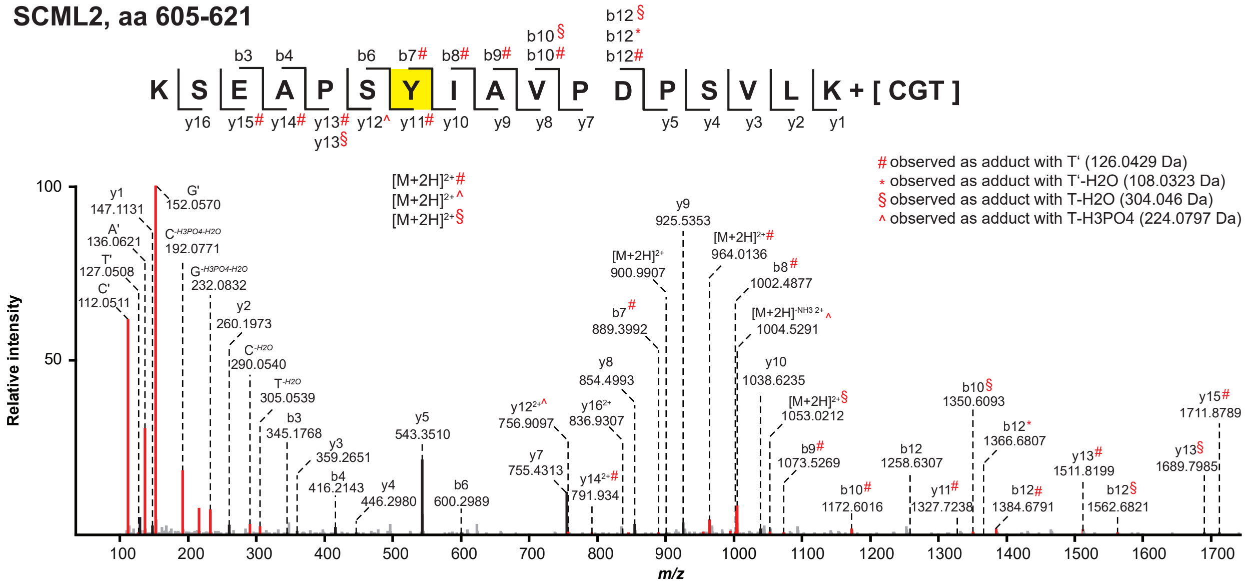

Index No. 16375; RT: 2379.71 s; *m/z*: 914.717; z: 3; score: 16.9748

12) SCML2, aa 642-656

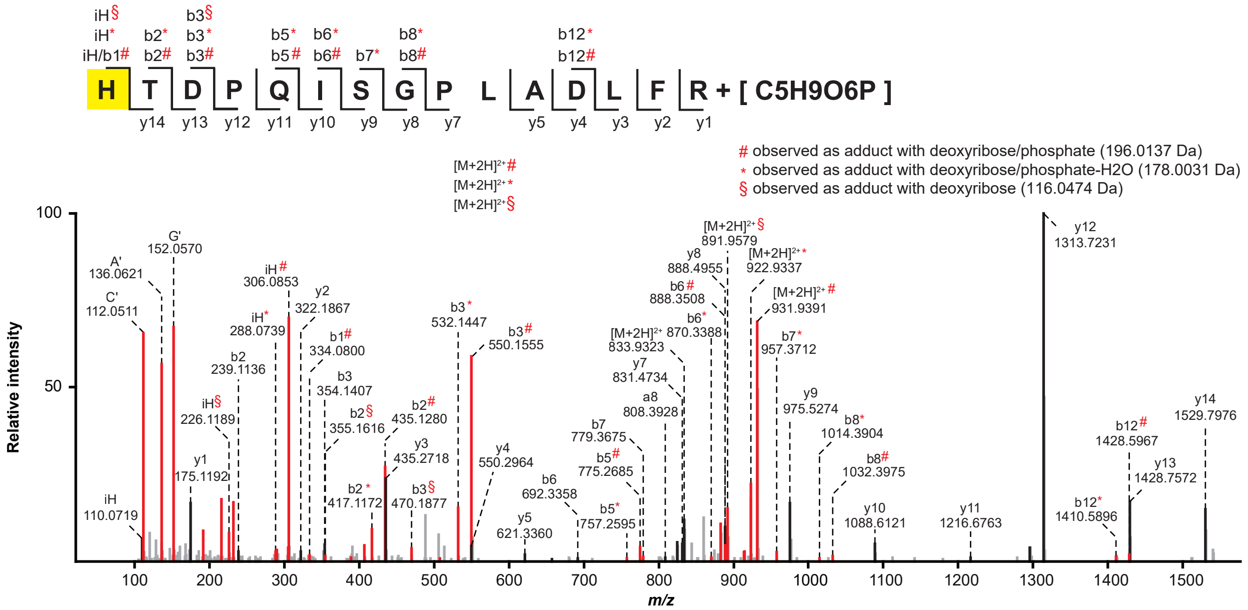

Index No. 20844; RT: 2929.88 s; *m/z*: 931.939; z: 2; score: 27.3861

13) SCML2, aa 687-693

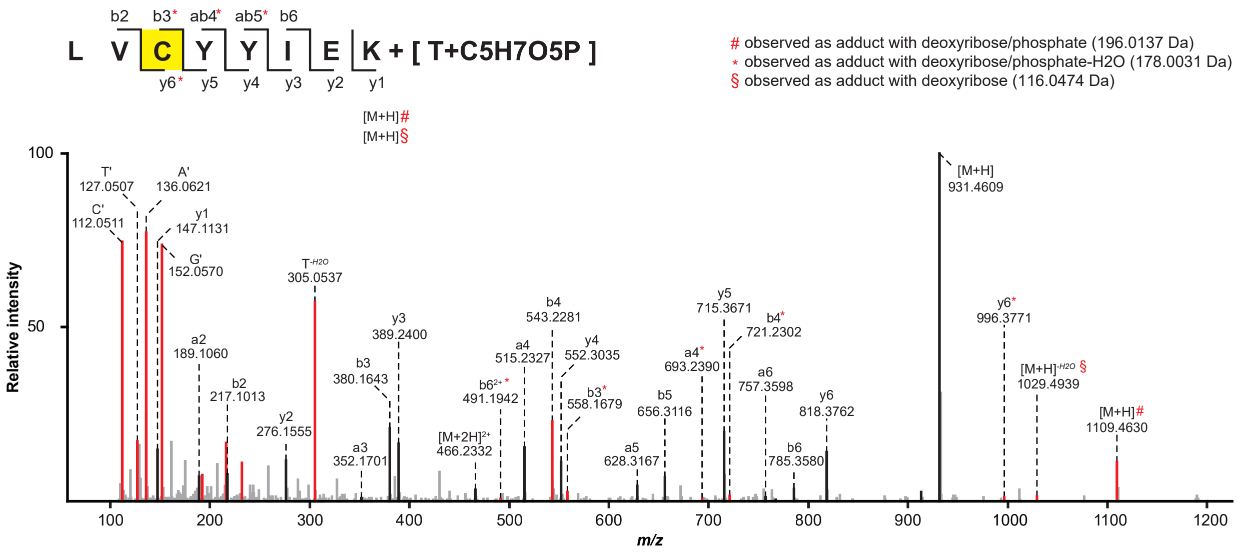

Index No. 15706; RT: 2254.15 s; *m/z*: 716.263; z: 2; score: 12.6367

14) SCML2, aa 687-693

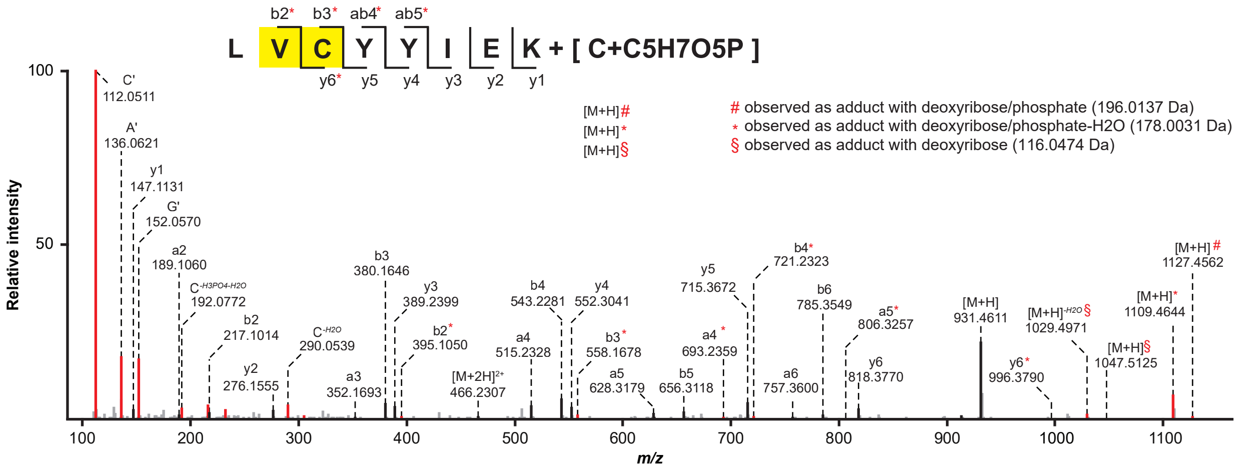

Index No. 13366; RT: 1966 s; *m/z*: 708.763; z: 2; score: 11.9248

TOPPView spectra of SCML2 UV XL

1)

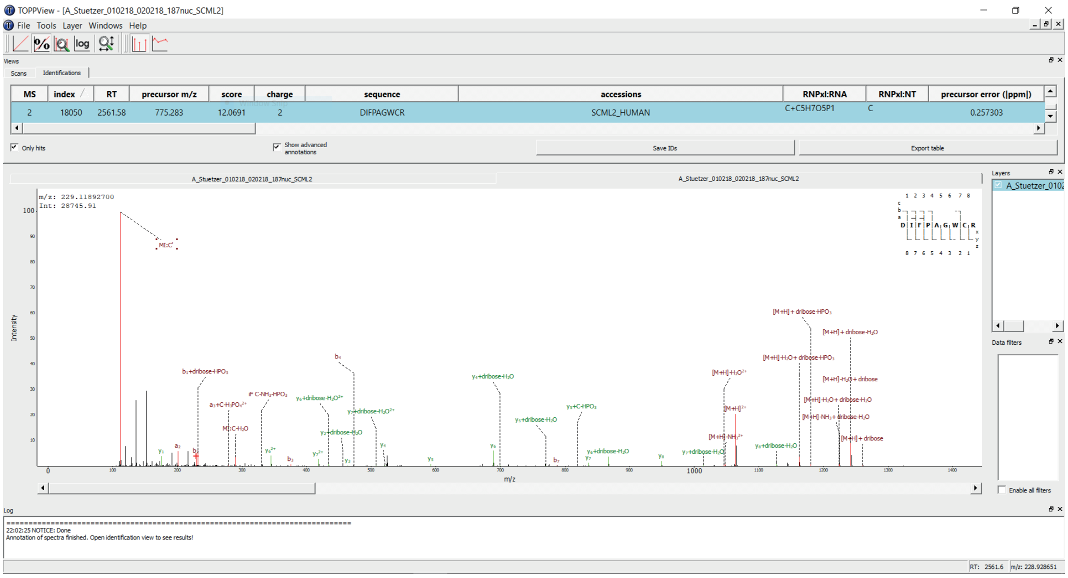

2)

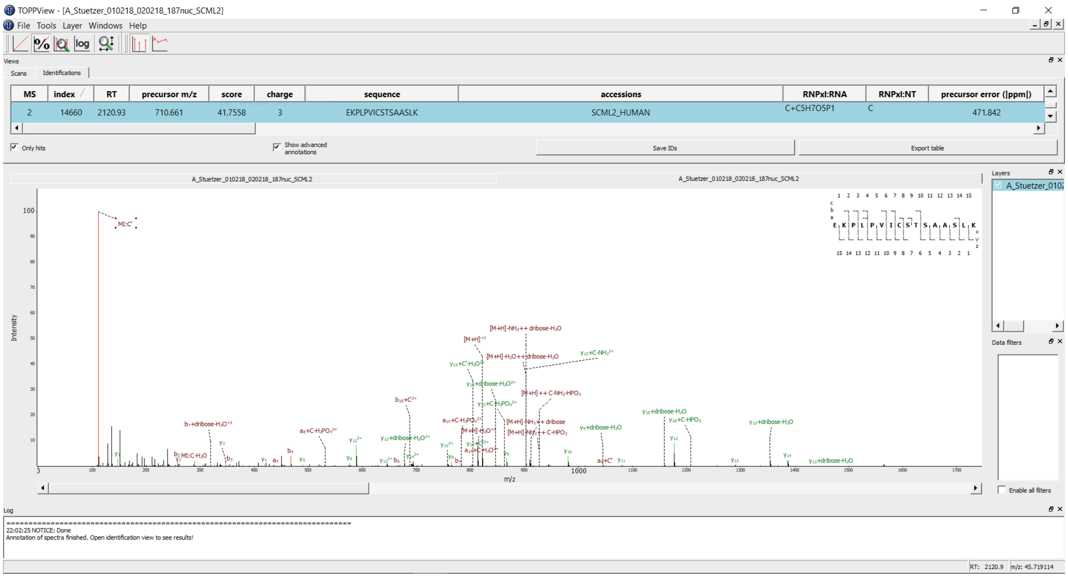

3)

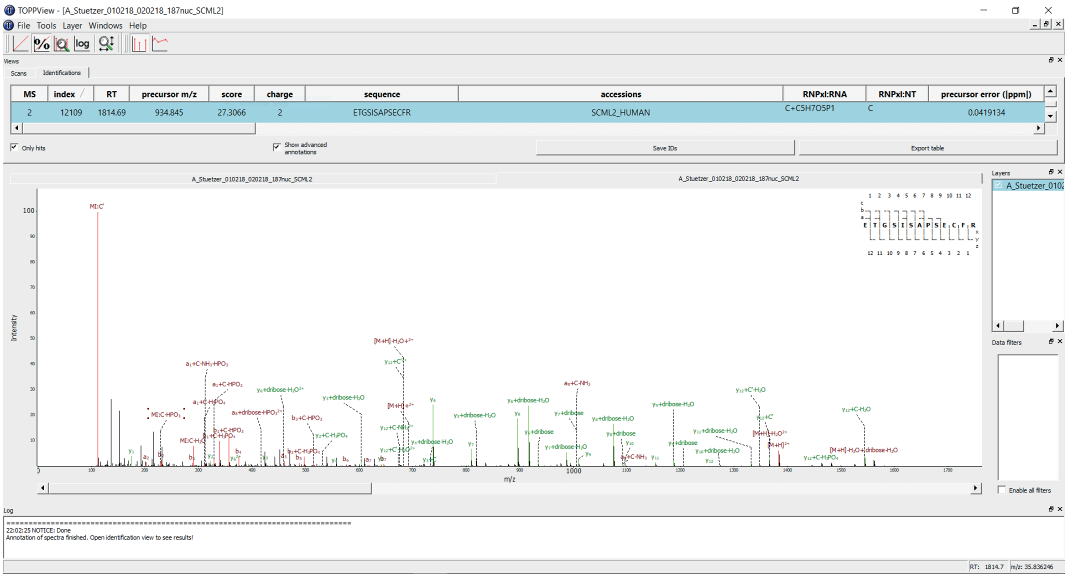

4)

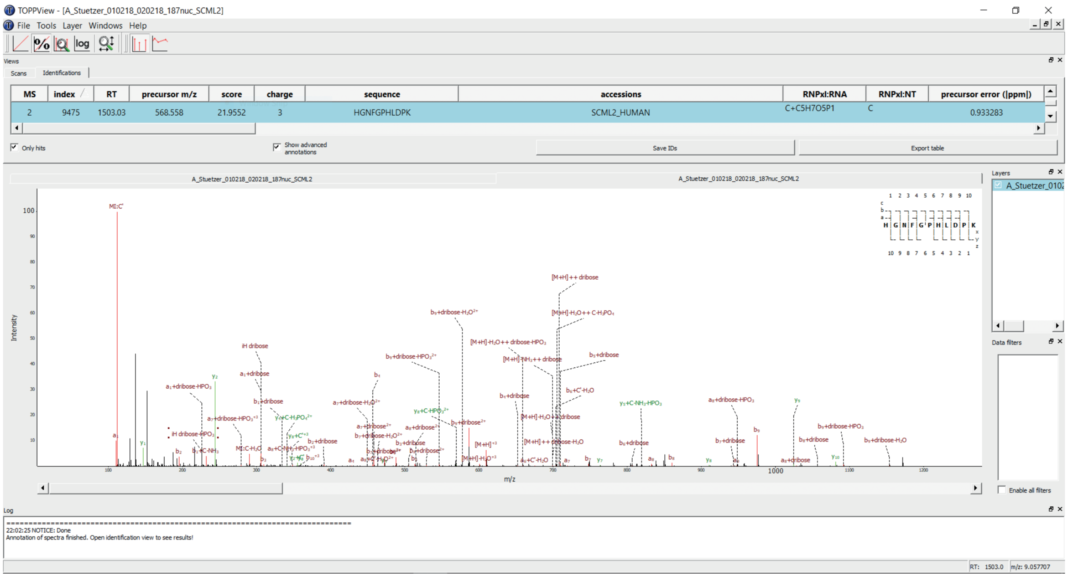
TOPPView - [A_Stuetzer_010218_020218_187nuc_SCML2]
File Tools Layer Windows Help
MS | index | RT | precursor m/z | score | charge | sequence | accessions | RNPxl:RNA | RNPxl:NT | precursor error (|ppm|)
2 | 9475 | 1503.03 | 568.558 | 21.9552 | 3 | HGNFGPHLDPK | SCML2_HUMAN | C+C5H7O5P1 | C | 0.933283
Only hits
Show advanced annotations
Save IDs
Export table
A_Stuetzer_010218_020218_187nuc_SCML2
Intensity
m/z
Log
22:02:25 NOTICE: Done
Annotation of spectra finished. Open identification view to see results!
RT: 1503.0 m/z: 9.057707

5)

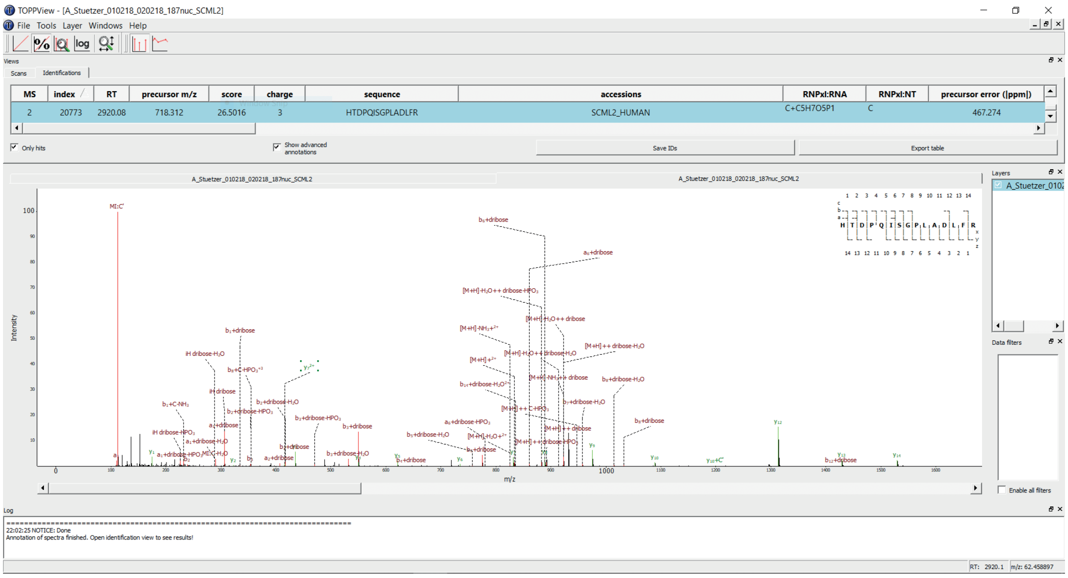
TOPPView - [A_Stuetzer_010218_020218_187nuc_SCML2]
File Tools Layer Windows Help
MS | index | RT | precursor m/z | score | charge | sequence | accessions | RNPxl:RNA | RNPxl:NT | precursor error (|ppm|)
2 | 20773 | 2920.08 | 718.312 | 26.5016 | 3 | HTDPQISGPLADLFR | SCML2_HUMAN | C+C5H7O5P1 | C | 467.274
Only hits
Show advanced annotations
Save IDs
Export table
A_Stuetzer_010218_020218_187nuc_SCML2
Intensity
m/z
Log
22:02:25 NOTICE: Done
Annotation of spectra finished. Open identification view to see results!
RT: 2920.1 m/z: 62.458897

6)

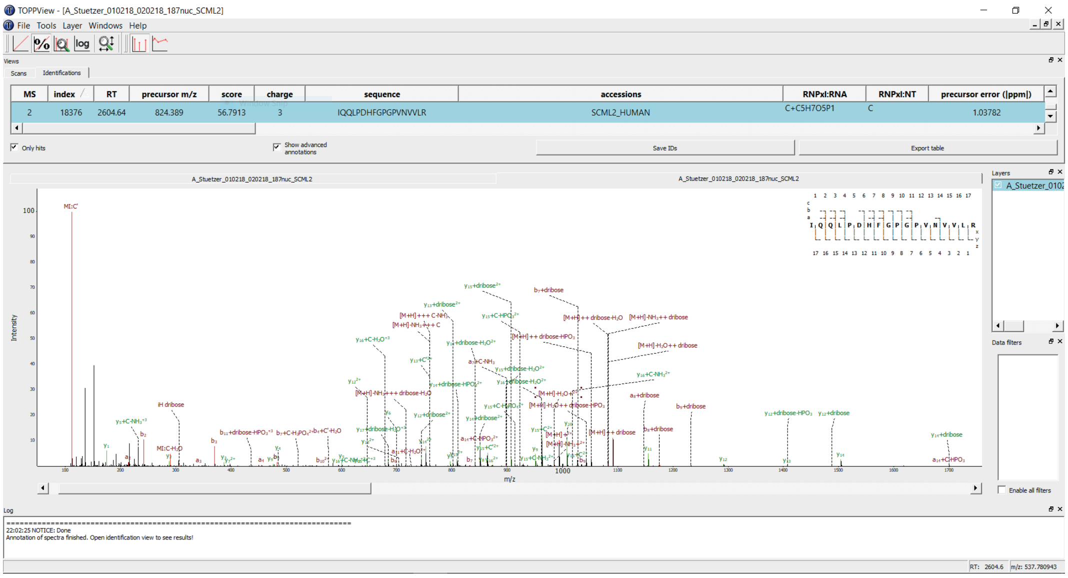
TOPPView - [A_Stuetzer_010218_020218_187nuc_SCML2]
File Tools Layer Windows Help
MS | index | RT | precursor m/z | score | charge | sequence | accessions | RNPxl:RNA | RNPxl:NT | precursor error (|ppm|)
2 | 18376 | 2604.64 | 824.389 | 56.7913 | 3 | IQQLPDHFGPGPVNVVLR | SCML2_HUMAN | C+C5H7O5P1 | C | 1.03782
Only hits
Show advanced annotations
Save IDs
Export table
A_Stuetzer_010218_020218_187nuc_SCML2
Intensity
m/z
Log
22:02:25 NOTICE: Done
Annotation of spectra finished. Open identification view to see results!
RT: 2604.6 m/z: 537.780943

7)

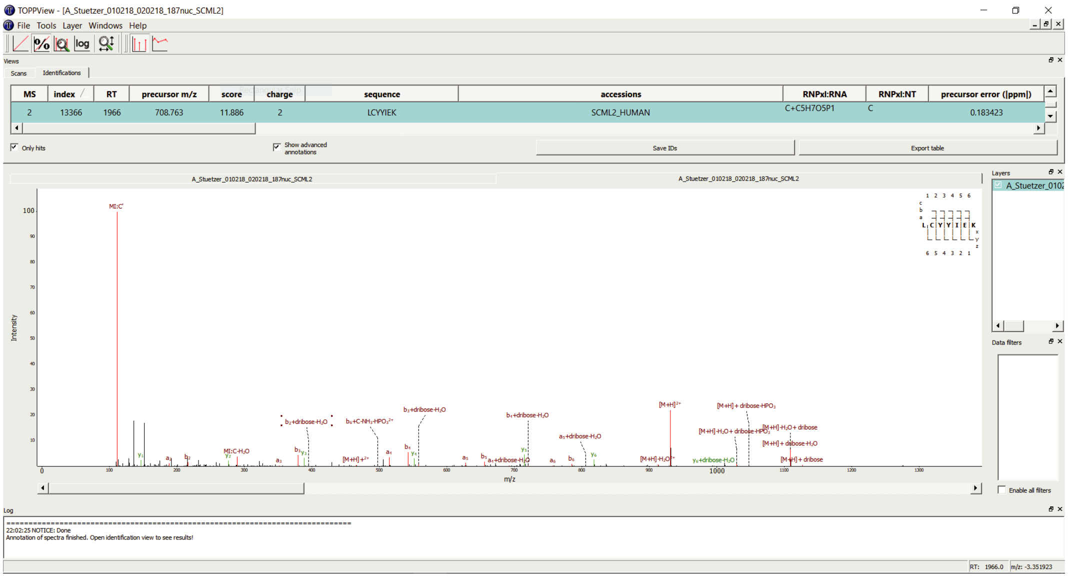
TOPPView - [A_Stuetzer_010218_020218_187nuc_SCML2]
File Tools Layer Windows Help
MS | index | RT | precursor m/z | score | charge | sequence | accessions | RNPxl:RNA | RNPxl:NT | precursor error (|ppm|)
2 | 13366 | 1966 | 708.763 | 11.886 | 2 | LCYYIEK | SCML2_HUMAN | C+C5H7O5P1 | C | 0.183423
Only hits
Show advanced annotations
Save IDs
Export table
Intensity
m/z
22:02:25 NOTICE: Done
Annotation of spectra finished. Open identification view to see results!

8)

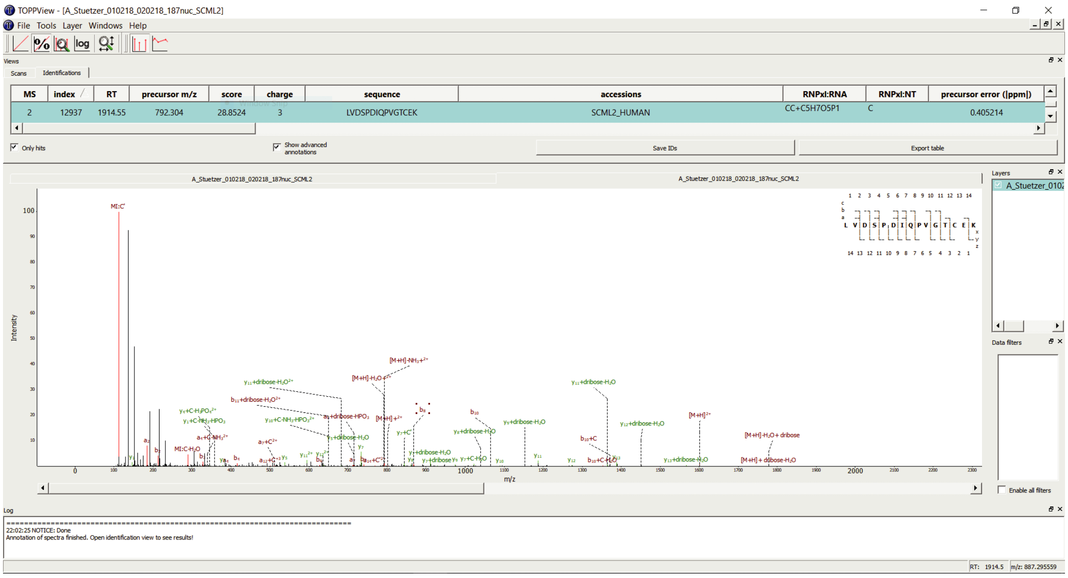
TOPPView - [A_Stuetzer_010218_020218_187nuc_SCML2]
File Tools Layer Windows Help
MS | index | RT | precursor m/z | score | charge | sequence | accessions | RNPxl:RNA | RNPxl:NT | precursor error (|ppm|)
2 | 12937 | 1914.55 | 792.304 | 28.8524 | 3 | LVDSPDIQPVGTCEK | SCML2_HUMAN | CC+C5H7O5P1 | C | 0.405214
Only hits
Show advanced annotations
Save IDs
Export table
Intensity
m/z
22:02:25 NOTICE: Done
Annotation of spectra finished. Open identification view to see results!

9)

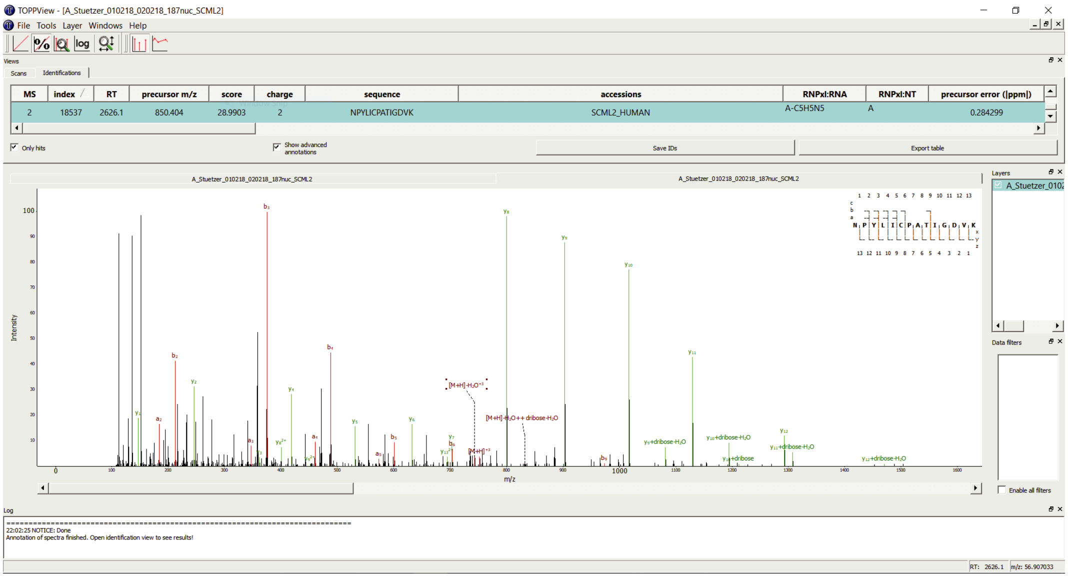
TOPPView - [A_Stuetzer_010218_020218_187nuc_SCML2]
File Tools Layer Windows Help
MS | index | RT | precursor m/z | score | charge | sequence | accessions | RNPxl:RNA | RNPxl:NT | precursor error (|ppm|)
2 | 18537 | 2626.1 | 850.404 | 28.9903 | 2 | NPYLICPATIGDVK | SCML2_HUMAN | A-C5H5N5 | A | 0.284299
Only hits
Show advanced annotations
Save IDs
Export table
Intensity
m/z
22:02:25 NOTICE: Done
Annotation of spectra finished. Open identification view to see results!

10)

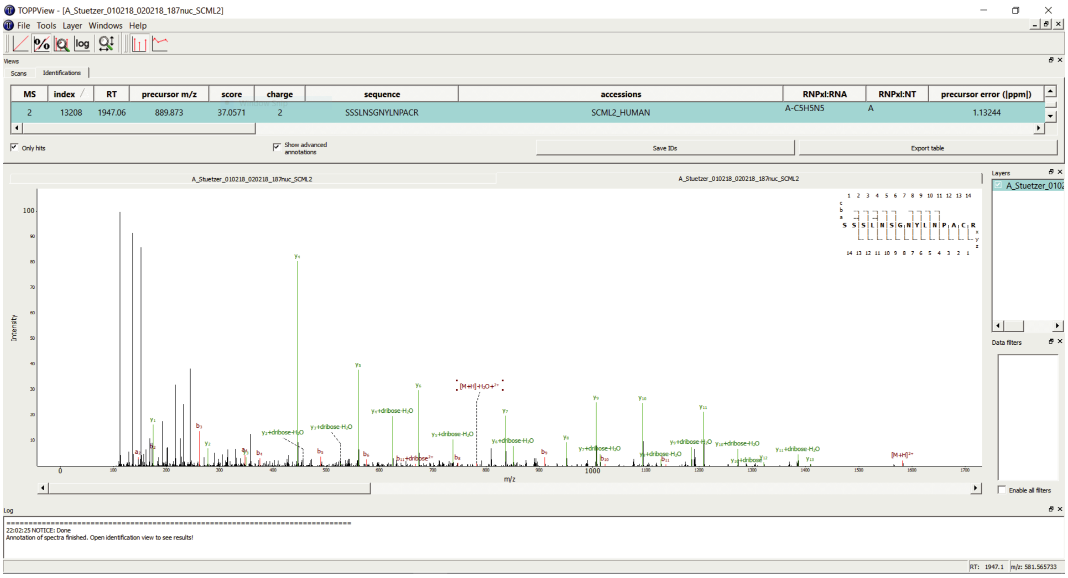

TOPPView spectra of SCML2 CTRL

1)

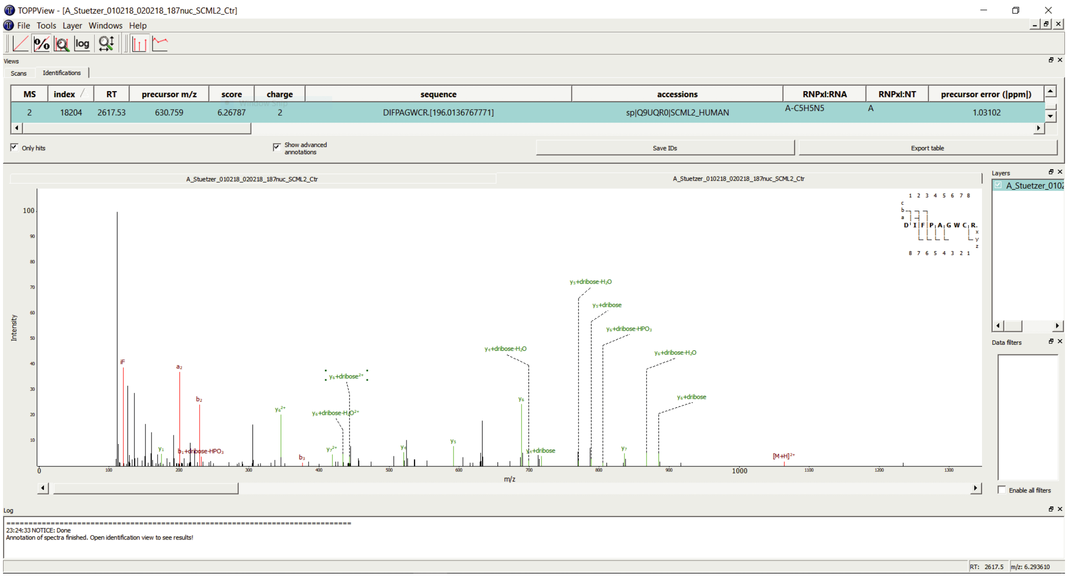

2)

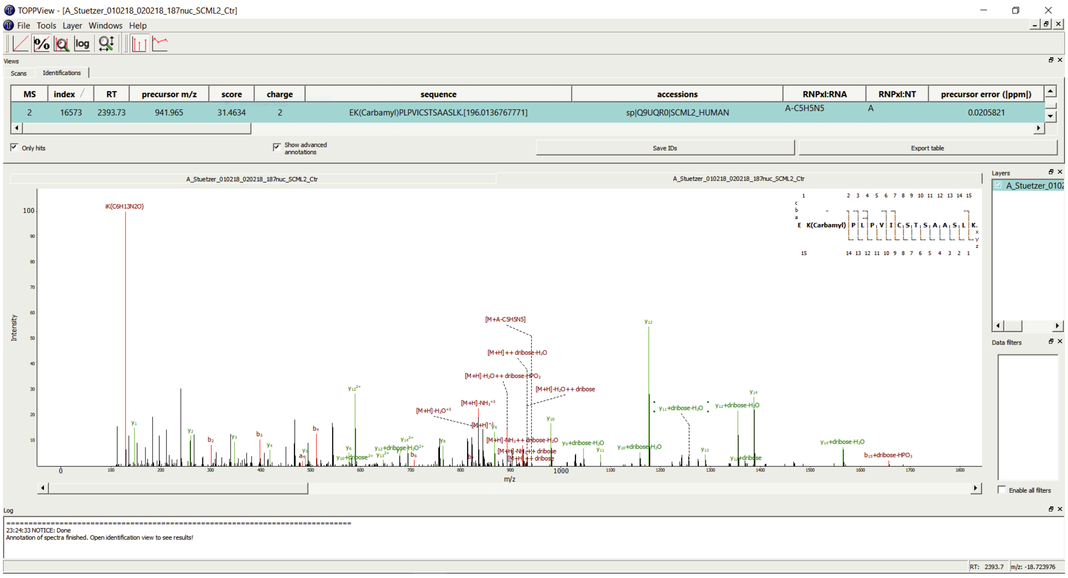

3)

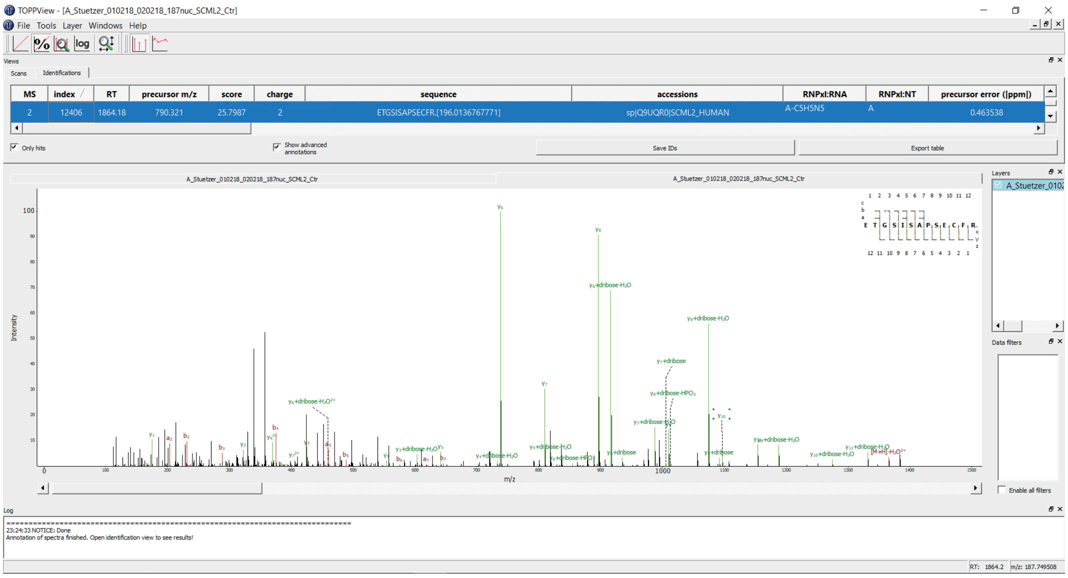
TOPPView - [A_Stuetzer_010218_020218_187nuc_SCML2_Ctr]
MS | index | RT | precursor m/z | score | charge | sequence | accessions | RNPxl:RNA | RNPxl:NT | precursor error (|ppm|)
2 | 12406 | 1864.18 | 790.321 | 25.7987 | 2 | ETGSISAPSECFR.[196.0136767771] | sp|Q9UQR0|SCML2_HUMAN | A-C5H5N5 | A | 0.463538
23:24:33 NOTICE: Done
Annotation of spectra finished. Open identification view to see results!

4)

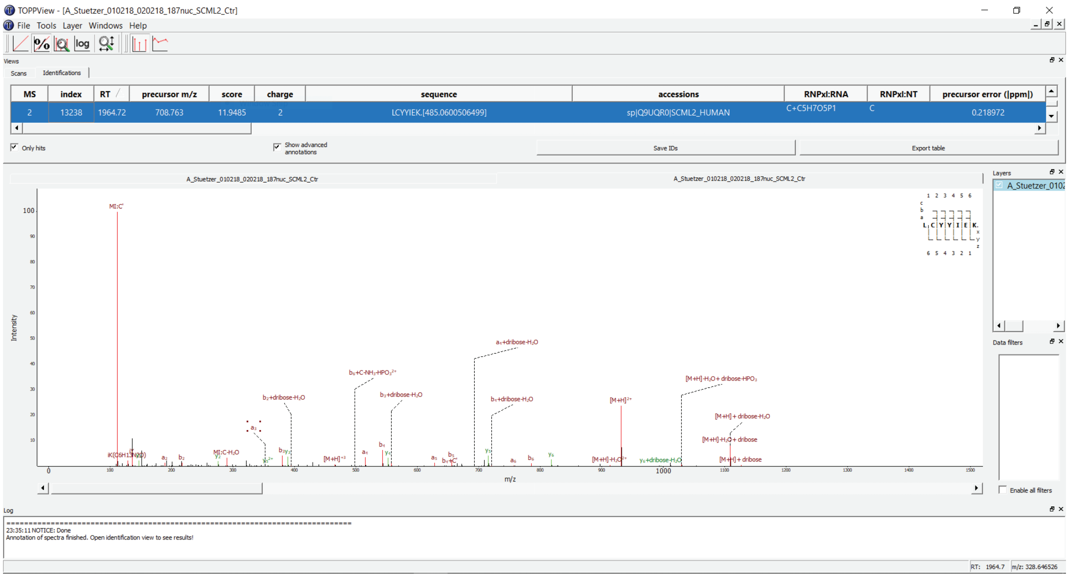
TOPPView - [A_Stuetzer_010218_020218_187nuc_SCML2_Ctr]
MS | index | RT | precursor m/z | score | charge | sequence | accessions | RNPxl:RNA | RNPxl:NT | precursor error (|ppm|)
2 | 13238 | 1964.72 | 708.763 | 11.9485 | 2 | LCYYIEK.[485.0600506499] | sp|Q9UQR0|SCML2_HUMAN | C+C5H7O5P1 | C | 0.218972
23:35:11 NOTICE: Done
Annotation of spectra finished. Open identification view to see results!

5)

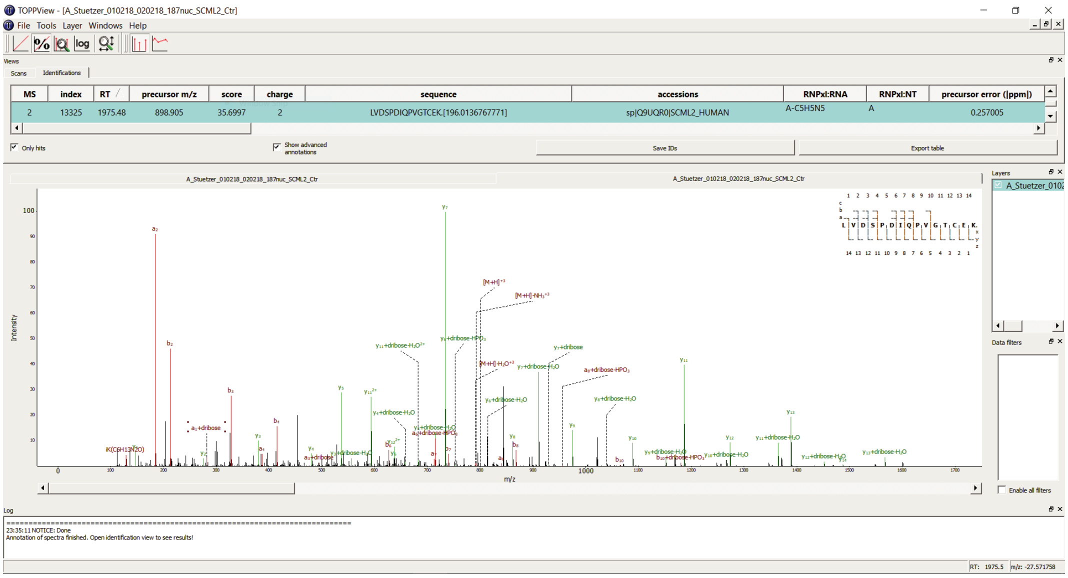
TOPPView - [A_Stuetzer_010218_020218_187nuc_SCML2_Ctr]
MS | index | RT | precursor m/z | score | charge | sequence | accessions | RNPxl:RNA | RNPxl:NT | precursor error (|ppm|)
2 | 13325 | 1975.48 | 898.905 | 35.6997 | 2 | LVDSPDIQPVGTCEK.[196.0136767771] | sp|Q9UQR0|SCML2_HUMAN | A-C5H5N5 | A | 0.257005
23:35:11 NOTICE: Done
Annotation of spectra finished. Open identification view to see results!

6)

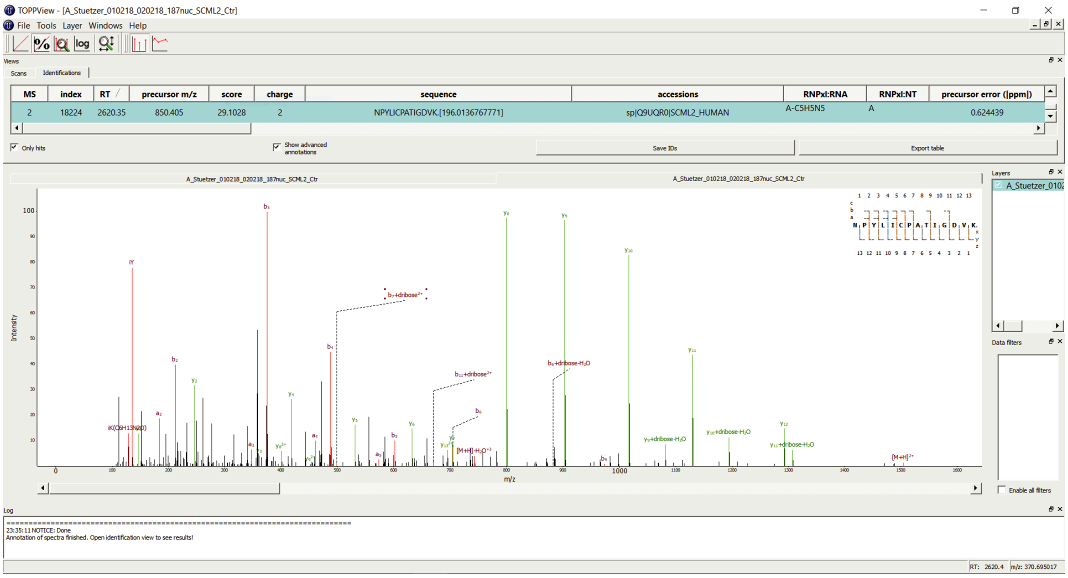

TOPPView spectra of SCML2 UV XL + H1.4

1)

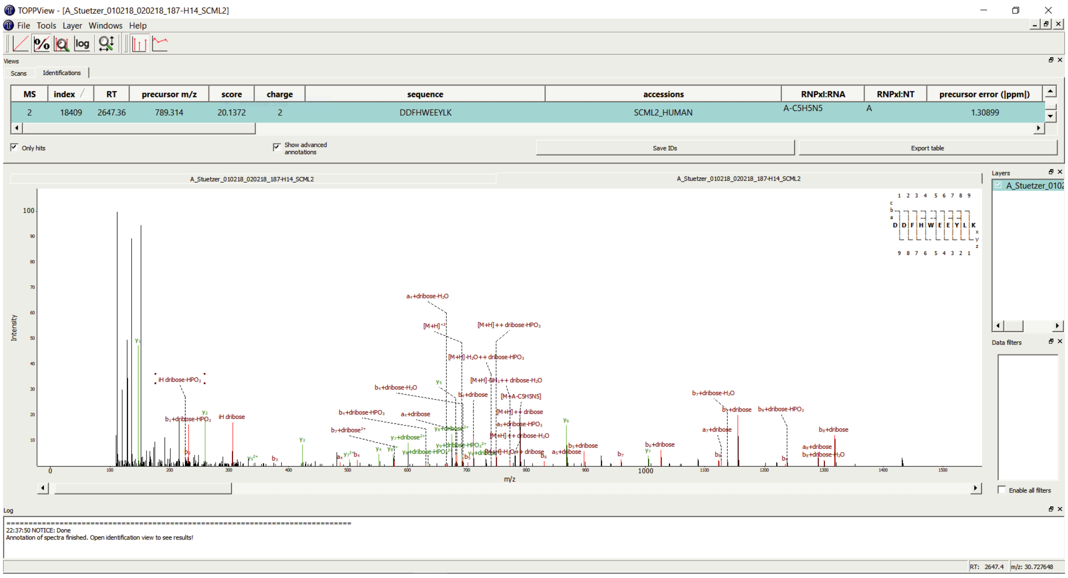

2)

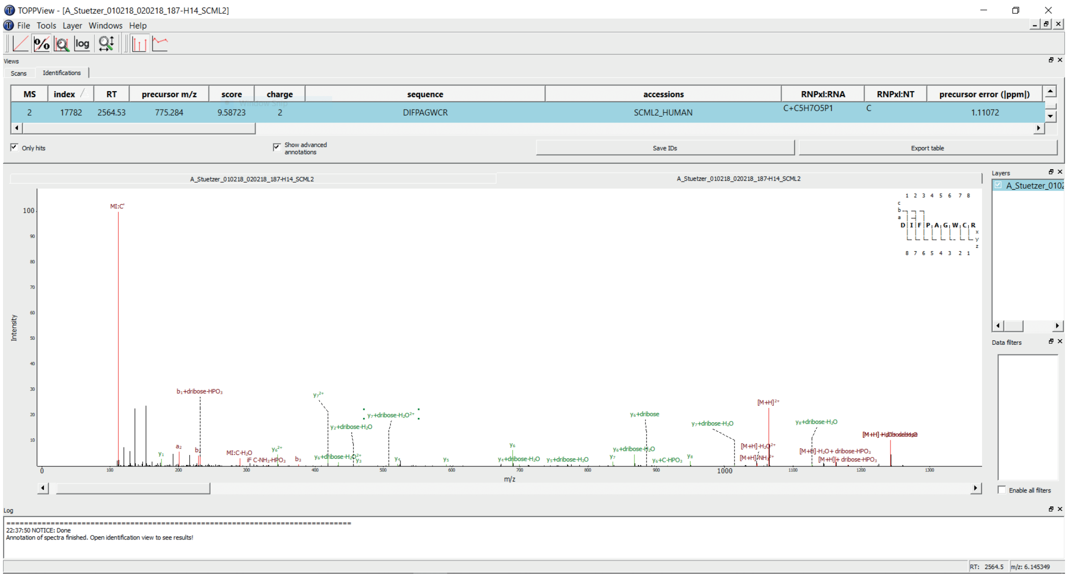
TOPPView - [A_Stuetzer_010218_020218_187-H14_SCML2]
File Tools Layer Windows Help
MS | index | RT | precursor m/z | score | charge | sequence | accessions | RNPxl:RNA | RNPxl:NT | precursor error (|ppm|)
2 | 17782 | 2564.53 | 775.284 | 9.58723 | 2 | DIFPAGWCR | SCML2_HUMAN | C+C5H7O5P1 | C | 1.11072
Only hits
Show advanced annotations
Save IDs
Export table
A_Stuetzer_010218_020218_187-H14_SCML2
Intensity
m/z
Layers
Data filters
Enable all filters
Log
22:37:50 NOTICE: Done
Annotation of spectra finished. Open identification view to see results!
RT: 2564.5
m/z: 6.145349

3)

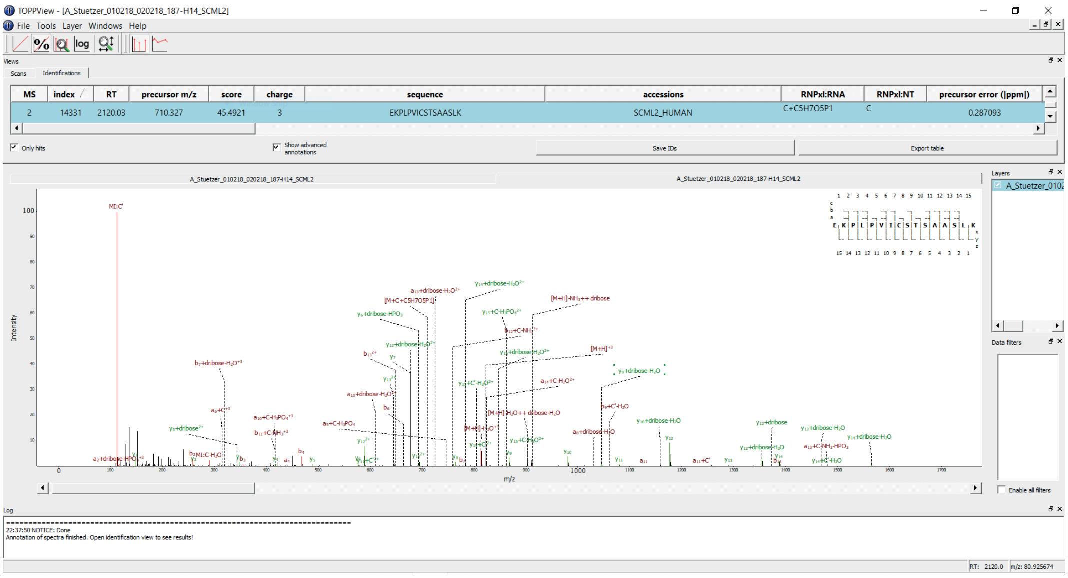
TOPPView - [A_Stuetzer_010218_020218_187-H14_SCML2]
File Tools Layer Windows Help
MS | index | RT | precursor m/z | score | charge | sequence | accessions | RNPxl:RNA | RNPxl:NT | precursor error (|ppm|)
2 | 14331 | 2120.03 | 710.327 | 45.4921 | 3 | EKPLPVICSTSAASLK | SCML2_HUMAN | C+C5H7O5P1 | C | 0.287093
Only hits
Show advanced annotations
Save IDs
Export table
A_Stuetzer_010218_020218_187-H14_SCML2
Intensity
m/z
Layers
Data filters
Enable all filters
Log
22:37:50 NOTICE: Done
Annotation of spectra finished. Open identification view to see results!
RT: 2120.0
m/z: 80.925674

4)

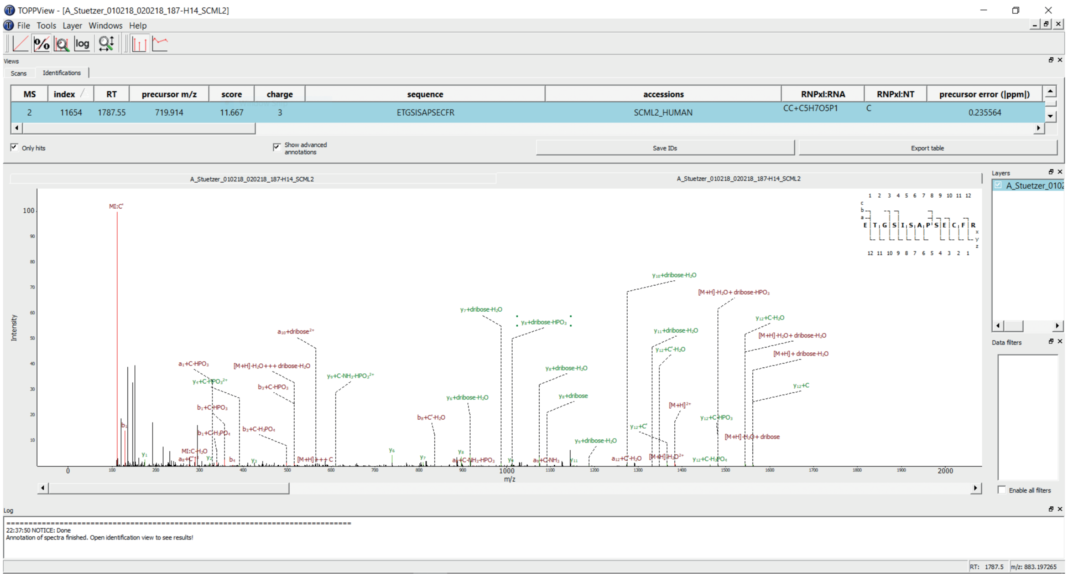
TOPPView - [A_Stuetzer_010218_020218_187-H14_SCML2]
File Tools Layer Windows Help
MS | index | RT | precursor m/z | score | charge | sequence | accessions | RNPxl:RNA | RNPxl:NT | precursor error (|ppm|)
2 | 11654 | 1787.55 | 719.914 | 11.667 | 3 | ETGSISAPSECFR | SCML2_HUMAN | CC+C5H7O5P1 | C | 0.235564
Only hits
Show advanced annotations
Save IDs
Export table
A_Stuetzer_010218_020218_187-H14_SCML2
Intensity
m/z
Layers
Data filters
Enable all filters
Log
22:37:50 NOTICE: Done
Annotation of spectra finished. Open identification view to see results!
RT: 1787.5
m/z: 883.197265

5)

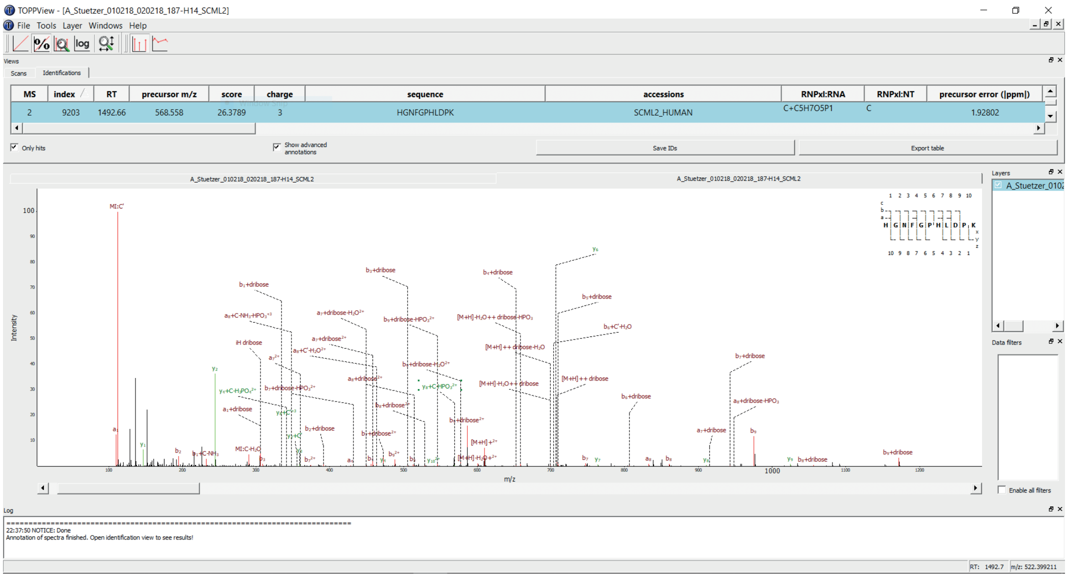
TOPPView - [A_Stuetzer_010218_020218_187-H14_SCML2]
File Tools Layer Windows Help
MS | index | RT | precursor m/z | score | charge | sequence | accessions | RNPxl:RNA | RNPxl:NT | precursor error (|ppm|)
2 | 9203 | 1492.66 | 568.558 | 26.3789 | 3 | HGNFGPHLDPK | SCML2_HUMAN | C+C5H7O5P1 | C | 1.92802
Only hits
Show advanced annotations
Save IDs
Export table
A_Stuetzer_010218_020218_187-H14_SCML2
Intensity
m/z
22:37:50 NOTICE: Done
Annotation of spectra finished. Open identification view to see results!
RT: 1492.7 m/z: 522.399211

6)

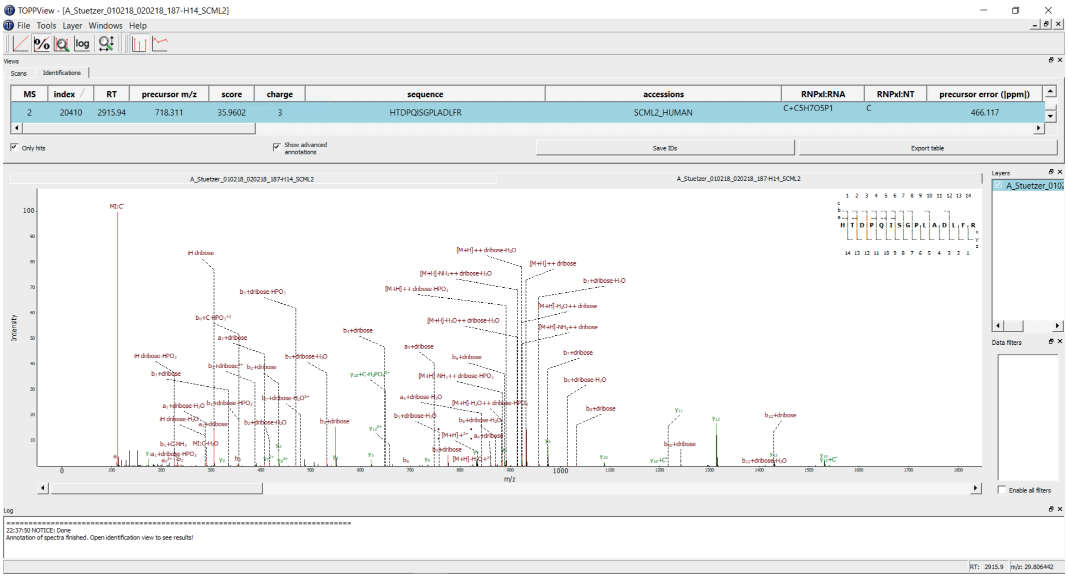
TOPPView - [A_Stuetzer_010218_020218_187-H14_SCML2]
File Tools Layer Windows Help
MS | index | RT | precursor m/z | score | charge | sequence | accessions | RNPxl:RNA | RNPxl:NT | precursor error (|ppm|)
2 | 20410 | 2915.94 | 718.311 | 35.9602 | 3 | HTDPQISGPLADLFR | SCML2_HUMAN | C+C5H7O5P1 | C | 466.117
Only hits
Show advanced annotations
Save IDs
Export table
A_Stuetzer_010218_020218_187-H14_SCML2
Intensity
m/z
22:37:50 NOTICE: Done
Annotation of spectra finished. Open identification view to see results!
RT: 2915.9 m/z: 29.806442

7)

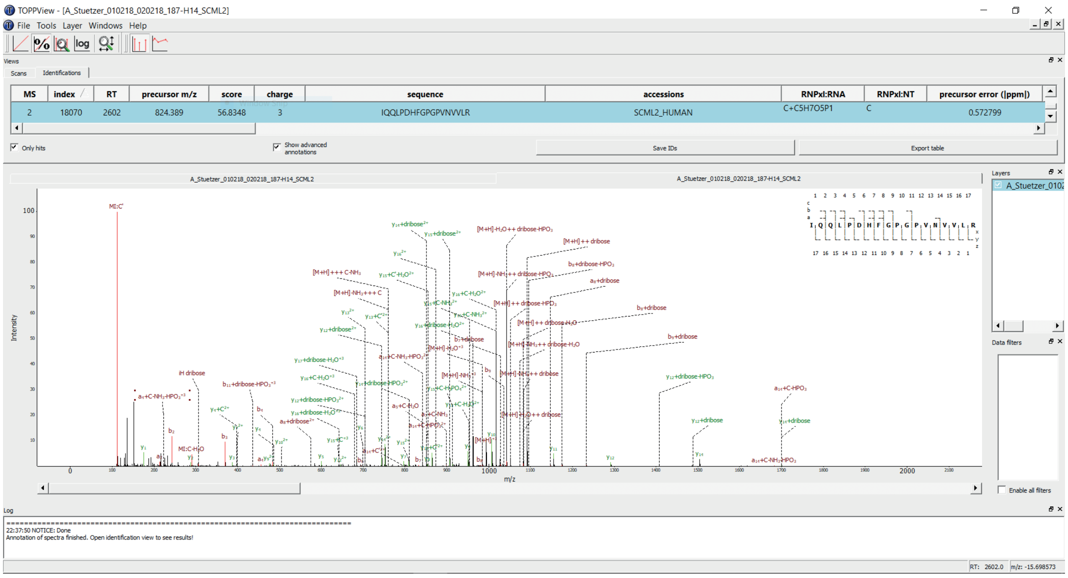
TOPPView - [A_Stuetzer_010218_020218_187-H14_SCML2]
File Tools Layer Windows Help
MS | index | RT | precursor m/z | score | charge | sequence | accessions | RNPxl:RNA | RNPxl:NT | precursor error (|ppm|)
2 | 18070 | 2602 | 824.389 | 56.8348 | 3 | IQQLPDHFGPGPVNVVLR | SCML2_HUMAN | C+C5H7O5P1 | C | 0.572799
Only hits
Show advanced annotations
Save IDs
Export table
A_Stuetzer_010218_020218_187-H14_SCML2
Intensity
m/z
22:37:50 NOTICE: Done
Annotation of spectra finished. Open identification view to see results!
RT: 2602.0 m/z: -15.698573

8)

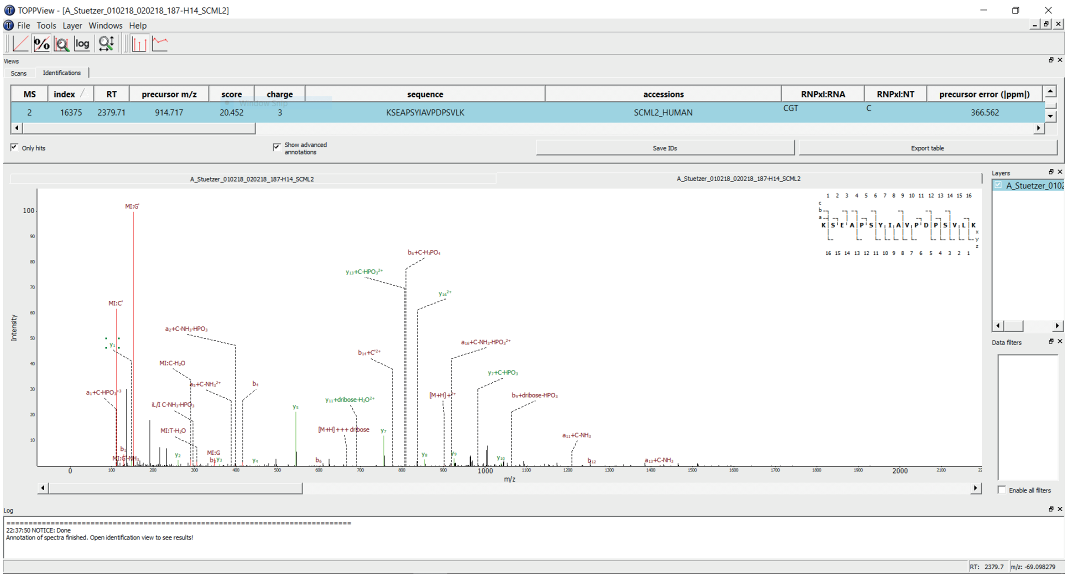
TOPPView - [A_Stuetzer_010218_020218_187-H14_SCML2]
File Tools Layer Windows Help
MS index RT precursor m/z score charge sequence accessions RNPxl:RNA RNPxl:NT precursor error (|ppm|)
2 16375 2379.71 914.717 20.452 3 KSEAPSYIAVPDPSVLK SCML2_HUMAN CGT C 366.562
Only hits
Show advanced annotations
Save IDs
Export table
A_Stuetzer_010218_020218_187-H14_SCML2
Intensity
m/z
Layers
Data filters
Enable all filters
Log
22:37:50 NOTICE: Done
Annotation of spectra finished. Open identification view to see results!
RT: 2379.7
m/z: -69.098279

9)

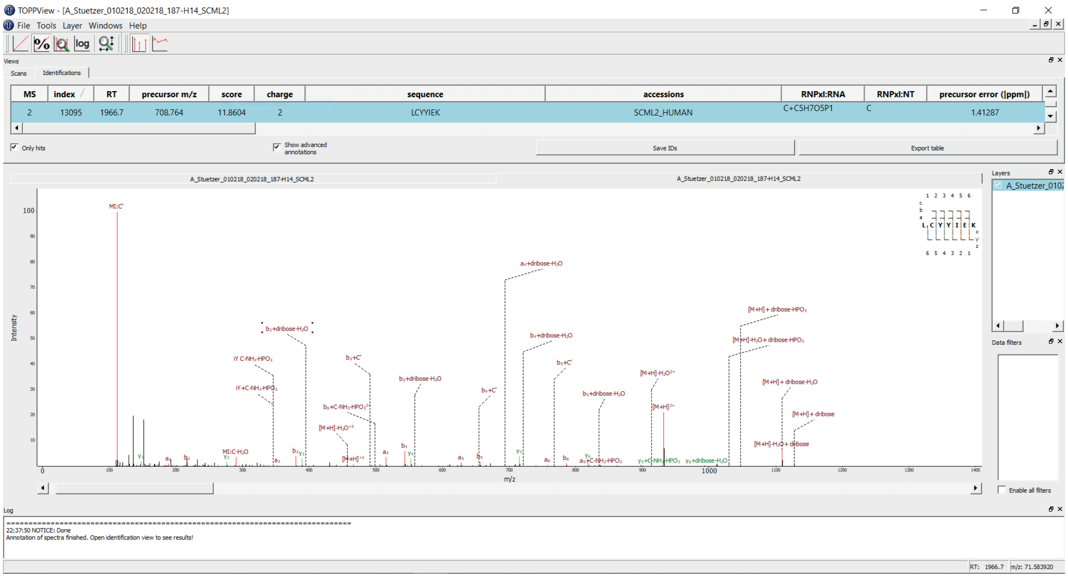
TOPPView - [A_Stuetzer_010218_020218_187-H14_SCML2]
File Tools Layer Windows Help
MS index RT precursor m/z score charge sequence accessions RNPxl:RNA RNPxl:NT precursor error (|ppm|)
2 13095 1966.7 708.764 11.8604 2 LCYYIEK SCML2_HUMAN C+C5H7O5P1 C 1.41287
Only hits
Show advanced annotations
Save IDs
Export table
A_Stuetzer_010218_020218_187-H14_SCML2
Intensity
m/z
Layers
Data filters
Enable all filters
Log
22:37:50 NOTICE: Done
Annotation of spectra finished. Open identification view to see results!
RT: 1966.7
m/z: 71.583920

10)

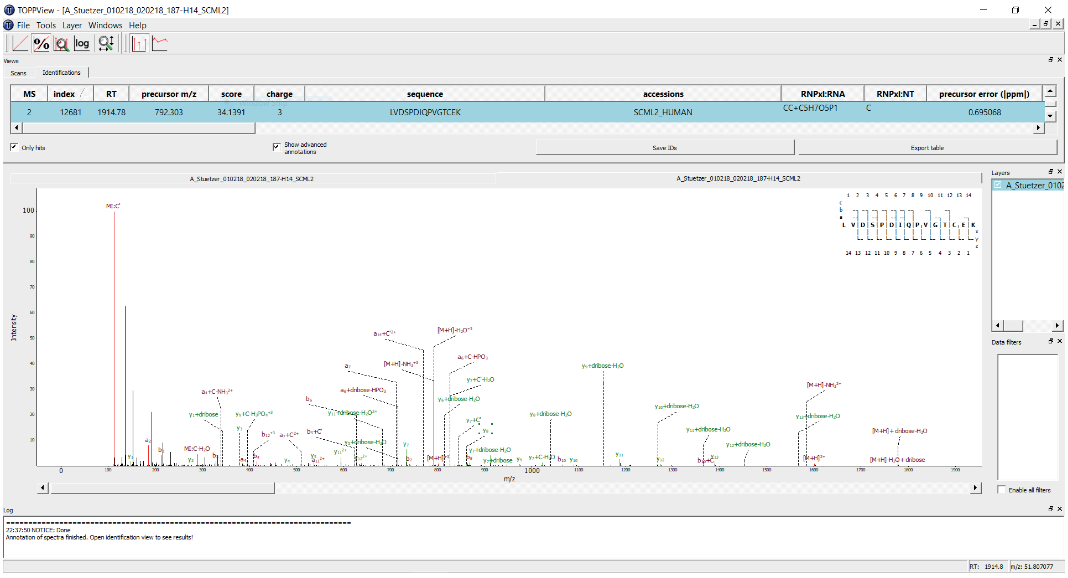
TOPPView - [A_Stuetzer_010218_020218_187-H14_SCML2]
File Tools Layer Windows Help
MS index RT precursor m/z score charge sequence accessions RNPxl:RNA RNPxl:NT precursor error (|ppm|)
2 12681 1914.78 792.303 34.1391 3 LVDSPDIQPVGTCEK SCML2_HUMAN CC+C5H7O5P1 C 0.695068
Only hits
Show advanced annotations
Save IDs
Export table
A_Stuetzer_010218_020218_187-H14_SCML2
Intensity
m/z
Layers
Data filters
Enable all filters
Log
22:37:50 NOTICE: Done
Annotation of spectra finished. Open identification view to see results!
RT: 1914.8
m/z: 51.807077

11)

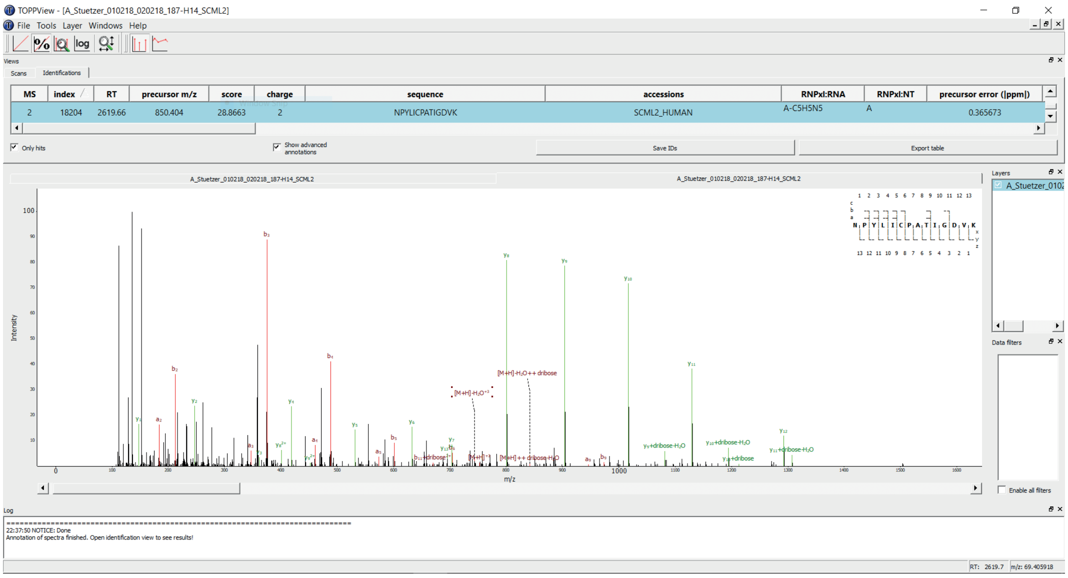
TOPPView - [A_Stuetzer_010218_020218_187-H14_SCML2]
File Tools Layer Windows Help
MS | index | RT | precursor m/z | score | charge | sequence | accessions | RNPxl:RNA | RNPxl:NT | precursor error (|ppm|)
2 | 18204 | 2619.66 | 850.404 | 28.8663 | 2 | NPYLICPATIGDVK | SCML2_HUMAN | A-C5H5N5 | A | 0.365673
Only hits
Show advanced annotations
Save IDs
Export table
A_Stuetzer_010218_020218_187-H14_SCML2
Intensity
m/z
22:37:50 NOTICE: Done
Annotation of spectra finished. Open identification view to see results!
RT: 2619.7
m/z: 69.405918

12)

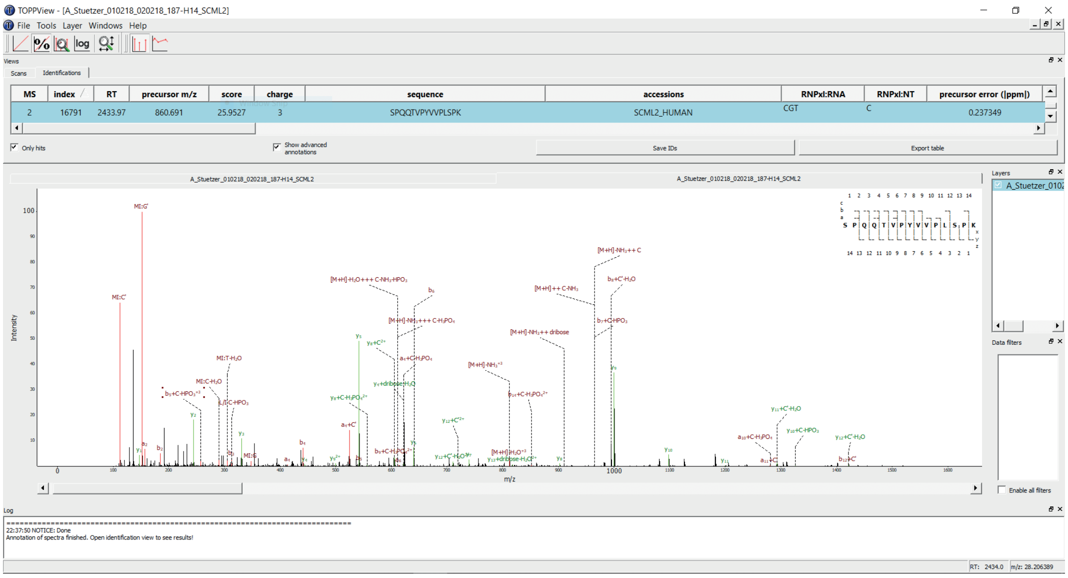
TOPPView - [A_Stuetzer_010218_020218_187-H14_SCML2]
File Tools Layer Windows Help
MS | index | RT | precursor m/z | score | charge | sequence | accessions | RNPxl:RNA | RNPxl:NT | precursor error (|ppm|)
2 | 16791 | 2433.97 | 860.691 | 25.9527 | 3 | SPQQTVPYVVPLSPK | SCML2_HUMAN | CGT | C | 0.237349
Only hits
Show advanced annotations
Save IDs
Export table
A_Stuetzer_010218_020218_187-H14_SCML2
Intensity
m/z
22:37:50 NOTICE: Done
Annotation of spectra finished. Open identification view to see results!
RT: 2434.0
m/z: 28.206389

13)

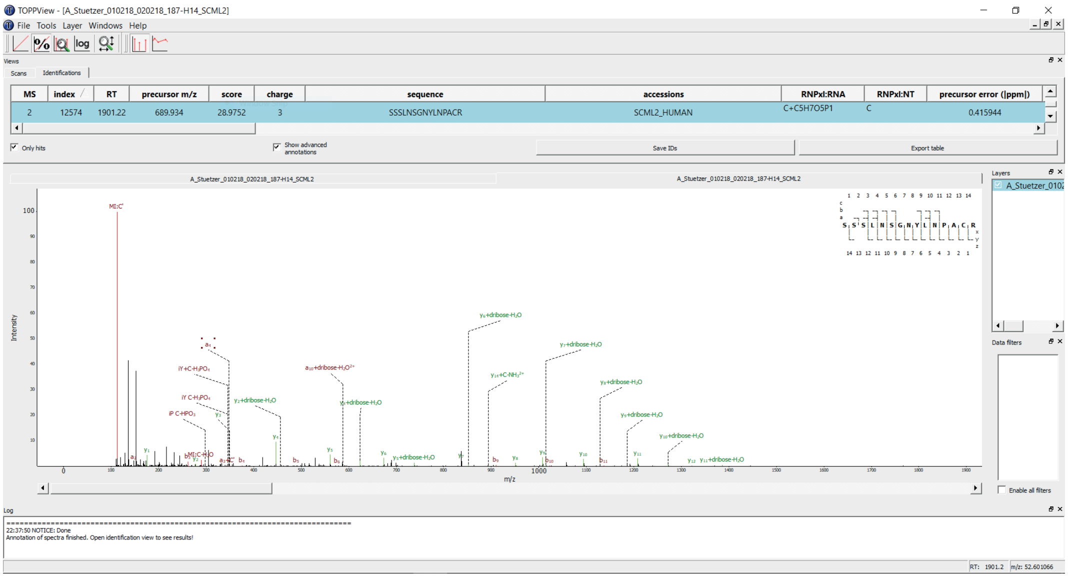
TOPPView - [A_Stuetzer_010218_020218_187-H14_SCML2]
File Tools Layer Windows Help
MS | index | RT | precursor m/z | score | charge | sequence | accessions | RNPxl:RNA | RNPxl:NT | precursor error (|ppm|)
2 | 12574 | 1901.22 | 689.934 | 28.9752 | 3 | SSSLNSGNYLNPACR | SCML2_HUMAN | C+C5H7O5P1 | C | 0.415944
Only hits
Show advanced annotations
Save IDs
Export table
A_Stuetzer_010218_020218_187-H14_SCML2
Intensity
m/z
22:37:50 NOTICE: Done
Annotation of spectra finished. Open identification view to see results!
RT: 1901.2
m/z: 52.601066

14)

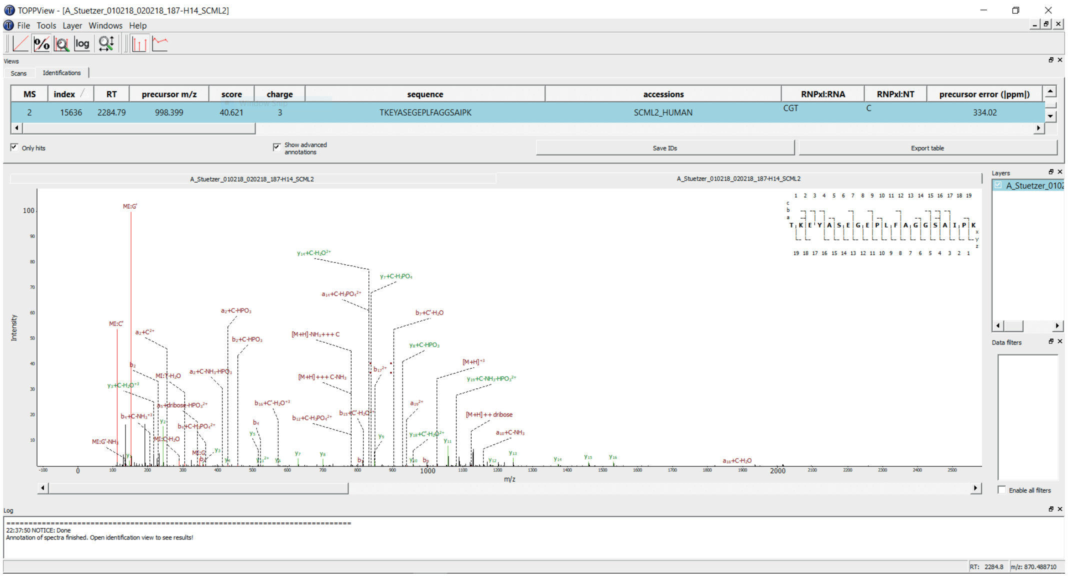

15)

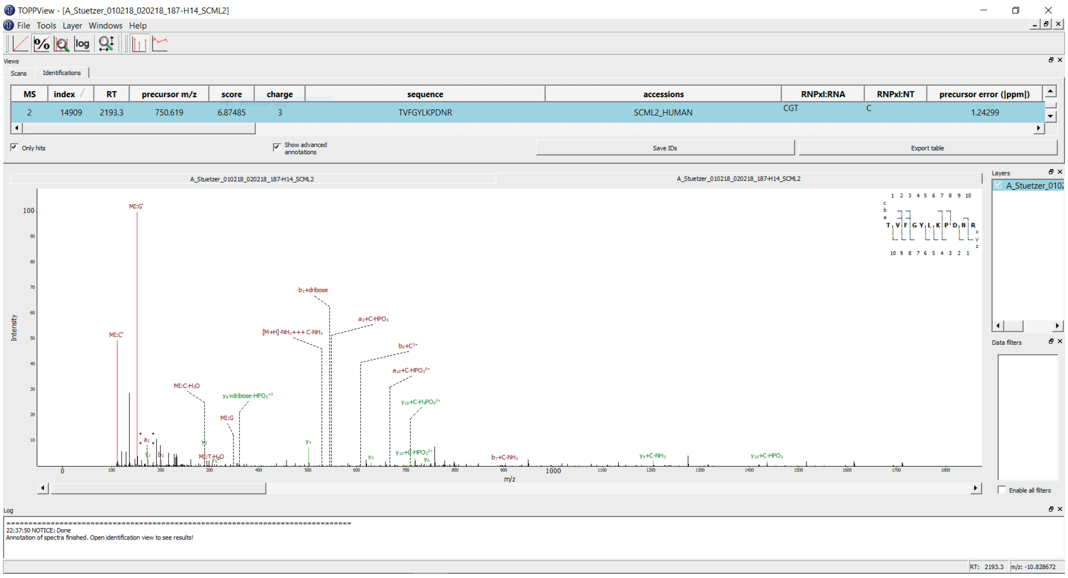

TOPPView spectra of SCML2 CTRL + H1.4

1)

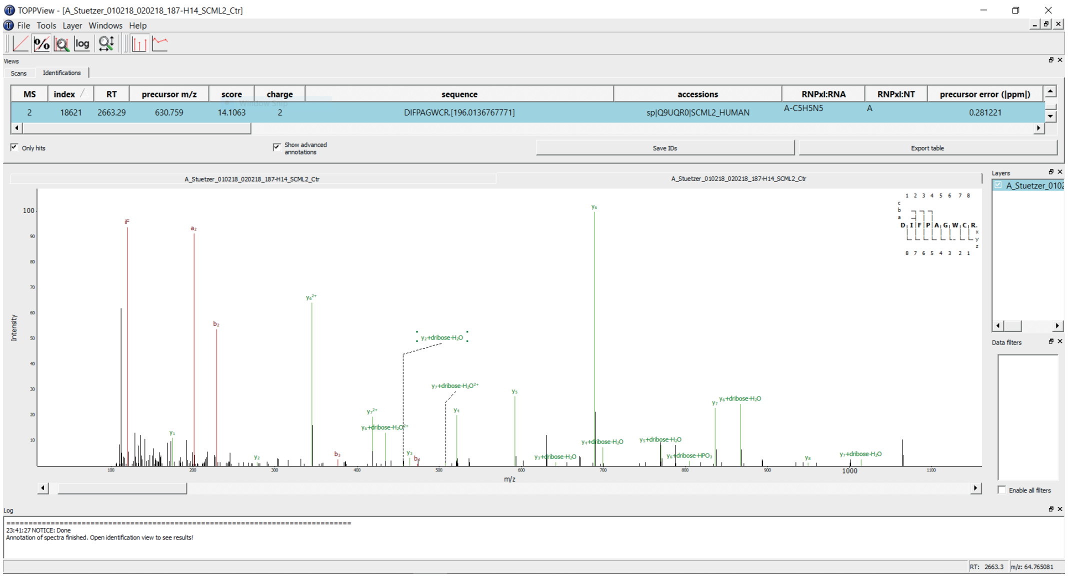

2)

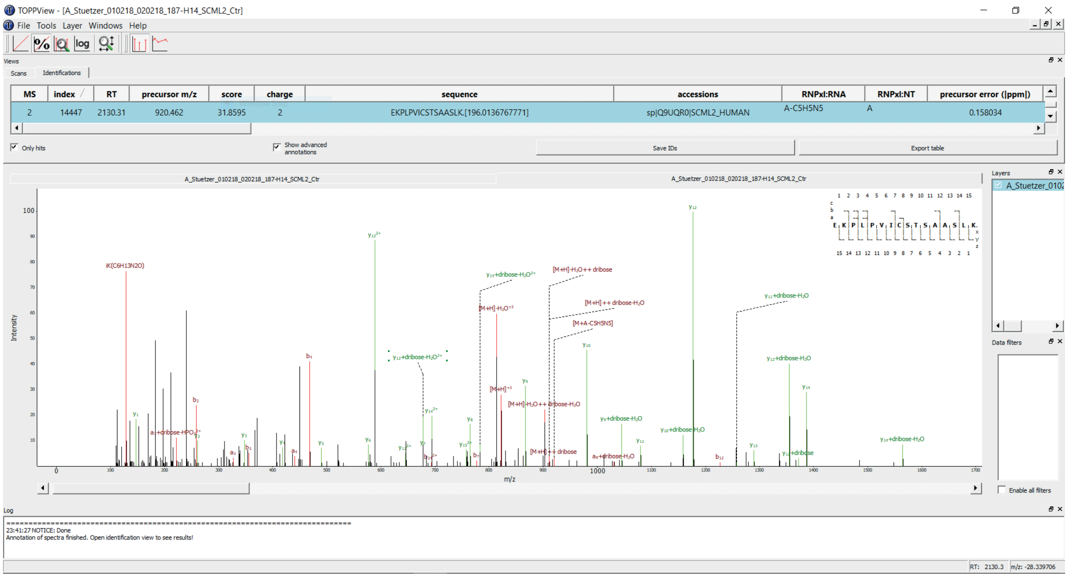
TOPPView - [A_Stuetzer_010218_020218_187-H14_SCML2_Ctr]
File Tools Layer Windows Help
MS | index | RT | precursor m/z | score | charge | sequence | accessions | RNPxl:RNA | RNPxl:NT | precursor error (|ppm|)
2 | 14447 | 2130.31 | 920.462 | 31.8595 | 2 | EKPLPVICSTSAASLK.[196.0136767771] | sp|Q9UQR0|SCML2_HUMAN | A-C5H5N5 | A | 0.158034
Only hits
Show advanced annotations
Save IDs
Export table
A_Stuetzer_010218_020218_187-H14_SCML2_Ctr
Intensity
m/z
Layers
Data filters
Enable all filters
Log
23:41:27 NOTICE: Done
Annotation of spectra finished. Open identification view to see results!

3)

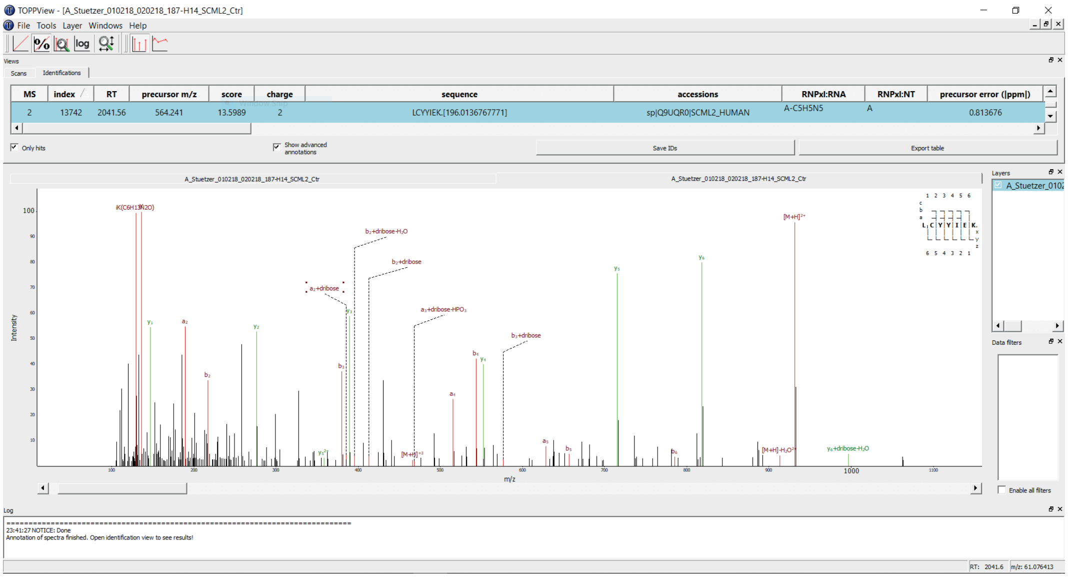
TOPPView - [A_Stuetzer_010218_020218_187-H14_SCML2_Ctr]
File Tools Layer Windows Help
MS | index | RT | precursor m/z | score | charge | sequence | accessions | RNPxl:RNA | RNPxl:NT | precursor error (|ppm|)
2 | 13742 | 2041.56 | 564.241 | 13.5989 | 2 | LCYYIEK.[196.0136767771] | sp|Q9UQR0|SCML2_HUMAN | A-C5H5N5 | A | 0.813676
Only hits
Show advanced annotations
Save IDs
Export table
A_Stuetzer_010218_020218_187-H14_SCML2_Ctr
Intensity
m/z
Layers
Data filters
Enable all filters
Log
23:41:27 NOTICE: Done
Annotation of spectra finished. Open identification view to see results!

4)

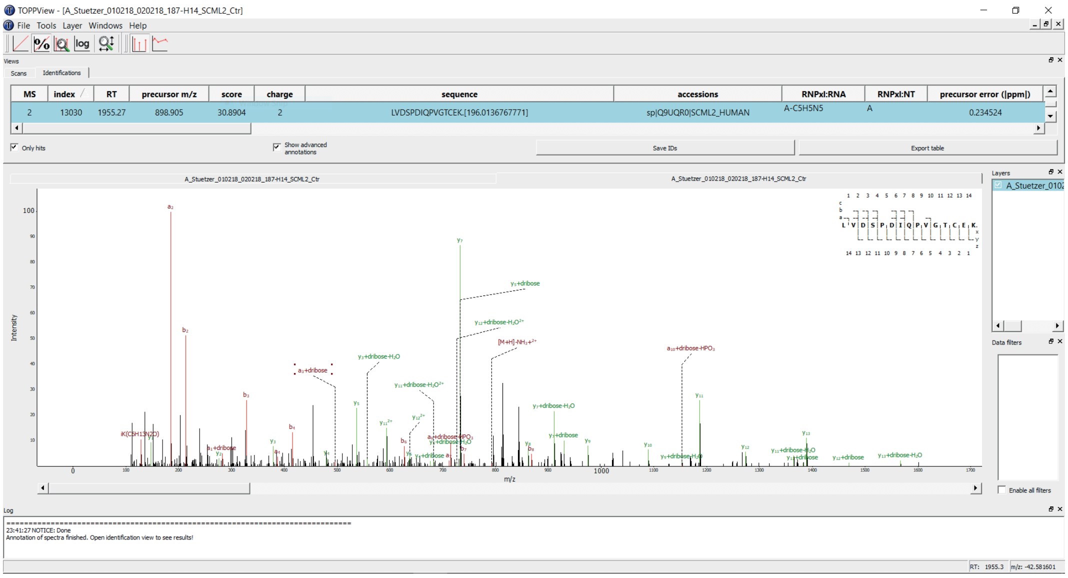
TOPPView - [A_Stuetzer_010218_020218_187-H14_SCML2_Ctr]
File Tools Layer Windows Help
MS | index | RT | precursor m/z | score | charge | sequence | accessions | RNPxl:RNA | RNPxl:NT | precursor error (|ppm|)
2 | 13030 | 1955.27 | 898.905 | 30.8904 | 2 | LVDSPDIQPVGTCEK.[196.0136767771] | sp|Q9UQR0|SCML2_HUMAN | A-C5H5N5 | A | 0.234524
Only hits
Show advanced annotations
Save IDs
Export table
A_Stuetzer_010218_020218_187-H14_SCML2_Ctr
Intensity
m/z
Layers
Data filters
Enable all filters
Log
23:41:27 NOTICE: Done
Annotation of spectra finished. Open identification view to see results!

5)

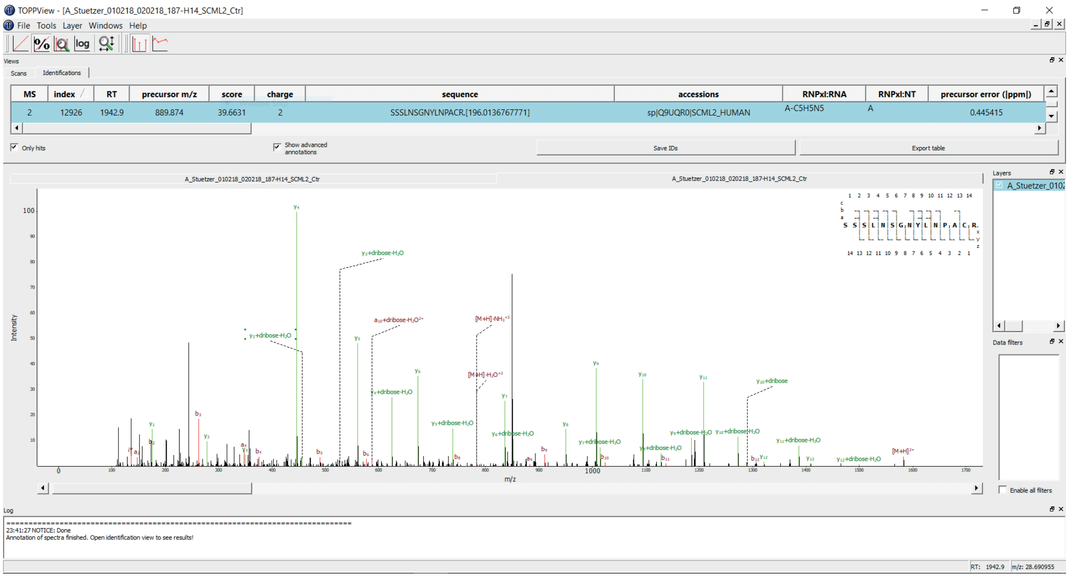
TOPPView - [A_Stuetzer_010218_020218_187-H14_SCML2_Ctr]
File Tools Layer Windows Help
Views
Scans
Identifications
MS
index
RT
precursor m/z
score
charge
sequence
accessions
RNPxl:RNA
RNPxl:NT
precursor error (|ppm|)
2
12926
1942.9
889.874
39.6631
2
SSSLNSGNYLNPACR.[196.0136767771]
sp|Q9UQR0|SCML2_HUMAN
A-C5H5N5
A
0.445415
Only hits
Show advanced annotations
Save IDs
Export table
A_Stuetzer_010218_020218_187-H14_SCML2_Ctr
Intensity
m/z
Layers
Data filters
Enable all filters
Log
23:41:27 NOTICE: Done
Annotation of spectra finished. Open identification view to see results!
RT: 1942.9
m/z: 28.690955
