# Supplementary material for: Analysis of protein-DNA interactions in chromatin by UV induced cross-linking and mass spectrometry
Source: Nat Commun. 2020 Oct 16;11:5250. doi: 10.1038/s41467-020-19047-7 (PMC7567871; doi:10.1038/s41467-020-19047-7)

*Supplementary Data 6*

# Analysis of protein-DNA interactions in chromatin by UV induced cross-linking and mass spectrometry

Stützer *et al.*

## List of contents

TOPPView MS/MS spectra of cross-linked proteins
in HeLa mononucleosomes p. 1-22

# TOPPView spectra of cross-linked proteins in HeLa mononucleosomes

## 1) Y-box-binding protein 3

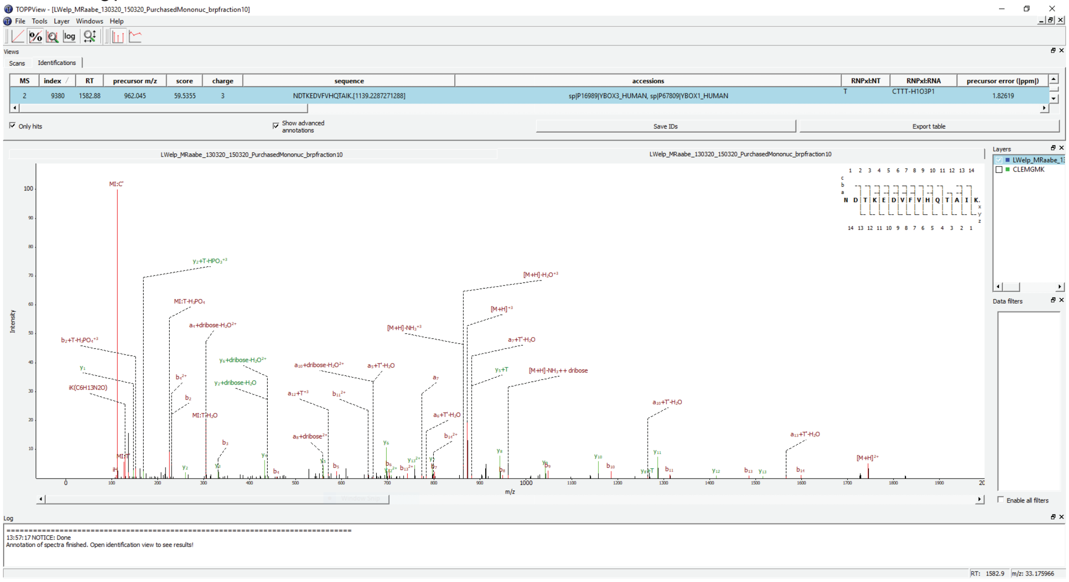


## 2) WD repeat-containing protein 46

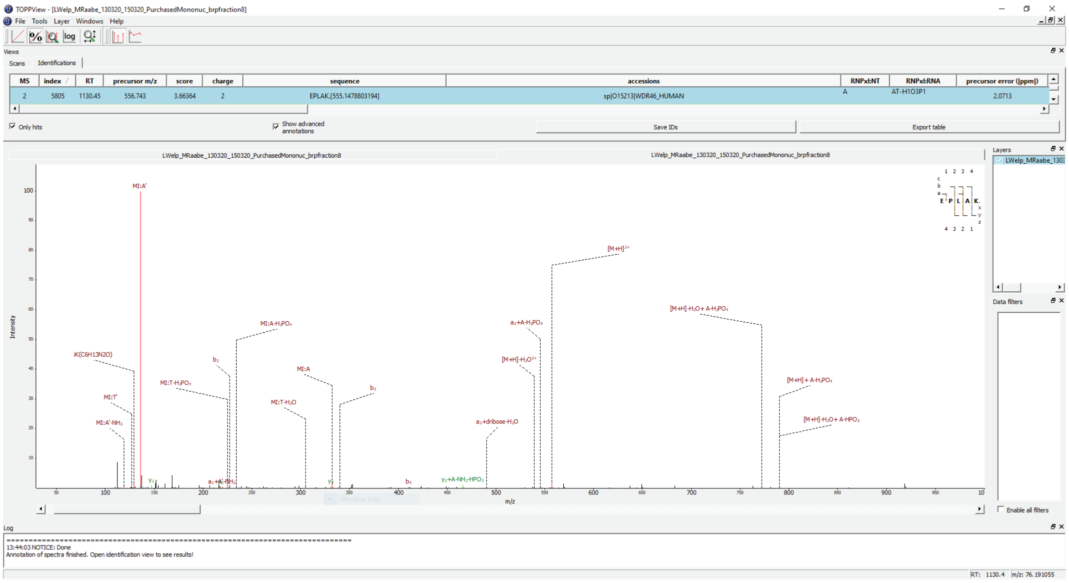


## 3) Histone-lysine N-methyltransferase SUV39H2

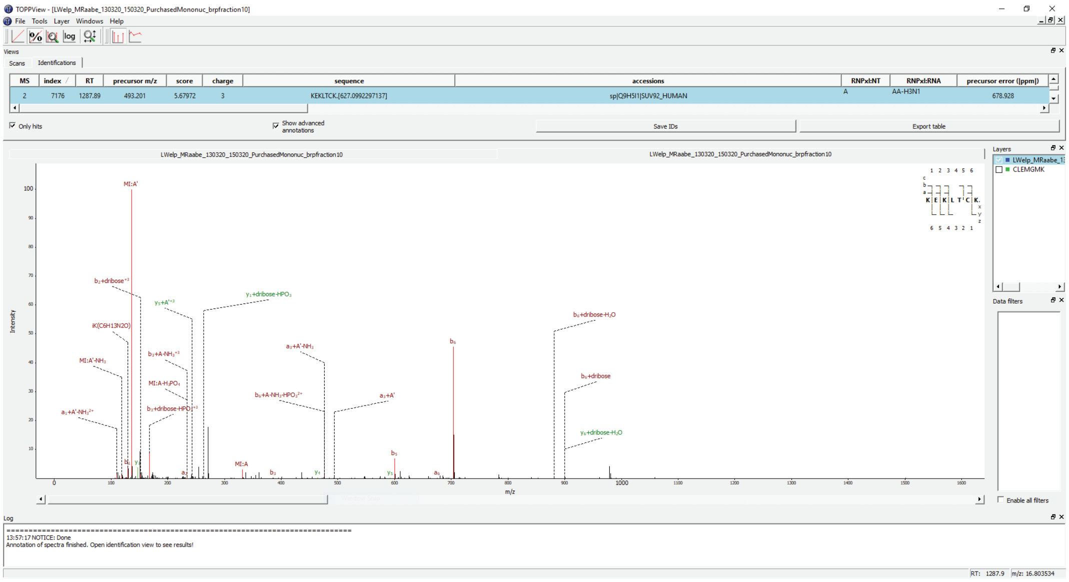

## 4) Catenin Delta I

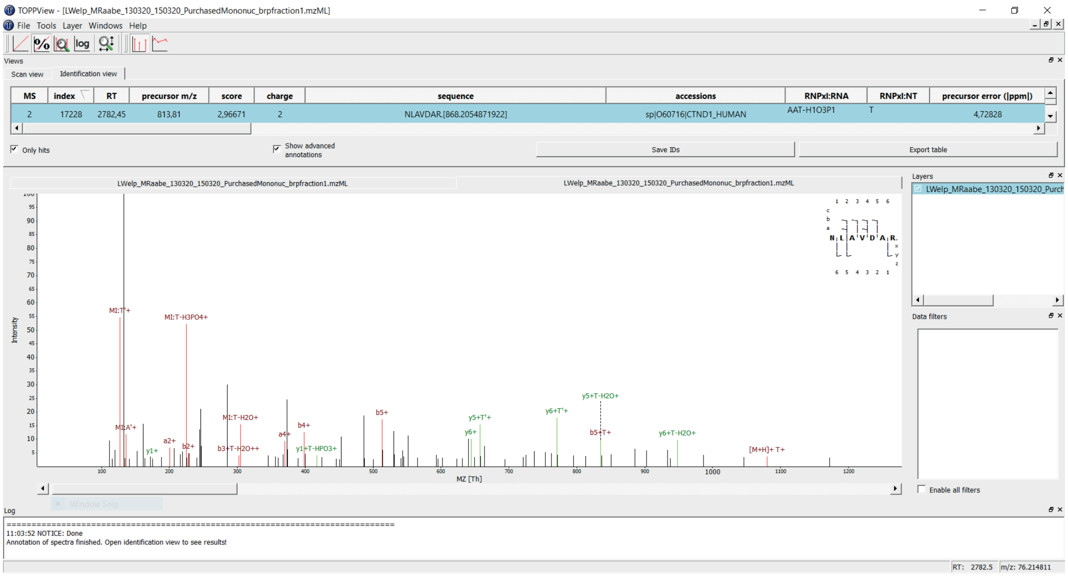


## 5) Histone H2A

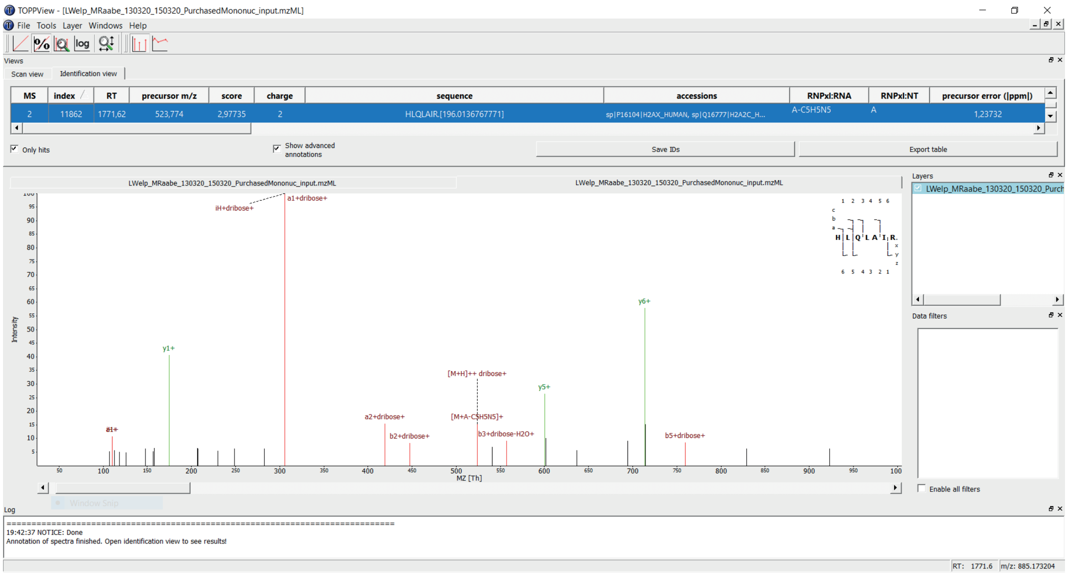


## 6)

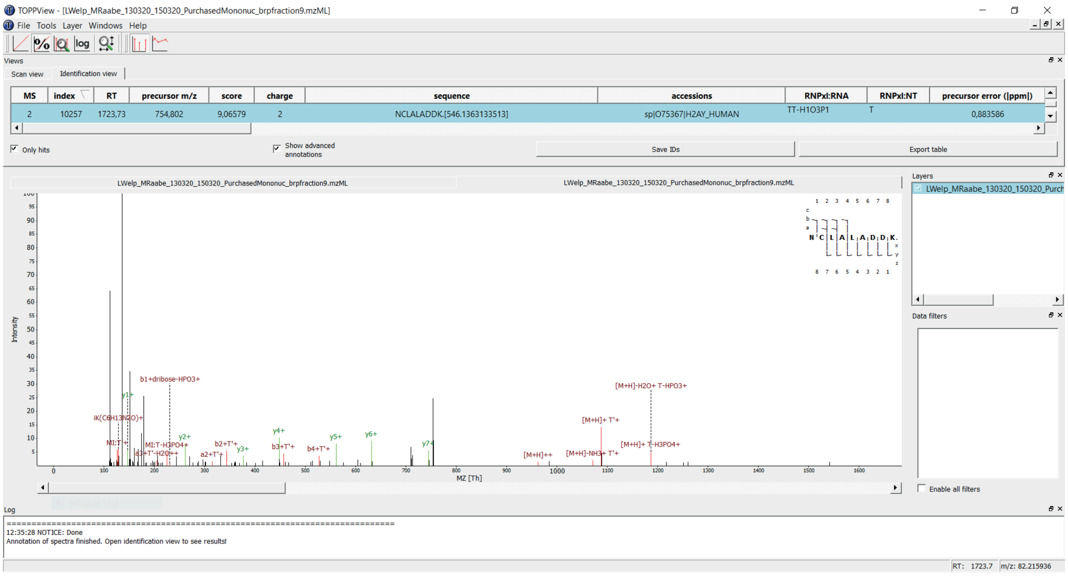

7) Histone H2A

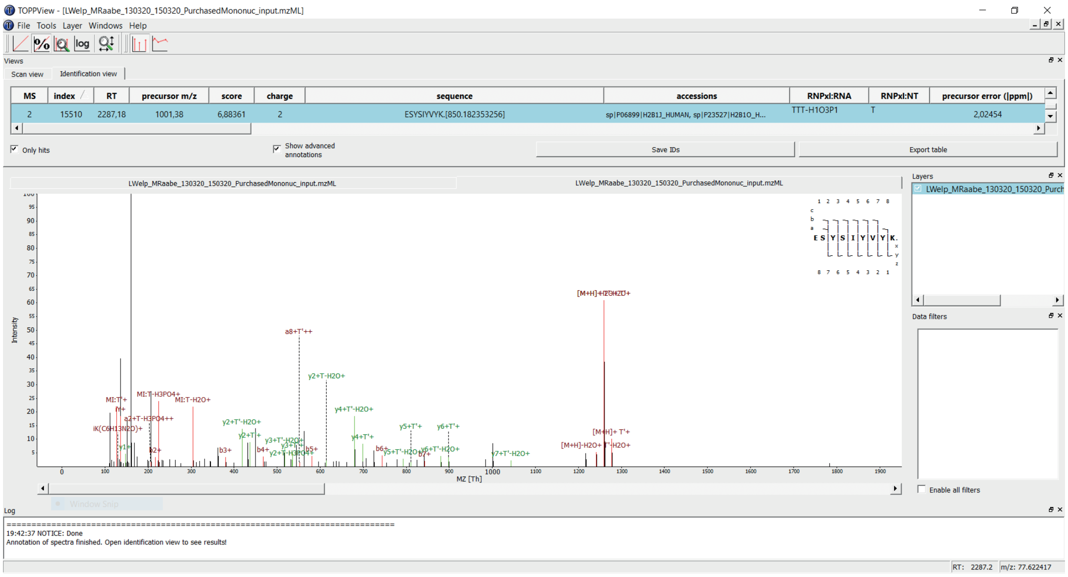


8) Histone H2B

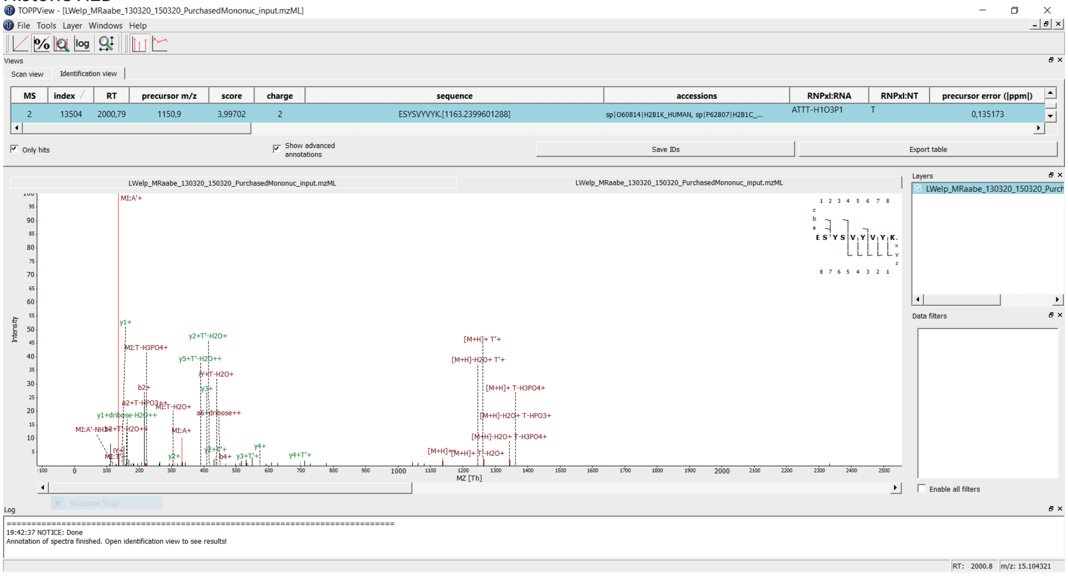


9)

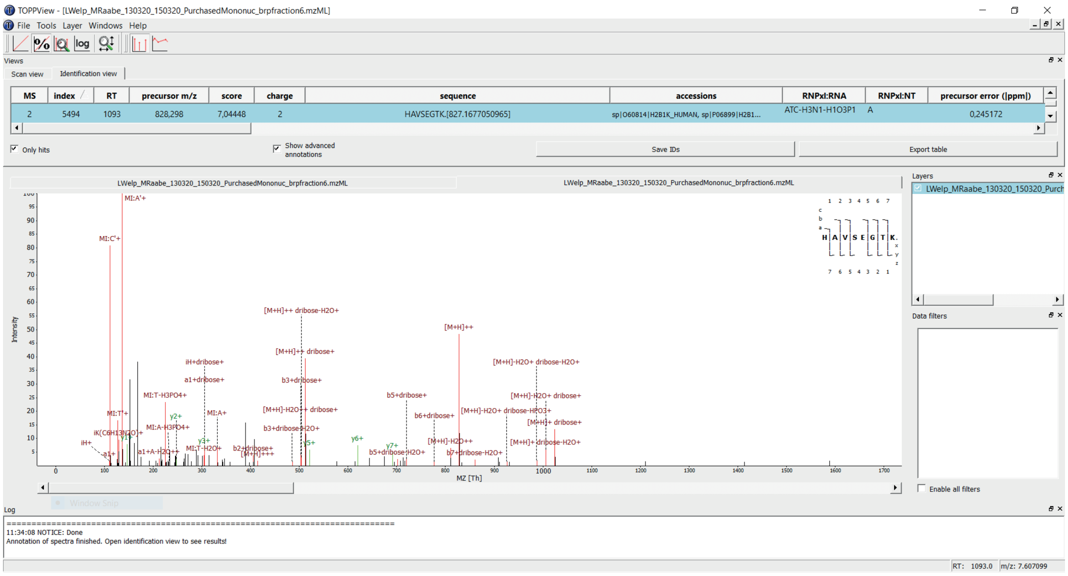

10)

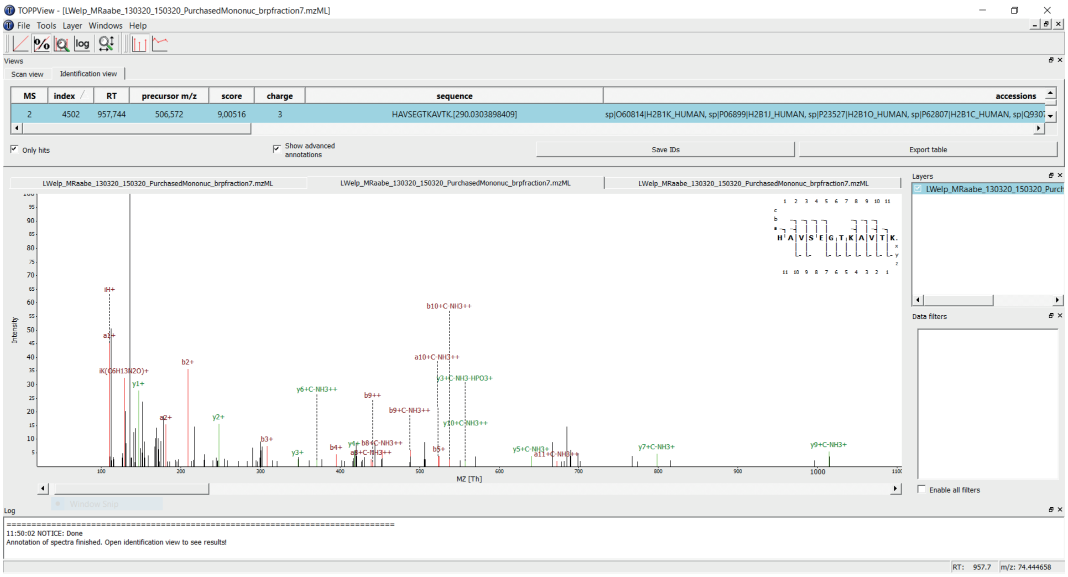
TOPPView - [LWelp_MRaabe_130320_150320_PurchasedMononuc_brpfraction7.mzML]
File Tools Layer Windows Help
Scan view
Identification view
MS | index | RT | precursor m/z | score | charge | sequence | accessions
2 | 4502 | 957,744 | 506,572 | 9,00516 | 3 | HAVSEGTKAVTK.[290.0303898409] | sp|O60814|H2B1K_HUMAN, sp|P06899|H2B1J_HUMAN, sp|P23527|H2B1O_HUMAN, sp|P62807|H2B1C_HUMAN, sp|Q930
Only hits
Show advanced annotations
Save IDs
Export table
Layers
Data filters
Enable all filters
Intensity
MZ [Th]
Log
11:50:02 NOTICE: Done
Annotation of spectra finished. Open identification view to see results!
RT: 957.7
m/z: 74.444658

11)

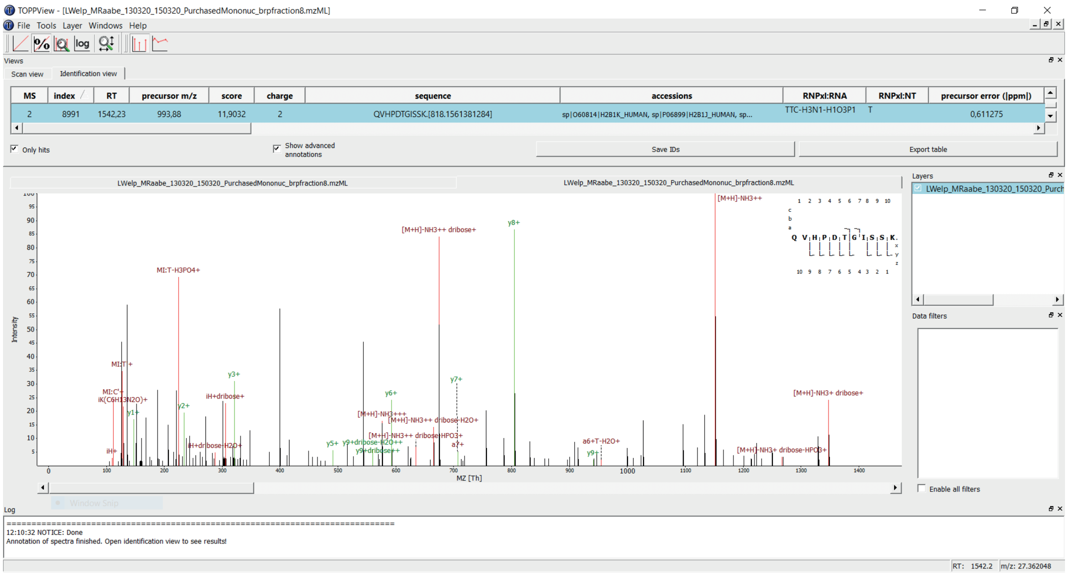
TOPPView - [LWelp_MRaabe_130320_150320_PurchasedMononuc_brpfraction8.mzML]
File Tools Layer Windows Help
Scan view
Identification view
MS | index | RT | precursor m/z | score | charge | sequence | accessions | RNPxl:RNA | RNPxl:NT | precursor error (|ppm|)
2 | 8991 | 1542,23 | 993,88 | 11,9032 | 2 | QVHPDTGISSK.[818.1561381284] | sp|O60814|H2B1K_HUMAN, sp|P06899|H2B1J_HUMAN, sp... | TTC-H3N1-H1O3P1 | T | 0,611275
Only hits
Show advanced annotations
Save IDs
Export table
Layers
Data filters
Enable all filters
Intensity
MZ [Th]
Log
12:10:32 NOTICE: Done
Annotation of spectra finished. Open identification view to see results!
RT: 1542.2
m/z: 27.362048

12)

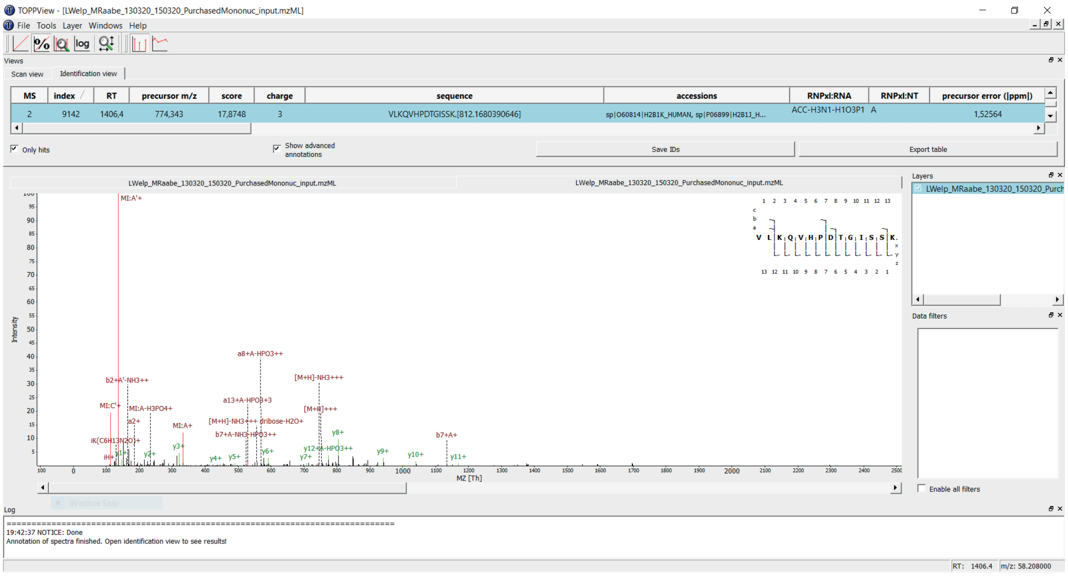
TOPPView - [LWelp_MRaabe_130320_150320_PurchasedMononuc_input.mzML]
File Tools Layer Windows Help
Scan view
Identification view
MS | index | RT | precursor m/z | score | charge | sequence | accessions | RNPxl:RNA | RNPxl:NT | precursor error (|ppm|)
2 | 9142 | 1406,4 | 774,343 | 17,8748 | 3 | VLKQVHPDTGISSK.[812.1680390646] | sp|O60814|H2B1K_HUMAN, sp|P06899|H2B1J_H... | ACC-H3N1-H1O3P1 | A | 1,52564
Only hits
Show advanced annotations
Save IDs
Export table
Layers
Data filters
Enable all filters
Intensity
MZ [Th]
Log
19:42:37 NOTICE: Done
Annotation of spectra finished. Open identification view to see results!
RT: 1406.4
m/z: 58.208000

13) Histone H3

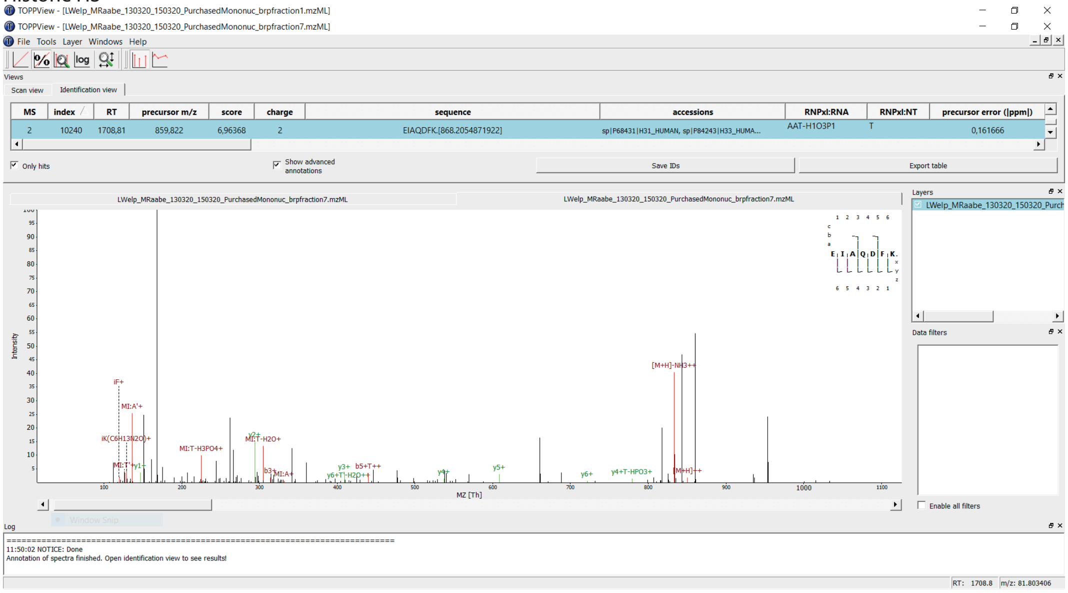


14)

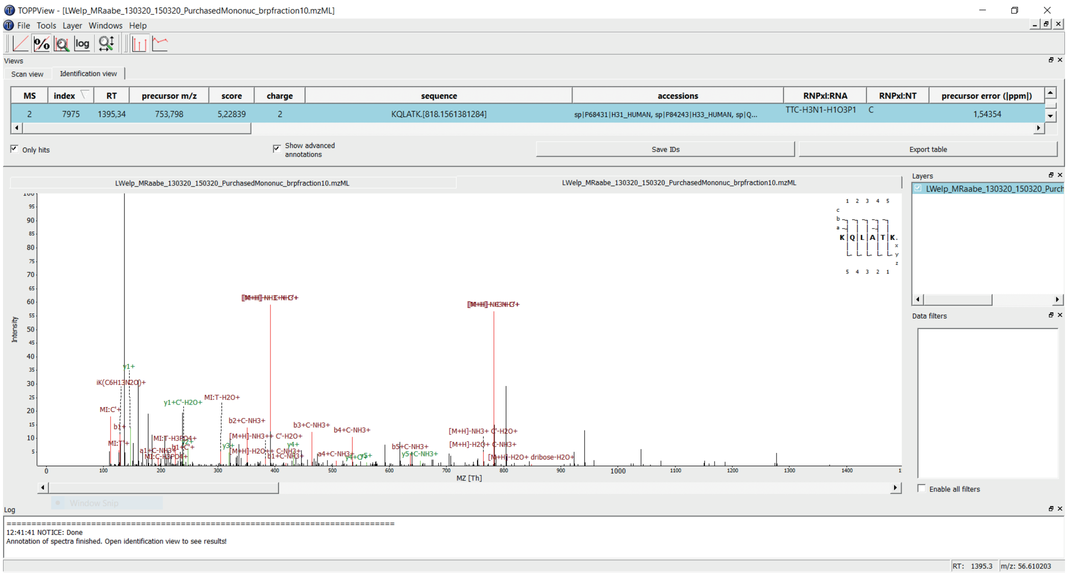


15)

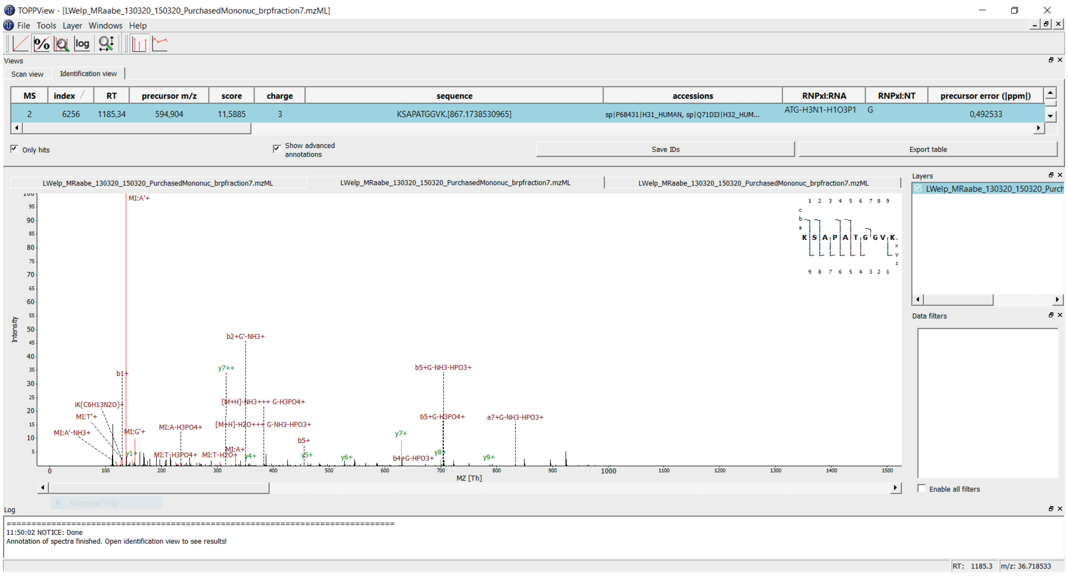

**16)**

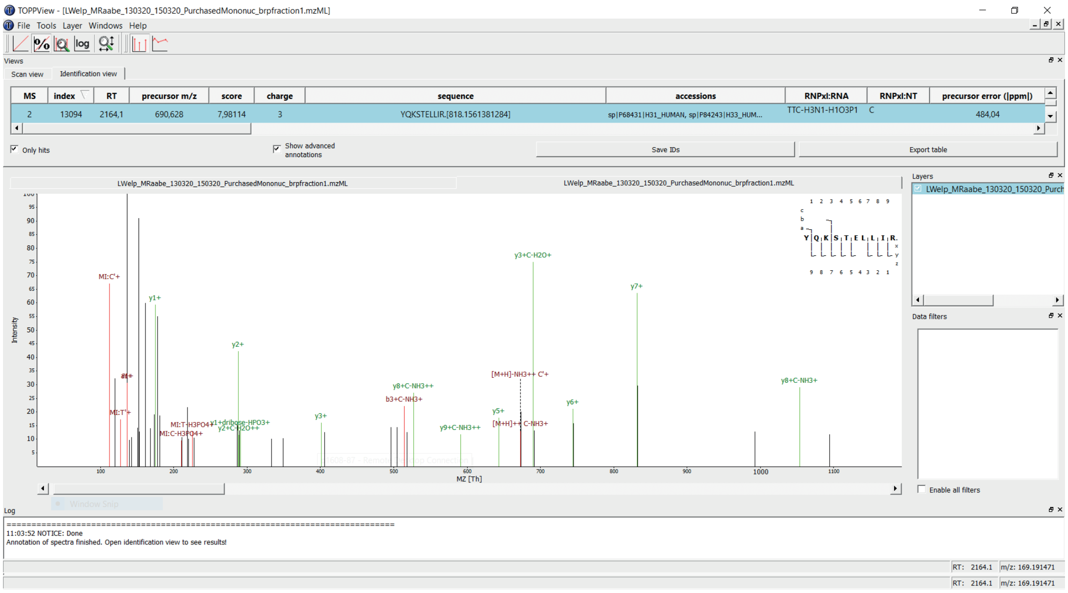


**17)**

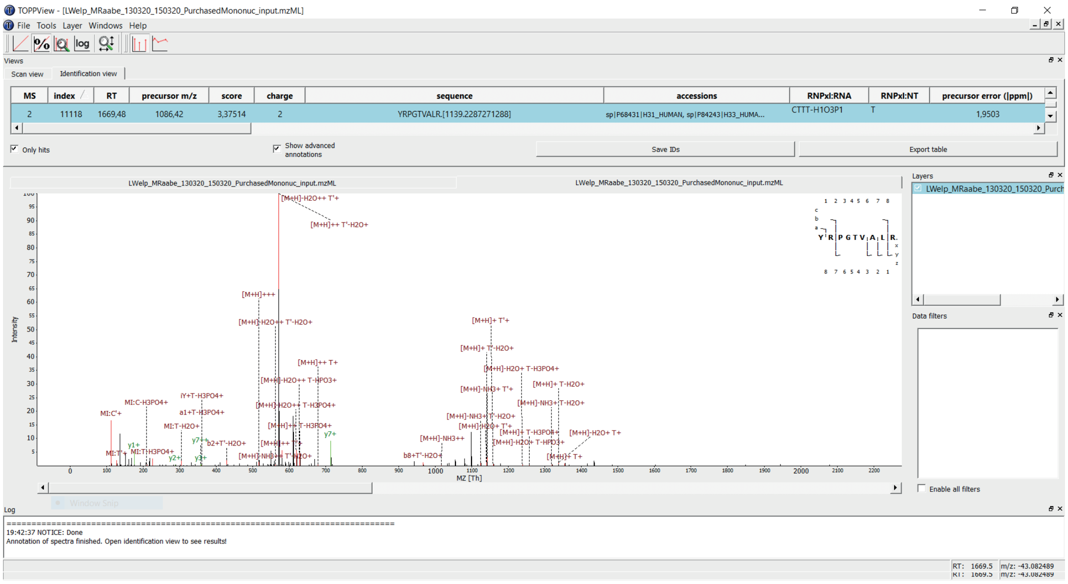


**18)** Histone 4

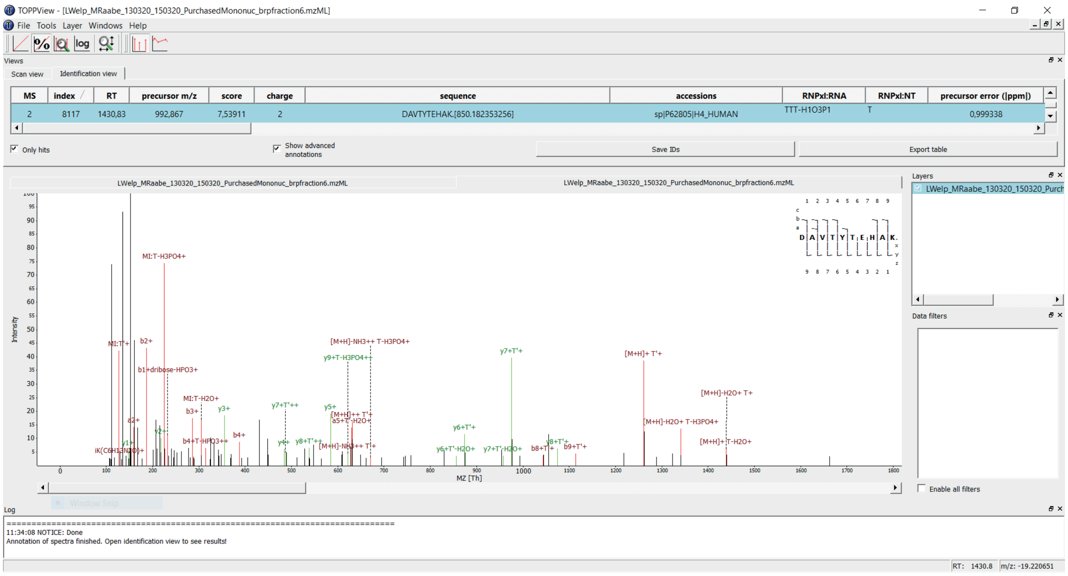

## 19)

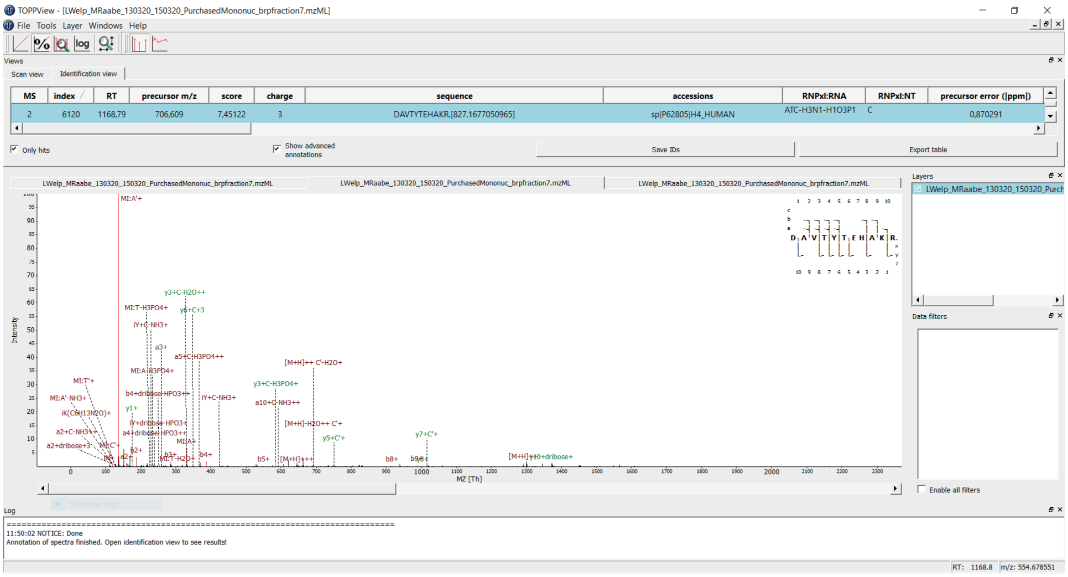


## 20) Heterogeneous nuclear ribonucleoprotein C-like 1

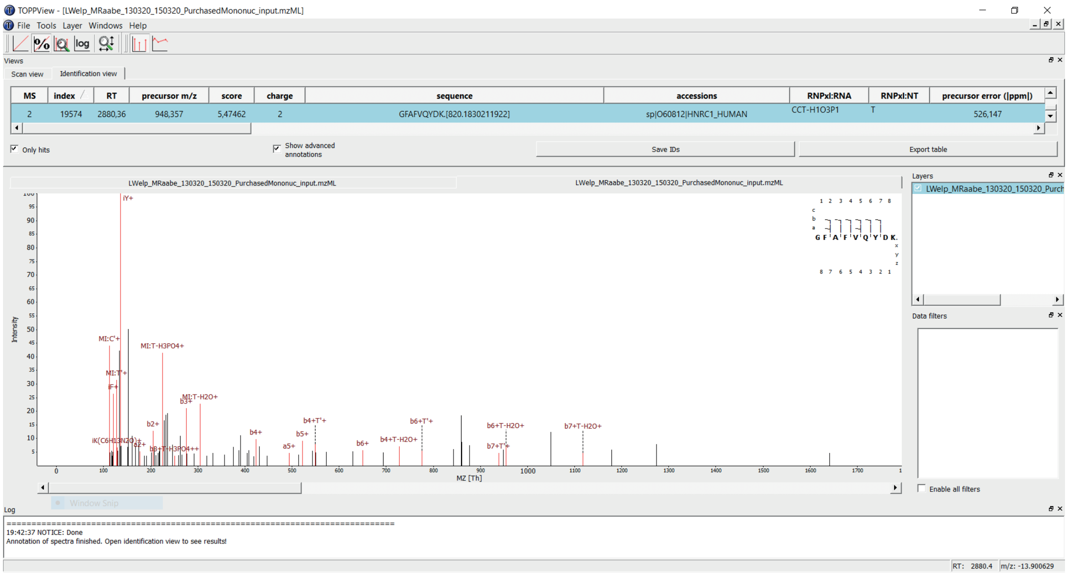


## 21)

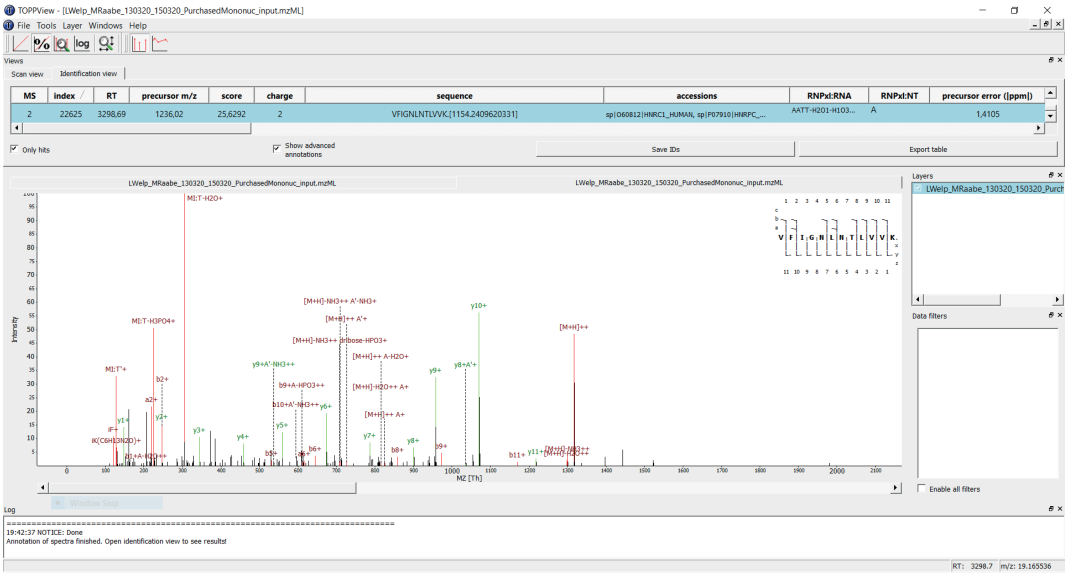

## 22) Heterogeneous nuclear ribonucleoprotein D-like

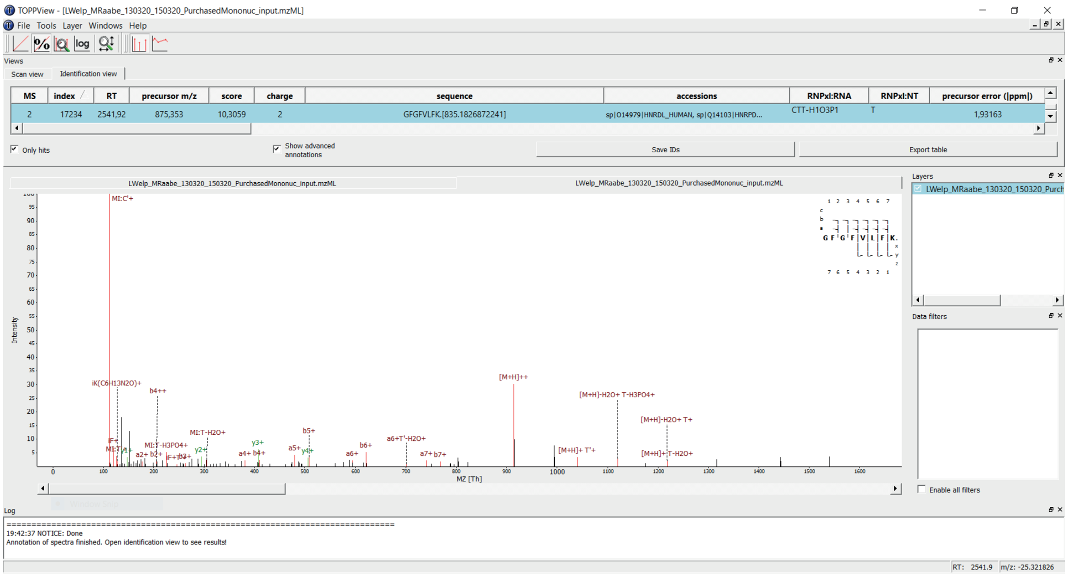


## 23)

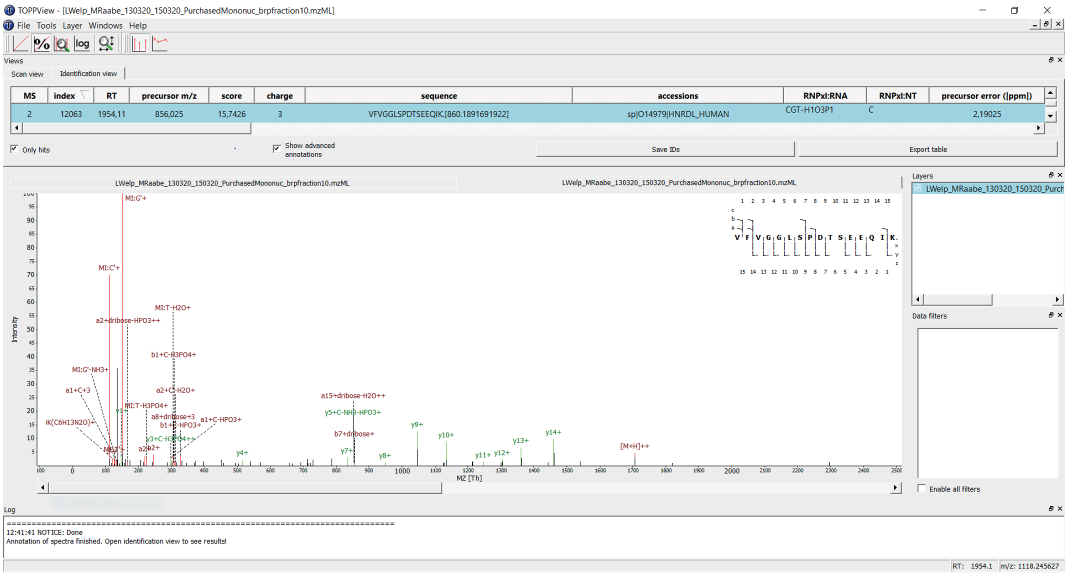


## 24) Heterogeneous nuclear ribonucleoproteins C1/C2

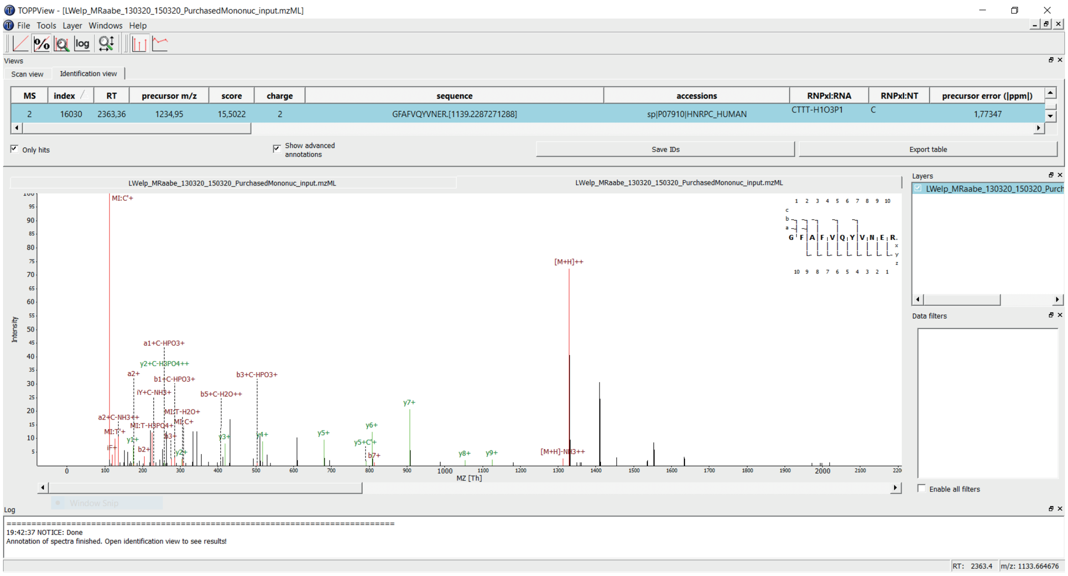

# 25)

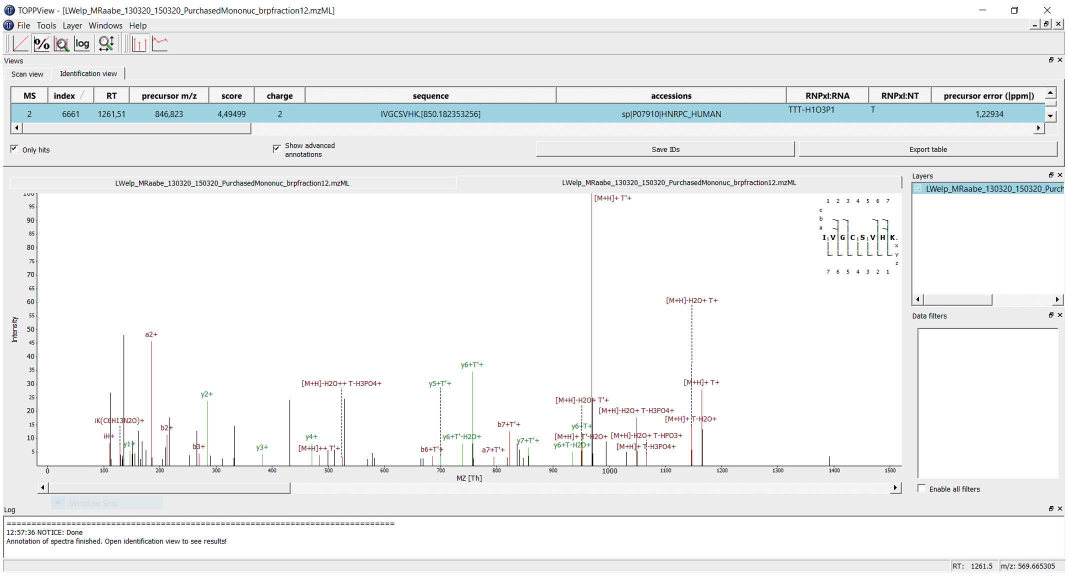


# 26) Heterogeneous nuclear ribonucleoprotein D0

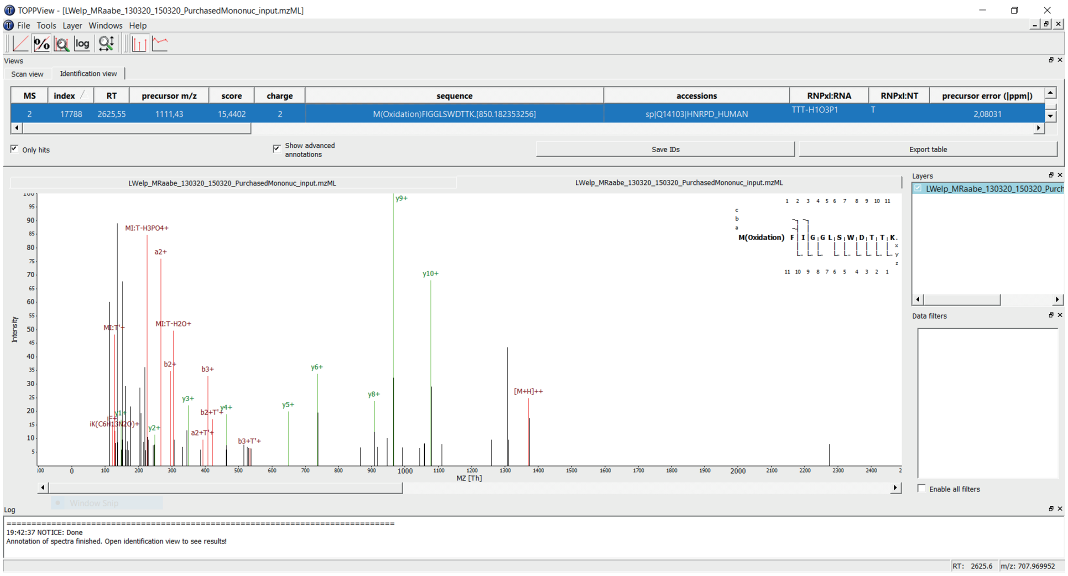


# 27) Myb-binding protein 1A

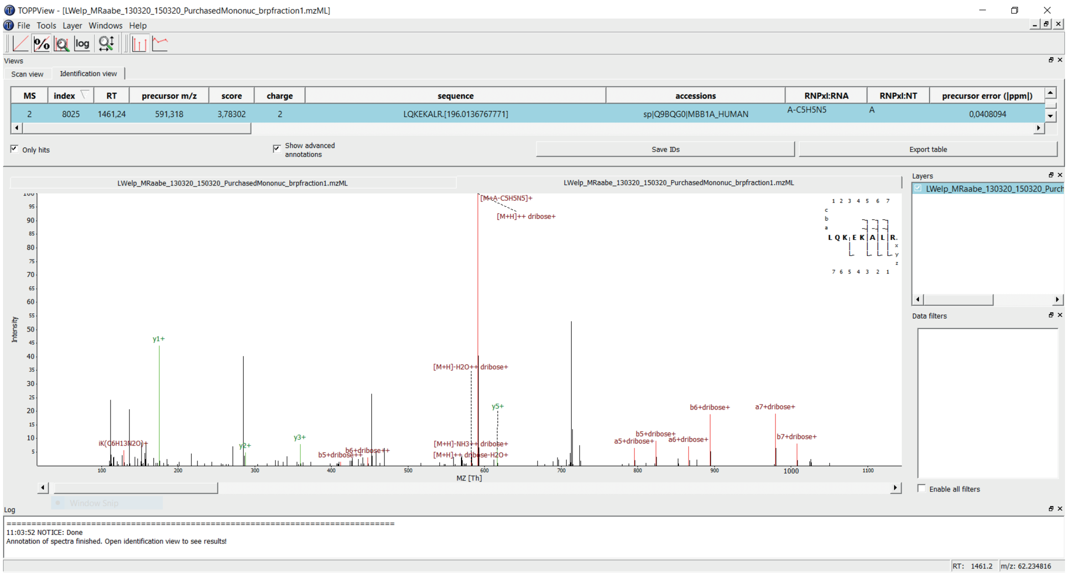

## 28) Histone-lysine N-methyltransferase NSD2

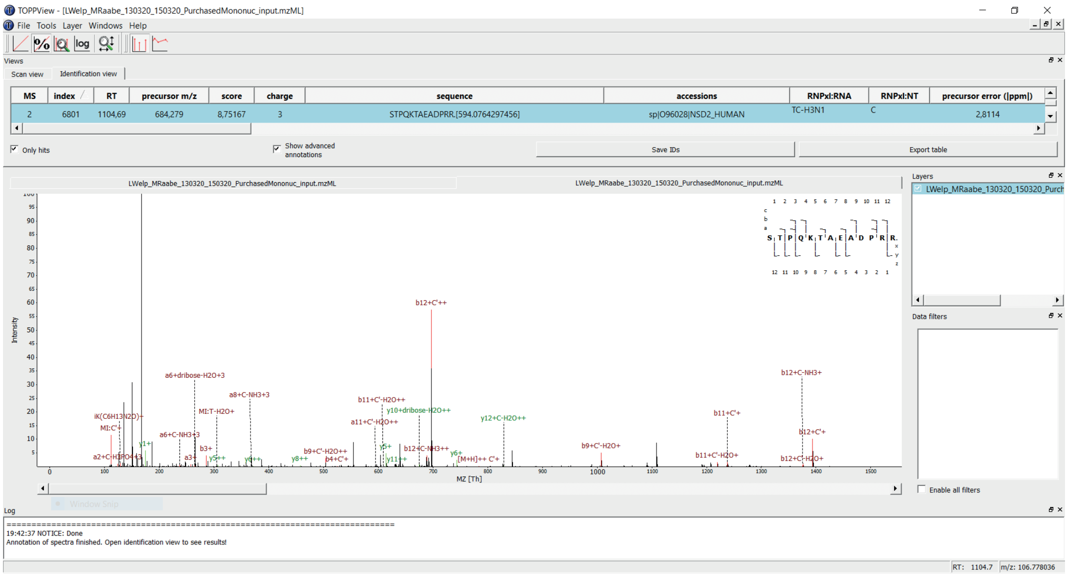


## 29) PHD finger protein 20

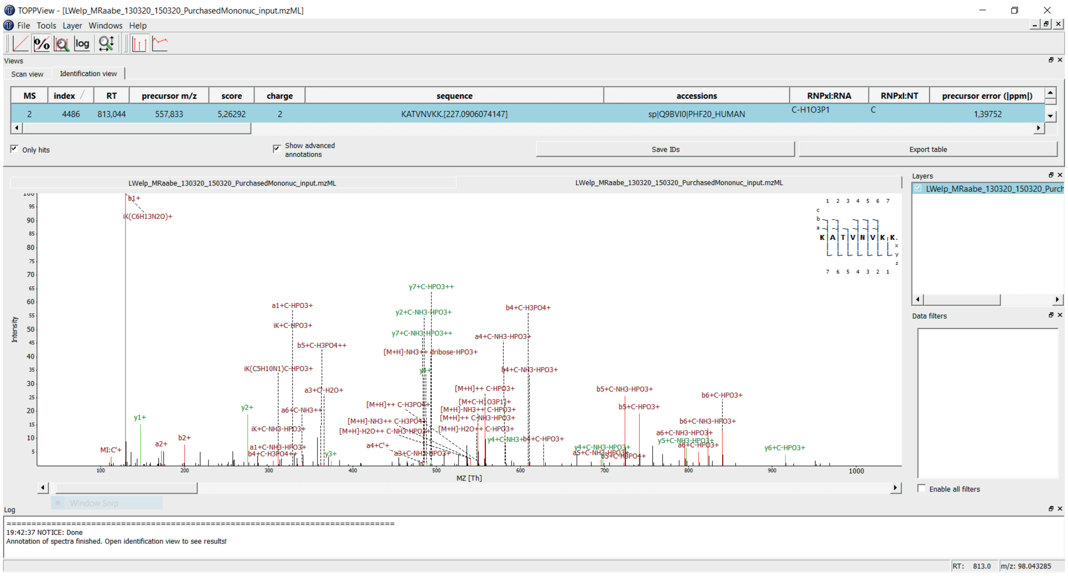


## 30) Polypyrimidine tract-binding protein 1

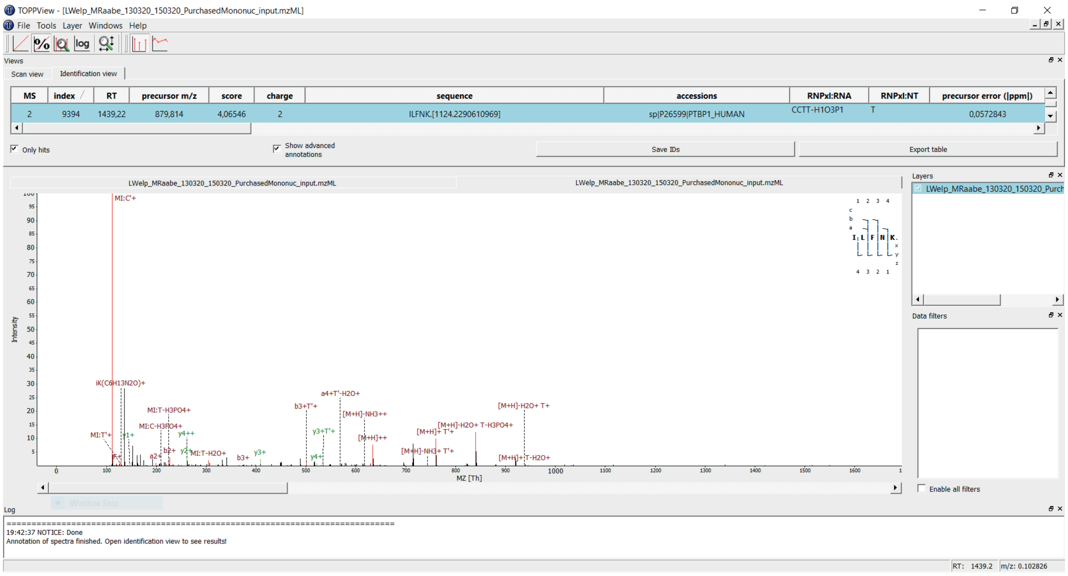

## 31) RNA-binding protein Raly

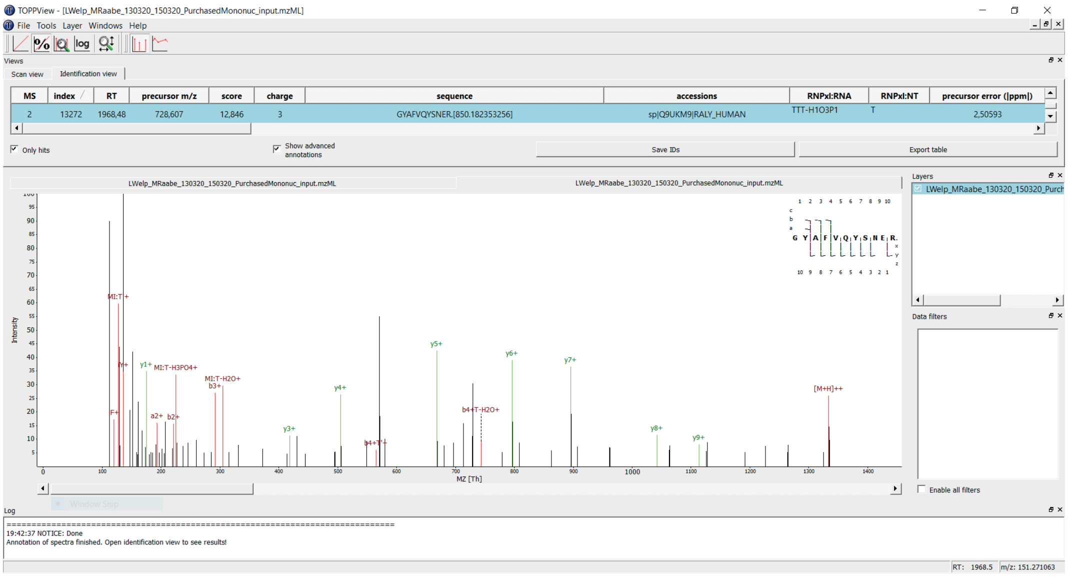


## 32)

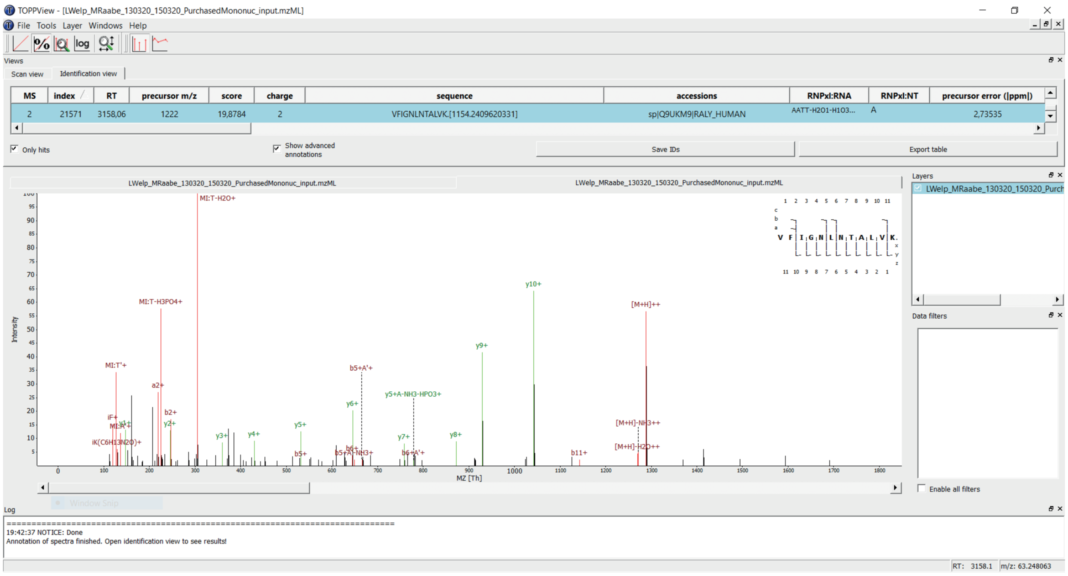


## 33) Heterogeneous nuclear ribonucleoprotein A1

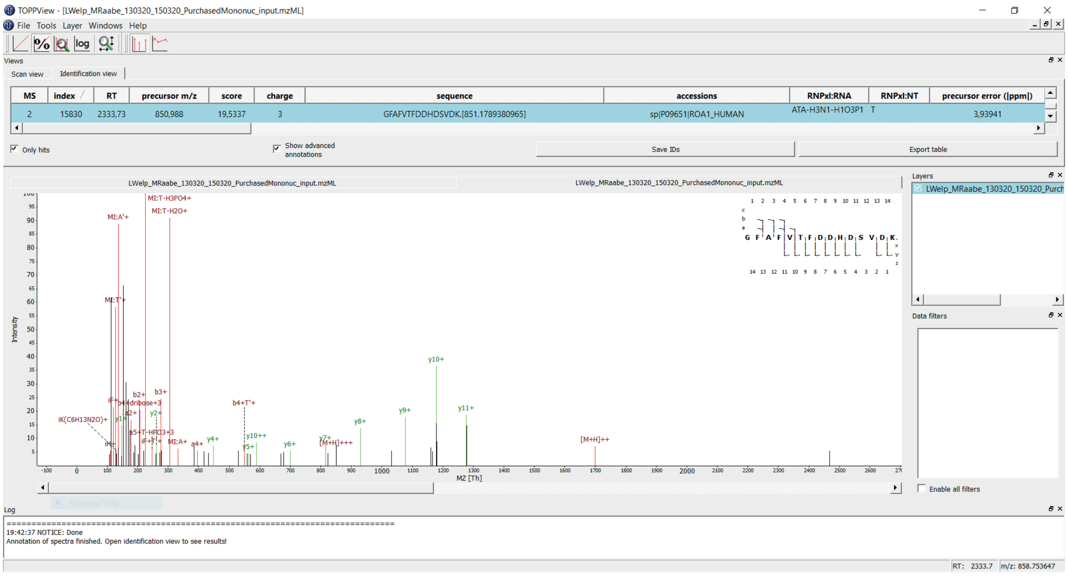

## 34)

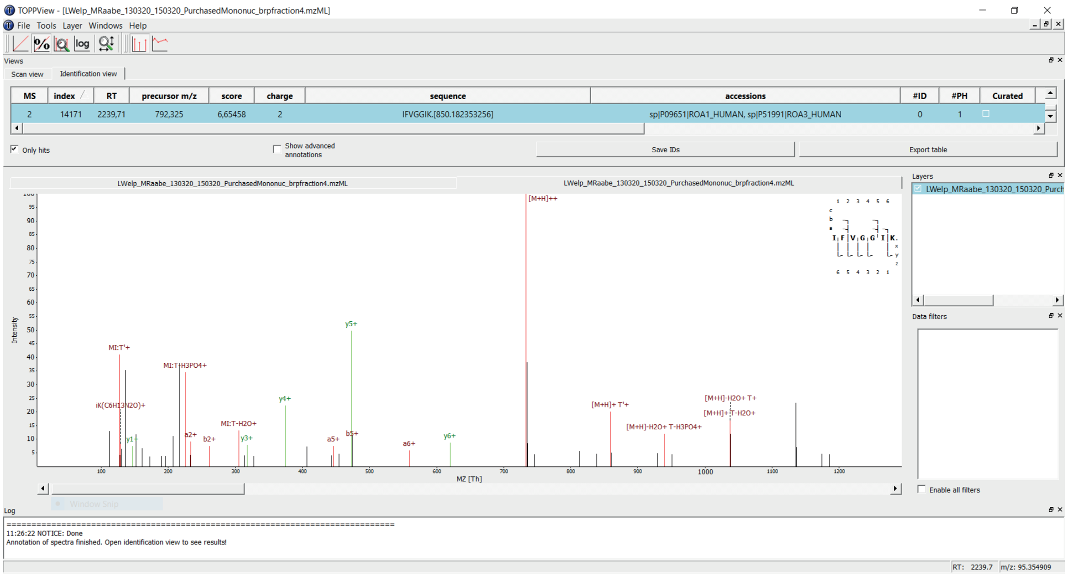


## 35) Splicing factor 3B subunit 4

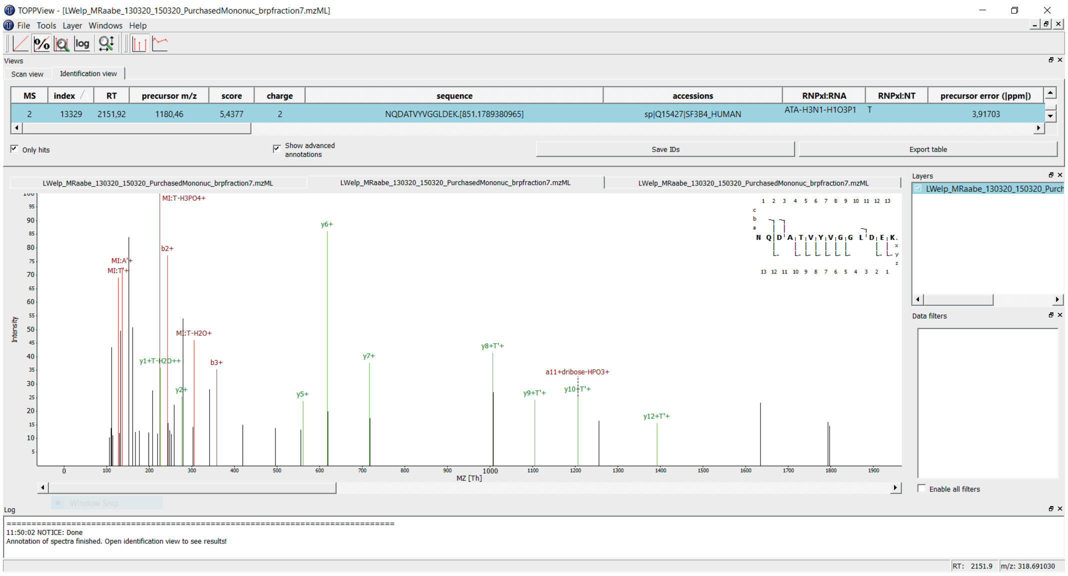


## 36) DNA topoisomerase 2-alpha

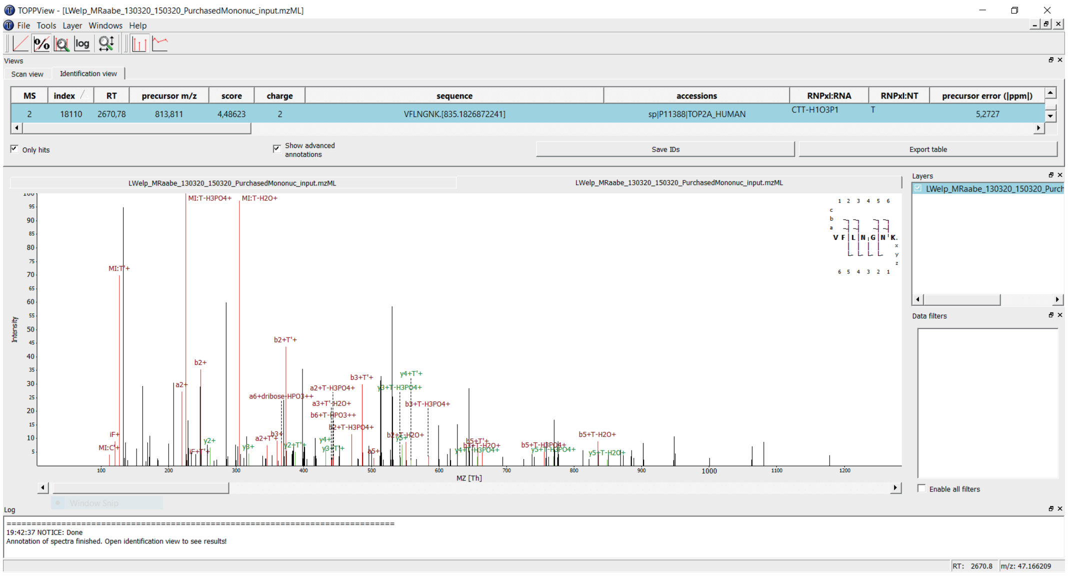

## 37) Tripartite motif-containing protein 26

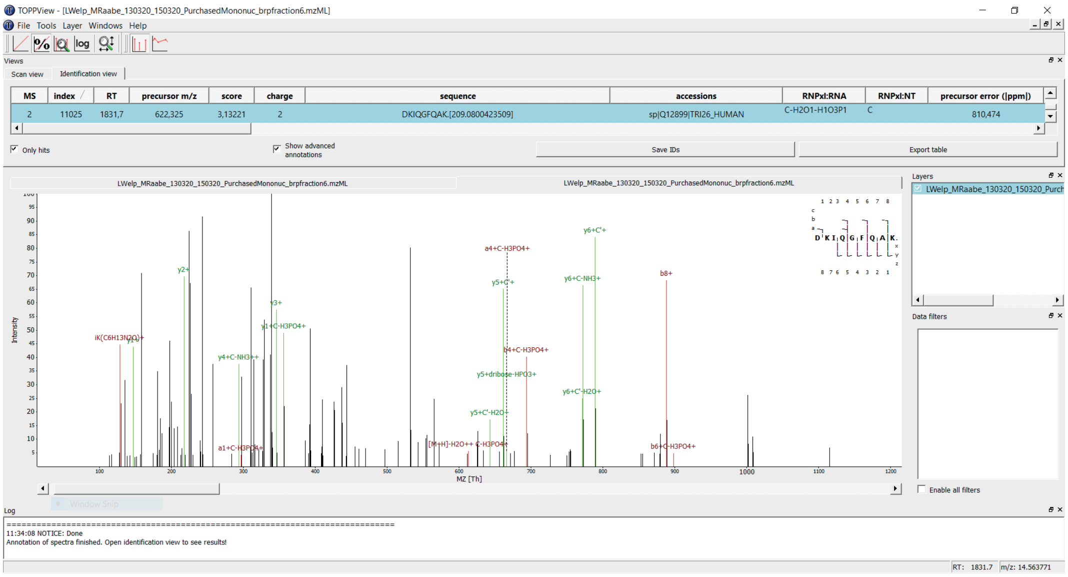


## 38) Y-box-binding protein 3

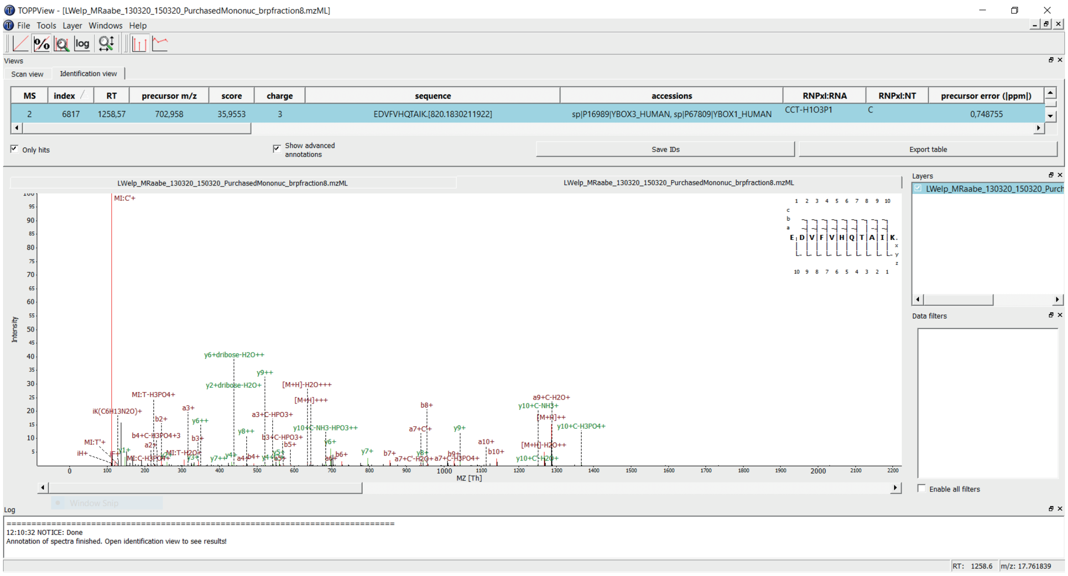


## 39)

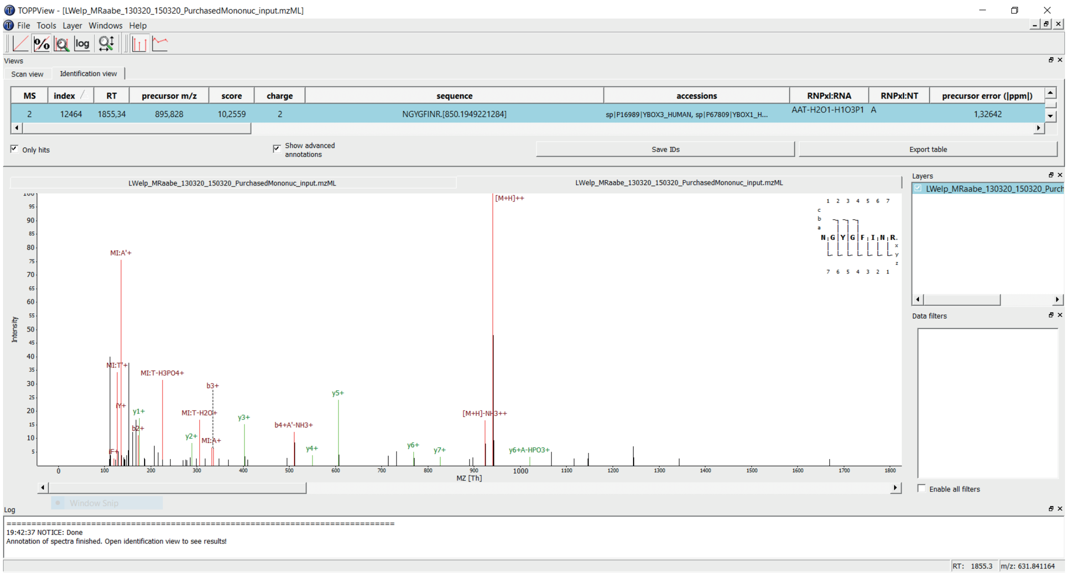

## 40) Neuroblast differentiation-associated protein AHNAK

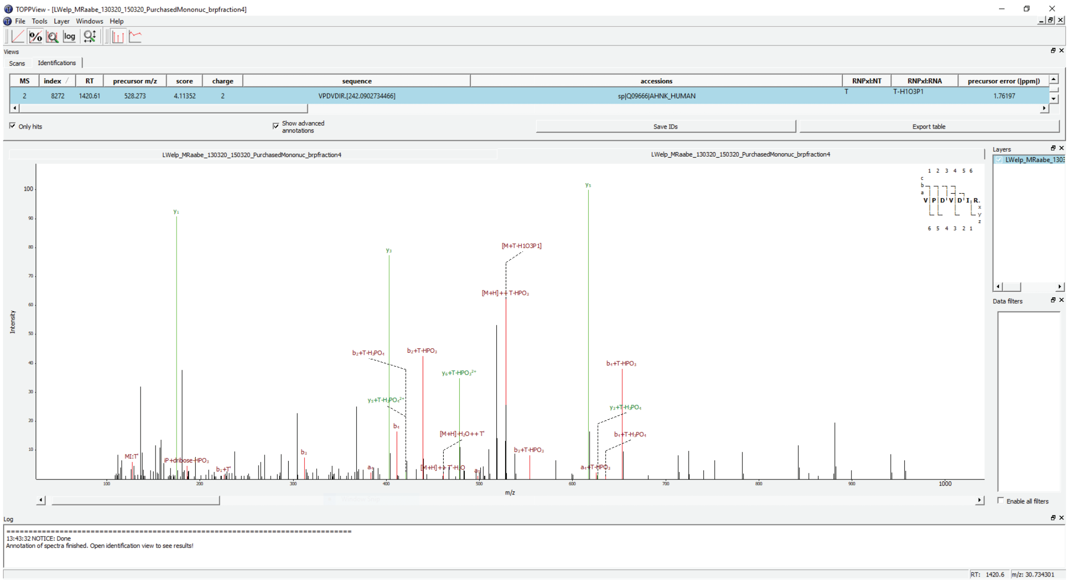


## 41) Protein arginine N-methyltransferase 5

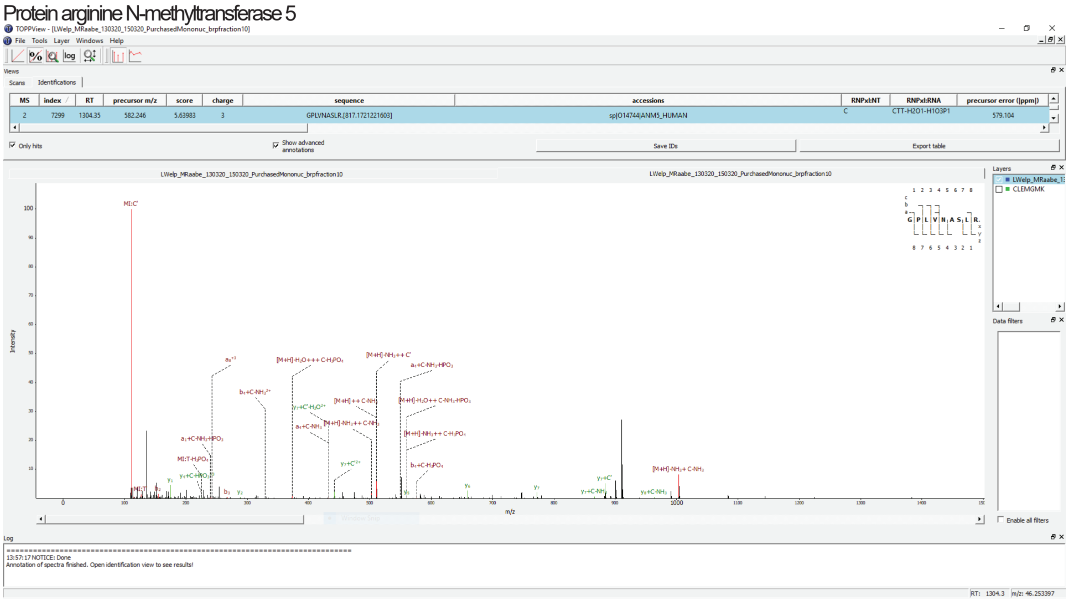


## 42) Serine-protein kinase ATM

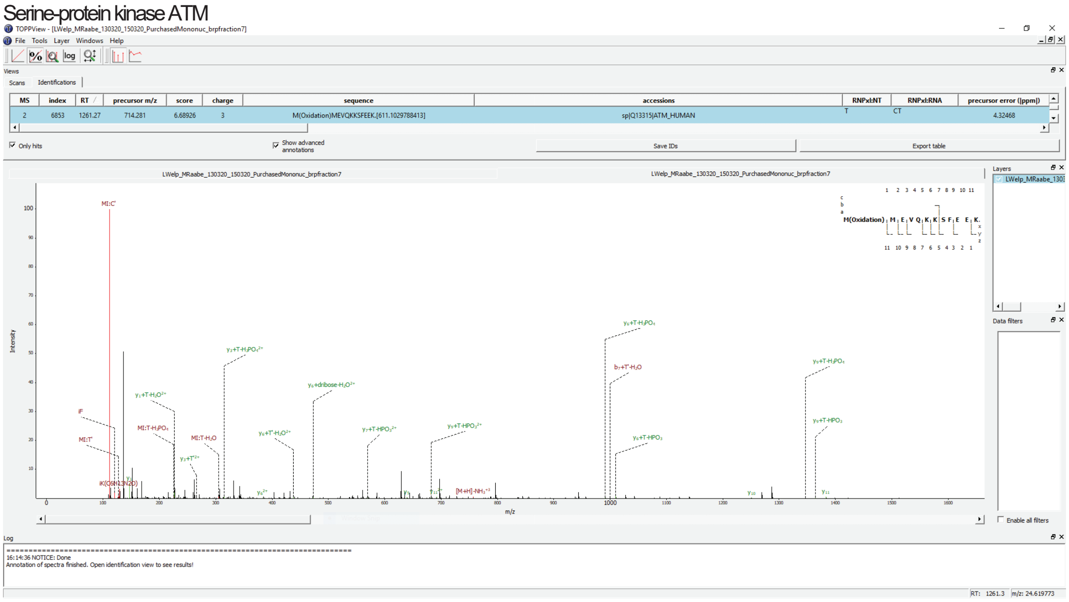

## 43) Class E basic helix-loop-helix protein 40

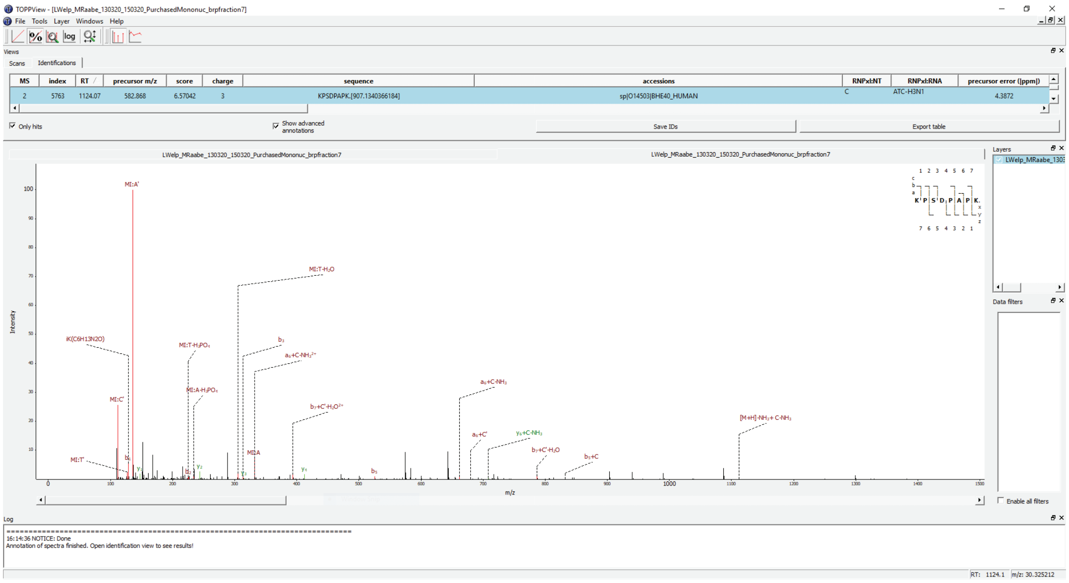


## 44) Bromodomain-containing protein 4

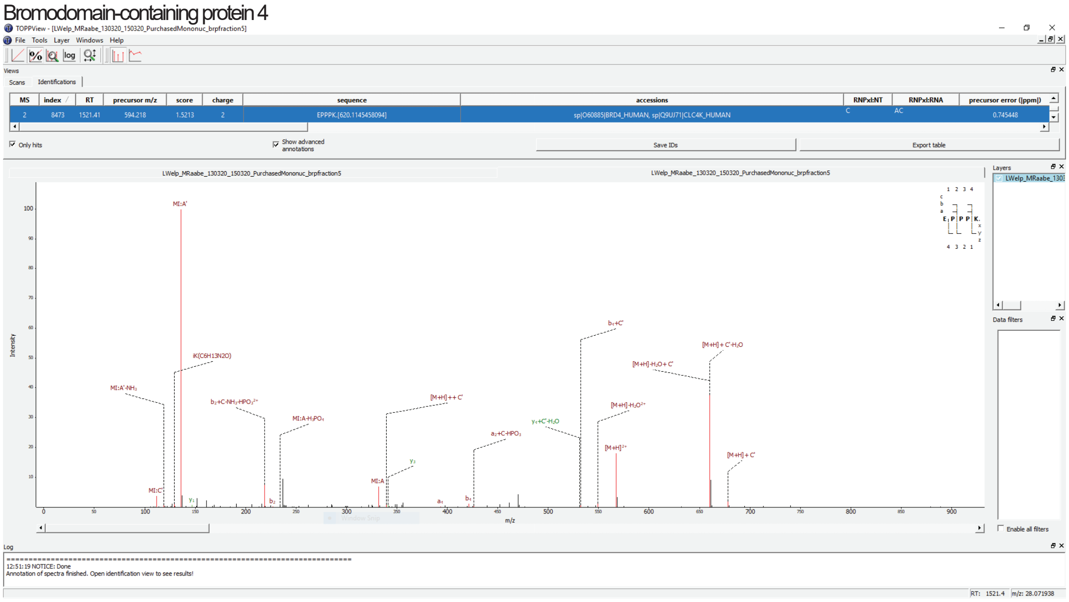


## 45) Chromatin assembly factor 1 subunit A

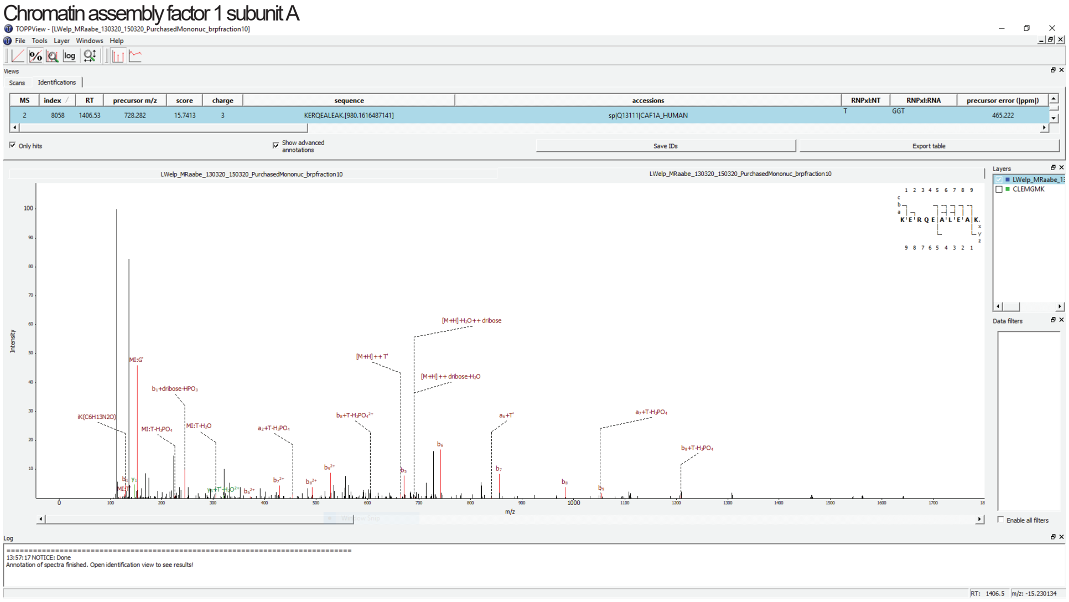

## 46) Centromere protein F

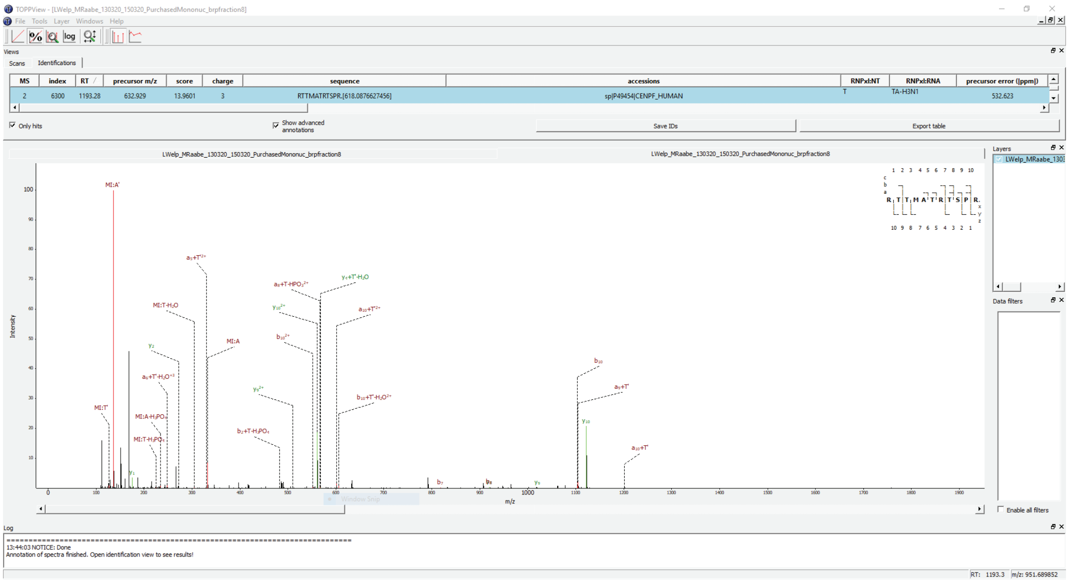


## 47) Histone H2B

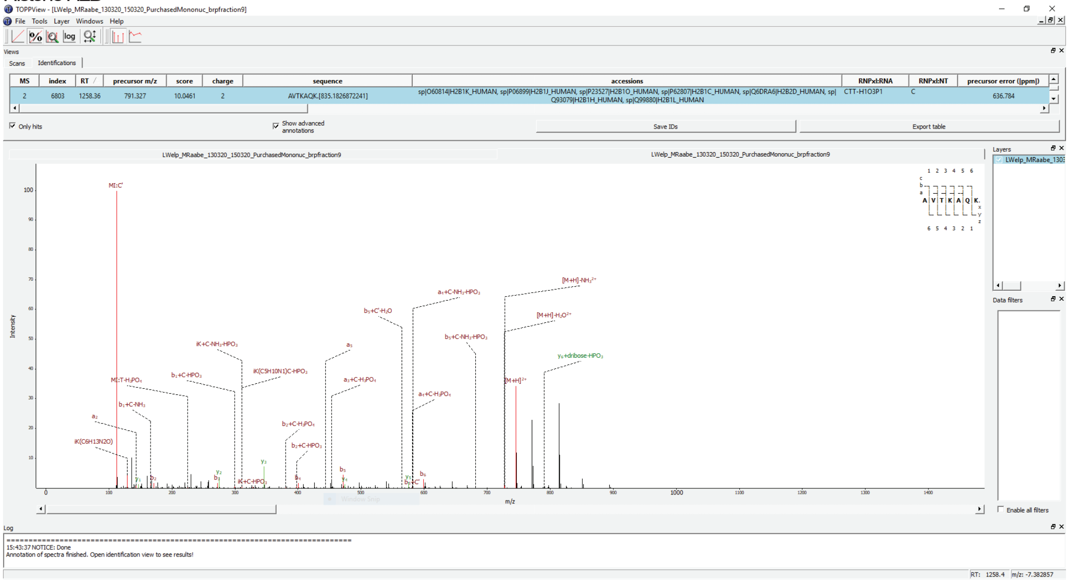


## 48)

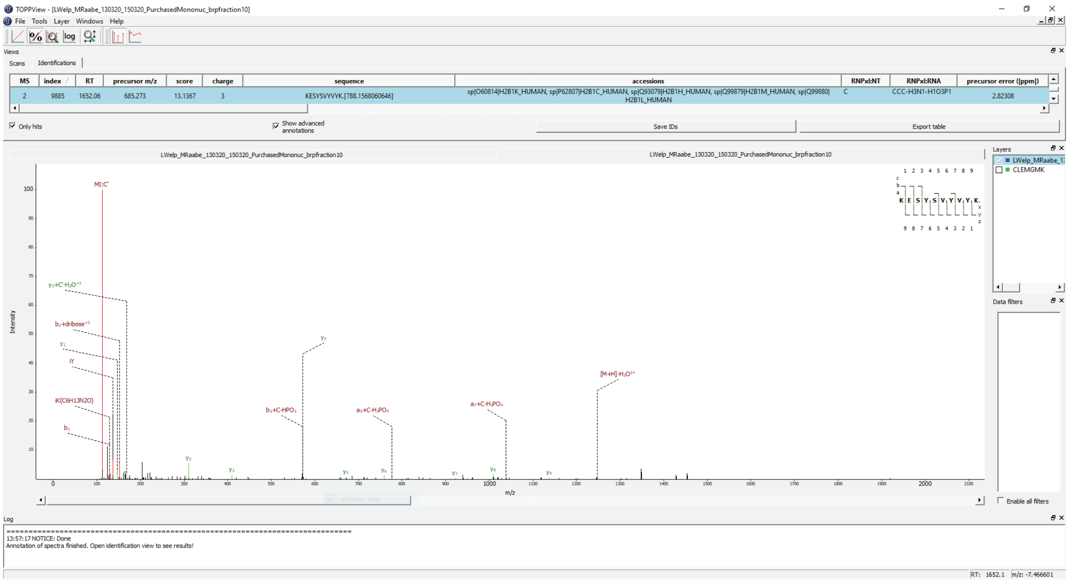

49)

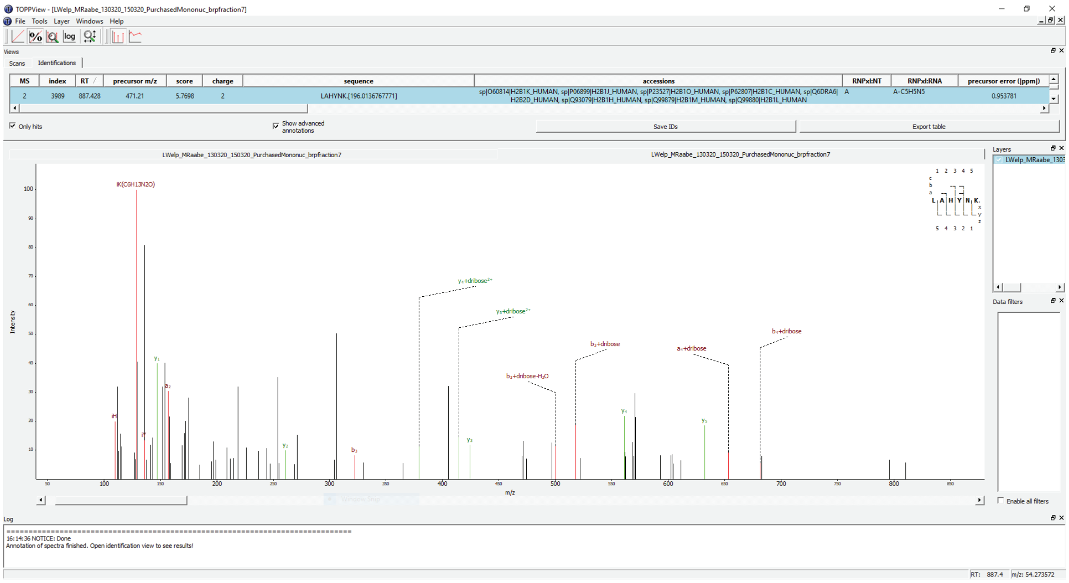
TOPPView - [LWelp_MRaabe_130320_150320_PurchasedMononuc_brpfraction7]
MS | index | RT | precursor m/z | score | charge | sequence | accessions | RNPxl:NT | RNPxl:RNA | precursor error (|ppm|)
2 | 3989 | 887.428 | 471.21 | 5.7698 | 2 | LAHYNK.[196.0136767771] | A | A-C5H5N5 | 0.953781
Save IDs
Export table
Annotation of spectra finished. Open identification view to see results!

50)

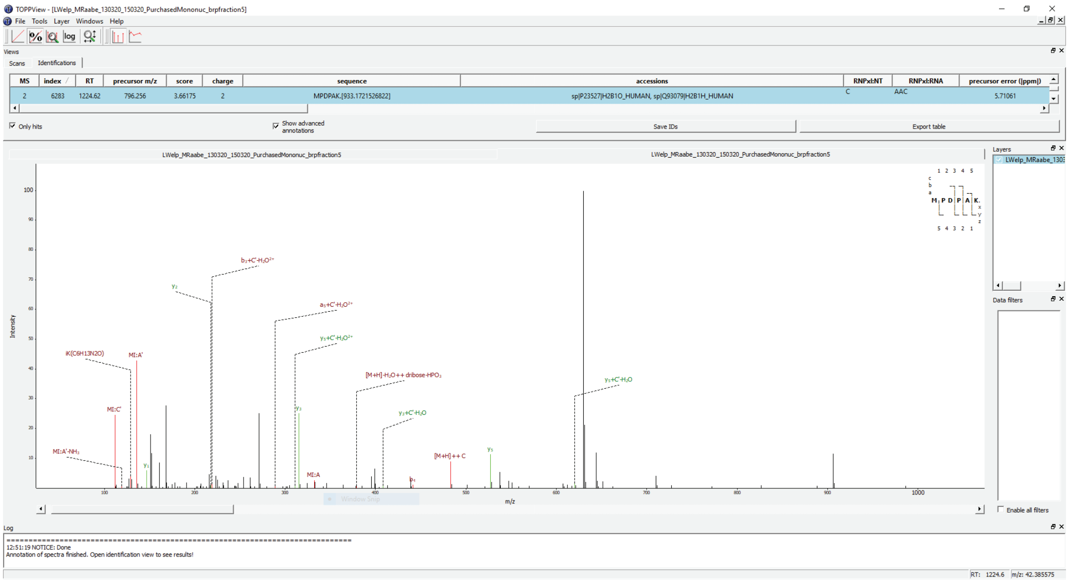
TOPPView - [LWelp_MRaabe_130320_150320_PurchasedMononuc_brpfraction5]
MS | index | RT | precursor m/z | score | charge | sequence | accessions | RNPxl:NT | RNPxl:RNA | precursor error (|ppm|)
2 | 6283 | 1224.62 | 796.256 | 3.66175 | 2 | MPDPAK.[933.1721526822] | sp|P23527|H2B1O_HUMAN, sp|Q93079|H2B1H_HUMAN | C | AAC | 5.71061
Save IDs
Export table
Annotation of spectra finished. Open identification view to see results!

51)

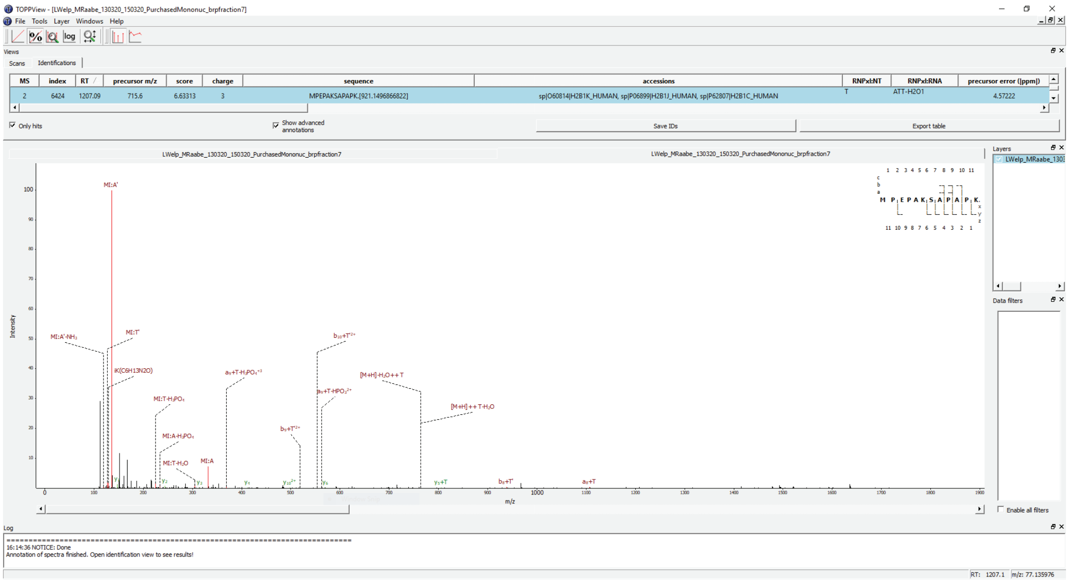
TOPPView - [LWelp_MRaabe_130320_150320_PurchasedMononuc_brpfraction7]
MS | index | RT | precursor m/z | score | charge | sequence | accessions | RNPxl:NT | RNPxl:RNA | precursor error (|ppm|)
2 | 6424 | 1207.09 | 715.6 | 6.63313 | 3 | MPEPAKSAPAPK.[921.1496866822] | T | ATT-H2O1 | 4.57222
Save IDs
Export table
Annotation of spectra finished. Open identification view to see results!

## 52) Histone H4

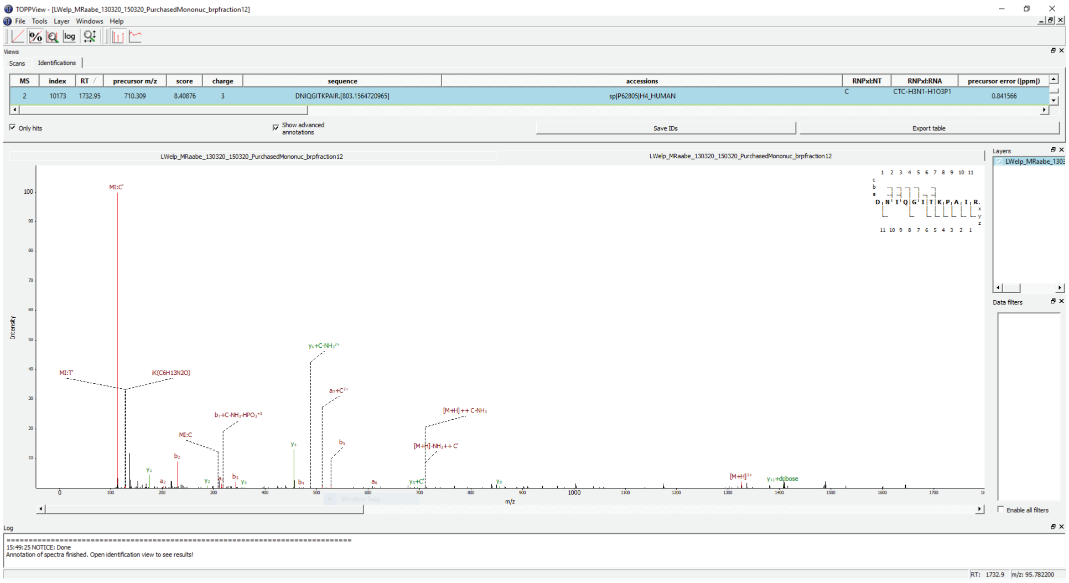


## 53)

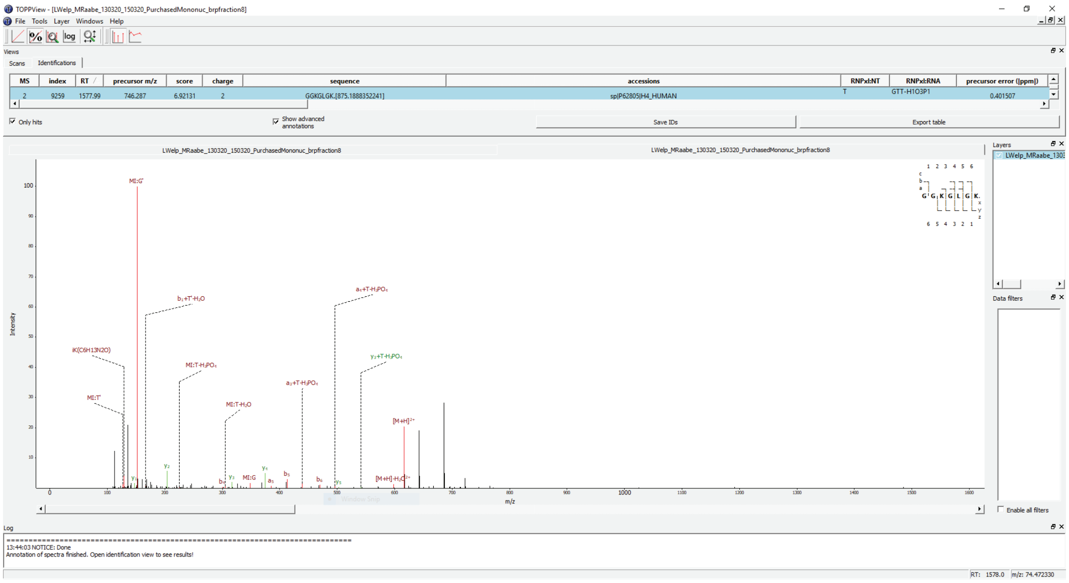


## 54) Histone H3

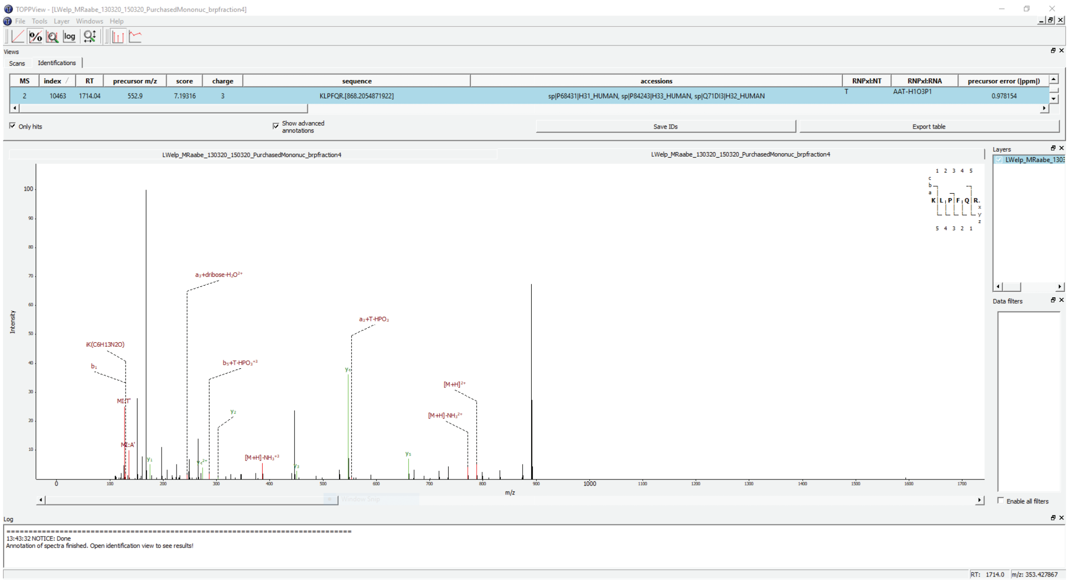

55)

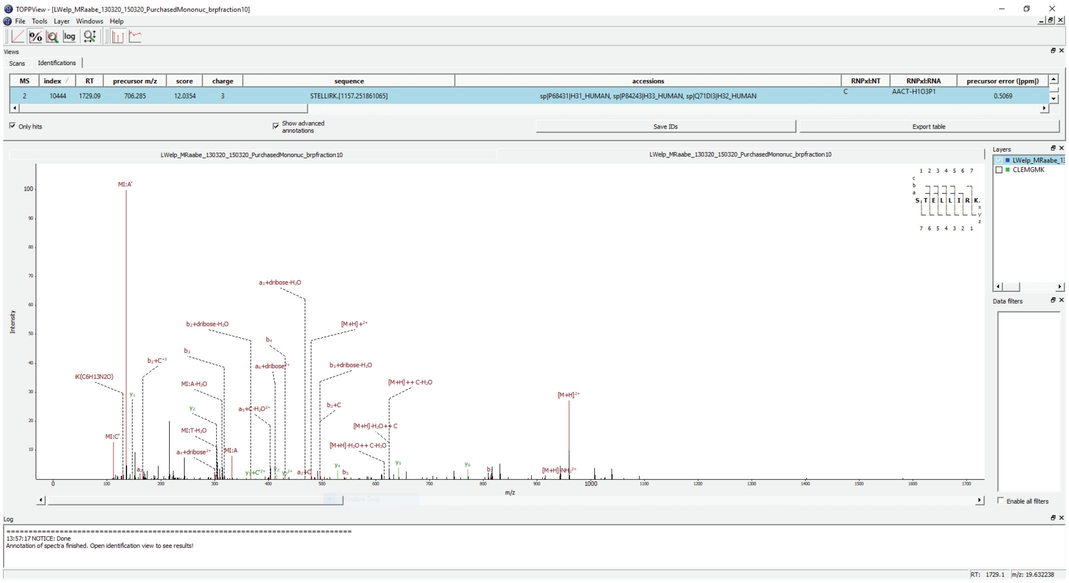


56) 28 kDa heat- and acid-stable phosphoprotein

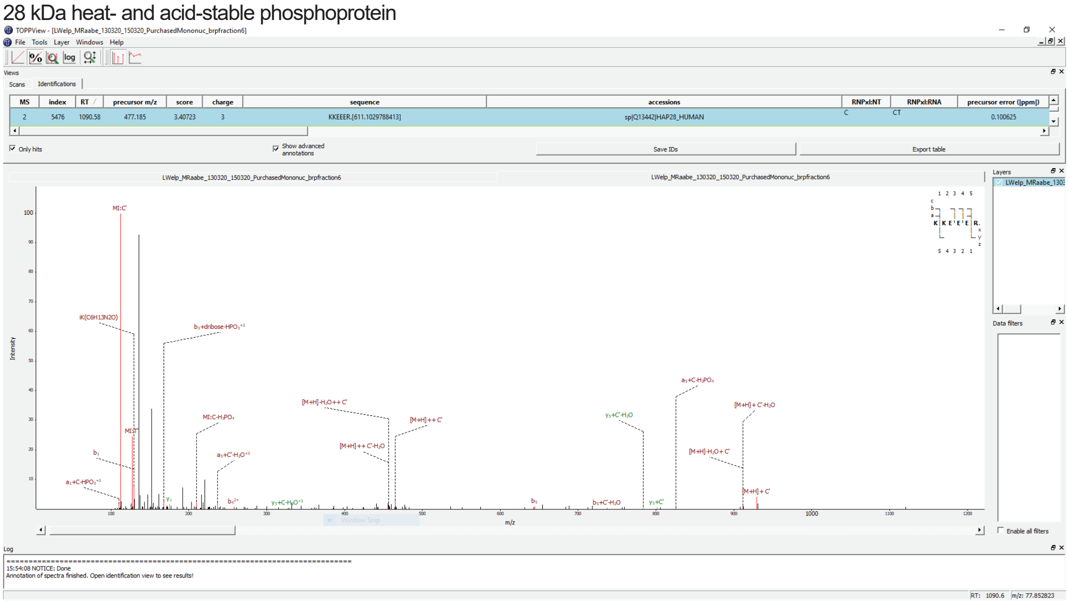


57) Putative RNA-binding protein Luc7-like 2

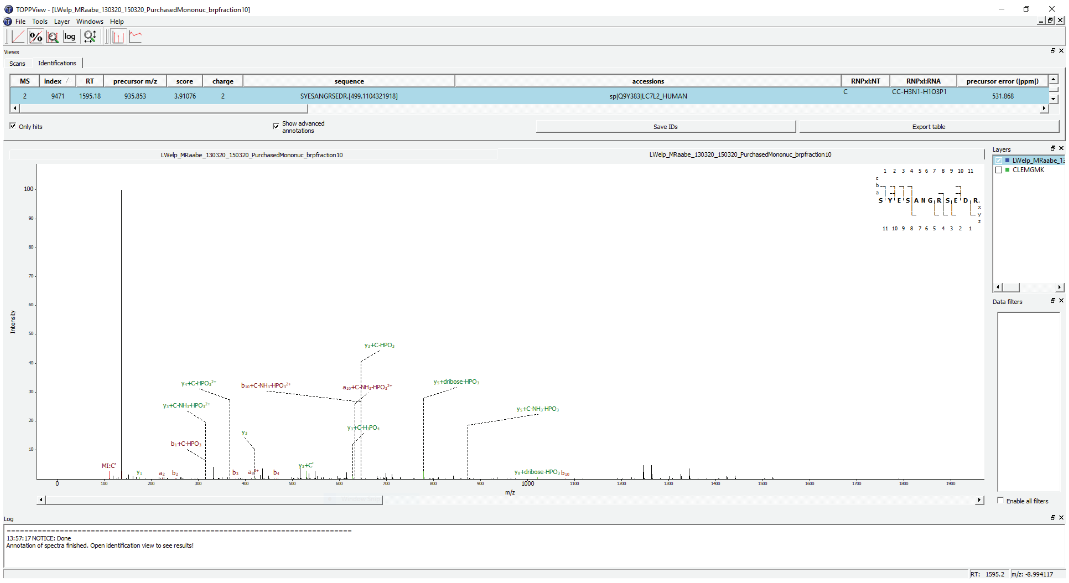

## 58) Protein LSM14 homolog A

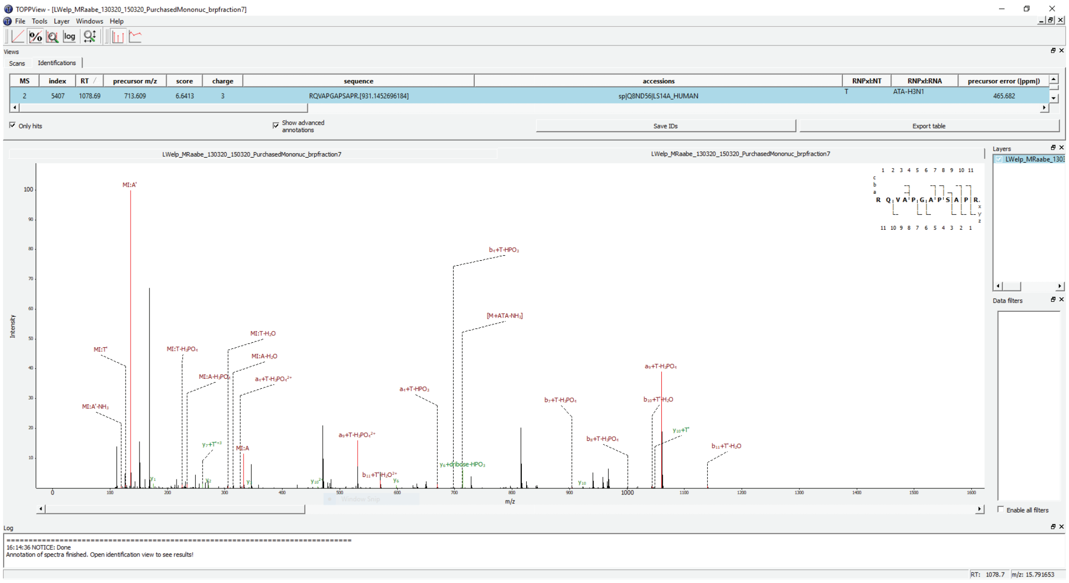


## 59) Nucleolar GTP-binding protein 1

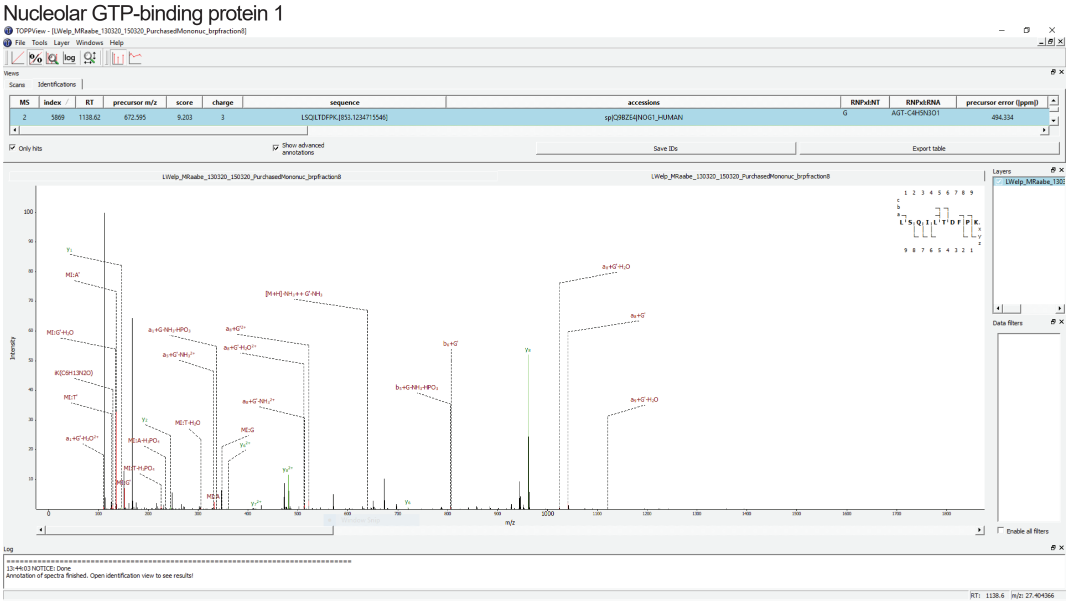


## 60)

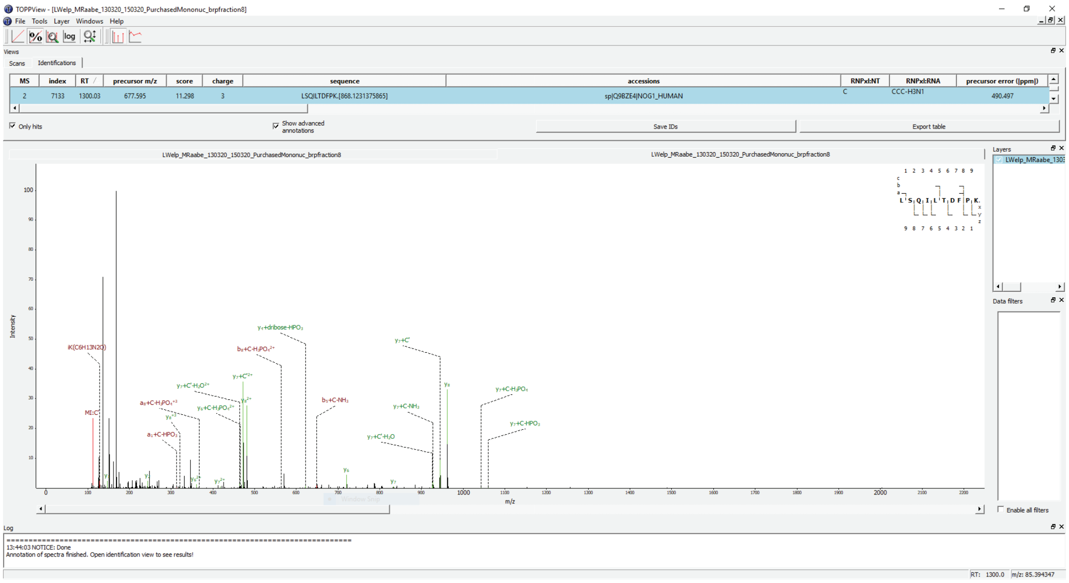

## 61) Polypyrimidine tract-binding protein 1

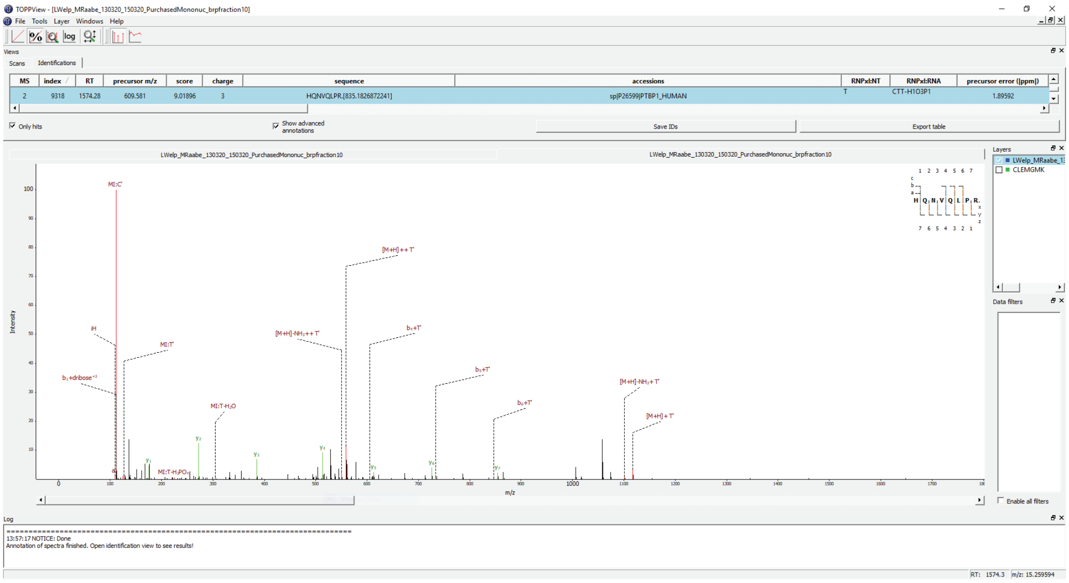


## 62) Receptor of activated protein C kinase 1

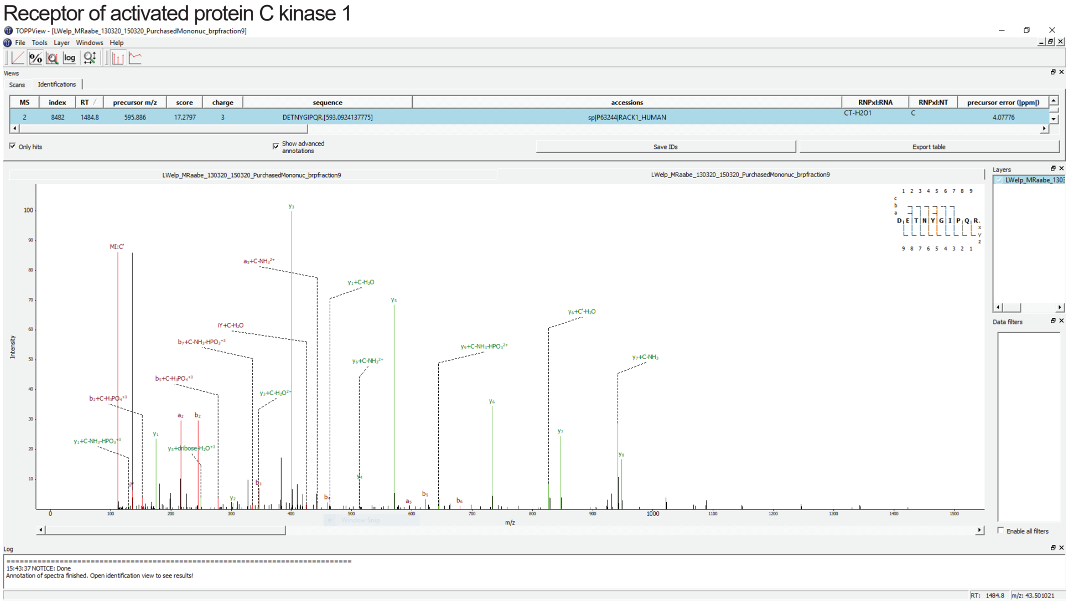


## 63) ELKS/Rab6-interacting/CAST family member 1

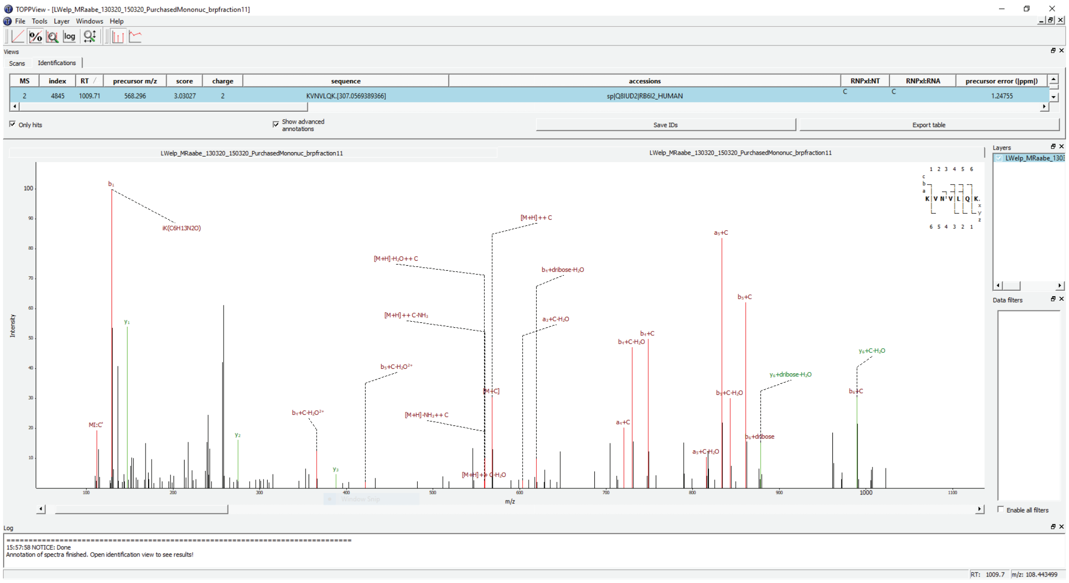

## 64) 60S ribosomal protein L30

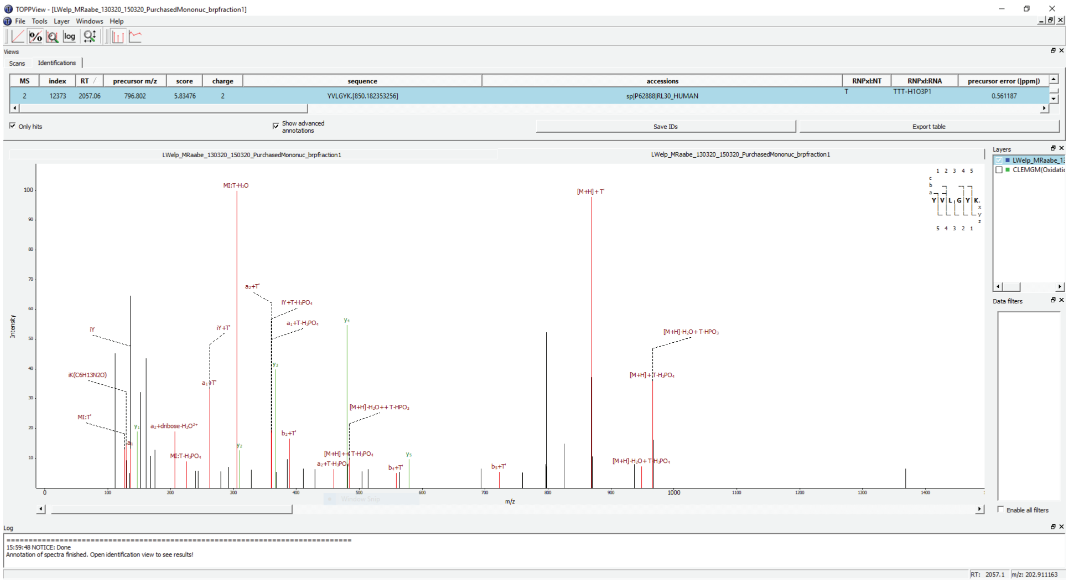


## 65) 40S ribosomal protein S28

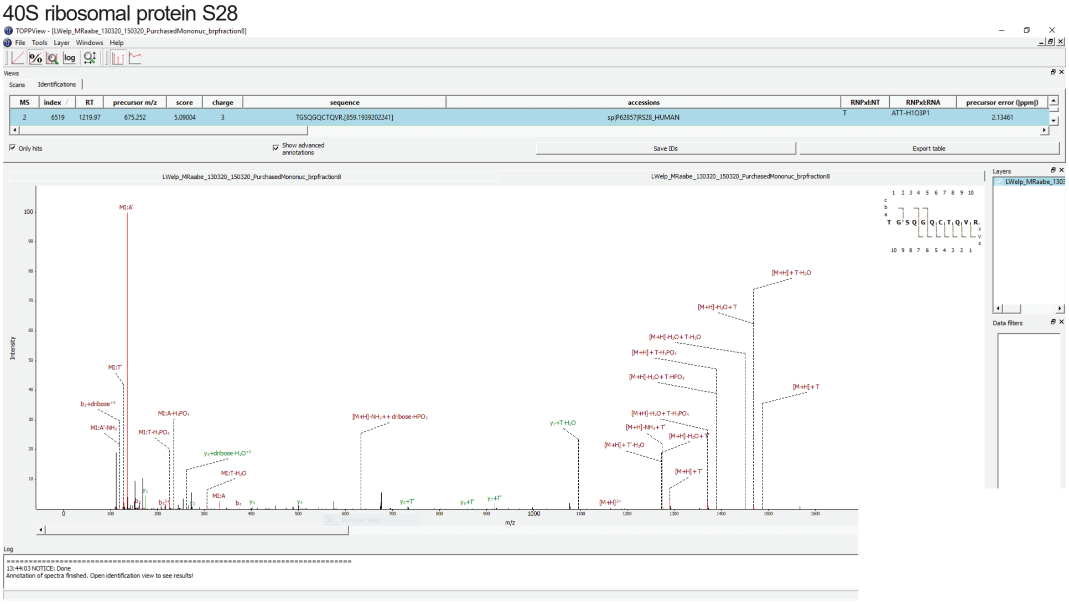

Supplement: Supplementary file 8 — Supplementary Data 6 [file 41467_2020_19047_MOESM8_ESM.pdf]
