# Supplementary material for: Analysis of protein-DNA interactions in chromatin by UV induced cross-linking and mass spectrometry
Source: Nat Commun. 2020 Oct 16;11:5250. doi: 10.1038/s41467-020-19047-7 (PMC7567871; doi:10.1038/s41467-020-19047-7)

Supplementary Data 8

Analysis of protein-DNA interactions in chromatin by UV induced cross-linking and mass spectrometry

Stützer *et al.*

List of contents

UV cross-linked HeLa nuclei (chromatin precipitation)
RNP^{xl}search_settings#1

TOPPView spectra – **unambiguous** cross-link spectrum matches p. 2-12
TOPPView spectra – **ambiguous** cross-link spectrum matches p. 13-16

UV cross-linked HeLa nuclei (chromatin precipitation)
RNP^{xl}search_settings#2

TOPPView spectra – **unambiguous** cross-link spectrum matches p. 17-24
TOPPView spectra – **ambiguous** cross-link spectrum matches p. 25-28

UV cross-linked HeLa nuclei (SEC)

TOPPView spectra – **unambiguous** cross-link spectrum matches p. 29-38
TOPPView spectra – **deoxyribose** cross-link spectrum matches p. 39-52
TOPPView spectra – **ambiguous** cross-link spectrum matches p. 53-54

UV cross-linked HeLa nuclei (chromatin precipitation)
RNP^{xl}search_settingsRNA

TOPPView spectra – **unambiguous** cross-link spectrum matches p. 54

TOPPView spectra - unambiguous cross-link spectrum matches

UV cross-linked HeLa nuclei (chromatin precipitation), RNP^{xl}search_settings#1

1) Nucleosome-remodeling factor subunit BPTF

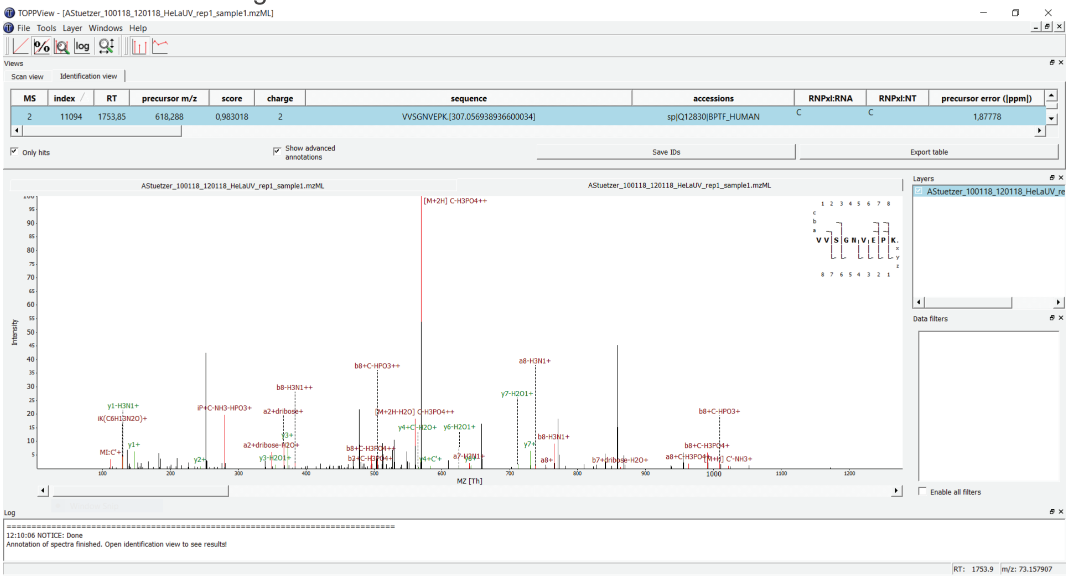

2)

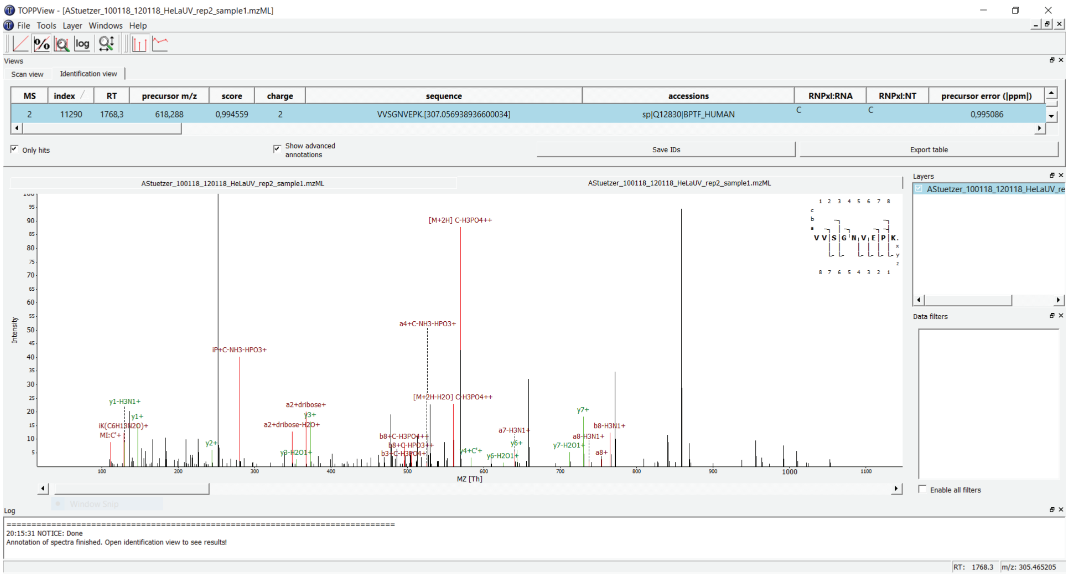

3)

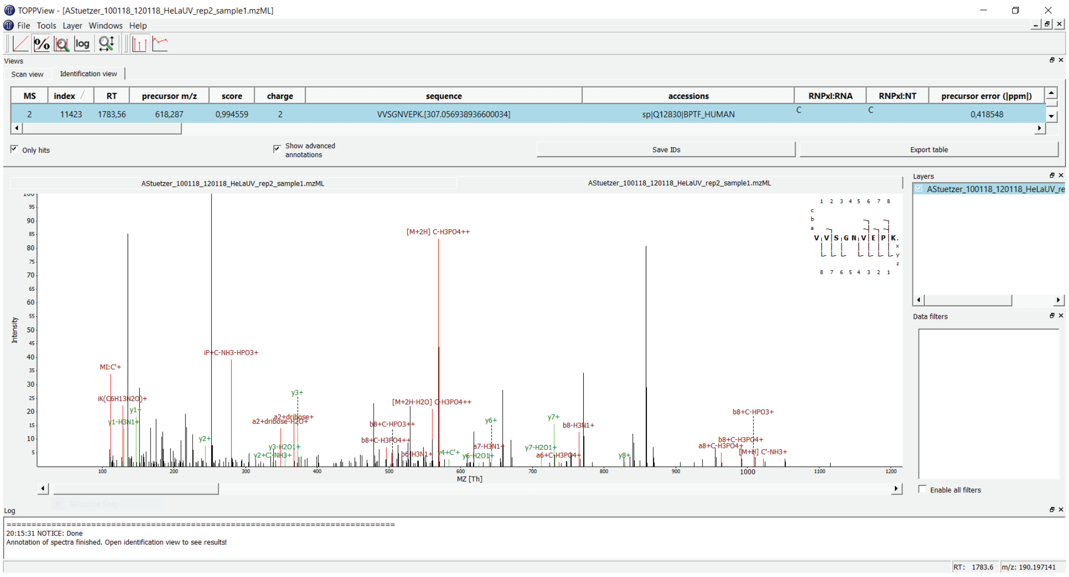

4) E3 ubiquitin-protein ligase BRE1A

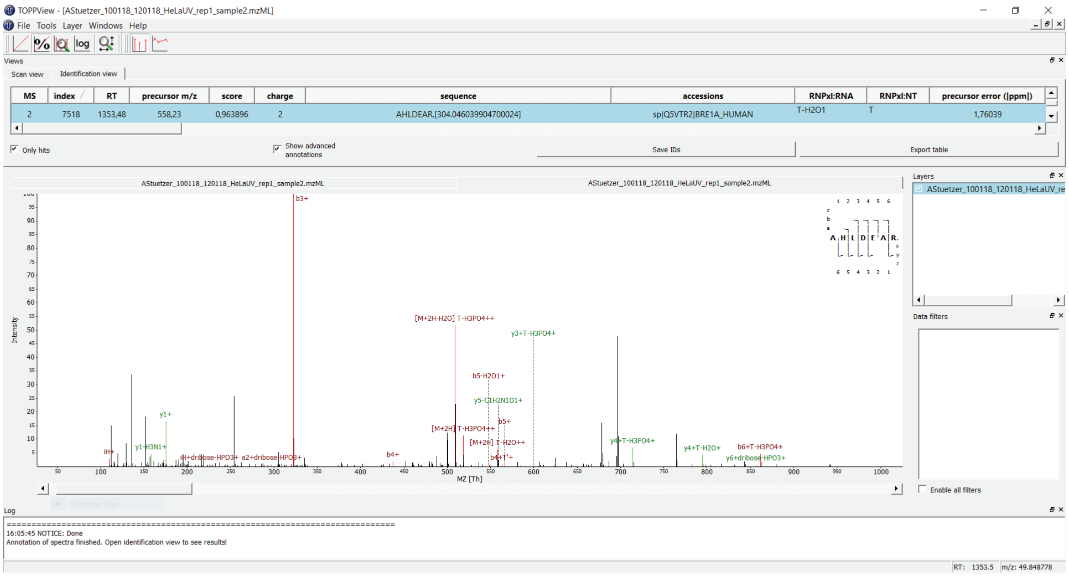

5) E3 ubiquitin-protein ligase BRE1B

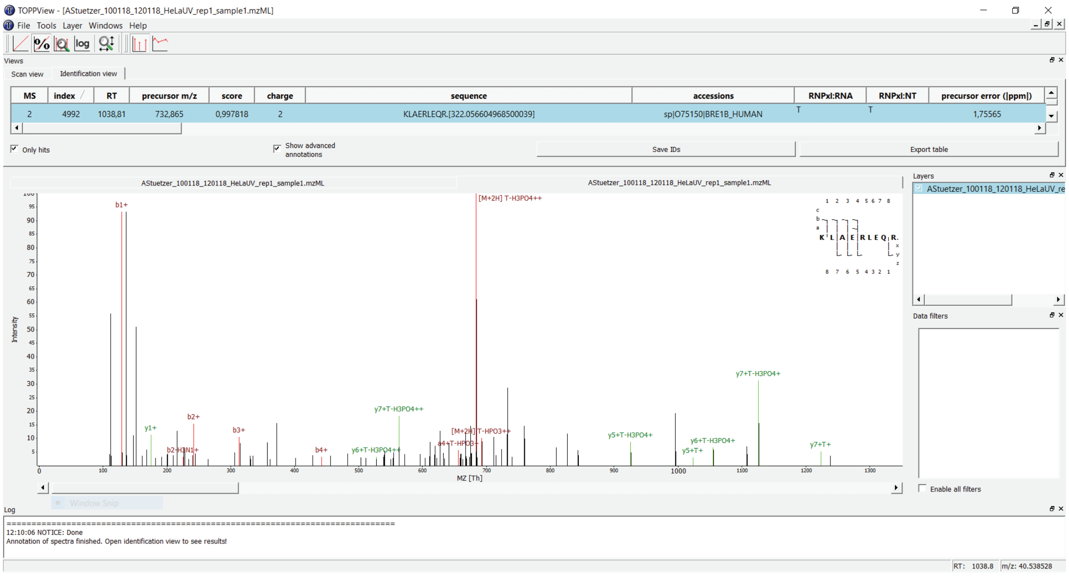

6) Centrosome-associated protein 350

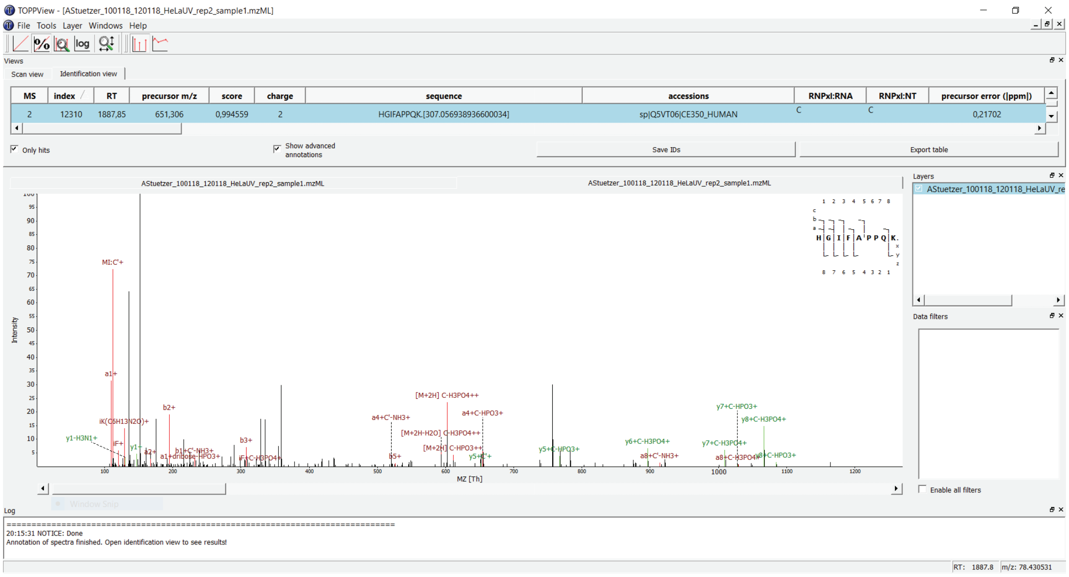

7) CCAAT/enhancer-binding protein epsilon

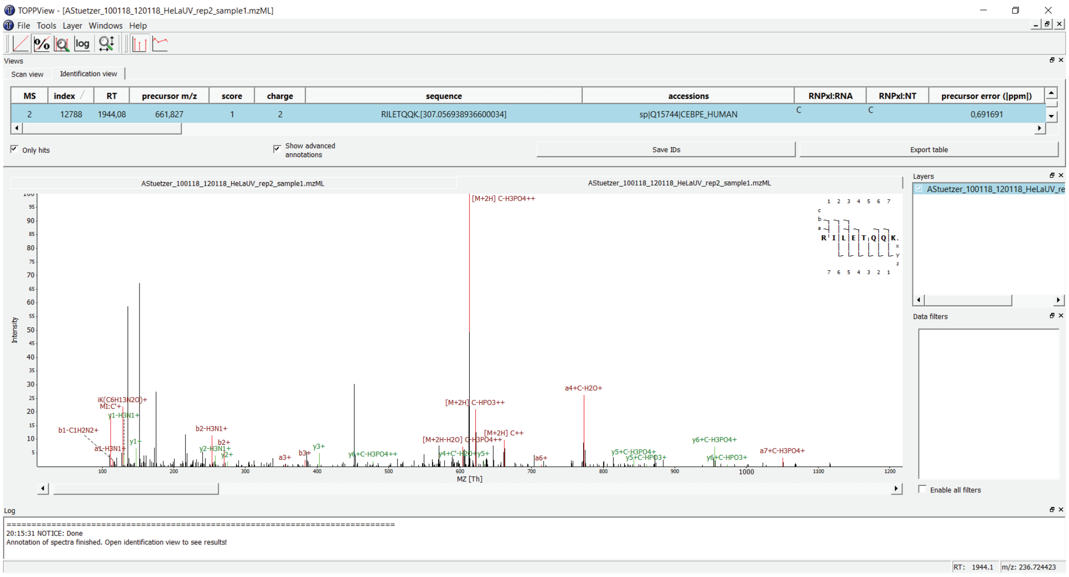

8) Centrosomal protein of 85 kDa

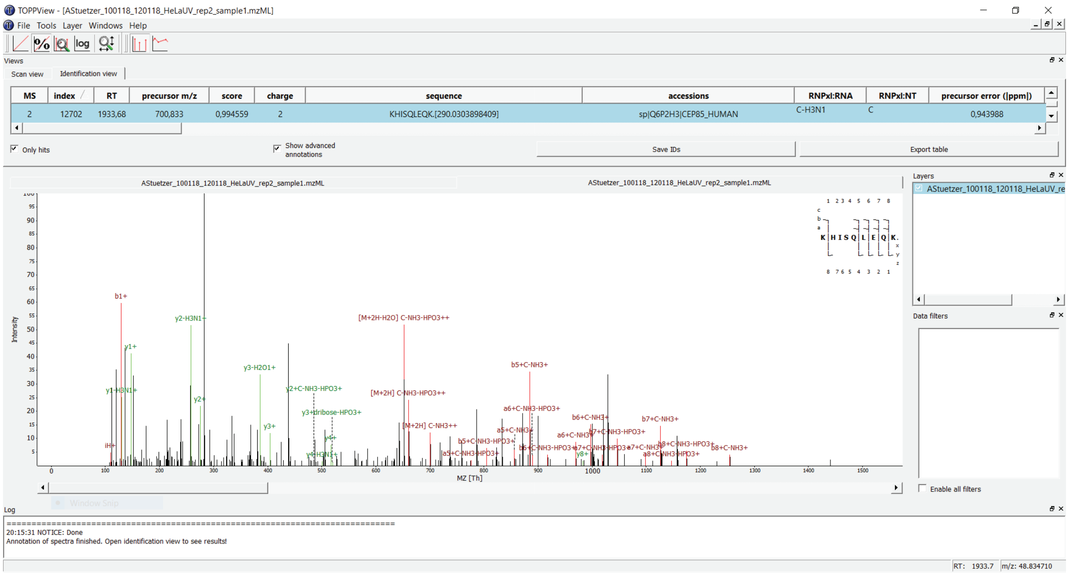

9) Transcription factor COE2

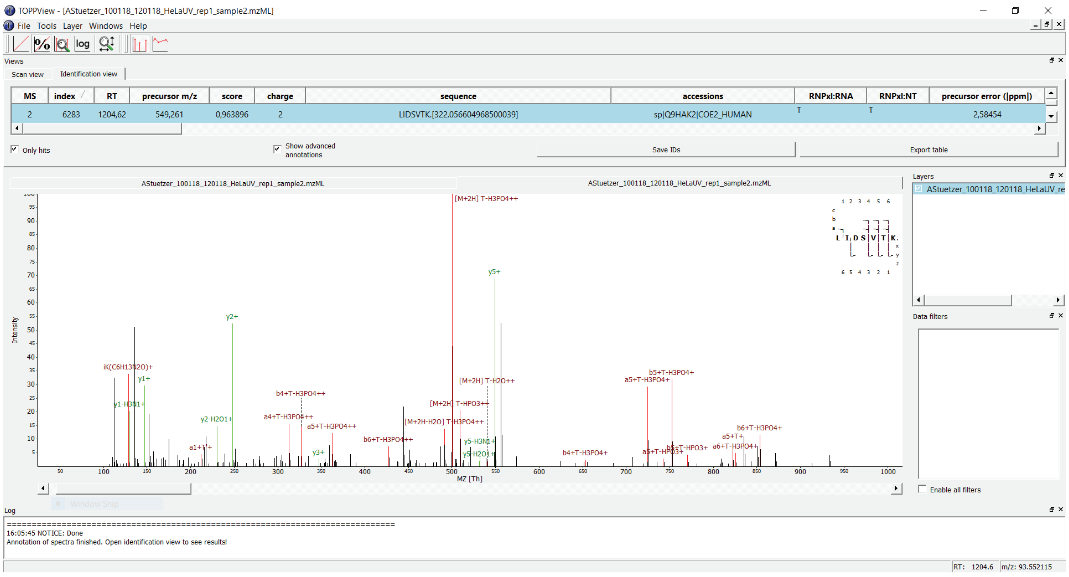

10)

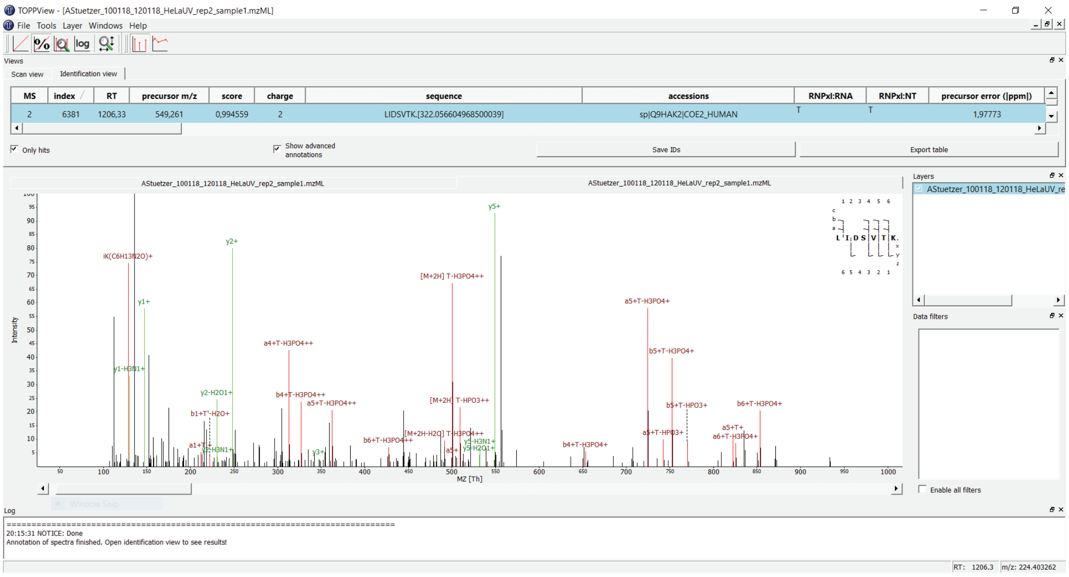

11) DNA damage-binding protein 1

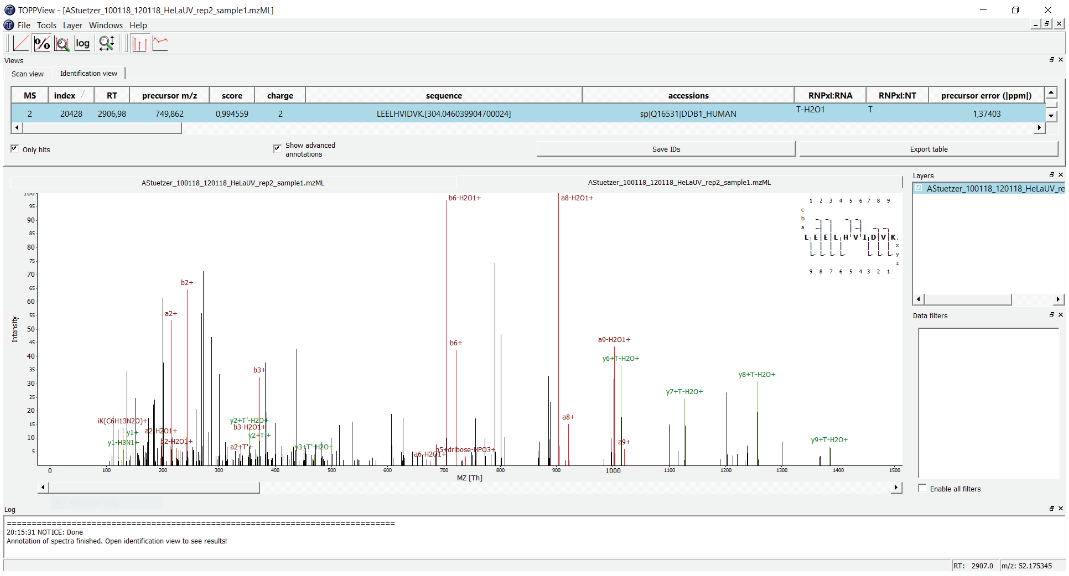

12) Histone H3

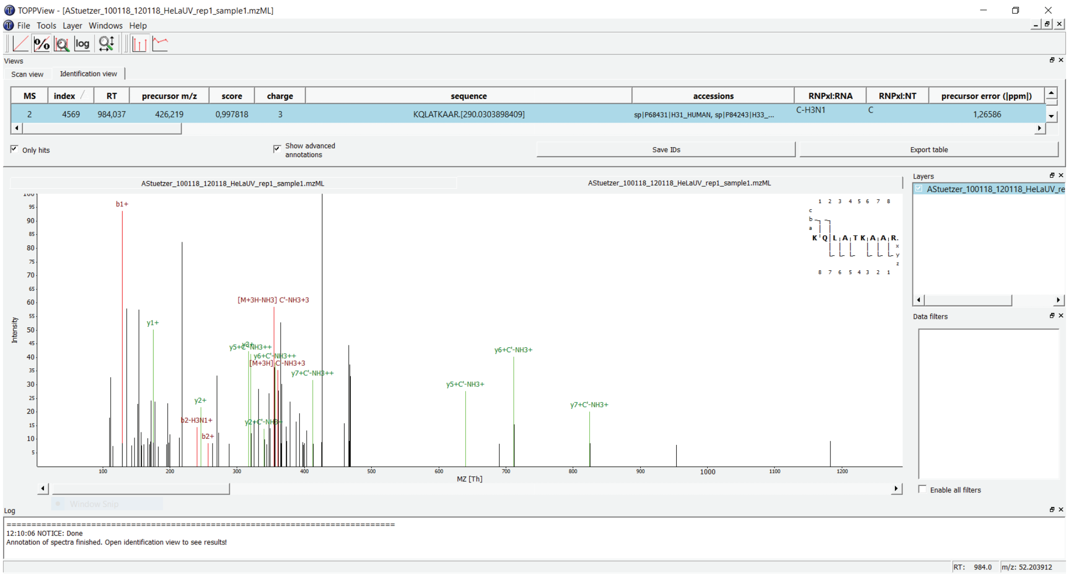

13)

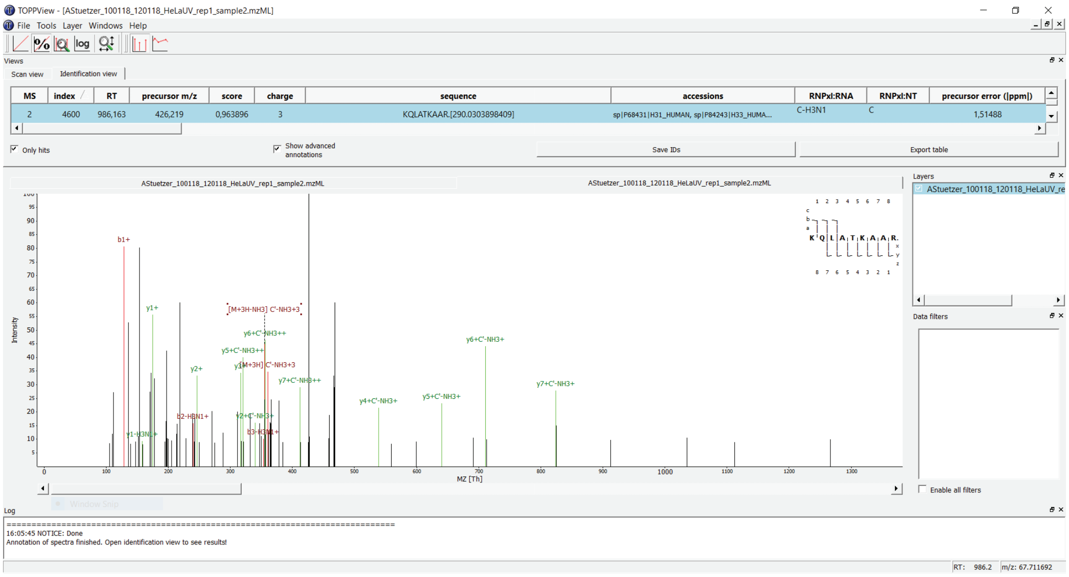

14) Hepatic leukemia factor

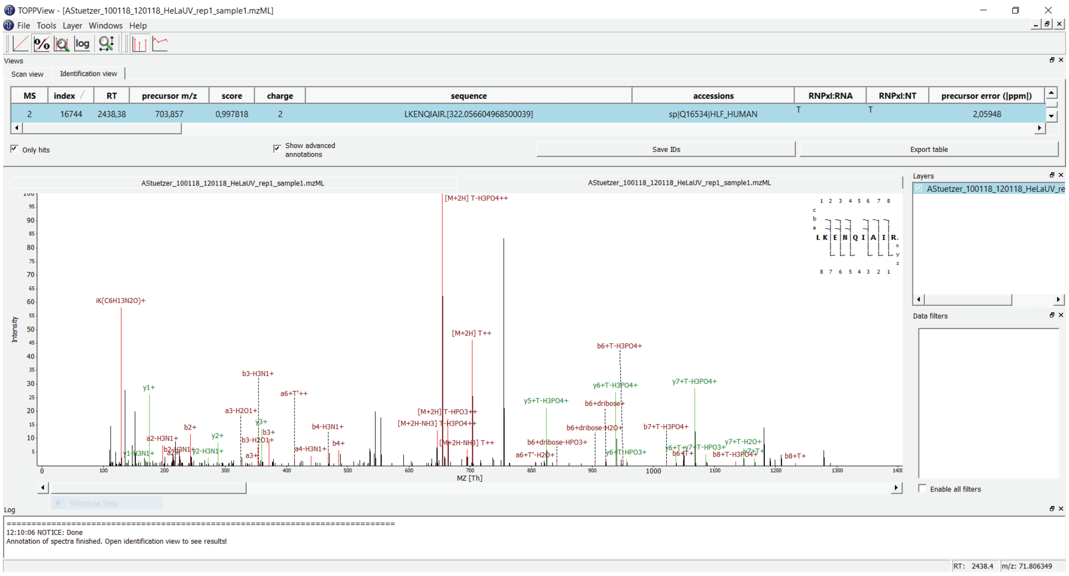

15)

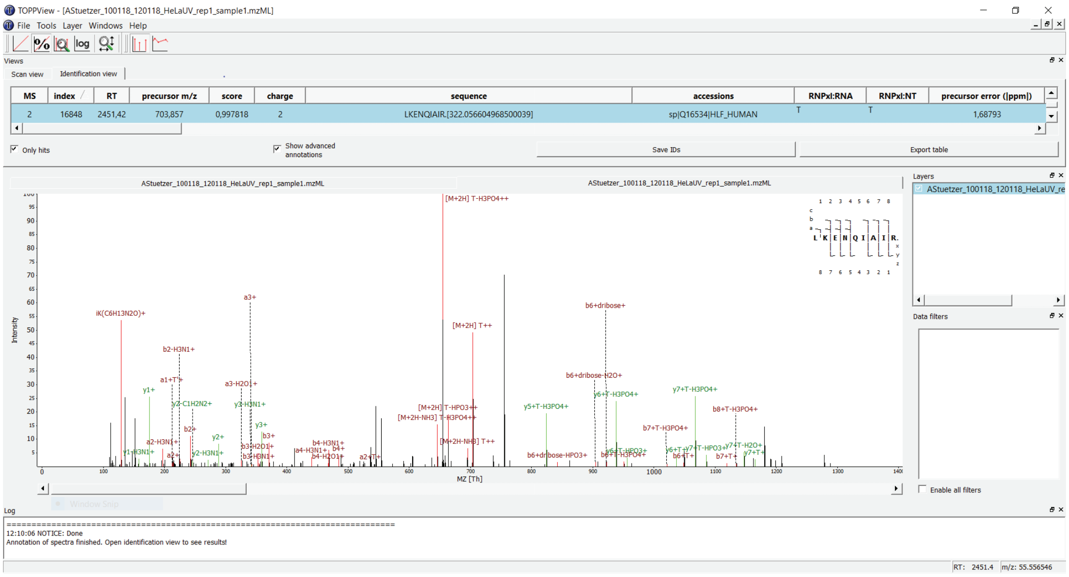

16) Zinc finger protein Pegasus

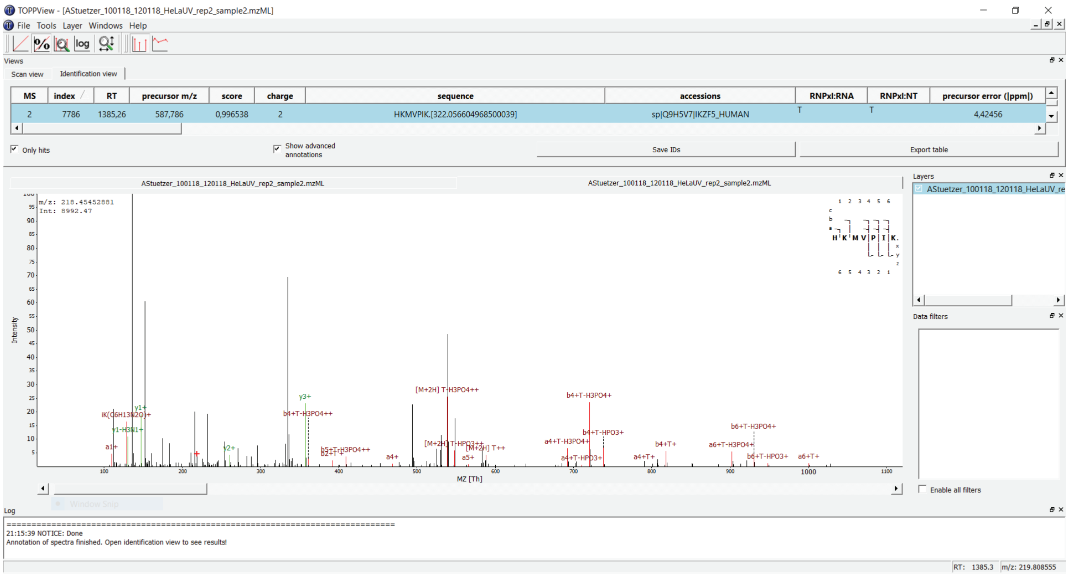

17) Phosphatidate phosphatase LPIN3

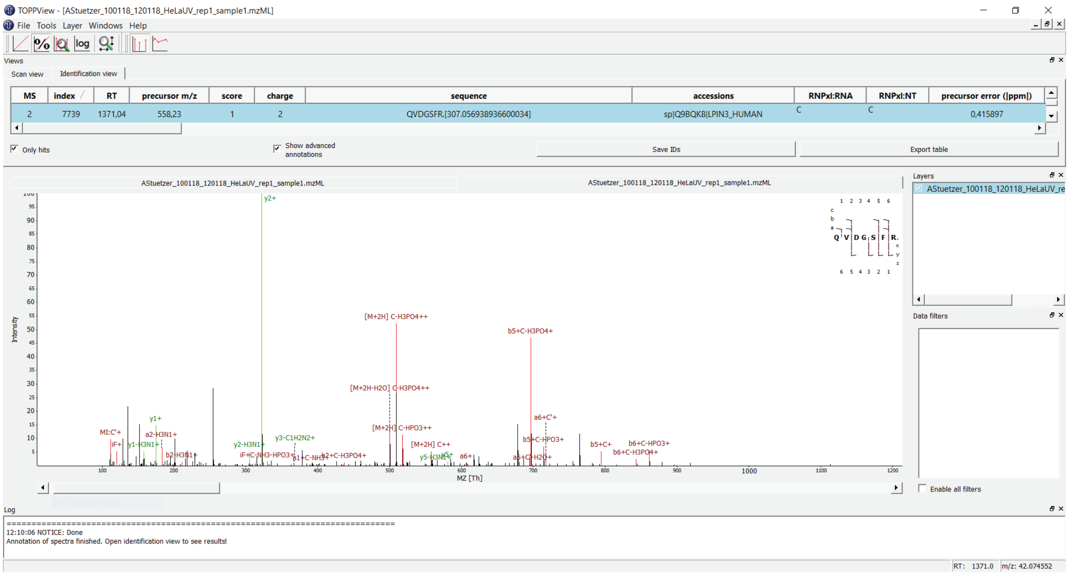

18)

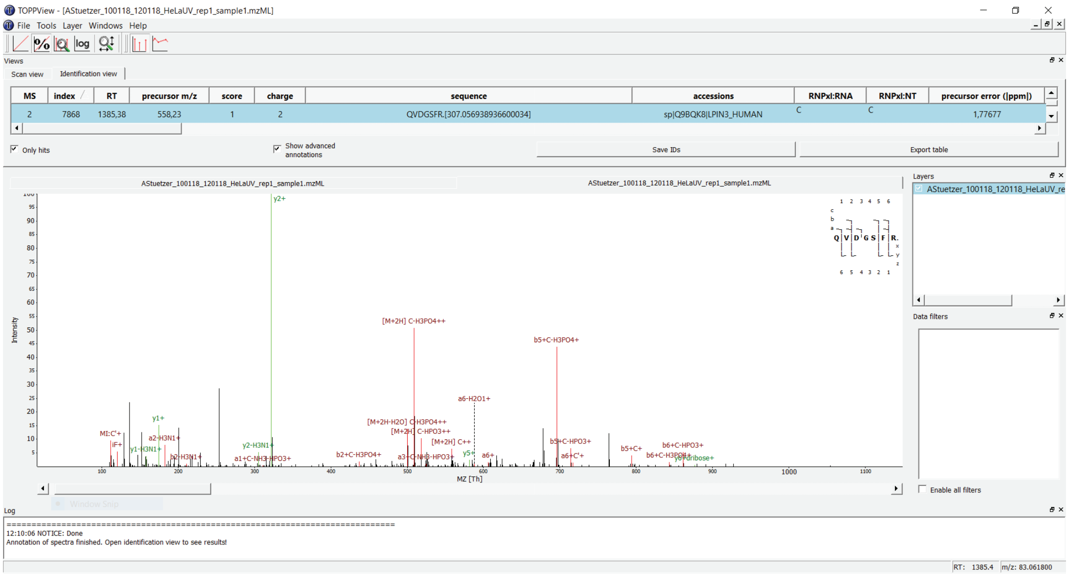

19)

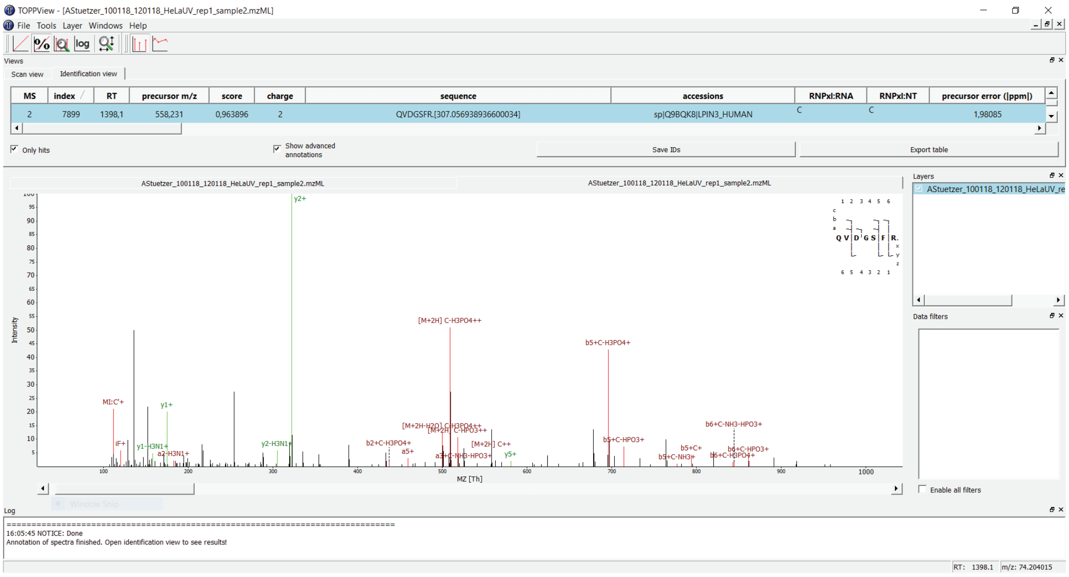

20) Transcriptional repressor p66-alpha

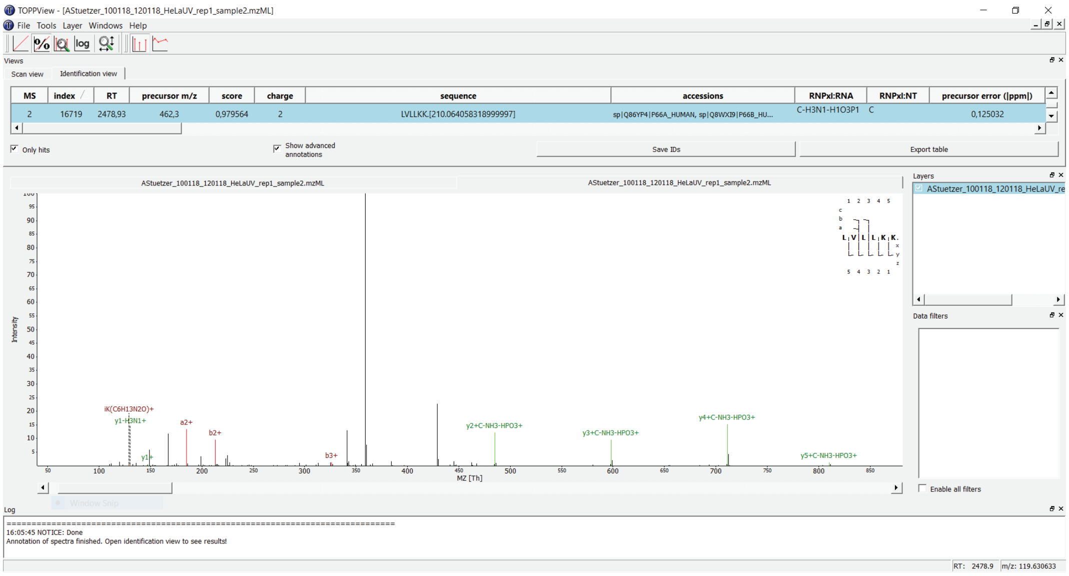

21) Programmed cell death protein 7

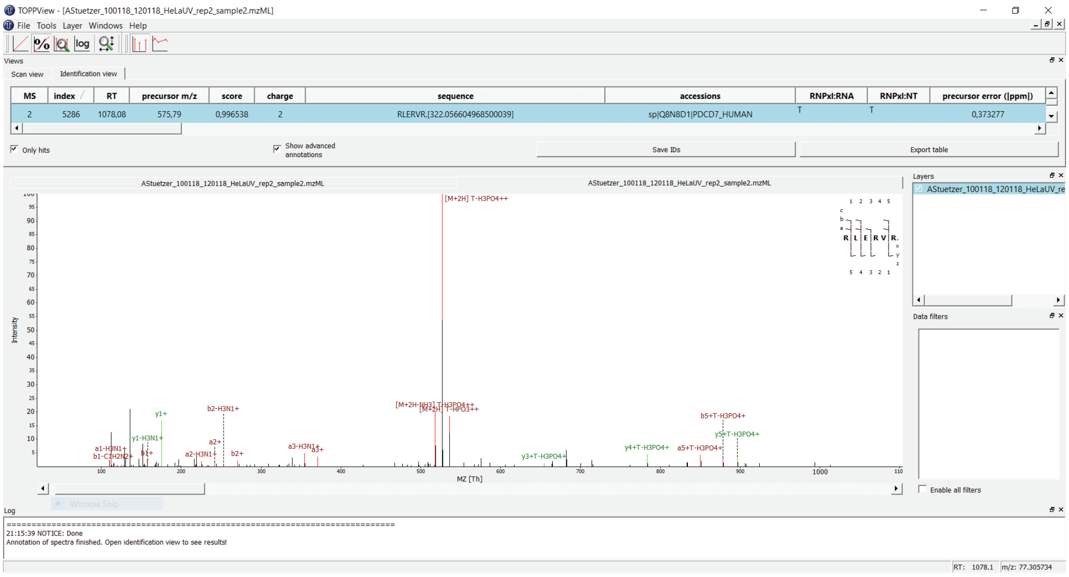

22) PDZ and LIM domain protein 2

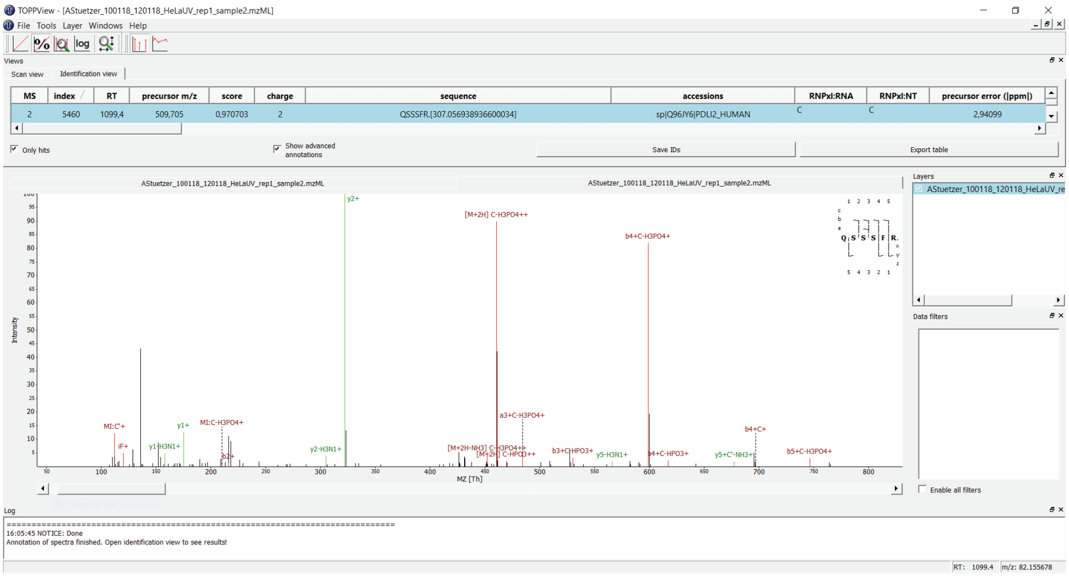

23) PHD finger protein 19

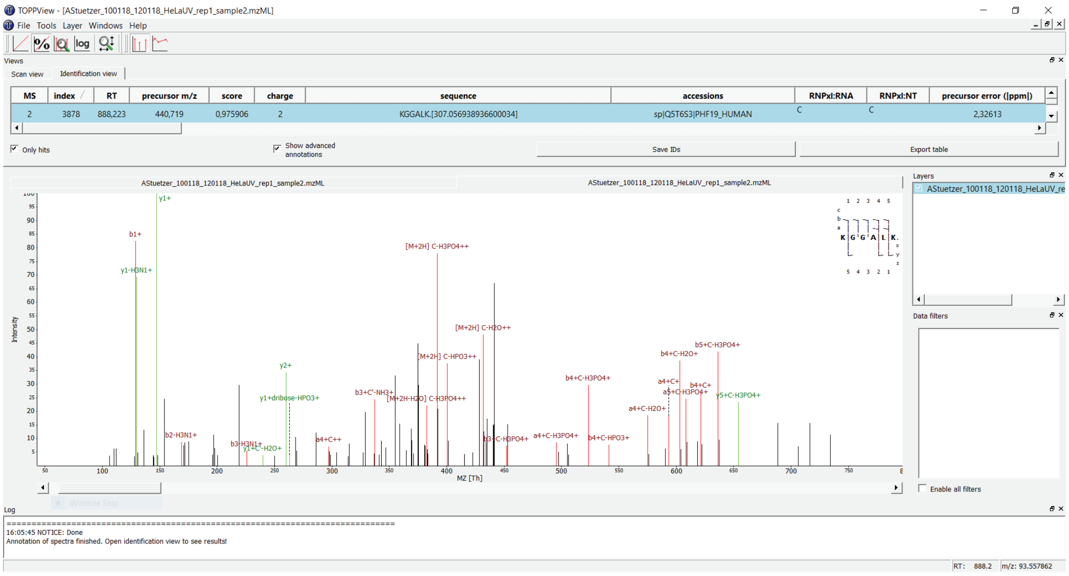

24) PR domain zinc finger protein 5

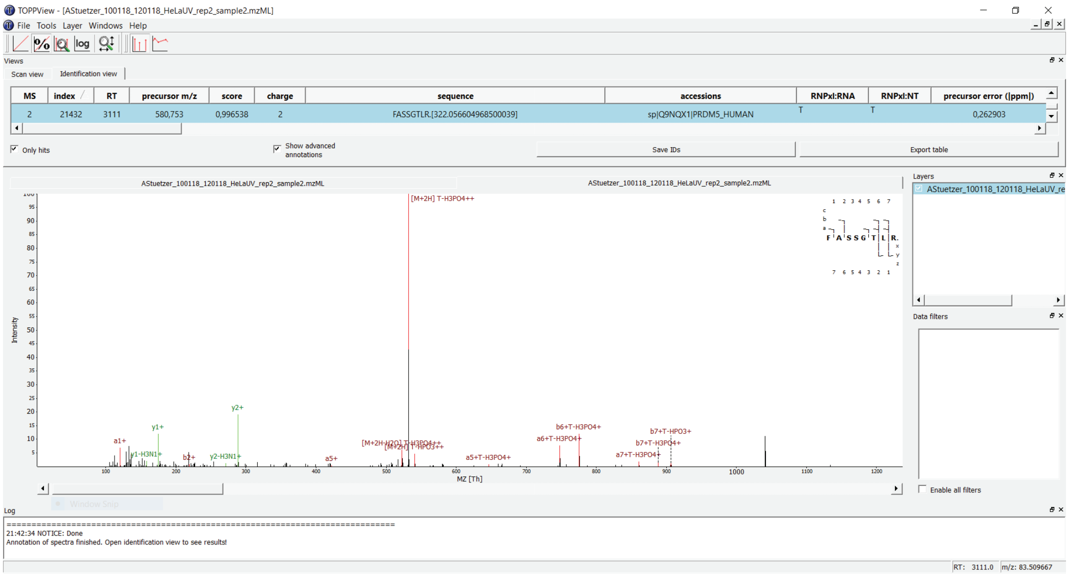

25) Gelsolin

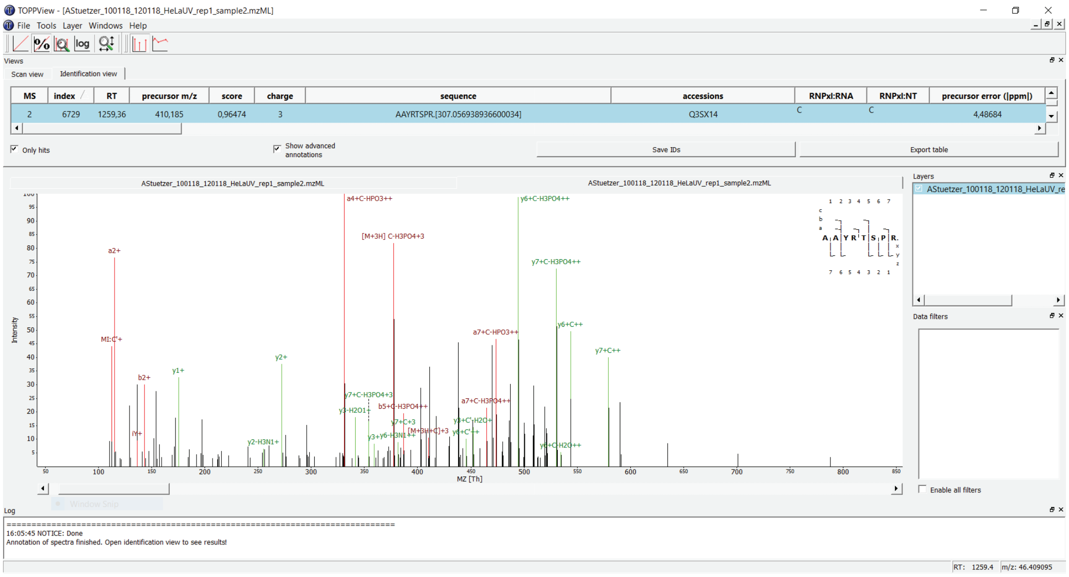

26)

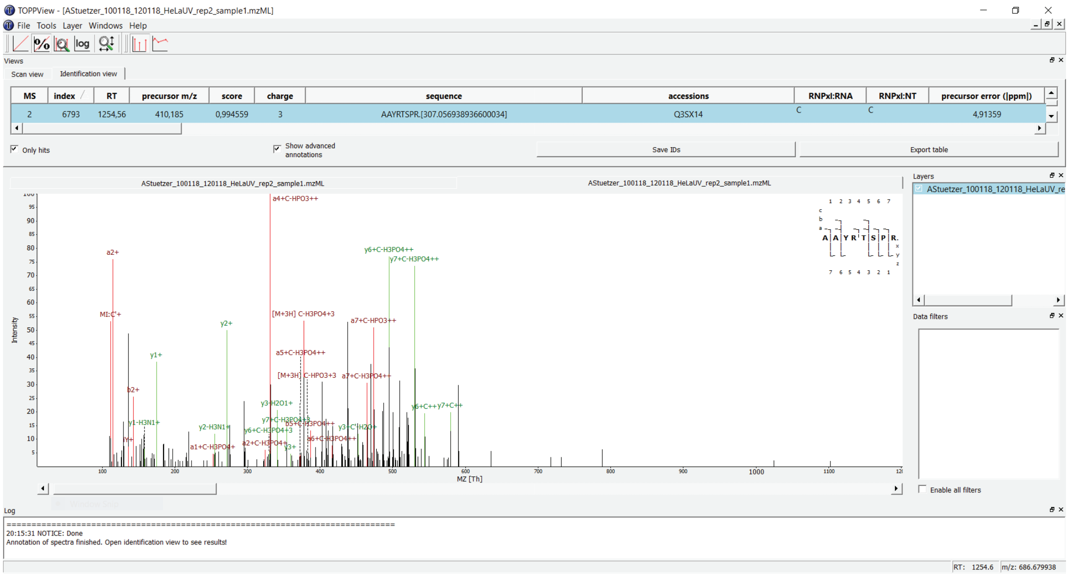

27) DNA-binding protein SMUBP-2

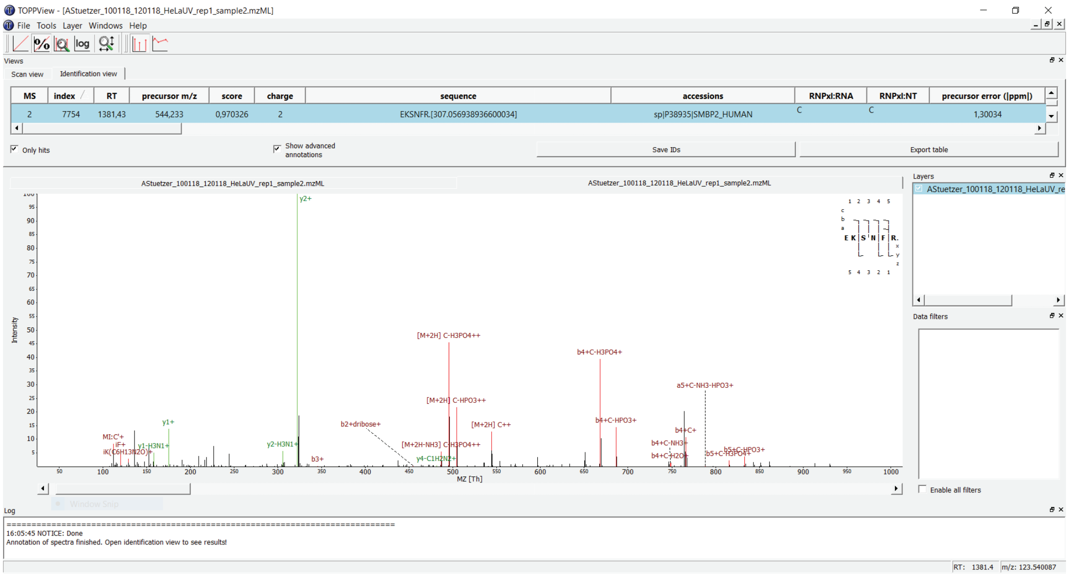

28) General transcription factor IIF subunit 1

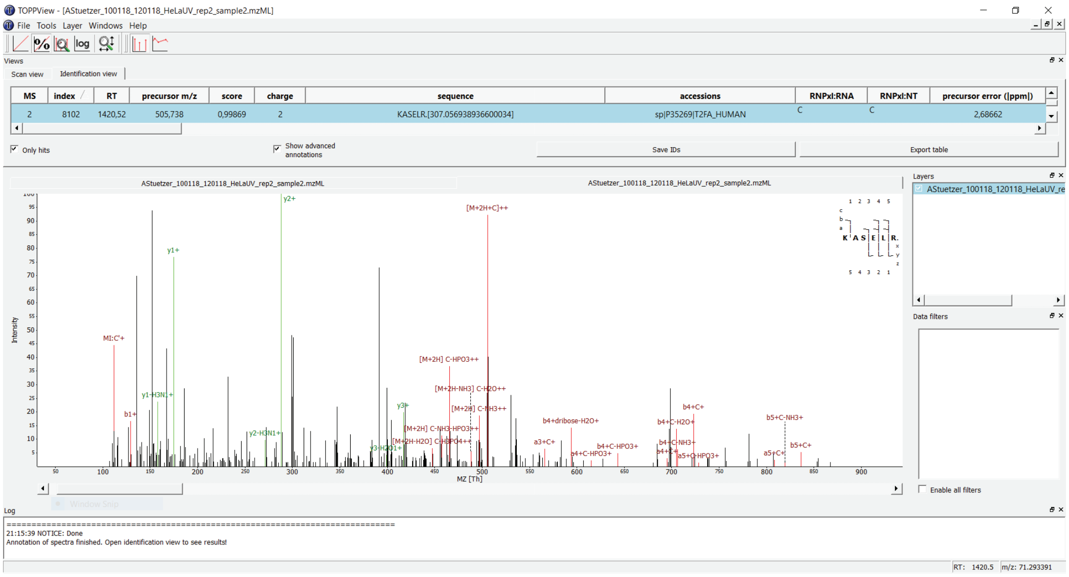

29) Pre-rRNA-processing protein TSR1 homolog

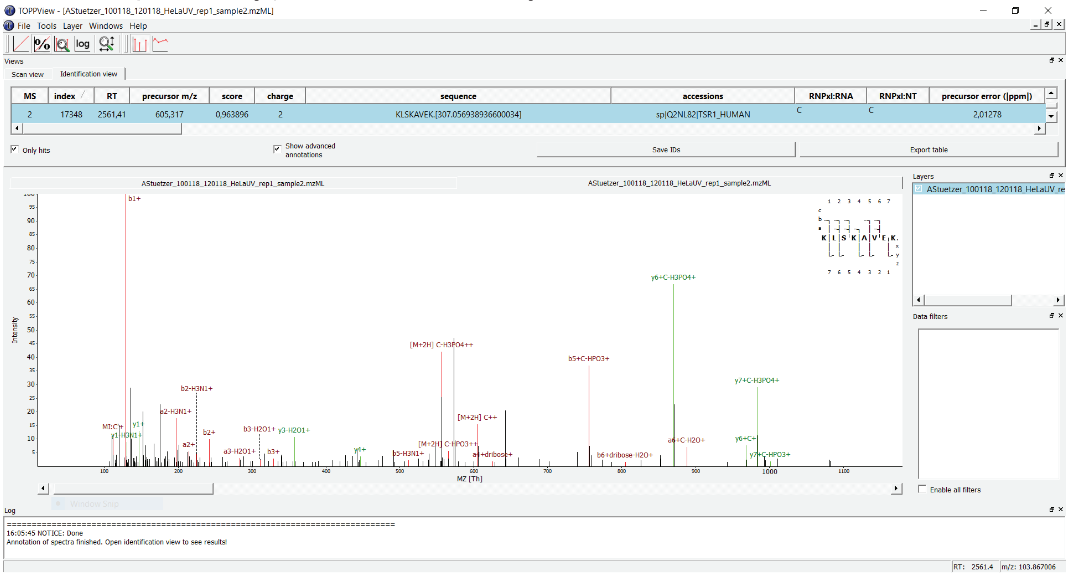

1.30) UBX domain-containing protein 2B

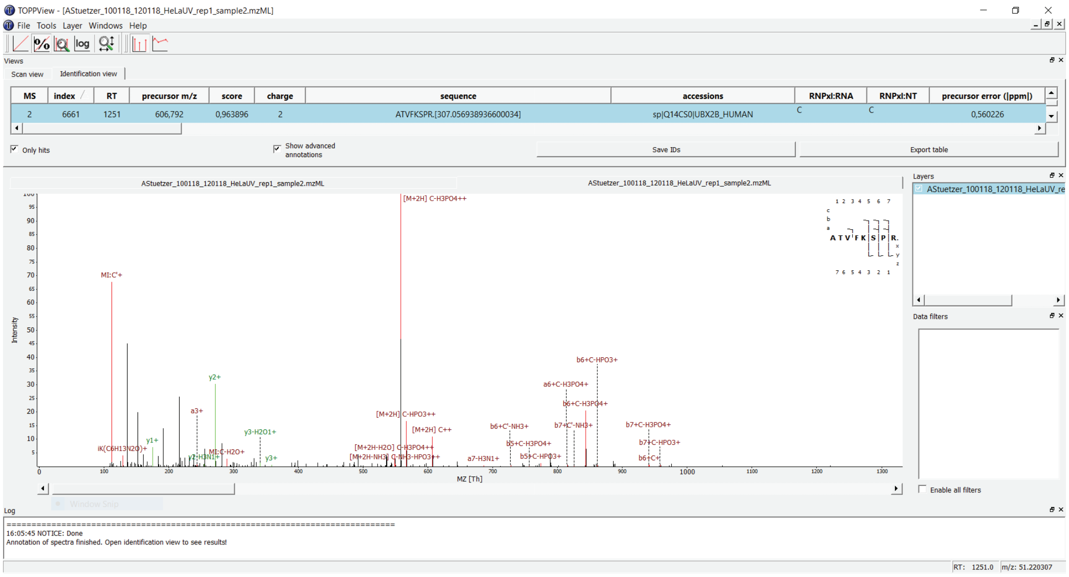

31) Serine/threonine-protein kinase VRK1

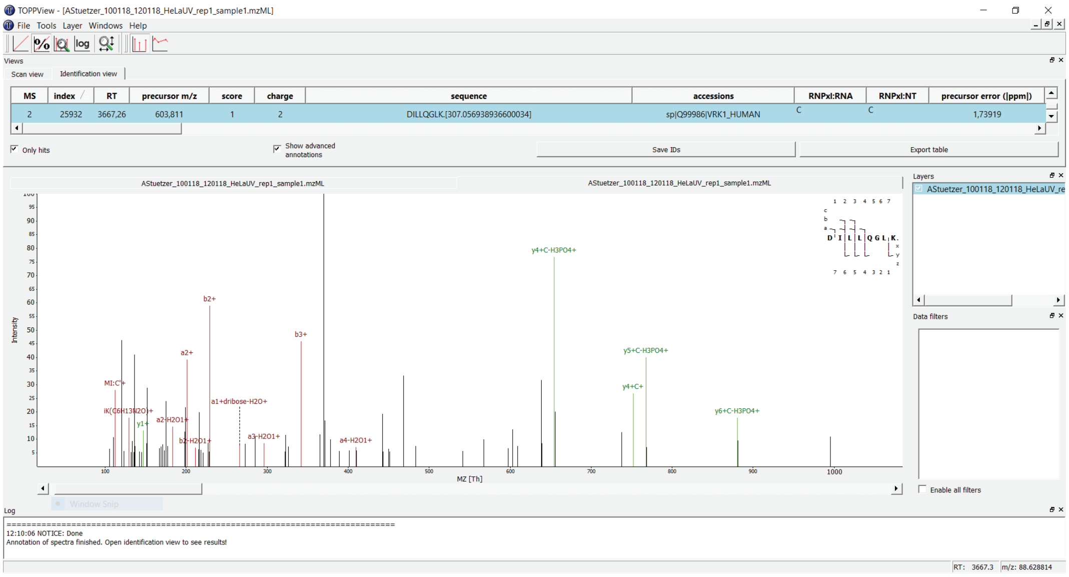

1.32) Zinc finger protein 333

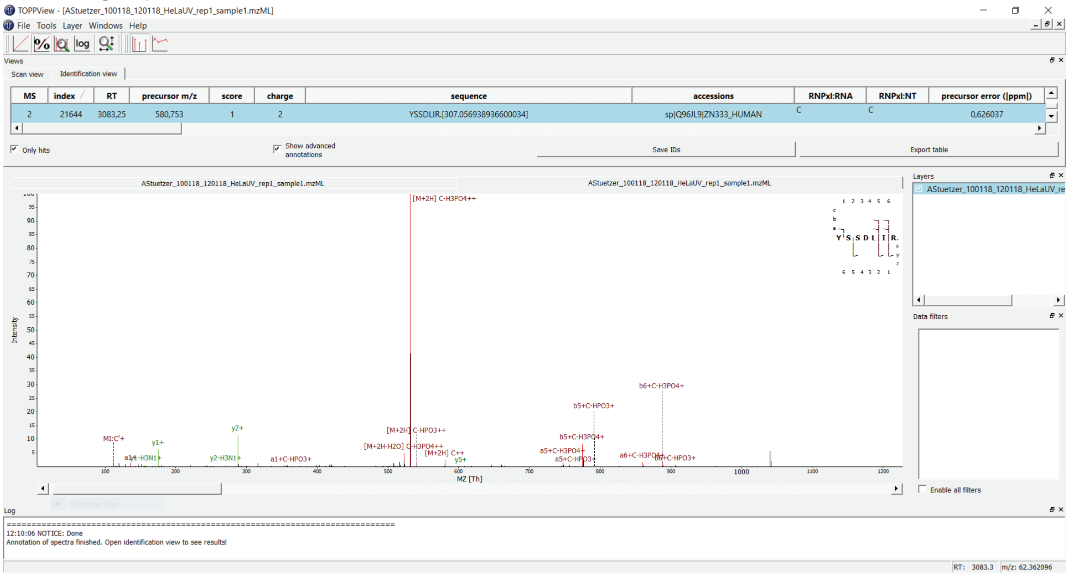

33) Zinc finger protein 469

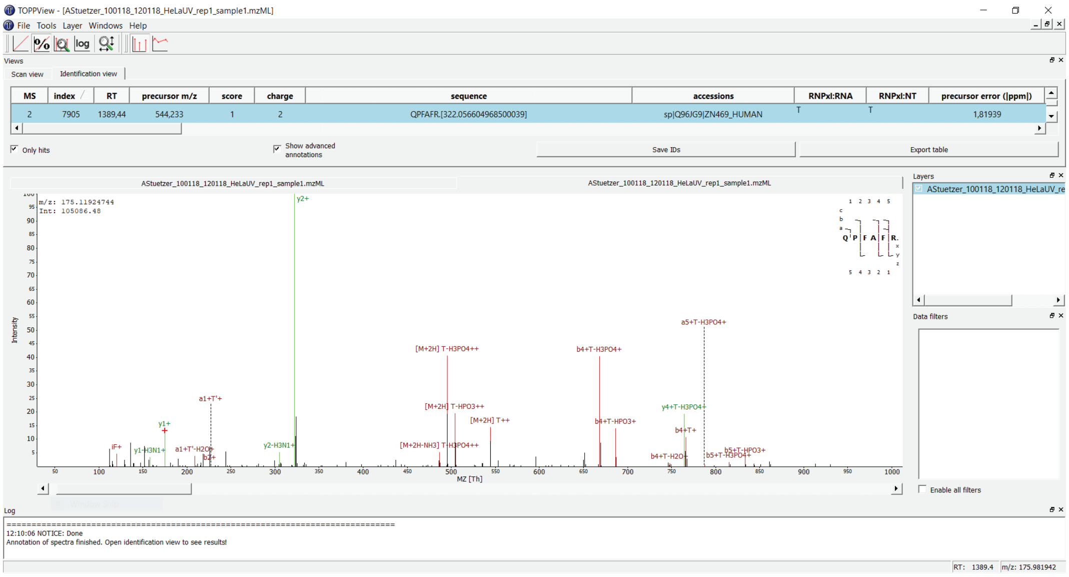

34) Zinc finger protein 549

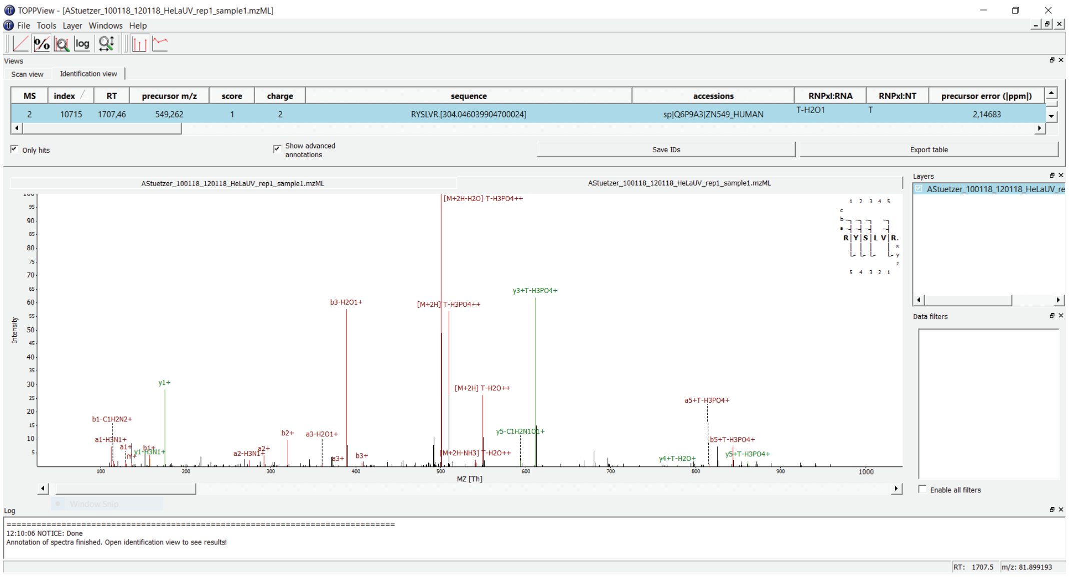

35)

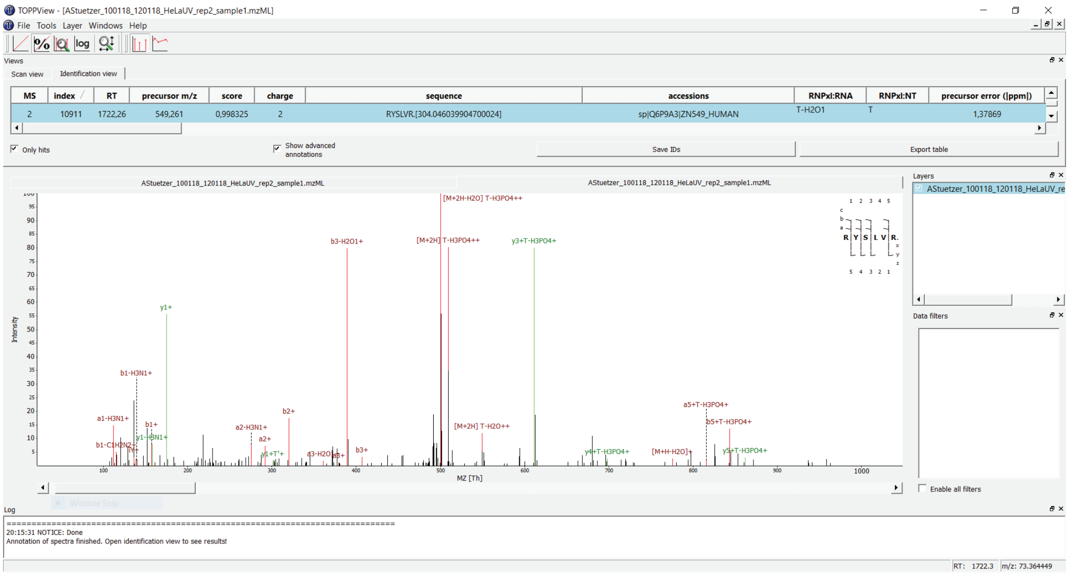

36) Zinc finger protein 888

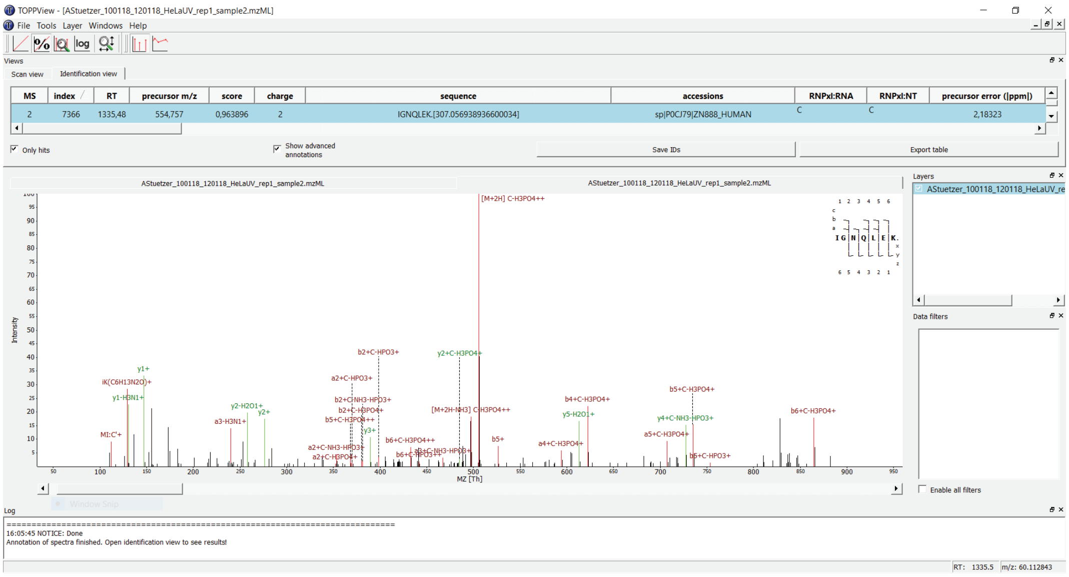

TOPPView spectra - ambiguous cross-link spectrum matches

UV cross-linked HeLa nuclei (chromatin precipitation), RNP[xl]search_settings#1

1.37)

1) A-kinase anchor protein 17A

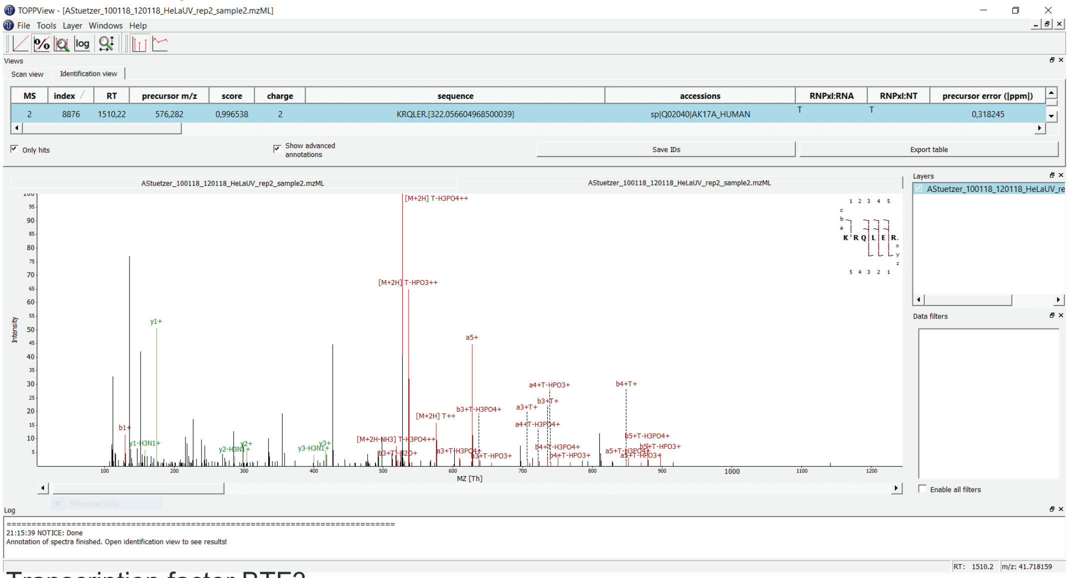

2) Transcription factor BTF3

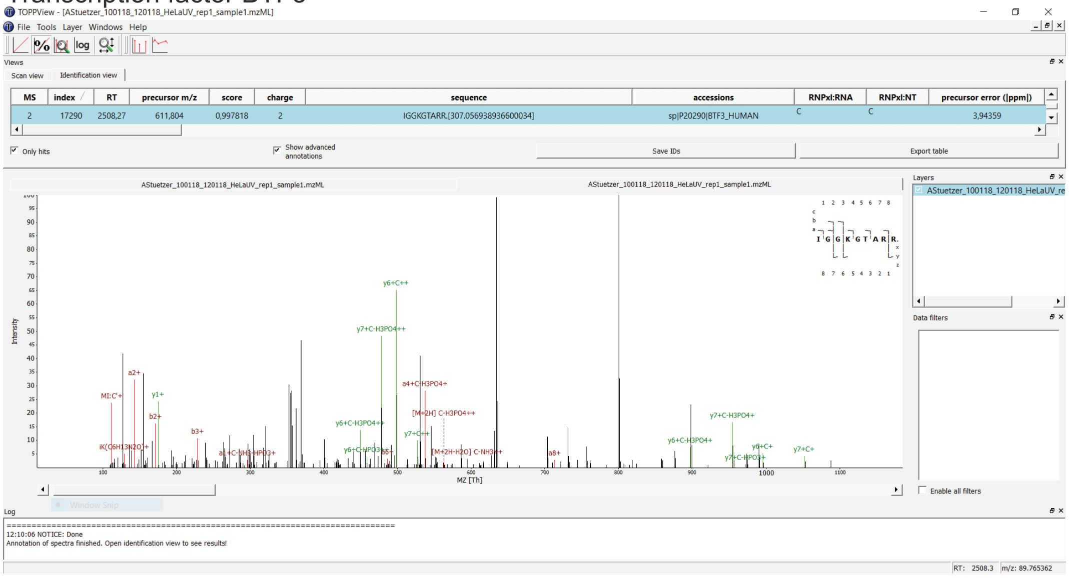

3) Cell death activator CIDE-A

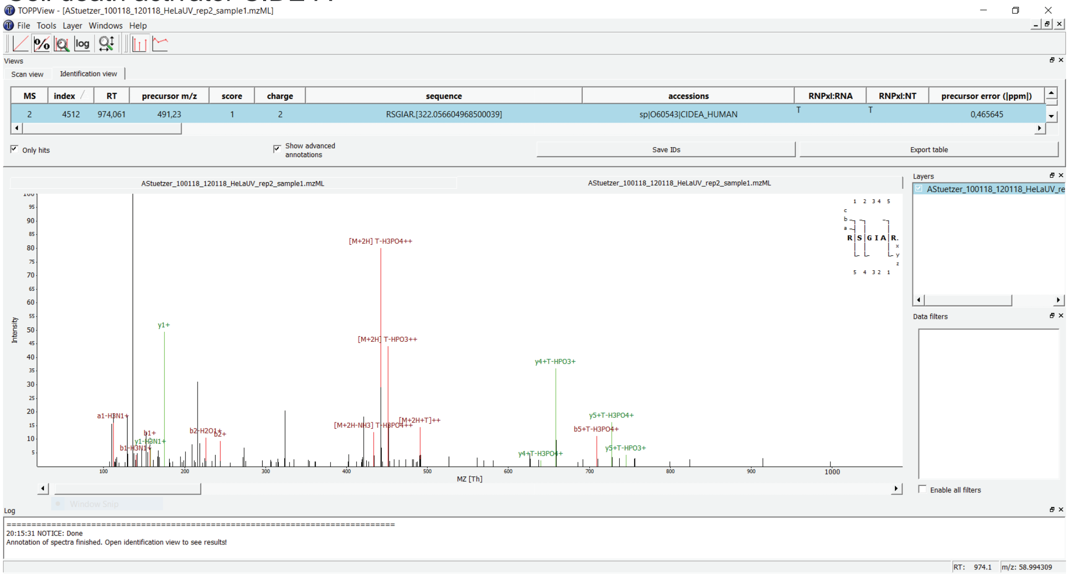

4) DNA polymerase alpha subunit B

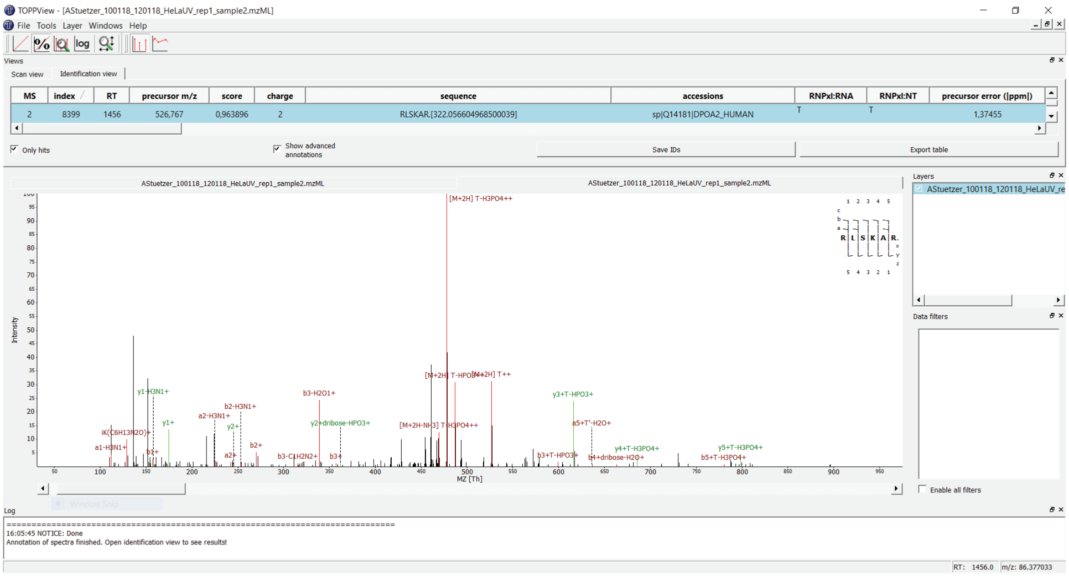

5) E1A-binding protein p400

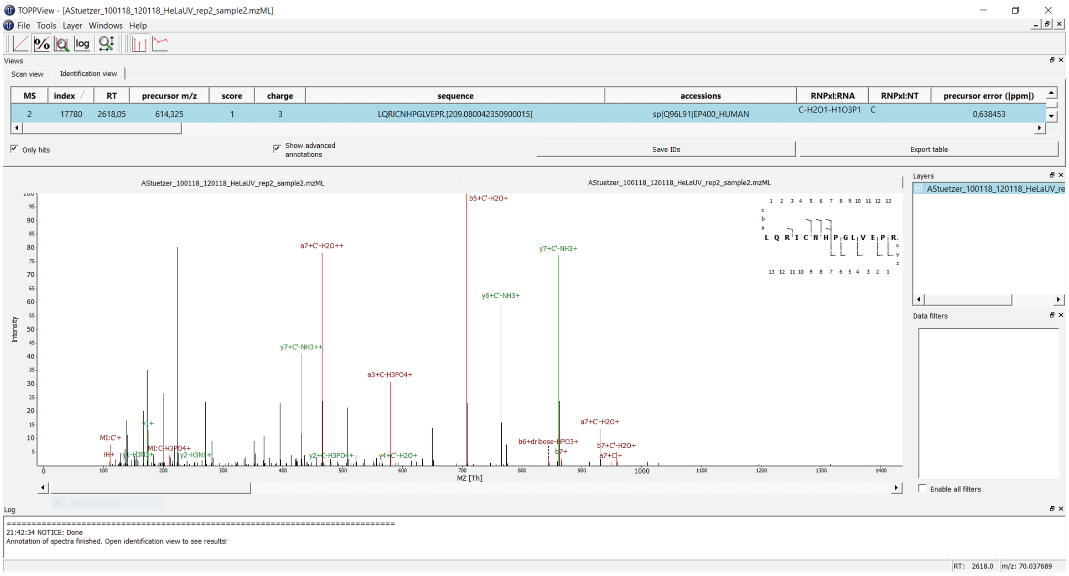

6) Uveal autoantigen with coiled-coil domains and ankyrin repeats

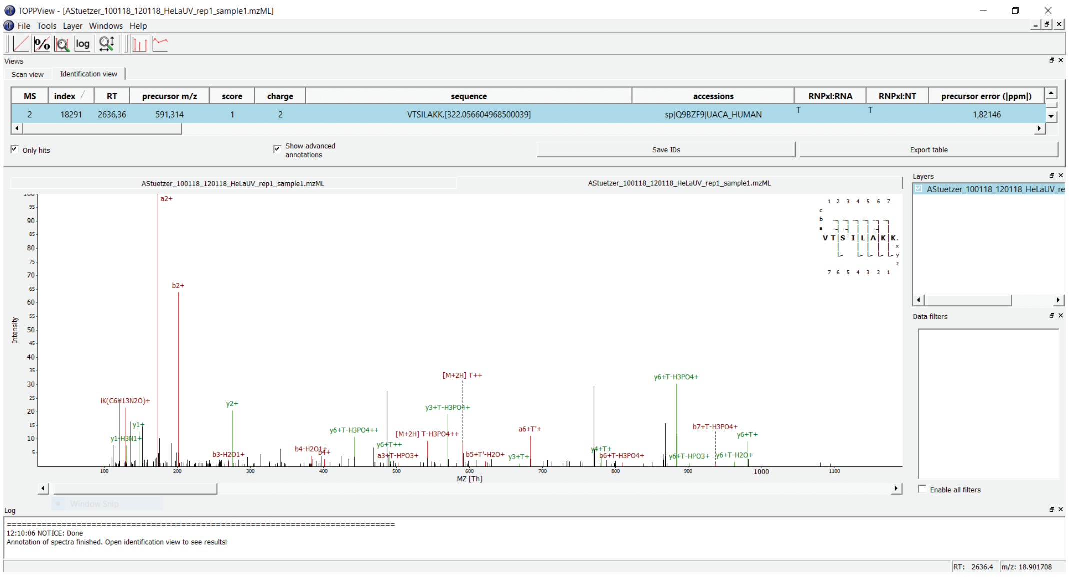

7)

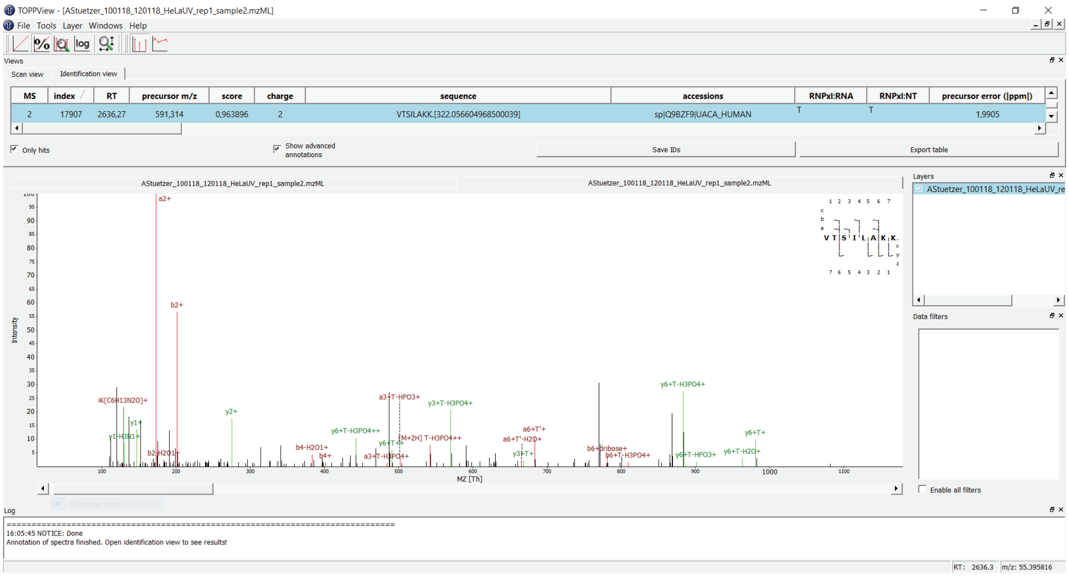

TOPPView - [AStuetzer_100118_120118_HeLaUV_rep1_sample2.mzML]
File Tools Layer Windows Help
Views
Scan view
Identification view
MS | index | RT | precursor m/z | score | charge | sequence | accessions | RNPxl:RNA | RNPxl:NT | precursor error (|ppm|)
2 | 17907 | 2636,27 | 591,314 | 0,963896 | 2 | VTSILAKK.[322.056604968500039] | sp|Q9BZF9|UACA_HUMAN | T | T | 1,9905
Only hits
Show advanced annotations
Save IDs
Export table
AStuetzer_100118_120118_HeLaUV_rep1_sample2.mzML
Layers
Data filters
Enable all filters
Intensity
MZ [Th]
Log
16:05:45 NOTICE: Done
Annotation of spectra finished. Open identification view to see results!
RT: 2636.3
m/z: 55.395816

8)

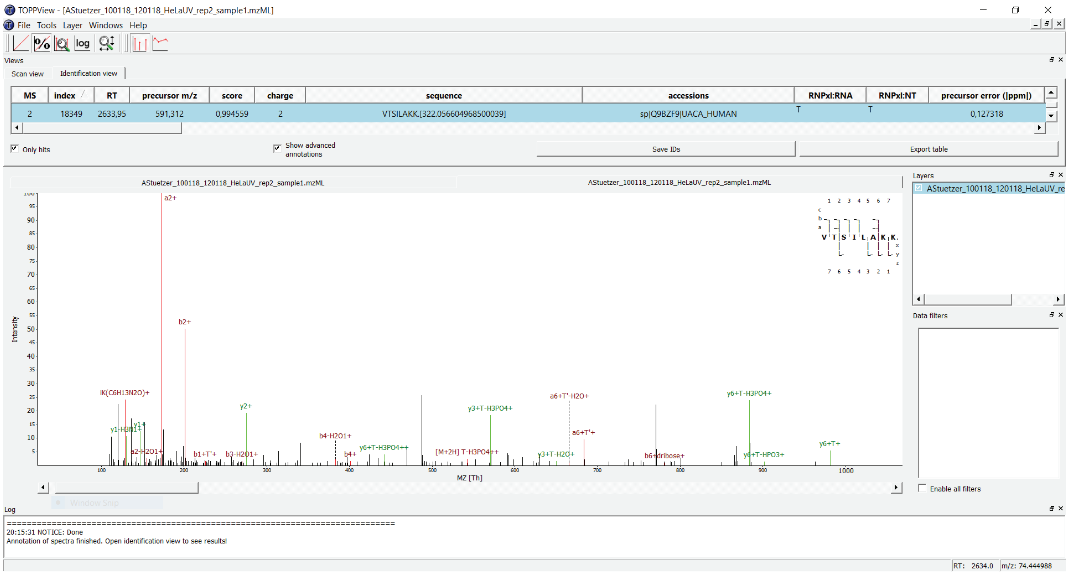

TOPPView - [AStuetzer_100118_120118_HeLaUV_rep2_sample1.mzML]
File Tools Layer Windows Help
Views
Scan view
Identification view
MS | index | RT | precursor m/z | score | charge | sequence | accessions | RNPxl:RNA | RNPxl:NT | precursor error (|ppm|)
2 | 18349 | 2633,95 | 591,312 | 0,994559 | 2 | VTSILAKK.[322.056604968500039] | sp|Q9BZF9|UACA_HUMAN | T | T | 0,127318
Only hits
Show advanced annotations
Save IDs
Export table
AStuetzer_100118_120118_HeLaUV_rep2_sample1.mzML
Layers
Data filters
Enable all filters
Intensity
MZ [Th]
Log
20:15:31 NOTICE: Done
Annotation of spectra finished. Open identification view to see results!
RT: 2634.0
m/z: 74.444988

9)

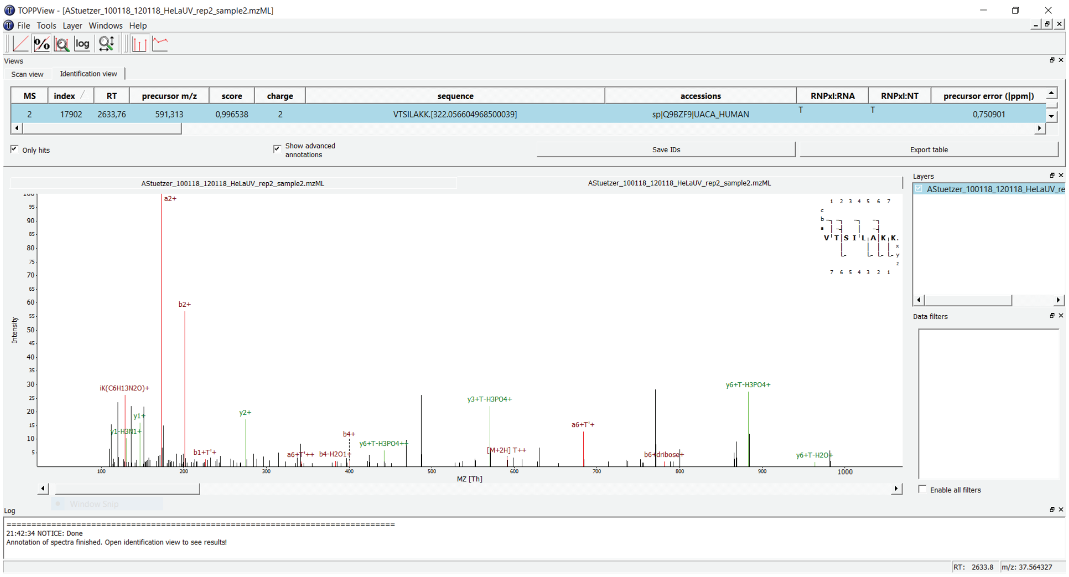

TOPPView - [AStuetzer_100118_120118_HeLaUV_rep2_sample2.mzML]
File Tools Layer Windows Help
Views
Scan view
Identification view
MS | index | RT | precursor m/z | score | charge | sequence | accessions | RNPxl:RNA | RNPxl:NT | precursor error (|ppm|)
2 | 17902 | 2633,76 | 591,313 | 0,996538 | 2 | VTSILAKK.[322.056604968500039] | sp|Q9BZF9|UACA_HUMAN | T | T | 0,750901
Only hits
Show advanced annotations
Save IDs
Export table
AStuetzer_100118_120118_HeLaUV_rep2_sample2.mzML
Layers
Data filters
Enable all filters
Intensity
MZ [Th]
Log
21:42:34 NOTICE: Done
Annotation of spectra finished. Open identification view to see results!
RT: 2633.8
m/z: 37.564327

10) Zinc finger protein 658B

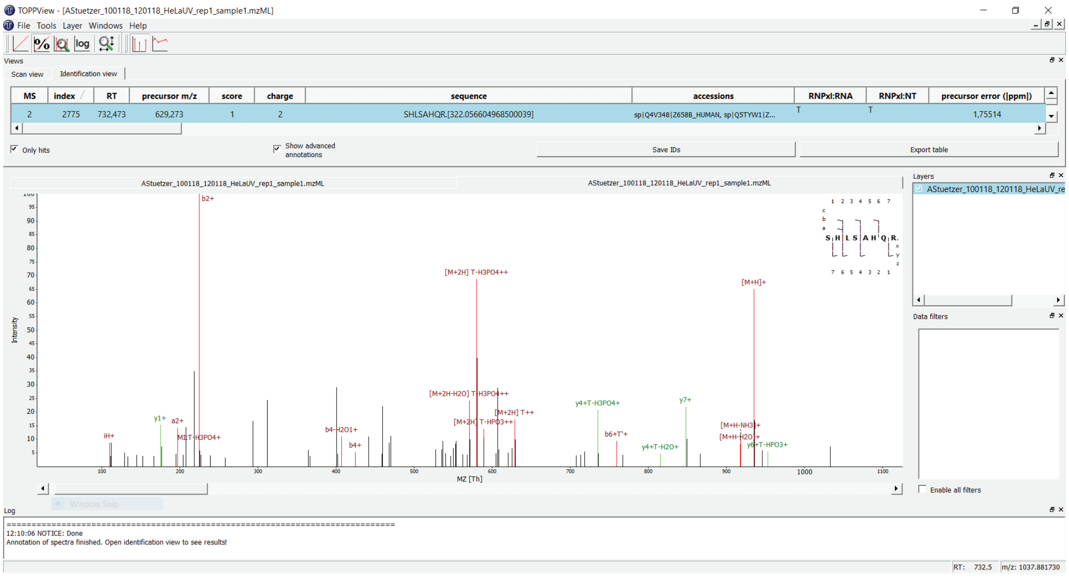

11) Zinc finger protein 40

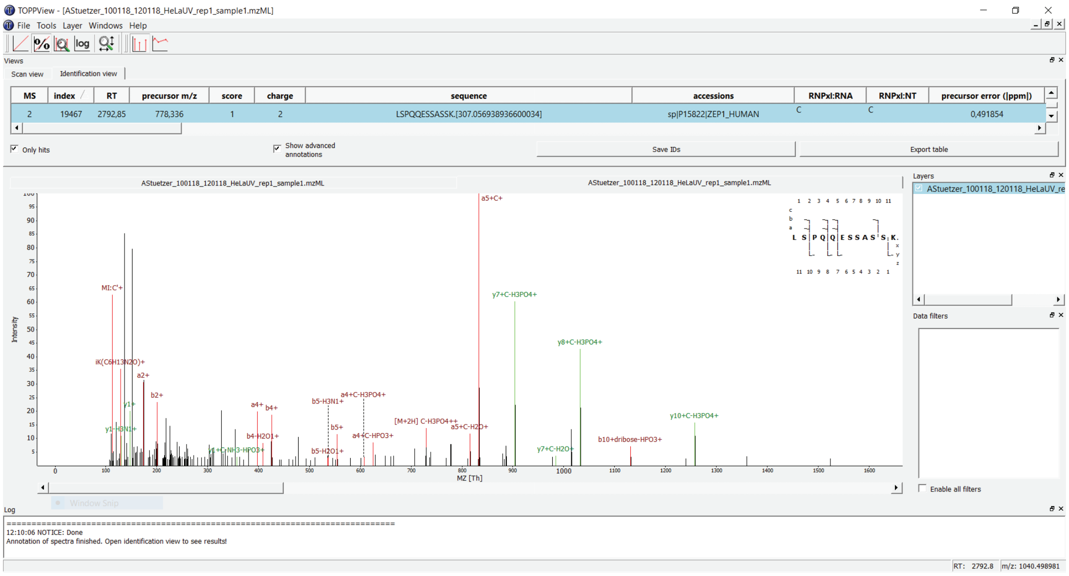

12) Zinc finger protein 229

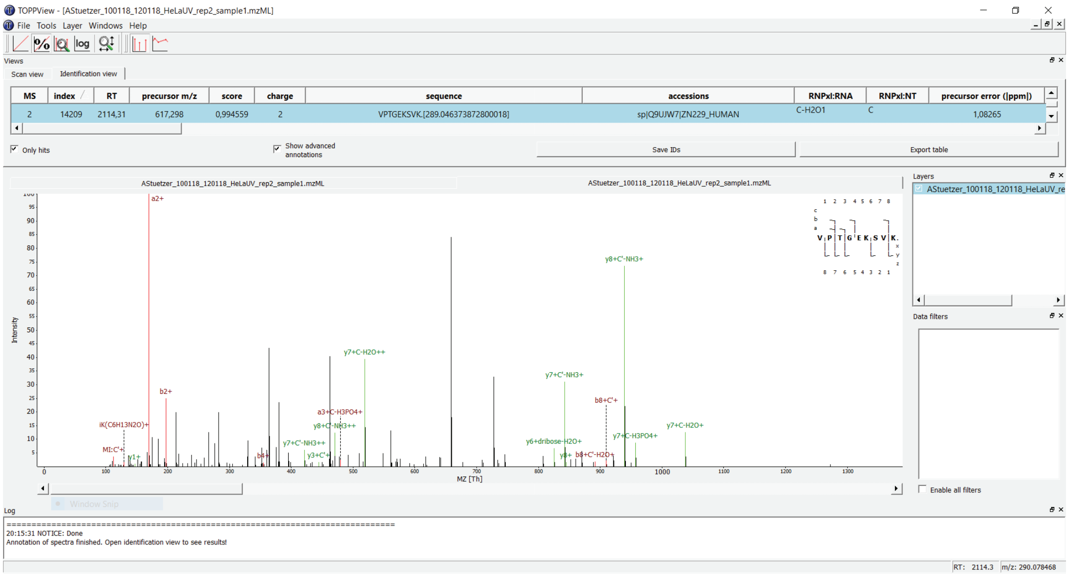

TOPPView spectra - unambiguous cross-link spectrum matches

UV cross-linked HeLa nuclei (chromatin precipitation), RNP^{xl}search_settings#2

1) E3 ubiquitin-protein ligase BRE1B

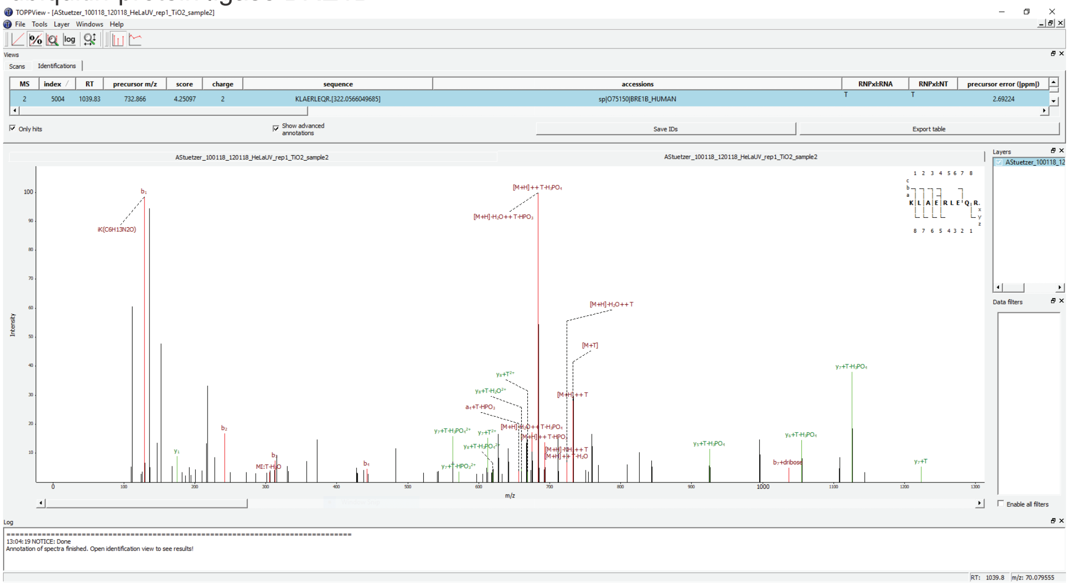

2)

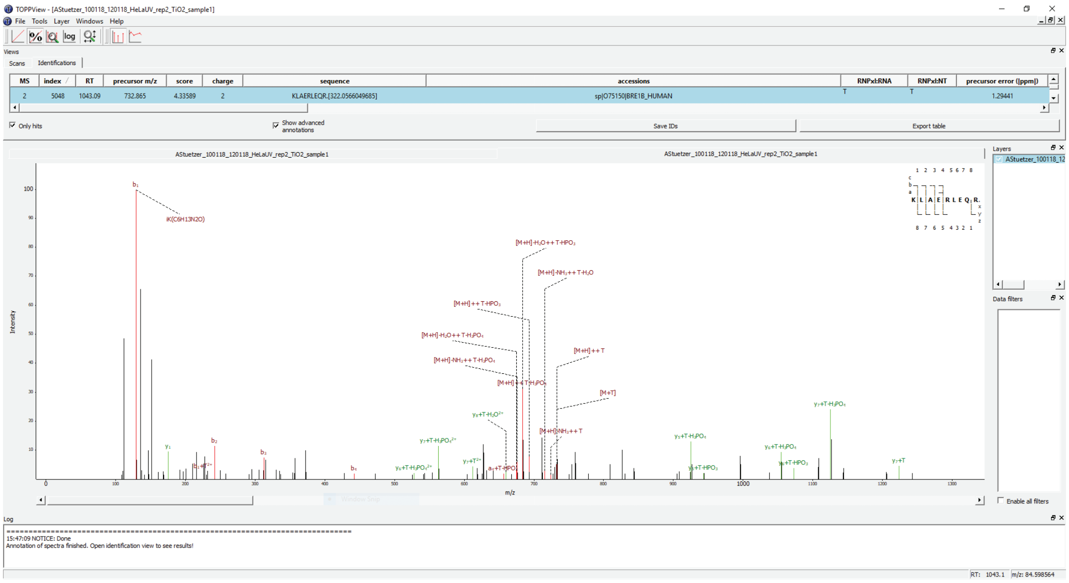

3)

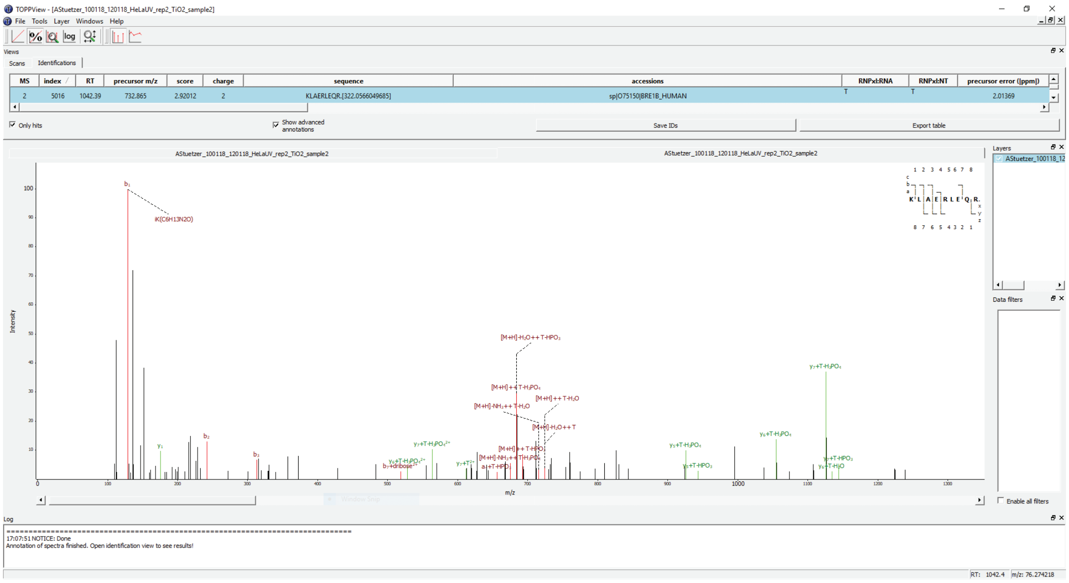

4) Centrosomal protein of 85 kDa

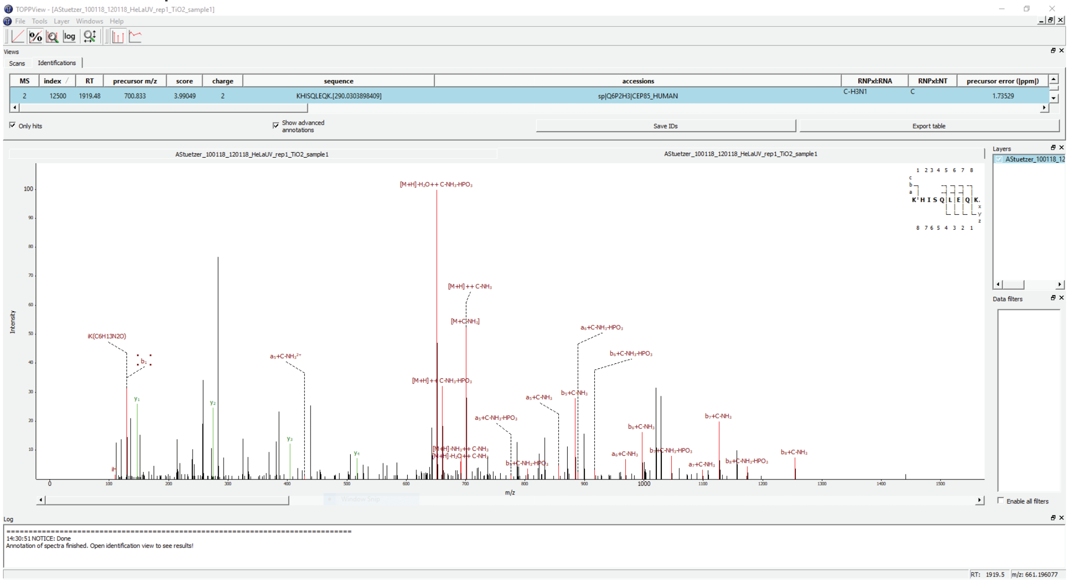

5)

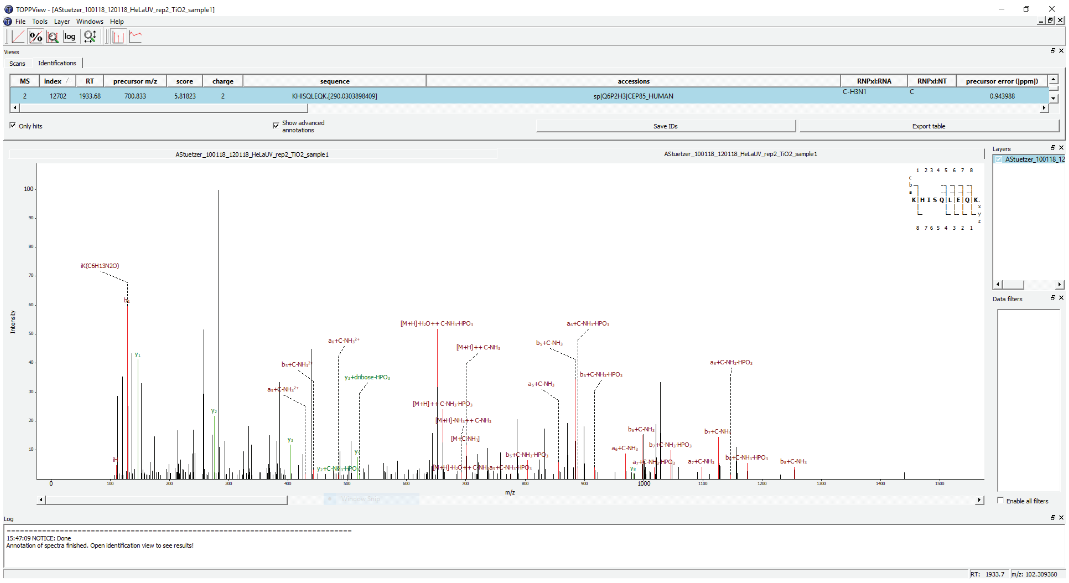

6) Dual specificity protein phosphatase 16

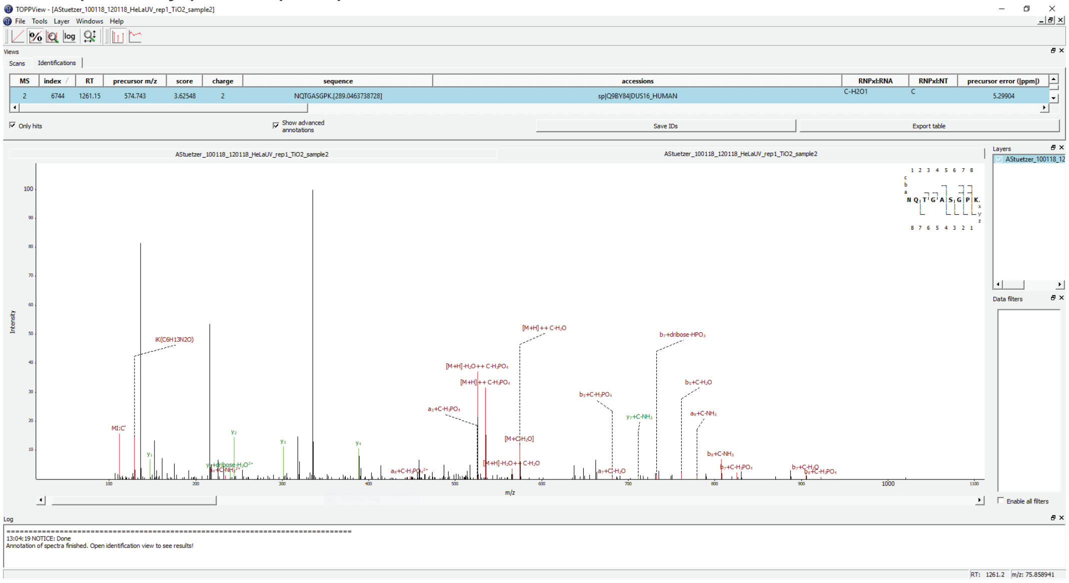

7)

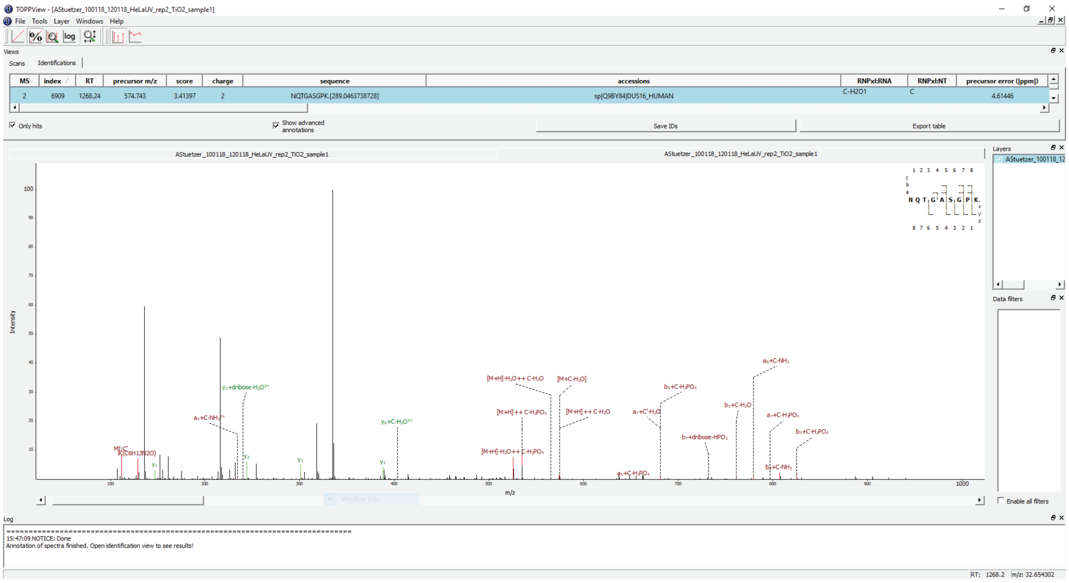

8)

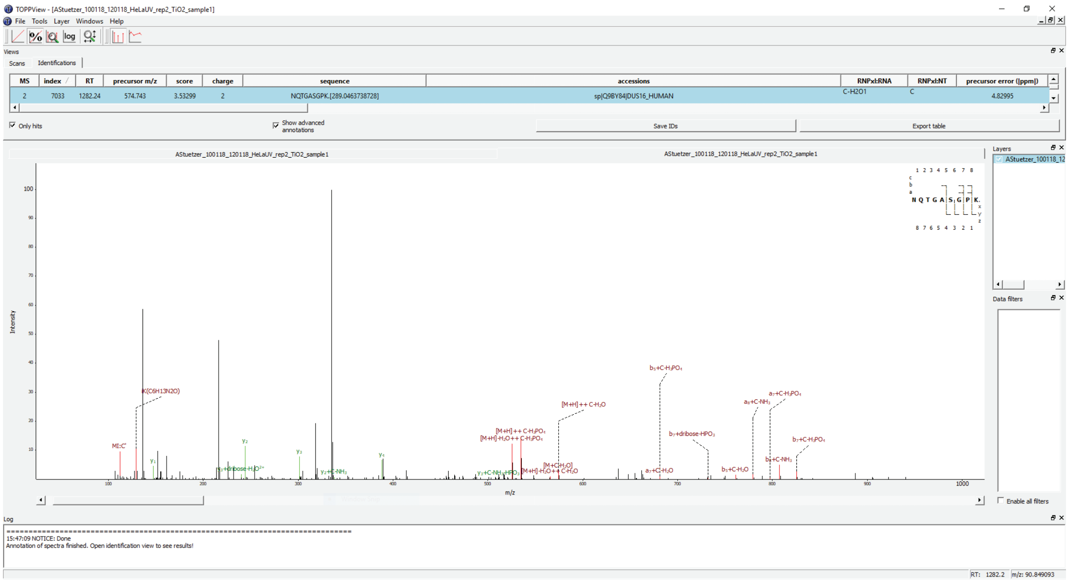

9) UDP-N-acetylglucosamine--peptide N-acetylglucosaminyltransferase 110 kDa subunit

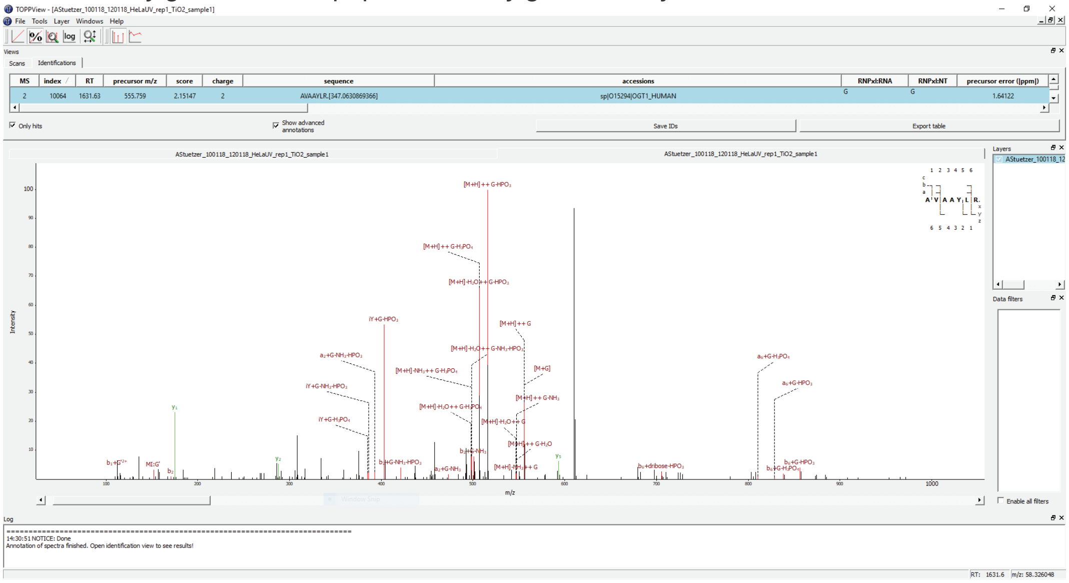

10)

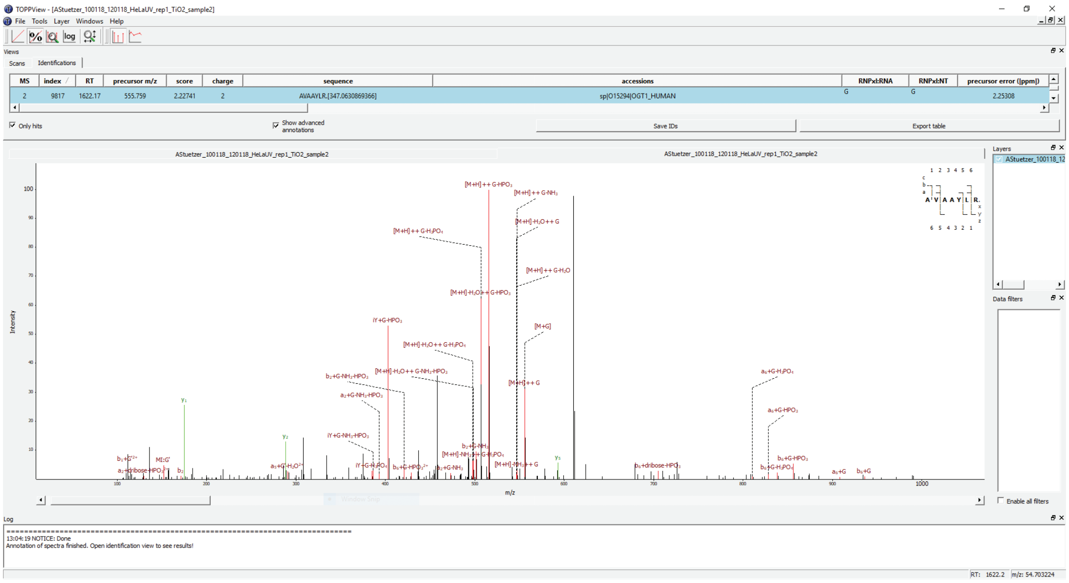
TOPPView - [AStuetzer_100118_120118_HeLaUV_rep1_TiO2_sample2]
MS | index | RT | precursor m/z | score | charge | sequence | accessions | RNPxl:RNA | RNPxl:NT | precursor error (|ppm|)
2 | 9817 | 1622.17 | 555.759 | 2.22741 | 2 | AVAAYLR.[347.0630869366] | sp|O15294|OGT1_HUMAN | G | G | 2.25308
Only hits
Show advanced annotations
Save IDs
Export table
Intensity
m/z
13:04:19 NOTICE: Done
Annotation of spectra finished. Open identification view to see results!

11)

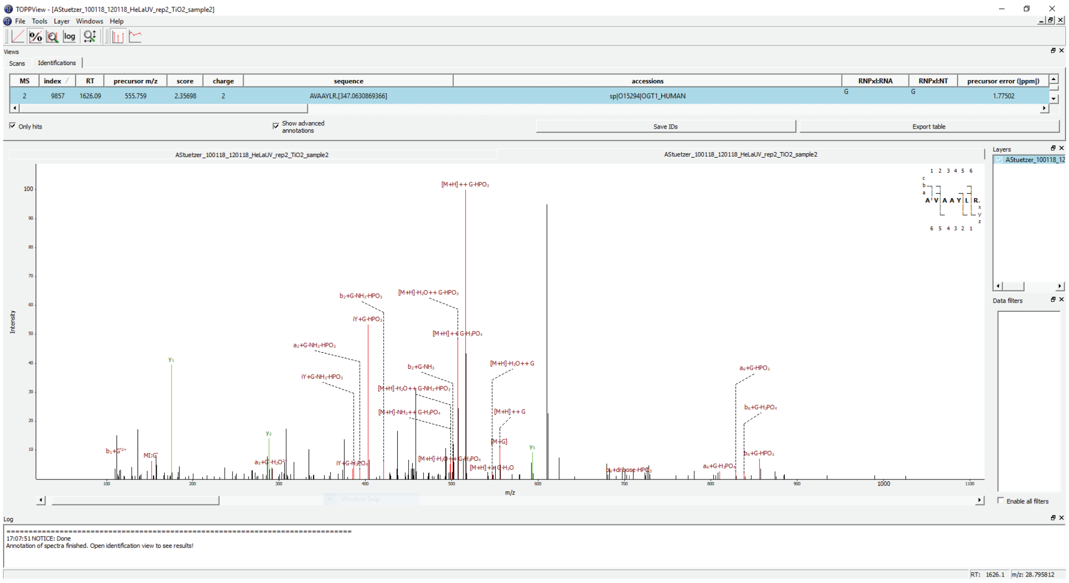
TOPPView - [AStuetzer_100118_120118_HeLaUV_rep2_TiO2_sample2]
MS | index | RT | precursor m/z | score | charge | sequence | accessions | RNPxl:RNA | RNPxl:NT | precursor error (|ppm|)
2 | 9857 | 1626.09 | 555.759 | 2.35698 | 2 | AVAAYLR.[347.0630869366] | sp|O15294|OGT1_HUMAN | G | G | 1.77502
Only hits
Show advanced annotations
Save IDs
Export table
Intensity
m/z
17:07:51 NOTICE: Done
Annotation of spectra finished. Open identification view to see results!

12)

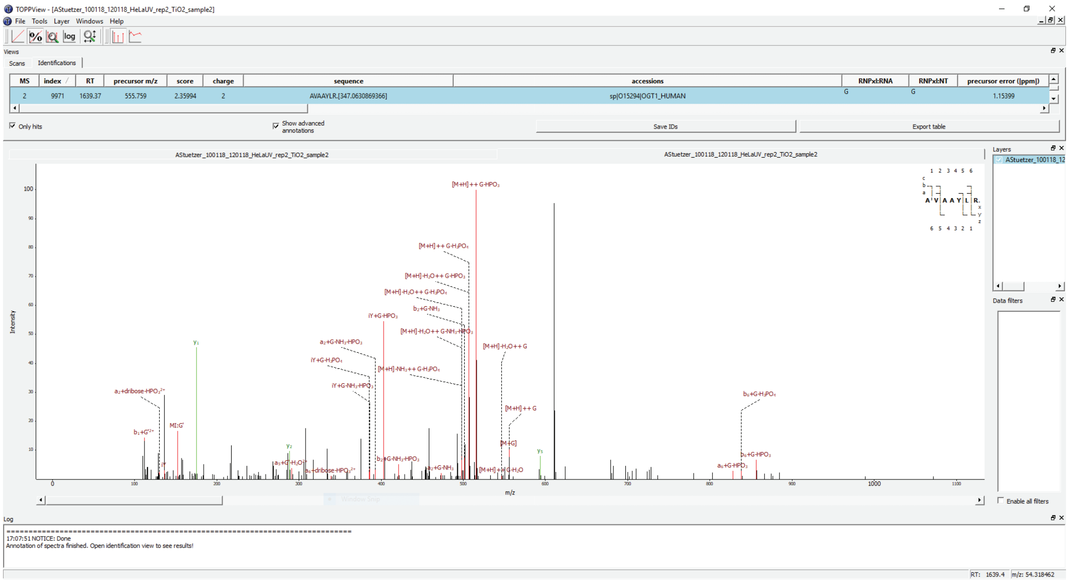
TOPPView - [AStuetzer_100118_120118_HeLaUV_rep2_TiO2_sample2]
MS | index | RT | precursor m/z | score | charge | sequence | accessions | RNPxl:RNA | RNPxl:NT | precursor error (|ppm|)
2 | 9971 | 1639.37 | 555.759 | 2.35994 | 2 | AVAAYLR.[347.0630869366] | sp|O15294|OGT1_HUMAN | G | G | 1.15399
Only hits
Show advanced annotations
Save IDs
Export table
Intensity
m/z
17:07:51 NOTICE: Done
Annotation of spectra finished. Open identification view to see results!

13) PHD finger protein 19

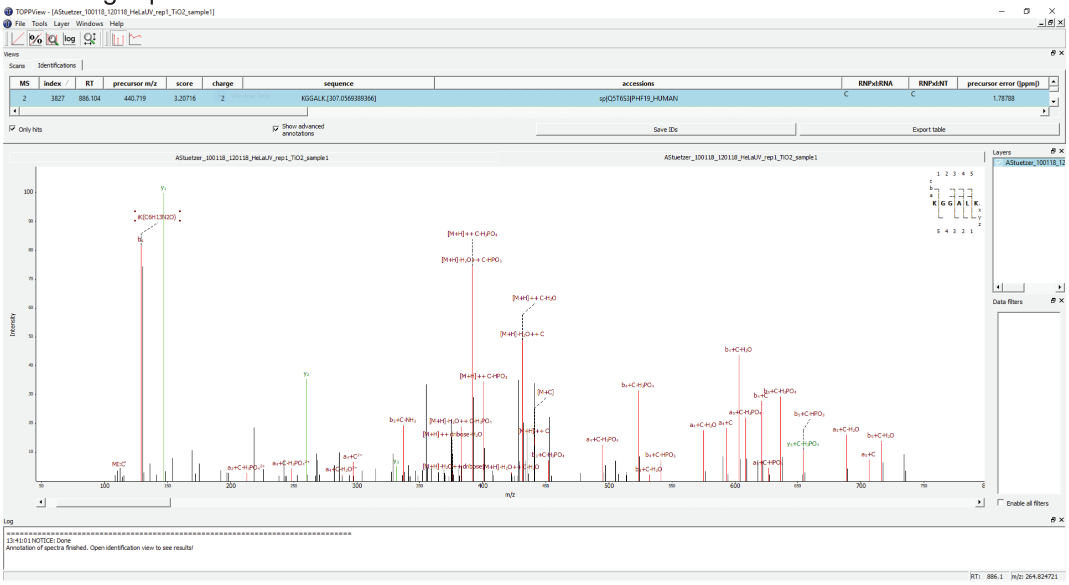

14)

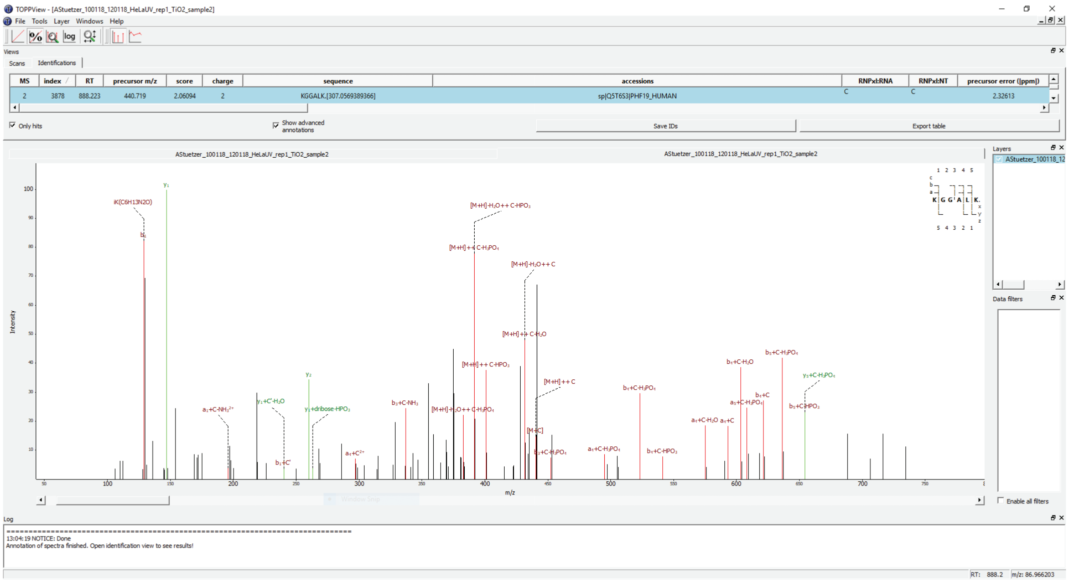

15)

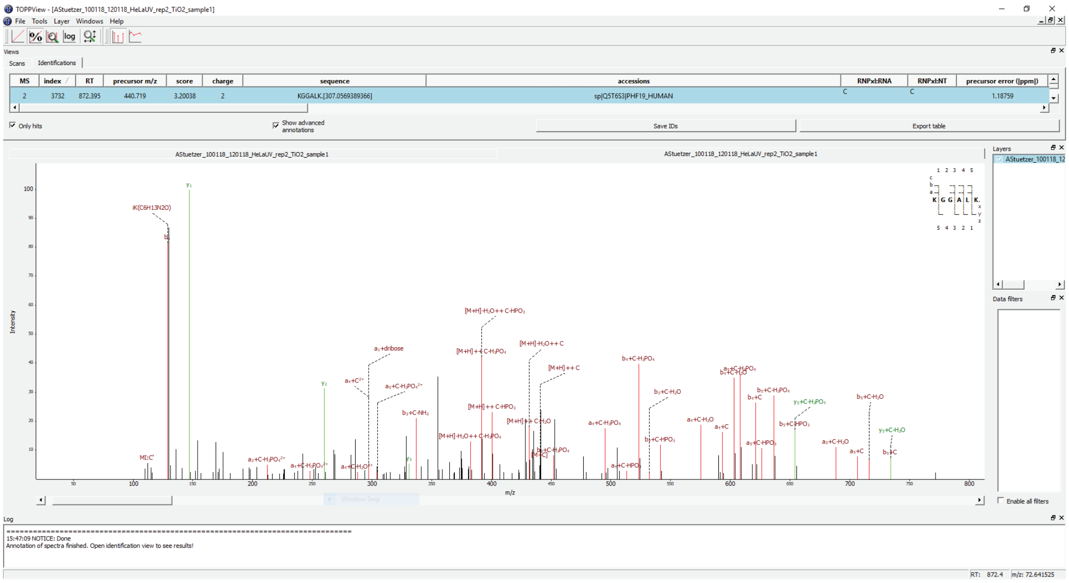

16)

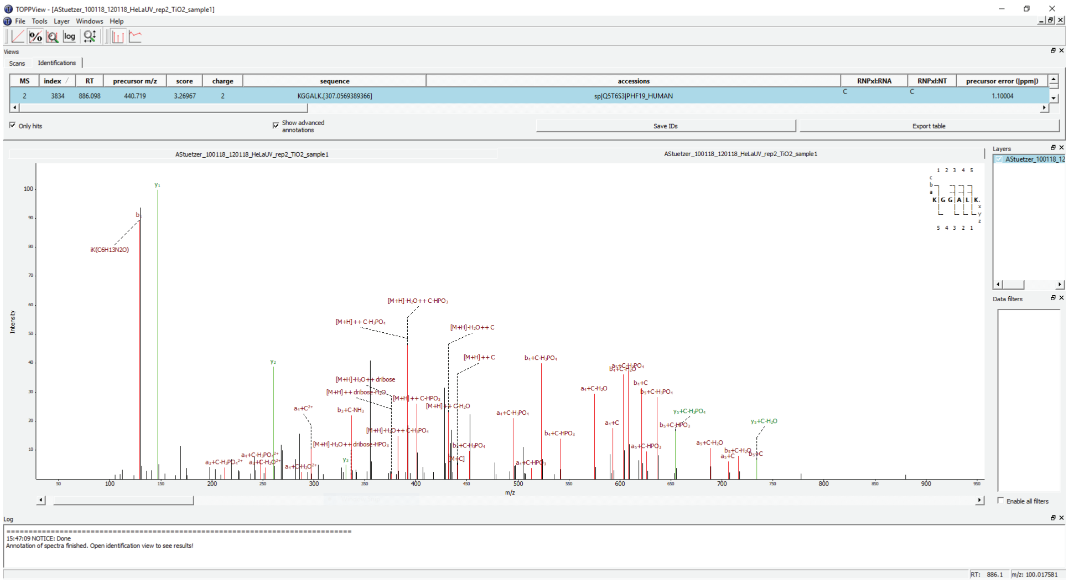

17) Ribonuclease H2 subunit B

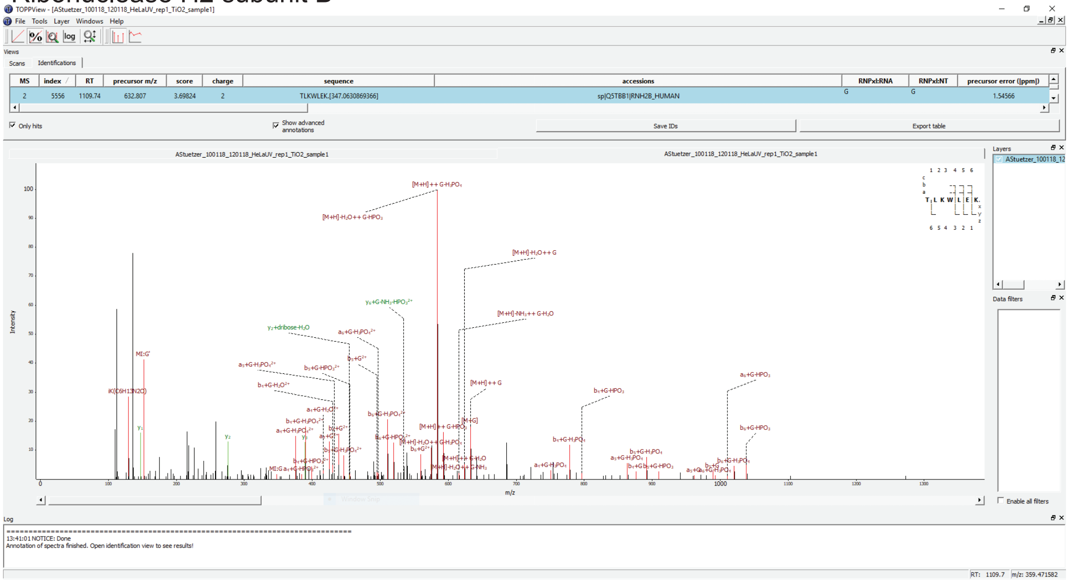

18)

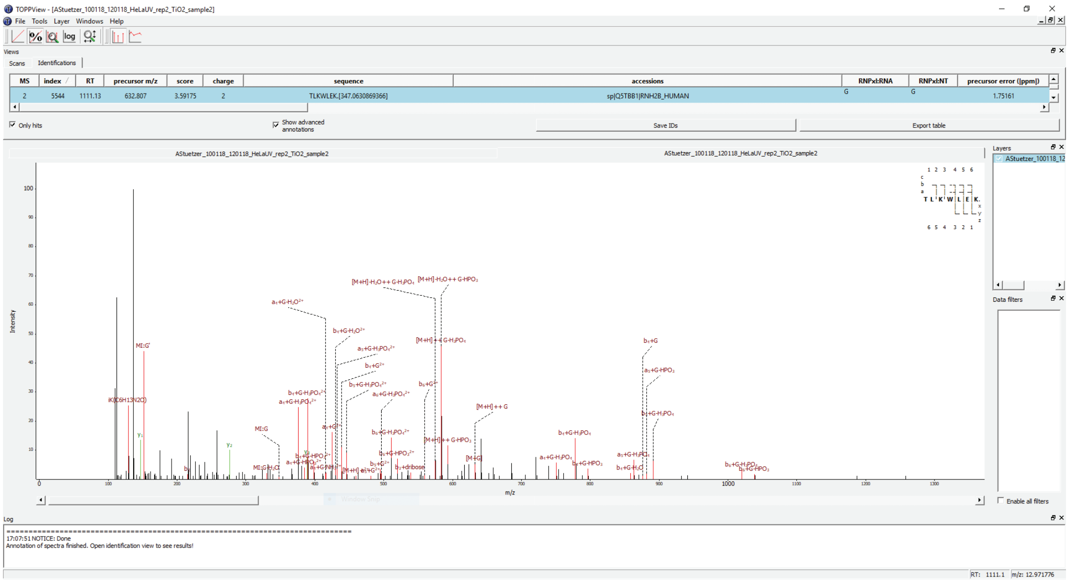

19) DNA-directed RNA polymerase III subunit RPC4

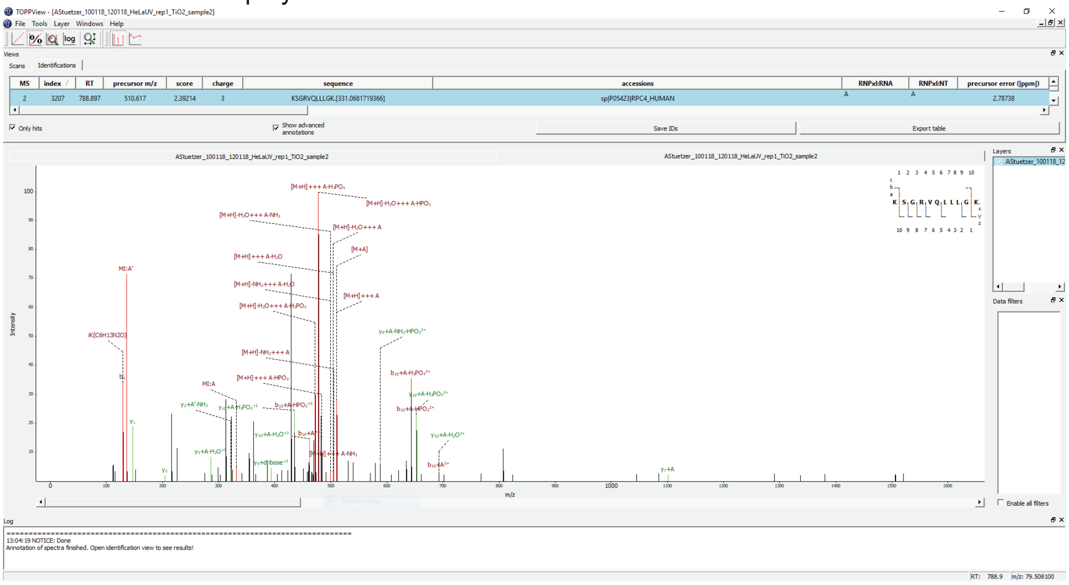

20) Titin

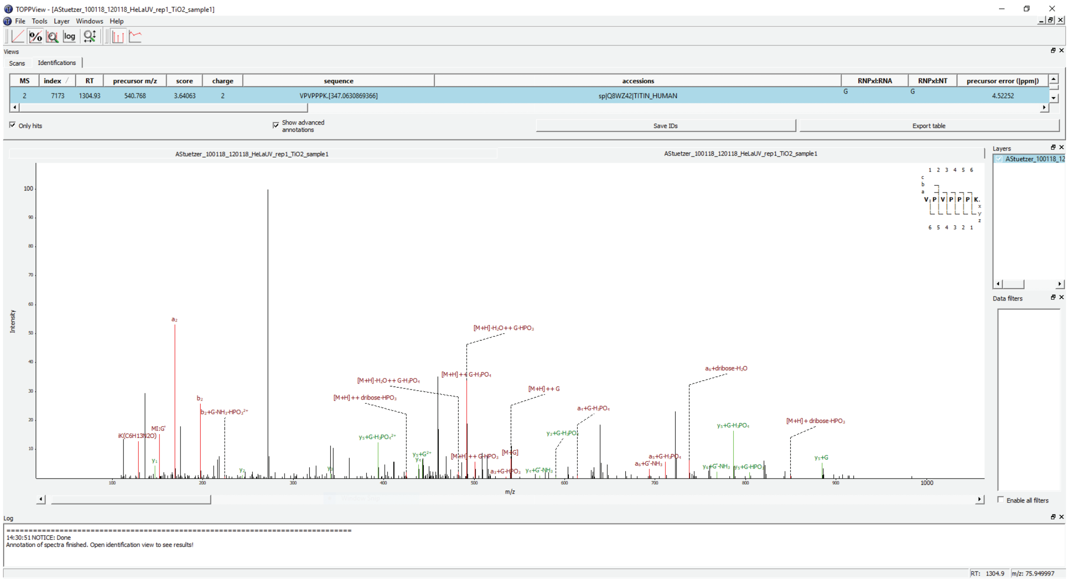

21)

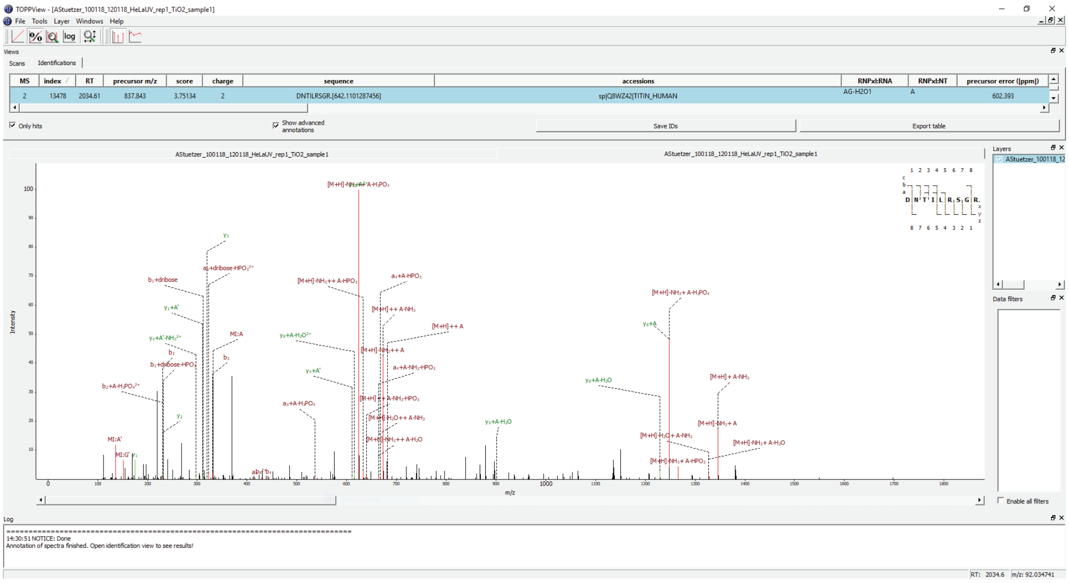

22) Ubiquitin-like modifier-activating enzyme 5

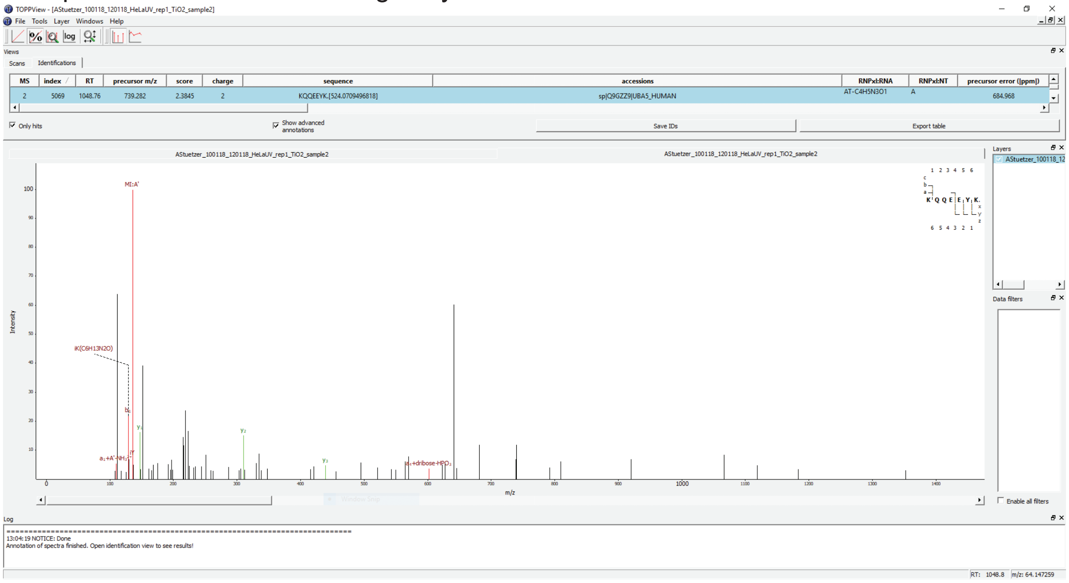

23) Zinc finger and BTB domain-containing protein 38

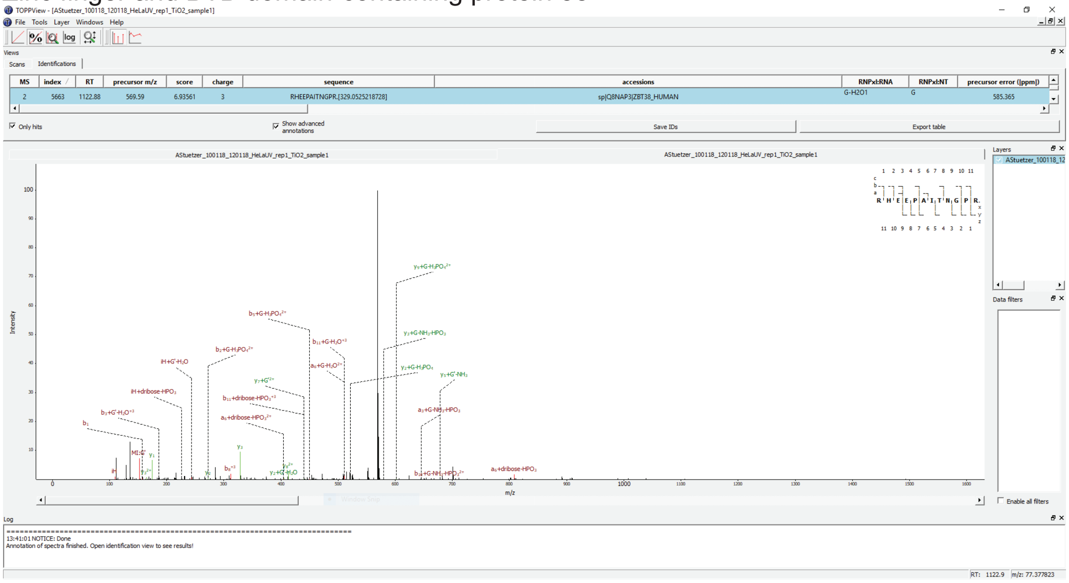

24) Zinc finger homeobox protein 2

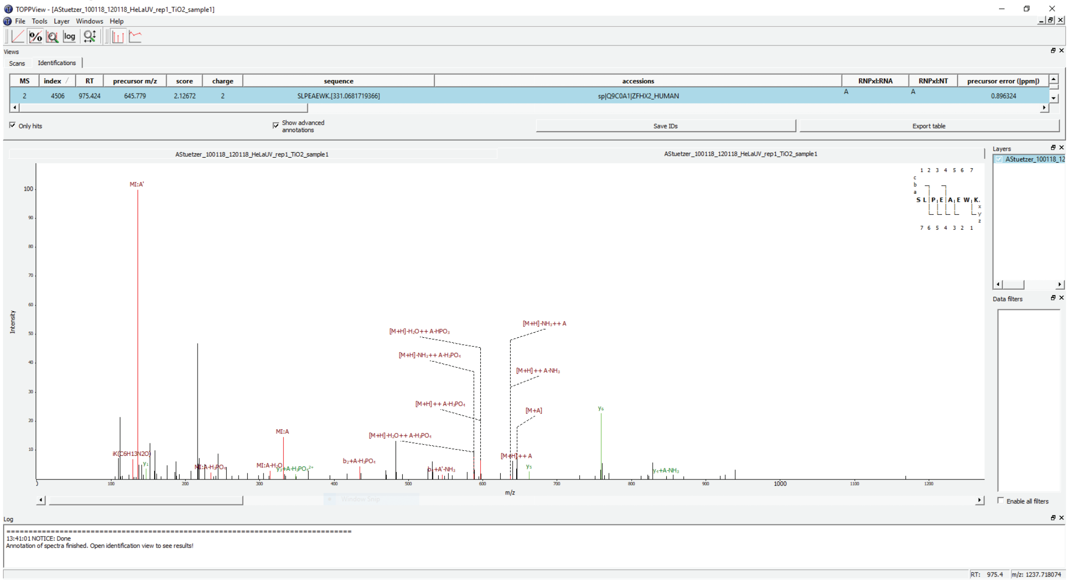

TOPPView spectra - ambiguous cross-link spectrum matches

UV cross-linked HeLa nuclei (chromatin precipitation), RNP[xl]search_settings#2

1) Nucleosome-remodeling factor subunit BPTF

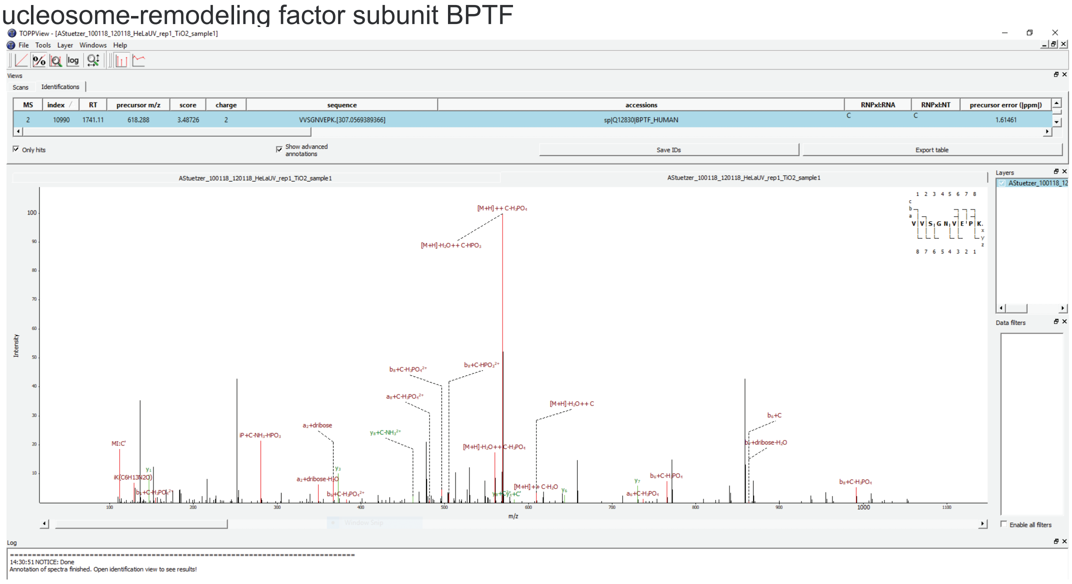

2) Elongin-A

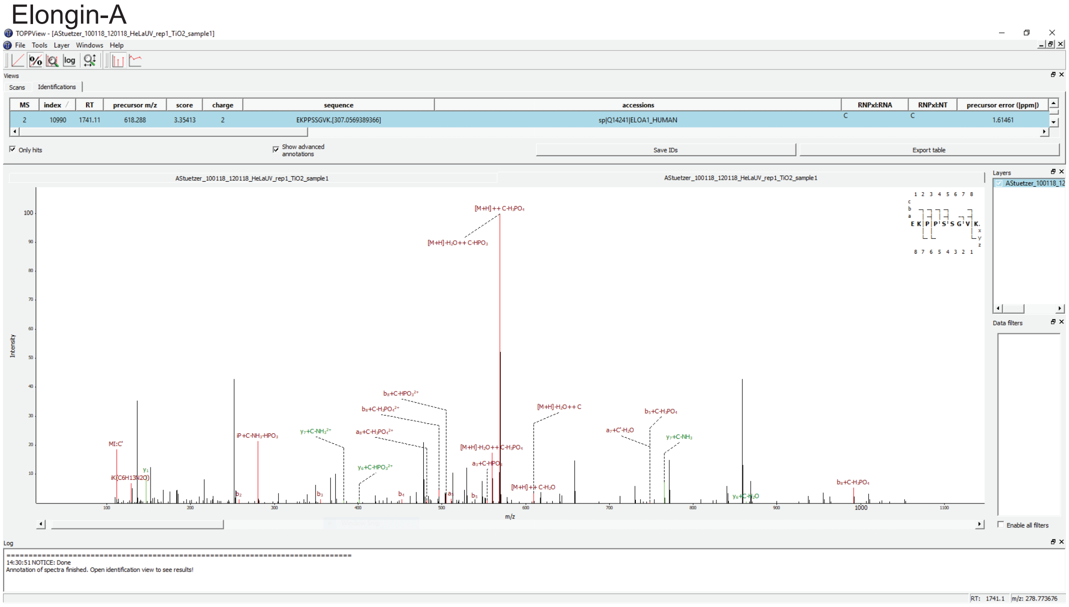

3) Nucleosome-remodeling factor subunit BPTF

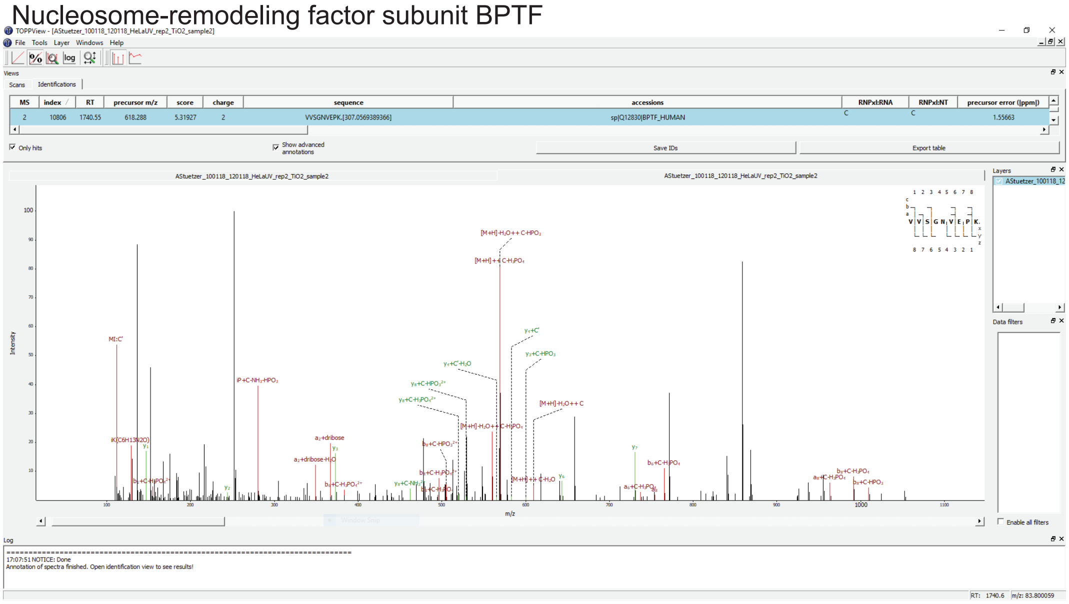

4) Dual specificity protein phosphatase 16

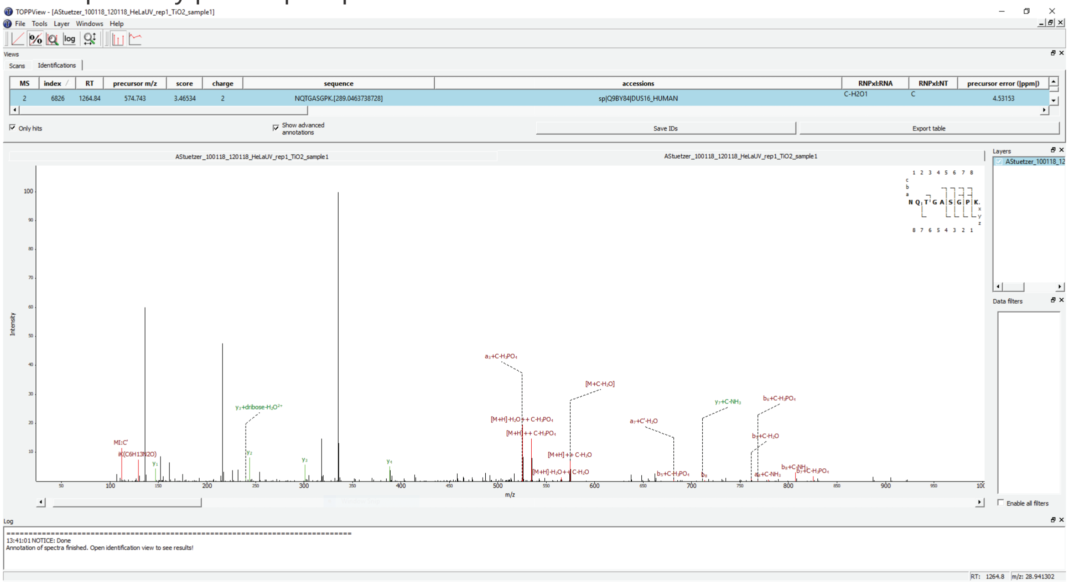

5)

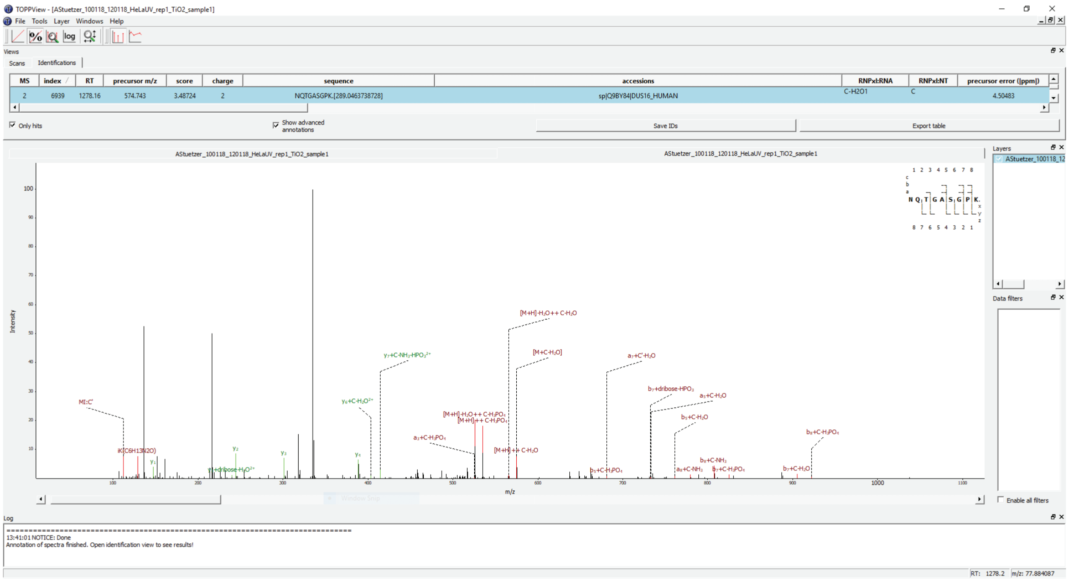

6) Tyrosine-protein kinase Fyn

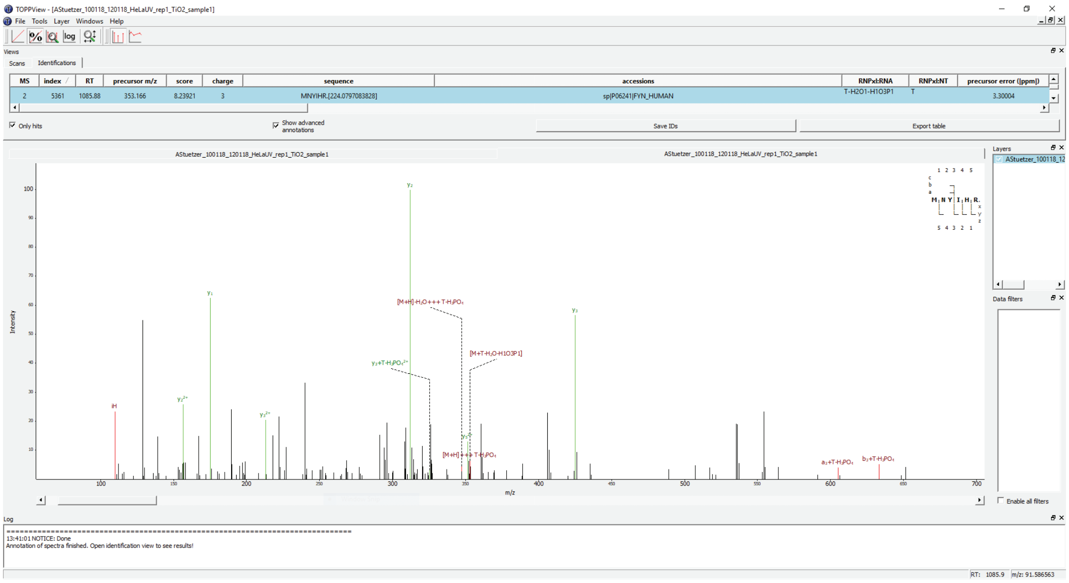

7) Tyrosine-protein kinase JAK2

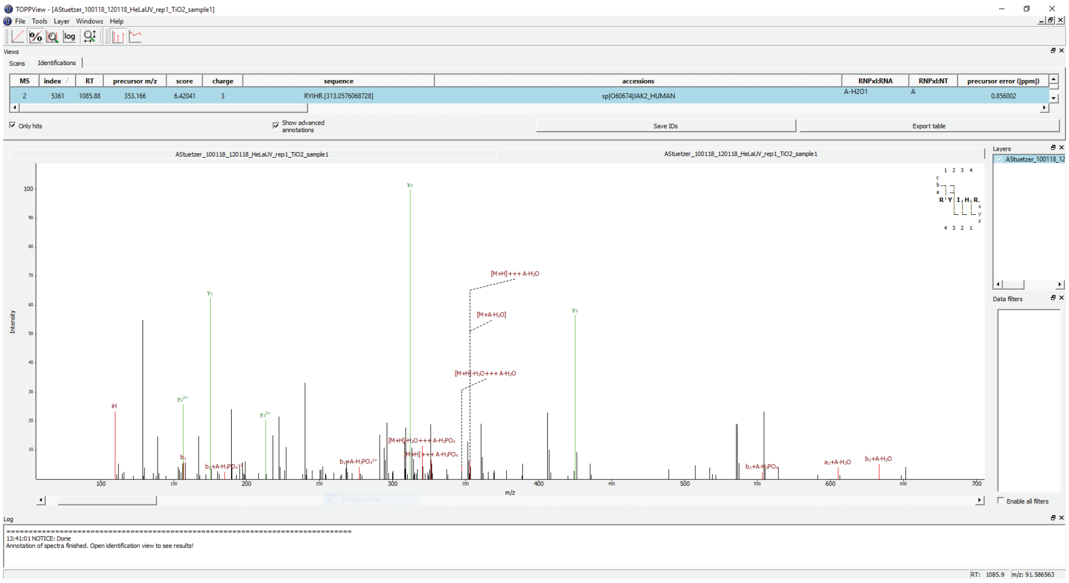

8) Calcium/calmodulin-dependent protein kinase type 1

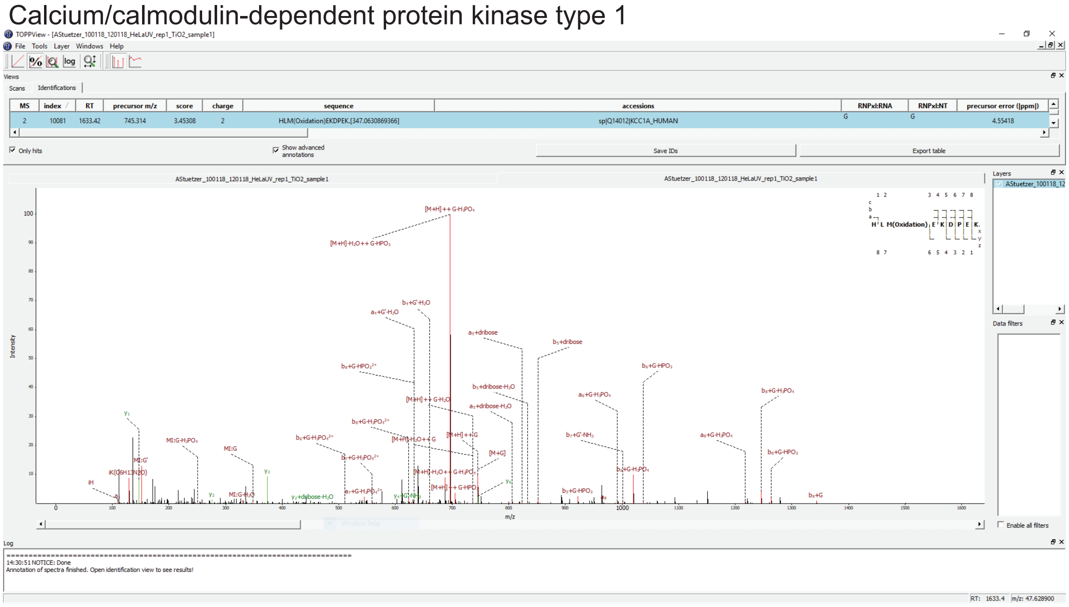

9) PiggyBac transposable element-derived protein 5

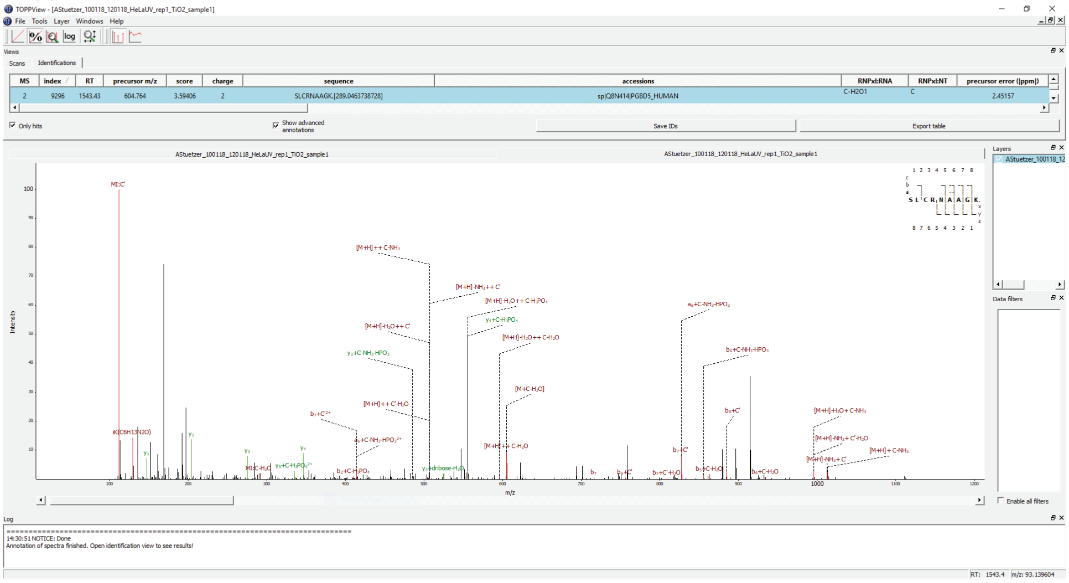

10) Arginine-glutamic acid dipeptide repeats protein

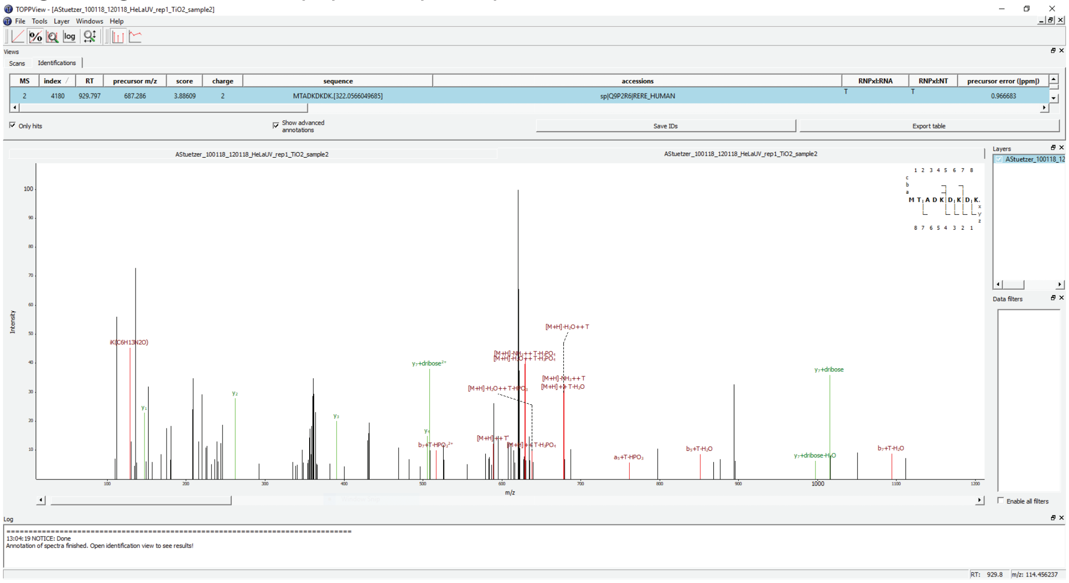

11) Zinc finger protein 98

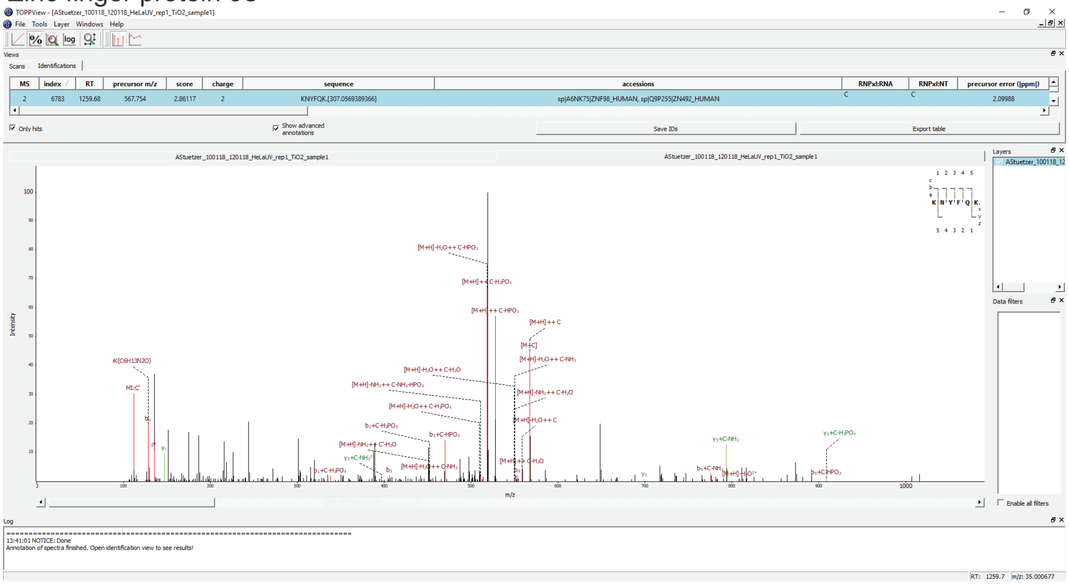

TOPPView spectra - unambiguous cross-link spectrum matches

UV cross-linked HeLa nuclei (SEC)

1) Constitutive coactivator of PPAR-gamma-like protein 1

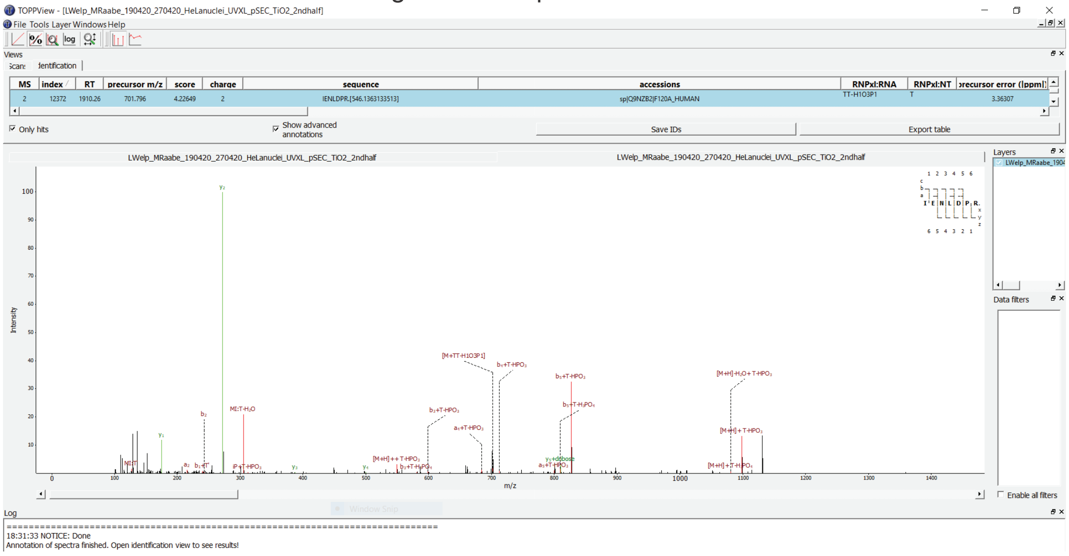

2) Glyceraldehyde-3-phosphate dehydrogenase

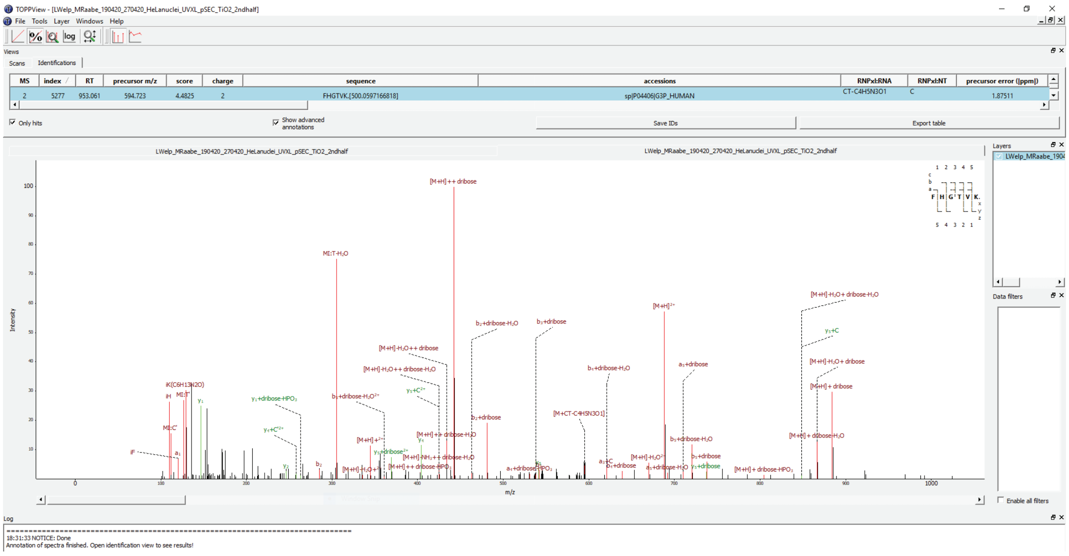

3) Histone H2A

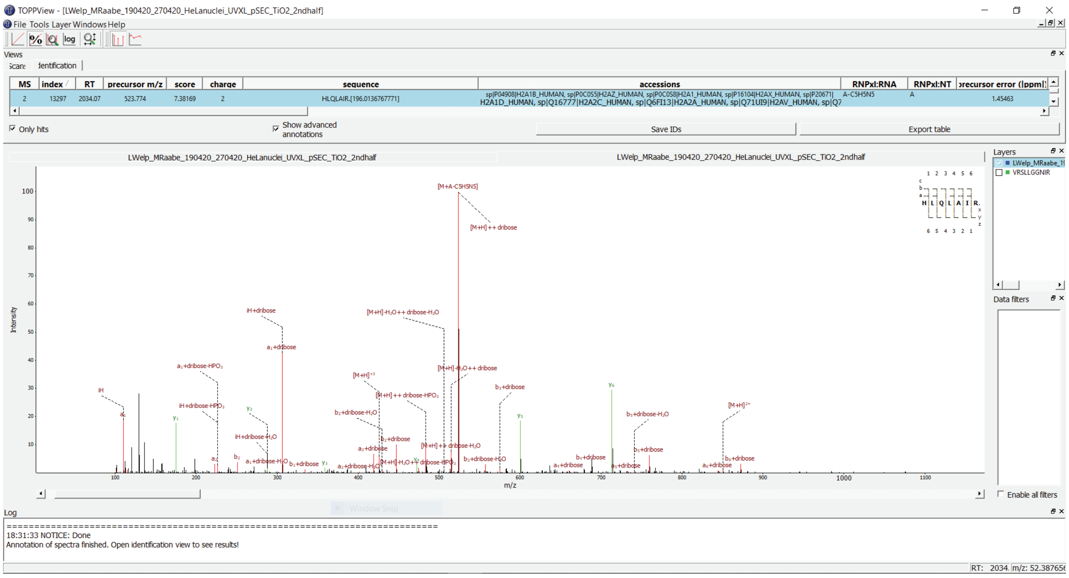

4)

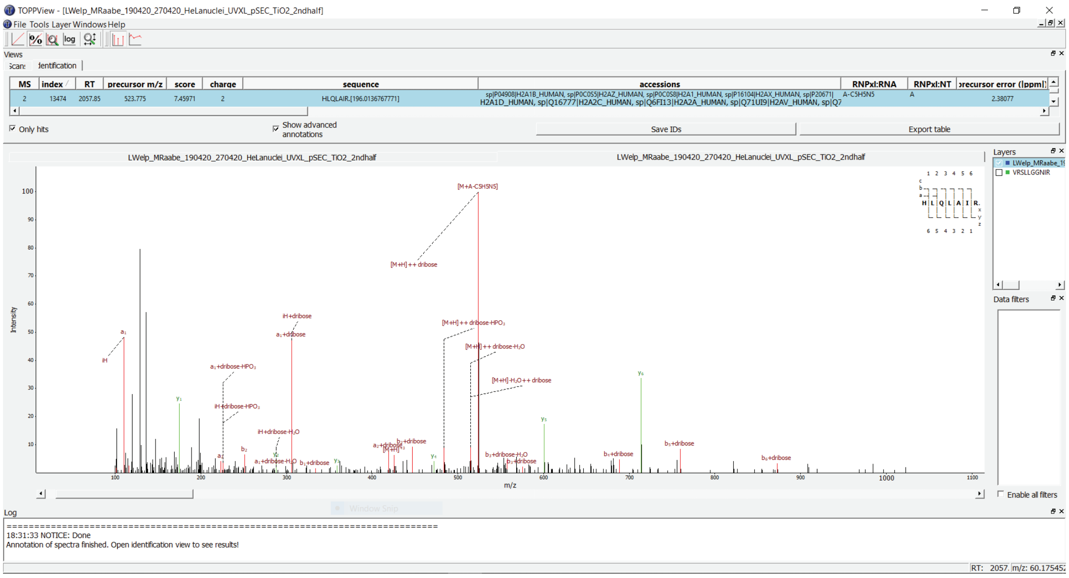

5) Histone H2B

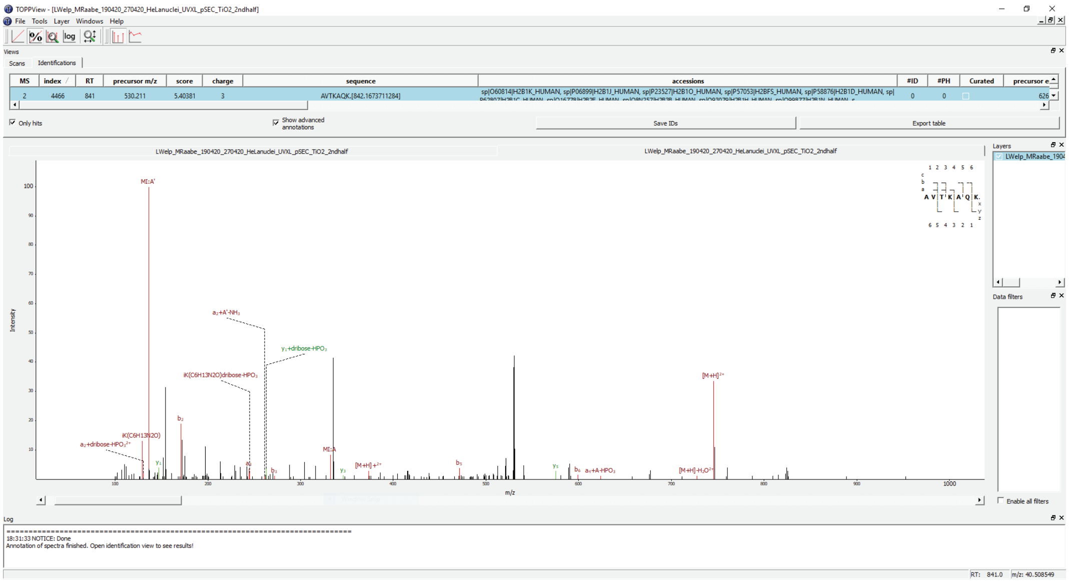

6)

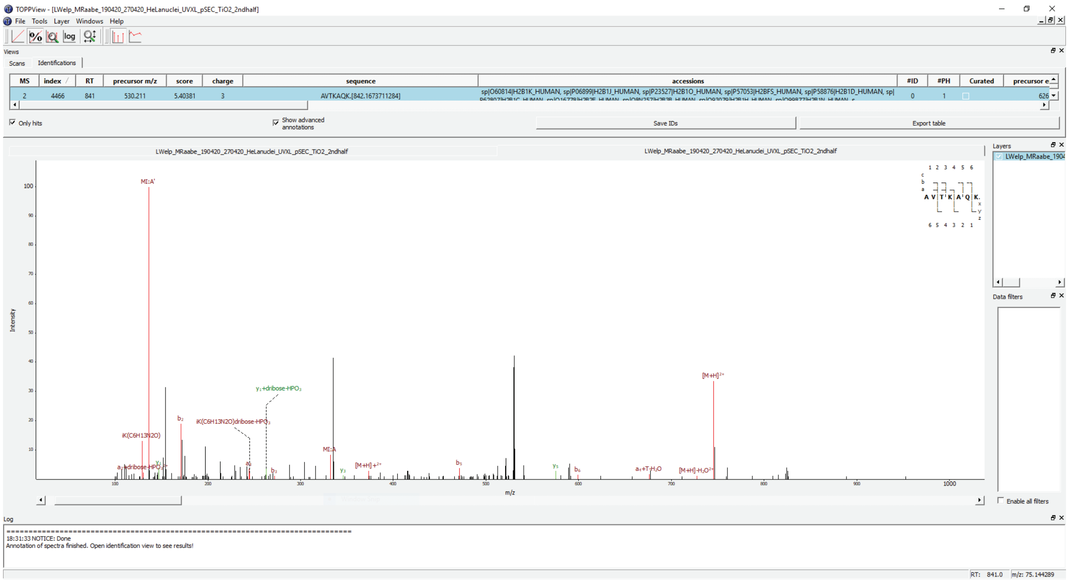

7)

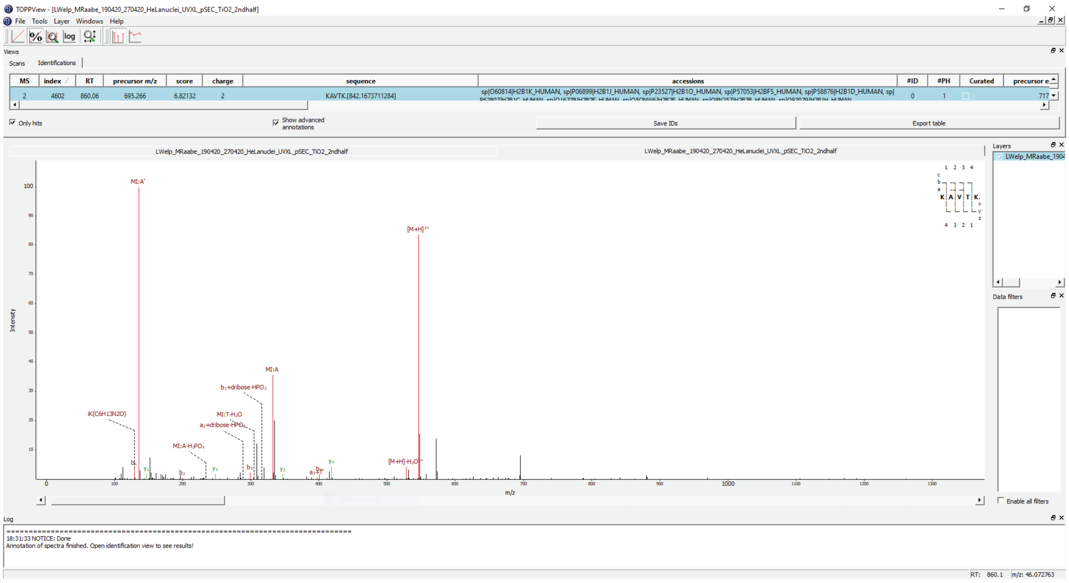
TOPPView - [LWelp_MRaabe_190420_270420_HeLanuclei_UVXL_pSEC_TiO2_2ndhalf]
MS | index | RT | precursor m/z | score | charge | sequence | accessions | #ID | #PH | Curated
2 | 4602 | 860.06 | 695.266 | 6.82132 | 2 | KAVTK.[842.1673711284]
Save IDs
Export table
MI:A'
[M+H]2+
MI:A
m/z
Intensity

8)

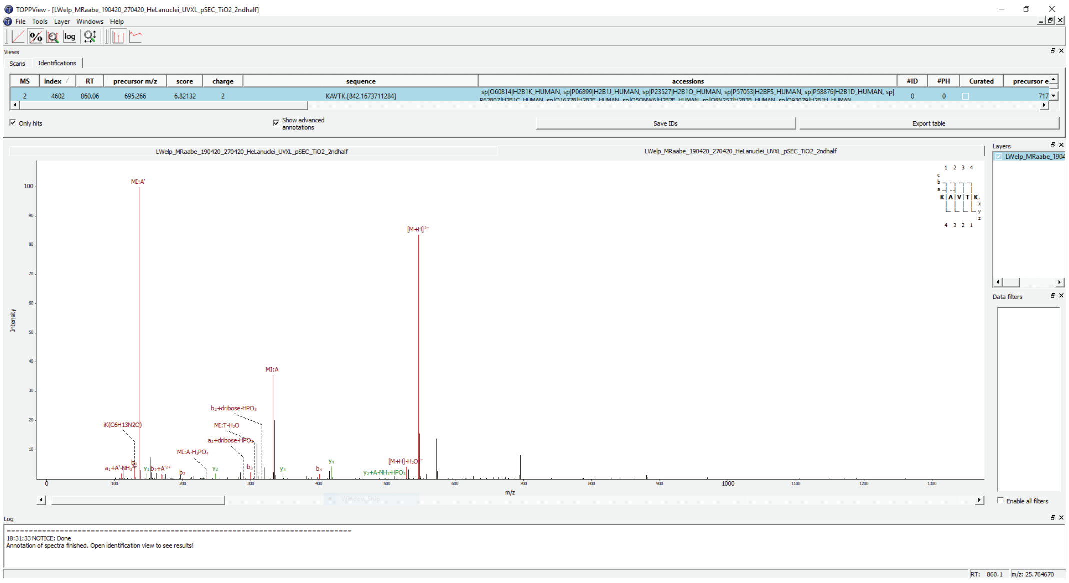
TOPPView - [LWelp_MRaabe_190420_270420_HeLanuclei_UVXL_pSEC_TiO2_2ndhalf]
MS | index | RT | precursor m/z | score | charge | sequence | accessions | #ID | #PH | Curated
2 | 4602 | 860.06 | 695.266 | 6.82132 | 2 | KAVTK.[842.1673711284]
Save IDs
Export table
MI:A'
[M+H]2+
MI:A
m/z
Intensity

9)

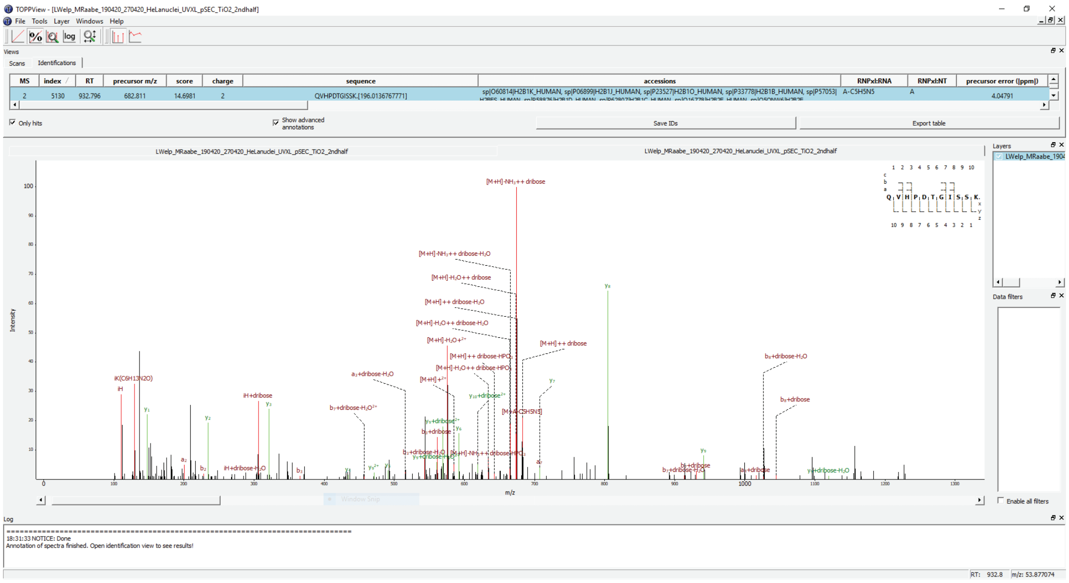
TOPPView - [LWelp_MRaabe_190420_270420_HeLanuclei_UVXL_pSEC_TiO2_2ndhalf]
MS | index | RT | precursor m/z | score | charge | sequence | accessions | RNPxl:RNA | RNPxl:NT | precursor error (|ppm|)
2 | 5130 | 932.796 | 682.811 | 14.6981 | 2 | QVHPDTGISSK.[196.0136767771] | A-C5H5N5 | A | 4.04791
Save IDs
Export table
[M+H]-NH3++ dribose
m/z
Intensity

10) Histone H3

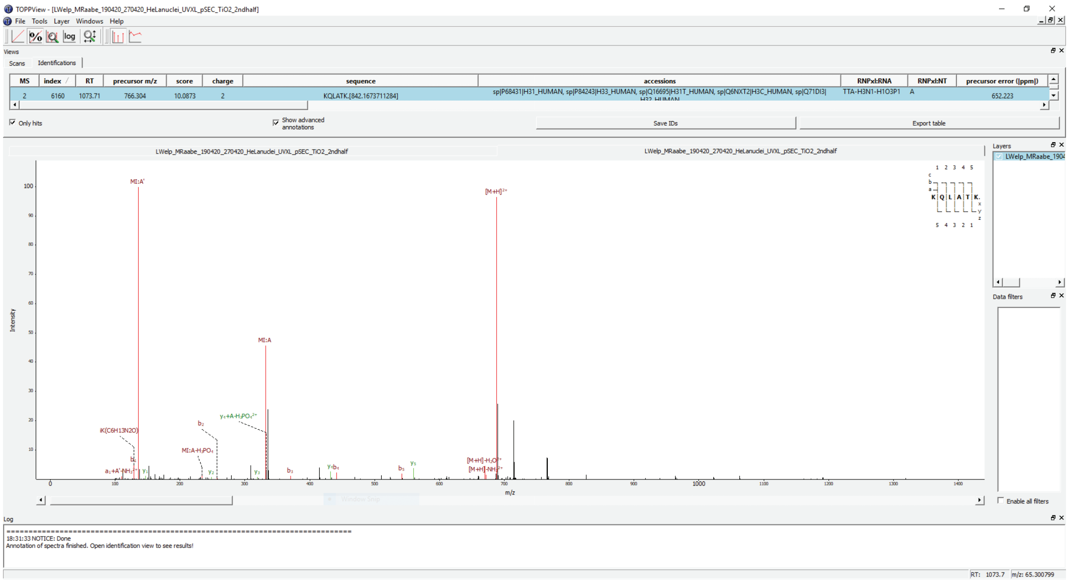

11)

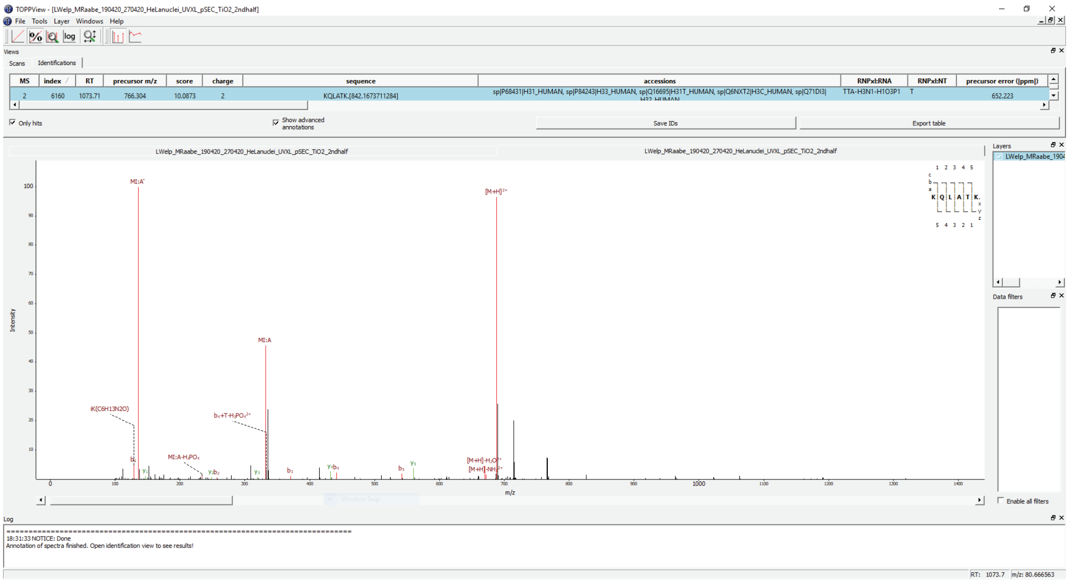

12)

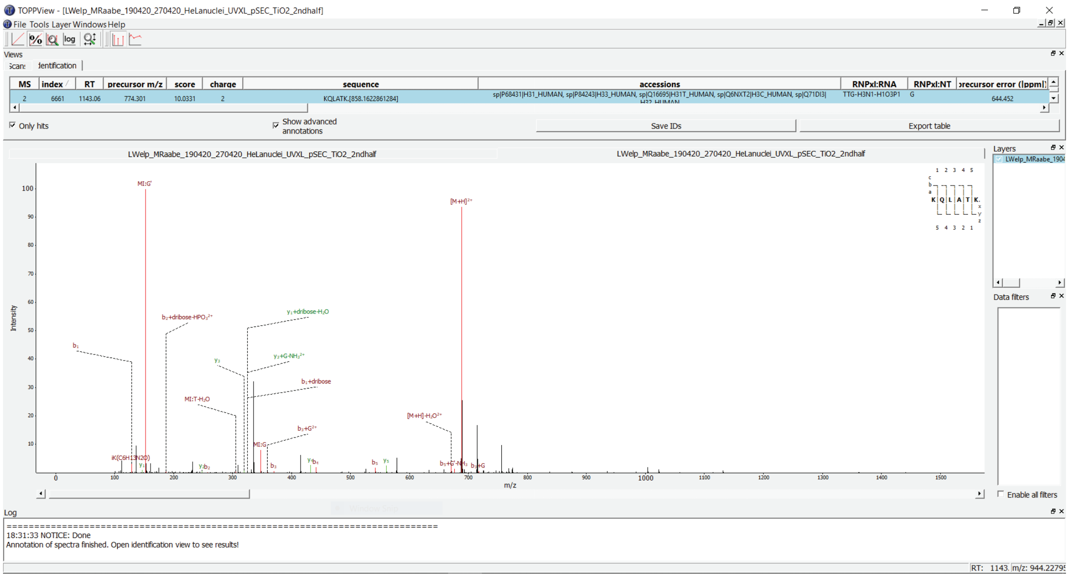

13)

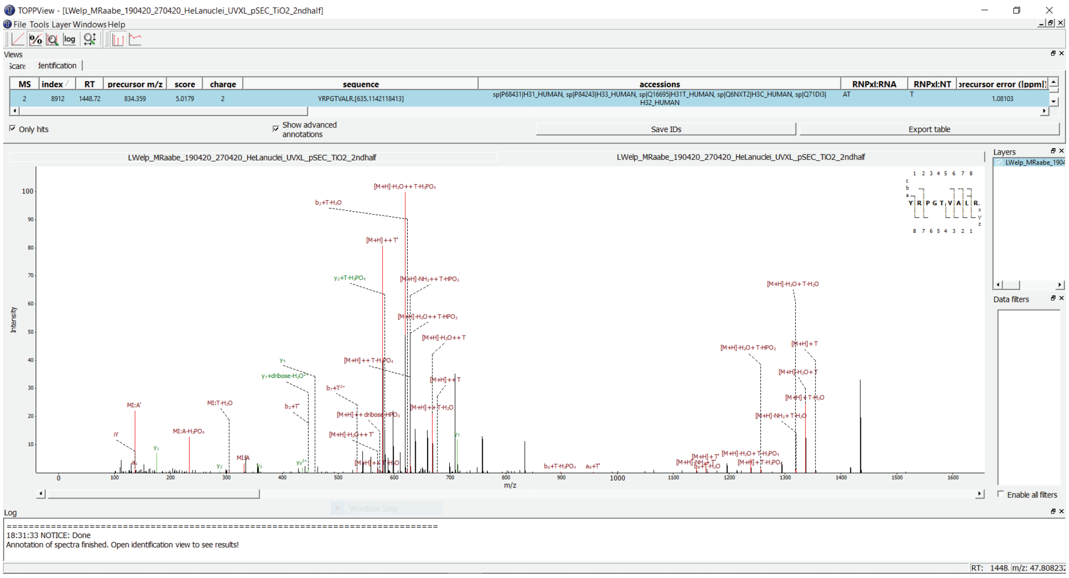
TOPPView - [LWelp_MRaabe_190420_270420_HeLanuclei_UVXL_pSEC_TiO2_2ndhalf]
MS | index | RT | precursor m/z | score | charge | sequence | accessions | RNPxl:RNA | RNPxl:NT | precursor error (ppm)
2 | 8912 | 1448.72 | 834.359 | 5.0179 | 2 | YRPGTVALR.[635.1142118413] | AT | T | 1.08103
Only hits
Show advanced annotations
Save IDs
Export table
18:31:33 NOTICE: Done
Annotation of spectra finished. Open identification view to see results!

14)

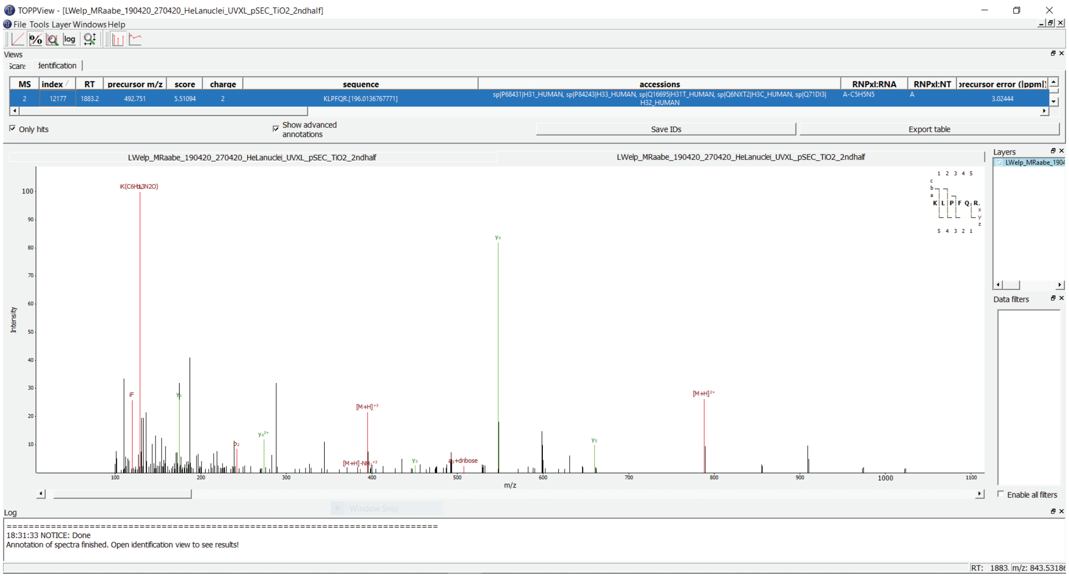
TOPPView - [LWelp_MRaabe_190420_270420_HeLanuclei_UVXL_pSEC_TiO2_2ndhalf]
MS | index | RT | precursor m/z | score | charge | sequence | accessions | RNPxl:RNA | RNPxl:NT | precursor error (ppm)
2 | 12177 | 1883.2 | 492.751 | 5.51094 | 2 | KLPFQR.[196.0136767771] | A-C5H5N5 | A | 3.02444
Only hits
Show advanced annotations
Save IDs
Export table
18:31:33 NOTICE: Done
Annotation of spectra finished. Open identification view to see results!

15)

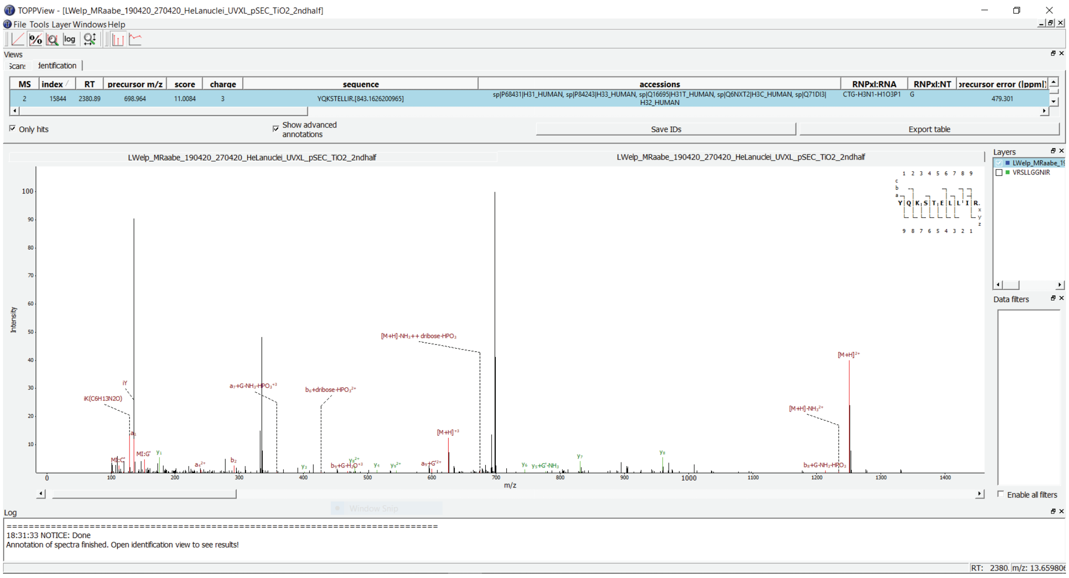
TOPPView - [LWelp_MRaabe_190420_270420_HeLanuclei_UVXL_pSEC_TiO2_2ndhalf]
MS | index | RT | precursor m/z | score | charge | sequence | accessions | RNPxl:RNA | RNPxl:NT | precursor error (ppm)
2 | 15844 | 2380.89 | 698.964 | 11.0084 | 3 | YQKSTELLIR.[843.1626200965] | CTG-H3N1-H1O3P1 | G | 479.301
Only hits
Show advanced annotations
Save IDs
Export table
18:31:33 NOTICE: Done
Annotation of spectra finished. Open identification view to see results!

16) Histone H4

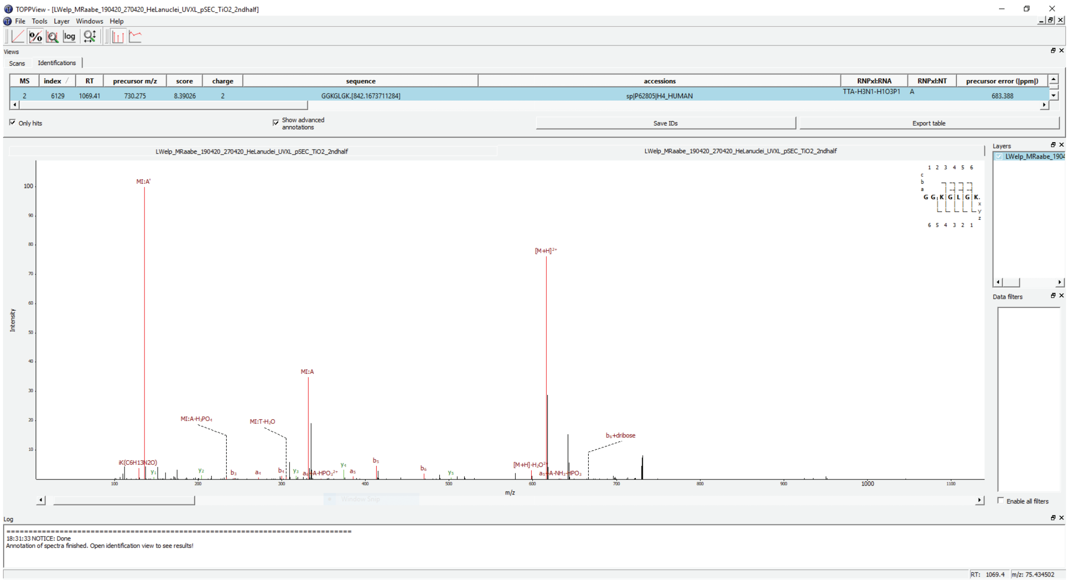

17)

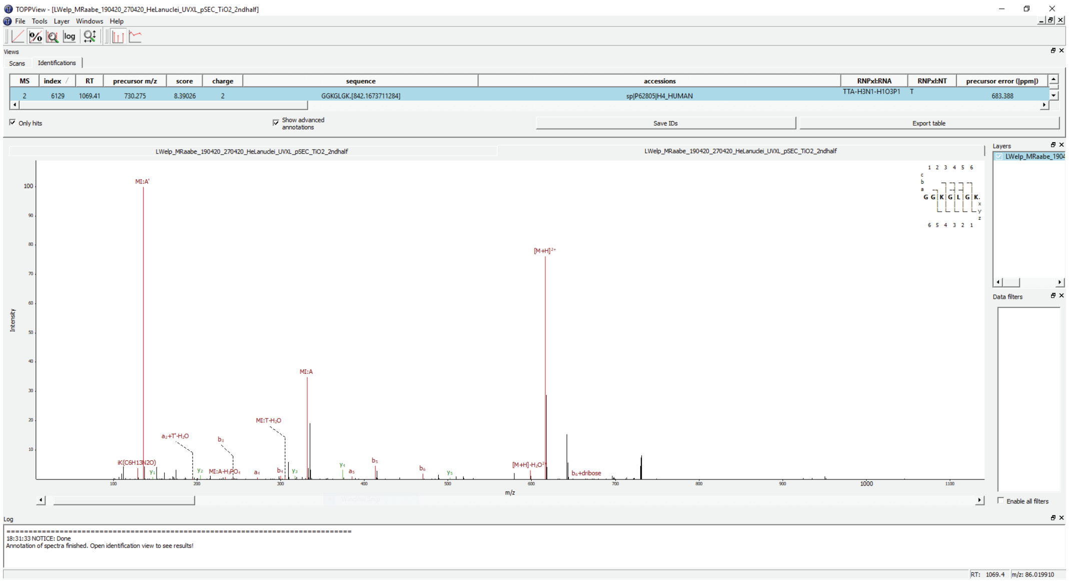

18)

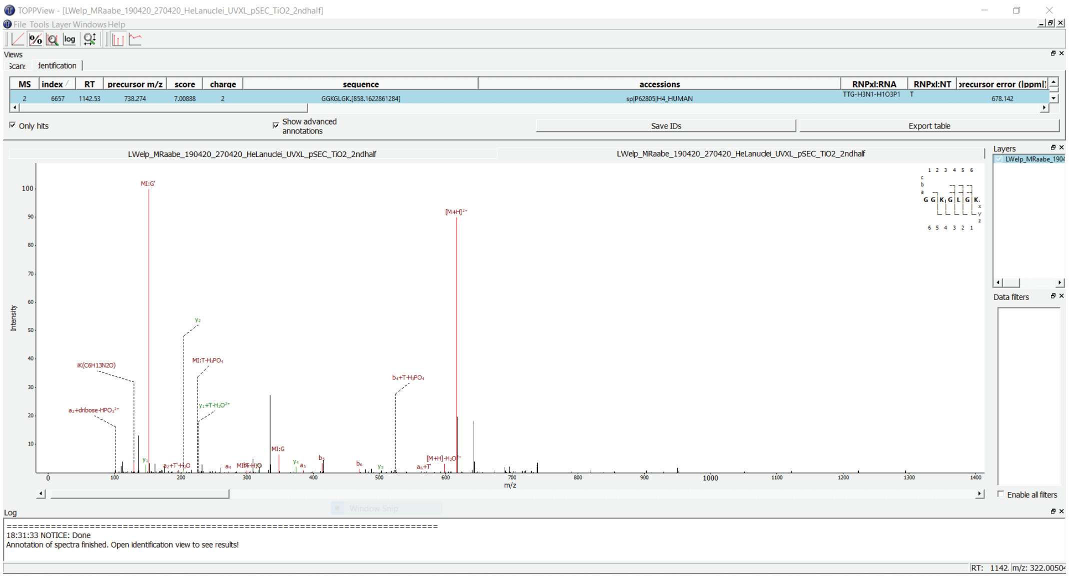

19)

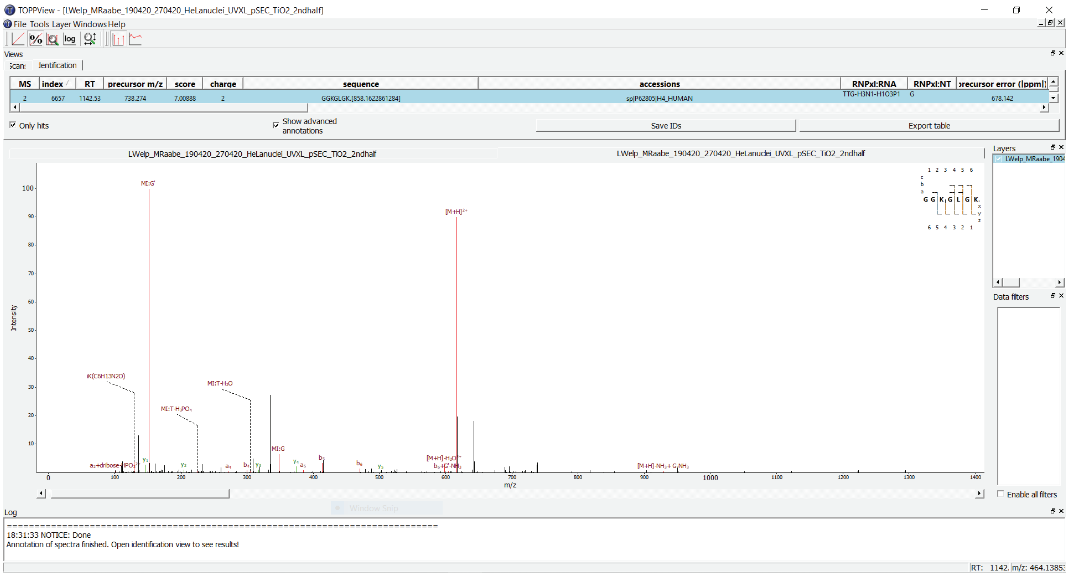
TOPPView - [LWelp_MRaabe_190420_270420_HeLanuclei_UVXL_pSEC_TiO2_2ndhalf]
GGKGLGK.[858.1622861284]
sp|P62805|H4_HUMAN
LWelp_MRaabe_190420_270420_HeLanuclei_UVXL_pSEC_TiO2_2ndhalf
18:31:33 NOTICE: Done
Annotation of spectra finished. Open identification view to see results!

20)

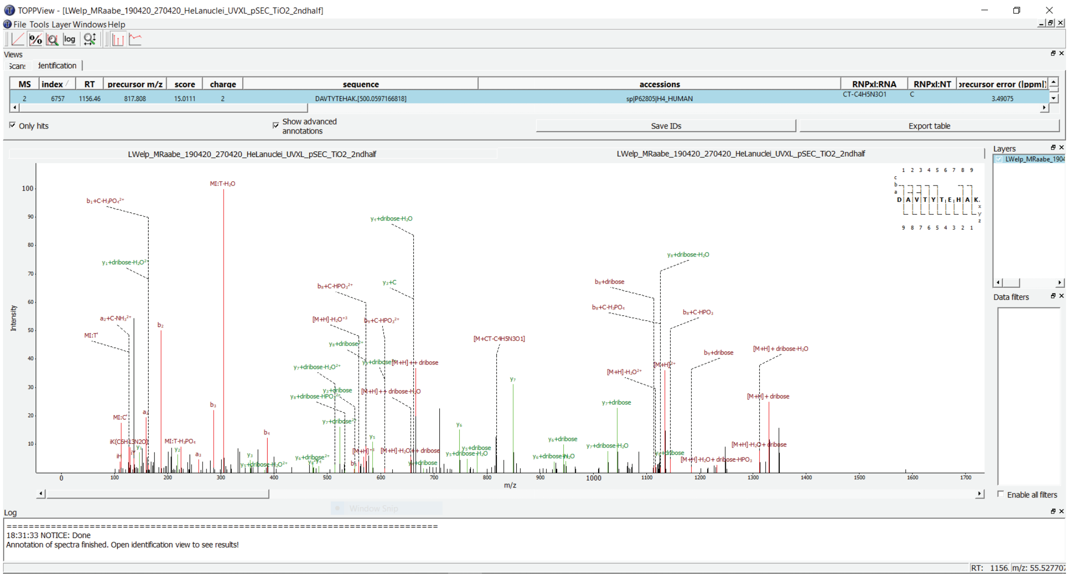
TOPPView - [LWelp_MRaabe_190420_270420_HeLanuclei_UVXL_pSEC_TiO2_2ndhalf]
DAVTYTEHAK.[500.0597166818]
sp|P62805|H4_HUMAN
LWelp_MRaabe_190420_270420_HeLanuclei_UVXL_pSEC_TiO2_2ndhalf
18:31:33 NOTICE: Done
Annotation of spectra finished. Open identification view to see results!

21)

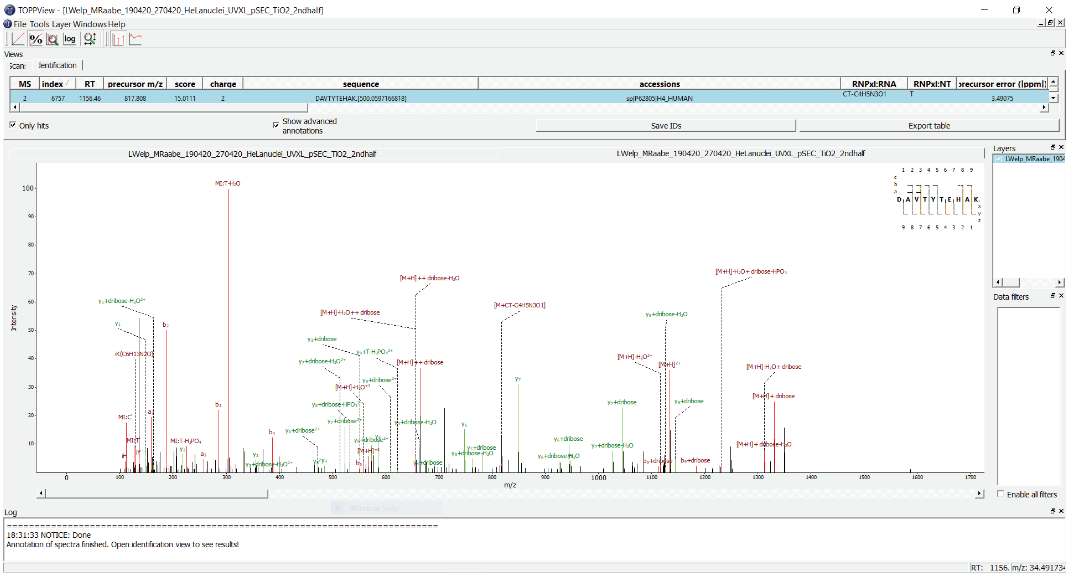
TOPPView - [LWelp_MRaabe_190420_270420_HeLanuclei_UVXL_pSEC_TiO2_2ndhalf]
DAVTYTEHAK.[500.0597166818]
sp|P62805|H4_HUMAN
LWelp_MRaabe_190420_270420_HeLanuclei_UVXL_pSEC_TiO2_2ndhalf
18:31:33 NOTICE: Done
Annotation of spectra finished. Open identification view to see results!

22)

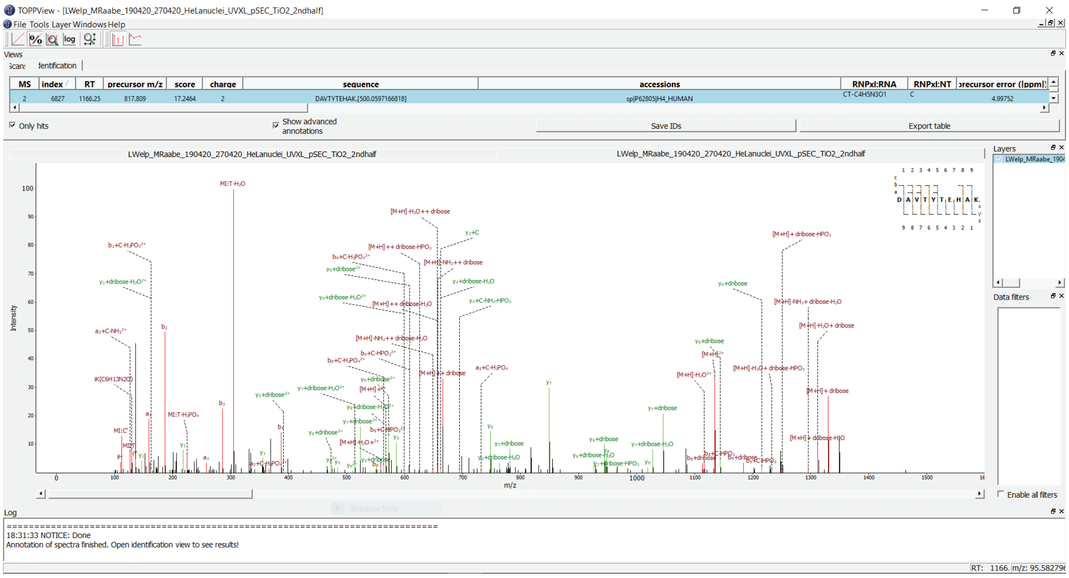

23)

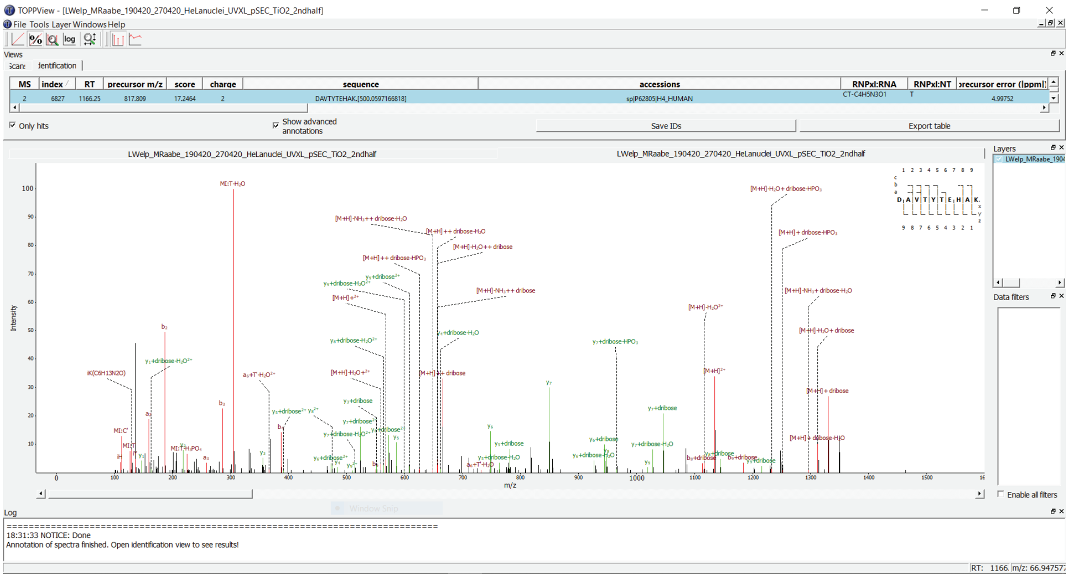

24) Histone-lysine N-methyltransferase 2C

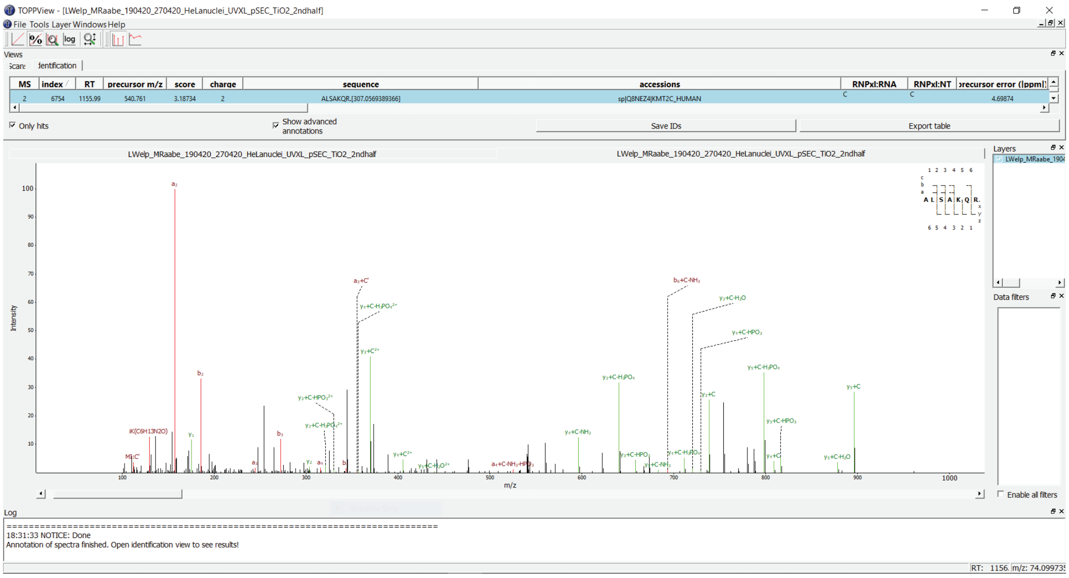

25) RNA-binding motif protein, X-linked-like-2

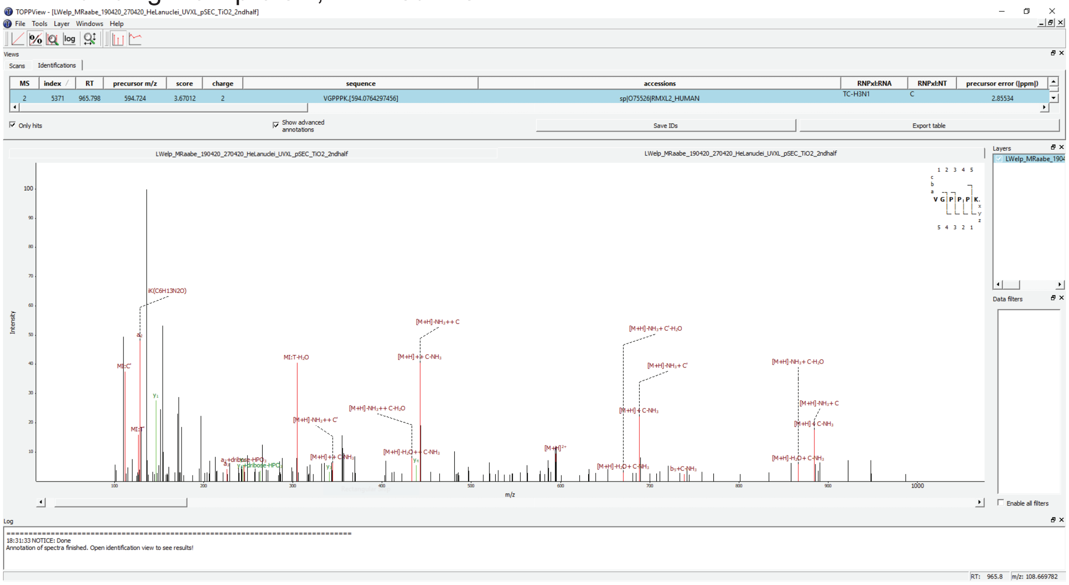

26) Single-stranded DNA-binding protein

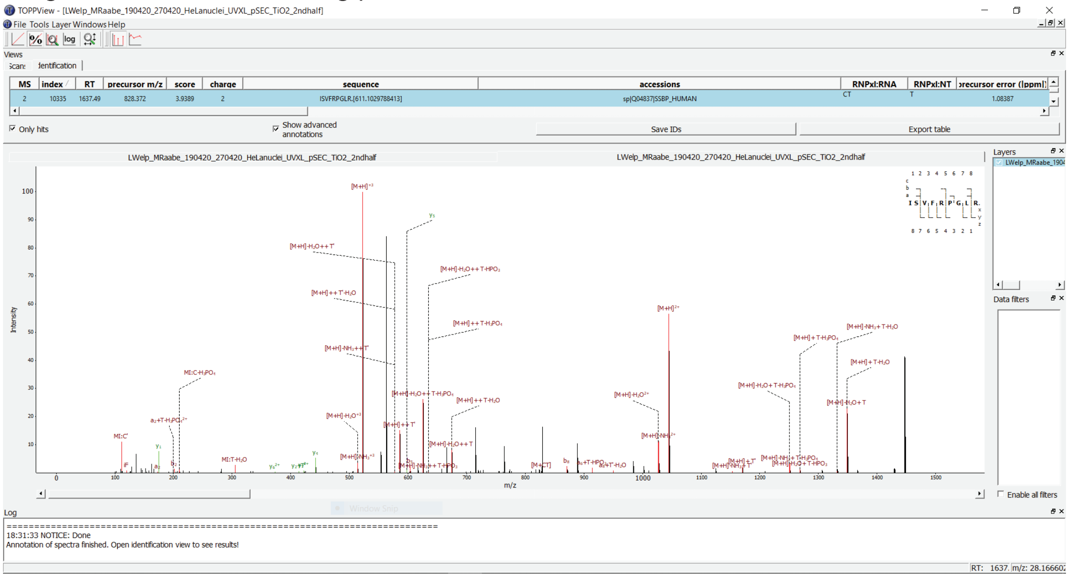

27)

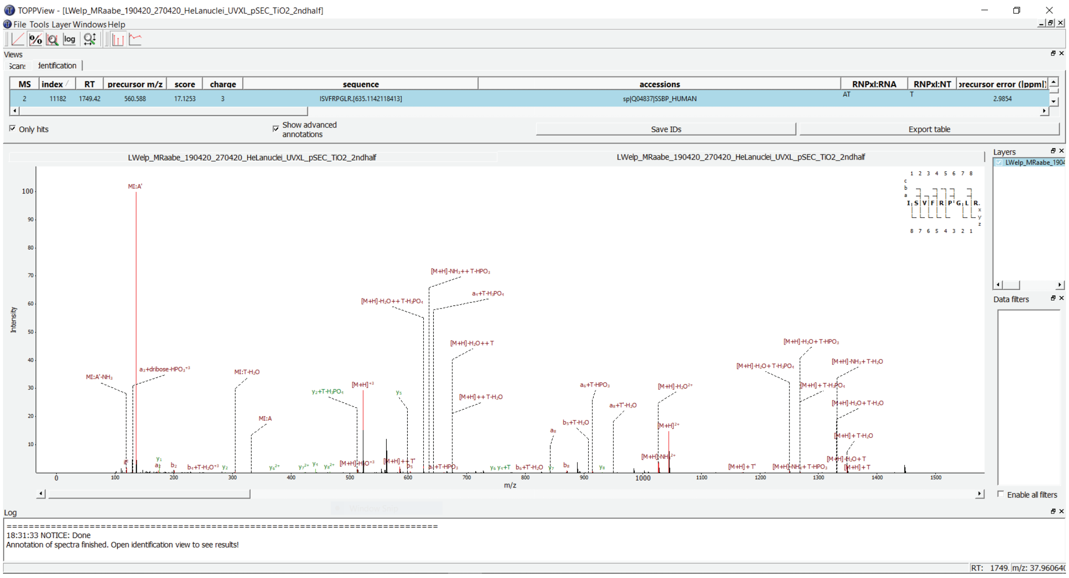

28)

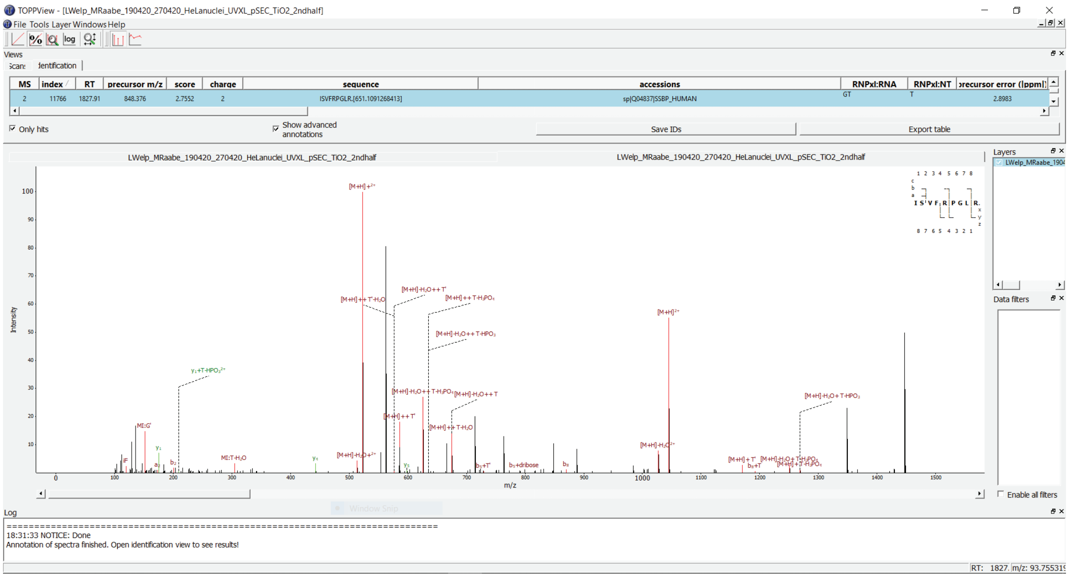

29)

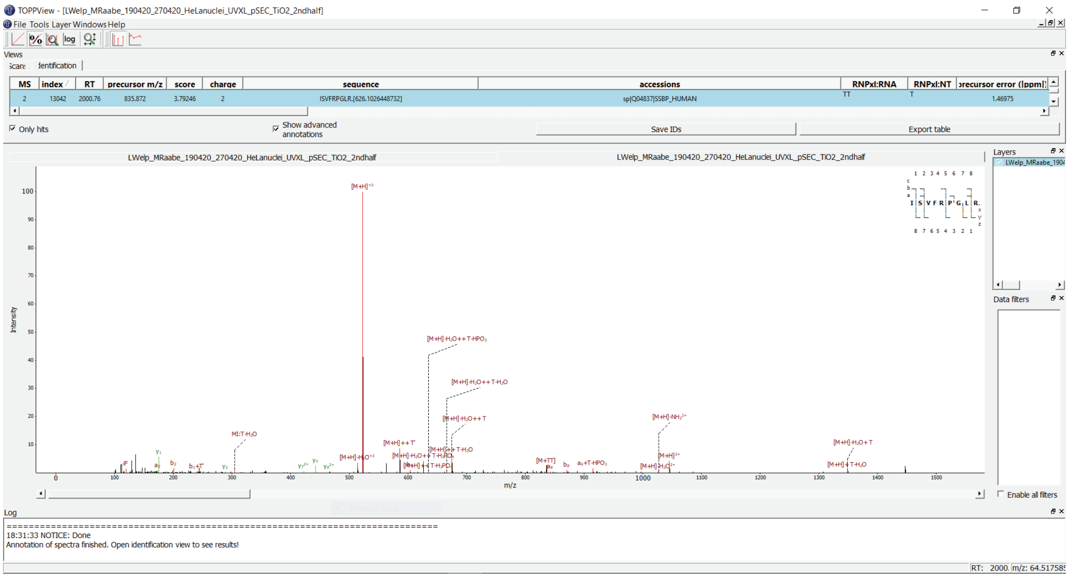

30) Titin

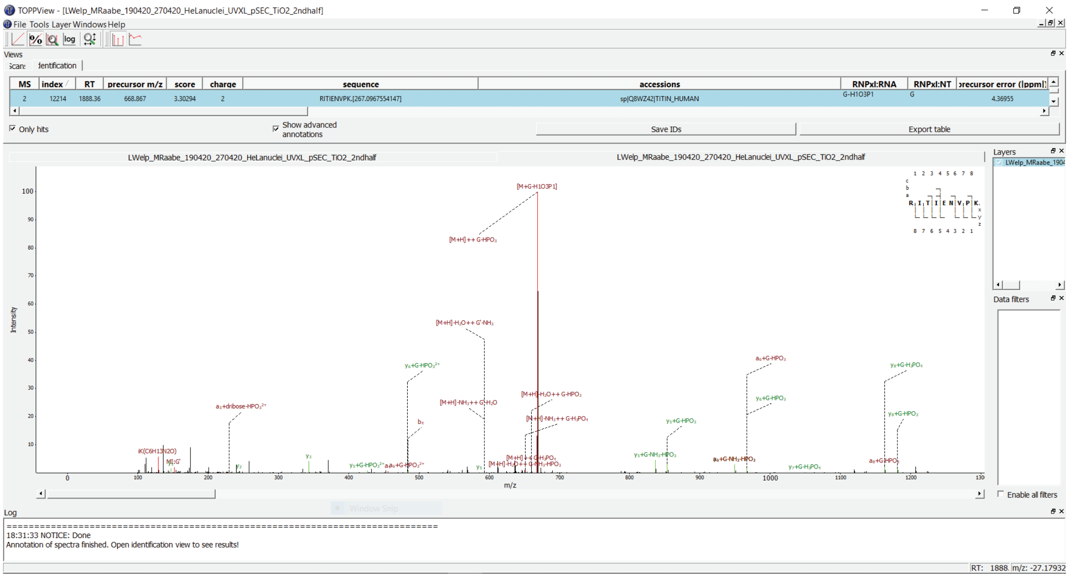

TOPPView spectra - deoxyribose cross-link spectrum matches

UV cross-linked HeLa nuclei (SEC)

1) Heterogeneous nuclear ribonucleoproteins C1/C2

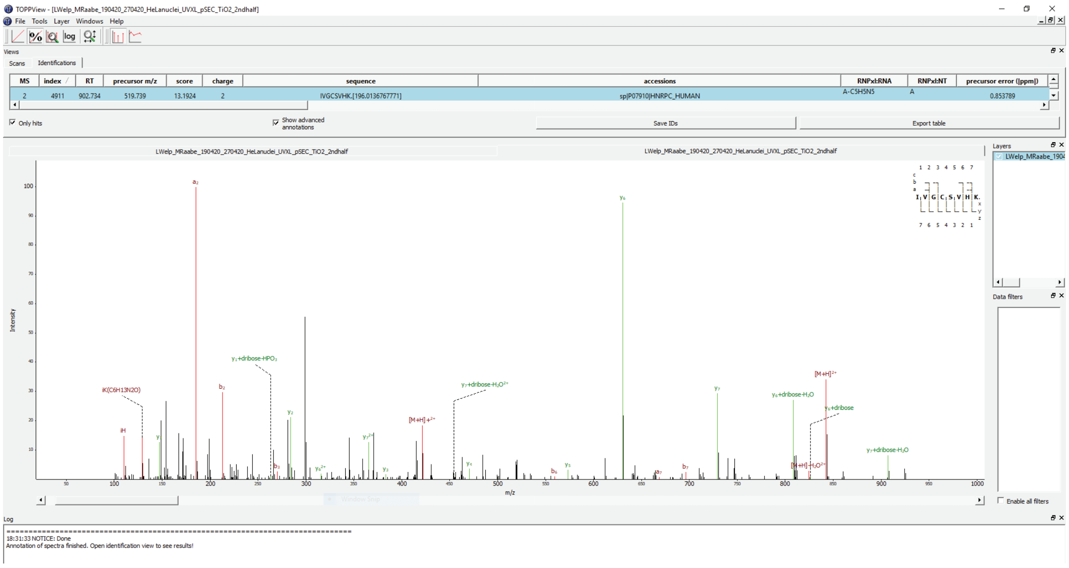

2)

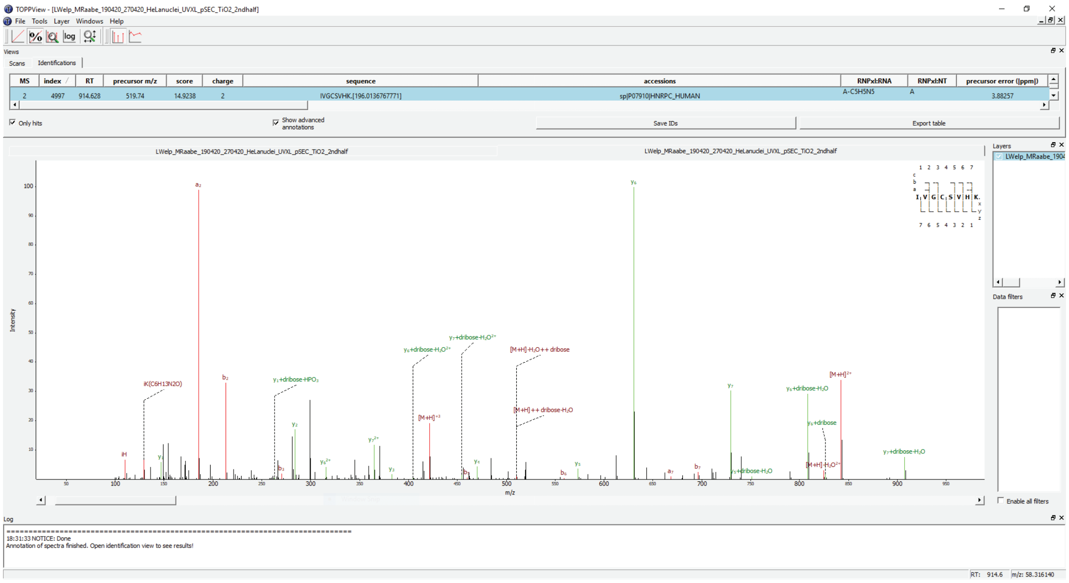

3) Tubulin alpha-3C chain

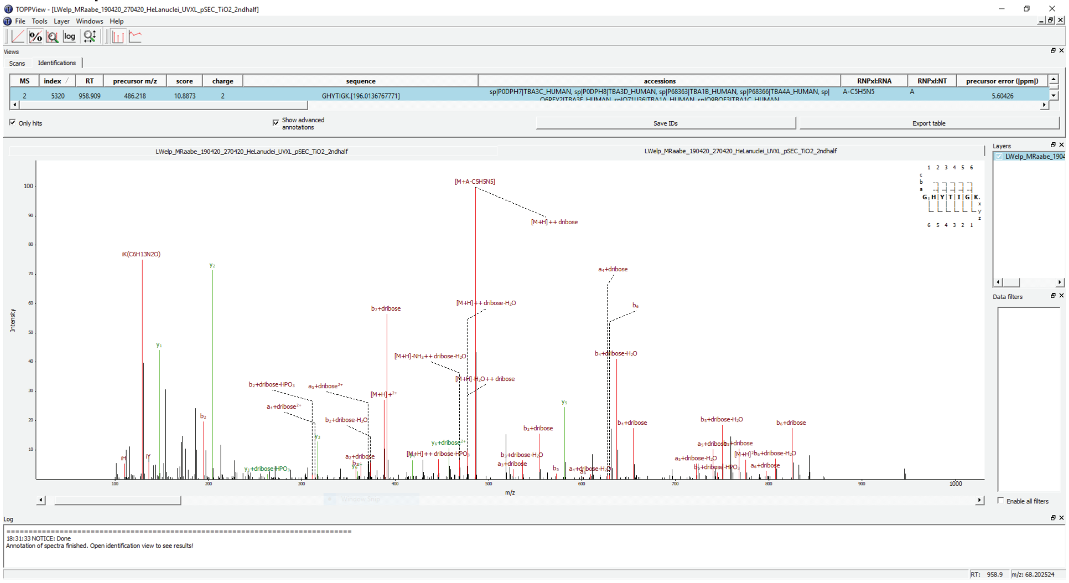

4)

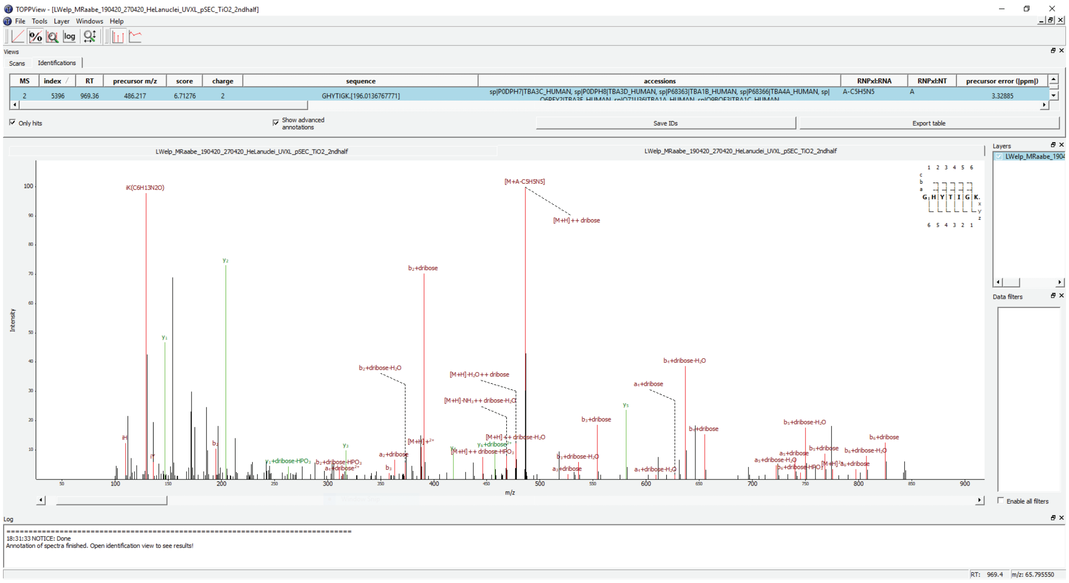
TOPPView - [LWelp_MRaabe_190420_270420_HeLanuclei_UVXL_pSEC_TiO2_2ndhalf]
MS | index | RT | precursor m/z | score | charge | sequence | accessions | RNPxl:RNA | RNPxl:NT | precursor error (|ppm|)
2 | 5396 | 969.36 | 486.217 | 6.71276 | 2 | GHYTIGK.[196.0136767771] | A-C5H5N5 | A | 3.32885
Only hits
Show advanced annotations
Save IDs
Export table
Layers
Data filters
Enable all filters
Log
18:31:33 NOTICE: Done
Annotation of spectra finished. Open identification view to see results!

5) Actin

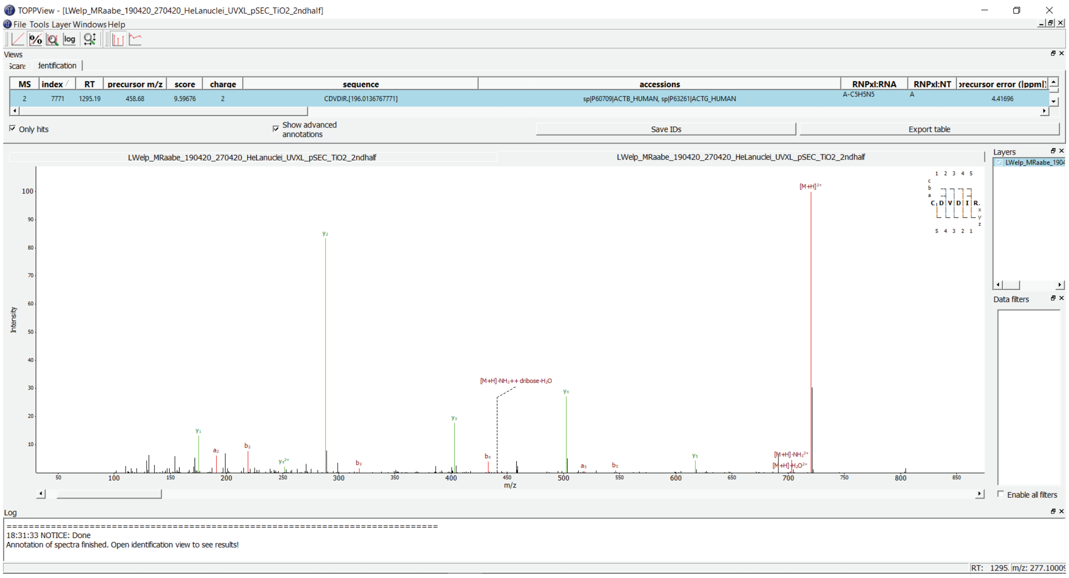
TOPPView - [LWelp_MRaabe_190420_270420_HeLanuclei_UVXL_pSEC_TiO2_2ndhalf]
MS | index | RT | precursor m/z | score | charge | sequence | accessions | RNPxl:RNA | RNPxl:NT | precursor error (|ppm|)
2 | 7771 | 1295.19 | 458.68 | 9.59676 | 2 | CDVDIR.[196.0136767771] | sp|P60709|ACTB_HUMAN, sp|P63261|ACTG_HUMAN | A-C5H5N5 | A | 4.41696
Only hits
Show advanced annotations
Save IDs
Export table
Layers
Data filters
Enable all filters
Log
18:31:33 NOTICE: Done
Annotation of spectra finished. Open identification view to see results!

6)

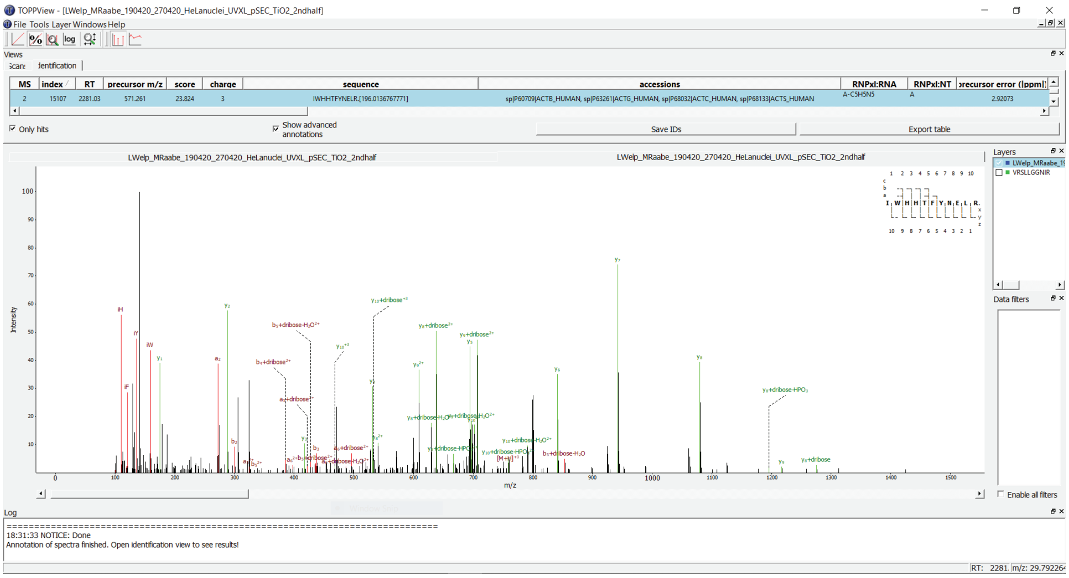
TOPPView - [LWelp_MRaabe_190420_270420_HeLanuclei_UVXL_pSEC_TiO2_2ndhalf]
MS | index | RT | precursor m/z | score | charge | sequence | accessions | RNPxl:RNA | RNPxl:NT | precursor error (|ppm|)
2 | 15107 | 2281.03 | 571.261 | 23.824 | 3 | IWHHTFYNELR.[196.0136767771] | sp|P60709|ACTB_HUMAN, sp|P63261|ACTG_HUMAN, sp|P68032|ACTC_HUMAN, sp|P68133|ACTS_HUMAN | A-C5H5N5 | A | 2.92073
Only hits
Show advanced annotations
Save IDs
Export table
Layers
VRSLLGGNIR
Data filters
Enable all filters
Log
18:31:33 NOTICE: Done
Annotation of spectra finished. Open identification view to see results!

7) Clathrin heavy chain 1

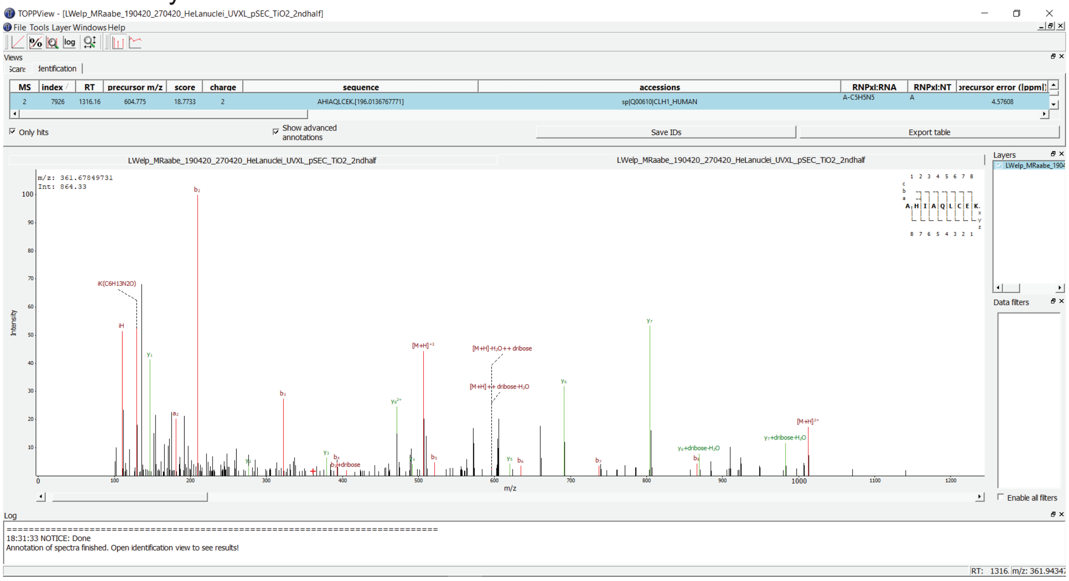

8) Carbamoyl-phosphate synthase

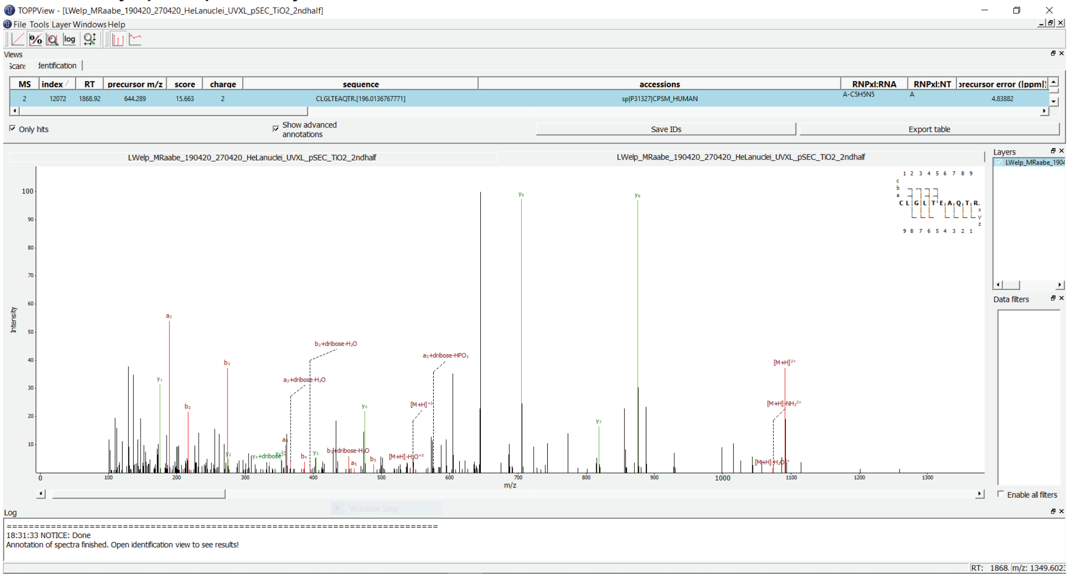

9)

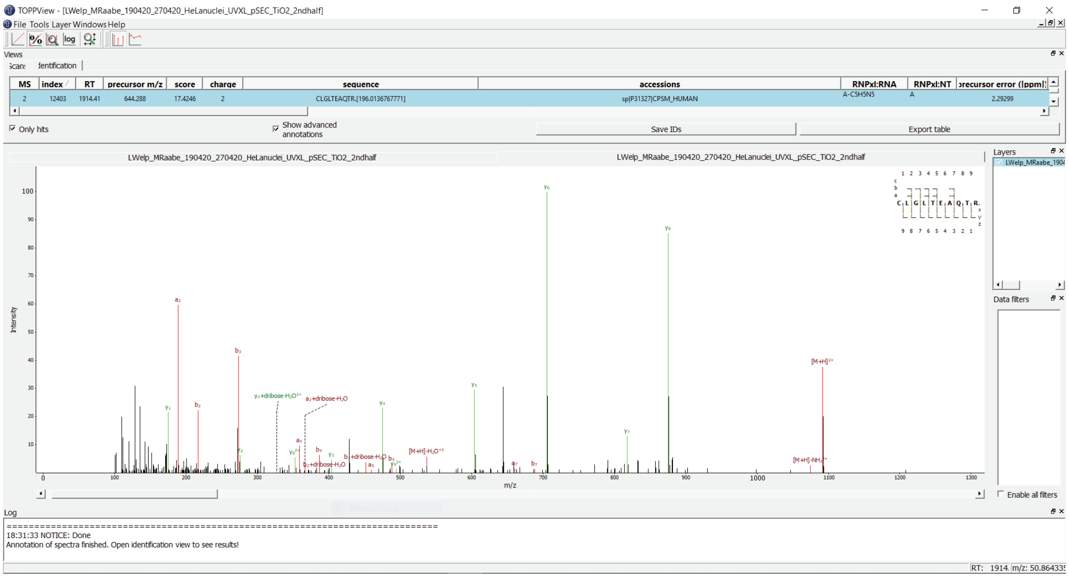

10) Complement decay-accelerating factor

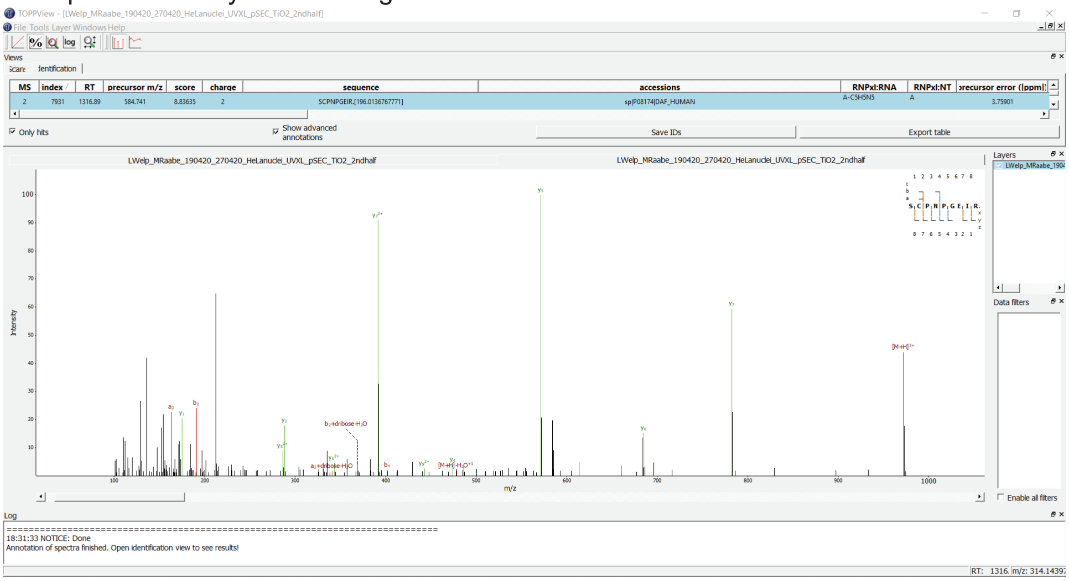

11) Elongation factor 1-alpha 1

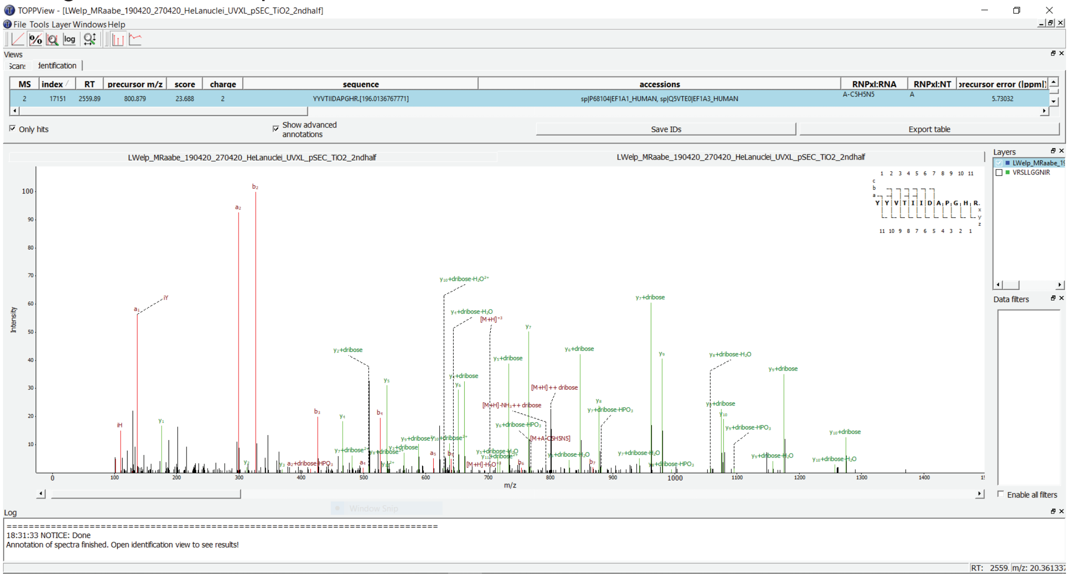

12) Alpha-enolase

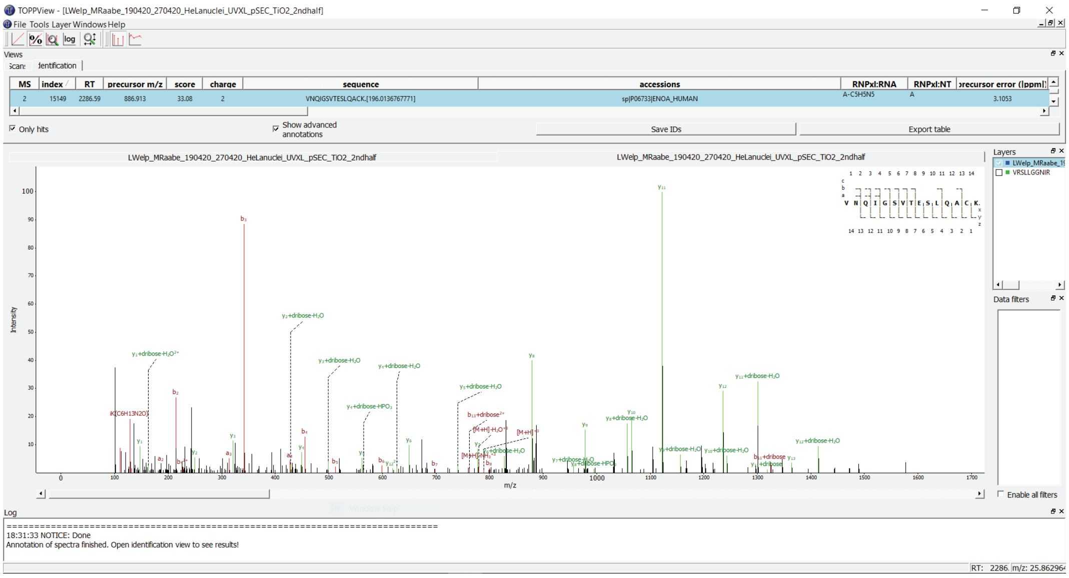

13) Glyceraldehyde-3-phosphate dehydrogenase

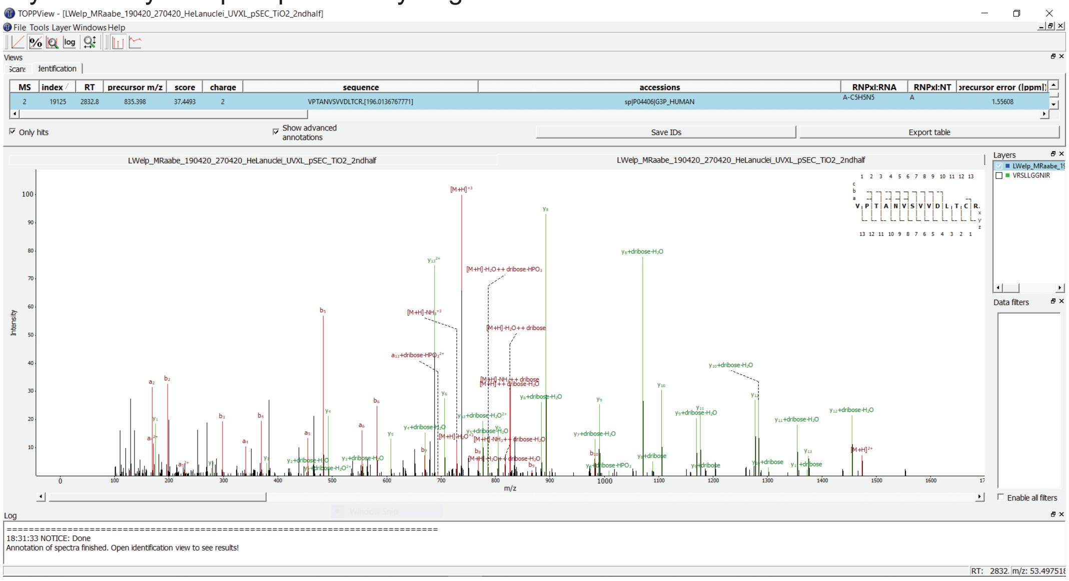

14)

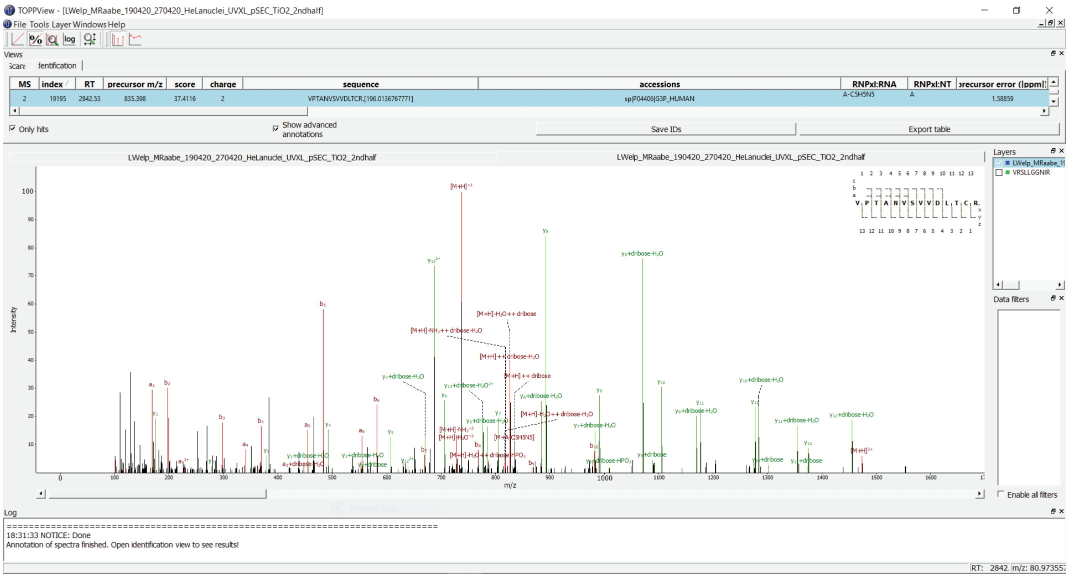

15) Heterogeneous nuclear ribonucleoprotein U

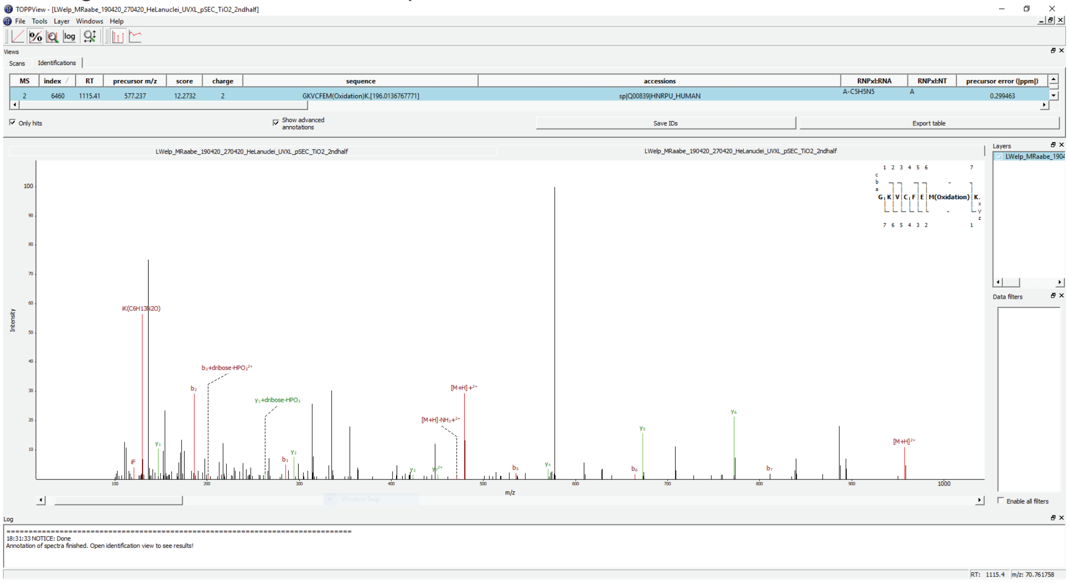

16) Heat shock protein HSP 90-alpha

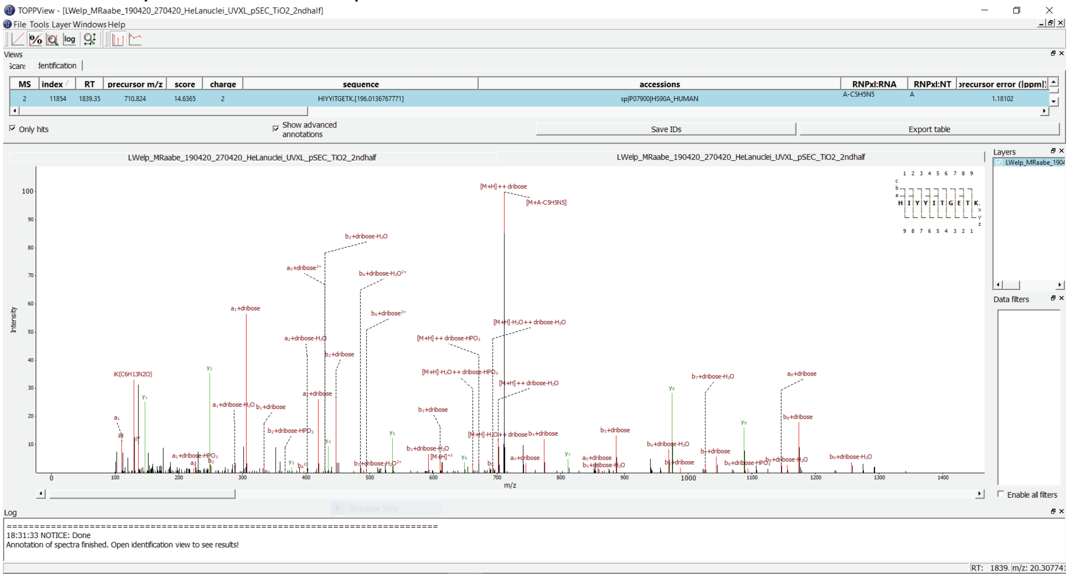

17) Heat shock protein HSP 90-beta

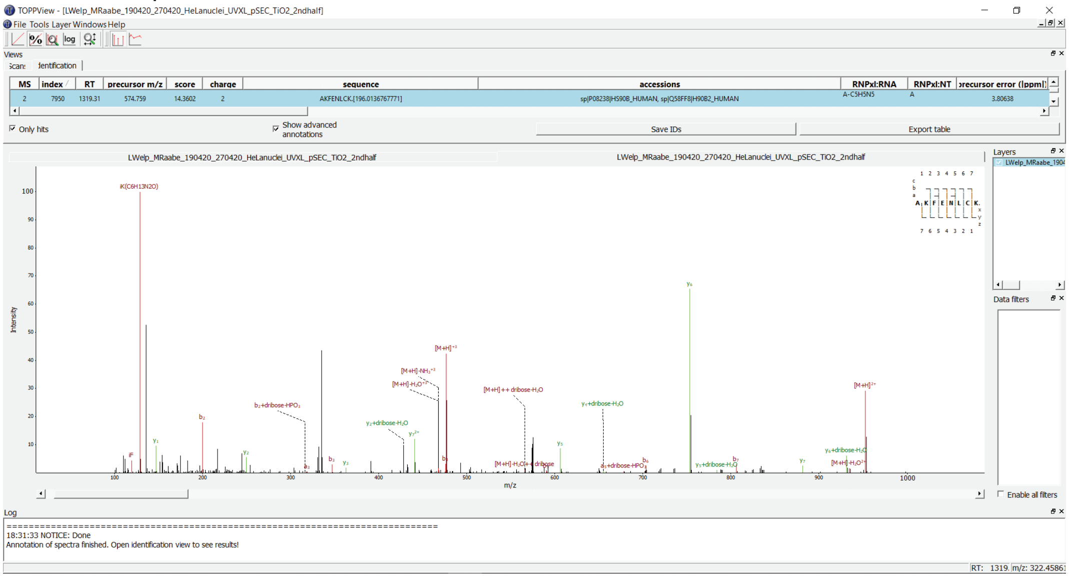

18) Heat shock cognate 71 kDa protein

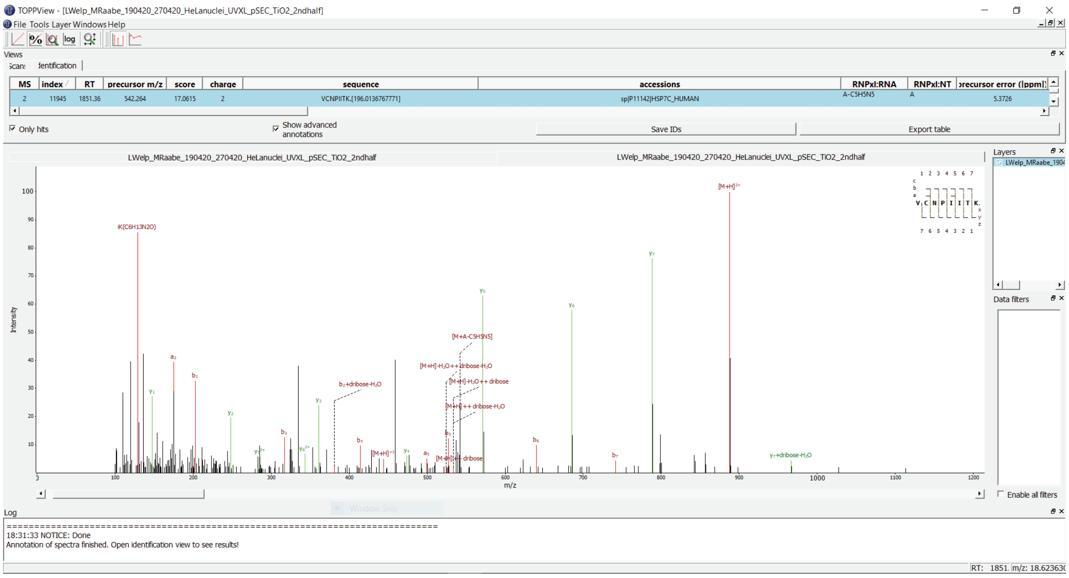

19) Pyruvate kinase PKM

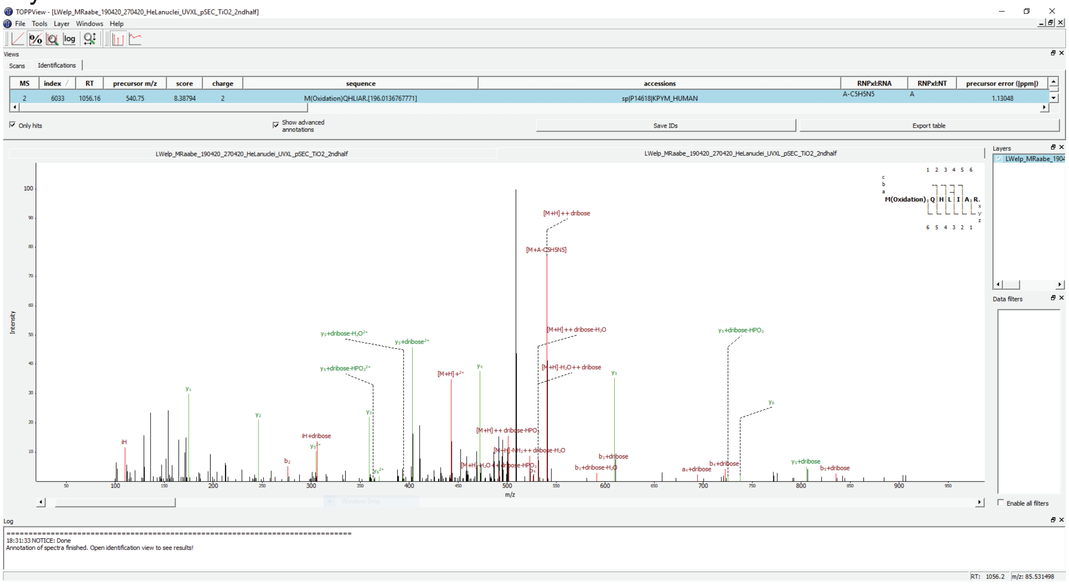

20) L-lactate dehydrogenase A chain

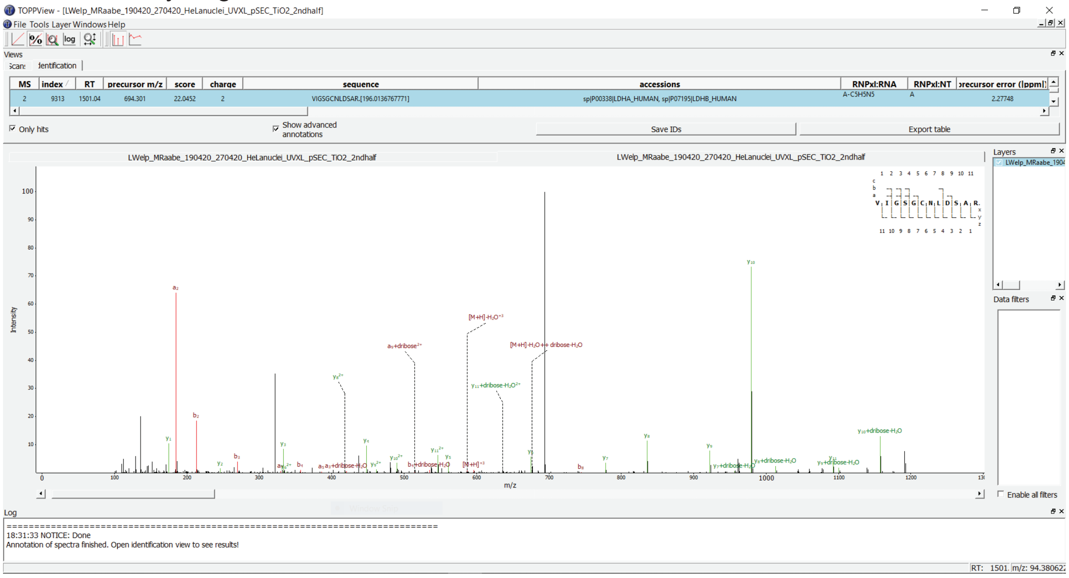

21)

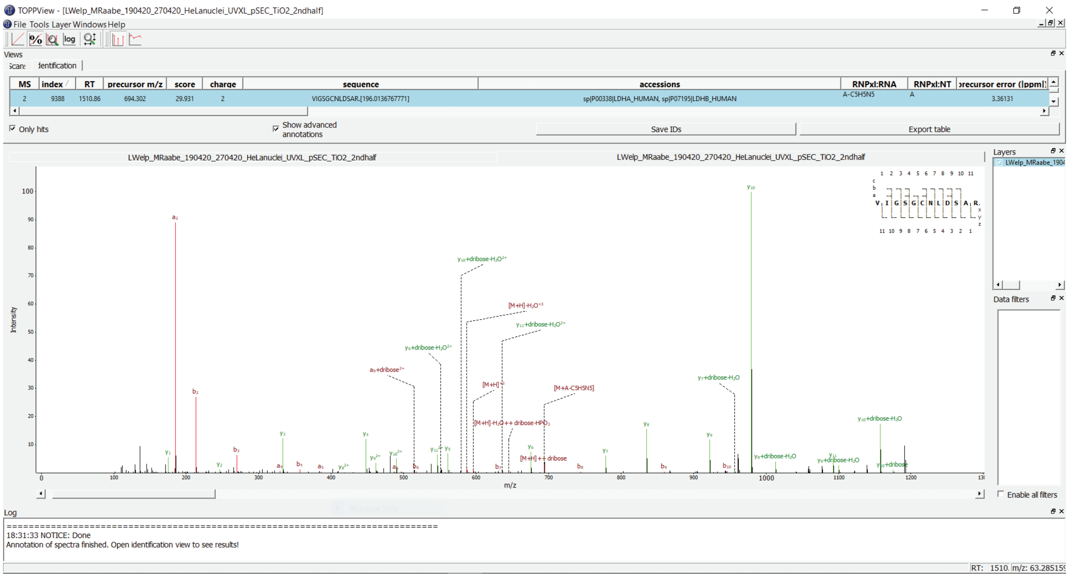

22) L-lactate dehydrogenase B chain

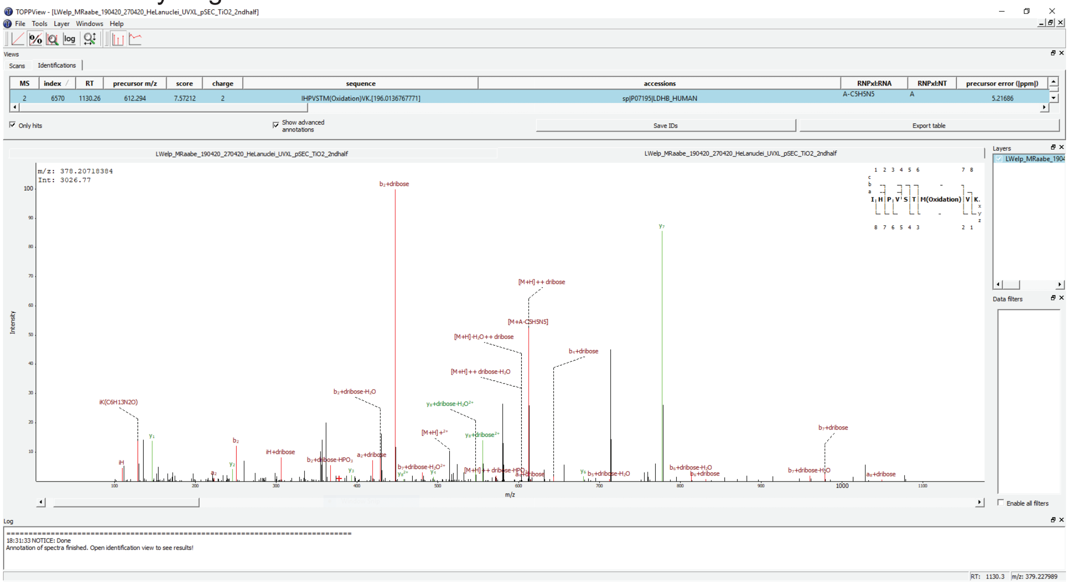

23) Proliferating cell nuclear antigen

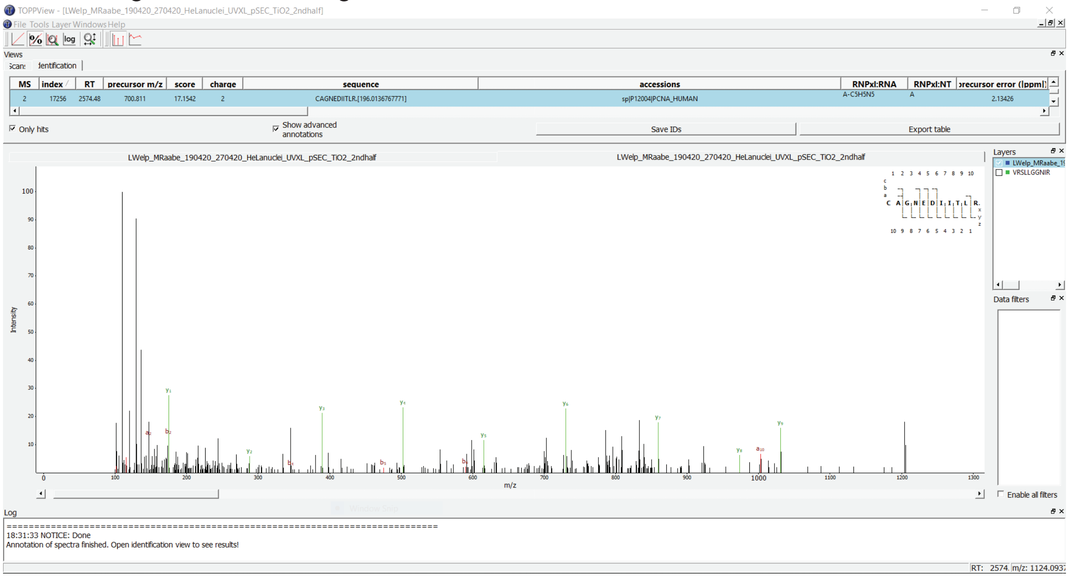

24) Peptidyl-prolyl cis-trans isomerase H

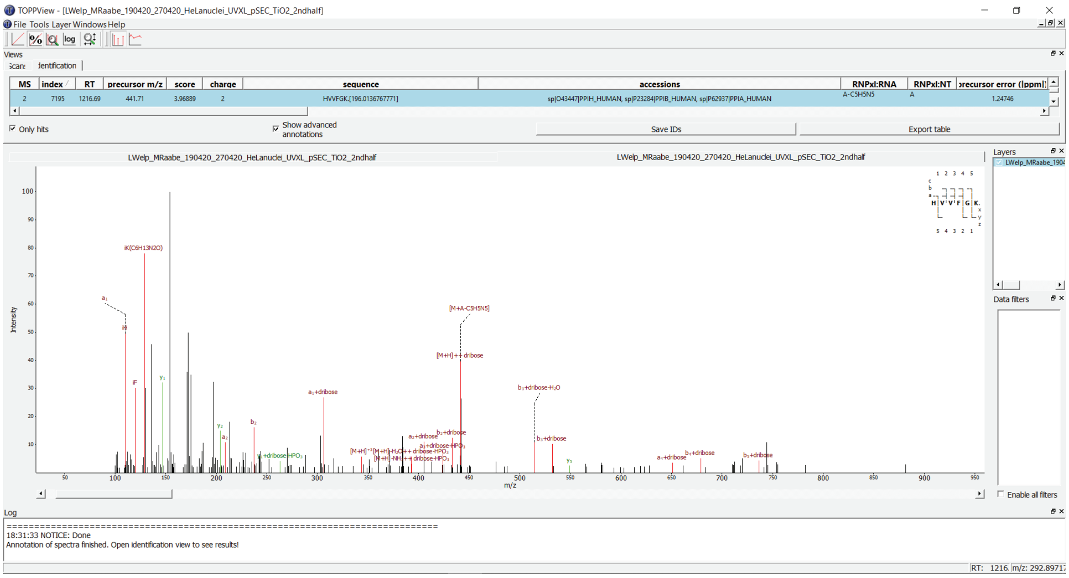

25) GTP-binding nuclear protein Ran

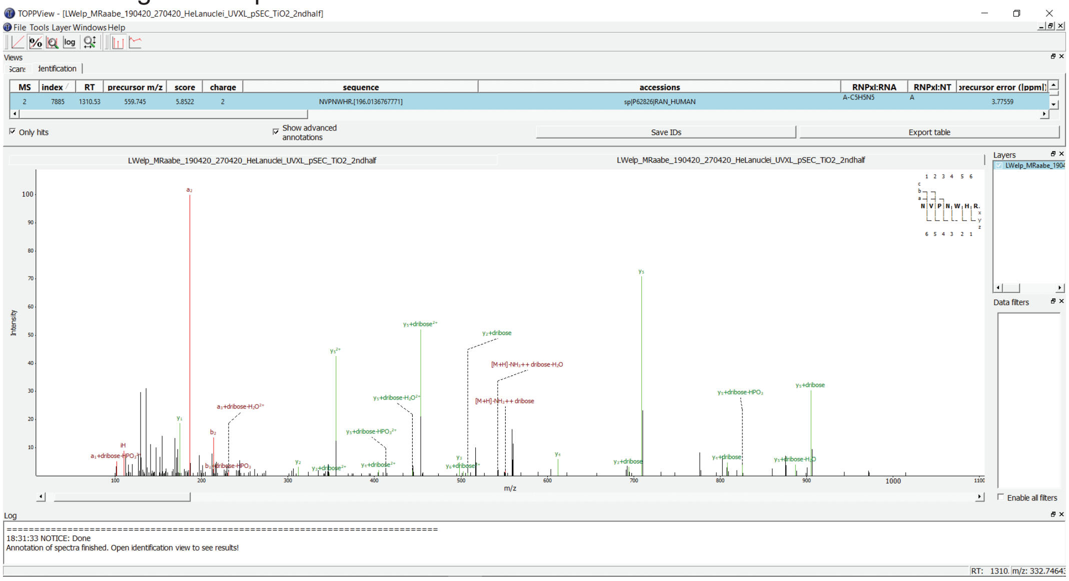

26)

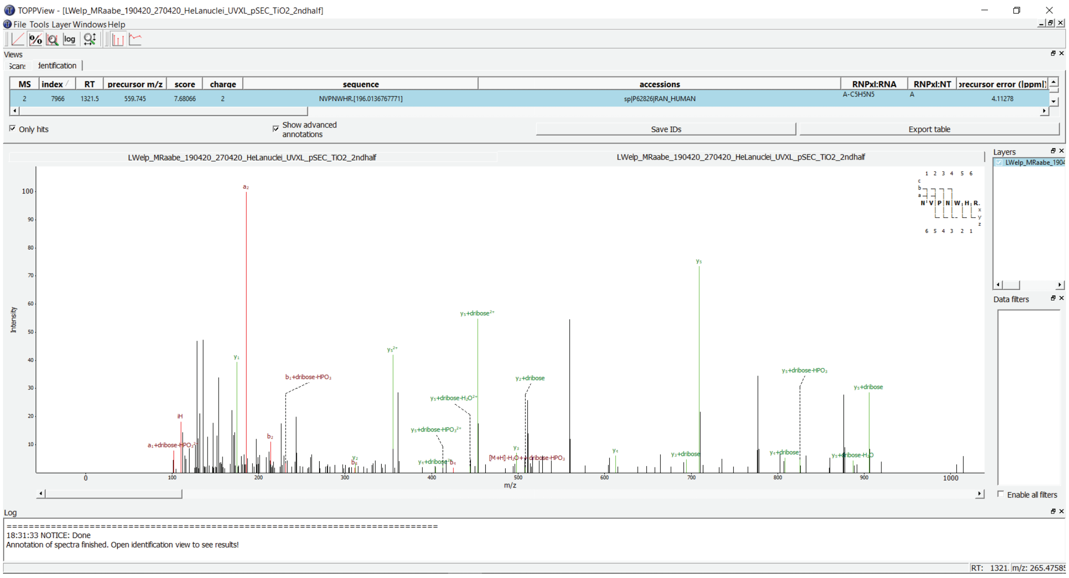

27) 60S ribosomal protein L11

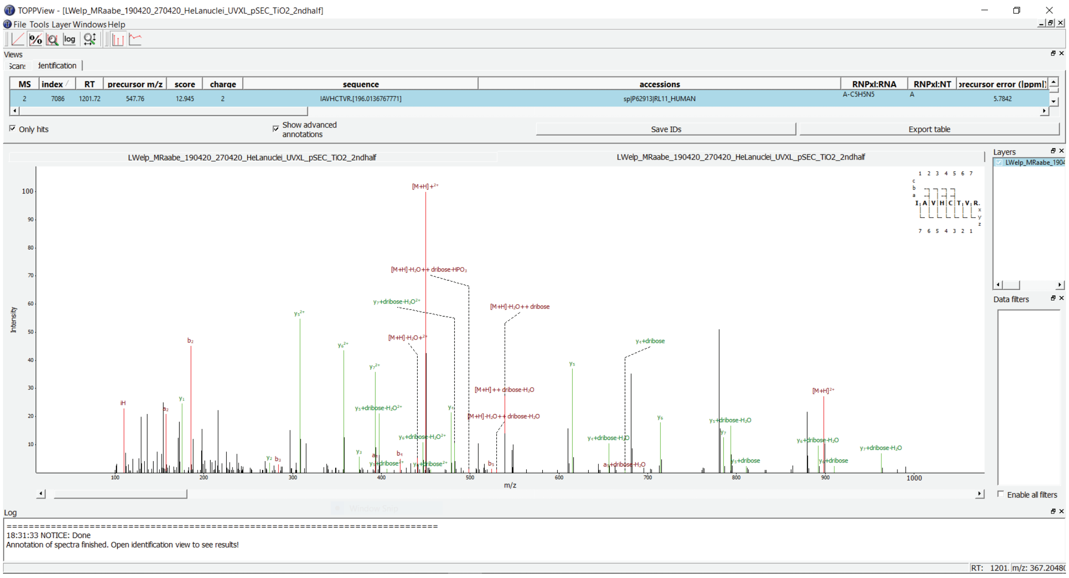

28)

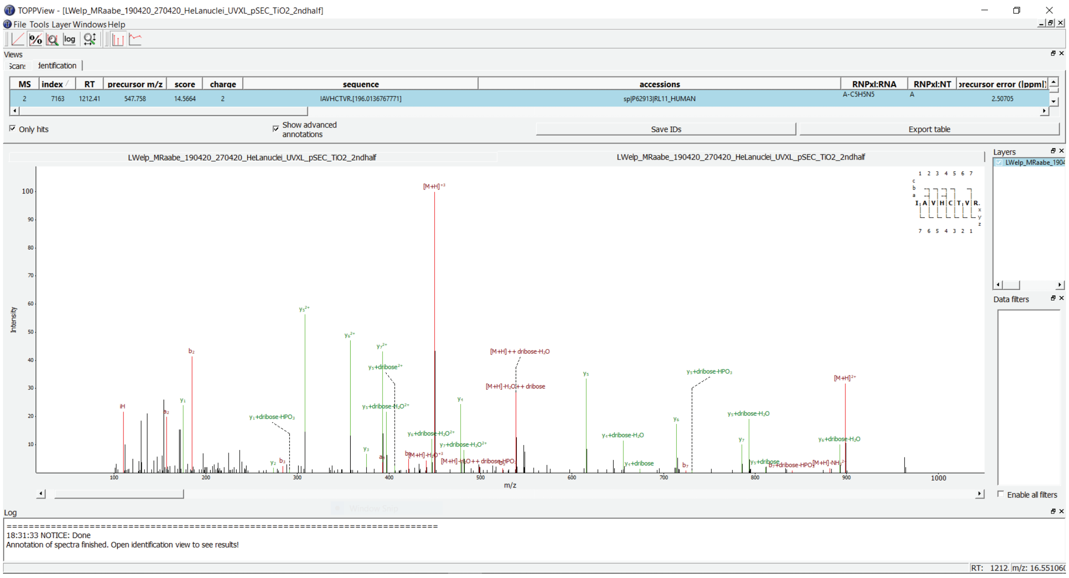

29)

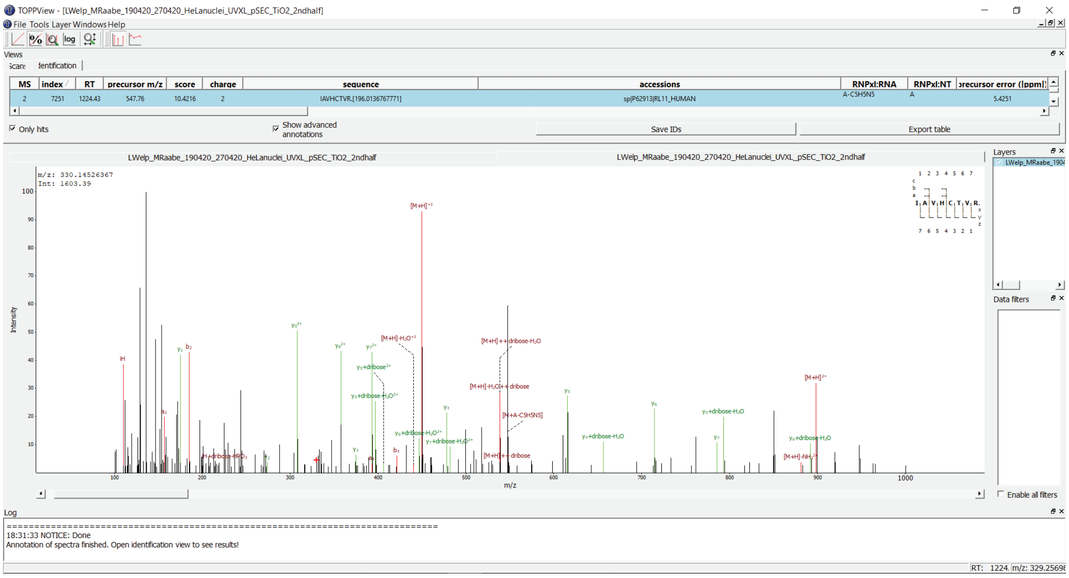

30) 60S ribosomal protein L12

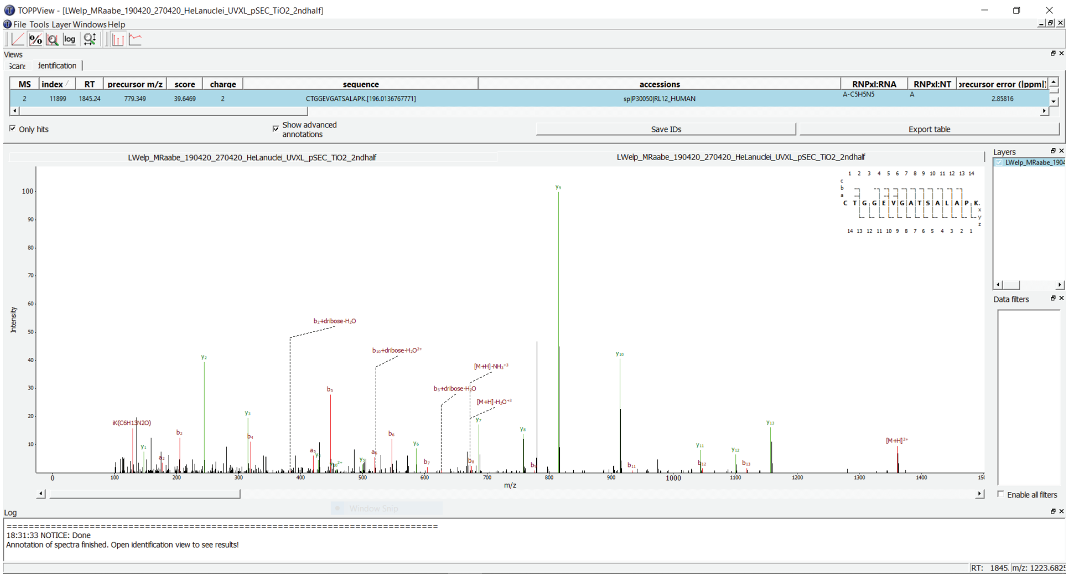

31) 60S ribosomal protein L13a

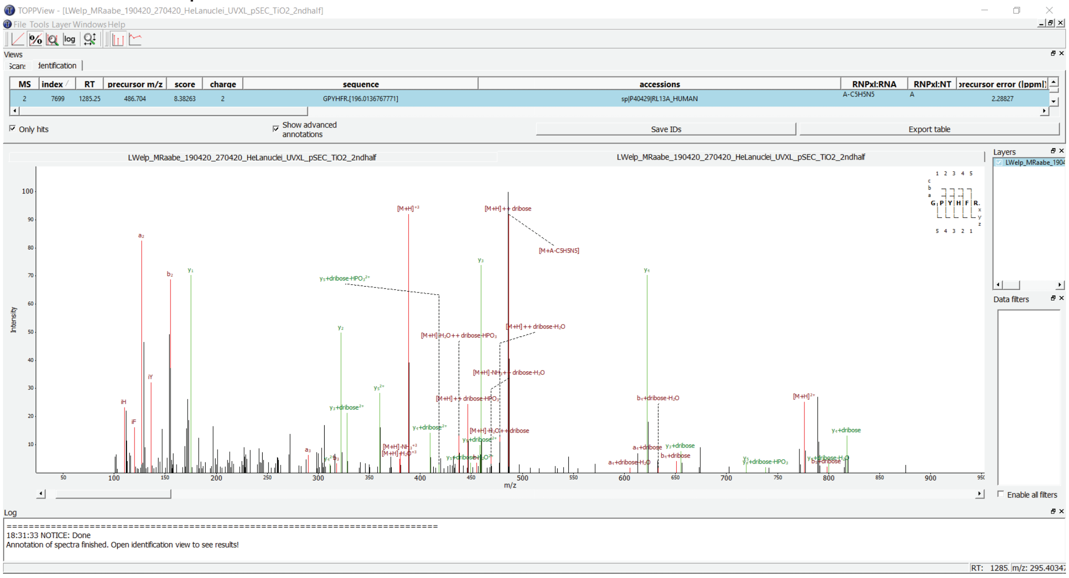

32) 60S ribosomal protein L14

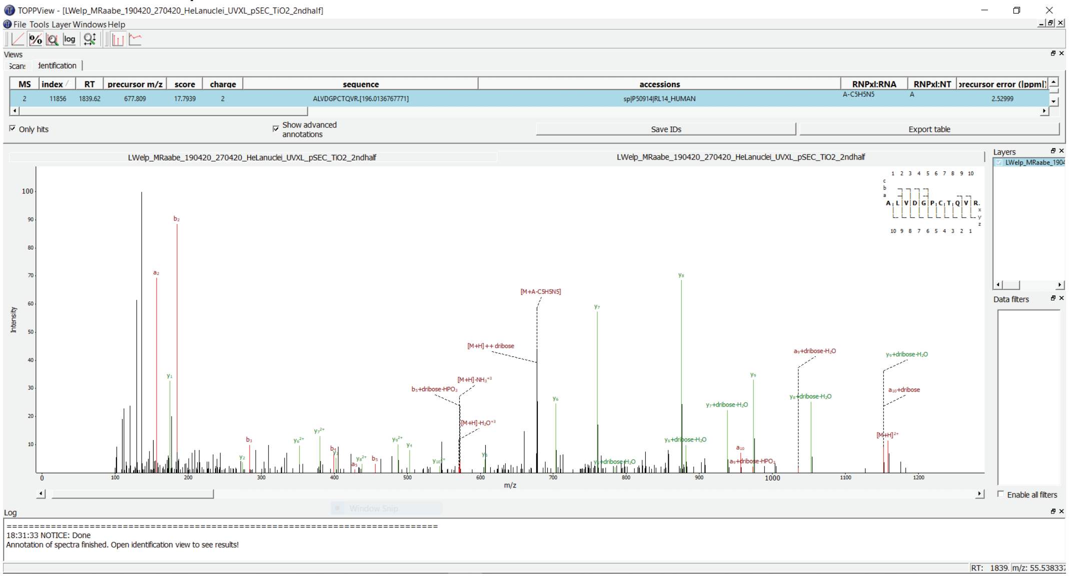

33) 60S ribosomal protein L18

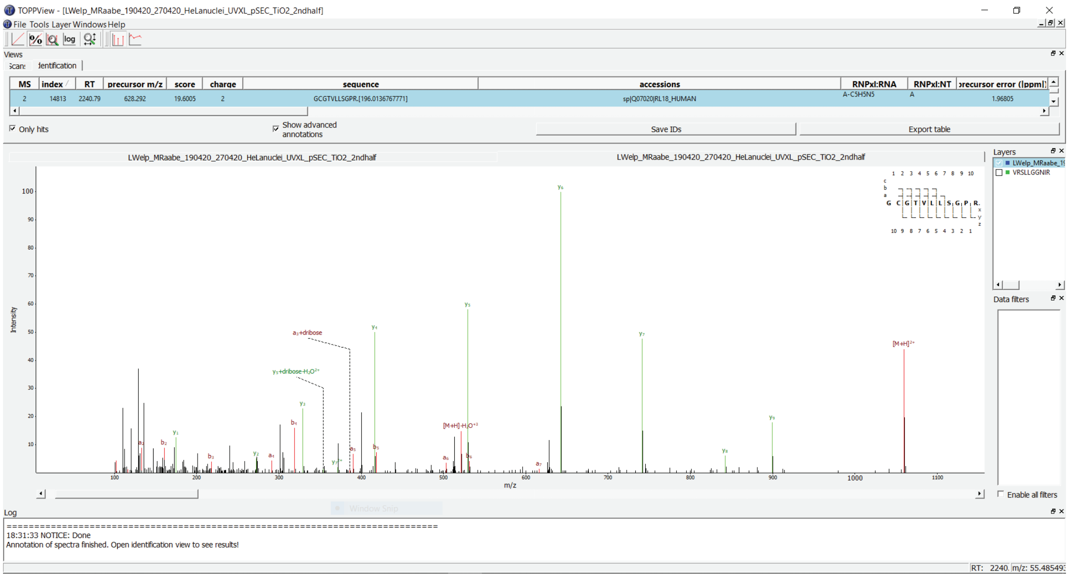

34) 60S ribosomal protein L3

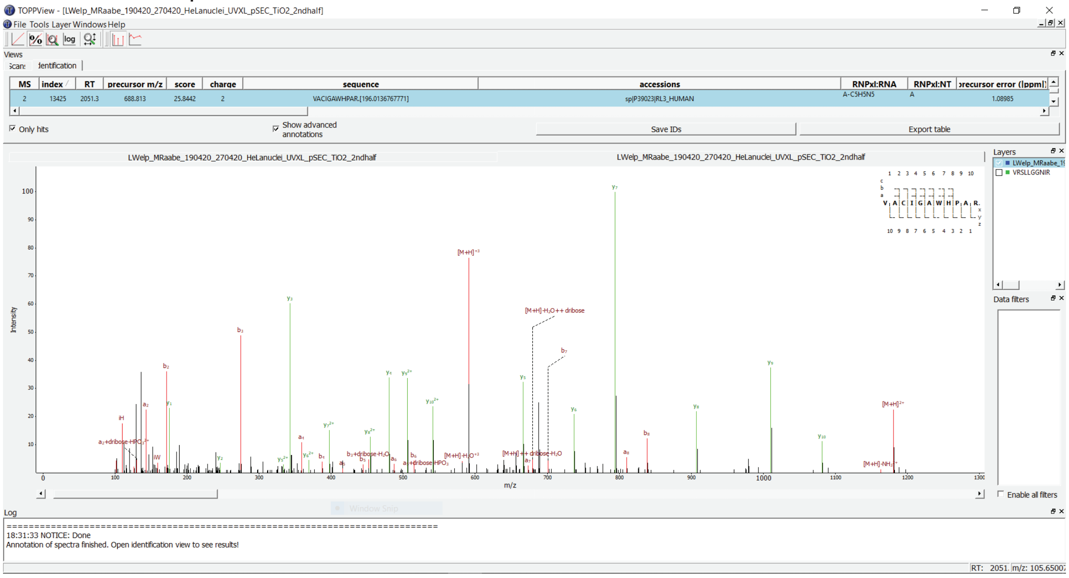

35) 60S ribosomal protein L36

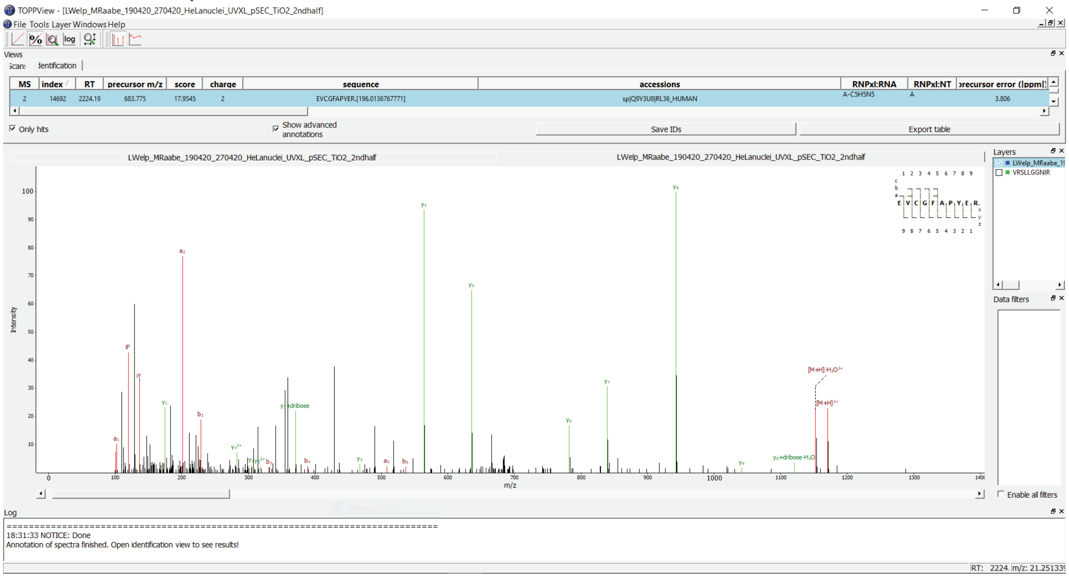

36) 40S ribosomal protein S19

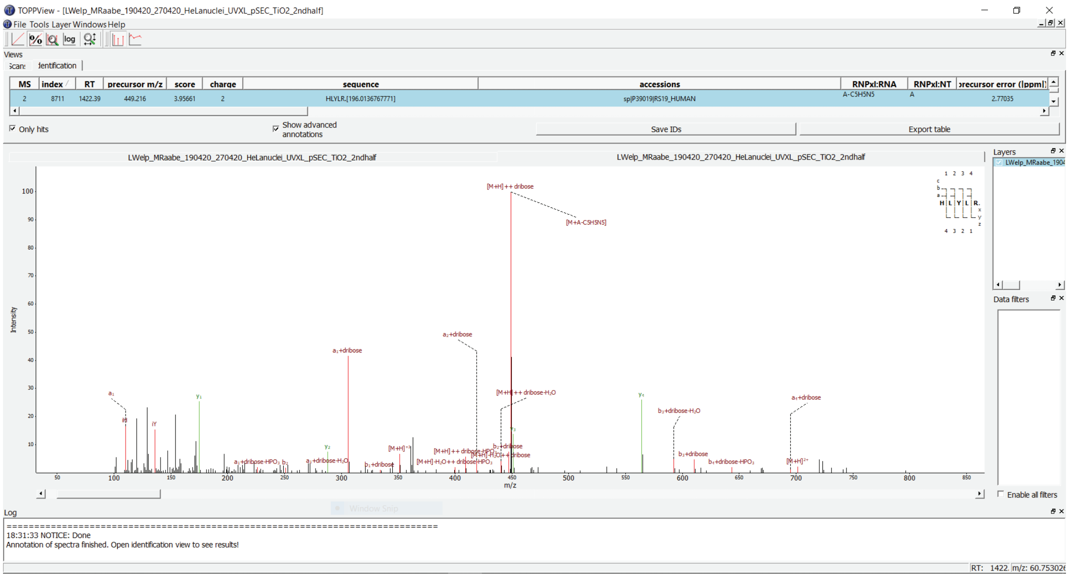

37) 40S ribosomal protein S27

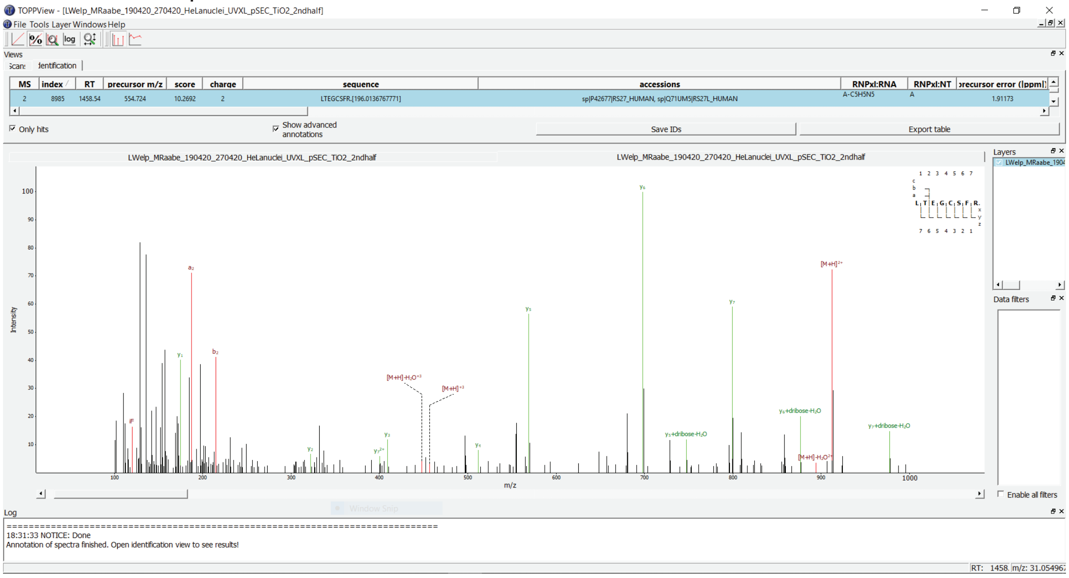

38) 40S ribosomal protein S3

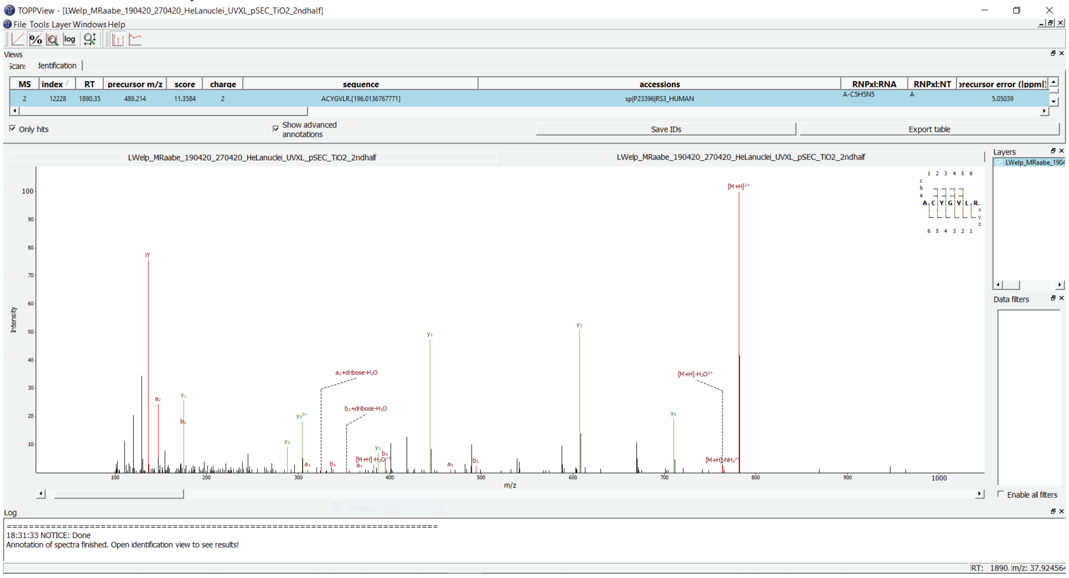

39) 40S ribosomal protein S4, X isoform

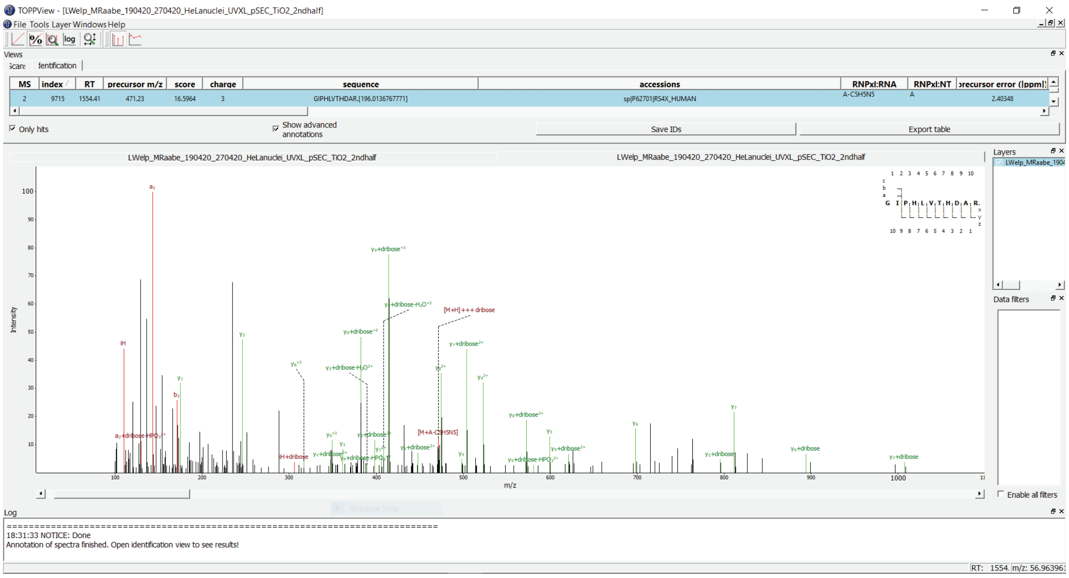

40) 40S ribosomal protein S5

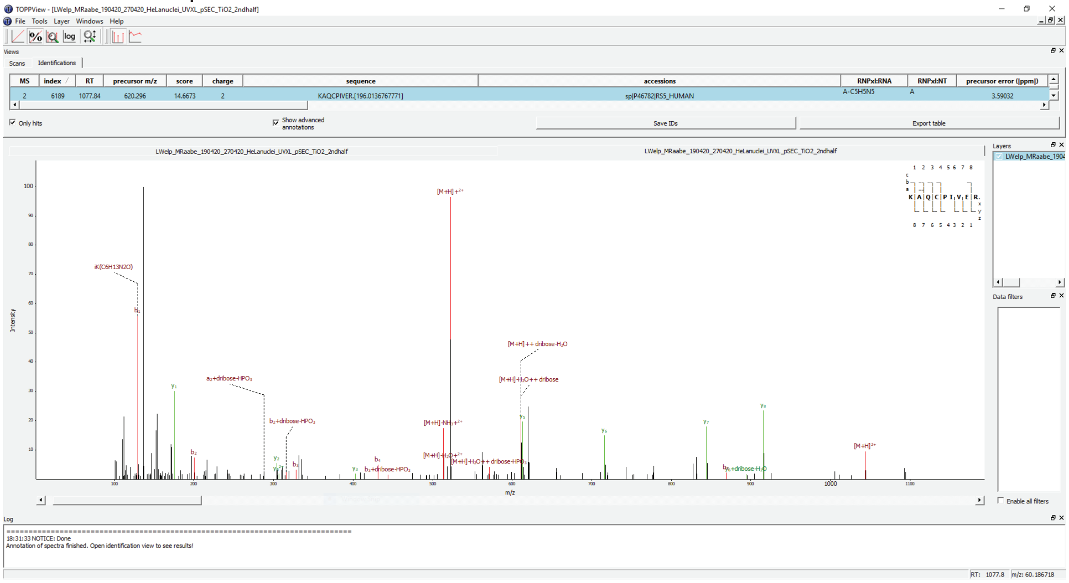

41) Tubulin alpha-3C chain

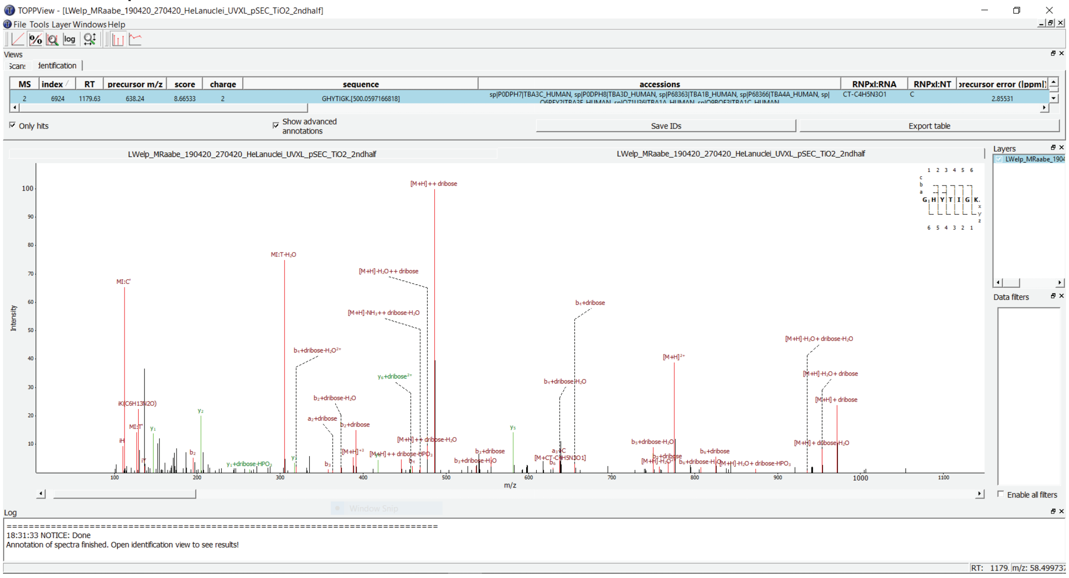

42)

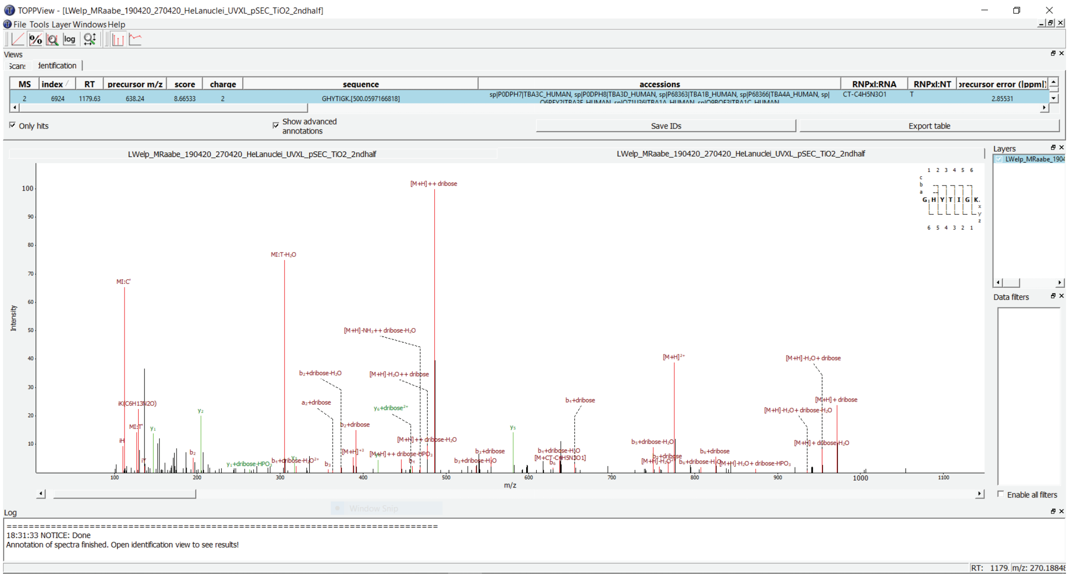

TOPPView spectra - ambiguous cross-link spectrum matches

UV cross-linked HeLa nuclei (SEC)

1) DNA (cytosine-5)-methyltransferase

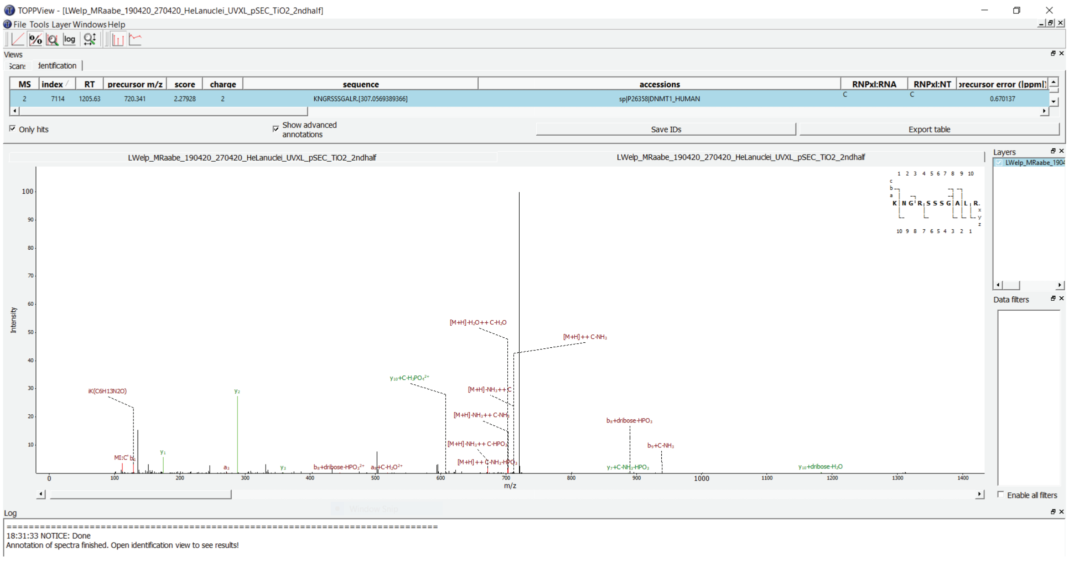

2) Endoplasmic reticulum-Golgi intermediate compartment protein 1

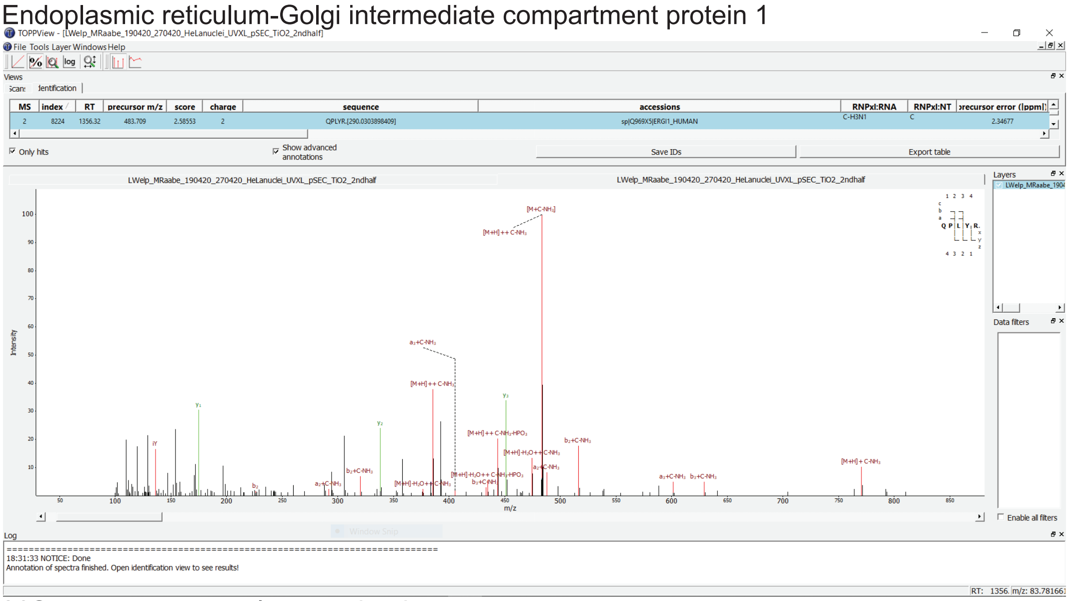

3) 26S proteasome regulatory subunit 7

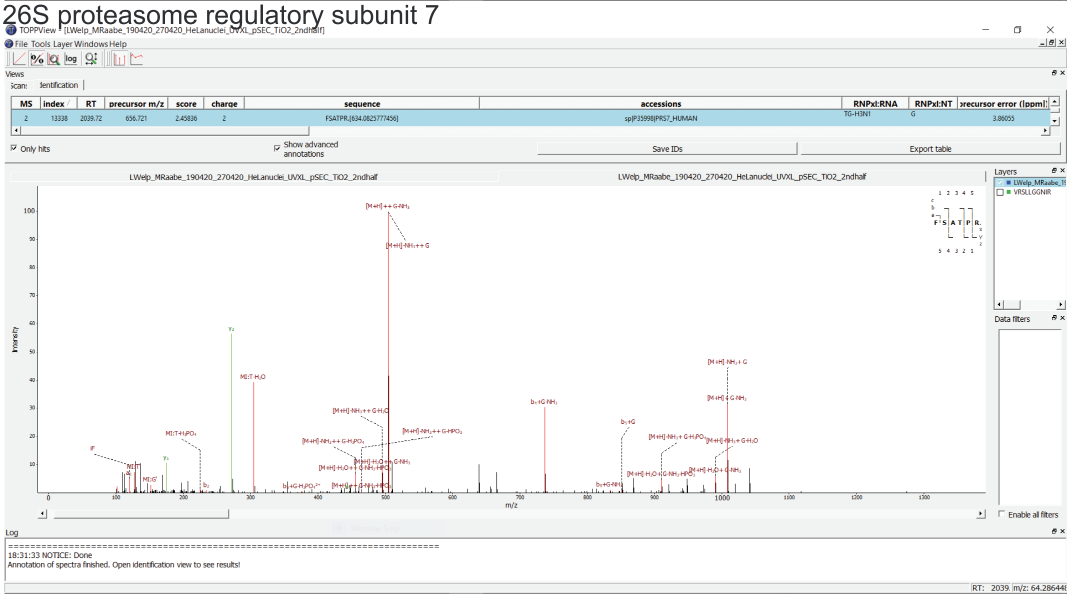

4) Structural maintenance of chromosomes protein 2

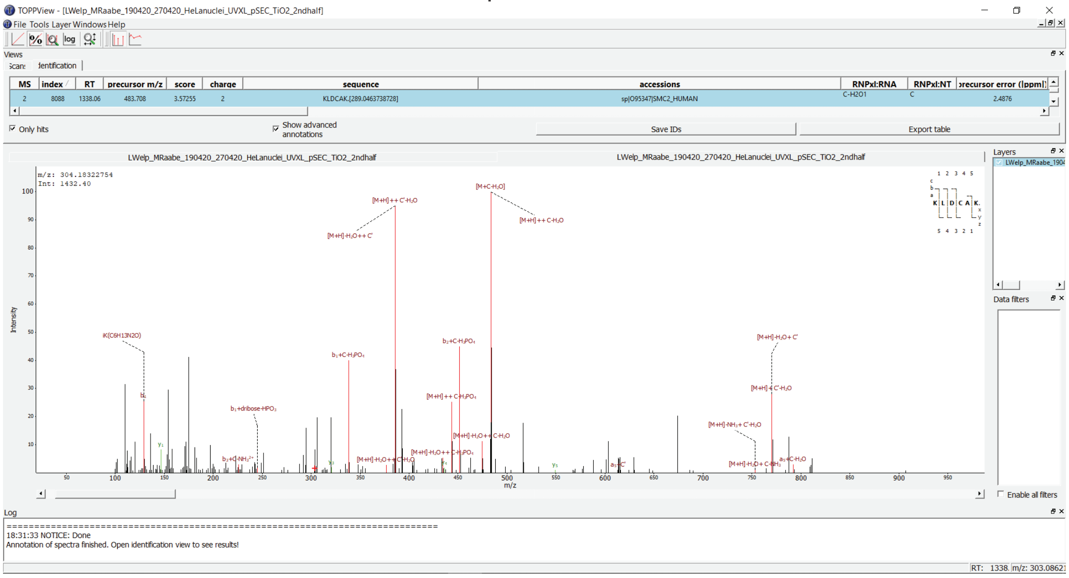

TOPPView spectra - unambiguous cross-link spectrum matches

UV cross-linked HeLa nuclei (chromatin precipiation), RNP[xl]search_settingsRNA

1) Heterogeneous nuclear ribonucleoprotein M

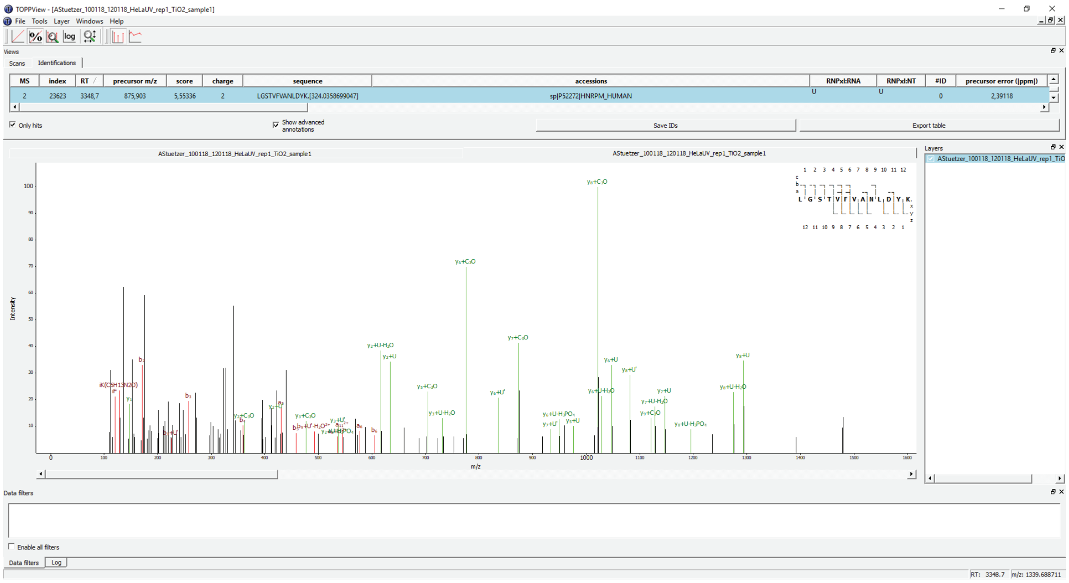

Supplement: Supplementary file 10 — Supplementary Data 8 [file 41467_2020_19047_MOESM10_ESM.pdf]
